# Difluorocarbene enables to access 2-fluoroindoles from *ortho*-vinylanilines

**DOI:** 10.1038/s41467-021-25313-z

**Published:** 2021-08-17

**Authors:** Jianke Su, Xinyuan Hu, Hua Huang, Yu Guo, Qiuling Song

**Affiliations:** 1 Institute of Next Generation Matter Transformation, College of Material Sciences Engineering, Huaqiao University, Xiamen, Fujian China; 2 Key Laboratory of Molecule Synthesis and Function Discovery, Fujian Province University, College of Chemistry at Fuzhou University, Fuzhou, Fujian China

**Keywords:** Chemical libraries, Synthetic chemistry methodology

## Abstract

2-Fluoroindoles as an important structural scaffold are widely existing in many bioactive or therapeutic agents. Despite their potential usefulness, efficient constructions of 2-fluoroindole derivatives are very sparce. The development of straightforward synthetic approaches to access 2-fluoroindoles is highly desirable for studying their fundamental properties and applications. Herein, we report an efficient and general strategy for the construction of 2-fluoroindoles in which a wide variety of 2-fluoroindoles were accessed with high efficiency and chemoselectivity. Instead of starting from indole skeletons, our strategy constructs indole scaffolds alongside the incorporation of fluorine atom on C2 position in a formal [4+1] cyclization from readily accessible *ortho*-vinylanilines and difluorocarbene. In our protocol, commercially accessible halodifluoroalkylative reagents provide one carbon and one fluorine atom by cleaving one C-N tertiary bond and forming one C-N bond and one C-C double bond with *ortho*-vinylanilines. Downstream transformations on 2-fluoroindoles lead to various valuable bioactive molecules which demonstrated significant synthetic advantages over previous reports. And mechanistic studies suggest that the reaction undergoes a cascade difluorocarbene-trapping and intramolecular Michael addition reaction followed by C*sp*^*3*^-F bond cleavage.

## Abstract (web summary)

2-Fluoroindoles are an important structural scaffold in many bioactive or therapeutic agents, but efficient constructions of 2-fluoroindole derivatives are very sparce. Here the authors report an efficient and general strategy for the construction of 2-fluoroindoles in which a wide variety of 2-fluoroindoles were accessed with high efficiency and chemoselectivity.

## Introduction

The introduction of fluorinated groups (including fluorine atom) into a target molecule would have significant influence on the parent molecule’s reactivity, selectivity, physical and biological properties^1–7^. Therefore, it becomes a critical strategy in pharmaceuticals for finding therapeutical agents^8–16^, where fluorine atom is often employed as a bioisostere of hydrogen atom based on the above features.

**Fig. 1:**
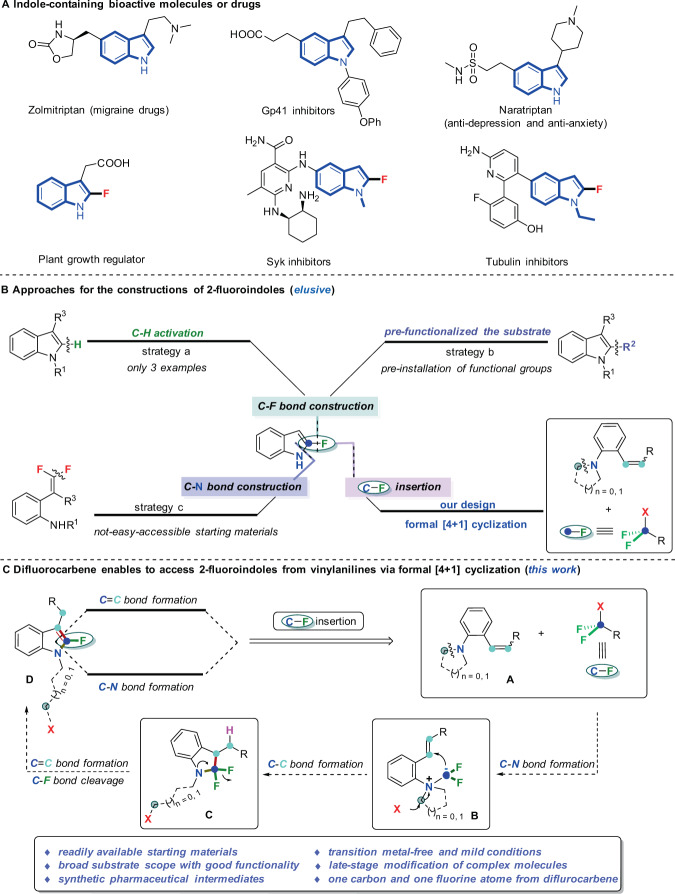
The importance and synthetic approaches to 2-fluoroindoles. **A** Importance of indoles and 2-fluoroindoles. **B** Approaches for the construction of 2-fluoroindoles. **C** Difluorocarbene enables to access 2-fluoroindoles from vinylanilines (*this work*).

Indoles, as one of most important heterocyclic scaffolds, are widespread in a broad variety of natural products^17–20^ and bioactive compounds^17,21,22^ (Fig. 1A, Top). Meanwhile, 2-fluoroindoles are also key structural motifs which are widely existing in some biologically active compounds and demonstrate unique bioactivities^23–25^(Fig. 1A, Bottom). Because of the importance of C2-fluorine-containing indoles in medicinal chemistry, great attention has been devoted to the synthesis of 2-fluoroindoles^26–35^. However, efficient synthetic methods for the construction of this type of indole derivatives are very rare. Generally, there are three ways to access the target molecules (Fig. 1B): (a) copper-mediated aminoquinoline-directed fluorination of aromatic C–H bonds. However, there were only 3 examples about 2-fluoroindole synthesis with moderate yields^26,27^, and they are limited to 3-aminoquinolineindole derivatives, which seriously limits the application of indole skeleton. (b) pre-functionalization of indoles is mandatory to install some active functional groups, such as –COOH^28^ or –SnMe_3_^29^, to C2 position of indoles for further fluorination, which require multi-step synthesis and need toxic organotin reagents, thus severely restrict the substrate scope. (c) silver or base-promoted C–N bond formation from *ortho*-amino-*gem-*difluorostyrenes to construct 2-fluororindoles^30–32^, this tactic requires complicated substrates with both *gem*-difluorostyrene and *ortho*-aromatic secondary amino functionalities, which are demanding and difficult to access, thus set a severe restriction on their applications in chemical field. Given the paucity of efficient synthetic methods and the huge challenges in the synthesis of 2-fluoroindole derivatives, the development of a convenient approach to synthesize 2-fluoroindole derivatives from simple and readily available starting materials is highly appealing and desirable.

Considering the difficulty in accessing the starting materials and limitations of substrates in the previous strategies, we wondered whether a strategy could be developed by using readily accessible starting materials in a one-step protocol. Retrosynthetic analysis of 2-fluoroindole is oriented to the *o*-alkenylanilines and a reagent which provides both C and F atoms (Fig. 1C). At this point, halodifluorinated reagents were considered due to their structural motif and their wide applications as fluorinated sources^36,37^. In addition, our recent research efforts culminated in discovery and development of several transformations featuring in situ generated difluorocarbene (:CF_2_) as C1 synthons to assemble various valuable *N*-containing compounds^38–44^. Moreover, the intramolecular capture of difluorocarbene by a thiolate and Frustrated Lewis Pairs was also developed^45,46^. Therefore, halodifluorinated reagent might be an ideal source to accomplish our goal. Inspired by our pioneering work^38^ and the previous research on interaction of tertiary amines with difluorocarbene^47–49^, and considering that difluoromethyl ammonium salt as the key intermediate from tertiary amine is readily decomposed to lead to a difluoromethyl anion in the reaction, we envisage that if a suitable electrophilic group is introduced into the substrate, difluorocarbene can be inserted into the targeted molecule via a nucleophilic attack with both carbon and fluorine atoms on, thus 2-fluoroindole framework might be resulted. There is a challenge in this hypothesis: maintenance of fluorine atom, since fluorine atoms from difluorocarbene are completely discarded and could not be reused in previous reactions, and it is an inevitable shortcoming for such transformations. To our delight, when we installed Michael reaction acceptors (MRAs)^50,51^ as electrophilic site on the substrates, a series of 2-fluoroindole derivatives were constructed smoothly (>90 examples). This strategy will open an avenue for difluorocarbene-involved transformations and will add significant synthetic values to fluorine chemistry.

Herein, we report the first general and highly efficient strategy for the synthesis of 2-fluoroindoles. This method would allow facile entry to construction of 2-fluoroindoles from simple and readily available starting materials under the mild conditions in the absence of transition-metal catalysis, and readily elaborate the late-stage modifications of pharmaceuticals and natural products with broad substrate scope and excellent functional group tolerance, and it will be a groundbreaking synthesis for 2-fluoroindole compounds, which will greatly promote the rapid development of fluorine chemistry and pharmaceutical chemistry^52–54^.

## Results

### Investigation of reaction conditions

To validate our conjecture, 3-(2-(dimethylamino)phenyl)-1-phenylprop-2-en-1-one (**1a**) and BrCF_2_COOEt (**2a**) were chosen as the model substrates using K_2_CO_3_ as base. To our delight, without any other additives in CH_3_CN, the desired 2-flurorindole product **3a** was obtained in 76% isolated yield. Replacing K_2_CO_3_ with K_3_PO_4_ resulted in a superior result (Table 1, entry 7), and further base screening indicated that K_3_PO_4_ was the best one in comparison with KOH, Cs_2_CO_3_, Na_2_CO_3_, NaOH as well as Na_3_PO_4_ (entries 2–7). Encouraged by this promising result, we further screened other solvents (THF, 1,4-dioxane, DME and toluene), and CH_3_CN was found still to be the most effective one (entries 7–11). Difluorinated reagents were subsequently examined and the results suggested that among BrCF_2_COOEt (**2a**), BrCF_2_PO(OEt)_2_ (**2b**)^55^, TMSCF_2_Br (**2c**)^56^ ClCF_2_COONa (**2d**), BrCF_2_COOK (**2e**), BrCF_2_COONa (**2f**), the best reaction efficiency was endowed by **2a** and **2b** (entries 12–16). Given the price and the ready accessibility of raw materials, we finally chose **2a** as the source of difluorocarbene (See Supplementary Tables 1–5 in Supplementary Information for details).

**Table 1:** The condition screening for our difluorocarbene-enabled 2-fluoroindole synthesis.


Entries	base	solvent	[:CF_2_]	Yield (%)^*a*^
1	K_2_CO_3_	CH_3_CN	**2a**	76^*b*^
2	KOH	CH_3_CN	**2a**	45
3	Cs_2_CO_3_	CH_3_CN	**2a**	60
4	Na_2_CO_3_	CH_3_CN	**2a**	61
5	NaOH	CH_3_CN	**2a**	trace
6	Na_3_PO_4_	CH_3_CN	**2a**	56
7	K_3_PO_4_	CH_3_CN	**2a**	93(90)^*b*^
8	K_3_PO_4_	THF	**2a**	15
9	K_3_PO_4_	1,4-dioxane	**2a**	trace
10	K_3_PO_4_	DME	**2a**	trace
11	K_3_PO_4_	toluene	**2a**	n.r.
12	K_3_PO_4_	CH_3_CN	**2b**	88
13	K_3_PO_4_	CH_3_CN	**2c**	18
14	K_3_PO_4_	CH_3_CN	**2d**	66
15	K_3_PO_4_	CH_3_CN	**2e**	49
16	K_3_PO_4_	CH_3_CN	**2** **f**	57

Reaction condition: ^*a*^**1a** (0.2 mmol), **2** (3 equiv, 0.6 mmol), base (3 equiv.), H_2_O (0.1 mL), solvent (2 mL) under 90 °C for 12 h, N_2_; GC yields; ^*b*^isolated yields; under 25 °C.

*n.r.* no reaction.

### Synthetic scope

**Fig. 2:**
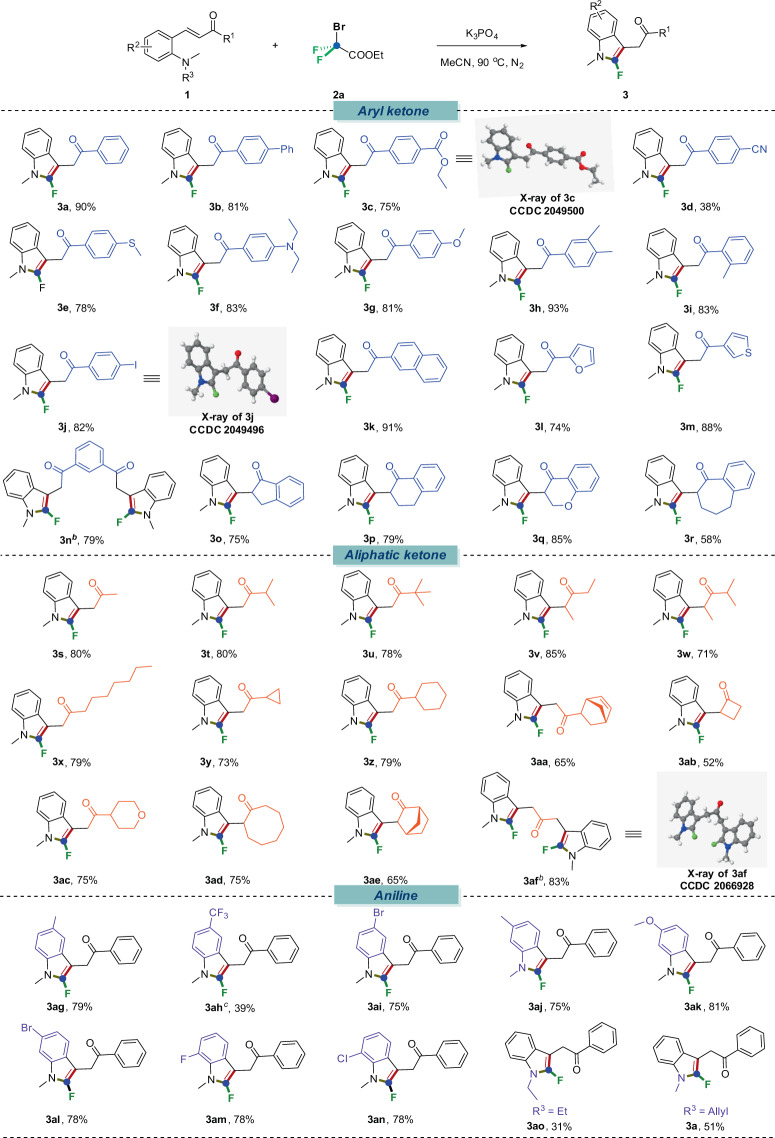
Scope of the α, β-unsaturated ketone-containing anilines^*a*^. Reaction conditions: ^*a*^**1** (0.2 mmol), **2a** (3 equiv., 0.6 mmol), K_3_PO_4_ (3 equiv.), H_2_O (0.1 mL), CH_3_CN (2 mL) under 90 °C for 12 h, N_2_. ^*b*^**1** (0.2 mmol), **2a** (6 equiv., 1.2 mmol), K_3_PO_4_ (6 equiv.), H_2_O (0.15 mL), CH_3_CN (3 mL) under 90 °C for 12 h, N_2_. ^*c*^24 h.

With the optimized conditions in hand, we systematically investigated the scope of the difluorocarbene-enabled access to 2-fluoroindoles from *ortho*-vinylanilines (Fig. 2). First, we explored the scope of α,β-unsaturated ketone moiety, which suggested that our reaction was compatible to both aryl and aliphatic α,β-unsaturated ketones. For aromatic ones, substrates bearing electron-neutral (**1a**–**1b**), electron-deficient (**1c**–**1d**), as well as electron-rich substituents (**1e**–**1g**) at the *para*-position of the aromatic rings all furnished the desired 2-fluoroindole products (**3a**–**3g**) smoothly. And with electronically neutral bis-methyl (**1h**) and *ortho*-methyl (**1i**) substrates, the corresponding products **3h**–**3i** were procured in 81 and 93% yields respectively. The accommodation of iodine substituents (**1j**) signified the further potential structural elaborations as a handle. Fused ring reactant like 1-naphthaldehyde (**1k**) was also a suitable candidate for this transformation. This study was auspiciously and effortlessly extendable to a series of heteroaromatic ketone-containing furan (**3l**) and thiophene (**3m**) cores. And for cyclic aryl ketone (**1o**–**1r**), these targeted products could be obtained successfully under the standard conditions as well (**3o**–**3r**). Notably, this protocol also featured an admirable scope with respect to aliphatic ketone substrates. No matter that it was a chain ketone or a cyclic ketone, the corresponding desired products **3s**–**3ae** were all smoothly delivered (52–80% yields), including four-membered (**3ab**), six-membered (**3ac**), eight-membered cyclic ketones (**3ad**) as well as bicyclic ketone (**3ae**). Interestingly, substrates tethered with two moieties of reaction sites (**1n**, **1af**) underwent this transformation very well to deliver the products which contain two 2-fluoroindole skeletons (**3n**, **3af**) by simple increasing the equivalent of BrCF_2_COOEt. We next surveyed the scope of R^2^ group on the aromatic ring of aniline skeleton. To our delight, a wide range of functionality was compatible under our standard conditions, and alkyl (**1ag**, **1aj**), trifluoromethyl (**1ah**), alkoxyl (**1ak**) and halo (**1ai**, **1al**–**1an**) substituted substrates were all converted into the corresponding products (**3ag**–**3an**) in moderate to excellent yields (39–81%). To assess the susceptibility of different C–N bonds towards scission, a panel of *N*-substituted tertiary amines was examined under the standard conditions. *N*-methylanilines with a different *N*-substituents (**1ao**, **1ap**) were inspected, and when the R^3^ was ethyl group, *N*-ethylindole (**3ao**) was obtained in a satisfied yield. Interestingly, when the R^3^ is allyl group, contrary to expectations, it was cleaved prior to the C_methyl_–N bond and **3a** was procured in 51% yield.

**Fig. 3:**
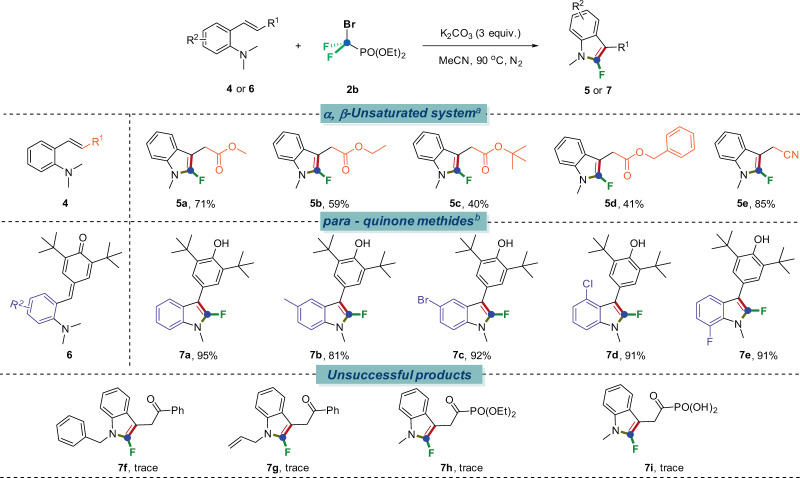
Scope of other α, β-unsaturated system-containing anilines. Reaction conditions: ^*a*^**4** (0.2 mmol), **2b** (3 equiv., 0.6 mmol), K_2_CO_3_ (3 equiv.), H_2_O (0.1 mL), CH_3_CN (2 mL) under 90 °C for 12 h, N_2_. ^*b*^**6** (0.2 mmol), **2a** (3 equiv., 0.6 mmol), K_3_PO_4_ (3 equiv.), CH_3_CN (2 mL) under 90 °C for 12 h, N_2_.

We next evaluated the scope of other Michael acceptors (Fig. 3), expectedly, these substrates were equipotent to afford the corresponding 2-fluoroindole products in moderate to excellent yields under marginally reoptimized conditions as follows: diethyl BrCF_2_PO(OEt)_2_ (**2b**) as the source of difluorocarbene, K_2_CO_3_ as the base and acetonitrile as the solvent at 90 °C for 12 h under N_2_ atmosphere (See Supplementary Tables 6–7 in Supplementary Information for details).

We then first explored *α, β*-unsaturated esters^50,51^. In addition to methyl acrylates, ethyl, *tert*-butyl and benzyl acrylates were also competent substrates to give the corresponding products (**5a**–**5d**) in moderate yields. Moreover, this system could also enable the synthesis of 2-fluoroindole scaffolds with other Michael acceptors such as acrylonitrile (**4e**) and *para*-quinone methides^57^ (**6a**–**6e**) with good efficiency, generating functional products (**5e**, **7a**–**7e)** that are highly valuable structural motifs in medicinal chemistry. In order to further prove the universality of this reaction, we introduced other alkyl groups (like benzyl and allyl group) as substituents on *N*-atom, and also tried phosphonates (including phosphonic acid) as electron withdrawing groups. However, only trace amount of the corresponding products (**7f**–**7i)** was detected. We then tried several other difluoroalkylating reagents which could serve as the sources of difluorocarbene, but no good results were obtained.

**Fig. 4:**
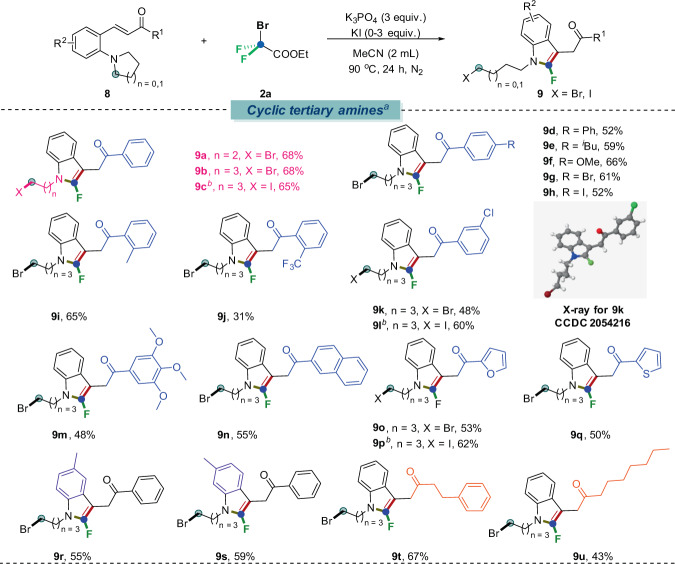
Scope of cyclic tertiary anilines. Reaction conditions: ^*a*^**8** (0.2 mmol), **2a** (3 equiv., 0.6 mmol), K_3_PO_4_ (3 equiv.), H_2_O (0.1 mL), CH_3_CN (2 mL) under 90 °C for 24 h, N_2_. ^*b*^**8** (0.2 mmol), **2a** (3 equiv., 0.6 mmol), KI (3 equiv.), K_3_PO_4_ (3 equiv.), H_2_O (0.1 mL), CH_3_CN (2 mL) under 90 °C for 24 h, N_2_.

We also paid our attention to the substrates with different cyclic tertiary amines, if successful, *N*-tethered long chain aliphatic halides would be rendered, which would add more values for further structural elaborations as a functional handle. The results were summarized in Fig. 4. It turned out that both four-membered (**8a**) and five-membered (**8b**–**8u**) cyclic tertiary amines were well-suited for this transformation, exhibiting insensitivity toward aryl substitutions (alkyl, alkoxyl, phenyl, halo, fused, thiophene and furan etc.). The corresponding target products (**9a**–**9u**) were procured in moderate yields. Remarkably, when KI as a nucleophile was added into the reaction system, the *N*-aliphatic-tethered-iodinated-2-fluoroindoles were obtained (**9c**, **9l**, **9p**) in decent yields accordingly.

**Fig. 5:**
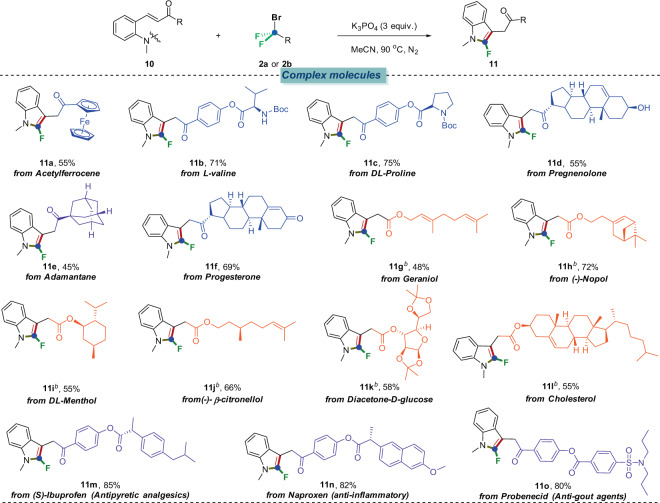
Scope of complex molecules^*a*^. Reaction conditions: ^*a*^**10** (0.2 mmol), **2a** (3 equiv., 0.6 mmol), K_3_PO_4_ (3 equiv.), H_2_O (0.1 mL), CH_3_CN (2 mL) under 90 °C for 12 h, N_2_. ^*b*^**10** (0.2 mmol), **2b** (3 equiv., 0.6 mmol), K_2_CO_3_ (3 equiv.), CH_3_CN (2 mL) under 90 °C for 12 h, N_2_.

The above results clearly demonstrated that our method has a broad substrate scope and wide functional group compatibility, which further prompted our endeavors to extrapolate this strategy to late-stage modifications of bioactive molecules and therapeutic agents (Fig. 5). Gratifyingly, a series of bioactive molecules (Acetylferrocene, L-valine, DL-proline, Pregnenolone, Adamantane, Progesterone, Geraniol, (-)-Nopol, DL-Menthol, (-)-β-citronellol, Geraniol, Cholesterol) were all derivatized into the corresponding Michael acceptors and installed into our substrates (**10a**–**o**) which, upon treatment with BrCF_2_COOEt (**2a**) or BrCF_2_PO(OEt)_2_ (**2b**) under the established standard conditions, were all smoothly incorporated into the eventual 2-fluoroindole derivatives (**11a**–**o**). Moreover, drug compounds, such as (s)-Ibuprofen (**10m**, Antipyretic analgesics), Naproxen (**10n**, Anti-inflammatory), Probenecid (**10o**, Anti-gout agents) were also successfully introduced into the corresponding 2-fluoroindole derivatives (**11m**–**o**) without loss of efficiency. By combining the 2-fluoroindole skeleton and bioactive structural motifs together, it was thus envisioned that our system would simplify access to discover more potential bioactive molecules.

### Synthetic application

**Fig. 6:**
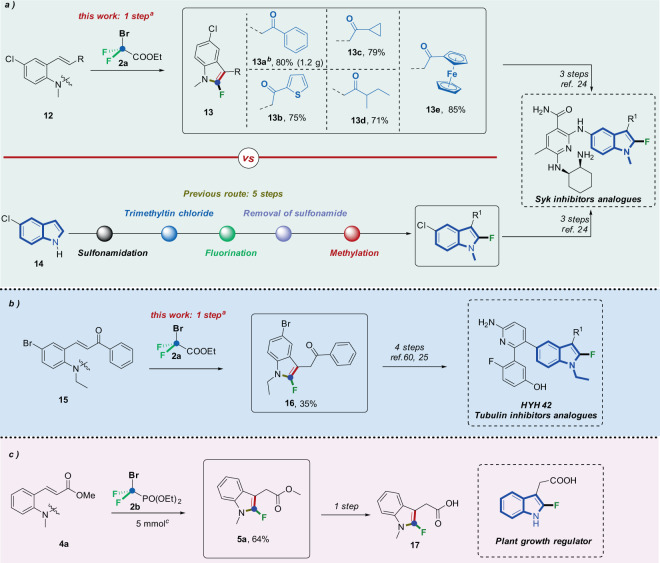
Synthetic applications. Reaction conditions: ^*a*^**12** or **15** (0.2 mmol), **2a** (3 equiv., 0.6 mmol), K_3_PO_4_ (3 equiv.), H_2_O (0.1 mL), CH_3_CN (2 mL) under 90 °C for 12 h, N_2_. ^*b*^**12a** (5 mmol), **2a** (3 equiv., 15 mmol), K_3_PO_4_ (3 equiv.), H_2_O (1.5 mL), CH_3_CN (25 mL) under 90 °C for 24 h, N_2_. ^*c*^**4a** (5 mmol), **2b** (3 equiv., 15 mmol), K_2_CO_3_ (3 equiv.), H_2_O (1.5 mL), CH_3_CN (25 mL) under 90 °C for 24 h, N_2_.

To further demonstrate the synthetic utility of our strategy, we chose 5-chloro-*N*-methyl-2-fluoroindoles (**13**), the key intermediate for the synthesis of Syk (Spleen tyrosine kinase) inhibitors drug, as the target molecule (Fig. 6a). This reaction could be readily scaled-up from substrate **12a** to 5 mmol without loss of efficiency (**13a**, 80% yield). Of note, according to the existing reports^24^, five-step synthesis was required for the construction of 5-chloro-*N*-methyl-2-fluoroindoles (**13**): (1) protection of N-H in indole with tosyl chloride, (2) trimethyltin was installed to activate indole by Sn(Me)_3_Cl, (3) fluorination with selectfluor, (4) removal of sufonamide by KOH, (5) dimethyl sulfate was introduced into the product as a methylation reagent. And for the starting material **12**, it can be obtained by a sequential aromatic nucleophilic substitution between *ortho*-fluorobenzaldehyes and dimethylamines and aldol condensation between the aforementioned products and ketones. The final product (the starting material **12** for our reaction) could be obtained in the two-step synthesis with a total yield over 90%. And the price of the substrates is very cheap (*ortho*-fluorobenzaldehye: 1 $/g, dimethylamine: 0.03 $/g). Compared with the previous method, our route not only significantly decreases the step count (1 step vs. 5 steps), but also could avoid the use of strong base (KOH and NaH), toxic organotin reagents and dimethyl sulfate^58,59^. Meanwhile, our strategy could introduce very valuable functional groups in one step on C3 position of indoles as well. HYH42 is a highly specific inhibitor for human non-small cell lung cancer cells (NCI-H460, IC50 < 1 μM) which is commonly used in pharmaceutical research^25^. Remarkably, starting from readily accessible *ortho*-vinylaniline **15**, our reaction enabled us to access the key intermediate **16** in a single step with a yield of 35% through the direct 2-fluoroindolylation reaction, then HYH42 can be obtained by coupling^25,60^ from the key intermediate **16** (Fig. 6b). Moreover, we utilized our methodology on the formal synthesis of **5a**, which is the key intermediate of a potential plant growth hormone regulated transcription factor^23^, and the reaction could be scaled up to 5 mmol and the targeted product **5a** was obtained in 64% yield (Fig. 6c). Of note, there is no other efficient report on its synthesis prior to our method.

### Mechanistic studies

**Fig. 7:**
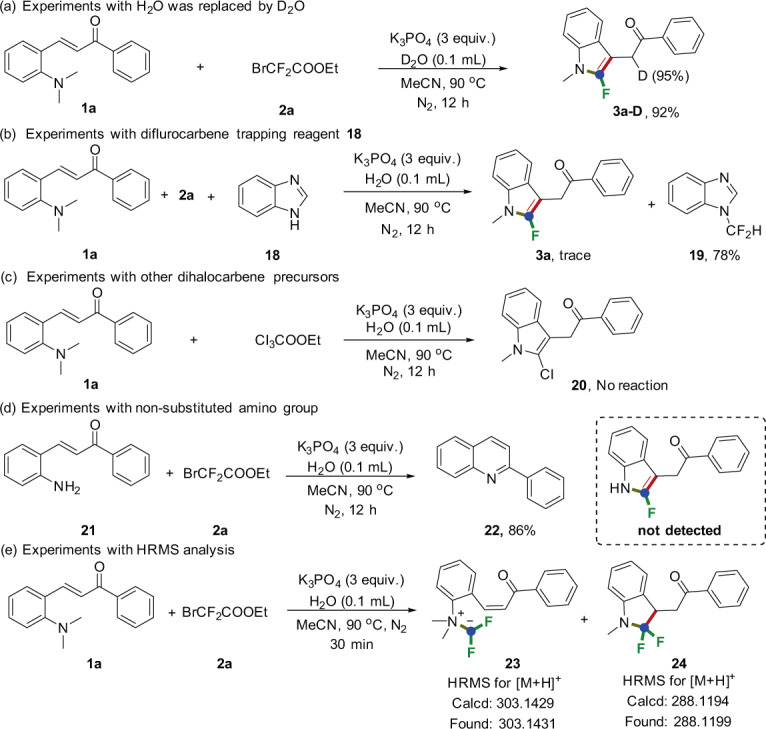
Control experiments. **a** Deuterium-labeling experiment. **b** Diflurocarbene trapping. **c** Control experiment of dichlorocarbene. **d** Experiments with non-substituted amino group. **e** Experiments with HRMS analysis.

Intrigued by the features of the presented methodology, we next conducted several control experiments to shed light on the reaction mechanism (Fig. 7). When water was replaced by deuterium oxide, one deuterium atom was incorporated at α-position of carbonyl group of the final product **3a-D** with 95% deuterization rate in the crude mixture (Fig. 7, eq. a). When diflurocarbene trapping reagent, namely, benzimidazole (**18**), were added individually into this system, 1-(difluoromethyl)-1H-benzo[d]imidazole (**19**) was obtained in 78% yield alongside a trace amount of **3a**, suggesting the existence of difluorocarbene species (Fig. 7, eq. b). We then considered whether other dihalocarbene species (:CCl_2_) are compatible with the reactions, of note, no desired products **20** were detected, which indicates that difluorocarbene species has some special characteristics, which isn’t possessed by other dihalocarbenes (Fig. 7, eq. c). In order to indicate the essence of tertiary amino group on the substrate, primary amine **21** was subjected to the reaction conditions, not surprisingly, no 2-fluoroindole formation was detected owing to the rapid cyclization to render 2-phenylquinoline **22** (Fig. 7, eq. d). Moreover, the HRMS (high resolution mass spectrometry) analysis of the mixture of the standard reaction for 30 min showed the two peaks at m/z = 303.1431 and m/z = 288.1199, which matched the ionic compound form possible intermediates **23** and **24** (calcd mass: 303.1429 and 288.1194) (Fig. 7, eq. e).

### Proposed mechanism

**Fig. 8:**
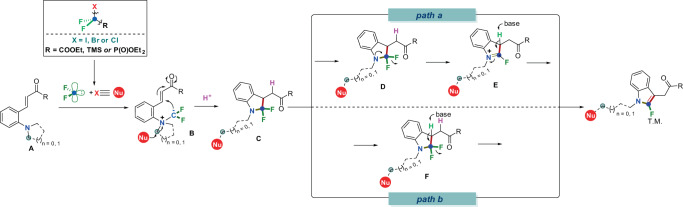
Plausible mechanism. Possible reaction mechanism of difluorocarbene enables to access 2-fluoroindoles from *ortho*-vinylanilines.

On the basis of the above results, a plausible mechanism is proposed as depicted in Fig. 8. Tertiary amine **A** reacts with the in situ generated difluorocarbene (:CF_2_) species which is unmasked from halodifluoroalkyl reagents (BrCF_2_COOEt (**2a**) or BrCF_2_PO(OEt)_2_ (**2b**)) in the presence of the base to deliver ammonium salt **B**. Internal nucleophile (X−) attacks the α carbon of ammonium salt **B** to break the C–N bond under mild and transition-metal free and oxidant-free conditions^38,47^. The newly formed difluoromethyl anion further attacks unsaturated double bond intramolecularly to render intermediate **C** via Michael addition and protonation. According to our previous work, the CF_2_ group on the nitrogen atom is unstable and readily to undertake C–F cleavage under the action of lone pair electrons of the nitrogen atom. Meanwhile, the hydrogen atom adjacent to CF_2_ is base-sensitive and easy to be deprotonated, thus a proposed E2cb mechanism is also possible. Based on the above discussion, there might be two possible paths to render products. One possible path is the C*sp*^*3*^–F bond adjacent to N atom is vulnerable in basic conditions to lead to intermediate **D** via a C*sp*^*3*^–F bond cleavage. Finally, with the participation of base, the final product is produced by the driving force to reconstruct the aromatic system (path a). Another way for the formation of the final product is caused by a E2cb pathway with the loss of H-F under basic conditions, once again, the driving force should be the reconstruction of the aromatic ring—indoles (path b).

In summary, we have reported a straightforward synthesis of 2-fluoroindoles from readily accessible *ortho*-vinylanilines with halodifluoroalkylative reagents as difluorocarbene sources, which provide one carbon atom and one fluorine atom. This general and highly selective transformation provides operationally simple and robust access to versatile 2-fluoroindoles under a mild condition without any transition metals or oxidants. The products obtained can further engage in various derivatizations where the 2-fluoroindole motif functions as an ideal drug skeleton, enabling a fast and orthogonal transformation to many useful building blocks. In a broader context, these features emphasize the value of the presented methodology for the versatile synthesis of 2-fluoroindoles derivatives which are ubiquitously found in bioactive molecules. Further investigations to extend the reaction scope and applications of this process are currently in progress.

## Methods

### General procedure for synthesis of 2-fluoroindoles from chalcones or *p*-Quinone Methide

In air, chalcones or *p*-Quinone Methide (0.2 mmol) and K_3_PO_4_ (3 eq, 0.6 mmol) were added to a Schlenk tube equipped with a stir bar. The vessel was evacuated and filled with N_2_ (three cycles). BrCF_2_COOEt (3 eq, 0.6 mmol), H_2_O (0.1 mL) and CH_3_CN (2 mL) added in turn by syringe under N_2_ atmosphere. The resulting reaction mixture was stirred vigorously at 90 °C for 12 h. Upon completion of the reaction, the solvent was evaporated under reduced pressure and the residue was purified by flash column chromatography to give the desired products.

### General procedure for synthesis of 2-fluoroindoles from *α*, *β*-unsaturated esters or acrylonitrile

In air, *α, β*-unsaturated esters (0.2 mmol) or acrylonitrile and K_2_CO_3_ (3 eq, 0.6 mmol) were added to a Schlenk tube equipped with a stir bar. The vessel was evacuated and filled with N_2_ (three cycles). BrCF_2_PO(OEt)_2_ (3 eq, 0.6 mmol), H_2_O (0.1 mL) and CH_3_CN (2 mL) added in turn by syringe under N_2_ atmosphere. The resulting reaction mixture was stirred vigorously at 90 °C for 12 h. Upon completion of the reaction, the solvent was evaporated under reduced pressure and the residue was purified by flash column chromatography to give the desired products.

### General procedure for synthesis of *N*-tethered long chain aliphatic bromine

In air, cyclic tertiary amines (0.2 mmol) and K_3_PO_4_ (3 eq, 0.6 mmol) were added to a Schlenk tube equipped with a stir bar. The vessel was evacuated and filled with N_2_ (three cycles). BrCF_2_COOEt (3 eq, 0.6 mmol), H_2_O (0.1 mL) and CH_3_CN (2 mL) added in turn by syringe under N_2_ atmosphere. The resulting reaction mixture was stirred vigorously at 90 °C for 24 h. Upon completion of the reaction, the solvent was evaporated under reduced pressure and the residue was purified by flash column chromatography to give the desired products.

### General procedure for synthesis of *N*-tethered long chain aliphatic iodine

In air, cyclic tertiary amines (0.2 mmol), KI (3 eq, 0.2 mmol) and K_3_PO_4_ (3 eq, 0.6 mmol) were added to a Schlenk tube equipped with a stir bar. The vessel was evacuated and filled with N_2_ (three cycles). BrCF_2_COOEt (3 eq, 0.6 mmol), H_2_O (0.1 mL) and CH_3_CN (2 mL) added in turn by syringe under N_2_ atmosphere. The resulting reaction mixture was stirred vigorously at 90 °C for 24 h. Upon completion of the reaction, the solvent was evaporated under reduced pressure and the residue was purified by flash column chromatography to give the desired products.

## Supplementary information

Suppplementary information

## Data Availability

The data that support the findings of this study are available within the article and its Supplementary Information files. The X-ray crystallographic coordinates for structures reported in this article have been deposited at the Cambridge Crystallographic Data Centre (CCDC), under deposition number 2049500 (**3c**), 2049496 (**3j**), 2066928 (**3af**) and 2054216 (**9k)**. The data can be obtained free of charge from The Cambridge Crystallographic Data Centre via http:// www.ccdc.cam.ac.uk/data_request/cif.
