# Supplementary material for: Difluorocarbene enables to access 2-fluoroindoles from ortho-vinylanilines
Source: Nat Commun. 2021 Aug 17;12:4986. doi: 10.1038/s41467-021-25313-z (PMC8371155; doi:10.1038/s41467-021-25313-z)
Supplement: Supplementary file 1 — Suppplementary information [file 41467_2021_25313_MOESM1_ESM.pdf]

**Supplementary Information**

***For***

# Difluorocarbene enables to access 2-fluoroindoles from *ortho*-vinylanilines

Jianke Su,[1] Xinyuan Hu,[1] Hua Huang,[1] Yu Guo[1] and Qiuling Song*[1, 2]

[1] *Institute of Next Generation Matter Transformation, College of Material Sciences Engineering, Huaqiao University, Xiamen, Fujian, 361021, China*

[2] *Key Laboratory of Molecule Synthesis and Function Discovery, Fujian Province University, College of Chemistry at Fuzhou University, Fuzhou, Fujian, 350108, China*

**email: qsong@hqu.edu.cn*

# Table of Contents


1. Supplementary Methods ........................................................................................ 4
   1.1 General information ........................................................................................ 4
   1.2 Synthesis of starting materials ........................................................................ 5
2. Supplementary Discussion...................................................................................... 10
   2.1 Optimization studies ...................................................................................... 10
   2.2 Control experiments ...................................................................................... 15
   2.3 Crystal data..................................................................................................... 18
   2.4 Characterization data for products.................................................................. 22
   2.5 NMR spectroscopic data ................................................................................ 105
   2.6 Supplementary References ............................................................................ 393

# 1. Supplementary Methods

## 1.1 General information

All chemicals were purchased from Adamas Reagent, Energy chemical company ($BrCF_2COOEt$, $BrCF_2PO(OEt)_2$, $ClCF_2COONa$), Bide Pharmatech Ltd ($TMSCF_2Br$,), J&K SCIENTIFIC LTD ($ICF_2COOEt$)and Shang Fluoro Company ($ClCF_2H$). Unless otherwise stated, all experiments were conducted in a sealed tube under $N_2$ atmosphere. Reactions were monitored by TLC or GC-MS analysis. Flash column chromatography was performed over silica gel (200-300 mesh).

$^{1}$H-NMR and $^{13}$C-NMR spectra were recorded in $CDCl_3$ and DMSO-$d_6$ on a Bruker Avance 500 spectrometer (500 MHz $^{1}$H, 125 MHz $^{13}$C (CPD), 470 MHz $^{9}$F) at room temperature. Chemical shifts were reported in ppm on the scale relative to $CDCl_3$ ($\delta$ = 7.26 for $^{1}$H-NMR, $\delta$ = 77.00 for $^{13}$C-NMR) as an internal reference. Coupling constants (*J*) were reported in Hertz (Hz).

## 1.2 Synthesis of starting materials

### 1.2.1 General process 1：Preparation of chalcones (1)[1]

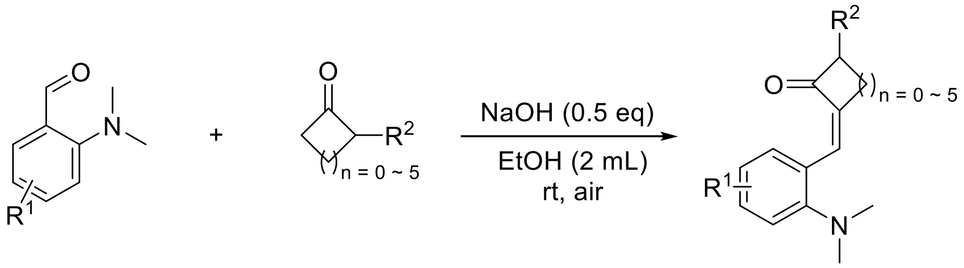


NaOH (75 mg, 0.5 equiv) was added under stirring to a solution of ketone (3 mmol, 1 equiv) and 2-(dimethylamino)benzaldehyde (3 mmol, 1 equiv) in ethanol (2 mL). The mixture was stirred for 24 h at room temperature. After, the reaction mixture was neutralized with HCl 5% until pH ≈ 7 and extracted with ethyl acetate (3 × 30 mL). Then, the organic layer was dried, concentrated, and purified by flash column chromatography (silica gel, petroleum ether: EtOAc =30:1, v/v) to give the desired products.

### 1.2.2 General process 2：Preparation of *α, β*-unsaturated esters (4a - 4d)[2]

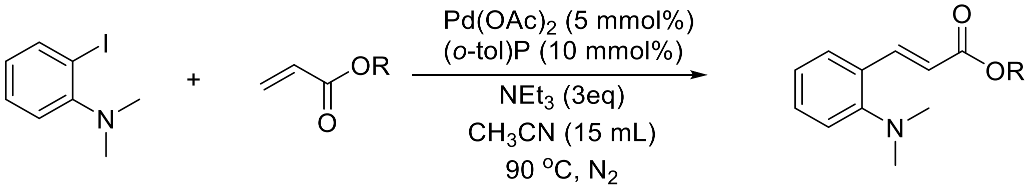


In air, Pd(OAc) (5 mol%, 36.6 mg) and tri(o-tolyl)phosphine (10 mol%, 91.2 mg) were added to a Schlenk tube equipped with a stir bar. The vessel was evacuated and filled with $N_2$ (three cycles). $NEt_3$ (3 eq, 909.6 mg), 2-iodo-*N, N*-dimethylaniline (3 mmol, 738 mg) acrylate (1.2 eq, 3.6 mmol) and $CH_3CN$ (15 mL) added in turn by syringe under $N_2$ atmosphere. The resulting reaction mixture was stirred vigorously at 90 ℃ for 12 h. Upon completion of the reaction, the solvent was evaporated under reduced pressure and the residue was purified by flash column chromatography (silica gel, petroleum ether: EtOAc =40:1, v/v) to give the desired products.

### 1.2.3 General process 3: Preparation of acrylonitrile (4e)[2]

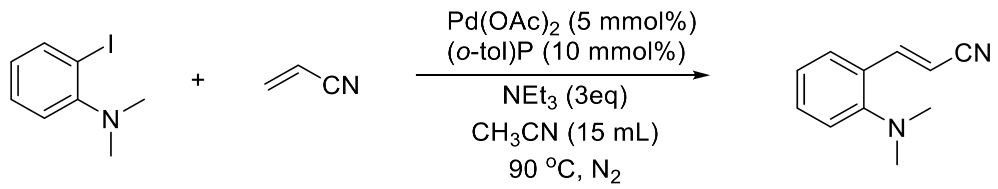


In air, Pd(OAc) (5 mol%, 36.6 mg) and tri(o-tolyl)phosphine (10 mol%, 91.2 mg) were added to a Schlenk tube equipped with a stir bar. The vessel was evacuated and filled with $N_2$ (three cycles). $NEt_3$ (3 eq, 9 mmol, 909.6 mg), 2-iodo-*N*, *N*-dimethylaniline (3 mmol, 738 mg), acrylonitrile (1.2 eq, 3.6 mmol) and $CH_3CN$ (15 mL) added in turn by syringe under $N_2$ atmosphere. The resulting reaction mixture was stirred vigorously at 90 °C for 12 h. Upon completion of the reaction, the solvent was evaporated under reduced pressure and the residue was purified by flash column chromatography (silica gel, petroleum ether: EtOAc =40:1, v/v) to give the desired products.

### 1.2.4 General process 4: Preparation of *p*-Quinone Methide Substrates (6a - 6e)[3]

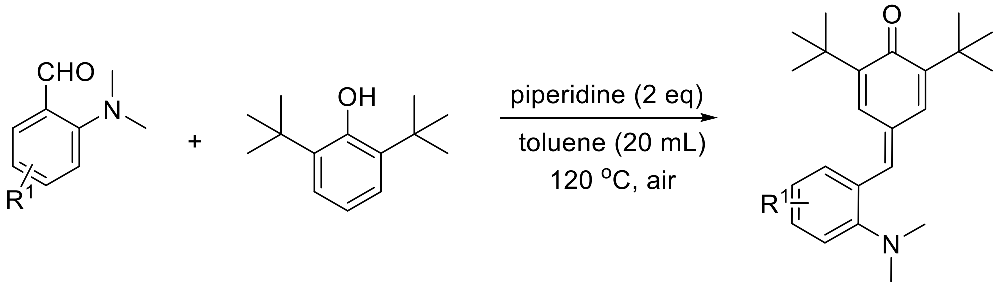


A mixture of 2-(dimethylamino)benzaldehyde (5 mmol,) 2,6-di-*tert*-butylphenol (1.3 equiv, 6.5 mmol,) and piperidine (2 equiv, 10 mmol) in toluene (20 mL) was stirred for 120 °C for 8 h. Upon completion of the reaction, the solvent was evaporated under reduced pressure and the residue was purified by flash column chromatography (silica gel, petroleum ether: EtOAc =40:1, v/v) to give the desired products.

## 1.3 Synthesis of 2-fluoroindoles

### 1.3.1 General process 5: For synthesis of 2-fluoroindoles from chalcones or *p*-Quinone Methide

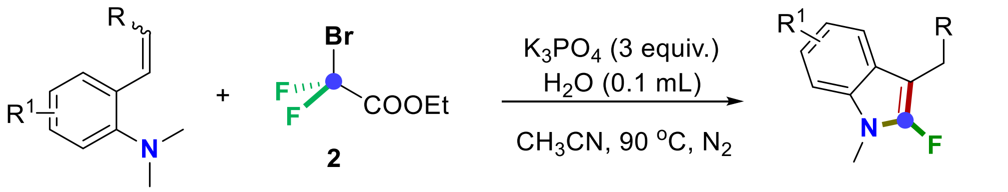


In air, chalcones or *p*-Quinone Methide (0.2 mmol) and $K_3PO_4$ (3 eq, 0.6 mmol) were added to a Schlenk tube equipped with a stir bar. The vessel was evacuated and filled with $N_2$ (three cycles). $BrCF_2COOEt$ (3 eq, 0.6 mmol), $H_2O$ (0.1 mL) and $CH_3CN$ (2 mL) added in turn by syringe under $N_2$ atmosphere. The resulting reaction mixture was stirred vigorously at 90 °C for 12 h. Upon completion of the reaction, the solvent was evaporated under reduced pressure and the residue was purified by flash column chromatography (silica gel, petroleum ether: $CH_2Cl_2$ =10:1, v/v) to give the desired products.

### 1.3.2 General process 6: For synthesis of 2-fluoroindoles from *α, β*-unsaturated esters or acrylonitrile

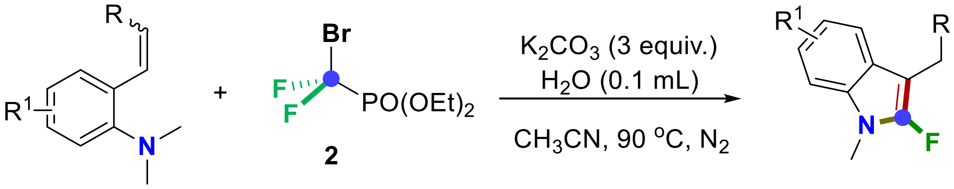


In air, *α, β*-unsaturated esters (0.2 mmol) or acrylonitrile and $K_2CO_3$ (3 eq, 0.6 mmol) were added to a Schlenk tube equipped with a stir bar. The vessel was evacuated and filled with $N_2$ (three cycles). $BrCF_2PO(OEt)_2$ (3 eq, 0.6 mmol), $H_2O$ (0.1 mL) and $CH_3CN$ (2 mL) added in turn by syringe under $N_2$ atmosphere. The resulting reaction mixture was stirred vigorously at 90 °C for 12 h. Upon completion of the reaction, the solvent was evaporated under reduced pressure and the residue was purified by flash column chromatography (silica gel, petroleum ether: $CH_2Cl_2$ =10:1, v/v) to give the desired products.

### 1.3.3 General process 7: For synthesis of *N*-tethered long chain aliphatic bromine

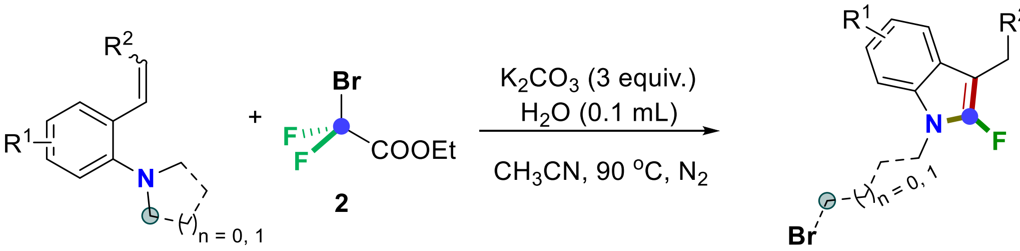


In air, cyclic tertiary amines (0.2 mmol) and $K_3PO_4$ (3 eq, 0.6 mmol) were added to a Schlenk tube equipped with a stir bar. The vessel was evacuated and filled with $N_2$ (three cycles). $BrCF_2COOEt$ (3 eq, 0.6 mmol), $H_2O$ (0.1 mL) and $CH_3CN$ (2 mL) added in turn by syringe under $N_2$ atmosphere. The resulting reaction mixture was stirred vigorously at 90 °C for 24 h. Upon completion of the reaction, the solvent was evaporated under reduced pressure and the residue was purified by flash column chromatography (silica gel, petroleum ether: $CH_2Cl_2$ =10:1, v/v) to give the desired products.

### 1.3.4 General process 8: For synthesis of *N*-tethered long chain aliphatic iodine

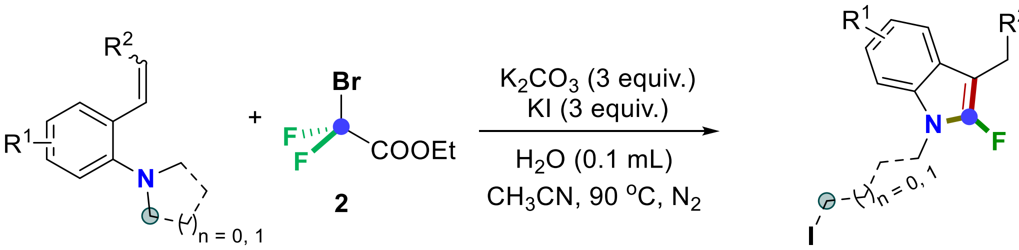


In air, cyclic tertiary amines (0.2 mmol), KI (3 eq, 0.2 mmol) and $K_3PO_4$ (3 eq, 0.6 mmol) were added to a Schlenk tube equipped with a stir bar. The vessel was evacuated and filled with $N_2$ (three cycles). $BrCF_2COOEt$ (3 eq, 0.6 mmol), $H_2O$ (0.1 mL) and $CH_3CN$ (2 mL) added in turn by syringe under $N_2$ atmosphere. The resulting reaction mixture was stirred vigorously at 90 °C for 24 h. Upon completion of the reaction, the solvent was evaporated under reduced pressure and the residue was purified by flash column chromatography (silica gel, petroleum ether: $CH_2Cl_2$ =10:1, v/v) to give the desired products.

### 1.3.5 General process 9：For synthesis of 13a

In air, chalcones **12a** (5 mmol) and $K_3PO_4$ (3 eq, 15 mmol) were added to a Schlenk tube equipped with a stir bar. The vessel was evacuated and filled with $N_2$ (three cycles). $BrCF_2COOEt$ (3 eq, 15 mmol), $H_2O$ (1.5 mL) and $CH_3CN$ (25 mL) added in turn by syringe under $N_2$ atmosphere. The resulting reaction mixture was stirred vigorously at 90 ºC for 24 h. Upon completion of the reaction, the solvent was evaporated under reduced pressure and the residue was purified by flash column chromatography (silica gel, petroleum ether: $CH_2Cl_2$ =10:1, v/v) to give the desired products.

### 1.3.6 General process 10：For synthesis of 5a

In air, *α, β*-unsaturated esters **4a** (5 mmol) and $K_2CO_3$ (3 eq, 15 mmol) were added to a Schlenk tube equipped with a stir bar. The vessel was evacuated and filled with $N_2$ (three cycles). $BrCF_2PO(OEt)_2$ (3 eq, 15 mmol), $H_2O$ (1.5 mL) and $CH_3CN$ (25 mL) added in turn by syringe under $N_2$ atmosphere. The resulting reaction mixture was stirred vigorously at 90 ºC for 24 h. Upon completion of the reaction, the solvent was evaporated under reduced pressure and the residue was purified by flash column chromatography (silica gel, petroleum ether: $CH_2Cl_2$ =10:1, v/v) to give the desired products.

# 2. Supplementary Discussion

## 2.1 Optimization studies

### 2.1.1 Optimization of the reaction conditions of the synthesis of 2-fluoroindole (3a)

**Supplementary Table 1. The effects of base**

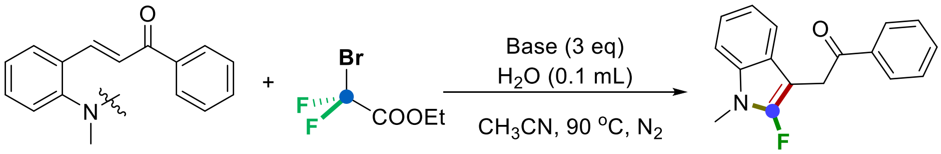


**1a** **2a** **3a**

| Entry | Base (3 equiv) | Yield (%)[a] |
| --- | --- | --- |
| 1 | $K_2CO_3$ | 76 |
| 2 | KOH | 45 |
| 3 | $Cs_2CO_3$ | 60 |
| 4 | $Na_2CO_3$ | 61 |
| 5 | NaOH | Trace |
| 6 | $Na_3PO_4$ | 56 |
| 7 | HCOOK | Trace |
| 8 | $^t$BuCOOK | Trace |
| 9 | $CH_3COOK$ | Trace |
| 10 | $^t$BuOK | 52 |
| 11 | $KHF_2$ | 18 |
| 12 | $K_3PO_4$ | 93(90)[b] |
| 13 | $K_3PO_4$ (1.5 eq) | 46 |
| 14 | $K_3PO_4$ (2 eq) | 61 |
| 15 | $K_3PO_4$ (2.5 eq) | 79 |
| 16 | $K_3PO_4$ (3.5 eq) | 83 |

[a] Reaction condition : **1a** (0.2 mmol), **2a** (3 equiv, 0.6 mmol), base (x equiv.), $H_2O$ (0.1 mL), $CH_3CN$ (2 mL) under 90 °C for 12 h, $N_2$ ; GC yields; [b] isolated yields.

**Supplementary Table 2. The effects of $H_2O$**

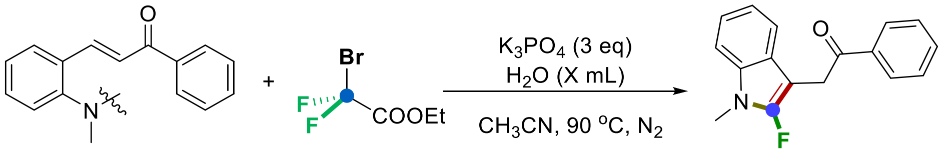


**1a** **2a** **3a**

| Entry | $H_2O$ (X mL) | Yield (%)[a] |
| --- | --- | --- |
| 1 | 0.01 | 79 |
| 2 | 0.05 | 84 |
| 3 | 0.1 | 93(90)[b] |
| 4 | 0.3 | 90 |

[a] Reaction condition : **1a** (0.2 mmol), **2a** (3 equiv, 0.6 mmol), $K_3PO_4$ (3 equiv.), $H_2O$ (X mL), $CH_3CN$ (2 mL) under 90 °C for 12 h, $N_2$ ; GC yields.

## Supplementary Table 3. The effects of solvent

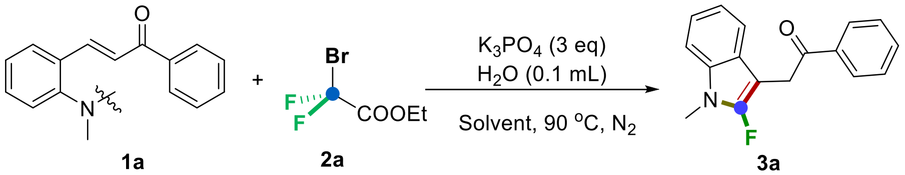


| Entry | solvent (2 mL) | Yield (%)[a] |
|---|---|---|
| 1 | $CH_3CN$ | 93(90)[b] |
| 2 | THF | 15 |
| 3 | 1.4-dioxane | Trace |
| 4 | DME | Trace |
| 5 | Toluene | NR |

[a] Reaction condition : **1a** (0.2 mmol), **2a** (3 equiv, 0.6 mmol), $K_3PO_4$ (3 equiv.), $H_2O$ (0.1 mL), solvent (2 mL) under 90 °C for 12 h, $N_2$ ; GC yields; [b] isolated yields.

## Supplementary Table 4. The effects of temperature

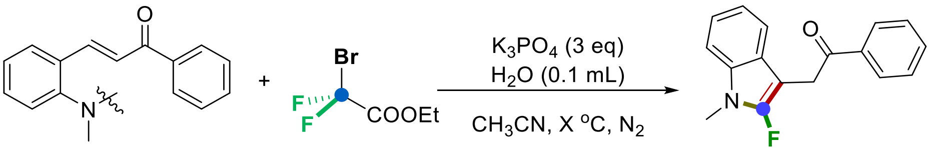


| Entry | T °C | Yield (%)[a] |
|---|---|---|
| 1 | 80 | 81 |
| 2 | 90 | 93(90)[b] |
| 3 | 100 | 93 |
| 4 | 110 | 88 |

Reaction condition : **1a** (0.2 mmol), **2a** (3 equiv, 0.6 mmol), $K_3PO_4$ (3 equiv.), $H_2O$ (0.1 mL), $CH_3CN$ (2 mL) under X °C for 12 h, $N_2$ ; GC yields; [b] isolated yield.

**Supplementary Table 5. The effects of halodifluorinated reagents**

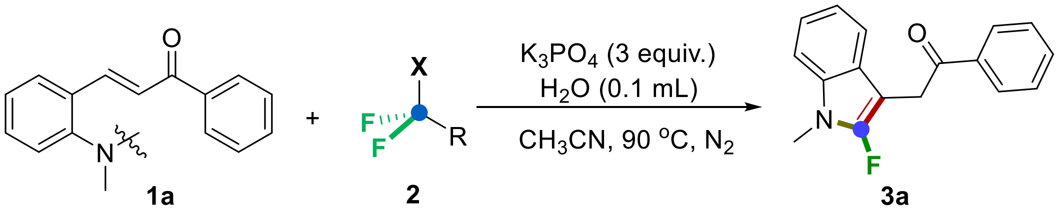


| entry | base | solvent | [:$CF_2$] (3 equiv.) | yield (%)[a] |
|---|---|---|---|---|
| 1 | $K_3PO_4$ | $CH_3CN$ | **2a** | 93(90)[b] |
| 2 | $K_3PO_4$ | $CH_3CN$ | **2b** | 88 |
| 3 | $K_3PO_4$ | $CH_3CN$ | **2c** | 18 |
| 4 | $K_3PO_4$ | $CH_3CN$ | **2d** | 66 |
| 5 | $K_3PO_4$ | $CH_3CN$ | **2e** | 49 |
| 6 | $K_2CO_3$ | $CH_3CN$ | **2f** | 57 |
| 7 | $K_2CO_3$ | $CH_3CN$ | **2g** | Trace |
| 8 | $K_2CO_3$ | $CH_3CN$ | **2h** | 49 |
| 9 | $K_3PO_4$ | $CH_3CN$ | **2a** (1.5 equiv.) | 70 |
| 10 | $K_3PO_4$ | $CH_3CN$ | **2a** (2 equiv.) | 77 |
| 11 | $K_3PO_4$ | $CH_3CN$ | **2a** (2.5 equiv.) | 82 |
| 12 | $K_3PO_4$ | $CH_3CN$ | **2a** (3.5 equiv.) | 94 |

Reaction condition: [a] **1a** (0.2 mmol), **2** (X equiv, X mmol), base (3 equiv.), $H_2O$ (0.1 mL), solvent (2 mL) for 12 h, $N_2$ ; GC yields; [b] isolated yields.

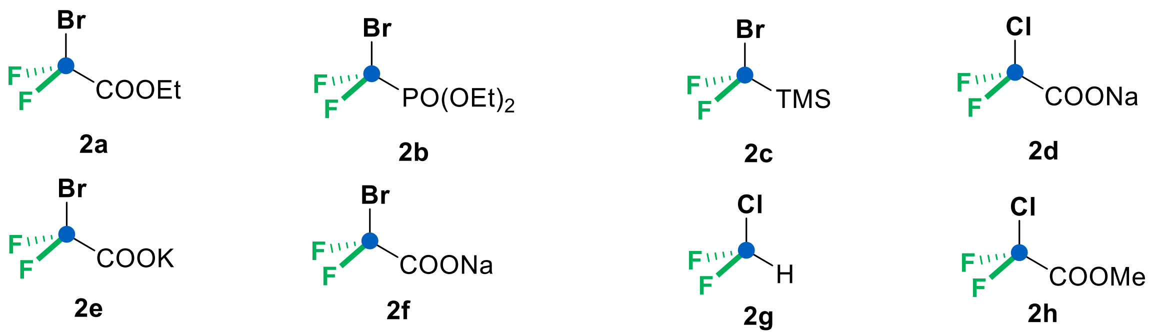

## 2.1.2 Supplementary Table 6. Optimization of the reaction conditions of the synthesis of 2-fluoroindole (5a)

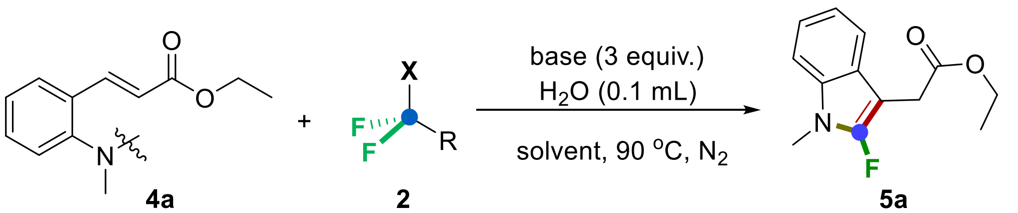


4a + 2 → 5a

| entry | base | solvent | [:$CF_2$] | T (°C) | yield (%)[a] |
|---|---|---|---|---|---|
| 1 | $K_2CO_3$ | $CH_3CN$ | **2a** | 90 | 25 |
| 2 | $K_3PO_4$ | $CH_3CN$ | **2a** | 90 | 27 |
| 3 | $Na_2CO_3$ | $CH_3CN$ | **2a** | 90 | trace |
| 4 | $Cs_2CO_3$ | $CH_3CN$ | **2a** | 90 | 51(45)[b] |
| 5 | NaOH | $CH_3CN$ | **2a** | 90 | trace |
| 6 | $^t$BuoK | $CH_3CN$ | **2a** | 90 | trace |
| 7 | $Li_2CO_3$ | $CH_3CN$ | **2a** | 90 | trace |
| 8 | AcOK | $CH_3CN$ | **2a** | 90 | N.D. |
| 9 | $CF_3COONa$ | $CH_3CN$ | **2a** | 90 | N.D. |
| 10 | $Na_3PO_4$ | $CH_3CN$ | **2a** | 90 | 28 |
| 11 | AcONa | $CH_3CN$ | **2a** | 90 | trace |
| 12 | $NaH_2PO_4$ | $CH_3CN$ | **2a** | 90 | trace |
| 13 | $Cs_2CO_3$ | $CH_3CN$ | **2a** | 100 | 50 |
| 14 | $Cs_2CO_3$ | $CH_3CN$ | **2a** | 120 | 53 |
| 15 | $Cs_2CO_3$ | 1,4-dioxane | **2a** | 90 | trace |
| 16 | $Cs_2CO_3$ | THF | **2a** | 90 | trace |
| 17 | $Cs_2CO_3$ | $CH_3OH$ | **2a** | 90 | 39 |
| 18 | $Cs_2CO_3$ | EtOH | **2a** | 90 | trace |
| 19 | $Cs_2CO_3$ | acetone | **2a** | 90 | trace |
| 20 | $Cs_2CO_3$ | toluene | **2a** | 90 | trace |
| 21 | $Cs_2CO_3$ | DMF | **2a** | 90 | trace |
| 22 | $Cs_2CO_3$ | DMSO | **2a** | 90 | trace |
| 23 | $K_2CO_3$ | $CH_3CN$ | **2b** | 90 | 65(59)[b] |
| 24 | $NH_4OAc$ | DCM | **2c** | rt | 30 |
| 25 | $K_2CO_3$ | $CH_3CN$ | **2d** | 90 | 18 |
| 26 | KOH | $CH_3CN$ | **2e** | 90 | NR |
| 27 | $K_2CO_3$ | $CH_3CN$ | **2f** | 90 | 19 |

Reaction condition: [a] **4a** (0.2 mmol), **2** (3 equiv, 0.6 mmol), base (3 equiv.), $H_2O$ (0.1 mL), solvent (2 mL) under 90 °C for 12 h, $N_2$ ; GC yields; [b] isolated yields.

### 2.1.3 Supplementary Table 7. Optimization of the reaction conditions of the synthesis of 2-fluoroindole (9b)

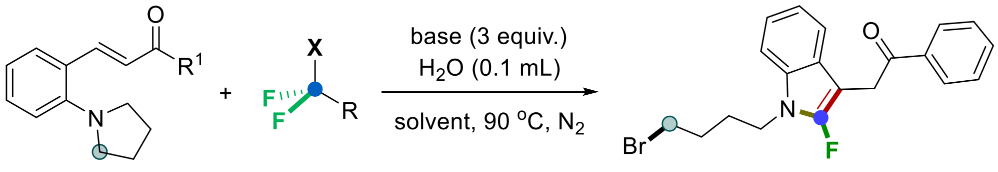


**8b** **2** **9b**

| entry | base + additive | solvent | [:CF$_2$] | T ($^o$C) | yield (%)[a] |
|---|---|---|---|---|---|
| 1 | $K_2CO_3$ | $CH_3CN$ | **2a** | 90 | 29 |
| 2 | $K_3PO_4$ | $CH_3CN$ | **2a** | 90 | 57 |
| 3 | $Na_2CO_3$ | $CH_3CN$ | **2a** | 90 | 23 |
| 4 | $Cs_2CO_3$ | $CH_3CN$ | **2a** | 90 | 43 |
| 5 | NaOH | $CH_3CN$ | **2a** | 90 | <10 |
| 6 | KF | $CH_3CN$ | **2a** | 90 | 19 |
| 7 | MeONa | $CH_3CN$ | **2a** | 90 | trace |
| 8 | AcOK | $CH_3CN$ | **2a** | 90 | trace |
| 9 | $Na_2HPO_4$ | $CH_3CN$ | **2a** | 90 | trace |
| 10 | $Na_3PO_4$ | $CH_3CN$ | **2a** | 90 | trace |
| 11 | AcONa | $CH_3CN$ | **2a** | 90 | trace |
| 12 | $NaH_2PO_4$ | $CH_3CN$ | **2a** | 90 | trace |
| 13 | NaOH | $CH_3CN$ | **2a** | 90 | <10 |
| 14 | NaF | $CH_3CN$ | **2a** | 90 | 19 |
| 15 | $NaNH_2$ | $CH_3CN$ | **2a** | 90 | trace |
| 16 | Triethylamine | $CH_3CN$ | **2a** | 90 | NR |
| 17 | AcONa | $CH_3CN$ | **2a** | 90 | trace |
| 18 | $^t$BuOK | $CH_3CN$ | **2a** | 90 | NR |
| 19 | $KHF_2$ | $CH_3CN$ | **2a** | 90 | NR |
| 20 | DBU | $CH_3CN$ | **2a** | 90 | NR |
| 21 | $K_3PO_4$+ KBr | $CH_3CN$ | **2a** | 90 | 59 |
| 22 | $K_3PO_4$+ NaBr | $CH_3CN$ | **2a** | 90 | 55 |
| 23 | $K_3PO_4$+ ZnBr | $CH_3CN$ | **2a** | 90 | 14 |
| 24 | $K_3PO_4$ | $CH_3CN$ | **2a** | 100 | 66 |
| 25 | $K_3PO_4$ | $CH_3CN$ | **2a** | 120 | 65 |
| 26 | $K_3PO_4$ | $CH_3CN$ | **2a** | 90 | 71[b](68)[bc] |
| 27 | $K_3PO_4$ | 1,4-dioxane | **2a** | 90 | trace |
| 28 | $K_3PO_4$ | THF | **2a** | 90 | trace |
| 29 | $K_3PO_4$ | chlorobenzene | **2a** | 90 | NR |
| 30 | $K_3PO_4$ | DMA | **2a** | 90 | NR |
| 31 | $K_3PO_4$ | acetone | **2a** | 90 | NR |
| 32 | $K_3PO_4$ | toluene | **2a** | 90 | NR |
| 33 | $K_3PO_4$ | DMF | **2a** | 90 | trace |
| 34 | $K_3PO_4$ | DMSO | **2a** | 90 | trace |
| 35 | $K_3PO_4$ | $CH_3CN$ | **2b** | 90 | 50 |
| 36 | $K_3PO_4$ | DCM | **2c** | rt | 38 |
| 37 | $K_2CO_3$ | $CH_3CN$ | **2d** | 90 | 11 |
| 38 | KOH | $CH_3CN$ | **2e** | 90 | trace |
| 39 | $K_2CO_3$ | $CH_3CN$ | **2f** | 90 | 10 |

Reaction condition: [a] **4a** (0.2 mmol), **2** (3 equiv, 0.6 mmol), base (3 equiv.), $H_2O$ (0.1 mL), solvent (2 mL) under 90 $^o$C for 12 h, $N_2$ ; GC yields; [b] 24 h; [c] isolated yields.

## 2.2 Control experiments

### 2.2.1 Experiments with $H_2O$ was replaced by $D_2O$

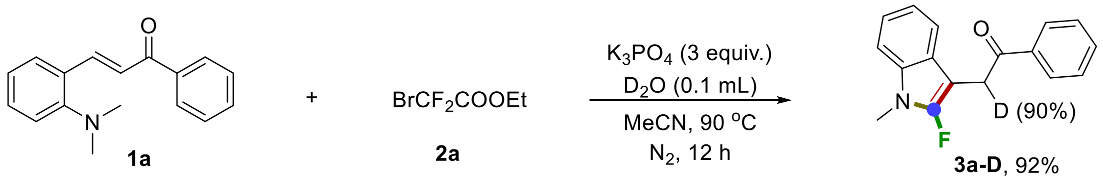


In air, chalcones **1a** (0.2 mmol) and $K_3PO_4$ (3 eq, 0.6 mmol) were added to a Schlenk tube equipped with a stir bar. The vessel was evacuated and filled with $N_2$ (three cycles). $BrCF_2COOEt$ (3 eq, 0.6 mmol), $D_2O$ (0.1 mL) and $CH_3CN$ (2 mL) added in turn by syringe under $N_2$ atmosphere. The resulting reaction mixture was stirred vigorously at 90 °C for 12 h. Upon completion of the reaction, the solvent was evaporated under reduced pressure and the residue was purified by flash column chromatography (silica gel, petroleum ether: $CH_2Cl_2$ =10:1, v/v) to give the desired products.

### 2.2.2 Experiments with diflurocarbene trapping reagent 18

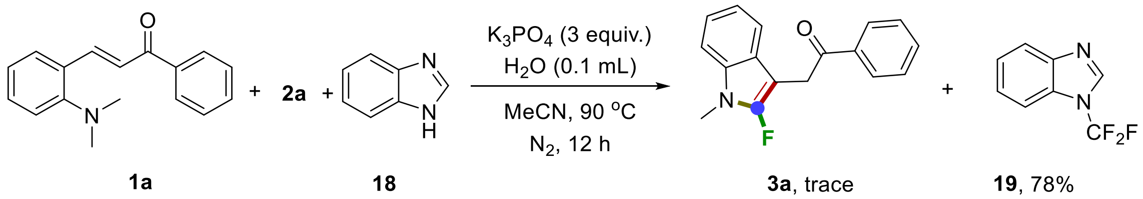


In air, chalcones **1a** (0.2 mmol), Benzimidazole **18** (3 eq, 0.6 mmol) and $K_3PO_4$ (3 eq, 0.6 mmol) were added to a Schlenk tube equipped with a stir bar. The vessel was evacuated and filled with $N_2$ (three cycles). $BrCF_2COOEt$ (3 eq, 0.6 mmol), $H_2O$ (0.1 mL) and $CH_3CN$ (2 mL) added in turn by syringe under $N_2$ atmosphere. The resulting reaction mixture was stirred vigorously at 90 °C for 12 h. Upon completion of the reaction, the solvent was evaporated under reduced pressure and the residue was purified by flash column chromatography (silica gel, petroleum ether: $CH_2Cl_2$ =10:1, v/v) to give the desired products.

### 2.2.3 experiments with HRMS analysis

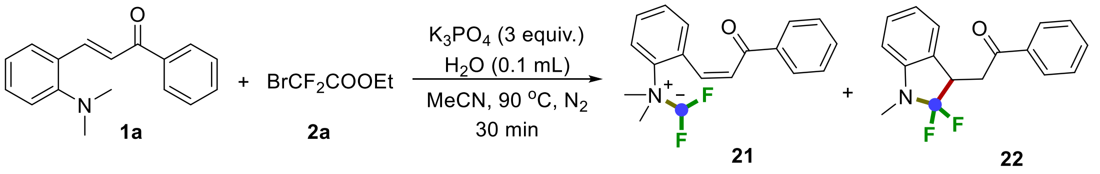


$C_{18}H_{19}F_2NO^+$: 303.1429 Found: 303.1431

$C_{17}H_{16}F_2NO^+$: 288.1194 288.1199

In air, chalcones **1a** (0.2 mmol), and $K_3PO_4$ (3 eq, 0.6 mmol) were added to a Schlenk tube equipped with a stir bar. The vessel was evacuated and filled with $N_2$ (three cycles). $BrCF_2COOEt$ (3 eq, 0.6 mmol), $H_2O$ (0.1 mL) and $CH_3CN$ (2 mL) added in turn by syringe under $N_2$ atmosphere. The resulting reaction mixture was stirred vigorously at 90 ºC for 30 min.

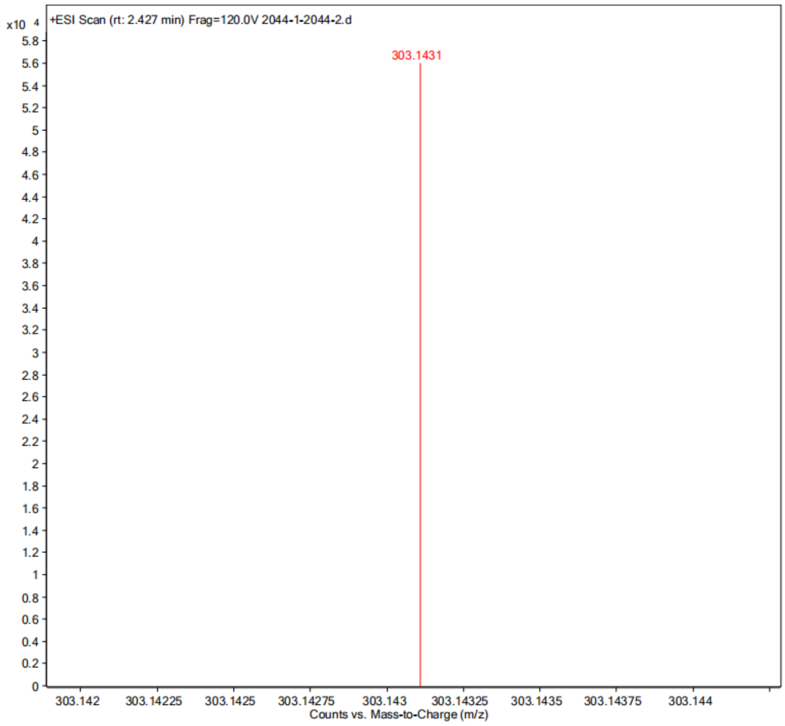

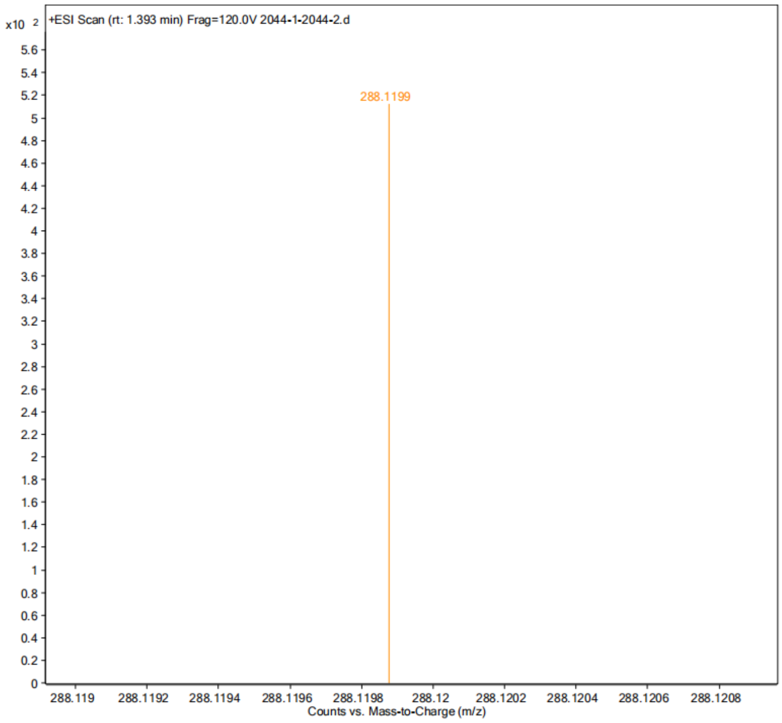
+ESI Scan (rt: 1.393 min) Frag=120.0V 2044-1-2044-2.d
x10 2
288.1199
Counts vs. Mass-to-Charge (m/z)

## 2.3 Crystal data

### 2.3.1 Crystal data of 3c

Crystallographic data for compound **3c** (CCDC-2049500) has been deposited with the Cambridge Crystallographic Data Centre, Copies of the data can be obtained, free of charge, on application to CCDC (Email: deposit@ccdc.cam.ac.uk).

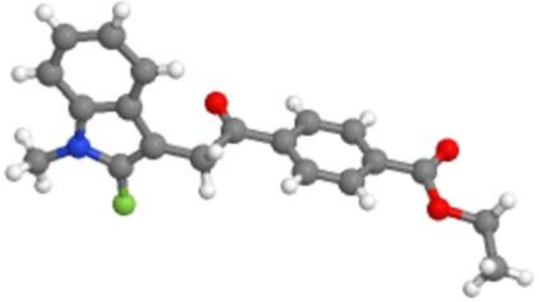

| | | | |
|---|---|---|---|
| Bond precision: | C-C = 0.0031 A | | Wavelength=0.71073 |
| Cell: | a=10.0500(7) | b=16.5285(10) | c=10.6310(7) |
| | alpha=90 | beta=102.560(7) | gamma=90 |
| Temperature: | 298 K | | |

| | Calculated | Reported |
|---|---|---|
| Volume | 1723.7(2) | 1723.7(2) |
| Space group | P 21/n | P 1 21/n 1 |
| Hall group | -P 2yn | -P 2ybc (x- |
| Moiety formula | C20 H18 F N O3 | C20 H18 F N O3 |
| Sum formula | C20 H18 F N O3 | C20 H18 F N O3 |
| Mr | 339.35 | 339.37 |
| Dx,g cm-3 | 1.308 | 1.308 |
| Z | 4 | 4 |
| Mu (mm-1) | 0.095 | 0.095 |
| F000 | 712.0 | 712.4 |
| F000’ | 712.38 | |
| h,k,lmax | 11,19,12 | 11,19,12 |
| Nref | 3025 | 3017 |
| Tmin,Tmax | | 0.714,1.000 |
| Tmin’ | | |

Correction method= # Reported T Limits: Tmin=0.714 Tmax=1.000
AbsCorr = MULTI-SCAN

Data completeness= 0.997 Theta(max)= 24.990

R(reflections)= 0.0512( 2204) wR2(reflections)= 0.1735( 3017)

S = 1.173 Npar= 227

### 2.3.2 Crystal data of 3j

Crystallographic data for compound **3j** (CCDC-2049496) has been deposited with the Cambridge Crystallographic Data Centre, Copies of the data can be obtained, free of charge, on application to CCDC (Email: deposit@ccdc.cam.ac.uk).

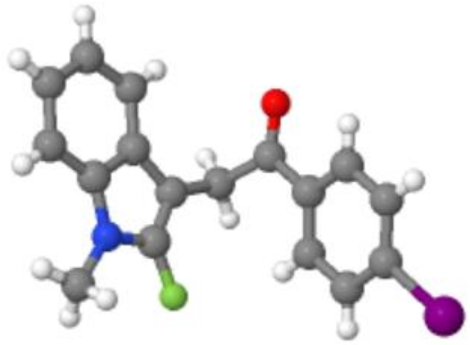

Bond precision: C-C = 0.0096 A Wavelength=1.54178

| | | | |
|---|---|---|---|
| Cell: | a=9.1707(6) | b=9.4718(6) | c=9.6305(6) |
| | alpha=92.666(2) | beta=104.612(2) | gamma=113.963(2) |
| Temperature: | 100 K | | |

| | Calculated | Reported |
|---|---|---|
| Volume | 729.13(8) | 729.13(8) |
| Space group | P -1 | P -1 |
| Hall group | -P 1 | -P 1 |
| Moiety formula | C17 H13 F I N O | C17 H13 F I N O |
| Sum formula | C17 H13 F I N O | C17 H13 F I N O |
| Mr | 393.18 | 393.18 |
| Dx,g cm-3 | 1.791 | 1.791 |
| Z | 2 | 2 |
| Mu (mm-1) | 17.339 | 17.339 |
| F000 | 384.0 | 384.0 |
| F000' | 384.49 | |
| h,k,lmax | 11,11,11 | 11,11,11 |
| Nref | 2678 | 2665 |
| Tmin,Tmax | 0.066,0.044 | 0.032,0.167 |
| Tmin' | 0.010 | |

Correction method= # Reported T Limits: Tmin=0.032 Tmax=0.167
AbsCorr = MULTI-SCAN

Data completeness= 0.995 Theta(max)= 68.416

R(reflections)= 0.0718( 2648) wR2(reflections)= 0.1742( 2665)

S = 1.186 Npar= 191

### 2.3.3 Crystal data of 3af

Crystallographic data for compound **3af** (CCDC-2066928) has been deposited with the Cambridge Crystallographic Data Centre, Copies of the data can be obtained, free of charge, on application to CCDC (Email: deposit@ccdc.cam.ac.uk).

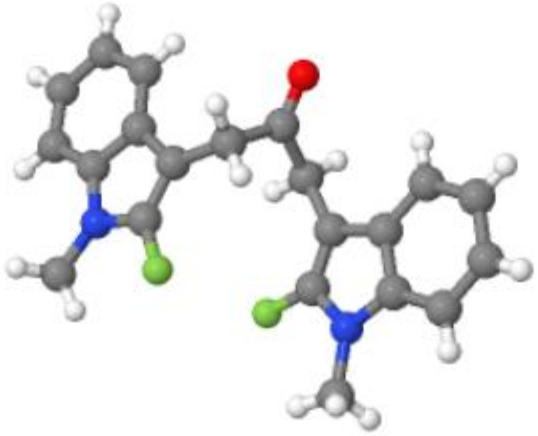

```
Bond precision:   C-C = 0.0028 A           Wavelength=0.71073

Cell:             a=7.3109(8)      b=14.1821(18)      c=16.6260(19)
                  alpha=90         beta=90            gamma=90
Temperature:      298 K

                  Calculated                 Reported
Volume            1723.9(3)                  1723.8(4)
Space group       P c c n                    P c c n
Hall group        -P 2ab 2ac                 -P 2ab 2ac
Moiety formula    C21 H18 F2 N2 O            C21 H18 F2 N2 O
Sum formula       C21 H18 F2 N2 O            C21 H18 F2 N2 O
Mr                352.37                     352.39
Dx,g cm-3         1.358                      1.358
Z                 4                          4
Mu (mm-1)         0.099                      0.099
F000              736.0                      736.4
F000'             736.38
h,k,lmax          8,16,19                    8,16,19
Nref              1523                       1519
Tmin,Tmax                                    0.810,1.000
Tmin'

Correction method= # Reported T Limits: Tmin=0.810 Tmax=1.000
AbsCorr = MULTI-SCAN

Data completeness= 0.997          Theta(max)= 24.990

R(reflections)= 0.0455( 1143)     wR2(reflections)= 0.1564( 1519)

S = 1.058                    Npar= 119
```

### 2.3.4 Crystal data of 9k

Crystallographic data for compound **9k** (CCDC-2054216) has been deposited with the Cambridge Crystallographic Data Centre, Copies of the data can be obtained, free of charge, on application to CCDC (Email: deposit@ccdc.cam.ac.uk).

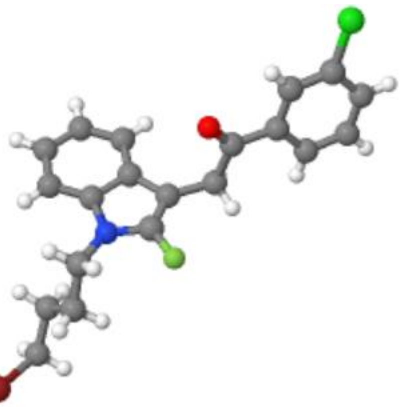

Bond precision: C-C = 0.0104 A    Wavelength=0.71073

| | | | |
|---|---|---|---|
| Cell: | a=8.9676(11) | b=10.2298(10) | c=20.982(2) |
| | alpha=90 | beta=100.505(11) | gamma=90 |
| Temperature: | 298 K | | |

| | Calculated | Reported |
|---|---|---|
| Volume | 1892.6(4) | 1892.6(4) |
| Space group | P 21/c | P 1 21/c 1 |
| Hall group | -P 2ybc | -P 2ybc |
| Moiety formula | C20 H18 Br Cl F N O | C20 H18 Br Cl F N O |
| Sum formula | C20 H18 Br Cl F N O | C20 H18 Br Cl F N O |
| Mr | 422.70 | 422.73 |
| Dx,g cm-3 | 1.484 | 1.484 |
| Z | 4 | 4 |
| Mu (mm-1) | 2.330 | 2.330 |
| F000 | 856.0 | 855.8 |
| F000' | 855.78 | |
| h,k,lmax | 10,12,24 | 10,12,24 |
| Nref | 3335 | 3325 |
| Tmin,Tmax | | 0.014,1.000 |
| Tmin' | | |

Correction method= # Reported T Limits: Tmin=0.014 Tmax=1.000
AbsCorr = MULTI-SCAN

Data completeness= 0.997    Theta(max)= 25.000

R(reflections)= 0.0832( 1548)    wR2(reflections)= 0.2443( 3325)

S = 1.051    Npar= 225

## 2.4 Characterization data for products

### (E)-3-(2-(dimethylamino)phenyl)-1-phenylprop-2-en-1-one (1a)

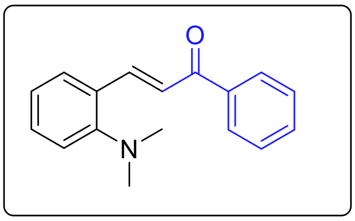

**$^1$H NMR** (500 MHz, Chloroform-*d*) δ 8.18 (d, *J* = 15.8 Hz, 1H), 8.03 (d, *J* = 1.6 Hz, 2H), 7.65 (s, 1H), 7.58 (s, 1H), 7.53 – 7.48 (m, 3H), 7.35 (s, 1H), 7.08 – 7.02 (m, 2H), 2.78 (s, 6H).

**$^{13}$C NMR** (126 MHz, Chloroform-*d*) δ 191.1, 154.3, 143.3, 138.5, 132.6, 131.1, 128.6, 128.5, 128.5, 128.4, 122.0, 121.6 118.4, 45.0.

**HRMS** (ESI, m/z) calcd for $C_{17}H_{18}NO^+$ $[M+H]^+$: 252.1383; found: 252.1381.

### (E)-1-([1,1'-biphenyl]-4-yl)-3-(2-(dimethylamino)phenyl)prop-2-en-1-one (1b)

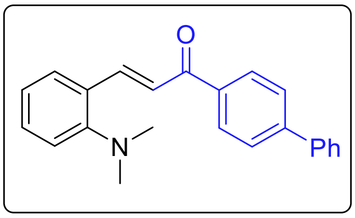

**$^1$H NMR** (500 MHz, Chloroform-*d*) δ 8.25 (d, *J* = 15.8 Hz, 1H), 8.15 (d, *J* = 8.4 Hz, 2H), 7.77 – 7.72 (m, 2H), 7.72 – 7.66 (m, 3H), 7.58 (d, *J* = 15.9 Hz, 1H), 7.49 (dd, *J* = 8.3, 7.0 Hz, 2H), 7.45 – 7.34 (m, 2H), 7.08 (ddd, *J* = 16.3, 7.8, 1.1 Hz, 2H), 2.81 (s, 6H).

**$^{13}$C NMR** (126 MHz, Chloroform-*d*) δ 190.5, 154.3, 145.3, 143.2, 140.0, 137.3, 131.1, 129.2, 129.0, 128.5, 128.4, 128.2, 127.3, 127.3, 122.3, 121.5, 118.5, 45.1.

**HRMS** (ESI, m/z) calcd for $C_{23}H_{22}NO^+$ $[M+H]^+$: 328.1696; found: 328.1691.

### (E)-4-(3-(2-(dimethylamino)phenyl)acryloyl)benzoate (1c)

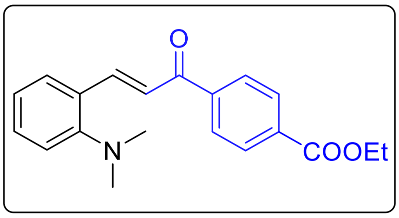

**$^1$H NMR** (500 MHz, Chloroform-*d*) δ 8.19 – 8.11 (m, 3H), 8.03 (dd, *J* = 8.3, 1.9 Hz, 2H), 7.63 (d, *J* = 7.7 Hz, 1H), 7.45 (d, *J* = 15.4 Hz, 1H), 7.32 (d, *J* = 3.3 Hz, 1H), 7.07 – 6.97 (m, 2H), 4.39 (ddd, *J* = 7.1, 3.3, 1.5 Hz, 2H), 2.75 (s, 6H), 1.39 (ddt, *J* = 7.2, 3.3, 1.8 Hz, 3H).

**$^{13}$C NMR** (126 MHz, Chloroform-*d*) δ 190.7, 165.9, 154.4, 144.2, 141.9, 133.7, 131.3, 129.7, 128.4, 128.3, 128.1, 122.1, 121.3, 118.5, 61.4, 45.0, 14.3.

**HRMS** (ESI, m/z) calcd for $C_{17}H_{18}NO^+$ $[M+H]^+$: 324.1594; found: 324.1598.

## (E)-4-(3-(2-(dimethylamino)phenyl)acryloyl)benzonitrile (1d)

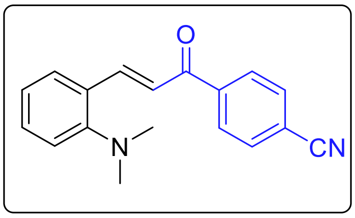


**$^{1}$H NMR** (500 MHz, Chloroform-*d*) δ 8.18 (d, *J* = 15.8 Hz, 1H), 8.08 (dd, *J* = 8.3, 1.6 Hz, 2H), 7.79 (d, *J* = 8.4 Hz, 2H), 7.63 (dd, *J* = 7.8, 1.7 Hz, 1H), 7.42 (d, *J* = 15.8 Hz, 1H), 7.37 (ddd, *J* = 8.7, 7.4, 1.6 Hz, 1H), 7.09 – 7.01 (m, 2H), 2.78 (d, *J* = 1.4 Hz, 6H).

**$^{13}$C NMR** (126 MHz, Chloroform-*d*) δ 189.8, 154.6, 145.0, 141.9, 132.5, 131.6, 128.9, 128.5, 127.8, 122.1, 120.6, 118.6, 118.1, 115.7, 45.1.

**HRMS** (ESI, m/z) calcd for $C_{18}H_{17}N_2O^+$ $[M+H]^+$: 277.1335; found: 277.1335.

## (E)-3-(2-(dimethylamino)phenyl)-1-(4-(methylthio)phenyl)prop-2-en-1-one (1e)

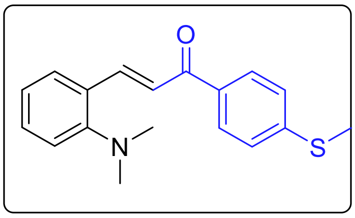


**$^{1}$H NMR** (500 MHz, Chloroform-*d*) δ 8.16 (d, *J* = 15.8 Hz, 1H), 7.97 (d, *J* = 8.5 Hz, 2H), 7.64 (dd, *J* = 7.8, 1.6 Hz, 1H), 7.50 (s, 1H), 7.38 – 7.29 (m, 3H), 7.09 – 7.00 (m, 2H), 2.78 (s, 6H), 2.54 (s, 3H).

**$^{13}$C NMR** (126 MHz, Chloroform-*d*) δ 189.8, 154.2, 145.3, 143.0, 134.8, 130.9, 129.0, 128.5, 128.4, 125.1, 122.0, 121.2, 118.4, 45.0, 14.9.

**HRMS** (ESI, m/z) calcd for $C_{18}H_{20}NOS^+$ $[M+H]^+$: 298.1260; found: 298.1258.

## (E)-1-(4-(diethylamino)phenyl)-3-(2-(dimethylamino)phenyl)prop-2-en-1-one (1f)

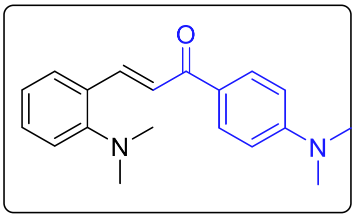


**$^{1}$H NMR** (500 MHz, Chloroform-*d*) δ 8.12 (d, *J* = 15.7 Hz, 1H), 8.01 (d, *J* = 9.1 Hz, 2H), 7.64 (dd, *J* = 7.7, 1.7 Hz, 1H), 7.55 (d, *J* = 15.7 Hz, 1H), 7.32 (ddd, *J* = 8.5, 7.3, 1.6 Hz, 1H), 7.09 – 6.97 (m, 2H), 6.68 (d, *J* = 9.0 Hz, 2H), 3.44 (q, *J* = 7.1 Hz, 4H), 2.78 (s, 6H), 1.22 (t, *J* = 7.1 Hz, 6H).

**$^{13}$C NMR** (126 MHz, Chloroform-*d*) δ 188.1, 153.9, 151.1, 140.7, 131.1, 130.3, 129.2, 128.3, 125.6, 121.9, 121.8, 118.3, 110.4, 44.9, 44.6, 12.6.

**HRMS** (ESI, m/z) calcd for $C_{21}H_{27}N_2O^+$ $[M+H]^+$: 323.2118; found: 323.2116.

### (E)-3-(2-(dimethylamino)phenyl)-1-(4-methoxyphenyl)prop-2-en-1-one (1g)

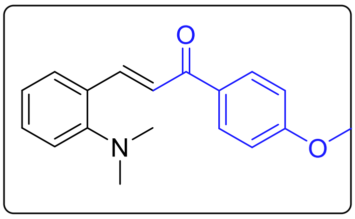


**$^1$H NMR** (500 MHz, Chloroform-*d*) δ 8.16 (d, *J* = 15.7 Hz, 1H), 8.10 – 8.02 (m, 2H), 7.64 (dd, *J* = 7.7, 1.6 Hz, 1H), 7.51 (d, *J* = 15.7 Hz, 1H), 7.34 (td, *J* = 8.2, 7.7, 1.6 Hz, 1H), 7.09 – 6.95 (m, 4H), 3.88 (s, 3H), 2.78 (s, 6H).

**$^{13}$C NMR** (126 MHz, Chloroform-*d*) δ 189.3, 163.3, 154.2, 142.4, 131.4, 130.8, 128.7, 128.3, 122.0, 121.4, 118.4, 113.8, 55.5, 45.0.

**HRMS** (ESI, m/z) calcd for $C_{18}H_{20}NO_2^+$ $[M+H]^+$: 282.1489; found: 282.1484.

### (E)-3-(2-(dimethylamino)phenyl)-1-(3,4-dimethylphenyl)prop-2-en-1-one (1h)

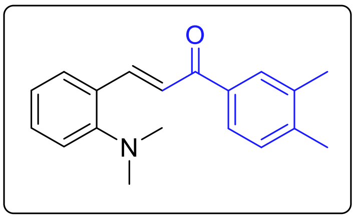


**$^1$H NMR** (500 MHz, Chloroform-*d*) δ 8.18 (dd, *J* = 15.9, 1.9 Hz, 1H), 7.84 (s, 1H), 7.79 (d, *J* = 2.0 Hz, 1H), 7.66 (dd, *J* = 7.7, 1.6 Hz, 1H), 7.51 (d, *J* = 15.8 Hz, 1H), 7.35 (ddd, *J* = 8.6, 7.3, 1.6 Hz, 1H), 7.26 (d, *J* = 7.8 Hz, 1H), 7.08 – 7.02 (m, 2H), 2.79 (s, 6H), 2.35 (d, *J* = 5.8 Hz, 6H).

**$^{13}$C NMR** (126 MHz, Chloroform-*d*) δ 190.8, 154.2, 142.7, 142.1, 136.9, 136.4, 130.9, 129.8, 129.8, 128.8, 128.3, 126.29, 122.0, 121.8, 118.4, 450, 20.0, 19.9.

**HRMS** (ESI, m/z) calcd for $C_{19}H_{22}NO^+$ $[M+H]^+$: 280.1696; found: 280.1695.

### (E)-3-(2-(dimethylamino)phenyl)-1-(o-tolyl)prop-2-en-1-one (1i)

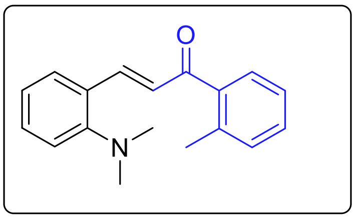


**$^1$H NMR** (500 MHz, Chloroform-*d*) δ 7.83 (d, *J* = 16.1 Hz, 1H), 7.62 – 7.57 (m, 1H), 7.50 (d, *J* = 7.5 Hz, 1H), 7.41 – 7.33 (m, 2H), 7.28 (d, *J* = 7.6 Hz, 2H), 7.11 (dd, *J* = 16.2, 1.2 Hz, 1H), 7.03 (d, *J* = 8.1 Hz, 2H), 2.72 (s, 6H), 2.47 (s, 3H).

**$^{13}$C NMR** (126 MHz, Chloroform-*d*) δ 197.5, 154.1, 144.6, 139.5, 136.7, 131.3, 130.3, 128.4, 128.0,

126.3, 125.4, 122.1, 118.4, 45.0, 20.2.

**HRMS** (ESI, m/z) calcd for $C_{18}H_{20}NO^+$ $[M+H]^+$: 266.1539; found: 266.1535.

### (E)-3-(2-(dimethylamino)phenyl)-1-(4-iodophenyl)prop-2-en-1-one (1j)

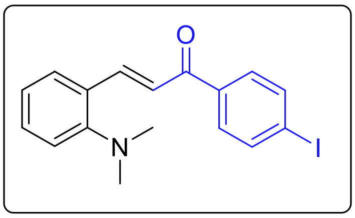


**$^1$H NMR** (500 MHz, Chloroform-*d*) δ 8.17 (d, *J* = 15.8 Hz, 1H), 7.85 (d, *J* = 8.4 Hz, 2H), 7.76 – 7.71 (m, 2H), 7.63 (dd, *J* = 7.8, 1.6 Hz, 1H), 7.42 (d, *J* = 15.8 Hz, 1H), 7.35 (ddd, *J* = 8.3, 7.3, 1.6 Hz, 1H), 7.08 – 7.01 (m, 2H), 2.77 (s, 6H).

**$^{13}$C NMR** (126 MHz, Chloroform-*d*) δ 190.3, 154.4, 143.9, 137.9, 137.8, 131.3, 130.0, 128.4, 128.2, 122.1, 120.9, 118.5, 100.3, 45.1.

**HRMS** (ESI, m/z) calcd for $C_{17}H_{17}INO^+$ $[M+H]^+$: 378.0349; found: 378.0349.

### (E)-3-(2-(dimethylamino)phenyl)-1-(naphthalen-2-yl)prop-2-en-1-one (1k)

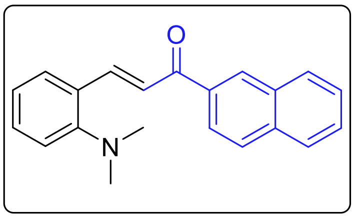


**$^1$H NMR** (500 MHz, Chloroform-*d*) δ 8.57 (d, *J* = 1.7 Hz, 1H), 8.27 (d, *J* = 15.8 Hz, 1H), 8.14 (dd, *J* = 8.6, 1.8 Hz, 1H), 8.00 (dd, *J* = 8.0, 1.4 Hz, 1H), 7.95 (d, *J* = 8.6 Hz, 1H), 7.90 (dd, *J* = 8.1, 1.3 Hz, 1H), 7.73 (dd, *J* = 7.6, 1.6 Hz, 1H), 7.66 (d, *J* = 15.8 Hz, 1H), 7.59 (dddd, *J* = 19.8, 8.1, 6.9, 1.4 Hz, 2H), 7.38 (ddd, *J* = 8.5, 7.2, 1.7 Hz, 1H), 7.08 (t, *J* = 7.8 Hz, 2H), 2.81 (s, 6H).

**$^{13}$C NMR** (126 MHz, Chloroform-*d*) δ 191.0, 154.3, 143.3, 135.9, 135.5, 132.7, 131.1, 129.9, 129.6, 128.6, 128.4, 128.3, 127.9, 126.8, 124.7, 122.1, 121.6, 121.6, 118.5, 45.1.

**HRMS** (ESI, m/z) calcd for $C_{21}H_{20}NO^+$ $[M+H]^+$: 302.1539; found: 302.1545.

### (E)-3-(2-(dimethylamino)phenyl)-1-(furan-2-yl)prop-2-en-1-one (1l)

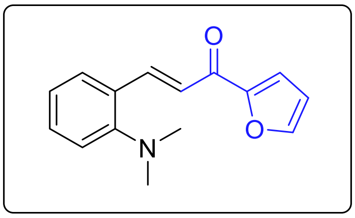


**$^1$H NMR** (500 MHz, Chloroform-*d*) δ 8.24 (d, *J* = 15.9 Hz, 1H), 7.65 – 7.59 (m, 2H), 7.40 (d, *J* = 15.9 Hz, 1H), 7.35 – 7.29 (m, 2H), 7.06 – 6.98 (m, 2H), 6.57 (dd, *J* = 3.6, 1.7 Hz, 1H), 2.76 (s, 6H).

**$^{13}$C NMR** (126 MHz, Chloroform-*d*) δ 178.6, 154.4, 153.9, 146.4, 146.3, 142.3, 131.1, 128.4, 128.3, 122.0, 120.6, 120.6, 118.4, 117.2, 117.2, 112. 5, 45.0.

**HRMS** (ESI, m/z) calcd for $C_{15}H_{16}NO_2^+$ [M+H]$^+$: 212.1176; found: 212.1174.

### (E)-3-(2-(dimethylamino)phenyl)-1-(thiophen-3-yl)prop-2-en-1-one (1m)

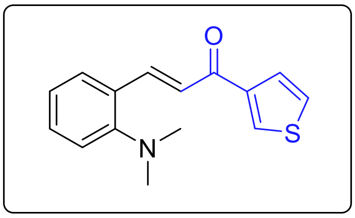


**$^1$H NMR** (500 MHz, Chloroform-*d*) δ 8.21 – 8.13 (m, 2H), 7.68 (dd, *J* = 5.1, 1.2 Hz, 1H), 7.63 (dd, *J* = 7.8, 1.6 Hz, 1H), 7.40 – 7.32 (m, 3H), 7.08 – 7.01 (m, 2H), 2.78 (s, 6H).

**$^{13}$C NMR** (126 MHz, Chloroform-*d*) δ 184.5, 154.2, 143.4, 142.5, 131.8, 131.0, 128.4, 128.4, 127.6, 126.4, 122.20 122.0, 118.4, 45.0.

**HRMS** (ESI, m/z) calcd for $C_{15}H_{16}NOS^+$ [M+H]$^+$: 258.0947; found: 258.0941.

### (E)-1,1'-(1,3-phenylene)bis(3-(2-(dimethylamino)phenyl)prop-2-en-1-one) (1n)

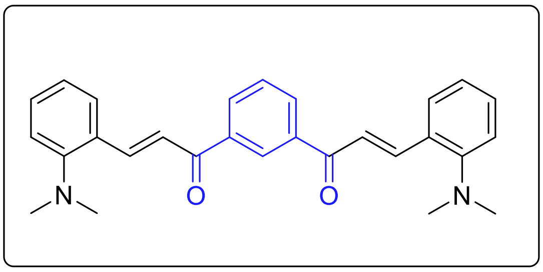


**$^1$H NMR** (500 MHz, Chloroform-*d*) δ 8.70 (d, *J* = 2.0 Hz, 1H), 8.29 – 8.22 (m, 4H), 7.70 – 7.64 (m, 3H), 7.58 (d, *J* = 15.8 Hz, 2H), 7.40 – 7.35 (m, 2H), 7.10 – 7.03 (m, 4H), 2.80 (s, 12H).

**$^{13}$C NMR** (126 MHz, Chloroform-*d*) δ 190.4, 154.4, 144.0, 144.0, 138.9, 132.3, 131.3, 129.0, 128.5, 128.4, 128.2, 122.1, 121.1, 118.5, 45.1.

**HRMS** (ESI, m/z) calcd for $C_{28}H_{29}N_2O_2^+$ [M+H]$^+$: 425.2224; found: 425.2227.

### (E)-2-(2-(dimethylamino)benzylidene)-2,3-dihydro-1H-inden-1-one (1o)

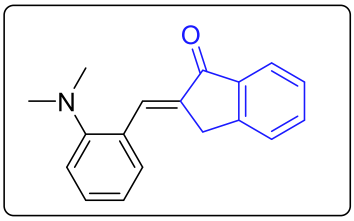


**$^1$H NMR** (500 MHz, Chloroform-*d*) δ 8.01 (d, *J* = 2.3 Hz, 1H), 7.92 (d, *J* = 7.7 Hz, 1H), 7.61 (ddd, *J* = 19.6, 7.5, 1.6 Hz, 2H), 7.55 – 7.50 (m, 1H), 7.42 (td, *J* = 7.6, 6.9, 2.0 Hz, 1H), 7.34 (ddd, *J* =

8.4, 7.2, 1.6 Hz, 1H), 7.09 – 7.01 (m, 2H), 4.00 (s, 2H), 2.77 (s, 6H).

**$^{13}$C NMR** (126 MHz, Chloroform-*d*) δ 194.5, 154.8, 149.8, 138.5, 134.4, 133.6, 132.4, 130.3, 129.8, 128.3, 127.5, 126.2, 124.3, 121.5, 118.2, 44.9, 32.1.

**HRMS** (ESI, m/z) calcd for $C_{18}H_{18}NO^+$ $[M+H]^+$: 264.1383; found: 264.1381.

## (E)-2-(2-(dimethylamino)benzylidene)-3,4-dihydronaphthalen-1(2H)-one (1p)

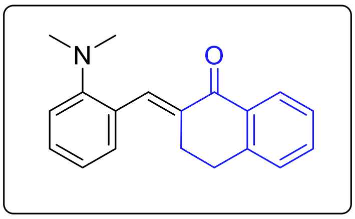


**$^1$H NMR** (500 MHz, Chloroform-*d*) δ 8.19 (d, *J* = 7.8 Hz, 1H), 8.00 (s, 1H), 7.49 (td, *J* = 7.4, 1.3 Hz, 1H), 7.41 – 7.35 (m, 1H), 7.31 (ddd, *J* = 16.2, 8.0, 1.7 Hz, 2H), 7.26 (d, *J* = 7.6 Hz, 1H), 7.05 (dd, *J* = 8.1, 1.2 Hz, 1H), 7.00 (td, *J* = 7.4, 1.2 Hz, 1H), 3.11 (td, *J* = 6.4, 1.7 Hz, 2H), 2.96 (t, *J* = 6.5 Hz, 2H), 2.78 (s, 6H).

**$^{13}$C NMR** (126 MHz, Chloroform-*d*) δ 188.2, 153.8, 143.4, 136.4, 133.8, 133.7, 133.2, 130.4, 129.5, 128.6, 128.3, 128.2, 127.0, 120.8, 120.8, 117.6, 44.4, 29.2, 27.7.

**HRMS** (ESI, m/z) calcd for $C_{19}H_{20}NO^+$ $[M+H]^+$: 278. 1539; found: 278.1546.

## (E)-3-(2-(dimethylamino)benzylidene)chroman-4-one (1q)

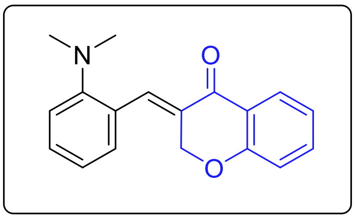


**$^1$H NMR** (500 MHz, Chloroform-*d*) δ 8.06 (dd, *J* = 7.8, 1.8 Hz, 1H), 8.00 (d, *J* = 1.9 Hz, 1H), 7.46 (ddd, *J* = 8.6, 7.1, 1.8 Hz, 1H), 7.34 (ddd, *J* = 8.6, 7.1, 1.9 Hz, 1H), 7.10 – 7.01 (m, 3H), 6.98 (ddd, *J* = 15.8, 7.8, 1.1 Hz, 2H), 5.27 (d, *J* = 1.8 Hz, 2H), 2.75 (s, 6H).

**$^{13}$C NMR** (126 MHz, Chloroform-*d*) δ 182.7, 161.3, 154.0, 137.1, 135.6, 130.6, 130.5, 129.0, 127.9, 127.0, 122.4, 121.8, 120.9, 117.9, 117.9, 68.2, 44.0.

**HRMS** (ESI, m/z) calcd for $C_{18}H_{18}NO_2^+$ $[M+H]^+$: 280.1332; found: 280.1328.

## (E)-6-(2-(dimethylamino)benzylidene)-6,7,8,9-tetrahydro-5H-benzo[7]annulen-5-one (1r)

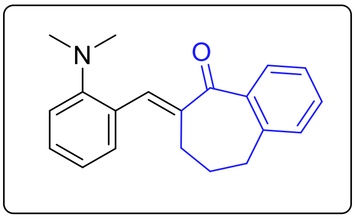


$^{1}$**H NMR** (500 MHz, Chloroform-*d*) δ 8.12 (s, 1H), 7.84 (dd, *J* = 7.6, 1.5 Hz, 1H), 7.48 (td, *J* = 7.4, 1.5 Hz, 1H), 7.43 – 7.33 (m, 3H), 7.23 (dd, *J* = 7.5, 1.2 Hz, 1H), 7.10 – 7.02 (m, 2H), 2.93 (t, *J* = 6.9 Hz, 2H), 2.83 (s, 6H), 2.64 (t, *J* = 6.9 Hz, 2H), 2.09 (p, *J* = 6.9 Hz, 2H).

$^{13}$**C NMR** (126 MHz, Chloroform-*d*) δ 198.0, 153.9, 139.7, 139.3, 138.1, 135.7, 132.3, 129.9, 129.5, 129.9, 128.9, 128.7, 127.0, 121.0, 117.6, 44.5, 31.7, 27.1, 24.8.

**HRMS** (ESI, m/z) calcd for $C_{20}H_{22}NO^+$ $[M+H]^+$: 292.1696; found: 292.1701.

## (E)-4-(2-(dimethylamino)phenyl)but-3-en-2-one (1s)

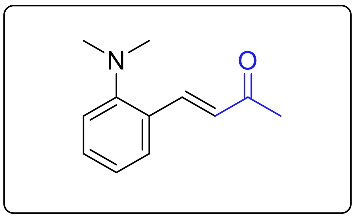


$^{1}$**H NMR** (500 MHz, Chloroform-*d*) δ 7.90 (d, *J* = 16.4 Hz, 1H), 7.50 (dd, *J* = 7.7, 1.6 Hz, 1H), 7.32 (td, *J* = 7.6, 7.2, 1.4 Hz, 1H), 7.07 – 7.03 (m, 1H), 7.00 (t, *J* = 7.5 Hz, 1H), 6.64 (d, *J* = 16.5 Hz, 1H), 2.75 (d, *J* = 1.0 Hz, 6H), 2.39 (s, 3H).

$^{13}$**C NMR** (126 MHz, Chloroform-*d*) δ 199.1, 153.8, 142.0,131.0, 128.1, 128.1, 126.9, 122.3, 118.4, 45.0, 27.0.

**HRMS** (ESI, m/z) calcd for $C_{12}H_{16}NO^+$ $[M+H]^+$: 190.1226; found: 190.1222.

## (E)-1-(2-(dimethylamino)phenyl)-4-methylpent-1-en-3-one (1t)

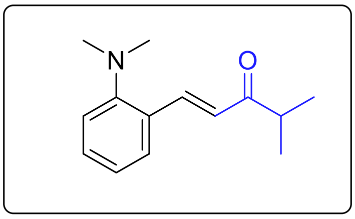


$^{1}$**H NMR** (500 MHz, Chloroform-*d*) δ 7.97 (d, *J* = 16.3 Hz, 1H), 7.52 (dd, *J* = 7.7, 1.6 Hz, 1H), 7.35 – 7.27 (m, 1H), 7.04 – 6.94 (m, 2H), 6.73 (d, *J* = 16.3 Hz, 1H), 3.04 (p, *J* = 6.9 Hz, 1H), 2.74 (s, 6H), 1.18 (d, *J* = 7.0 Hz, 6H).

$^{13}$**C NMR** (126 MHz, Chloroform-*d*) δ 204.5, 153.9, 140.7, 130.8, 128.3, 128.1, 124.4, 122.2, 118.4, 45.0, 38.3, 18.8.

**HRMS** (ESI, m/z) calcd for $C_{14}H_{20}NO^+$ $[M+H]^+$: 218.1539; found: 218.1538.

### (E)-1-(2-(dimethylamino)phenyl)-4,4-dimethylpent-1-en-3-one (1u)

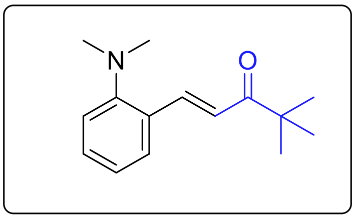

**$^{1}$H NMR** (500 MHz, Chloroform-*d*) δ 8.05 (d, *J* = 15.7 Hz, 1H), 7.54 (dd, *J* = 7.7, 1.6 Hz, 1H), 7.33 – 7.27 (m, 1H), 7.12 (d, *J* = 15.8 Hz, 1H), 7.03 (dd, *J* = 8.2, 1.2 Hz, 1H), 6.99 (td, *J* = 7.6, 1.4 Hz, 1H), 2.74 (d, *J* = 1.1 Hz, 6H), 1.24 (s, 9H).

**$^{13}$C NMR** (126 MHz, Chloroform-*d*) δ 204.6, 154.1, 141.1, 130.6, 128.5, 128.2, 121.9, 120.2, 118.4, 44.9, 43.2, 26.5.

**HRMS** (ESI, m/z) calcd for $C_{15}H_{22}NO^{+}$ $[M+H]^{+}$: 232.1696; found: 232.1699.

### (E)-1-(2-(dimethylamino)phenyl)-2-methylpent-1-en-3-one (1v)

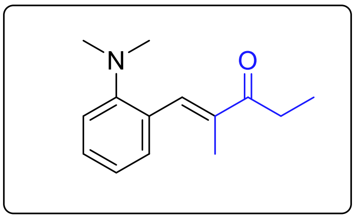

**$^{1}$H NMR** (500 MHz, Chloroform-*d*) δ 7.67 (s, 1H), 7.35 – 7.22 (m, 2H), 7.07 – 6.96 (m, 2H), 2.88 (q, *J* = 7.3 Hz, 2H), 2.74 (s, 6H), 2.05 (d, *J* = 1.4 Hz, 3H), 1.19 (t, *J* = 7.3 Hz, 3H).

**$^{13}$C NMR** (126 MHz, Chloroform-*d*) δ 203.4, 153.0, 138.1, 135.4, 130.5, 129.3, 129.0, 121.2, 117.5, 44.5, 30.8, 13.3, 9.0.

**HRMS** (ESI, m/z) calcd for $C_{14}H_{20}NO^{+}$ $[M+H]^{+}$: 218.1539; found: 218.1535.

### (E)-1-(2-(dimethylamino)phenyl)-2,4-dimethylpent-1-en-3-one (1w)

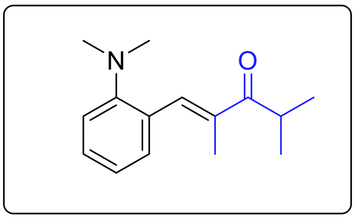

**$^{1}$H NMR** (500 MHz, Chloroform-*d*) δ 7.67 (s, 1H), 7.34 – 7.26 (m, 2H), 7.06 – 6.97 (m, 2H), 3.55 (p, *J* = 6.8 Hz, 1H), 2.73 (s, 6H), 2.05 (d, *J* = 1.5 Hz, 3H), 1.19 (d, *J* = 6.8 Hz, 6H).

**$^{13}$C NMR** (126 MHz, Chloroform-*d*) δ 206.9, 153.0, 137.9, 137.9, 134.6, 130.6, 129.3, 129.2, 121.3, 117.5, 44.5, 34.0, 19.8, 13.6.

**HRMS** (ESI, m/z) calcd for $C_{15}H_{22}NO^{+}$ $[M+H]^{+}$: 232.1696; found: 232.1696.

### (E)-1-(2-(dimethylamino)phenyl)dec-1-en-3-one (1x)

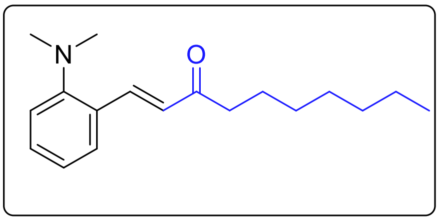

**$^{1}$H NMR** (500 MHz, Chloroform-*d*) δ 7.92 (d, *J* = 16.4 Hz, 1H), 7.52 (dd, *J* = 7.7, 1.6 Hz, 1H), 7.32 (ddd, *J* = 8.5, 7.3, 1.7 Hz, 1H), 7.04 (dd, *J* = 8.3, 1.1 Hz, 1H), 7.00 (td, *J* = 7.4, 1.2 Hz, 1H), 6.67 (d, *J* = 16.4 Hz, 1H), 2.75 (s, 6H), 2.69 (t, *J* = 7.5 Hz, 2H), 1.68 (q, *J* = 7.3 Hz, 2H), 1.34 (dd, *J* = 4.8, 2.8 Hz, 4H), 1.32 – 1.27 (m, 4H), 0.88 (t, *J* = 6.8 Hz, 3H).

**$^{13}$C NMR** (126 MHz, Chloroform-*d*) δ 201.4, 153.8, 140.8, 130.8, 128.3, 128.0, 126.2, 122.3, 118.4, 45.0, 40.2, 31.7, 29.4, 29.2, 24.7, 22.6, 14.1.

**HRMS** (ESI, m/z) calcd for $C_{18}H_{28}NO^+$ $[M+H]^+$: 274.2165; found: 274.2161.

## (E)-1-cyclopropyl-3-(2-(dimethylamino)phenyl)prop-2-en-1-one (1y)

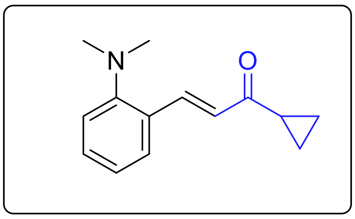

**$^{1}$H NMR** (500 MHz, Chloroform-*d*) δ 8.01 (d, *J* = 16.3 Hz, 1H), 7.53 (dd, *J* = 7.6, 1.6 Hz, 1H), 7.31 (ddd, *J* = 8.6, 7.3, 1.6 Hz, 1H), 7.04 (dd, *J* = 8.2, 1.2 Hz, 1H), 7.00 (td, *J* = 7.5, 1.2 Hz, 1H), 6.80 (d, *J* = 16.3 Hz, 1H), 2.75 (s, 6H), 2.32 (td, *J* = 7.8, 3.9 Hz, 1H), 1.14 (dt, *J* = 4.6, 3.3 Hz, 2H), 0.95 (dq, *J* = 7.2, 3.6 Hz, 2H).

**$^{13}$C NMR** (126 MHz, Chloroform-*d*) δ 200.5, 153.9, 140.5, 130.8, 128.3, 128.1, 126.3, 122.2, 118.4, 45.0, 18.9, 11.1.

**HRMS** (ESI, m/z) calcd for $C_{14}H_{18}NO^+$ $[M+H]^+$: 216.1383; found: 216.1378.

## (E)-1-cyclohexyl-3-(2-(dimethylamino)phenyl)prop-2-en-1-one (1z)

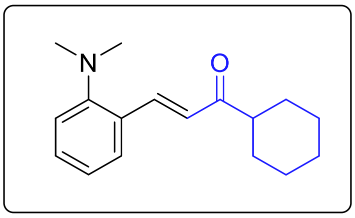

**$^{1}$H NMR** (500 MHz, Chloroform-*d*) δ 7.94 (d, *J* = 16.5 Hz, 1H), 7.53 – 7.47 (m, 1H), 7.27 (dd, *J* = 8.1, 3.0 Hz, 1H), 7.02 – 6.93 (m, 2H), 6.71 (dd, *J* = 16.3, 2.6 Hz, 1H), 2.71 (d, *J* = 4.2 Hz, 7H), 1.87 (t, *J* = 6.4 Hz, 2H), 1.79 (dd, *J* = 8.5, 4.3 Hz, 2H), 1.73 – 1.65 (m, 1H), 1.47 – 1.38 (m, 2H), 1.37 – 1.27 (m, 2H), 1.22 (td, *J* = 8.6, 4.5 Hz, 1H).

**$^{13}$C NMR** (126 MHz, Chloroform-*d*) δ 203.6, 153.9, 140.5, 140.5, 130.7, 128.3, 128.1, 124.5, 124.4,

122.1, 118.3, 48.7, 44.9, 29.0, 26.0, 25.8.

**HRMS** (ESI, m/z) calcd for $C_{17}H_{24}NO^+$ $[M+H]^+$: 258.1852; found: 258.1847.

## (E)-1-((1S,4S)-bicyclo[2.2.1]hept-5-en-2-yl)-3-(2-(dimethylamino)phenyl)prop-2-en-1-one (1aa)

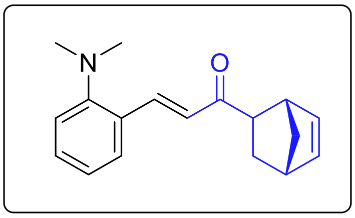


**$^1$H NMR** (500 MHz, Chloroform-*d*) δ 7.98 (s, 1H), 7.57 (s, 1H), 7.34 (s, 1H), 7.06 (d, *J* = 1.1 Hz, 2H), 6.77 (s, 1H), 6.23 (s, 2H), 3.09 (s, 1H), 2.97 (s, 1H), 2.81 (s, 1H), 2.78 (s, 6H), 2.03 (s, 1H), 1.51 (s, 1H), 1.42 (d, *J* = 11.9 Hz, 2H).

**$^{13}$C NMR** (126 MHz, Chloroform-*d*) δ 200.9, 153.9, 140.2, 137.4, 131.9, 130.7, 128.5, 128.1, 125.5, 122.2, 118.4, 49.9, 46.4, 45.0, 42.9, 28.3.

**HRMS** (ESI, m/z) calcd for $C_{18}H_{22}NO^+$ $[M+H]^+$: 268.1696; found: 268.1700.

## (E)-2-(2-(dimethylamino)benzylidene)cyclobutan-1-one (1ab)

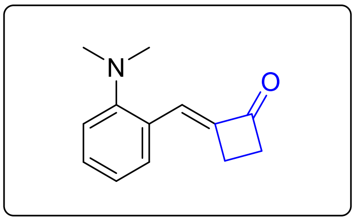


**$^1$H NMR** (500 MHz, Chloroform-*d*) δ 7.55 (dd, *J* = 7.8, 1.6 Hz, 1H), 7.44 (t, *J* = 2.8 Hz, 1H), 7.31 (ddd, *J* = 8.7, 7.3, 1.6 Hz, 1H), 7.05 (dd, *J* = 8.1, 1.2 Hz, 1H), 7.00 (td, *J* = 7.5, 1.2 Hz, 1H), 3.12 – 3.07 (m, 2H), 2.95 (ddd, *J* = 9.8, 6.6, 2.8 Hz, 2H), 2.73 (s, 6H).

**$^{13}$C NMR** (126 MHz, Chloroform-*d*) δ 200.1, 155.2, 144.7, 130.7, 129.0, 127.6, 124.4, 122.0, 118.7, 45.3, 45.2, 23.2.

**HRMS** (ESI, m/z) calcd for $C_{13}H_{16}NO^+$ $[M+H]^+$: 202.1226; found: 202.1221.

## (E)-3-(2-(dimethylamino)benzylidene)tetrahydro-4H-pyran-4-one (1ac)

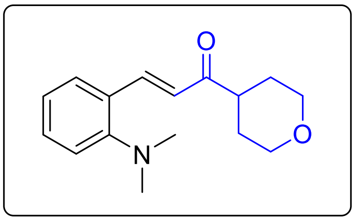


**$^1$H NMR** (500 MHz, Chloroform-*d*) δ 7.94 (dd, *J* = 16.3, 1.6 Hz, 1H), 7.47 (dd, *J* = 7.8, 1.8 Hz, 1H),

7.25 (td, $J$ = 7.5, 1.9 Hz, 1H), 7.00 – 6.90 (m, 2H), 6.71 (dd, $J$ = 16.3, 1.5 Hz, 1H), 3.96 (dd, $J$ = 11.6, 3.4 Hz, 2H), 3.47 – 3.36 (m, 2H), 2.89 (dd, $J$ = 6.4, 4.2 Hz, 1H), 2.68 (s, 6H), 1.74 (d, $J$ = 2.5 Hz, 4H).

**$^{13}$C NMR** (126 MHz, Chloroform-*d*) δ 201.4, 154.0, 141.1, 131.0, 128.1, 128.0, 123.5, 122.2, 118.4, 67.2, 45.6, 45.0, 28.6.

**HRMS** (ESI, m/z) calcd for $C_{16}H_{22}NO_2^+$ $[M+H]^+$: 260.1645; found: 232.1649.

## (E)-2-(2-(dimethylamino)benzylidene)cyclooctan-1-one (1ad)

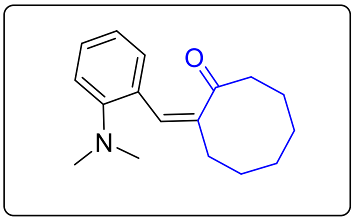

**$^1$H NMR** (500 MHz, Chloroform-*d*) δ 7.53 (s, 1H), 7.29 – 7.24 (m, 2H), 7.02 – 6.99 (m, 1H), 6.98 – 6.94 (m, 1H), 2.84 – 2.80 (m, 2H), 2.70 (s, 8H), 1.86 (p, $J$ = 6.2 Hz, 2H), 1.71 (p, $J$ = 6.3 Hz, 2H), 1.64 (dt, $J$ = 11.4, 5.9 Hz, 2H), 1.55 – 1.51 (m, 2H).

**$^{13}$C NMR** (126 MHz, Chloroform-*d*) δ 208.0, 153.7, 138.8, 136.1, 130.2, 129.3, 129.1, 120.9, 117.5, 44.3, 39.4, 30.1, 29.6, 26.7, 25.9, 25.8.

**HRMS** (ESI, m/z) calcd for $C_{17}H_{24}NO^+$ $[M+H]^+$: 258.1852; found: 258.1853.

## (1R,4S)-3-((E)-2-(dimethylamino)benzylidene)bicyclo[2.2.1]heptan-2-one (1ae)

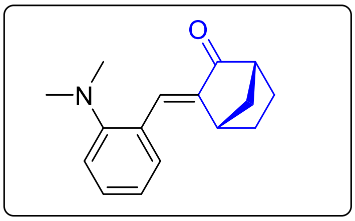

**$^1$H NMR** (500 MHz, Chloroform-*d*) δ 7.41 (s, 1H), 7.36 (d, $J$ = 7.5 Hz, 1H), 7.28 – 7.22 (m, 1H), 7.02 – 6.93 (m, 2H), 3.53 (d, $J$ = 4.3 Hz, 1H), 2.77 (d, $J$ = 4.6 Hz, 1H), 2.69 (d, $J$ = 3.3 Hz, 6H), 2.05 – 1.97 (m, 1H), 1.93 (tt, $J$ = 12.2, 4.0 Hz, 1H), 1.76 – 1.68 (m, 2H), 1.65 (d, $J$ = 9.4 Hz, 2H).

**$^{13}$C NMR** (126 MHz, Chloroform-*d*) δ 207.1, 154.0, 140.3, 130.0, 129.6, 128.4, 125.9, 121.4, 117.9, 49.1, 44.6, 40.3, 38.1, 27.5, 24.5.

**HRMS** (ESI, m/z) calcd for $C_{16}H_{20}NO^+$ $[M+H]^+$: 242.1539; found:.242.1533

## (1E,4E)-1,5-bis(2-(dimethylamino)phenyl)penta-1,4-dien-3-one (1af)

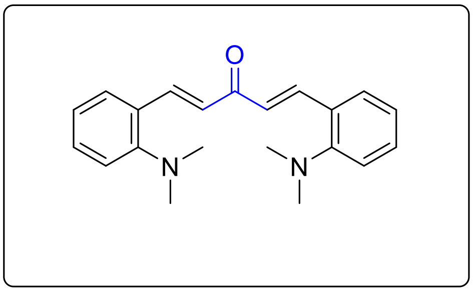

**$^{1}$H NMR** (500 MHz, Chloroform-*d*) δ 8.11 (d, *J* = 16.1 Hz, 2H), 7.62 (dd, *J* = 7.8, 1.6 Hz, 2H), 7.35 (ddd, *J* = 8.5, 7.3, 1.6 Hz, 2H), 7.12 – 7.02 (m, 6H), 2.79 (s, 12H).

**$^{13}$C NMR** (126 MHz, Chloroform-*d*) δ 190.2, 154.0, 141.2, 130.8, 128.6, 128.3, 125.1, 122.1, 118.4, 45.0.

**HRMS** (ESI, m/z) calcd for $C_{21}H_{25}N_2O^+$ [M+H]$^+$: 321.1961; found: 321.1956.

## (E)-3-(2-(dimethylamino)-5-methylphenyl)-1-phenylprop-2-en-1-one (1ag)

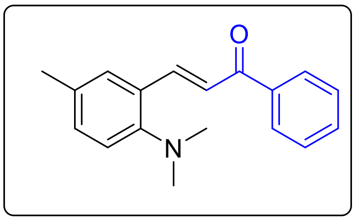

**$^{1}$H NMR** (500 MHz, Chloroform-*d*) δ 8.23 (dd, *J* = 15.8, 11.3 Hz, 1H), 8.09 – 8.04 (m, 2H), 7.60 – 7.48 (m, 5H), 7.20 – 7.14 (m, 1H), 6.99 (dd, *J* = 8.2, 2.9 Hz, 1H), 2.75 (d, *J* = 2.3 Hz, 6H), 2.35 (s, 3H).

**$^{13}$C NMR** (126 MHz, Chloroform-*d*) δ 191.1, 152.1, 143.2, 143.2, 138.6, 132.6, 131.9, 131.9, 131.6, 128.7, 128.6, 128.6, 128.5, 121.5, 118.6, 45.3, 20.7.

**HRMS** (ESI, m/z) calcd for $C_{18}H_{20}NO^+$ [M+H]$^+$: 266.1539; found: 266.1535.

## (E)-3-(2-(dimethylamino)-5-(trifluoromethyl)phenyl)-1-phenylprop-2-en-1-one (1ah)

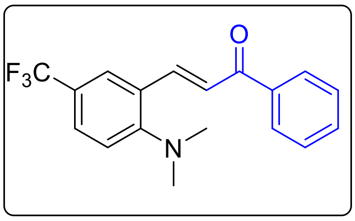

**$^{1}$H NMR** (500 MHz, Chloroform-*d*) δ 8.11 – 8.04 (m, 3H), 7.83 (d, *J* = 2.3 Hz, 1H), 7.63 – 7.57 (m, 1H), 7.56 – 7.47 (m, 4H), 7.07 (d, *J* = 8.5 Hz, 1H), 2.86 (s, 6H).

**$^{13}$C NMR** (126 MHz, Chloroform-*d*) δ 190.4, 156.3, 142.2, 138.1, 132.9, 128.7, 128.5, 127.6, 127.4 (q, *J* = 3.7 Hz), 125.6 (q, *J* = 3.7 Hz), 123.3, 122.1, 117.9, 44.4.

**$^{19}$F NMR** (471 MHz, Chloroform-*d*) δ -61.72.

**HRMS** (ESI, m/z) calcd for $C_{18}H_{17}NO^+$ $[M+H]^+$: 320.1257; found:.320.1254

## (E)-3-(5-bromo-2-(dimethylamino)phenyl)-1-phenylprop-2-en-1-one (1ai)

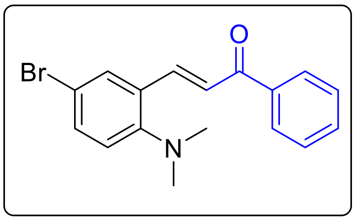


$^1$H NMR (500 MHz, Chloroform-*d*) δ 8.10 – 8.02 (m, 3H), 7.73 (d, *J* = 2.4 Hz, 1H), 7.62 – 7.56 (m, 1H), 7.54 – 7.45 (m, 3H), 7.41 (dd, *J* = 8.6, 2.4 Hz, 1H), 6.92 (d, *J* = 8.6 Hz, 1H), 2.76 (s, 6H).
$^{13}$C NMR (126 MHz, Chloroform-*d*) δ 190.5, 153.1, 141.6, 138.2, 133.4, 132.8, 130.7, 130.4, 128.7, 128.5, 122.2, 120.2, 114.6, 44.9.
**HRMS** (ESI, m/z) calcd for $C_{17}H_{17}BrNO^+$ $[M+Na]^+$: 352.0307; found: 352.0307

## (E)-3-(2-(dimethylamino)-4-methylphenyl)-1-phenylprop-2-en-1-one (1aj)

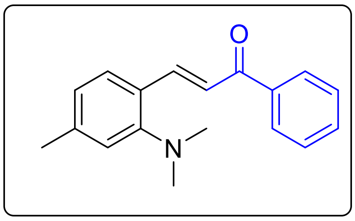


**$^1$H NMR** (500 MHz, Chloroform-*d*) δ 8.18 (d, *J* = 15.7 Hz, 1H), 8.08 – 7.98 (m, 2H), 7.56 (dd, *J* = 7.7, 1.8 Hz, 2H), 7.52 – 7.44 (m, 3H), 6.90 – 6.81 (m, 2H), 2.78 (s, 6H), 2.37 (s, 3H).
**$^{13}$C NMR** (126 MHz, Chloroform-*d*) δ 191.2, 154.3, 143.3, 141.6, 138.7, 132.5, 132.5, 128.6, 128.5, 128.4, 125.7, 123.0, 120.6, 119.2, 45.1, 21.3.
**HRMS** (ESI, m/z) calcd for $C_{18}H_{20}NO^+$ $[M+H]^+$: 266.1539; found: 266.1538.

## (E)-3-(2-(dimethylamino)-4-methoxyphenyl)-1-phenylprop-2-en-1-one (1ak)

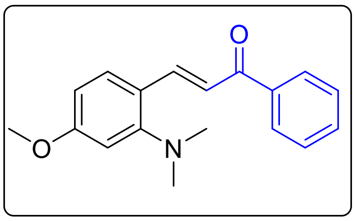


**$^1$H NMR** (500 MHz, Chloroform-*d*) δ 8.11 (d, *J* = 15.7 Hz, 1H), 8.04 – 7.99 (m, 2H), 7.63 – 7.58 (m, 1H), 7.58 – 7.53 (m, 1H), 7.49 (t, *J* = 7.6 Hz, 2H), 7.42 (d, *J* = 15.7 Hz, 1H), 6.58 (d, *J* = 6.3 Hz, 2H), 3.83 (s, 3H), 2.78 (s, 6H).
**$^{13}$C NMR** (126 MHz, Chloroform-*d*) δ 191.1, 162.3, 156.1, 143.1, 138.8, 132.3, 130.1, 128.5, 128.4, 121.2, 119.3, 107.3, 104.6, 55.3, 44.9.

**HRMS** (ESI, m/z) calcd for $C_{18}H_{20}NO_2^+$ $[M+H]^+$: 282.1489; found: 282.1487.

**(E)-3-(4-bromo-2-(dimethylamino)phenyl)-1-phenylprop-2-en-1-one (1al)**

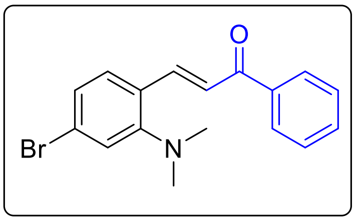


**$^1$H NMR** (500 MHz, Chloroform-*d*) δ 8.05 – 7.97 (m, 3H), 7.54 – 7.39 (m, 5H), 7.12 – 7.03 (m, 2H), 2.73 – 2.66 (m, 6H).

**$^{13}$C NMR** (126 MHz, Chloroform-*d*) δ 190.4, 155.0, 142.0, 142.0, 138.3, 132.7, 129.6, 128.6, 128.5, 127.1, 125.1, 124.9, 121.7, 121.7, 121.5, 121.5, 44.7.

**HRMS** (ESI, m/z) calcd for $C_{17}H_{18}BNO^+$ $[M+H]^+$: 330.0488; found: 330.0489.

**(E)-3-(2-(dimethylamino)-3-fluorophenyl)-1-phenylprop-2-en-1-one(1am)**

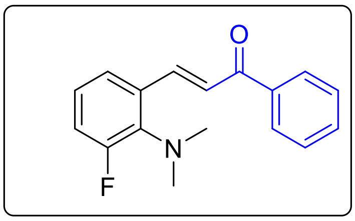


**$^1$H NMR** (500 MHz, Chloroform-*d*) δ 8.06 – 8.01 (m, 2H), 7.97 (d, *J* = 16.1 Hz, 1H), 7.77 (dd, *J* = 16.0, 1.3 Hz, 1H), 7.57 – 7.51 (m, 1H), 7.47 (t, *J* = 7.5 Hz, 2H), 7.21 (td, *J* = 8.2, 6.3 Hz, 1H), 6.78 (d, *J* = 8.2 Hz, 1H), 6.72 (dd, *J* = 10.8, 8.2 Hz, 1H), 2.75 (s, 6H).

**$^{13}$C NMR** (126 MHz, Chloroform-*d*) δ 191.0, 156.0, 156.0, 138.3, 136.5, 132.7, 131.0, 130.9, 128.6, 128.5, 125.3, 125.2, 116.2, 114.0, 114.0, 109.2, 109.0, 44.9.

**$^{19}$F NMR** (471 MHz, Chloroform-*d*) δ -109.91.

**HRMS** (ESI, m/z) calcd for $C_{17}H_{18}FNO^+$ $[M+H]^+$:270.1289; found: 270.1287.

**(E)-3-(3-chloro-2-(dimethylamino)phenyl)-1-phenylprop-2-en-1-one (1an) 2:1**

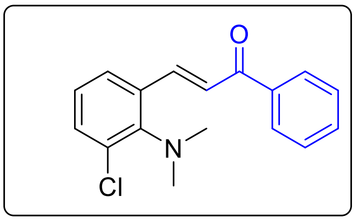


**$^1$H NMR** (500 MHz, Chloroform-*d*) δ 8.06 – 7.98 (m, 2H), 7.93 – 7.78 (m, 2H), 7.61 – 7.55 (m, 1H), 7.53 – 7.47 (m, 1H), 7.36 – 7.29 (m, 1H), 7.19 (t, *J* = 8.0 Hz, 1H), 7.10 – 6.94 (m, 3H), 2.76 (s, 4H), 2.47 (s, 2H).

**$^{13}$C NMR** (126 MHz, Chloroform-*d*) δ 191.5, 191.3, 155.5, 139.9, 138.3, 135.2, 135.0, 132.8, 132.4,

130.1, 129.1, 128.6, 128.5, 128.0, 127.6, 126.9, 126.3, 123.7, 122.8, 116.8, 116.4, 44.5, 43.2.
**HRMS** (ESI, m/z) calcd for $C_{17}H_{17}ClNO^+$ $[M+Na]^+$: 352.0307; found: 352.0307.

### (E)-3-(2-(ethyl(methyl)amino)phenyl)-1-phenylprop-2-en-1-one (1ao)

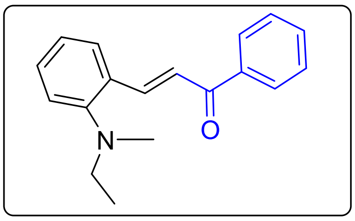

**$^1$H NMR** (500 MHz, Chloroform-*d*) δ 8.24 (d, *J* = 15.8 Hz, 1H), 8.08 – 8.02 (m, 2H), 7.68 (dd, *J* = 7.8, 1.7 Hz, 1H), 7.59 – 7.46 (m, 4H), 7.34 (ddd, *J* = 8.5, 7.3, 1.6 Hz, 1H), 7.10 – 7.01 (m, 2H), 3.01 (q, *J* = 7.1 Hz, 2H), 2.75 (s, 3H), 1.13 (t, *J* = 7.1 Hz, 3H).
**$^{13}$C NMR** (126 MHz, Chloroform-*d*) δ 191.0, 153.9, 143.3, 138.6, 132.6, 132.6, 130.9, 129.2, 128.6, 128.5, 128.6, 122.2, 121.4, 121.4, 119.8, 52.1, 52.1, 40.7, 12.6.
**HRMS** (ESI, m/z) calcd for $C_{18}H_{20}NO^+$ $[M+H]^+$: 266.1539; found: 266.1538.

### (E)-3-(2-(allyl(methyl)amino)phenyl)-1-phenylprop-2-e (1ap)

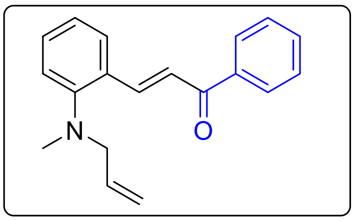

**$^1$H NMR** (500 MHz, Chloroform-*d*) δ 8.24 (d, *J* = 15.9 Hz, 1H), 8.08 – 8.01 (m, 2H), 7.68 (dd, *J* = 7.7, 1.6 Hz, 1H), 7.59 – 7.54 (m, 1H), 7.53 – 7.46 (m, 3H), 7.34 (ddd, *J* = 8.6, 7.2, 1.6 Hz, 1H), 7.09 – 7.02 (m, 2H), 5.91 (ddt, *J* = 16.4, 10.2, 6.1 Hz, 1H), 5.29 – 5.13 (m, 2H), 3.56 (dt, *J* = 6.1, 1.5 Hz, 2H), 2.74 (s, 3H).
**$^{13}$C NMR** (126 MHz, Chloroform-*d*) δ 191.0, 153.5, 143.1, 138.5, 134.8, 132.6, 132.6, 131.0, 128.9, 128.6, 128.6, 128.2, 122.3, 121.7, 121.7, 119.6, 117.7, 61.1, 61.1, 40.6.
**HRMS** (ESI, m/z) calcd for $C_{19}H_{20}NO^+$ $[M+H]^+$: 278.1539; found: 278.1541.

### methyl (E)-3-(2-(dimethylamino)phenyl)acrylate (4a)

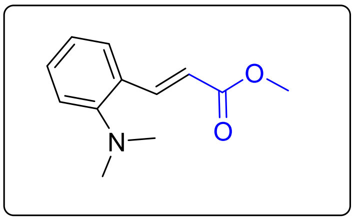

**$^1$H NMR** (500 MHz, Chloroform-*d*) δ 8.06 (d, *J* = 16.2 Hz, 1H), 7.50 (dd, *J* = 7.6, 1.6 Hz, 1H), 7.32

(ddd, *J* = 8.6, 7.3, 1.7 Hz, 1H), 7.05 (dd, *J* = 8.1, 1.1 Hz, 1H), 7.03 – 6.98 (m, 1H), 6.41 (d, *J* = 16.2 Hz, 1H), 3.81 (s, 3H), 2.76 (s, 6H).

**$^{13}$C NMR** (126 MHz, Chloroform-*d*) δ 168.0, 153.7, 143.1, 130.7, 128.2, 128.1, 122.1, 118.4, 117.0, 51.6, 45.0.

**HRMS** (ESI, m/z) calcd for $C_{12}H_{16}NO^+$ $[M+H]^+$: 206.1176; found: 206.1171.

## ethyl (E)-3-(2-(dimethylamino)phenyl)acrylate (4b)

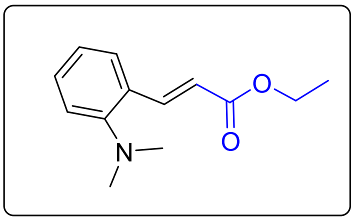

**$^{1}$H NMR** (500 MHz, Chloroform-*d*) δ 8.06 (d, *J* = 16.1 Hz, 1H), 7.50 (dd, *J* = 7.8, 1.7 Hz, 1H), 7.34 – 7.28 (m, 1H), 7.04 (dd, *J* = 8.2, 1.1 Hz, 1H), 7.02 – 6.97 (m, 1H), 6.41 (d, *J* = 16.1 Hz, 1H), 4.27 (q, *J* = 7.1 Hz, 2H), 2.75 (s, 7H), 1.34 (t, *J* = 7.1 Hz, 3H).

**$^{13}$C NMR** (126 MHz, Chloroform-*d*) δ 167.5, 153.6, 142.8, 130.7, 128.2, 128.2, 122.1, 118.3, 117.4, 60.3, 45.0, 14.4.

**HRMS** (ESI, m/z) calcd for $C_{13}H_{18}NO_2^+$ $[M+H]^+$: 2520.1332; found: 220.1325.

## tert-butyl (E)-3-(2-(dimethylamino)phenyl)acrylate (4c)

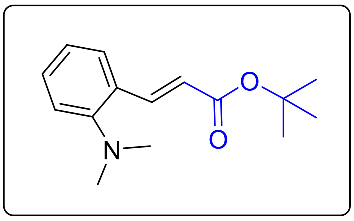

**$^{1}$H NMR** (500 MHz, Chloroform-*d*) δ 7.99 (d, *J* = 16.1 Hz, 1H), 7.52 – 7.45 (m, 1H), 7.28 (ddd, *J* = 8.7, 7.3, 1.6 Hz, 1H), 7.05 – 6.93 (m, 2H), 6.34 (d, *J* = 16.1 Hz, 1H).

**$^{13}$C NMR** (126 MHz, Chloroform-*d*) δ 166.8, 153.5, 141.7, 130.5, 128.3, 128.1, 122.1, 119.2, 118.3, 80.0, 44.9, 28.3.

**HRMS** (ESI, m/z) calcd for $C_{15}H_{22}NO_2^+$ $[M+H]^+$: 248.1645; found: 248.1641.

## benzyl (E)-3-(2-(dimethylamino)phenyl)acrylate (4d)

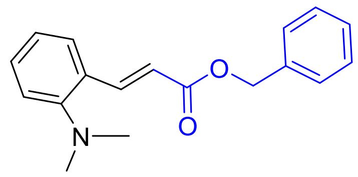


**$^{1}$H NMR** (500 MHz, Chloroform-*d*) δ 8.25 (d, *J* = 15.8 Hz, 1H), 7.58 (dd, *J* = 7.7, 1.8 Hz, 1H), 7.55 – 7.50 (m, 2H), 7.48 – 7.44 (m, 2H), 7.44 – 7.38 (m, 2H), 7.12 – 7.02 (m, 2H), 6.57 (d, *J* = 16.1 Hz, 1H), 5.36 (s, 2H), 2.82 (s, 6H).
**$^{13}$C NMR** (126 MHz, Chloroform-*d*) δ 167.3, 153.8, 143.5, 136.5, 130.9, 128.7, 128.3, 128.3, 128., 128.1, 122.2, 118.5, 117.1, 66.2, 45.0.
**HRMS** (ESI, m/z) calcd for $C_{18}H_{20}NO_2^+$ [M+H]$^+$: 282.1489; found: 282.1491.

## (E)-3-(2-(dimethylamino)phenyl)acrylonitrile (4e) 2:1

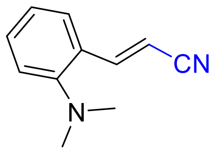


**$^{1}$H NMR** (500 MHz, Chloroform-*d*) δ 8.07 – 7.91 (m, 0H), 7.74 (d, *J* = 16.8 Hz, 1H), 7.40 – 7.31 (m, 2H), 7.10 – 6.97 (m, 2H), 5.86 (d, *J* = 16.8 Hz, 1H), 5.42 (d, *J* = 12.0 Hz, 0H), 2.72 (d, *J* = 1.6 Hz, 6H).
**$^{13}$C NMR** (126 MHz, Chloroform-*d*) δ 153.5, 148.7, 147.6, 131.7, 131.4, 128.9, 127.6, 127.4, 122.6, 122.2, 119.0, 118.9, 118.3, 95.1, 94.0,45.0, 45.0.
**HRMS** (ESI, m/z) calcd for $C_{11}H_{13}N_2O^+$ [M+H]$^+$: 173.1073; found: 173.1074.

## 2,6-di-tert-butyl-4-(2-(dimethylamino)benzylidene)cyclohexa-2,5-dien-1-ol (6a)

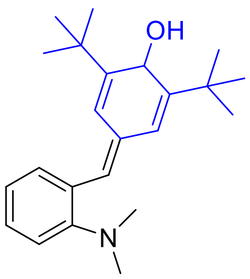


**$^{1}$H NMR** (500 MHz, Chloroform-*d*) δ 7.56 (d, *J* = 2.4 Hz, 1H), 7.39 – 7.33 (m, 2H), 7.32 (s, 1H), 7.11 (d, *J* = 2.4 Hz, 1H), 7.06 (ddd, *J* = 14.9, 7.8, 1.1 Hz, 2H), 2.80 (s, 6H), 1.34 (d, *J* = 18.5 Hz, 19H).

**$^{13}$C NMR** (126 MHz, Chloroform-*d*) δ 186.6, 154.2, 148.8, 147.2, 142.3, 135.4, 132.7, 130.2, 130.1, 128.8, 128.6, 121.5, 117.9, 44.9, 35.4, 35.0, 29.6 (d, *J* = 8.8 Hz).

**HRMS** (ESI, m/z) calcd for $C_{23}H_{34}NO^{+}$ $[M+H]^{+}$: 340.2635; found: 340.2634.

### 2,6-di-tert-butyl-4-(2-(dimethylamino)-5-methylbenzylidene)cyclohexa-2,5-dien-1-ol (6b)

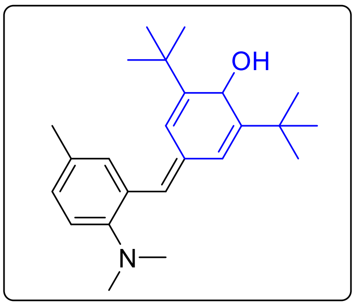


**$^{1}$H NMR** (500 MHz, Chloroform-*d*) δ 7.60 (d, *J* = 2.4 Hz, 1H), 7.35 (s, 1H), 7.23 (d, *J* = 2.1 Hz, 1H), 7.17 (dd, *J* = 8.3, 2.2 Hz, 1H), 7.12 (d, *J* = 2.4 Hz, 1H), 6.99 (d, *J* = 8.2 Hz, 1H), 2.77 (s, 6H), 2.34 (s, 3H), 1.35 (d, *J* = 12.8 Hz, 18H).

**$^{13}$C NMR** (126 MHz, Chloroform-*d*) δ 186.6, 152.1, 148.6, 147.2, 142.2, 135.4, 133.3, 131.0, 130.8, 130.3, 128.9, 128.7, 117.9, 45.1, 35.4, 35.0, 29.6, 29.6, 20.7.

**HRMS** (ESI, m/z) calcd for $C_{24}H_{36}NO^{+}$ $[M+Na]^{+}$: 376.2611; found: 376.2613.

### 4-(5-bromo-2-(dimethylamino)benzylidene)-2,6-di-tert-butylcyclohexa-2,5-dien-1-one (6c)

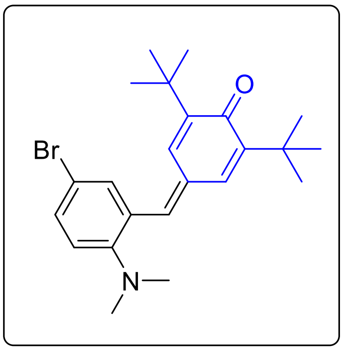


**$^{1}$H NMR** (500 MHz, Chloroform-*d*) δ 7.48 (dd, *J* = 4.0, 2.4 Hz, 2H), 7.42 (dd, *J* = 8.7, 2.4 Hz, 1H), 7.17 (s, 1H), 7.06 (d, *J* = 2.6 Hz, 1H), 6.92 (d, *J* = 8.6 Hz, 1H), 2.77 (s, 6H), 1.33 (d, *J* = 8.0 Hz, 18H).

**$^{13}$C NMR** (126 MHz, Chloroform-*d*) δ 186.5, 153.0 149.4, 147.8, 139.9, 135.0, 134.9, 132.5, 131.0, 130.4, 128.0, 119.5, 113.9, 44.7, 35.5, 35.1, 29.6, 29.5.

**HRMS** (ESI, m/z) calcd for $C_{23}H_{31}NO^{+}$ $[M+H]^{+}$: 416.1584; found: 416.1581.

### 2,6-di-tert-butyl-4-(2-chloro-6-(dimethylamino)benzylidene)cyclohexa-2,5-dien-

**1-one (6d)**

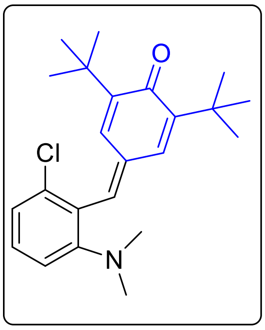


**$^{1}$H NMR** (500 MHz, Chloroform-*d*) δ 7.22 (t, *J* = 8.1 Hz, 1H), 7.08 – 7.04 (m, 2H), 7.03 (s, 1H), 6.97 – 6.91 (m, 2H), 2.69 (s, 6H), 1.28 (d, *J* = 58.0 Hz, 18H).

**$^{13}$C NMR** (126 MHz, Chloroform-*d*) δ 186.8, 154.2, 148.0, 147.6, 138.3, 134.8, 134.2, 132.6, 129.9, 129.6, 126.6, 126.0, 122.6, 116.2, 43.8, 36.0, 35.2, 35.0, 29.6, 29.6.

**HRMS** (ESI, m/z) calcd for $C_{23}H_{31}NO^{+}$ $[M+H]^{+}$: 372.2089; found: 372.2086.

**2,6-di-tert-butyl-4-(2-(dimethylamino)-3-fluorobenzylidene)cyclohexa-2,5-dien-1-one (6e)**

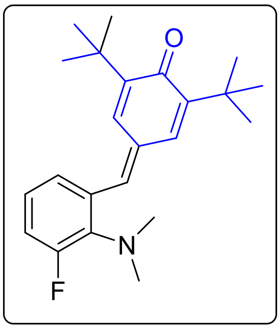


**$^{1}$H NMR** (500 MHz, Chloroform-*d*) δ 7.29 (td, *J* = 8.2, 6.3 Hz, 1H), 7.15 (dd, *J* = 6.0, 2.4 Hz, 1H), 7.09 (d, *J* = 2.5 Hz, 1H), 6.91 (s, 1H), 6.82 (d, *J* = 8.2 Hz, 1H), 6.79 – 6.73 (m, 1H), 2.77 (s, 6H), 1.34 (s, 9H), 1.27 (s, 9H).

**$^{13}$C NMR** (126 MHz, Chloroform-*d*) δ 186.7, 160.9, 158.9, 154.8 (d, *J* = 5.6 Hz), 148.1, 147.3, 134.7 (d, *J* = 4.9 Hz), 130.6 (d, *J* = 10.8 Hz), 129.7 (d, *J* = 6.4 Hz), 116.3 (d, *J* = 15.4 Hz), 113.2 (d, *J* = 2.8 Hz), 108.5 (d, *J* = 23.7 Hz), 44.4, 35.3, 35.0, 29.6 (d, *J* = 1.9 Hz).

**$^{19}$F NMR** (471 MHz, Chloroform-*d*) δ -110.25.

**HRMS** (ESI, m/z) calcd for $C_{23}H_{31}NO^{+}$ $[M+H]^{+}$: 356.2384; found: 356.2387.

**(E)-3-(2-(azetidin-1-yl)phenyl)-1-phenylprop-2-en-1-one (8a)**

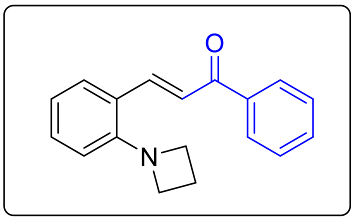


**$^1$H NMR** (500 MHz, Chloroform-*d*) δ 8.09 (d, *J* = 15.4 Hz, 1H), 8.06 – 7.99 (m, 2H), 7.55 (tdd, *J* = 7.8, 5.7, 1.5 Hz, 2H), 7.48 (td, *J* = 7.4, 6.9, 1.3 Hz, 2H), 7.34 (d, *J* = 15.3 Hz, 1H), 7.25 (ddd, *J* = 8.4, 7.1, 1.6 Hz, 1H), 6.81 (t, *J* = 7.4 Hz, 1H), 6.49 (dd, *J* = 8.3, 1.2 Hz, 1H), 4.00 (dd, *J* = 8.3, 6.4 Hz, 4H), 2.34 – 2.25 (m, 2H).

**$^{13}$C NMR** (126 MHz, Chloroform-*d*) δ 190.3, 151.9, 143.2, 138.5, 132.6, 132.6, 131.1, 128.6, 128.5, 128.3, 121.5, 120.6, 120.6, 118.6, 113.6, 55.3, 17.5.

**HRMS** (ESI, m/z) calcd for $C_{18}H_{18}NO^+$ $[M+H]^+$: 264.1383; found: 264.1382.

## (E)-1-phenyl-3-(2-(pyrrolidin-1-yl)phenyl)prop-2-en-1-one (8b)

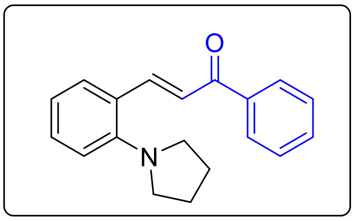


**$^1$H NMR** (500 MHz, Chloroform-*d*) δ 8.21 (d, *J* = 15.4 Hz, 1H), 8.06 – 7.99 (m, 2H), 7.55 (td, *J* = 7.1, 6.7, 1.5 Hz, 2H), 7.51 – 7.45 (m, 2H), 7.35 (d, *J* = 15.4 Hz, 1H), 7.26 (ddd, *J* = 8.6, 7.1, 1.7 Hz, 1H), 6.89 – 6.81 (m, 2H), 3.34 – 3.26 (m, 4H), 1.95 – 1.86 (m, 4H).

**$^{13}$C NMR** (126 MHz, Chloroform-*d*) δ 190.6, 150.6, 145.4, 145.4, 138.6, 132.5, 130.9, 129.1, 128.6, 128.5, 124.6, 120.1, 119.1, 115.5, 52.6, 25.6.

**HRMS** (ESI, m/z) calcd for $C_{17}H_{18}NO_2^+$ $[M+H]^+$: 252.1383; found: 252.1381.

## (E)-1-([1,1'-biphenyl]-4-yl)-3-(2-(pyrrolidin-1-yl)phenyl)prop-2-en-1-one (8d)

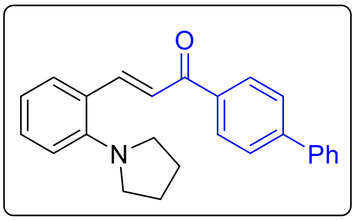


**$^1$H NMR** (500 MHz, Chloroform-*d*) δ 8.27 (d, *J* = 15.4 Hz, 1H), 8.13 (d, *J* = 7.9 Hz, 2H), 7.74 (d, *J* = 8.0 Hz, 2H), 7.67 (d, *J* = 7.7 Hz, 2H), 7.60 (d, *J* = 7.7 Hz, 1H), 7.49 (t, *J* = 7.5 Hz, 2H), 7.45 – 7.37 (m, 2H), 7.29 (t, *J* = 7.8 Hz, 1H), 6.89 (t, *J* = 9.1 Hz, 2H), 3.35 (d, *J* = 6.3 Hz, 4H), 1.95 (d, *J* = 6.1 Hz, 4H).

**$^{13}$C NMR** (126 MHz, Chloroform-*d*) δ 190.0, 150.6, 145.3, 145.3, 140.1, 137.3, 130.9, 129.1, 129.1,

129.0, 128.2, 127.3, 127.2, 120.0, 119.0, 115.5, 52.6, 25.6.

**HRMS** (ESI, m/z) calcd for $C_{25}H_{24}NO^+$ $[M+H]^+$: 354.1852; found: 354.1856.

## (E)-1-(4-isobutylphenyl)-3-(2-(pyrrolidin-1-yl)phenyl)prop-2-en-1-one (8e)

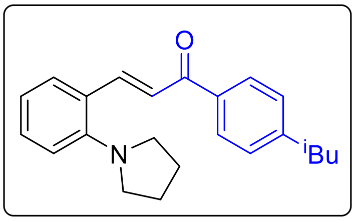


**$^1$H NMR** (500 MHz, Chloroform-*d*) δ 8.23 (d, *J* = 15.5 Hz, 1H), 7.98 (d, *J* = 8.2 Hz, 2H), 7.58 (dd, *J* = 7.7, 1.6 Hz, 1H), 7.39 (d, *J* = 15.4 Hz, 1H), 7.27 (dd, *J* = 8.1, 6.2 Hz, 3H), 6.90 – 6.85 (m, 2H), 3.36 – 3.30 (m, 4H), 2.56 (d, *J* = 7.3 Hz, 2H), 1.95 – 1.91 (m, 4H), 0.94 (d, *J* = 6.6 Hz, 6H).

**$^{13}$C NMR** (126 MHz, Chloroform-*d*) δ 190.2, 150.5, 147.0, 144.9, 136.3, 130.8, 129.3, 129.0, 128.5, 124.8, 120.2, 119.1, 115.4, 52.6, 45.5, 30.2, 25.5, 22.4.

**HRMS** (ESI, m/z) calcd for $C_{23}H_{28}NO^+$ $[M+H]^+$: 334.2165; found: 334.2169.

## (E)-1-(4-methoxyphenyl)-3-(2-(pyrrolidin-1-yl)phenyl)prop-2-en-1-one (8f)

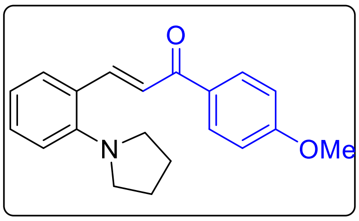


**$^1$H NMR** (500 MHz, Chloroform-*d*) δ 8.20 (d, *J* = 15.4 Hz, 1H), 8.08 – 8.01 (m, 2H), 7.56 (dd, *J* = 7.9, 1.6 Hz, 1H), 7.37 (d, *J* = 15.4 Hz, 1H), 7.26 (td, *J* = 8.2, 7.7, 1.6 Hz, 1H), 7.01 – 6.96 (m, 2H), 6.90 – 6.82 (m, 2H), 3.88 (d, *J* = 1.0 Hz, 3H), 3.36 – 3.26 (m, 4H), 1.98 – 1.89 (m, 4H).

**$^{13}$C NMR** (126 MHz, Chloroform-*d*) δ 188.9, 163.2, 150.5, 144.5, 131.4, 130.7, 130.6, 129.0, 120.0, 119.0, 115.4, 113.8, 55.5, 52.5, 25.5.

**HRMS** (ESI, m/z) calcd for $C_{20}H_{22}NO_2^+$ $[M+H]^+$: 308.1645; found: 308.1642.

## (E)-1-(4-bromophenyl)-3-(2-(pyrrolidin-1-yl)phenyl)prop-2-en-1-one (8g)

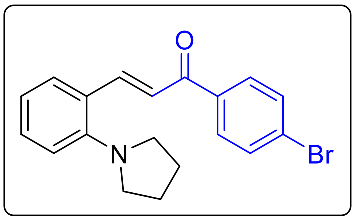


**$^1$H NMR** (500 MHz, Chloroform-*d*) δ 8.23 (d, *J* = 15.4 Hz, 1H), 7.89 (d, *J* = 8.5 Hz, 2H), 7.62 (d, *J* = 8.5 Hz, 2H), 7.56 (dd, *J* = 7.8, 1.7 Hz, 1H), 7.31 – 7.25 (m, 2H), 6.89 – 6.83 (m, 2H), 3.34 –

3.30 (m, 4H), 1.95 – 1.90 (m, 4H).

**$^{13}$C NMR** (126 MHz, Chloroform-*d*) δ 189.4, 150.7, 145.9, 137.3, 131.9, 131.1, 130.0, 129.1, 127.6, 124.3, 119.3, 119.0, 115.5, 52.6, 25.6.

**HRMS** (ESI, m/z) calcd for $C_{19}H_{19}BrNO^+$ $[M+H]^+$: 356.0645; found: 356.0649.

## (E)-1-(4-iodophenyl)-3-(2-(pyrrolidin-1-yl)phenyl)prop-2-en-1-one (8h)

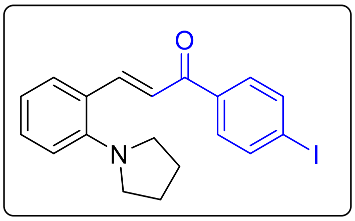

**$^{1}$H NMR** (500 MHz, Chloroform-*d*) δ 8.21 (s, 1H), 7.84 (d, *J* = 8.5 Hz, 2H), 7.73 (d, *J* = 8.5 Hz, 2H), 7.55 (d, *J* = 7.2 Hz, 1H), 7.28 (d, *J* = 15.3 Hz, 2H), 6.87 (d, *J* = 8.7 Hz, 2H), 3.32 (s, 4H), 1.93 (s, 4H).

**$^{13}$C NMR** (126 MHz, Chloroform-*d*) δ 189.6, 150.7, 145.9, 137.9, 131.1, 129.9, 129.1, 124.3, 119.3, 119.0, 115.5, 100.3, 52.7, 25.6.

**HRMS** (ESI, m/z) calcd for $C_{19}H_{19}INO^+$ $[M+H]^+$: 404.0506; found: 404.0507.

## (E)-3-(2-(pyrrolidin-1-yl)phenyl)-1-(o-tolyl)prop-2-en-1-one (8i)

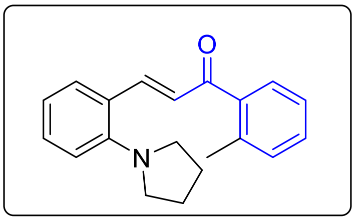

**$^{1}$H NMR** (500 MHz, Chloroform-*d*) δ 7.84 (d, *J* = 16.0 Hz, 1H), 7.56 – 7.52 (m, 1H), 7.49 (d, *J* = 7.3 Hz, 1H), 7.36 (td, *J* = 7.4, 1.5 Hz, 1H), 7.29 – 7.23 (m, 3H), 6.98 (d, *J* = 15.9 Hz, 1H), 6.89 – 6.82 (m, 2H), 3.26 – 3.18 (m, 4H), 2.46 (s, 3H), 1.90 – 1.81 (m, 4H).

**$^{13}$C NMR** (126 MHz, Chloroform-*d*) δ 197.2, 150.4, 146.7, 139.7, 136.6, 131.2, 131.1, 130.1, 128.9, 127.8, 125.4, 125.0, 124.2, 119.3, 115.6, 52.6, 25.4, 20.1.

**HRMS** (ESI, m/z) calcd for $C_{20}H_{24}NO^+$ $[M+H]^+$: 292.1696; found: 292.1693.

## (E)-3-(2-(pyrrolidin-1-yl)phenyl)-1-(2-(trifluoromethyl)phenyl)prop-2-en-1-one (8j)

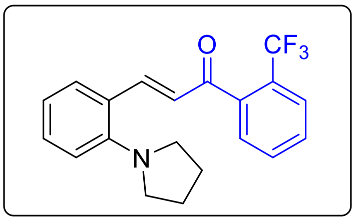


**$^{1}$H NMR** (500 MHz, Chloroform-*d*) δ 7.78 – 7.71 (m, 1H), 7.66 – 7.55 (m, 3H), 7.53 – 7.43 (m, 2H), 7.26 (ddd, *J* = 8.6, 7.3, 1.7 Hz, 1H), 6.91 – 6.75 (m, 3H), 3.23 – 3.08 (m, 4H), 1.91 – 1.74 (m, 4H).

**$^{13}$C NMR** (126 MHz, Chloroform-*d*) δ 195.6, 131.5, 129.6, 129.0, 128.0, 126.7 (q, *J* = 4.9 Hz), 125.1, 124.8, 123.7, 122.7, 119.3, 115.6, 52.6, 25.3.

**$^{19}$F NMR** (471 MHz, Chloroform-*d*) δ -58.09.

**HRMS** (ESI, m/z) calcd for $C_{19}H_{20}F_3NO^{3+}$ $[M+H]^+$: 346.1413; found: 346.1411.

## (E)-1-(3-chlorophenyl)-3-(2-(pyrrolidin-1-yl)phenyl)prop-2-en-1-one (8k)

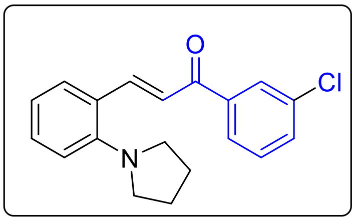


**$^{1}$H NMR** (500 MHz, Chloroform-*d*) δ 8.22 (d, *J* = 15.5 Hz, 1H), 7.98 (t, *J* = 1.9 Hz, 1H), 7.89 (dt, *J* = 7.7, 1.4 Hz, 1H), 7.59 – 7.52 (m, 2H), 7.43 (t, *J* = 7.8 Hz, 1H), 7.31 – 7.25 (m, 2H), 6.90 – 6.83 (m, 2H), 3.36 – 3.30 (m, 4H), 1.97 – 1.91 (m, 4H).

**$^{13}$C NMR** (126 MHz, Chloroform-*d*) δ 189.4, 150.7, 146.3, 140.2, 134.8, 132.4, 131.2, 129.9, 129.10, 128.5, 126.5, 124.2, 119.5, 119.0, 115.5, 52.7, 25.6.

**HRMS** (ESI, m/z) calcd for $C_{17}H_{18}NO^+$ $[M+H]^+$: 312.1150; found: 312.1155.

## (E)-3-(2-(pyrrolidin-1-yl)phenyl)-1-(3,4,5-trimethoxyphenyl)prop-2-en-1-one (8m)

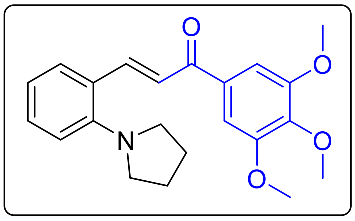


**$^{1}$H NMR** (500 MHz, Chloroform-*d*) δ 8.22 (d, *J* = 15.3 Hz, 1H), 7.56 (dd, *J* = 7.7, 1.6 Hz, 1H), 7.34 – 7.25 (m, 4H), 6.91 – 6.84 (m, 2H), 3.94 (d, *J* = 5.6 Hz, 9H), 3.37 – 3.30 (m, 4H), 1.96 – 1.89 (m, 4H).

**$^{13}$C NMR** (126 MHz, Chloroform-*d*) δ 189.2, 153.1, 150.5, 145.4, 133.9, 130.9, 129.1, 124.6, 119.7, 119.0, 115.5, 106.0, 61.0, 56.4, 52.5, 25.5.

**HRMS** (ESI, m/z) calcd for $C_{22}H_{26}NO_4^+$ [M+H]$^+$: 368.1856; found: 368.1855.

## (E)-1-(naphthalen-2-yl)-3-(2-(pyrrolidin-1-yl)phenyl)prop-2-en-1-one (8n)

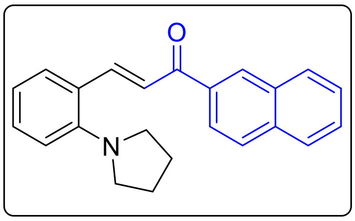

**$^1$H NMR** (500 MHz, Chloroform-*d*) δ 8.57 (d, *J* = 1.7 Hz, 1H), 8.32 (dd, *J* = 15.5, 1.2 Hz, 1H), 8.15 (dd, *J* = 8.6, 1.5 Hz, 1H), 7.99 (dd, *J* = 8.0, 1.4 Hz, 1H), 7.94 (d, *J* = 8.6 Hz, 1H), 7.92 – 7.86 (m, 1H), 7.66 (dd, *J* = 7.7, 1.6 Hz, 1H), 7.64 – 7.50 (m, 3H), 7.30 (ddd, *J* = 8.6, 7.2, 1.7 Hz, 1H), 6.94 – 6.85 (m, 2H), 3.41 – 3.29 (m, 4H), 1.96 – 1.86 (m, 4H).

**$^{13}$C NMR** (126 MHz, Chloroform-*d*) δ 190.5, 150.6, 145.4, 135.9, 135.4, 132.7, 131.0, 129.8, 129.6, 129.2, 128.5, 128.3, 127.9, 126.8, 124.6, 120.1, 120.1, 119.1, 115.5, 52.6, 25.6.

**HRMS** (ESI, m/z) calcd for $C_{23}H_{22}NO^+$ [M+H]$^+$: 328.1696; found: 328.1699.

## (E)-1-(furan-2-yl)-3-(2-(pyrrolidin-1-yl)phenyl)prop-2-en-1-one (8o)

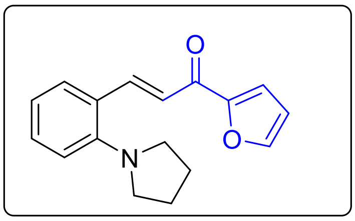

**$^1$H NMR** (500 MHz, Chloroform-*d*) δ 8.26 (d, *J* = 15.6 Hz, 1H), 7.64 (d, *J* = 1.6 Hz, 1H), 7.56 (dd, *J* = 7.8, 1.7 Hz, 1H), 7.29 (d, *J* = 3.5 Hz, 1H), 7.28 – 7.23 (m, 2H), 6.89 – 6.82 (m, 2H), 6.58 (dd, *J* = 3.5, 1.7 Hz, 1H), 3.37 – 3.30 (m, 4H), 1.97 – 1.92 (m, 4H).

**$^{13}$C NMR** (126 MHz, Chloroform-*d*) δ 178.4, 154.0, 150.6, 146.2, 144.5, 130.9, 129.1, 124.4, 119.3, 118.9, 117.0,115.4, 112.4, 52.6, 25.6.

**HRMS** (ESI, m/z) calcd for $C_{17}H_{18}NO_2^+$ [M+H]$^+$: 268.1332; found: 268.1328.

## (E)-3-(2-(pyrrolidin-1-yl)phenyl)-1-(thiophen-2-yl)prop-2-en-1-one (8q)

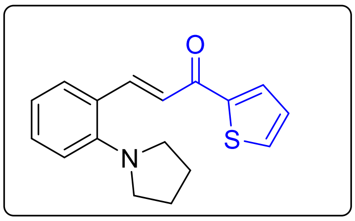

**$^1$H NMR** (500 MHz, Chloroform-*d*) δ 8.28 (d, *J* = 15.3 Hz, 1H), 7.87 (dd, *J* = 3.8, 1.2 Hz, 1H), 7.68 (d, *J* = 4.9 Hz, 1H), 7.58 (dd, *J* = 7.7, 1.6 Hz, 1H), 7.33 – 7.26 (m, 2H), 7.20 (dd, *J* = 4.9, 3.8 Hz,

1H), 6.93 – 6.85 (m, 2H), 3.41 – 3.32 (m, 4H), 1.99 – 1.93 (m, 4H).

**$^{13}$C NMR** (126 MHz, Chloroform-*d*) δ 182.3, 150.6, 146.0, 144.6, 133.4, 131.4, 130.9, 129.1, 128.2, 124.4, 119.7, 119.0, 115.4, 52.6, 25.6.

**HRMS** (ESI, m/z) calcd for $C_{17}H_{20}NOS^+$ [M+H]$^+$: 284.1104; found: 284.1109.

## (E)-3-(5-methyl-2-(pyrrolidin-1-yl)phenyl)-1-phenylprop-2-en-1-one (8r)

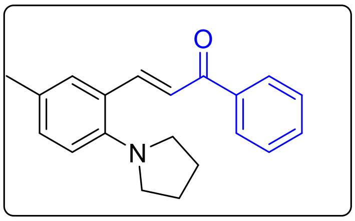


**$^1$H NMR** (500 MHz, Chloroform-*d*) δ 8.22 (d, *J* = 15.4 Hz, 1H), 7.89 (d, *J* = 8.5 Hz, 2H), 7.63 (d, *J* = 8.5 Hz, 2H), 7.56 (dd, *J* = 7.8, 1.7 Hz, 1H), 7.32 – 7.26 (m, 3H), 6.91 – 6.83 (m, 2H), 3.37 – 3.30 (m, 4H), 1.98 – 1.92 (m, 4H).

**$^{13}$C NMR** (126 MHz, Chloroform-*d*) δ 189.5, 150.7, 146.0, 137.3, 131.9, 131.1, 130.0, 129.1, 127.5, 124.3, 119.4, 119.0, 115.5, 52.6, 25.6.

**HRMS** (ESI, m/z) calcd for $C_{20}H_{24}NO^+$ [M+H]$^+$: 292.1696; found: 292.1694.

## (E)-3-(4-methyl-2-(pyrrolidin-1-yl)phenyl)-1-phenylprop-2-en-1-one (8s)

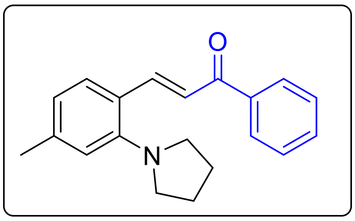


**$^1$H NMR** (500 MHz, Chloroform-*d*) δ 8.23 (d, *J* = 15.4 Hz, 1H), 8.07 – 8.01 (m, 2H), 7.60 – 7.54 (m, 1H), 7.50 (dd, *J* = 8.3, 6.5 Hz, 3H), 7.35 (d, *J* = 15.4 Hz, 1H), 6.70 (d, *J* = 6.4 Hz, 2H), 3.37 – 3.30 (m, 4H), 2.35 (s, 3H), 1.96 – 1.89 (m, 4H).

**$^{13}$C NMR** (126 MHz, Chloroform-*d*) δ 190.7, 150.7, 145.4, 141.4, 138.8, 132.4, 129.0, 128.6, 128.4, 122.0, 120.3, 119.2, 116.2, 52.6, 25.5, 21.9.

**HRMS** (ESI, m/z) calcd for $C_{20}H_{24}NO^+$ [M+H]$^+$: 292.1696; found: 292.1699.

## (E)-1-(2-(dimethylamino)phenyl)-5-phenylpent-1-en-3-one (8t)

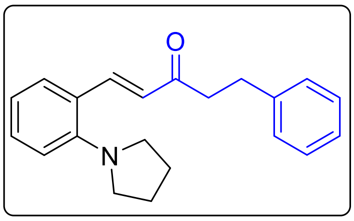

$H^+$

**$^{1}$H NMR** (500 MHz, Chloroform-*d*) δ 7.95 (d, *J* = 16.1 Hz, 1H), 7.45 (dd, *J* = 7.8, 1.7 Hz, 1H), 7.34 – 7.30 (m, 2H), 7.28 – 7.22 (m, 4H), 6.88 (ddd, *J* = 15.6, 8.0, 1.1 Hz, 2H), 6.58 (d, *J* = 16.0 Hz, 1H), 3.31 – 3.25 (m, 4H), 3.06 – 2.99 (m, 4H), 1.97 – 1.90 (m, 4H).

**$^{13}$C NMR** (126 MHz, Chloroform-*d*) δ 199.8, 150.4, 143.1, 141.4, 130.8, 128.9, 128.5, 128.5, 126.1, 124.6, 124.5, 119.4, 115.5, 52.6, 42.2, 30.5, 25.5.

**HRMS** (ESI, m/z) calcd for $C_{21}H_{26}NO^+$ $[M+H]^+$: 306.1852; found: 306.1850.

## (E)-1-(2-(dimethylamino)phenyl)dec-1-en-3-one (8u)

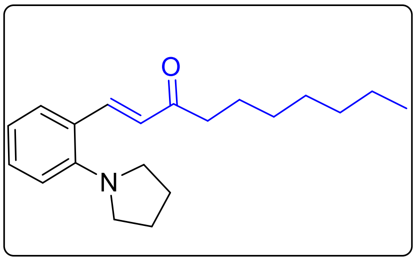

**$^{1}$H NMR** (500 MHz, Chloroform-*d*) δ 7.90 (d, *J* = 16.0 Hz, 1H), 7.43 (dd, *J* = 7.8, 1.7 Hz, 1H), 7.25 – 7.22 (m, 1H), 6.89 – 6.81 (m, 2H), 6.54 (d, *J* = 16.0 Hz, 1H), 3.29 – 3.24 (m, 4H), 2.63 (t, *J* = 7.5 Hz, 2H), 1.96 – 1.92 (m, 4H), 1.71 – 1.64 (m, 2H), 1.36 – 1.31 (m, 4H), 1.29 – 1.26 (m, 4H), 0.87 (s, 3H).

**$^{13}$C NMR** (126 MHz, Chloroform-*d*) δ 201.2, 150.3, 142.7, 130.6, 128.8, 124.8, 119.5, 115.5, 52.6, 40.6, 31.7, 29.4, 29.2, 25.4, 24.7, 22.6, 14.1.

**HRMS** (ESI, m/z) calcd for $C_{20}H_{32}NO^+$ $[M+H]^+$: 300.2322; found: 300.2321.

## (E)-1-acetylferroceneyl-3-(2-(dimethylamino)phenyl)prop-2-en-1-one (1z) (10a)

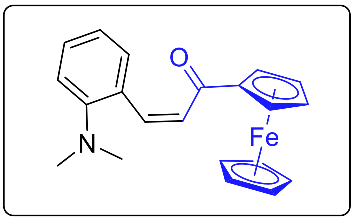

**$^{1}$H NMR** (500 MHz, Chloroform-*d*) δ 8.15 (d, *J* = 15.8 Hz, 1H), 7.63 (dd, *J* = 7.7, 1.6 Hz, 1H), 7.38 – 7.31 (m, 1H), 7.14 – 7.03 (m, 3H), 4.92 (t, *J* = 1.9 Hz, 2H), 4.57 (t, *J* = 2.0 Hz, 2H), 4.22 (s, 5H), 2.81 (s, 6H).

**$^{13}$C NMR** (126 MHz, Chloroform-*d*) δ 193.5, 154.1, 139.3, 130.6, 128.9, 128.2, 122.7, 122.0, 118.5, 80.9, 72.6, 70.1, 69.8, 45.0.

**HRMS** (ESI, m/z) calcd for $C_{21}H_{22}FeNO^+$ $[M+H]^+$: 360.1045; found: 360.1048.

## (E)-4-(3-(2-(dimethylamino)phenyl)acryloyl)phenyl(tert-butoxycarbonyl)-D-valinate (10b)

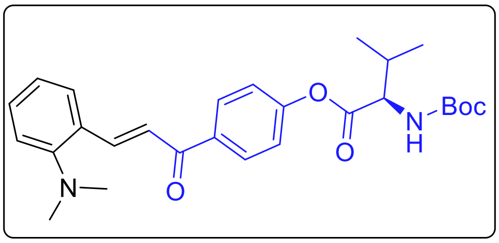


**$^{1}$H NMR** (500 MHz, Chloroform-*d*) δ 8.16 (d, *J* = 15.8 Hz, 1H), 8.06 (d, *J* = 8.4 Hz, 2H), 7.62 (dd, *J* = 7.8, 1.6 Hz, 1H), 7.46 (d, *J* = 15.8 Hz, 1H), 7.37 – 7.30 (m, 1H), 7.25 – 7.20 (m, 2H), 7.07 – 6.98 (m, 2H), 5.13 (s, 1H), 4.47 (dd, *J* = 9.1, 4.9 Hz, 1H), 2.76 (s, 6H), 1.47 (s, 9H), 1.09 (d, *J* = 6.9 Hz, 3H), 1.04 (d, *J* = 6.9 Hz, 3H).

**$^{13}$C NMR** (126 MHz, Chloroform-*d*) δ 189.8, 170.8, 155.8, 154.3, 153.7, 143.6, 136.3, 131.1, 130.2, 128.4, 128.3, 122.0, 121.6, 121.2, 118.5, 80.2, 58.9, 45.0, 31.3, 28.3, 19.2, 17.8.

**HRMS** (ESI, m/z) calcd for $C_{27}H_{35}N_2O_5^+$ $[M+H]^+$: 467.2536; found: 467.2536.

## (E)-1-(tert-butyl)2-(4-(3-(2-(dimethylamino)phenyl)acryloyl)phenyl)(S)-pyrrolidine-1,2-dicarboxylate (10c)

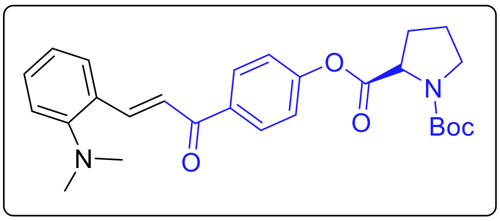


**$^{1}$H NMR** (500 MHz, Chloroform-*d*) δ 8.20 – 8.10 (m, 1H), 8.06 (dd, *J* = 20.8, 8.5 Hz, 1H), 7.97 (d, *J* = 8.7 Hz, 1H), 7.64 – 7.59 (m, 1H), 7.53 – 7.42 (m, 1H), 7.35 – 7.27 (m, 1H), 7.27 – 7.23 (m, 1H), 7.06 – 6.95 (m, 3H), 4.56 – 4.22 (m, 1H), 3.63 – 3.40 (m, 2H), 2.75 (d, *J* = 7.1 Hz, 6H), 2.43 – 2.10 (m, 2H), 2.06 – 1.92 (m, 2H), 1.51 – 1.42 (m, 9H).

**$^{13}$C NMR** (126 MHz, Chloroform-*d*) δ 189.7, 171.1, 161.8, 146.1 – 138.9 (m), 131.2, 131.1, 130.8, 130.2, 130.1, 128.4, 128.3, 122.1, 122.0, 121.7, 121.4, 121.3, 118.5, 118.4, 115.6, 80.4, 59.1, 45.0, 45.0, 31.0, 28.4, 28.4, 24.5, 23.7.

**HRMS** (ESI, m/z) calcd for $C_{27}H_{33}N_2O_5^+$ $[M+H]^+$:465.2384; found: 465.2384.

## (E)-3-(2-(dimethylamino)phenyl)-1-((3S,8S,9S,10R,14S,17R)-3-hydroxy-10-

**methyl-2,3,4,7,8,9,10,11,12,13,14,15,16,17-tetradecahydro-1H-cyclopenta[a]phenanthren-17-yl)prop-2-en-1-one (10d)**

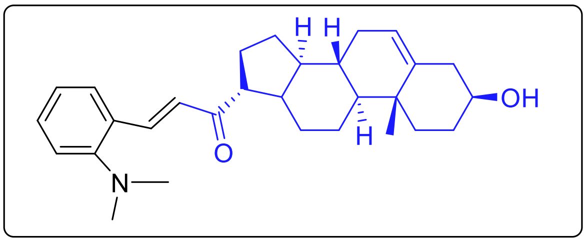


**$^1$H NMR** (500 MHz, Chloroform-*d*) δ 7.90 (d, $J$ = 16.0 Hz, 1H), 7.50 (d, $J$ = 7.7 Hz, 1H), 7.30 (t, $J$ = 7.8 Hz, 1H), 7.06 – 6.96 (m, 2H), 6.72 (d, $J$ = 16.1 Hz, 1H), 5.33 (d, $J$ = 4.5 Hz, 1H), 3.57 – 3.38 (m, 1H), 2.88 (t, $J$ = 8.6 Hz, 1H), 2.73 (s, 6H), 2.29 (ddd, $J$ = 29.7, 24.3, 11.6 Hz, 5H), 2.04 – 1.92 (m, 2H), 1.87 – 1.78 (m, 2H), 1.70 (q, $J$ = 10.2, 9.4 Hz, 2H), 1.63 – 1.40 (m, 7H), 1.25 (td, $J$ = 16.3, 14.0, 6.0 Hz, 3H), 1.07 (td, $J$ = 14.1, 4.1 Hz, 1H), 0.97 (s, 4H), 0.63 (s, 3H).

**$^{13}$C NMR** (126 MHz, Chloroform-*d*) δ 201.0, 154.1, 140.9, 139.9, 130.7, 128.1, 126.6, 122.1, 121.3, 118.4, 71.6, 61.6, 57.1, 50.1, 45.1, 45.0, 42.3, 39.2, 37.3, 36.6, 32.0, 31.9, 31.6, 24.7, 23.0, 21.2, 19.4, 13.5.

**HRMS** (ESI, m/z) calcd for $C_{29}H_{40}NO_2^+$ $[M+H]^+$: 434.3054; found: 434.3049.

## (E)-1-((1r,3R,5S,7s)-adamantan-1-yl)-3-(2-(dimethylamino)phenyl)prop-2-en-1-one (10e)

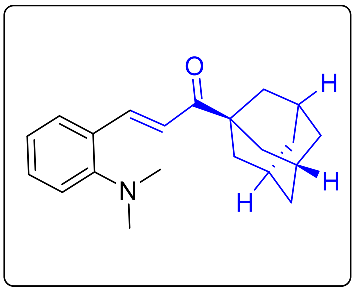


**$^1$H NMR** (500 MHz, Chloroform-*d*) δ 8.03 (d, $J$ = 15.7 Hz, 1H), 7.55 (dd, $J$ = 7.8, 1.6 Hz, 1H), 7.30 (ddd, $J$ = 8.6, 7.3, 1.6 Hz, 1H), 7.13 (d, $J$ = 15.7 Hz, 1H), 7.02 (dd, $J$ = 8.1, 1.2 Hz, 1H), 6.98 (td, $J$ = 7.6, 1.3 Hz, 1H), 2.74 (s, 6H), 2.08 (s, 3H), 1.90 (d, $J$ = 3.0 Hz, 6H), 1.80 – 1.71 (m, 7H).

**$^{13}$C NMR** (126 MHz, Chloroform-*d*) δ 204.2, 154.0, 141.1, 130.6, 128.7, 128.2, 121.9, 119.8, 118.3, 45.5, 44.9, 38.2, 37.4, 36.7, 36.5.

**HRMS** (ESI, m/z) calcd for $C_{21}H_{28}NO^+$ $[M+Na]^+$: 332.1985; found: 332.1989.

## (8S,9S,10R,14S,17R)-17-((E)-3-(2-(dimethylamino)phenyl)acryloyl)-10-methyl-1,2,6,7,8,9,10,11,12,13,14,15,16,17-tetradecahydro-3H-cyclopenta[a]phenanthren-3-one (10f)

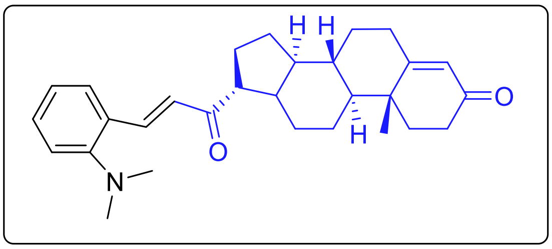

**$^{1}$H NMR** (500 MHz, Chloroform-*d*) δ 7.87 (d, *J* = 16.0 Hz, 1H), 7.46 (d, *J* = 7.8 Hz, 1H), 7.26 (s, 1H), 7.02 – 6.92 (m, 2H), 6.68 (d, *J* = 16.2 Hz, 1H), 5.67 (s, 1H), 2.85 (t, *J* = 8.9 Hz, 1H), 2.69 (s, 6H), 2.42 – 2.16 (m, 7H), 1.96 (d, *J* = 12.3 Hz, 3H), 1.83 (d, *J* = 13.3 Hz, 1H), 1.74 – 1.61 (m, 4H), 1.23 (ddt, *J* = 16.9, 12.1, 6.1 Hz, 3H), 1.12 (s, 3H), 0.98 (dtd, *J* = 27.0, 12.0, 11.3, 3.9 Hz, 3H), 0.63 (s, 3H).

**$^{13}$C NMR** (126 MHz, Chloroform-*d*) δ 200.5, 199.3, 171.1, 154.1, 139.9, 130.8, 128.4, 128.0, 126.4, 123.9, 123.8, 122.1, 118.4, 63.4, 61.4, 56.2, 56.0 53.7, 45.0, 44.8, 39.0, 38.6, 38.6, 35.7, 34.0, 32.8, 32.8, 32.0, 31.9, 24.6, 24.4, 22.9, 22.8, 21.1, 21.0, 17.4, 13.6, 13.3.

**HRMS** (ESI, m/z) calcd for $C_{29}H_{38}NO_2^+$ [M+H]$^+$: 432.2897; found: 432.2893.

## (E)-3,7-dimethylocta-2,6-dien-1-yl (E)-3-(2-(dimethylamino)phenyl)acrylate (10g)

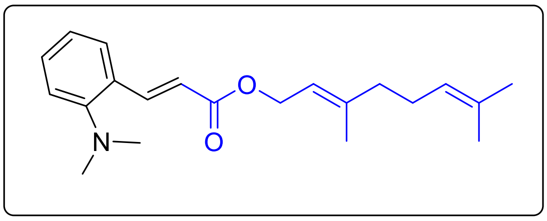

**$^{1}$H NMR** (500 MHz, Chloroform-*d*) δ 8.09 (d, *J* = 16.1 Hz, 1H), 7.48 (dd, *J* = 7.8, 1.6 Hz, 1H), 7.28 (td, *J* = 7.7, 1.6 Hz, 1H), 7.06 – 6.92 (m, 2H), 6.43 (d, *J* = 16.1 Hz, 1H), 5.53 – 5.38 (m, 1H), 5.12 (ddt, *J* = 8.5, 5.5, 1.4 Hz, 1H), 4.75 (d, *J* = 7.1 Hz, 2H), 2.73 (s, 6H), 2.13 (dd, *J* = 8.9, 6.3 Hz, 2H), 2.08 (dd, *J* = 9.2, 6.1 Hz, 2H), 1.76 (d, *J* = 1.5 Hz, 3H), 1.69 (d, *J* = 1.4 Hz, 3H), 1.62 (d, *J* = 1.5 Hz, 3H).

**$^{13}$C NMR** (126 MHz, Chloroform-*d*) δ 167.3, 153.6, 142.8, 141.8, 131.6, 130.7, 128.2, 123.9, 122.2, 118.9, 118.8, 118.3, 117.4, 61.2, 44.9, 39.6, 26.4, 25.7, 17.7, 16.5.

**HRMS** (ESI, m/z) calcd for $C_{21}H_{30}NO_2^+$ [M+H]$^+$: 328.2271; found: 328.2272.

## 2-((1R,5S)-6,6-dimethylbicyclo[3.1.1]hept-2-en-3-yl)ethyl(E)-3-(2-(dimethylamino)phenyl)acrylate (10h)

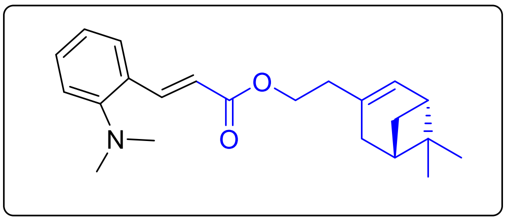

**$^{1}$H NMR** (500 MHz, Chloroform-*d*) δ 8.05 (d, *J* = 16.1 Hz, 1H), 7.50 (dd, *J* = 7.8, 1.6 Hz, 1H), 7.36 – 7.29 (m, 1H), 7.04 (d, *J* = 8.1 Hz, 1H), 7.00 (t, *J* = 7.5 Hz, 1H), 6.39 (d, *J* = 16.2 Hz, 1H), 5.38 – 5.32 (m, 1H), 4.23 (td, *J* = 6.9, 5.2 Hz, 2H), 2.75 (d, *J* = 1.0 Hz, 7H), 2.39 (dt, *J* = 8.8, 5.8 Hz, 3H), 2.31 – 2.19 (m, 2H), 2.11 (pd, *J* = 6.0, 2.3 Hz, 2H), 1.29 (s, 3H), 1.19 (d, *J* = 8.5 Hz, 1H), 0.86 (s, 3H).

**$^{13}$C NMR** (126 MHz, Chloroform-*d*) δ 167.4, 153.6, 144.4, 142.8, 130.7, 128.2, 128.2, 122.1, 118.8, 118.4, 117.4, 62.7, 45.8, 45.0, 40.8, 38.1, 36.1, 31.7, 31.4, 26.3, 21.2.

**HRMS** (ESI, m/z) calcd for $C_{22}H_{30}NO_2^+$ [M+H]$^+$:340.2271; found: 340.2268.

## (1R,2S,5R)-2-isopropyl-5-methylcyclohexyl(E)-3-(2-(dimethylamino)phenyl)acrylate (10i)

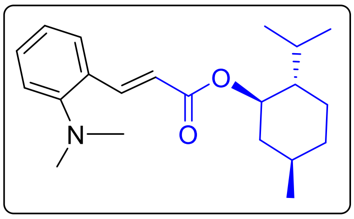


**$^{1}$H NMR** (500 MHz, Chloroform-*d*) δ 8.06 (d, *J* = 16.1 Hz, 1H), 7.53 (dd, *J* = 7.7, 1.6 Hz, 1H), 7.32 (ddd, *J* = 8.7, 7.3, 1.6 Hz, 1H), 7.05 (dd, *J* = 8.2, 1.2 Hz, 1H), 7.03 – 6.96 (m, 1H), 6.45 – 6.37 (m, 1H), 4.89 – 4.78 (m, 1H), 2.77 (s, 6H), 2.16 – 2.09 (m, 1H), 1.98 (dt, *J* = 7.0, 3.5 Hz, 1H), 1.73 (ddd, *J* = 12.8, 4.7, 2.6 Hz, 2H), 1.56 – 1.45 (m, 2H), 1.14 – 1.04 (m, 2H), 0.95 (d, *J* = 1.8 Hz, 3H), 0.94 (d, *J* = 2.1 Hz, 3H), 0.83 (d, *J* = 7.0 Hz, 3H).

**$^{13}$C NMR** (126 MHz, Chloroform-*d*) δ 167.0, 153.6, 142.5, 130.6, 128.2, 128.1, 122.1, 118.3, 117.9, 74.1, 47.3, 44.9, 41.1, 34.4, 31.5, 31.4, 26.5, 23.7, 23.7, 22.1, 20.8, 16.6.

**HRMS** (ESI, m/z) calcd for $C_{21}H_{32}NO_2^+$ [M+H]$^+$: 330.2428; found: 330.2427.

## (R)-3,7-dimethyloct-6-en-1-yl (E)-3-(2-(dimethylamino)phenyl)acrylate (10j)

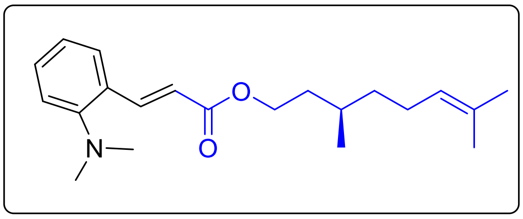


**$^{1}$H NMR** (500 MHz, Chloroform-*d*) δ 8.07 (d, *J* = 16.1 Hz, 1H), 7.50 (dd, *J* = 7.8, 1.7 Hz, 1H), 7.35 – 7.27 (m, 1H), 7.03 (d, *J* = 8.2 Hz, 1H), 6.99 (t, *J* = 7.5 Hz, 1H), 6.41 (d, *J* = 16.2 Hz, 1H), 5.12 (tt, *J* = 7.1, 1.7 Hz, 1H), 4.26 (q, *J* = 6.8 Hz, 2H), 2.75 (s, 6H), 2.02 (dq, *J* = 15.6, 7.4 Hz, 2H), 1.78 (dtd, *J* = 14.4, 7.8, 7.2, 4.3 Hz, 1H), 1.69 (d, *J* = 1.7 Hz, 3H), 1.62 (s, 4H), 1.57 – 1.49 (m, 1H), 1.41 (ddt, *J* = 12.1, 9.5, 6.0 Hz, 1H), 1.24 (dtd, *J* = 9.7, 7.5, 3.9 Hz, 1H), 0.97 (d, *J* = 6.6 Hz, 3H).

**$^{13}$C NMR** (126 MHz, Chloroform-*d*) δ 167.5, 153.6, 142.8, 131.2, 130.7, 128.2, 124.7, 122.1, 118.3, 117.4, 62.9, 44.9, 37.1, 35.6, 29.7, 25.7, 25.5, 19.5, 17.7.

**HRMS** (ESI, m/z) calcd for $C_{21}H_{32}NO_2^+$ $[M+H]^+$: 330.2428; found: 330.2425.

### (3aS,5R,6R,6aS)-5-((S)-2,2-dimethyl-1,3-dioxolan-4-yl)-2,2-dimethyltetrahydrofuro[2,3-d][1,3]dioxol-6-yl(E)-3-(2-(dimethylamino)phenyl)acrylate (10k)

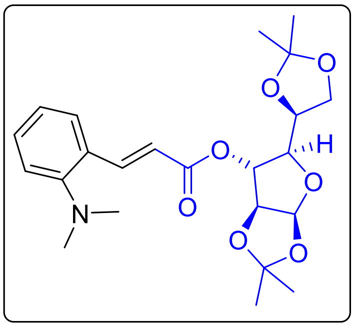

**$^1$H NMR** (500 MHz, Chloroform-*d*) δ 8.08 (d, *J* = 16.2 Hz, 1H), 7.48 (d, *J* = 7.7 Hz, 1H), 7.32 (ddd, *J* = 8.6, 7.4, 1.6 Hz, 1H), 7.07 – 7.02 (m, 1H), 7.01 – 6.95 (m, 1H), 6.38 (d, *J* = 16.2 Hz, 1H), 5.92 (d, *J* = 3.7 Hz, 1H), 5.38 (d, *J* = 2.7 Hz, 1H), 4.59 (d, *J* = 3.7 Hz, 1H), 4.35 – 4.28 (m, 2H), 4.08 (qd, *J* = 8.6, 5.1 Hz, 2H), 2.74 (s, 6H), 1.53 (s, 3H), 1.41 (s, 3H), 1.30 (d, *J* = 6.3 Hz, 6H).
**$^{13}$C NMR** (126 MHz, Chloroform-*d*) δ 166.0, 153.8, 144.2, 131.1, 128.2, 127.7, 122.2, 118.5, 116.1, 112.2, 109.3, 105.1, 83.4, 79.9, 72.6, 67.0, 45.0, 26.8, 26.8, 26.2, 25.3.
**HRMS** (ESI, m/z) calcd for $C_{23}H_{32}NO_7^+$ $[M+H]^+$:434.2173; found: 434.2175.

### (3S,8S,9S,10R,13R,14S,17R)-10,13-dimethyl-17-((R)-6-methylheptan-2-yl)-2,3,4,7,8,9,10,11,12,13,14,15,16,17-tetradecahydro-1H-cyclopenta[a]phenanthren-3-yl (E)-3-(2-(dimethylamino)phenyl)acrylate (10l)

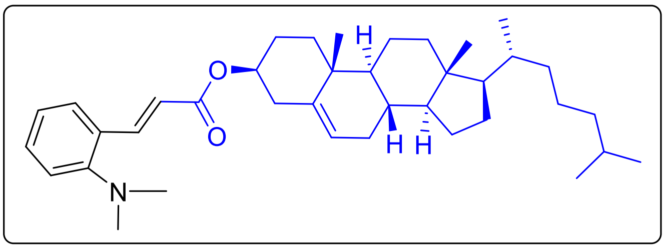

**$^1$H NMR** (500 MHz, Chloroform-*d*) δ 8.03 (d, *J* = 16.1 Hz, 1H), 7.50 (dd, *J* = 7.8, 1.6 Hz, 1H), 7.33 – 7.28 (m, 1H), 7.04 (dd, *J* = 8.1, 1.1 Hz, 1H), 7.02 – 6.96 (m, 1H), 6.39 (d, *J* = 16.1 Hz, 1H), 5.41 (d, *J* = 5.2 Hz, 1H), 4.76 (tdd, *J* = 10.8, 6.5, 4.5 Hz, 1H), 2.76 (s, 6H), 2.43 (s, 2H), 2.03 – 1.97 (m, 2H), 1.88 (s, 3H), 1.75 – 1.42 (m, 10H), 1.40 – 1.25 (m, 5H), 1.20 – 1.07 (m, 8H), 1.06 (s, 3H), 1.04 – 1.00 (m, 3H), 0.93 (d, *J* = 6.5 Hz, 4H), 0.88 (dd, *J* = 6.7, 2.3 Hz, 6H), 0.69 (s, 3H).
**$^{13}$C NMR** (126 MHz, Chloroform-*d*) δ 166.9, 153.6, 142.7, 130.6, 128.2, 122.6, 122.1, 118.3, 117.9, 73.9, 56.7, 56.2, 50.1, 45.0, 42.4, 39.8, 39.6, 38.3, 37.1, 36.7, 36.3, 35.8, 32.0, 31.9, 28.3, 28.0, 28.0, 24.3, 23.9, 22.9, 22.6, 21.1, 19.4, 18.8, 11.9.

**HRMS** (ESI, m/z) calcd for $C_{38}H_{58}NO_2^+$ [M+H]$^+$: 560.4462; found: 560.4458.

## (E)-4-(3-(2-(dimethylamino)phenyl)acryloyl)phenyl(R)-2-(4-isobutylphenyl)propanoate (10m)

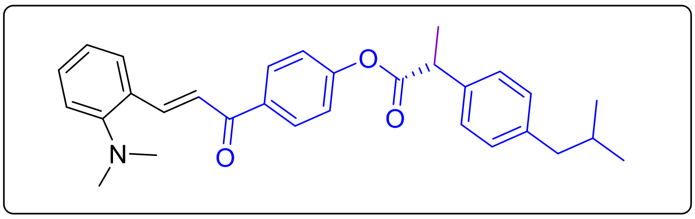


**$^1$H NMR** (500 MHz, Chloroform-*d*) δ 8.19 (d, *J* = 15.7 Hz, 1H), 8.06 (d, *J* = 8.2 Hz, 2H), 7.65 (d, *J* = 7.7 Hz, 1H), 7.48 (d, *J* = 15.8 Hz, 1H), 7.34 (d, *J* = 7.4 Hz, 3H), 7.18 (t, *J* = 8.8 Hz, 4H), 7.09 – 6.99 (m, 2H), 3.99 (d, *J* = 7.2 Hz, 1H), 2.78 (s, 6H), 2.50 (d, *J* = 7.2 Hz, 2H), 1.90 (dt, *J* = 13.7, 6.8 Hz, 1H), 1.65 (d, *J* = 7.2 Hz, 3H), 0.94 (d, *J* = 6.7 Hz, 6H).

**$^{13}$C NMR** (126 MHz, Chloroform-*d*) δ 189.8, 172.8, 154.3, 143.4, 141.0, 137.0, 136.0, 131.1, 130.1, 129.7, 128.4, 128.4, 127.3, 122.1, 121.7, 121.2, 118.5, 45.4, 45.1, 45.0, 30.2, 22.5, 18.5.

**HRMS** (ESI, m/z) calcd for $C_{30}H_{34}NO_3^+$ [M+H]$^+$: 456.2533; found: 456.2532.

## (E)-4-(3-(2-(dimethylamino)phenyl)acryloyl)phenyl (R)-2-(6-methoxynaphthalen-2-yl)propanoate (10n)

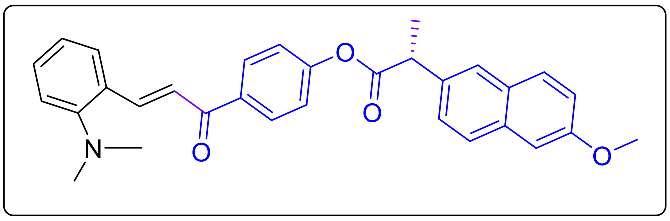


**$^1$H NMR** (500 MHz, Chloroform-*d*) δ 8.19 (d, *J* = 15.8 Hz, 1H), 8.04 (d, *J* = 8.7 Hz, 2H), 7.81 – 7.75 (m, 3H), 7.66 – 7.62 (m, 1H), 7.53 (dd, *J* = 8.6, 1.9 Hz, 1H), 7.47 (dd, *J* = 15.9, 1.4 Hz, 1H), 7.35 (ddd, *J* = 8.5, 7.3, 1.6 Hz, 1H), 7.20 (d, *J* = 8.8 Hz, 1H), 7.18 – 7.14 (m, 3H), 7.08 – 7.01 (m, 2H), 4.18 – 4.11 (m, 1H), 3.92 (s, 4H), 2.78 (s, 6H), 1.74 (d, *J* = 7.1 Hz, 3H).

**$^{13}$C NMR** (126 MHz, Chloroform-*d*) δ 189.8, 172.8, 157.9, 154.3, 143.5, 136.0, 134.9, 134.0, 131.1, 130.1, 129.4, 129.1, 128.5, 128.4, 127.6, 126.3, 126.1, 122.1, 121.7, 121.2, 119.3, 118.5, 105.7, 55.4, 45.7, 45.0, 18.5.

**HRMS** (ESI, m/z) calcd for $C_{31}H_{30}NO_4^+$ [M+H]$^+$: 480.2169; found: 480.2172.

## (E)-4-(3-(2-(dimethylamino)phenyl)acryloyl)phenyl4-(N,N-dipropylsulfamoyl)benzoate (10o)

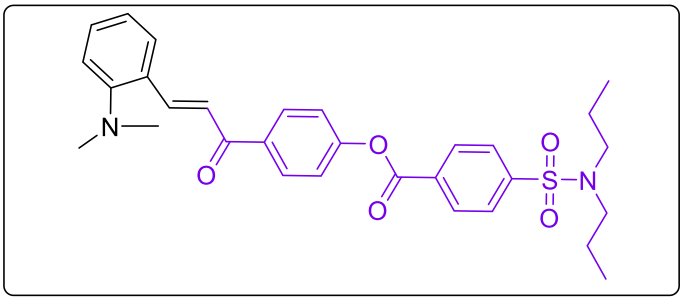


**$^{1}$H NMR** (500 MHz, Chloroform-*d*) δ 8.33 (d, *J* = 8.5 Hz, 2H), 8.19 (d, *J* = 15.8 Hz, 1H), 8.13 (d, *J* = 8.6 Hz, 2H), 7.96 (d, *J* = 8.4 Hz, 2H), 7.64 (dd, *J* = 7.8, 1.6 Hz, 1H), 7.50 (d, *J* = 15.8 Hz, 1H), 7.39 – 7.31 (m, 3H), 7.10 – 6.98 (m, 2H), 3.17 – 3.09 (m, 4H), 2.78 (s, 6H), 1.60 – 1.52 (m, 4H), 0.88 (t, *J* = 7.4 Hz, 6H).

**$^{13}$C NMR** (126 MHz, Chloroform-*d*) δ 189.7, 163.4, 154.3, 153.9, 143.7, 136.5, 132.4, 131.2, 130.9, 130.3, 128.4, 128.3, 127.3, 122.0, 121.7, 121.1, 118.5, 45.0, 45.0, 22.0, 11.2.

**HRMS** (ESI, m/z) calcd for $C_{30}H_{35}N_2O_5^+$ $[M+H]^+$: 535.2261; found: 535.2264.

## (E)-3-(5-chloro-2-(dimethylamino)phenyl)-1-phenylprop-2-en-1-one (12a)

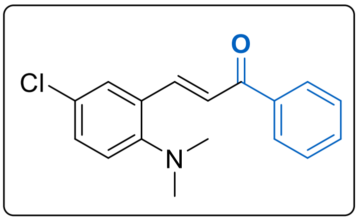


**$^{1}$H NMR** (500 MHz, Chloroform-*d*) δ 8.11 – 8.02 (m, 3H), 7.59 – 7.55 (m, 2H), 7.51 – 7.45 (m, 3H), 7.25 (dd, *J* = 8.7, 2.5 Hz, 1H), 6.95 (d, *J* = 8.6 Hz, 1H), 2.73 (s, 6H).

**$^{13}$C NMR** (126 MHz, Chloroform-*d*) δ 190.4, 152.7, 141.6, 138.2, 132.8, 130.5, 129.9, 128.7, 128.5, 127.8, 127.2, 122.2, 122.2, 119.8, 45.0.

**HRMS** (ESI, m/z) calcd for $C_{17}H_{17}ClNO^+$ $[M+H]^+$: 286.0993; found: 286.0992.

## (E)-3-(5-chloro-2-(dimethylamino)phenyl)-1-(thiophen-2-yl)prop-2-en-1-one (12b)

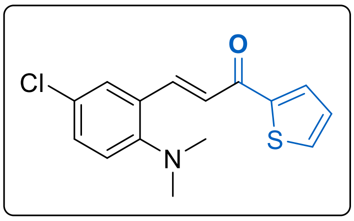


**$^{1}$H NMR** (500 MHz, Chloroform-*d*) δ 8.09 (d, *J* = 15.6 Hz, 1H), 7.87 (d, *J* = 3.9 Hz, 1H), 7.67 (d, *J* = 4.9 Hz, 1H), 7.56 (d, *J* = 2.6 Hz, 1H), 7.35 (d, *J* = 15.7 Hz, 1H), 7.25 (dd, *J* = 8.6, 2.5 Hz, 1H), 7.17 (t, *J* = 4.4 Hz, 1H), 6.96 (dd, *J* = 8.7, 2.0 Hz, 1H), 2.74 (s, 6H).

**$^{13}$C NMR** (126 MHz, Chloroform-*d*) δ 182.1, 152.8, 145.6, 140.9, 133.9, 133.9, 131.9, 130.6, 129.7, 128.3, 127.8, 127.1, 121.9, 121.9, 119.8, 45.0.

**HRMS** (ESI, m/z) calcd for $C_{15}H_{15}ClNOS^+$ $[M+H]^+$: 292.0557; found: 292.0555.

## (E)-3-(5-chloro-2-(dimethylamino)phenyl)-1-cyclopropylprop-2-en-1-one (12c)

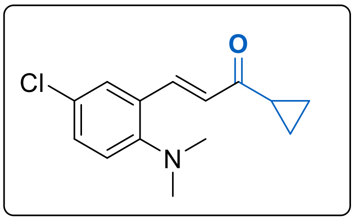


**$^1$H NMR** (500 MHz, Chloroform-*d*) δ 7.86 (dd, *J* = 16.2, 1.5 Hz, 1H), 7.47 (s, 1H), 7.29 – 7.20 (m, 1H), 7.00 – 6.92 (m, 1H), 6.71 (d, *J* = 16.2 Hz, 1H), 2.83 (qd, *J* = 6.9, 1.3 Hz, 1H), 2.72 (d, *J* = 0.9 Hz, 6H), 1.80 – 1.73 (m, 1H), 1.47 (dtd, *J* = 15.6, 6.9, 1.7 Hz, 1H), 1.15 (dd, *J* = 7.0, 1.6 Hz, 3H), 0.91 (td, *J* = 7.4, 1.6 Hz, 3H).

**$^{13}$C NMR** (126 MHz, Chloroform-*d*) δ 204.1, 152.4, 139.2, 130.3, 129.9, 127.6, 127.3, 125.6, 119.8, 45.8, 44.9, 26.4, 16.3, 11.8.

**HRMS** (ESI, m/z) calcd for $C_{14}H_{17}ClNO^+$ $[M+H]^+$: 250.0993; found: 250.0995.

## (E)-1-(5-chloro-2-(dimethylamino)phenyl)-4-methylhex-1-en-3-one (12d)

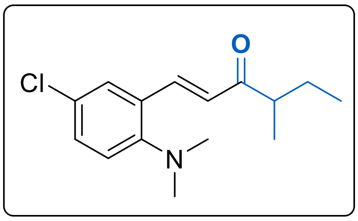


**$^1$H NMR** (500 MHz, Chloroform-*d*) δ 7.86 (d, *J* = 16.3 Hz, 1H), 7.45 (d, *J* = 2.5 Hz, 1H), 7.21 (dd, *J* = 8.6, 2.5 Hz, 1H), 6.92 (d, *J* = 8.6 Hz, 1H), 6.74 (d, *J* = 16.3 Hz, 1H), 2.69 (d, *J* = 0.9 Hz, 6H), 2.29 – 2.21 (m, 1H), 1.12 (dd, *J* = 4.5, 3.3 Hz, 2H), 0.94 (dd, *J* = 7.7, 3.5 Hz, 2H).

**$^{13}$C NMR** (126 MHz, Chloroform-*d*) δ 200.1, 152.4, 138.8, 130.3, 129.9, 127.6, 127.3, 126.88, 119.7, 44.9, 19.3, 11.3, 11.2.

**HRMS** (ESI, m/z) calcd for $C_{14}H_{17}ClNO^+$ $[M+H]^+$: 250.0993; found: 250.0993.

## (E)-3-(5-chloro-2-(dimethylamino)phenyl)-1-phenylprop-Acetylferrocene (12e)

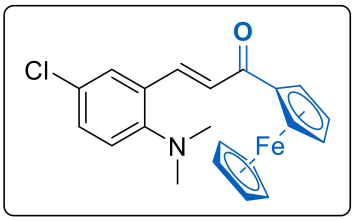


**$^1$H NMR** (500 MHz, Chloroform-*d*) δ 8.05 (d, *J* = 15.7 Hz, 1H), 7.57 (s, 1H), 7.34 – 7.21 (m, 2H), 7.06 (d, *J* = 15.8 Hz, 1H), 7.00 (d, *J* = 8.7 Hz, 1H), 4.92 (s, 2H), 4.60 (s, 2H), 4.22 (s, 5H), 2.77 (s,

6H).
**$^{13}$C NMR** (126 MHz, Chloroform-*d*) δ 193.12, 137.78, 130.09, 127.54, 123.50, 119.86, 72.78, 70.10, 69.80, 44.91.
**HRMS** (ESI, m/z) calcd for $C_{21}H_{21}ClFeNO^+$ $[M+H]^+$: 394.0656; found: 394.0655.

## (E)-3-(5-bromo-2-(ethyl(methyl)amino)phenyl)-1-phenylprop-2-en-1-one (15)

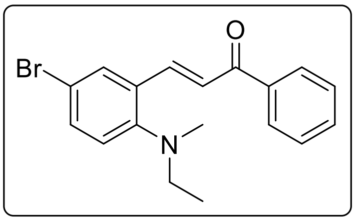


**$^1$H NMR** (500 MHz, Chloroform-*d*) δ 8.11 – 8.00 (m, 3H), 7.75 (d, *J* = 2.4 Hz, 1H), 7.58 (t, *J* = 7.4 Hz, 1H), 7.50 (t, *J* = 7.6 Hz, 2H), 7.46 (d, *J* = 15.8 Hz, 1H), 7.41 (dd, *J* = 8.7, 2.4 Hz, 1H), 6.93 (d, *J* = 8.7 Hz, 1H), 2.98 (q, *J* = 7.1 Hz, 2H), 2.73 (s, 3H), 1.11 (t, *J* = 7.1 Hz, 3H).
**$^{13}$C NMR** (126 MHz, Chloroform-*d*) δ 190.46, 152.71, 141.57, 138.20, 133.29, 132.81, 131.14, 130.55, 128.66, 128.53, 122.13, 122.11, 121.42, 114.79, 51.97, 51.95, 40.54, 12.48.
**HRMS** (ESI, m/z) calcd for $C_{18}H_{19}NO^+$ $[M+H]^+$: 344.0645; found: 344.0642.

## 2-(2-fluoro-1-methyl-1H-indol-3-yl)-1-phenylethan-1-one (3a)

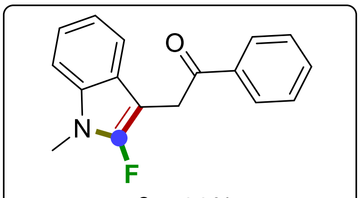


**3a**, 90%

The reaction was performed following the general procedure. The residue was purified by flash column chromatography (silica gel, petroleum ether: $CH_2Cl_2$ =10:1, v/v) to give the product as a yellow solid (48.1 mg, 90%). m.p. 152-154 °C.
**$^1$H NMR** (500 MHz, Chloroform-*d*) δ 8.15 – 8.08 (m, 2H), 7.61 – 7.54 (m, 2H), 7.49 (m, *J* = 8.4, 7.0 Hz, 2H), 7.25 – 7.16 (m, 3H), 4.35 (s, 2H), 3.61 (s, 3H).
**$^{13}$C NMR** (126 MHz, Chloroform-*d*) δ 196.7 (d, *J* = 1.6 Hz), 150.1 (d, *J* = 265.9 Hz), 136.4, 133.2, 130.6, 128.6 (d, *J* = 15.6 Hz), 125.5 (d, *J* = 5.9 Hz), 121.3 (d, *J* = 3.5 Hz), 120.5, 118.9 (d, *J* = 6.3 Hz), 108.9 (d, *J* = 1.8 Hz), 84.1 (d, *J* = 10.8 Hz), 32.9 (d, *J* = 3.0 Hz), 27.9 (d, *J* = 2.1 Hz).
**$^{19}$F NMR** (471 MHz, Chloroform-*d*) δ -138.82.
**HRMS** (ESI, m/z) calcd for $C_{11}H_{15}INO_2[M+H]^+$: 268.1132; found: 268.1124.

## 1-([1,1'-biphenyl]-4-yl)-2-(2-fluoro-1-methyl-1H-indol-3-yl)ethan-1-one (3b)

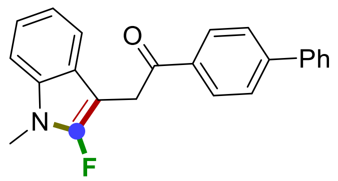


**3b**, 81%

The reaction was performed following the general procedure. The residue was purified by flash column chromatography (silica gel, petroleum ether: $CH_2Cl_2$ =10:1, v/v) to give the product as a yellow solid (55.6 mg, 81%). m.p. 161-163 °C.

**$^1$H NMR** (500 MHz, Chloroform-*d*) δ 8.15 (d, *J* = 8.5 Hz, 2H), 7.69 (d, *J* = 8.5 Hz, 2H), 7.65 – 7.61 (m, 2H), 7.53 (d, *J* = 7.8 Hz, 1H), 7.48 (t, *J* = 7.5 Hz, 2H), 7.43 – 7.39 (m, 1H), 7.23 – 7.19 (m, 2H), 7.16 (m, *J* = 8.1, 4.1 Hz, 1H), 4.35 (s, 2H), 3.64 (s, 3H).

**$^{13}$C NMR** (126 MHz, Chloroform-*d*) δ 196.3, 150.0 (d, *J* = 266.0 Hz), 145.8, 139.9, 135.1, 130.6, 129.1 (d, *J* = 20.0 Hz), 128.2, 127.3 (d, *J* = 1.7 Hz), 125.4 (d, *J* = 5.9 Hz), 121.3 (d, *J* = 3.5 Hz), 120.5, 118.9 (d, *J* = 6.3 Hz), 108.9, 84.1 (d, *J* = 10.8 Hz), 33.0 (d, *J* = 3.0 Hz), 28.0 (d, *J* = 2.1 Hz).

**$^{19}$F NMR** (471 MHz, Chloroform-*d*) δ -138.82.

**HRMS** (ESI, m/z) calcd for $C_{23}H_{18}FNO[M+H]^+$: 344.1445; found: 344.1445.

## ethyl 4-(2-(2-fluoro-1-methyl-1H-indol-3-yl)acetyl)benzoate (3c)

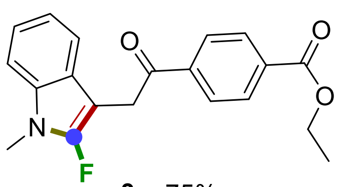


**3c**, 75%

The reaction was performed following the general procedure. The residue was purified by flash column chromatography (silica gel, petroleum ether: $CH_2Cl_2$ =10:1, v/v) to give the product as a yellow solid (50.9 mg, 75%). m.p. 159-161 °C.

**$^1$H NMR** (500 MHz, Chloroform-*d*) δ 8.15 – 8.07 (m, 4H), 7.49 (d, *J* = 7.9 Hz, 1H), 7.20 (d, *J* = 0.9 Hz, 2H), 7.15 (m, *J* = 5.1, 2.5 Hz, 1H), 4.41 (q, *J* = 7.1 Hz, 2H), 4.32 (s, 2H), 3.62 (s, 3H), 1.41 (t, *J* = 7.2 Hz, 3H).

**$^{13}$C NMR** (126 MHz, Chloroform-*d*) δ 196.21, 165.76, 149.99 (d, *J* = 266.1 Hz), 139.50, 134.21, 130.60, 129.78, 128.37, 125.25 (d, *J* = 5.8 Hz), 121.35 (d, *J* = 3.5 Hz), 120.53, 118.72 (d, *J* = 6.2 Hz), 108.91 (d, *J* = 1.8 Hz), 83.59 (d, *J* = 10.6 Hz), 61.44, 33.27 (d, *J* = 2.8 Hz), 27.96 (d, *J* = 1.9 Hz), 14.30.

**$^{19}$F NMR** (471 MHz, Chloroform-*d*) δ -138.62.

**HRMS** (ESI, m/z) calcd for $C_{20}H_{10}FNO_3[M+H]^+$: 340.1343; found: 340.1342.

## 4-(2-(2-fluoro-1-methyl-1H-indol-3-yl)acetyl)benzonitrile (3d)

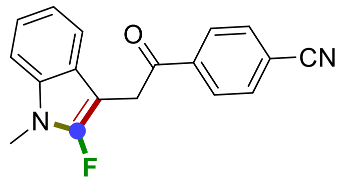


**3d**, 38%

The reaction was performed following the general procedure. The residue was purified by flash column chromatography (silica gel, petroleum ether: $CH_2Cl_2$ =10:1, v/v) to give the product as a yellow solid (22.2 mg, 38%). m.p. 134-136 °C.

**$^1$H NMR** (500 MHz, Chloroform-*d*) δ 8.12 (d, *J* = 8.5 Hz, 2H), 7.75 (d, *J* = 8.4 Hz, 2H), 7.48 – 7.45 (m, 1H), 7.21 (d, *J* = 4.0 Hz, 2H), 7.15 (m, *J* = 8.1, 4.1 Hz, 1H), 4.31 (s, 2H), 3.64 (s, 3H).

**$^{13}$C NMR** (126 MHz, Chloroform-*d*) δ 195.27, 149.93 (d, *J* = 266.2 Hz), 139.27, 132.49, 130.60, 128.89, 125.03 (d, *J* = 5.5 Hz), 121.5 (d, *J* = 3.5 Hz), 120. 7, 118.6 (d, *J* = 6.3 Hz), 117.9, 116.3, 109.0, 83.1 (d, *J* = 10.7 Hz), 33.3 (d, *J* = 2.8 Hz), 28.0(d, *J* = 1.8 Hz).

**$^{19}$F NMR** (471 MHz, Chloroform-*d*) δ -138.50.

**HRMS** (ESI, m/z) calcd for $C_{18}H_{13}FN_2O[M+H]^+$: 293.1085; found: 293.1089.

## 2-(2-fluoro-1-methyl-1H-indol-3-yl)-1-(4-(methylthio)phenyl)ethan-1-one (3e)

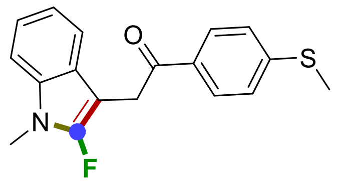


**3e**, 78%

The reaction was performed following the general procedure. The residue was purified by flash column chromatography (silica gel, petroleum ether: $CH_2Cl_2$ =10:1, v/v) to give the product as a yellow solid (48.8 mg, 78%). m.p. 100-102 °C.

**$^1$H NMR** (500 MHz, Chloroform-*d*) δ 7.98 (d, *J* = 8.6 Hz, 2H), 7.51 (m, *J* = 7.8, 1.1 Hz, 1H), 7.26 (d, *J* = 2.4 Hz, 1H), 7.25 (s, 1H), 7.20 (m, *J* = 3.7, 1.1 Hz, 2H), 7.14 (m, *J* = 8.1, 4.9, 3.4 Hz, 1H), 4.27 (s, 2H), 3.62 (s, 3H), 2.50 (s, 3H).

**$^{13}$C NMR** (126 MHz, Chloroform-*d*) δ 195.8 (d, *J* = 1.6 Hz), 149.9 (d, *J* = 265.7 Hz), 145.9, 132.6, 130.6, 128.9, 125.4 (d, *J* = 5.8 Hz), 125.01, 121.2 (d, *J* = 3.5 Hz), 120.4, 118.9 (d, *J* = 6.4 Hz), 108.8 (d, *J* = 1.8 Hz), 84.2 (d, *J* = 10.8 Hz), 32.8 (d, *J* = 3.0 Hz), 28.0 (d, *J* = 2.1 Hz), 14.8.

**$^{19}$F NMR** (471 MHz, Chloroform-*d*) δ -138.89.

**HRMS** (ESI, m/z) calcd for $C_{18}H_{16}FNOS[M+H]^+$: 314.1009; found: 314.1011.

## 1-(4-(diethylamino)phenyl)-2-(2-fluoro-1-methyl-1H-indol-3-yl)ethan-1-one (3f)

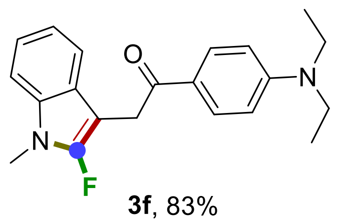

**3f**, 83%

The reaction was performed following the general procedure. The residue was purified by flash column chromatography (silica gel, petroleum ether: $CH_2Cl_2$ =10:1, v/v) to give the product as a yellow oil (56.1 mg, 83%).

**$^1$H NMR** (500 MHz, Chloroform-*d*) δ 7.99 (d, *J* = 9.0 Hz, 2H), 7.58 (d, *J* = 7.7 Hz, 1H), 7.21 – 7.09 (m, 3H), 6.63 (d, *J* = 9.1 Hz, 2H), 4.22 (s, 2H), 3.62 (s, 3H), 3.41 (q, *J* = 7.1 Hz, 5H), 1.20 (t, *J* = 7.1 Hz, 7H).

**$^{13}$C NMR** (126 MHz, Chloroform-*d*) δ 194.6, 151.2, 149.9 (d, *J* = 265.4 Hz), 131.1, 130.6, 125.7 (d, *J* = 6.0 Hz), 123.7, 121.0 (d, *J* = 3.6 Hz), 120.3, 119.2 (d, *J* = 6.4 Hz), 110.2, 108.7 (d, *J* = 1.8 Hz), 85.3 (d, *J* = 10.8 Hz), 44.5, 32.2 (d, *J* = 2.9 Hz), 27.9 (d, *J* = 2.1 Hz), 12.6.

**$^{19}$F NMR** (471 MHz, Chloroform-*d*) δ -139.34.

**HRMS** (ESI, m/z) calcd for $C_{21}H_{23}FN_2O[M+H]^+$: 339.1867; found: 339.1868.

## 2-(2-fluoro-1-methyl-1H-indol-3-yl)-1-(4-methoxyphenyl)ethan-1-one (3g)

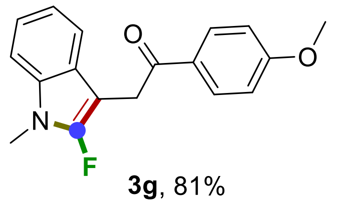

**3g**, 81%

The reaction was performed following the general procedure. The residue was purified by flash column chromatography (silica gel, petroleum ether: $CH_2Cl_2$ =10:1, v/v) to give the product as a yellow oil (48.1 mg, 83%).

**$^1$H NMR** (500 MHz, Chloroform-*d*) δ 8.06 (d, *J* = 8.9 Hz, 2H), 7.51 (d, *J* = 7.7 Hz, 1H), 7.19 (d, *J* = 3.7 Hz, 2H), 7.15 – 7.12 (m, 1H), 6.93 (d, *J* = 8.9 Hz, 2H), 4.26 (s, 2H), 3.86 (s, 3H), 3.63 (s, 3H).

**$^{13}$C NMR** (126 MHz, Chloroform-*d*) δ 195.3, 163.5, 149.9 (d, *J* = 265.9 Hz), 130.8, 130.6, 129.6, 129.5, 128.9, 125.9 (d, *J* = 30.7 Hz), 125.5 (d, *J* = 5.9 Hz), 121.2 (d, *J* = 3.5 Hz), 120.4, 118.93 (d, *J* = 6.3 Hz), 113.8, 108.8 (d, *J* = 1.7 Hz), 84.4 (d, *J* = 10.8 Hz), 55.5, 32.6 (d, *J* = 3.0 Hz), 28.0 (d, *J* = 2.1 Hz).

**$^{19}$F NMR** (471 MHz, Chloroform-*d*) δ -139.09.

**HRMS** (ESI, m/z) calcd for $C_{18}H_{16}FNO_2$ $[M+H]^+$: 298.1238; found: 298.1235.

## 1-(3,4-dimethylphenyl)-2-(2-fluoro-1-methyl-1H-indol-3-yl)ethan-1-one (3h)

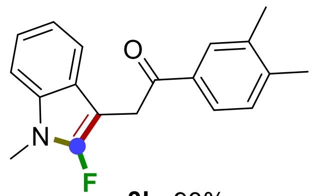


The reaction was performed following the general procedure. The residue was purified by flash column chromatography (silica gel, petroleum ether: $CH_2Cl_2$ =10:1, v/v) to give the product as a yellow solid (54.9 mg, 93%). m.p. 134-136 ºC.

**$^1$H NMR** (500 MHz, Chloroform-*d*) δ 7.91 (d, *J* = 1.8 Hz, 1H), 7.87 (m, *J* = 7.9, 2.0 Hz, 1H), 7.55 (d, *J* = 7.8 Hz, 1H), 7.25 (d, *J* = 7.9 Hz, 1H), 7.24 – 7.21 (m, 2H), 7.18 (m, *J* = 8.1, 5.9, 2.4 Hz, 1H), 4.33 (s, 2H), 3.64 (s, 3H), 2.35 (d, *J* = 2.8 Hz, 6H).

**$^{13}$C NMR** (126 MHz, Chloroform-*d*) δ 196.6, 150.0 (d, *J* = 265.6 Hz), 142.7, 137.0, 134.3, 130.6, 129.8 (d, *J* = 18.2 Hz), 126.3, 125.5 (d, *J* = 5.9 Hz), 121.2 (d, *J* = 3.4 Hz), 120.4, 118.9 (d, *J* = 6.3 Hz), 108.8 (d, *J* = 1.8 Hz), 84.4 (d, *J* = 10.7 Hz), 32. 8 (d, *J* = 2.8 Hz), 27.9 (d, *J* = 2.1 Hz), 20.0, 19.8.

**$^{19}$F NMR** (471 MHz, Chloroform-*d*) δ -139.02.

**HRMS** (ESI, m/z) calcd for $C_{19}H_{18}FNO[M+H]^+$: 296.1445; found: 296.1447.

## 2-(2-fluoro-1-methyl-1H-indol-3-yl)-1-(m-tolyl)ethan-1-one (3i)

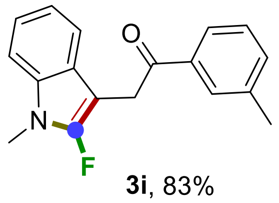


The reaction was performed following the general procedure. The residue was purified by flash column chromatography (silica gel, petroleum ether: $CH_2Cl_2$ =10:1, v/v) to give the product as a yellow solid (46.7 mg, 83%). m.p. 139-141 ºC.

**$^1$H NMR** (500 MHz, Chloroform-*d*) δ 7.84 (m, *J* = 7.7, 1.4 Hz, 1H), 7.48 (m, *J* = 7.8, 1.1 Hz, 1H), 7.37 (m, *J* = 7.4, 1.4 Hz, 1H), 7.29 (m, *J* = 7.6, 1.3 Hz, 1H), 7.24 (d, *J* = 7.7 Hz, 1H), 7.22 – 7.19 (m, 2H), 7.16 (ddd, *J* = 8.1, 5.4, 2.9 Hz, 1H), 4.27 (s, 2H), 3.62 (s, 3H), 2.49 (s, 3H).

**$^{13}$C NMR** (126 MHz, Chloroform-*d*) δ 200.7, 150.1 (d, *J* = 266.0 Hz), 138.6, 137.3, 131.9, 131.2, 130.5, 128.5, 125.5, 125.4 (d, *J* = 6.0 Hz), 121.1 (d, *J* = 3.6 Hz), 120.3, 118.6 (d, *J* = 6.4 Hz), 108.8 (d, *J* = 1.8 Hz), 84.1 (d, *J* = 11.0 Hz), 35.5 (d, *J* = 2.9 Hz), 27.8 (d, *J* = 2.0 Hz), 21.2.

**$^{19}$F NMR** (471 MHz, Chloroform-*d*) δ -139.15.

**HRMS** (ESI, m/z) calcd for $C_{18}H_{16}FNO[M+H]^+$: 282.1289; found: 282.1285.

## 2-(2-fluoro-1-methyl-1H-indol-3-yl)-1-(4-iodophenyl)ethan-1-one (3j)

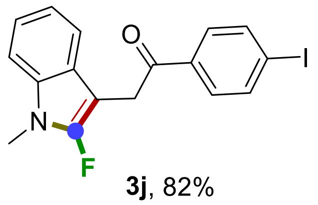

**3j**, 82%

The reaction was performed following the general procedure. The residue was purified by flash column chromatography (silica gel, petroleum ether: $CH_2Cl_2$ =10:1, v/v) to give the product as a yellow solid (66.6 mg, 82%). 149-151 °C.

**$^{1}$H NMR** (500 MHz, Chloroform-*d*) δ 7.81 (d, *J* = 8.4 Hz, 2H), 7.76 (d, *J* = 8.6 Hz, 2H), 7.50 (d, *J* = 7.7 Hz, 1H), 7.22 (q, *J* = 7.6 Hz, 2H), 7.19 – 7.14 (m, 1H), 4.26 (s, 2H), 3.62 (s, 3H).

**$^{13}$C NMR** (126 MHz, Chloroform-*d*) δ 196.0, 150.0 (d, *J* = 266.0 Hz), 137.9, 135.6, 130.6, 129.9, 125.3 (d, *J* = 5.7 Hz), 121.4 (d, *J* = 3.5 Hz), 120.6, 118.8 (d, *J* = 6.3 Hz), 109.0 (d, *J* = 1.7 Hz), 101.1, 83.8 (d, *J* = 10.8 Hz), 32.9 (d, *J* = 2.9 Hz), 28.0 (d, *J* = 1.9 Hz).

**$^{19}$F NMR** (471 MHz, Chloroform-*d*) δ -138.62.

HRMS (ESI, m/z) calcd for $C_{17}H_{13}FINO[M+H]^+$: 394.0099; found: 394.0101.

### 2-(2-fluoro-1-methyl-1H-indol-3-yl)-1-(naphthalen-2-yl)ethan-1-one (3k)

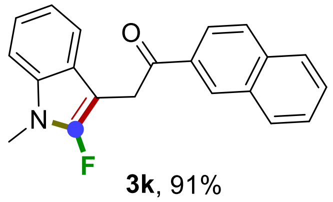

**3k**, 91%

The reaction was performed following the general procedure. The residue was purified by flash column chromatography (silica gel, petroleum ether: $CH_2Cl_2$ =10:1, v/v) to give the product as a yellow solid (57.7 mg, 91%). m.p. 153-155 °C.

**$^{1}$H NMR** (500 MHz, Chloroform-*d*) δ 8.65 (s, 1H), 8.12 (m, *J* = 8.5, 2.1 Hz, 1H), 7.98 (d, *J* = 8.0 Hz, 1H), 7.88 (m, *J* = 10.0, 8.1 Hz, 2H), 7.63 – 7.53 (m, 3H), 7.24 – 7.14 (m, 3H), 4.45 (s, 2H), 3.62 (d, *J* = 1.9 Hz, 3H).

**$^{13}$C NMR** (126 MHz, Chloroform-*d*) δ 196.7, 150.1 (d, *J* = 265.8 Hz), 135.6, 133.7, 132.6, 130.6, 130.2, 129.7, 128.5 (d, *J* = 4.1 Hz), 127.8, 126.8, 125. 5 (d, *J* = 5.7 Hz), 124.3, 121.3 (d, *J* = 3.5 Hz), 120.5, 118. 9 (d, *J* = 6.3 Hz), 108.9, 84.2 (d, *J* = 10.6 Hz), 33.0 (d, *J* = 2.8 Hz), 28.0 (d, *J* = 2.0 Hz).

**$^{19}$F NMR** (471 MHz, Chloroform-*d*) δ -138.77.

**HRMS** (ESI, m/z) calcd for $C_{21}H_{16}FNO[M+NA]^+$: 340.1108; found: 340.1111.

### 2-(2-fluoro-1-methyl-1H-indol-3-yl)-1-(furan-2-yl)ethan-1-one (3l)

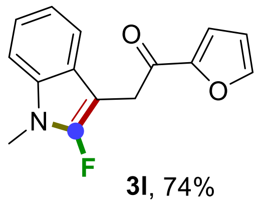

**3l**, 74%

The reaction was performed following the general procedure. The residue was purified by flash column chromatography (silica gel, petroleum ether: $CH_2Cl_2$ =10:1, v/v) to give the product as a yellow solid (38.0 mg, 74%). 133-135 ºC.

**$^{1}$H NMR** (500 MHz, Chloroform-*d*) δ 7.60 (s, 1H), 7.53 (dt, *J* = 7.9, 1.1 Hz, 1H), 7.28 – 7.26 (m, 1H), 7.20 (d, *J* = 3.6 Hz, 2H), 7.14 (m, *J* = 8.1, 4.8, 3.4 Hz, 1H), 6.53 (m, *J* = 3.6, 1.7 Hz, 1H), 4.17 (s, 2H), 3.64 (s, 3H).

**$^{13}$C NMR** (126 MHz, Chloroform-*d*) δ 185.7, 152.2, 150.2 (d, *J* = 266.3 Hz), 146.5, 130.5, 125.4 (d, *J* = 5.6 Hz), 121.3 (d, *J* = 3.4 Hz), 120.4, 118.9 (d, *J* = 6.4 Hz), 117.5, 112.3, 108.8 (d, *J* = 1.8 Hz), 83.7 (d, *J* = 11.0 Hz), 32.7 (d, *J* = 2.8 Hz), 28.0 (d, *J* = 2.1 Hz).

**$^{19}$F NMR** (471 MHz, Chloroform-*d*) δ -139.01.

**HRMS** (ESI, m/z) calcd for $C_{15}H_{12}FNO_2[M+H]^+$: 258.0925; found: 258.0932.

## 2-(2-fluoro-1-methyl-1H-indol-3-yl)-1-(thiophen-3-yl)ethan-1-one (3m)

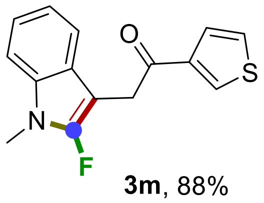

**3m**, 88%

The reaction was performed following the general procedure. The residue was purified by flash column chromatography (silica gel, petroleum ether: $CH_2Cl_2$ =10:1, v/v) to give the product as a yellow solid (48.1 mg, 88%). m.p. 139-141 ºC.

**$^{1}$H NMR** (500 MHz, Chloroform-*d*) δ 8.18 (m, *J* = 2.9, 1.3 Hz, 1H), 7.61 (m, *J* = 5.1, 1.3 Hz, 1H), 7.54 (m, *J* = 7.8, 1.1 Hz, 1H), 7.30 (m, *J* = 5.1, 2.9 Hz, 1H), 7.23 – 7.19 (m, 2H), 7.16 (m, *J* = 8.2, 5.2, 3.1 Hz, 1H), 4.21 (s, 2H), 3.63 (s, 3H).

**$^{13}$C NMR** (126 MHz, Chloroform-*d*) δ 190.9, 149.9 (d, *J* = 265.5 Hz), 141.3, 132.3, 130.5, 127.3, 126.2, 125.3 (d, *J* = 5.8 Hz), 121.2 (d, *J* = 3.5 Hz), 120.4, 118.8 (d, *J* = 6.3 Hz), 108.8 (d, *J* = 1.9 Hz), 84.0 (d, *J* = 10.7 Hz), 34.1 (d, *J* = 2.8 Hz), 27.9 (d, *J* = 2.0 Hz).

**$^{19}$F NMR** (471 MHz, Chloroform-*d*) δ -139.04.

**HRMS** (ESI, m/z) calcd for $C_{15}H_{12}FNOS[M+H]^+$: 274.0696; found: 274.0697.

## 1,1'-(1,3-phenylene)bis(2-(2-fluoro-1-methyl-1H-indol-3-yl)ethan-1-one) (3n)

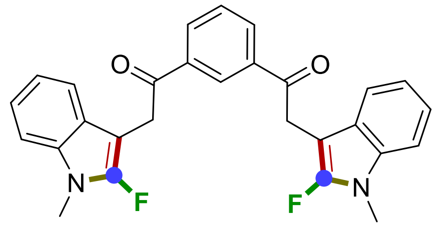


**3n**, 79%

The reaction was performed following the general procedure. The residue was purified by flash column chromatography (silica gel, petroleum ether: $CH_2Cl_2$ =10:1, v/v) to give the product as a yellow solid (72.1 mg, 79%). m.p. 179-181 °C.

**$^1$H NMR** (500 MHz, Chloroform-*d*) δ 8.80 (d, *J* = 2.0 Hz, 1H), 8.24 (m, *J* = 7.8, 1.8 Hz, 2H), 7.56 (t, *J* = 7.7 Hz, 1H), 7.51 (d, *J* = 7.7 Hz, 2H), 7.23 – 7.15 (m, 6H), 4.35 (s, 4H), 3.61 (s, 6H).

**$^{13}$C NMR** (126 MHz, Chloroform-*d*) δ 195.9, 150.0 (d, *J* = 266.1 Hz), 136.6, 132.7, 130.6, 129.2, 128.5, 125.3 (d, *J* = 5.7 Hz), 121.4 (d, *J* = 3.6 Hz), 120.5, 118.8 (d, *J* = 6.3 Hz), 108.9, 83.6 (d, *J* = 10.6 Hz), 33.0 (d, *J* = 2.7 Hz), 28.0 (d, *J* = 2.0 Hz).

**$^{19}$F NMR** (471 MHz, Chloroform-*d*) δ -138.51.

**HRMS** (ESI, m/z) calcd for $C_{28}H_{22}F_2N_2O_2[M+H]^+$: 457.1722; found: 457.1721.

## 2-(2-fluoro-1-methyl-1H-indol-3-yl)-2,3-dihydro-1H-inden-1-one (3o)

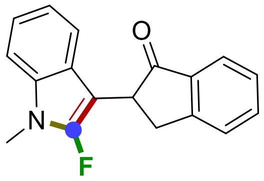


**3o**, 75%

The reaction was performed following the general procedure. The residue was purified by flash column chromatography (silica gel, petroleum ether: $CH_2Cl_2$ =10:1, v/v) to give the product as a yellow oil (41.9 mg, 75%).

**$^1$H NMR** (500 MHz, Chloroform-*d*) δ 7.89 (d, *J* = 7.7 Hz, 1H), 7.68 (m, *J* = 7.5, 1.3 Hz, 1H), 7.55 (d, *J* = 7.7 Hz, 1H), 7.46 (t, *J* = 7.5 Hz, 1H), 7.22 (d, *J* = 8.2 Hz, 1H), 7.17 (m, *J* = 8.2, 5.9, 2.3 Hz, 1H), 7.04 – 6.97 (m, 2H), 4.16 (m, *J* = 8.6, 4.9 Hz, 1H), 3.71 – 3.66 (m, 1H), 3.64 (s, 3H), 3.38 (m, *J* = 17.3, 4.8 Hz, 1H).

**$^{13}$C NMR** (126 MHz, Chloroform-*d*) δ 206.1, 153.5, 150.4 (d, *J* = 266.2 Hz), 136.4, 135.1, 130.7, 127.7, 126.6, 124.4, 124.1 (d, *J* = 5.8 Hz), 121.2 (d, *J* = 3.4 Hz), 120.3, 118.6 (d, *J* = 6.3 Hz), 109.0, 88.7 (d, *J* = 10.3 Hz), 42.8 (d, *J* = 3.1 Hz), 34.2, 27.9 (d, *J* = 2.2 Hz).

**$^{19}$F NMR** (471 MHz, Chloroform-*d*) δ -139.05.

**HRMS** (ESI, m/z) calcd for $C_{18}H_{14}FNO[M+H]^+$: 280.1132; found: 280.1129.

## 2-(2-fluoro-1-methyl-1H-indol-3-yl)-3,4-dihydronaphthalen-1(2H)-one (3p)

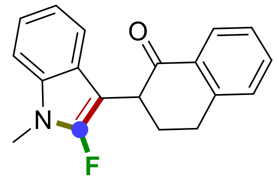


**3p**, 79%

The reaction was performed following the general procedure. The residue was purified by flash column chromatography (silica gel, petroleum ether: $CH_2Cl_2$ =10:1, v/v) to give the product as a yellow oil (46.3 mg, 79%).

**$^1$H NMR** (500 MHz, Chloroform-*d*) δ 8.16 (m, *J* = 7.8, 1.4 Hz, 1H), 7.54 (m, *J* = 7.5, 1.5 Hz, 1H), 7.40 – 7.35 (m, 1H), 7.33 (m, *J* = 7.8, 2.4, 1.4 Hz, 2H), 7.25 – 7.18 (m, 2H), 7.10 (m, *J* = 8.1, 6.9, 1.3 Hz, 1H), 4.08 (m, *J* = 12.6, 4.6 Hz, 1H), 3.65 (s, 3H), 3.22 (m, *J* = 16.4, 11.8, 4.4 Hz, 1H), 3.10 (m, *J* = 16.6, 4.0 Hz, 1H), 2.69 – 2.59 (m, 1H), 2.41 (m, *J* = 13.2, 4.3 Hz, 1H).

**$^{13}$C NMR** (126 MHz, Chloroform-*d*) δ 197.2, 149.8 (d, *J* = 266.0 Hz), 144.2, 133.4, 132.8, 130.7, 128.8, 128.0, 126.8, 124.7 (d, *J* = 6.1 Hz), 121.0 (d, *J* = 3.5 Hz), 120.2, 119.0 (d, *J* = 6.3 Hz), 109.0 (d, *J* = 1.8 Hz), 89.5 (d, *J* = 9.8 Hz), 44.4 (d, *J* = 3.3 Hz), 30.5, 29.6, 27.9 (d, *J* = 2.2 Hz).

**$^{19}$F NMR** (471 MHz, Chloroform-*d*) δ -137.74.

**HRMS** (ESI, m/z) calcd for $C_{19}H_{16}FNO[M+H]^+$: 294.1289; found: 294.1285.

## 3-(2-fluoro-1-methyl-1H-indol-3-yl)chroman-4-one (3q)

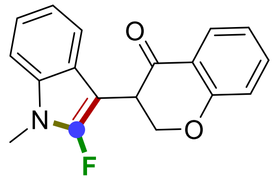


**3q**, 85%

The reaction was performed following the general procedure. The residue was purified by flash column chromatography (silica gel, petroleum ether: $CH_2Cl_2$ =10:1, v/v) to give the product as a yellow solid (50.2 mg, 85%). m.p. 139-141 °C.

**$^1$H NMR** (500 MHz, Chloroform-*d*) δ 8.03 (m, *J* = 7.9, 1.8 Hz, 1H), 7.54 (m, *J* = 8.7, 7.2, 1.8 Hz, 1H), 7.38 (d, *J* = 7.8 Hz, 1H), 7.26 – 7.20 (m, 2H), 7.17 – 7.11 (m, 1H), 7.10 – 7.03 (m, 2H), 4.77 – 4.69 (m, 1H), 4.62 (m, *J* = 11.3, 5.7 Hz, 1H), 4.41 (m, *J* = 12.8, 5.6 Hz, 1H), 3.66 (s, 3H).

**$^{13}$C NMR** (126 MHz, Chloroform-*d*) δ 191.5, 161.9, 150.4 (d, *J* = 267.3 Hz), 136.0, 130.7, 127.9, 124.4 (d, *J* = 6.2 Hz), 121.6, 121.4 (d, *J* = 3.4 Hz), 121.2, 120.6, 118.8 (d, *J* = 6.0 Hz), 117.9, 109.2, 83.8 (d, *J* = 10.1 Hz), 70.5 (d, *J* = 1.7 Hz), 42.8 (d, *J* = 3.5 Hz), 28.0 (d, *J* = 2.0 Hz).

**$^{19}$F NMR** (471 MHz, Chloroform-*d*) δ -135.79.

**HRMS** (ESI, m/z) calcd for $C_{18}H_{14}FNO_2[M+H]^+$: 296.1081; found: 296.1083.

## 6-(2-fluoro-1-methyl-1H-indol-3-yl)-6,7,8,9-tetrahydro-5H-benzo[7]annulen-5-one (3r)

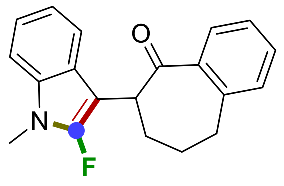

**3r**, 58%

The reaction was performed following the general procedure. The residue was purified by flash column chromatography (silica gel, petroleum ether: $CH_2Cl_2$ =10:1, v/v) to give the product as a yellow oil (35.6 mg, 88%).

**$^{1}$H NMR** (500 MHz, Chloroform-*d*) δ 7.73 – 7.66 (m, 1H), 7.45 (m, *J* = 7.5, 1.5 Hz, 1H), 7.37 (m, *J* = 7.9, 0.9 Hz, 1H), 7.33 (m, *J* = 7.5, 1.3 Hz, 1H), 7.28 (d, *J* = 7.6 Hz, 1H), 7.23 – 7.17 (m, 2H), 7.09 (m, *J* = 8.1, 6.8, 1.4 Hz, 1H), 4.29 (m, *J* = 12.0, 4.5 Hz, 1H), 3.65 (s, 3H), 3.17 (m, *J* = 15.4, 11.4, 3.9 Hz, 1H), 3.05 (m, *J* = 15.6, 5.5, 3.6 Hz, 1H), 2.50 – 2.40 (m, 1H), 2.24 – 2.10 (m, 2H), 1.92 – 1.83 (m, 1H).

**$^{13}$C NMR** (126 MHz, Chloroform-*d*) δ 205.3, 149.9 (d, *J* = 266.1 Hz), 141.4, 139.8, 131.7, 130.5, 129.7, 128.7, 126.7, 124.8 (d, *J* = 6.3 Hz), 121.0 (d, *J* = 3.5 Hz), 120.1, 119.3 (d, *J* = 6.3 Hz), 108.9 (d, *J* = 1.8 Hz), 90.3 (d, *J* = 9.2 Hz), 45.6 (d, *J* = 3.5 Hz), 33.0, 29.3 (d, *J* = 1.7 Hz), 27.9 (d, *J* = 2.3 Hz), 25.7.

**$^{19}$F NMR** (471 MHz, Chloroform-*d*) δ -137.22.

**HRMS** (ESI, m/z) calcd for $C_{20}H_{18}FNO[M+H]^+$: 308.1445; found:308.1446 .

## 1-(2-fluoro-1-methyl-1H-indol-3-yl)propan-2-one (3s)

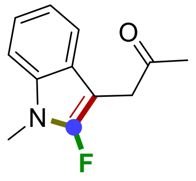

**3s**, 80%

The reaction was performed following the general procedure. The residue was purified by flash column chromatography (silica gel, petroleum ether: $CH_2Cl_2$ =10:1, v/v) to give the product as a yellow oil (32.8 mg, 80%).

**$^{1}$H NMR** (500 MHz, Chloroform-*d*) δ 7.42 (m, *J* = 7.8, 1.0 Hz, 1H), 7.24 – 7.20 (m, 2H), 7.16 (m, *J* = 8.1, 5.5, 2.8 Hz, 1H), 3.72 (s, 2H), 3.66 (s, 3H), 2.18 (s, 3H).

**$^{13}$C NMR** (126 MHz, Chloroform-*d*) δ 206.3, 150.2 (d, *J* = 266.2 Hz), 130.6, 125.2 (d, *J* = 5.8 Hz), 121.4 (d, *J* = 3.6 Hz), 120.5, 118.5 (d, *J* = 6.3 Hz), 108.9 (d, *J* = 1.8 Hz), 84.0 (d, *J* = 11.4 Hz), 37.7 (d, *J* = 2.7 Hz), 28.7, 28.0 (d, *J* = 1.9 Hz).

**$^{19}$F NMR** (471 MHz, Chloroform-*d*) δ -139.61.

**HRMS** (ESI, m/z) calcd for $C_{12}H_{12}FNO[M+H]^+$: 206.0976; found: 206.0977.

## 1-(2-fluoro-1-methyl-1H-indol-3-yl)-3-methylbutan-2-one (3t)

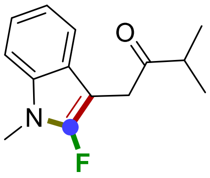

**3t**, 80%

The reaction was performed following the general procedure. The residue was purified by flash column chromatography (silica gel, petroleum ether: $CH_2Cl_2$ =10:1, v/v) to give the product as a yellow oil (37.3 mg, 80%).

**$^1$H NMR** (500 MHz, Chloroform-*d*) δ 7.42 (m, *J* = 7.8, 1.0 Hz, 1H), 7.24 – 7.21 (m, 2H), 7.15 (m, *J* = 8.1, 4.9, 3.4 Hz, 1H), 3.79 (s, 2H), 3.65 (s, 3H), 2.80 (p, *J* = 6.9 Hz, 1H), 1.14 (d, *J* = 6.8 Hz, 6H).

**$^{13}$C NMR** (126 MHz, Chloroform-*d*) δ 211.8, 150.2 (d, *J* = 266.0 Hz), 130.6, 125.4 (d, *J* = 5.9 Hz), 121.3 (d, *J* = 3.6 Hz), 120.4, 118.6 (d, *J* = 6.4 Hz), 108.9 (d, *J* = 1.8 Hz), 84.2 (d, *J* = 11.1 Hz), 39.4, 34.6 (d, *J* = 2.9 Hz), 27.9 (d, *J* = 2.1 Hz), 18.5.

**$^{19}$F NMR** (471 MHz, Chloroform-*d*) δ -139.64.

**HRMS** (ESI, m/z) calcd for $C_{14}H_{16}FNO[M+H]^+$: 234.1289; found: 234.1285.

## 1-(2-fluoro-1-methyl-1H-indol-3-yl)-3,3-dimethylbutan-2-one (3u)

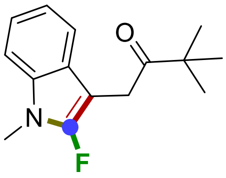

**3u**, 78%

The reaction was performed following the general procedure. The residue was purified by flash column chromatography (silica gel, petroleum ether: $CH_2Cl_2$ =10:1, v/v) to give the product as a yellow oil (38.6 mg, 78%).

**$^1$H NMR** (500 MHz, Chloroform-*d*) δ 7.41 (d, *J* = 7.8 Hz, 1H), 7.23 – 7.18 (m, 2H), 7.14 (m *J* = 8.1, 5.5, 2.7 Hz, 1H), 3.86 (s, 2H), 3.63 (s, 3H), 1.28 (s, 9H).

**$^{13}$C NMR** (126 MHz, Chloroform-*d*) δ 212.3, 150.2 (d, *J* = 265.4 Hz), 130.6, 125.6 (d, *J* = 6.0 Hz), 121.1 (d, *J* = 3.5 Hz), 120.2, 118.7 (d, *J* = 6.5 Hz), 108.8 (d, *J* = 1.8 Hz), 84.5 (d, *J* = 10.9 Hz), 44.5, 30.5 (d, *J* = 3.0 Hz), 27.9 (d, *J* = 2.1 Hz), 26.6.

**$^{19}$F NMR** (471 MHz, Chloroform-*d*) δ -139.58.

**HRMS** (ESI, m/z) calcd for $C_{15}H_{18}FNO[M+H]^+$: 248.1445; found: 248.1448.

## 2-(2-fluoro-1-methyl-1H-indol-3-yl)pentan-3-one (3v)

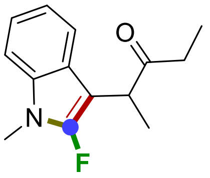

**3v**, 85%

The reaction was performed following the general procedure. The residue was purified by flash column chromatography (silica gel, petroleum ether: $CH_2Cl_2$ =10:1, v/v) to give the product as a yellow oil (39.6 mg, 85%).

**$^1$H NMR** (500 MHz, Chloroform-*d*) δ 7.47 (d, *J* = 8.0 Hz, 1H), 7.24 – 7.19 (m, 2H), 7.16 – 7.11 (m, 1H), 3.96 (q, *J* = 7.1 Hz, 1H), 3.66 (s, 3H), 2.52 – 2.35 (m, 2H), 1.50 (d, *J* = 7.0 Hz, 3H), 0.98 (t, *J* = 7.3 Hz, 3H).

**$^{13}$C NMR** (126 MHz, Chloroform-*d*) δ 211.3, 149.6 (d, *J* = 266.5 Hz), 130.5, 124.3 (d, *J* = 5.9 Hz), 121.3 (d, *J* = 3.4 Hz), 120. 5, 119.0 (d, *J* = 6.4 Hz), 108.9 (d, *J* = 1.9 Hz), 90.2 (d, *J* = 10.0 Hz), 41.5 (d, *J* = 2.9 Hz), 33.49, 27.9 (d, *J* = 2.2 Hz), 15.4, 8.1.

**$^{19}$F NMR** (471 MHz, Chloroform-*d*) δ -138.87.

**HRMS** (ESI, m/z) calcd for $C_{14}H_{16}FNO[M+H]^+$: 234.1289; found: 234.1290.

## 2-(2-fluoro-1-methyl-1H-indol-3-yl)-4-methylpentan-3-one (3w)

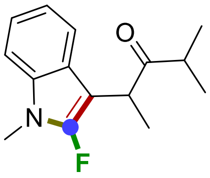

**3w**, 71%

The reaction was performed following the general procedure. The residue was purified by flash column chromatography (silica gel, petroleum ether: $CH_2Cl_2$ =10:1, v/v) to give the product as a yellow oil (35.1 mg, 71%).

**$^1$H NMR** (500 MHz, Chloroform-*d*) δ 7.51 – 7.47 (m, 1H), 7.24 – 7.18 (m, 2H), 7.13 (m, *J* = 8.1, 6.0, 2.1 Hz, 1H), 4.13 (q, *J* = 7.0 Hz, 1H), 3.65 (s, 3H), 2.77 (p, *J* = 6.9 Hz, 1H), 1.48 (d, *J* = 7.0 Hz, 3H), 1.10 (d, *J* = 7.0 Hz, 3H), 0.91 (d, *J* = 6.7 Hz, 3H).

**$^{13}$C NMR** (126 MHz, Chloroform-*d*) δ 214.3, 149.6 (d, *J* = 266.5 Hz), 130.5, 124.3 (d, *J* = 5.7 Hz), 121.2 (d, *J* = 3.4 Hz), 120.44, 119.2 (d, *J* = 6.4 Hz), 108.9 (d, *J* = 1.9 Hz), 90.0 (d, *J* = 10.1 Hz), 39.7 (d, *J* = 2.8 Hz), 38.2, 27.9 (d, *J* = 2.1 Hz), 19.5, 18.2, 15.7.

**$^{19}$F NMR** (471 MHz, Chloroform-*d*) δ -139.01.

**HRMS** (ESI, m/z) calcd for $C_{15}H_{18}FNO[M+H]^+$: 248.1145; found: 248.1448.

## 1-(2-fluoro-1-methyl-1H-indol-3-yl)nonan-2-one (3x)

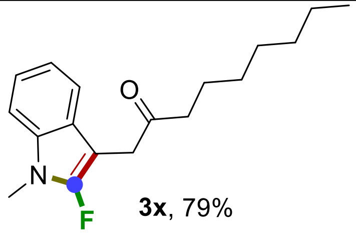

**3x**, 79%

The reaction was performed following the general procedure. The residue was purified by flash column chromatography (silica gel, petroleum ether: $CH_2Cl_2$ =10:1, v/v) to give the product as a yellow oil (45.7 mg, 79%).

**$^1$H NMR** (500 MHz, Chloroform-*d*) δ 7.42 (d, *J* = 7.8 Hz, 1H), 7.24 – 7.19 (m, 2H), 7.15 (m, *J* = 8.1, 5.4, 2.8 Hz, 1H), 3.70 (s, 2H), 3.65 (s, 3H), 2.47 (t, *J* = 7.4 Hz, 2H), 1.57 (p, *J* = 7.3 Hz, 2H), 1.23 (s, 8H), 0.87 (t, *J* = 6.9 Hz, 3H).

**$^{13}$C NMR** (126 MHz, Chloroform-*d*) δ 208.5, 150.2 (d, *J* = 266.0 Hz), 130.6, 125.3 (d, *J* = 5.9 Hz), 121.3 (d, *J* = 3.6 Hz), 120.5, 118.5 (d, *J* = 6.4 Hz), 108.8 (d, *J* = 1.8 Hz), 84.2 (d, *J* = 11.1 Hz), 41.3, 36.9 (d, *J* = 2.8 Hz), 31.7, 29.1 (d, *J* = 8.5 Hz), 28.0 (d, *J* = 2.0 Hz), 23.9, 22.6, 14.1.

**$^{19}$F NMR** (471 MHz, Chloroform-*d*) δ -139.70.

**HRMS** (ESI, m/z) calcd for $C_{18}H_{24}FNO[M+NA]^+$: 312.1734; found: 312.1739.

## cyclopropyl-2-(2-fluoro-1-methyl-1H-indol-3-yl)ethan-1-one (3y)

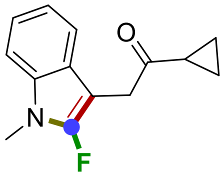

**3y**, 73%

The reaction was performed following the general procedure. The residue was purified by flash column chromatography (silica gel, petroleum ether: $CH_2Cl_2$ =10:1, v/v) to give the product as a yellow solid (33.7 mg, 73%). m.p. 155-157 °C.

**$^1$H NMR** (500 MHz, Chloroform-*d*) δ 7.45 (d, *J* = 7.8 Hz, 1H), 7.25 – 7.22 (m, 2H), 7.16 (m, *J* = 8.1, 5.3, 3.0 Hz, 1H), 3.85 (s, 2H), 3.66 (s, 3H), 2.06 (m, *J* = 7.8, 4.5 Hz, 1H), 1.09 – 1.02 (m, 2H), 0.84 (m, *J* = 7.2, 3.6 Hz, 2H).

**$^{13}$C NMR** (126 MHz, Chloroform-*d*) δ 208.1, 150.3 (d, *J* = 266.2 Hz), 130.6, 125.5(d, *J* = 5.9 Hz), 121.3 (d, *J* = 3.6 Hz), 120.4, 118.6 (d, *J* = 6.4 Hz), 108.8 (d, *J* = 1.8 Hz), 84.1 (d, *J* = 11.2 Hz), 37.4 (d, *J* = 2.7 Hz), 28.0 (d, *J* = 2.0 Hz), 19.4, 11.3.

**$^{19}$F NMR** (471 MHz, Chloroform-*d*) δ -139.61.

**HRMS** (ESI, m/z) calcd for $C_{14}H_{14}FNO[M+H]^+$: 232.1132; found: 232.1135.

## 1-cyclohexyl-2-(2-fluoro-1-methyl-1H-indol-3-yl)ethan-1-one (3z)

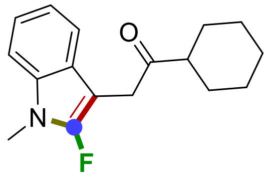


**3z**, 79%

The reaction was performed following the general procedure. The residue was purified by flash column chromatography (silica gel, petroleum ether: $CH_2Cl_2$ =10:1, v/v) to give the product as a yellow oil (43.2mg, 79%).

**$^{1}$H NMR** (500 MHz, Chloroform-*d*) δ 7.44 – 7.40 (m, 1H), 7.25 – 7.20 (m, 2H), 7.16 (m, *J* = 8.1, 5.7, 2.6 Hz, 1H), 3.78 (s, 2H), 3.67 (s, 3H), 2.55 (m, *J* = 11.5, 3.5 Hz, 1H), 1.93 – 1.87 (m, 2H), 1.83 – 1.78 (m, 2H), 1.47 – 1.39 (m, 2H), 1.34 – 1.20 (m, 4H).

**$^{13}$C NMR** (126 MHz, Chloroform-*d*) δ 210.9, 150.2 (d, *J* = 265.9 Hz), 130.6, 125.5 (d, *J* = 6.0 Hz), 121.2 (d, *J* = 3.6 Hz), 120.4, 118.6 (d, *J* = 6.5 Hz), 108.8 (d, *J* = 1.8 Hz), 84.1 (d, *J* = 11.2 Hz), 49.5, 34.7 (d, *J* = 2.9 Hz), 28.7, 28.0 (d, *J* = 2.0 Hz), 25.9, 25.7.

**$^{19}$F NMR** (471 MHz, Chloroform-*d*) δ -139.67.

**HRMS** (ESI, m/z) calcd for $C_{17}H_{20}FNO[M+H]^+$: 274.1602; found: 274.1600.

## 1-((1S,4S)-bicyclo[2.2.1]hept-5-en-2-yl)-2-(2-fluoro-1-methyl-1H-indol-3-yl)ethan-1-one (3aa)

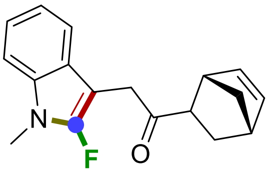


**3aa**, 65%

The reaction was performed following the general procedure. The residue was purified by flash column chromatography (silica gel, petroleum ether: $CH_2Cl_2$ =10:1, v/v) to give the product as a yellow oil (36.8 mg, 65%).

**$^{1}$H NMR** (500 MHz, Chloroform-*d*) δ 7.39 (d, *J* = 7.7 Hz, 1H), 7.21 (m, *J* = 3.9, 1.4 Hz, 2H), 7.14 (m, *J* = 8.1, 5.3, 3.1 Hz, 1H), 3.82 (s, 2H), 3.63 (s, 3H), 2.12 (s, 3H), 1.98 (s, 6H), 1.83 – 1.73 (m, 6H).

**$^{13}$C NMR** (126 MHz, Chloroform-*d*) δ 210.0, 150.3 (d, *J* = 266.0 Hz), 138.2, 137.9, 136.0, 131.3, 130.5, 125.5 (d, *J* = 5.9 Hz), 121.2 (d, *J* = 3.6 Hz), 120.4, 118.6 (d, *J* = 6.4 Hz), 108.9 (d, *J* = 1.8 Hz), 84.3 (d, *J* = 11.2 Hz), 52.4, 50.0, 49.4, 46.0, 45.8, 42.7, 41.8, 36.6 (d, *J* = 2.8 Hz), 29.7, 29.2, 28.0 (d, *J* = 2.0 Hz), 27.5.

**$^{19}$F NMR** (471 MHz, Chloroform-*d*) δ -139.58.

**HRMS** (ESI, m/z) calcd for $C_{18}H_{18}FNO[M+H]^+$: 284.1445; found: 284.1446.

## 2-(2-fluoro-1-methyl-1H-indol-3-yl)cyclobutan-1-one (3ab)

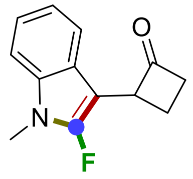

**3ab**, 52%

The reaction was performed following the general procedure. The residue was purified by flash column chromatography (silica gel, petroleum ether: $CH_2Cl_2$ =10:1, v/v) to give the product as a yellow oil (22.6 mg, 52%).

**$^{1}$H NMR** (500 MHz, Chloroform-*d*) δ 7.39 (d, *J* = 7.8 Hz, 1H), 7.23 – 7.17 (m, 2H), 7.13 (m, *J* = 8.1, 5.0, 3.3 Hz, 1H), 4.68 (m, *J* = 10.7, 8.5, 2.3 Hz, 1H), 3.62 (s, 3H), 3.27 (m, *J* = 17.7, 10.6, 8.6, 2.1 Hz, 1H), 3.17 (m, *J* = 17.7, 9.6, 4.8, 2.5 Hz, 1H), 2.54 (m, *J* = 10.9, 4.9 Hz, 1H), 2.35 (m, *J* = 11.0, 8.8 Hz, 1H).

**$^{13}$C NMR** (126 MHz, Chloroform-*d*) δ 208.4, 149.6 (d, *J* = 267.0 Hz), 130.5, 124.3 (d, *J* = 6.0 Hz), 121.3 (d, *J* = 3.4 Hz), 120.4, 118.7 (d, *J* = 6.4 Hz), 109.0 (d, *J* = 1.8 Hz), 86.7 (d, *J* = 10.0 Hz), 54.4 (d, *J* = 3.8 Hz), 45.22, 27.8 (d, *J* = 2.2 Hz), 27.2, 17.9 (d, *J* = 1.7 Hz).

**$^{19}$F NMR** (471 MHz, Chloroform-*d*) δ -137.31.

**HRMS** (ESI, m/z) calcd for $C_{13}H_{12}FNO[M+H]^+$: 218.0976; found: 218.0972.

## 3-(2-fluoro-1-methyl-1H-indol-3-yl)tetrahydro-4H-pyran-4-one (3ac)

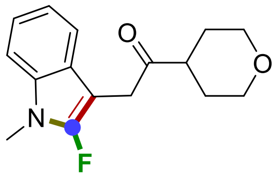

**3ac**, 75%

The reaction was performed following the general procedure. The residue was purified by flash column chromatography (silica gel, petroleum ether: $CH_2Cl_2$ =10:1, v/v) to give the product as a yellow oil (37.0 mg, 75%).

**$^{1}$H NMR** (500 MHz, Chloroform-*d*) δ 7.40 (d, *J* = 7.8 Hz, 1H), 7.24 – 7.19 (m, 2H), 7.15 (m, *J* = 8.1, 5.2, 3.1 Hz, 1H), 3.99 (m, *J* = 11.4, 3.5 Hz, 2H), 3.77 (s, 2H), 3.65 (s, 3H), 3.40 (m, *J* = 11.5, 7.8, 5.5 Hz, 2H), 2.73 (m, *J* = 15.3, 8.9, 6.9 Hz, 1H), 1.79 – 1.75 (m, 4H).

**$^{13}$C NMR** (126 MHz, Chloroform-*d*) δ 209.0, 150.2 (d, *J* = 266.0 Hz), 130.6, 125.3 (d, *J* = 5.8 Hz), 121.4 (d, *J* = 3.5 Hz), 120.5, 118.5 (d, *J* = 6.3 Hz), 108.9 (d, *J* = 1.8 Hz), 83.7 (d, *J* = 11.1 Hz), 67.2, 46.1, 34.6 (d, *J* = 2.9 Hz), 28.3, 28.0 (d, *J* = 2.1 Hz).

**$^{19}$F NMR** (471 MHz, Chloroform-*d*) δ -139.47.

**HRMS** (ESI, m/z) calcd for $C_{14}H_{14}FNO_2[M+H]^+$: 248.1081; found: 248.1082.

## 2-(2-fluoro-1-methyl-1H-indol-3-yl)cyclooctan-1-one (3ad)

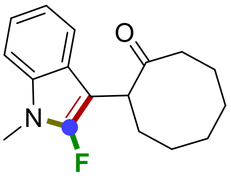

**3ad**, 75%

The reaction was performed following the general procedure. The residue was purified by flash column chromatography (silica gel, petroleum ether: $CH_2Cl_2$ =10:1, v/v) to give the product as a yellow oil (41.0 mg, 75%).

**$^1$H NMR** (500 MHz, Chloroform-*d*) δ 7.64 (m, *J* = 7.8, 1.1 Hz, 1H), 7.21 – 7.17 (m, 2H), 7.14 (m, *J* = 8.1, 5.6, 2.8 Hz, 1H), 3.90 (m, *J* = 12.2, 3.0 Hz, 1H), 3.62 (s, 3H), 2.76 (m, *J* = 12.5, 8.3, 5.9 Hz, 1H), 2.70 – 2.60 (m, 1H), 2.35 – 2.25 (m, 1H), 2.14 – 2.06 (m, 1H), 1.96 – 1.89 (m, 2H), 1.81 – 1.73 (m, 2H), 1.64 (m, *J* = 9.7, 3.8, 2.1 Hz, 2H), 1.56 – 1.49 (m, 1H), 1.43 (m, *J* = 11.4, 9.8, 3.8, 2.4 Hz, 1H), 0.93 – 0.77 (m, 1H).

**$^{13}$C NMR** (126 MHz, Chloroform-*d*) δ 215.2, 145.0 (d, *J* = 267.3 Hz), 130.3, 124.9 (d, *J* = 6.5 Hz), 121.2 (d, *J* = 3.4 Hz), 120.4, 119.4 (d, *J* = 6.3 Hz), 108. 7 (d, *J* = 1.8 Hz), 88.7 (d, *J* = 8.0 Hz), 48.7 (d, *J* = 3.6 Hz), 39.4, 29.1 (d, *J* = 2.3 Hz), 27.9 (d, *J* = 2.5 Hz), 27.8, 27.1, 26.4, 24.7.

**$^{19}$F NMR** (471 MHz, Chloroform-*d*) δ -135.56.

**HRMS** (ESI, m/z) calcd for $C_{17}H_{20}FNO[M+H]^+$: 274.1602; found:274.1605.

## (1R,4S)-3-(2-fluoro-1-methyl-1H-indol-3-yl)bicyclo[2.2.1]heptan-2-one (3ae)

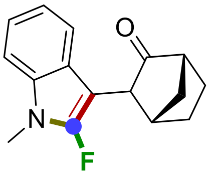

**3ae**, 65%

The reaction was performed following the general procedure. The residue was purified by flash column chromatography (silica gel, petroleum ether: $CH_2Cl_2$ =10:1, v/v) to give the product as a yellow oil (33.4 mg, 65%).

**$^1$H NMR** (500 MHz, Chloroform-*d*) δ 7.40 (d, *J* = 7.8 Hz, 1H), 7.23 – 7.19 (m, 2H), 7.14 (m, *J* = 8.1, 4.9, 3.4 Hz, 1H), 3.63 (s, 3H), 3.57 (d, *J* = 4.4 Hz, 1H), 2.85 (d, *J* = 4.8 Hz, 2H), 1.98 – 1.88 (m, 2H), 1.83 – 1.77 (m, 1H), 1.74 (m, *J* = 10.4, 1.6 Hz, 1H), 1.62 – 1.56 (m, 2H).

**$^{13}$C NMR** (126 MHz, Chloroform-*d*) δ 216.4, 150.0 (d, *J* = 267.4 Hz), 130.5, 128.6, 126.5, 125.8 (d, *J* = 6.5 Hz), 121.2 (d, *J* = 3.4 Hz), 120.3, 118.2 (d, *J* = 6.3 Hz), 108.9 (d, *J* = 1.7 Hz), 86.1 (d, *J* = 9.3 Hz), 51.0, 50.2 (d, *J* = 3.8 Hz), 41.7, 37.0, 27.9 (d, *J* = 2.3 Hz), 25.4, 22.5.

**$^{19}$F NMR** (471 MHz, Chloroform-*d*) δ -135.13.

**HRMS** (ESI, m/z) calcd for $C_{16}H_{16}FNO[M+H]^+$: 258.1289; found: 258.1291.

## 1,3-bis(2-fluoro-1-methyl-1H-indol-3-yl)propan-2-one (3af)

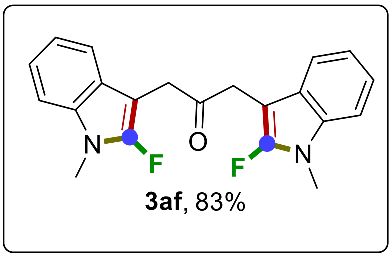

The reaction was performed following the general procedure. The residue was purified by flash column chromatography (silica gel, petroleum ether: $CH_2Cl_2$ =10:1, v/v) to give the product as a yellow solid (58.5 mg, 83%). m.p. 166-168 ºC.

**$^1$H NMR** (500 MHz, Chloroform-*d*) δ 7.34 (m, *J* = 7.8, 1.0 Hz, 2H), 7.25 – 7.20 (m, 4H), 7.14 (m, *J* = 8.2, 6.3, 1.9 Hz, 2H), 3.80 (s, 4H), 3.60 (s, 7H).

**$^{13}$C NMR** (126 MHz, Chloroform-*d*) δ 205.5, 150.2 (d, *J* = 266.3 Hz), 130.6, 125.3 (d, *J* = 5.9 Hz), 121.3 (d, *J* = 3.5 Hz), 120.4, 118.5 (d, *J* = 6.7 Hz), 108.8, 83.9 (d, *J* = 11.0 Hz), 35.6 (d, *J* = 2.1 Hz), 27.9 (d, *J* = 2.0 Hz).

**$^{19}$F NMR** (471 MHz, Chloroform-*d*) δ -139.43.

**HRMS** (ESI, m/z) calcd for $C_{21}H_{18}F_2N_2O[M+H]^+$: 353.1460; found: 353.1462.

## 2-(2-fluoro-1,5-dimethyl-1H-indol-3-yl)-1-phenylethan-1-one (3ag)

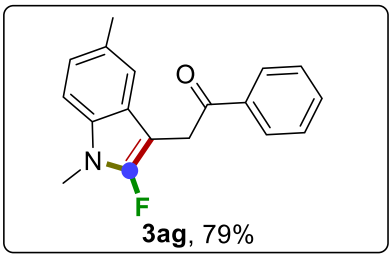

The reaction was performed following the general procedure. The residue was purified by flash column chromatography (silica gel, petroleum ether: $CH_2Cl_2$ =10:1, v/v) to give the product as a yellow solid (44.4 mg, 79%). m.p. 146-148 ºC.

**$^1$H NMR** (500 MHz, Chloroform-*d*) δ 8.11 (d, *J* = 7.0 Hz, 2H), 7.61 – 7.55 (m, 1H), 7.49 (m, *J* = 8.4, 7.0 Hz, 2H), 7.37 – 7.29 (m, 1H), 7.10 (d, *J* = 8.2 Hz, 1H), 7.04 (m, *J* = 8.4, 1.6 Hz, 1H), 4.31 (s, 2H), 3.60 (s, 3H), 2.47 (s, 3H).

**$^{13}$C NMR** (126 MHz, Chloroform-*d*) δ 196.8, 150.1 (d, *J* = 265.5 Hz), 136.4, 133.1, 129.8, 128.9, 128.6 (d, *J* = 9.5 Hz), 125.6 (d, *J* = 5.6 Hz), 122.7 (d, *J* = 3.6 Hz), 118.7 (d, *J* = 6.0 Hz), 108.6 (d, *J* = 1.7 Hz), 83.6 (d, *J* = 10.8 Hz), 32.9 (d, *J* = 2.8 Hz), 28.0 (d, *J* = 2.0 Hz), 21.6.

**$^{19}$F NMR** (471 MHz, Chloroform-*d*) δ -138.77.

**HRMS** (ESI, m/z) calcd for $C_{18}H_{16}FNO[M+H]^+$: 282.1289; found: 282.1291.

## 2-(2-fluoro-1-methyl-5-(trifluoromethyl)-1H-indol-3-yl)-1-phenylethan-1-one (3ah)

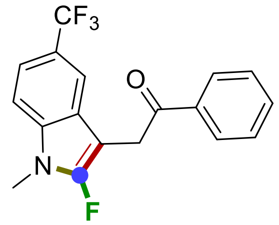

**3ah**, 39%

The reaction was performed following the general procedure. The residue was purified by flash column chromatography (silica gel, petroleum ether: $CH_2Cl_2$ =10:1, v/v) to give the product as a yellow oil (26.1 mg, 39%).

**$^1$H NMR** (500 MHz, Chloroform-*d*) δ 8.11 (d, *J* = 8.0 Hz, 2H), 7.77 (s, 1H), 7.62 (t, *J* = 7.3 Hz, 1H), 7.52 (t, *J* = 7.7 Hz, 2H), 7.46 (d, *J* = 7.9 Hz, 1H), 7.30 (d, *J* = 13.2 Hz, 2H), 4.38 (s, 2H), 3.71 (s, 3H).

**$^{13}$C NMR** (126 MHz, Chloroform-*d*) δ 196.0, 150.9 (d, *J* = 267.9 Hz), 136.3, 133.3, 132.1, 128.7, 128.4, 126.3, 125.0 (d, *J* = 6.4 Hz), 124.1, 122.9 (d, *J* = 32.0 Hz), 118.41 – 117.80 (m), 116.5 (dd, *J* = 7.0, 3.9 Hz), 109.1 (d, *J* = 2.2 Hz), 85.2 (d, *J* = 11.2 Hz), 32.4 (d, *J* = 3.0 Hz), 28.2 (d, *J* = 2.1 Hz).

**$^{19}$F NMR** (471 MHz, Chloroform-*d*) δ -60.38, -135.87.

**HRMS** (ESI, m/z) calcd for $C_{18}H_{13}F_4NO[M+H]^+$: 336.1006; found: 336.1011.

**2-(5-bromo-2-fluoro-1-methyl-1H-indol-3-yl)-1-phenylethan-1-one (3ai)**

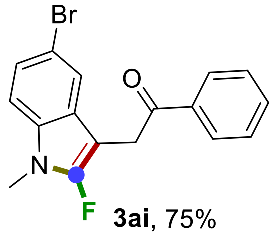

**3ai**, 75%

The reaction was performed following the general procedure. The residue was purified by flash column chromatography (silica gel, petroleum ether: $CH_2Cl_2$ =10:1, v/v) to give the product as a yellow solid (51.8 mg, 75%). m.p. 117-119 °C.

**$^1$H NMR** (500 MHz, Chloroform-*d*) δ 8.10 – 8.03 (m, 2H), 7.61 – 7.55 (m, 2H), 7.48 (m, *J* = 8.4, 7.1 Hz, 2H), 7.27 (d, *J* = 10.2 Hz, 1H), 7.06 (d, *J* = 8.6 Hz, 1H), 4.28 (s, 2H), 3.61 (s, 3H).

**$^{13}$C NMR** (126 MHz, Chloroform-*d*) δ 196.2, 150.5 (d, *J* = 267.6 Hz), 136.3, 133.3, 129.3, 128.7, 128.5, 127.1 (d, *J* = 6.0 Hz), 124.1 (d, *J* = 3.6 Hz), 121.4 (d, *J* = 6.3 Hz), 113.7, 110.5 (d, *J* = 2.1 Hz), 84.1 (d, *J* = 11.5 Hz), 32.6 (d, *J* = 3.0 Hz), 28.1.

**$^{19}$F NMR** (471 MHz, Chloroform-*d*) δ -136.49.

**HRMS** (ESI, m/z) calcd for $C_{17}H_{13}BrFNO[M+H]^+$: 346.0237; found: 346.0236.

**2-(2-fluoro-1,6-dimethyl-1H-indol-3-yl)-1-phenylethan-1-one (3aj)**

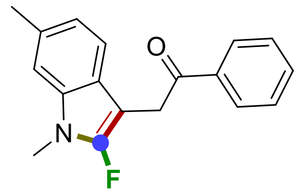

**3aj**, 75%

The reaction was performed following the general procedure. The residue was purified by flash column chromatography (silica gel, petroleum ether: $CH_2Cl_2$ =10:1, v/v) to give the product as a yellow solid (42.2 mg, 75%). m.p. 139-141 °C.

**$^{1}$H NMR** (500 MHz, Chloroform-*d*) δ 8.11 – 8.06 (m, 2H), 7.59 – 7.53 (m, 1H), 7.47 (t, *J* = 7.6 Hz, 2H), 7.40 (d, *J* = 7.9 Hz, 1H), 7.03 – 6.94 (m, 2H), 4.30 (s, 2H), 3.59 (s, 3H), 2.48 (s, 3H).

**$^{13}$C NMR** (126 MHz, Chloroform-*d*) δ 196.8 (d, *J* = 1.7 Hz), 149.6 (d, *J* = 264.9 Hz), 136.4, 133.1, 131.0 (d, *J* = 3.6 Hz), 130.9, 128.6 (d, *J* = 9.2 Hz), 123.1 (d, *J* = 5.8 Hz), 121.9, 118.6 (d, *J* = 6.3 Hz), 109.0, 83.8 (d, *J* = 10.8 Hz), 33.0 (d, *J* = 2.9 Hz), 27.9 (d, *J* = 2.1 Hz), 21.9.

**$^{19}$F NMR** (471 MHz, Chloroform-*d*) δ -139.76.

**HRMS** (ESI, m/z) calcd for $C_{18}H_{16}FNO[M+NA]^{+}$: 304.1109; found: 304.1108.

## 2-(2-fluoro-6-methoxy-1-methyl-1H-indol-3-yl)-1-phenylethan-1-one (3ak)

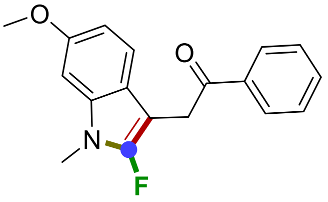

**3ak**, 81%

The reaction was performed following the general procedure. The residue was purified by flash column chromatography (silica gel, petroleum ether: $CH_2Cl_2$ =10:1, v/v) to give the product as a yellow oil (48.1mg, 81%).

**$^{1}$H NMR** (500 MHz, Chloroform-*d*) δ 8.09 – 8.05 (m, 2H), 7.58 – 7.52 (m, 1H), 7.46 (m, *J* = 8.4, 7.0 Hz, 2H), 7.38 (d, *J* = 8.6 Hz, 1H), 6.80 (m, *J* = 8.7, 2.3 Hz, 1H), 6.70 (d, *J* = 2.3 Hz, 1H), 4.28 (s, 2H), 3.85 (s, 3H), 3.57 (s, 3H).

**$^{13}$C NMR** (126 MHz, Chloroform-*d*) δ 196.7, 156.0 (d, *J* = 2.9 Hz), 149.2 (d, *J* = 264.1 Hz), 136.4, 133.08, 131.3, 128.6 (d, *J* = 11.8 Hz), 119.7 (d, *J* = 6.3 Hz), 119.4 (d, *J* = 5.8 Hz), 109.3, 93.7, 83.7 (d, *J* = 10.6 Hz), 55.9, 33.0 (d, *J* = 2.9 Hz), 28.0 (d, *J* = 2.1 Hz).

**$^{19}$F NMR** (471 MHz, Chloroform-*d*) δ -139.76.

**HRMS** (ESI, m/z) calcd for $C_{18}H_{16}FNO_2[M+H]^{+}$: 298.1238; found: 298.1235.

## 2-(6-bromo-2-fluoro-1-methyl-1H-indol-3-yl)-1-phenylethan-1-one (3al)

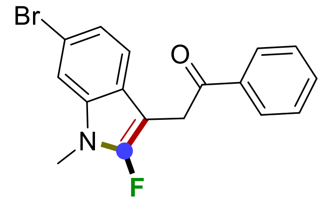

**3al**, 78%

The reaction was performed following the general procedure. The residue was purified by flash column chromatography (silica gel, petroleum ether: $CH_2Cl_2$ =10:1, v/v) to give the product as a yellow solid (53.8 mg, 78%). m.p. 157-159 °C.

**$^1$H NMR** (500 MHz, Chloroform-*d*) δ 8.09 (m, *J* = 8.3, 1.3 Hz, 2H), 7.64 – 7.57 (m, 2H), 7.51 (t, *J* = 7.7 Hz, 2H), 7.29 (m, *J* = 8.5, 2.0 Hz, 1H), 7.11 – 7.04 (m, 1H), 4.30 (s, 2H), 3.62 (s, 3H).

**$^{13}$C NMR** (126 MHz, Chloroform-*d*) δ 196.2, 150.5 (d, *J* = 267.6 Hz), 136.2, 133.3, 129.3, 128.6 (d, *J* = 31.8 Hz), 127.1 (d, *J* = 6.1 Hz), 124.1 (d, *J* = 3.5 Hz), 121.4 (d, *J* = 6.3 Hz), 113.7, 110.5 (d, *J* = 2.1 Hz), 84.1 (d, *J* = 11.2 Hz), 32.6 (d, *J* = 2.9 Hz), 28.1 (d, *J* = 1.9 Hz).

**$^{19}$F NMR** (471 MHz, Chloroform-*d*) δ -136.43.

**HRMS** (ESI, m/z) calcd for $C_{17}H_{13}BrFNO[M+H]^+$: 246.0237; found: 246.0237.

## 2-(2,7-difluoro-1-methyl-1H-indol-3-yl)-1-phenylethan-1-one (3am)

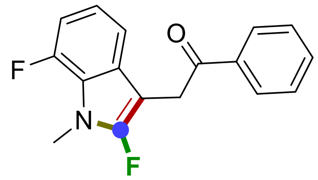

**3am**, 78%

The reaction was performed following the general procedure. The residue was purified by flash column chromatography (silica gel, petroleum ether: $CH_2Cl_2$ =10:1, v/v) to give the product as a yellow solid (44.5 mg, 78%). m.p. 144-146 °C.

**$^1$H NMR** (500 MHz, Chloroform-*d*) δ 8.15 – 8.09 (m, 2H), 7.64 – 7.59 (m, 1H), 7.52 (m, *J* = 8.4, 7.0 Hz, 2H), 7.11 (m, *J* = 8.1, 5.1 Hz, 1H), 7.03 (d, *J* = 8.1 Hz, 1H), 6.81 (m, *J* = 11.0, 7.9 Hz, 1H), 4.49 (s, 2H), 3.66 (s, 3H).

**$^{13}$C NMR** (126 MHz, Chloroform-*d*) δ 196.7, 158.1 (d, *J* = 6.5 Hz), 156.1 (d, *J* = 6.7 Hz), 150.0 (d, *J* = 266.1 Hz), 136.7, 133.1, 133.0 (d, *J* = 11.6 Hz), 121.4 (dd, *J* = 8.0, 3.3 Hz), 114.1 (dd, *J* = 19.4, 6.5 Hz), 105.9 (d, *J* = 19.4 Hz), 105.1 (dd, *J* = 6.3, 1.8 Hz), 82.6 (d, *J* = 13.1 Hz), 33.3 (t, *J* = 2.4 Hz), 28.4 (d, *J* = 1.8 Hz).

**$^{19}$F NMR** (471 MHz, Chloroform-*d*) δ -126.61 (d, *J* = 2.7 Hz), -138.92 (d, *J* = 3.5 Hz).

**HRMS** (ESI, m/z) calcd for $C_{17}H_{13}F_2NO[M+H]^+$: 286.1038; found: 286.1032.

## 2-(7-chloro-2-fluoro-1-methyl-1H-indol-3-yl)-1-phenylethan-1-one (3an)

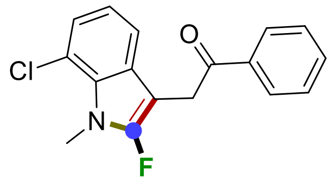

**3an**, 78%

The reaction was performed following the general procedure. The residue was purified by flash column chromatography (silica gel, petroleum ether: $CH_2Cl_2$ =10:1, v/v) to give the product as a yellow solid (47.0mg, 78%). m.p. 131-133 °C.

**$^{1}$H NMR** (500 MHz, Chloroform-*d*) δ 8.11 (d, *J* = 7.4 Hz, 2H), 7.64 – 7.57 (m, 1H), 7.51 (t, *J* = 7.7 Hz, 2H), 7.15 – 7.05 (m, 3H), 4.61 (s, 2H), 3.61 (s, 3H).

**$^{13}$C NMR** (126 MHz, Chloroform-*d*) δ 197.1, 151.2 (d, *J* = 265.9 Hz), 136.9, 133.1, 131.9, 128.5 (d, *J* = 44.1 Hz), 125.8 (d, *J* = 6.8 Hz), 122.5 (d, *J* = 6.5 Hz), 121.5 (d, *J* = 3.4 Hz), 121.3, 107.8 (d, *J* = 1.9 Hz), 84.7 (d, *J* = 12.5 Hz), 33.2 (d, *J* = 2.7 Hz), 28.2 (d, *J* = 1.9 Hz).

**$^{19}$F NMR** (471 MHz, Chloroform-*d*) δ -138.33.

**HRMS** (ESI, m/z) calcd for $C_{17}H_{13}ClFNO[M+H]^+$: 302.0742; found: 302.0736.

## 2-(1-ethyl-2-fluoro-1H-indol-3-yl)-1-phenylethan-1-one (3ao)

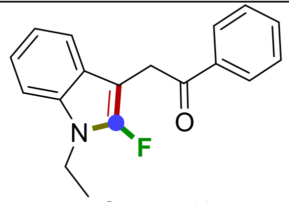

**3ao**, 31%

The reaction was performed following the general procedure. The residue was purified by flash column chromatography (silica gel, petroleum ether: $CH_2Cl_2$ =10:1, v/v) to give the product as a yellow oil (17.4 mg, 31%).

**$^{1}$H NMR** (500 MHz, Chloroform-*d*) δ 8.15 – 8.02 (m, 2H), 7.57 – 7.53 (m, 1H), 7.52 – 7.44 (m, 3H), 7.25 – 7.16 (m, 2H), 7.12 (m, *J* = 7.5, 1.2 Hz, 1H), 4.31 (s, 2H), 4.11 (q, *J* = 7.2 Hz, 2H), 1.38 (t, *J* = 7.2 Hz, 3H).

**$^{13}$C NMR** (126 MHz, Chloroform-*d*) δ 196.7, 149.7 (d, *J* = 265.5 Hz), 136.4, 133.1, 129.5, 128.6 (d, *J* = 11.9 Hz), 125.6 (d, *J* = 5.6 Hz), 121.1 (d, *J* = 3.4 Hz), 120.3, 118.9 (d, *J* = 6.3 Hz), 108.87, 84.1 (d, *J* = 10.6 Hz), 36.9, 32.9 (d, *J* = 3.0 Hz).

**$^{19}$F NMR** (471 MHz, Chloroform-*d*) δ -138.84.

**HRMS** (ESI, m/z) calcd for $C_{17}H_{14}FNO[M+H]^+$: 282.1289; found: 282.1294.

## methyl 2-(2-fluoro-1-methyl-1H-indol-3-yl)acetate (5a)

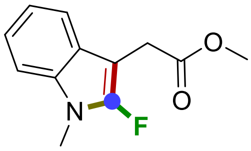

**5a**, 64%

The reaction was performed following the general procedure. The residue was purified by flash column chromatography (silica gel, petroleum ether: $CH_2Cl_2$ =10:1, v/v) to give the product as a yellow oil (28.3 mg, 64%).

**$^1$H NMR** (500 MHz, Chloroform-*d*) δ 7.52 (d, *J* = 7.7 Hz, 1H), 7.24 – 7.21 (m, 2H), 7.20 – 7.15 (m, 1H), 3.72 (d, *J* = 1.2 Hz, 5H), 3.64 (s, 3H).

**$^{13}$C NMR** (126 MHz, Chloroform-*d*) δ 171.8 (d, *J* = 2.0 Hz), 150.2 (d, *J* = 266.6 Hz), 130.5, 125.3 (d, *J* = 5.7 Hz), 121.3 (d, *J* = 3.5 Hz), 120.4, 118.6 (d, *J* = 6.4 Hz), 108.9 (d, *J* = 1.8 Hz), 83.7 (d, *J* = 10.8 Hz), 52.08, 28.1 (d, *J* = 2.5 Hz), 27.9 (d, *J* = 2.1 Hz).

**$^{19}$F NMR** (471 MHz, Chloroform-*d*) δ -139.45.

**HRMS** (ESI, m/z) calcd for $C_{12}H_{12}FNO_2[M+H]^+$: 222.0925; found: 222.0922.

## ethyl 2-(2-fluoro-1-methyl-1H-indol-3-yl)acetate (5b)

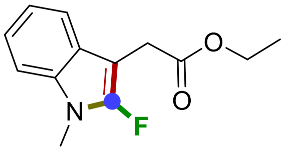

**5b**, 59%

The reaction was performed following the general procedure. The residue was purified by flash column chromatography (silica gel, petroleum ether: $CH_2Cl_2$ =10:1, v/v) to give the product as a yellow oil (27.7 mg, 59%).

**$^1$H NMR** (500 MHz, Chloroform-*d*) δ 7.52 (d, *J* = 7.8 Hz, 1H), 7.23 – 7.20 (m, 2H), 7.19 – 7.14 (m, 1H), 4.18 (q, *J* = 7.1 Hz, 2H), 3.69 (s, 2H), 3.64 (s, 3H), 1.27 (t, *J* = 7.1 Hz, 3H).

**$^{13}$C NMR** (126 MHz, Chloroform-*d*) δ 171.32 (d, *J* = 1.9 Hz), 150.19 (d, *J* = 266.6 Hz), 83.89 (d, *J* = 10.7 Hz), 60.91, 28.34 (d, *J* = 2.4 Hz), 27.90 (d, *J* = 2.0 Hz), 14.24.

**$^{19}$F NMR** (471 MHz, Chloroform-*d*) δ -139.50.

**HRMS** (ESI, m/z) calcd for $C_{13}H_{14}FNO_2[M+H]^+$: 236.1081; found: 236.1081.

## tert-butyl 2-(2-fluoro-1-methyl-1H-indol-3-yl)acetate (5c)

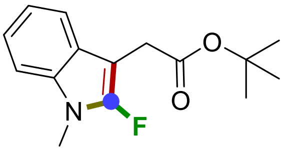

**5c**, 40%

The reaction was performed following the general procedure. The residue was purified by flash

column chromatography (silica gel, petroleum ether: $CH_2Cl_2$ =10:1, v/v) to give the product as a yellow oil (21.1 mg, 40%).

**$^1$H NMR** (500 MHz, Chloroform-*d*) δ 7.54 – 7.49 (m, 1H), 7.22 – 7.18 (m, 2H), 7.14 (m, *J* = 8.1, 5.4, 2.8 Hz, 1H), 3.64 (s, 3H), 3.59 (s, 2H), 1.45 (s, 9H).

**$^{13}$C NMR** (126 MHz, Chloroform-*d*) δ 170.6, 150.2 (d, *J* = 266.3 Hz), 130.5, 125.4 (d, *J* = 5.7 Hz), 121.1 (d, *J* = 3.6 Hz), 120.2, 118.8 (d, *J* = 6.5 Hz), 108.7 (d, *J* = 2.1 Hz), 84.4 (d, *J* = 10.9 Hz), 80.9, 29.7, 29.6 (d, *J* = 2.5 Hz), 28.1, 27.9 (d, *J* = 2.2 Hz).

**$^{19}$F NMR** (471 MHz, Chloroform-*d*) δ -139.69.

**HRMS** (ESI, m/z) calcd for $C_{15}H_{18}FNO_2[M+NA]^+$: 286.1214; found: 286.1216.

## benzyl 2-(2-fluoro-1-methyl-1H-indol-3-yl)acetate (5d)

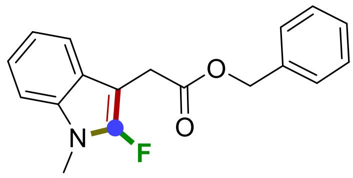

**5d**, 41%

The reaction was performed following the general procedure. The residue was purified by flash column chromatography (silica gel, petroleum ether: $CH_2Cl_2$ =10:1, v/v) to give the product as a yellow oil (24.4 mg, 41%).

**$^1$H NMR** (500 MHz, Chloroform-*d*) δ 7.52 – 7.49 (m, 1H), 7.35 (d, *J* = 1.2 Hz, 5H), 7.23 (d, *J* = 4.0 Hz, 2H), 7.16 (m, *J* = 8.1, 4.4 Hz, 1H), 5.17 (s, 2H), 3.76 (s, 2H), 3.64 (s, 3H).

**$^{13}$C NMR** (126 MHz, Chloroform-*d*) δ 171.2, 150.2 (d, *J* = 266.8 Hz), 136.0, 130.5, 128.5, 128.22, 125.3 (d, *J* = 5.7 Hz), 121.3 (d, *J* = 3.5 Hz), 120.4, 118.7, 108.8 (d, *J* = 1.8 Hz), 83.7 (d, *J* = 10.8 Hz), 66.7, 28.3 (d, *J* = 2.5 Hz), 27.9 (d, *J* = 2.1 Hz).

**$^{19}$F NMR** (471 MHz, Chloroform-*d*) δ -139.28.

**HRMS** (ESI, m/z) calcd for $C_{18}H_{16}FNO_2[M+H]^+$: 298.1238; found: 298.1235.

## 2-(2-fluoro-1-methyl-1H-indol-3-yl)acetonitrile (5e)

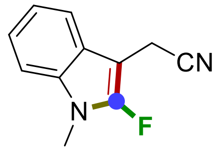

**5e**, 85%

The reaction was performed following the general procedure. The residue was purified by flash column chromatography (silica gel, petroleum ether: $CH_2Cl_2$ =10:1, v/v) to give the product as a yellow solid (32.0 mg, 85%). m.p. 111-113 °C.

**$^1$H NMR** (500 MHz, Chloroform-*d*) δ 7.57 (m, *J* = 7.7, 1.0 Hz, 1H), 7.30 – 7.19 (m, 3H), 3.75 (d, *J* = 0.8 Hz, 2H), 3.64 (s, 3H).

**$^{13}$C NMR** (126 MHz, Chloroform-*d*) δ 149.7 (d, *J* = 267.8 Hz), 130.4, 124.0 (d, *J* = 4.6 Hz), 122.0 (d, *J* = 3.3 Hz), 121.00, 118.0 (d, *J* = 6.1 Hz), 117.3, 109.2 (d, *J* = 1.8 Hz), 79.7 (d, *J* = 10.0 Hz), 28.0 (d, *J* = 2.1 Hz), 11.2 (d, *J* = 2.2 Hz).
**$^{19}$F NMR** (471 MHz, Chloroform-*d*) δ -138.09.
**HRMS** (ESI, m/z) calcd for $C_{11}H_9FN_2$[M+H]$^+$: 189.0823; found: 189.0822.

## 2,6-di-tert-butyl-4-(2-fluoro-1-methyl-1H-indol-3-yl)phenol (7a)

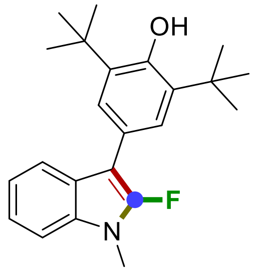


**7a**, 95%

The reaction was performed following the general procedure. The residue was purified by flash column chromatography (silica gel, petroleum ether: $CH_2Cl_2$ =10:1, v/v) to give the product as a yellow oil (67.1 mg, 95%).
**$^{1}$H NMR** (500 MHz, Chloroform-*d*) δ 7.81 (m, *J* = 7.8, 0.9 Hz, 1H), 7.49 (s, 2H), 7.31 – 7.26 (m, 2H), 7.22 – 7.18 (m, 1H), 5.21 (s, 1H), 3.71 (s, 3H), 1.52 (s, 18H).
**$^{13}$C NMR** (126 MHz, Chloroform-*d*) δ 152.8, 152.1, 148.8 (d, *J* = 268.1 Hz), 136.2, 130.5, 124.8 (d, *J* = 2.3 Hz), 124.5 (d, *J* = 5.5 Hz), 124.1, 123.4 (d, *J* = 4.0 Hz), 121.2 (d, *J* = 3.4 Hz), 120.6, 119.4 (d, *J* = 6.4 Hz), 108.9 (d, *J* = 1.8 Hz), 94.0 (d, *J* = 7.8 Hz), 34.5, 34.5, 30.4, 27.3 (d, *J* = 2.2 Hz).
**$^{19}$F NMR** (471 MHz, Chloroform-*d*) δ -139.28.
**HRMS** (ESI, m/z) calcd for $C_{23}H_{28}FNO$[M+NA]$^+$: 376.2047; found: 376.2046.

## 2,6-di-tert-butyl-4-(2-fluoro-1,5-dimethyl-1H-indol-3-yl)phenol (7b)

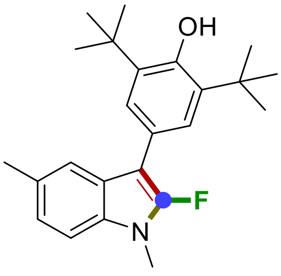


**7b**, 81%

The reaction was performed following the general procedure. The residue was purified by flash column chromatography (silica gel, petroleum ether: $CH_2Cl_2$ =10:1, v/v) to give the product as a yellow oil (59.5 mg, 81%).
**$^{1}$H NMR** (500 MHz, Chloroform-*d*) δ 7.67 (s, 1H), 7.56 (s, 2H), 7.22 (d, *J* = 8.2 Hz, 1H), 7.14 (m, *J* = 8.3, 1.6 Hz, 1H), 5.27 (s, 1H), 3.72 (s, 3H), 2.55 (s, 3H), 1.60 (s, 19H).

**$^{13}$C NMR** (126 MHz, Chloroform-*d*) δ 152.2, 148.91 (d, *J* = 267.8 Hz), 136.2, 129.9, 128.9, 125.0 (d, *J* = 2.2 Hz), 124.697 (d, *J* = 5.4 Hz), 123.6 (d, *J* = 4.0 Hz), 122.6 (d, *J* = 3.6 Hz), 119.4 (d, *J* = 6.2 Hz), 108.7 (d, *J* = 1.8 Hz), 93.7 (d, *J* = 8.0 Hz), 34.6, 30.49, 28.0 (d, *J* = 2.2 Hz), 21.9.
**$^{19}$F NMR** (471 MHz, Chloroform-*d*) δ -139.10.
**HRMS** (ESI, m/z) calcd for $C_{24}H_{30}FNO[M+H]^+$: 368.2384; found: 368.2382.

## 4-(5-bromo-2-fluoro-1-methyl-1H-indol-3-yl)-2,6-di-tert-butylphenol (7c)

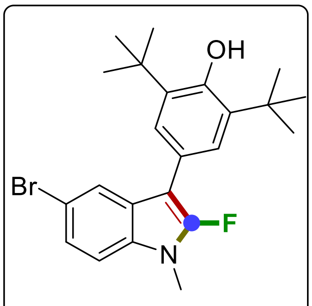


**7c**, 92%

The reaction was performed following the general procedure. The residue was purified by flash column chromatography (silica gel, petroleum ether: $CH_2Cl_2$ =10:1, v/v) to give the product as a yellow oil (79.3 mg, 92%).
**$^1$H NMR** (500 MHz, Chloroform-*d*) δ 7.92 (d, *J* = 1.9 Hz, 1H), 7.45 (s, 2H), 7.35 (m, *J* = 8.6, 1.9 Hz, 1H), 7.15 (d, *J* = 8.5 Hz, 1H), 5.27 (s, 1H), 3.70 (s, 3H), 1.54 (s, 18H).
**$^{13}$C NMR** (126 MHz, Chloroform-*d*) δ 152.5, 149.1 (d, *J* = 269.4 Hz), 136.4, 129.2, 126.2, 124.9 (d, *J* = 2.1 Hz), 124.1 (d, *J* = 3.6 Hz), 122.7 (d, *J* = 4.0 Hz), 122.1 (d, *J* = 6.6 Hz), 113.9, 110.5, 94.3, 34.51, 30.4, 28.1.
**$^{19}$F NMR** (471 MHz, Chloroform-*d*) δ -137.03.
**HRMS** (ESI, m/z) calcd for $C_{23}H_{27}BrFNO[M+NA]^+$: 454.1152; found: 454.1152.

## 2,6-di-tert-butyl-4-(4-chloro-2-fluoro-1-methyl-1H-indol-3-yl)phenol (7d)

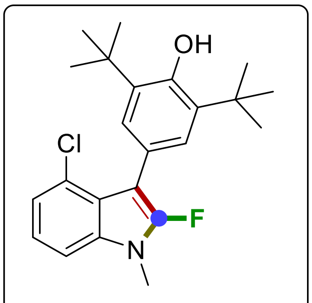


**7d**, 91%

The reaction was performed following the general procedure. The residue was purified by flash column chromatography (silica gel, petroleum ether: $CH_2Cl_2$ =10:1, v/v) to give the product as a yellow solid (70.5 mg, 91%). m.p. 129-131 ºC.
**$^1$H NMR** (500 MHz, Chloroform-*d*) δ 7.33 (s, 2H), 7.20 (m, *J* = 6.0, 3.2 Hz, 1H), 7.16 – 7.13 (m, 2H), 5.23 (s, 1H), 3.72 (s, 3H), 1.50 (s, 18H).

**$^{13}$C NMR** (126 MHz, Chloroform-*d*) δ 152.8, 149.4 (d, *J* = 266.9 Hz), 134.8, 131.6, 128.3, 126.2 (d, *J* = 6.6 Hz), 122.0, 121.9, 121.5, 121.4 (d, *J* = 3.4 Hz), 107.5, 95.2, 34.4, 30.5, 28.2.
**$^{19}$F NMR** (471 MHz, Chloroform-*d*) δ -137.89.
**HRMS** (ESI, m/z) calcd for $C_{23}H_{27}ClFNO[M+H]^+$: 388.1838; found: 388.1837.

## 2,6-di-tert-butyl-4-(2,7-difluoro-1-methyl-1H-indol-3-yl)phenol (7e)

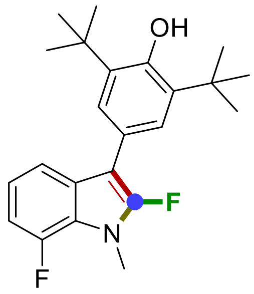


**7e**, 91%

The reaction was performed following the general procedure. The residue was purified by flash column chromatography (silica gel, petroleum ether: $CH_2Cl_2$ =10:1, v/v) to give the product as a yellow solid (67.6 mg, 91%). m.p. 121-123 °C.
**$^1$H NMR** (500 MHz, Chloroform-*d*) δ 7.53 (m, *J* = 2.7, 1.0 Hz, 2H), 7.19 (m, *J* = 8.0, 4.7 Hz, 1H), 7.09 (d, *J* = 8.0 Hz, 1H), 6.92 (m, *J* = 11.6, 7.9 Hz, 1H), 5.27 (s, 1H), 3.73 (s, 3H), 1.56 (s, 18H).
**$^{13}$C NMR** (126 MHz, Chloroform-*d*) δ 157.1 (dd, *J* = 247.2, 6.7 Hz), 152.5, 148.3 (d, *J* = 267.5 Hz), 135.5, 132.9 (d, *J* = 11.4 Hz), 126.3 (dd, *J* = 4.1, 2.2 Hz), 122.3 (d, *J* = 4.0 Hz), 121.5 (dd, *J* = 8.2, 3.2 Hz), 112.7 (dd, *J* = 18.4, 5.7 Hz), 106.6 (d, *J* = 20.9 Hz), 105.0 (t, *J* = 2.4 Hz), 93.4 (dd, *J* = 9.6, 2.7 Hz), 34.5, 30.5, 28.4 (d, *J* = 2.1 Hz).
**$^{19}$F NMR** (471 MHz, Chloroform-*d*) δ -117.19, 138.80.
**HRMS** (ESI, m/z) calcd for $C_{19}H_{16}FNO[M+H]^+$: 416.1692; found: 416.1702.

## 2-(1-(3-bromopropyl)-2-fluoro-1H-indol-3-yl)-1-phenylethan-1-onee (9a)

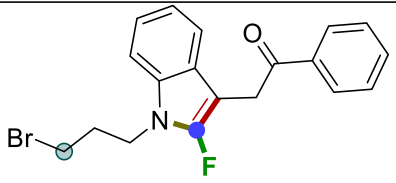


**9a**, 68%

The reaction was performed following the general procedure. The residue was purified by flash column chromatography (silica gel, petroleum ether: $CH_2Cl_2$ =10:1, v/v) to give the product as a yellow solid (50.7 mg, 68%). m.p. 162-164 °C.
**$^1$H NMR** (500 MHz, Chloroform-*d*) δ 8.11 – 8.04 (m, 2H), 7.60 – 7.53 (m, 1H), 7.52 – 7.45 (m, 3H), 7.31 – 7.28 (m, 1H), 7.23 – 7.18 (m, 1H), 7.14 (td, *J* = 7.6, 1.1 Hz, 1H), 4.32 (s, 2H), 4.24 (t, *J* = 6.6 Hz, 2H), 3.33 (t, *J* = 6.3 Hz, 2H), 2.37 – 2.29 (m, 2H).

**$^{13}$C NMR** (126 MHz, Chloroform-*d*) δ 196.5, 149.8 (d, *J* = 266.3 Hz), 136.3, 133.2, 130.0, 128.6, 128.5, 125.6 (d, *J* = 5.9 Hz), 121.5 (d, *J* = 3.4 Hz), 120.6, 119.1 (d, *J* = 6.4 Hz), 109.0, 84.7 (d, *J* = 10.5 Hz), 40.3, 32.9 (d, *J* = 2.8 Hz), 32.6, 29.9.
**$^{19}$F NMR** (471 MHz, Chloroform-*d*) δ -138.18.
**HRMS** (ESI, m/z) calcd for $C_{19}H_{17}BrFNO[M+H]^+$: 374.0550; found: 374.0547.

## 2-(1-(4-bromobutyl)-2-fluoro-1H-indol-3-yl)-1-phenylethan-1-one (9b)

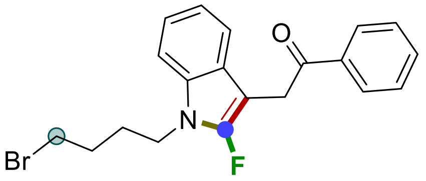

**9b**, 68%

The reaction was performed following the general procedure. The residue was purified by flash column chromatography (silica gel, petroleum ether: $CH_2Cl_2$ =10:1, v/v) to give the product as a yellow oil (52.6 mg, 68%). m.p. 165-167 °C.
**$^{1}$H NMR** (500 MHz, Chloroform-*d*) δ 8.12 – 8.02 (m, 2H), 7.58 – 7.53 (m, 1H), 7.52 – 7.49 (m, 1H), 7.47 (dd, *J* = 8.4, 7.0 Hz, 2H), 7.24 – 7.17 (m, 2H), 7.14 (m, *J* = 8.1, 6.6, 1.7 Hz, 1H), 4.32 (s, 2H), 4.09 (t, *J* = 6.7 Hz, 2H), 3.36 (t, *J* = 6.5 Hz, 2H), 2.01 – 1.90 (m, 2H), 1.86 – 1.75 (m, 2H).
**$^{13}$C NMR** (126 MHz, Chloroform-*d*) δ 196.6, 149.8 (d, *J* = 265.8 Hz), 136.4, 133.1, 129.8, 128.6, 128.5, 125. 6 (d, *J* = 6.1 Hz), 121.4 (d, *J* = 3.5 Hz), 120.5, 119.1 (d, *J* = 6.4 Hz), 109.0, 84.4 (d, *J* = 10.8 Hz), 41.2, 33.0(d, *J* = 2.8 Hz), 32.8, 29.7, 28.2.
**$^{19}$F NMR** (471 MHz, Chloroform-*d*) δ -138.37.
**HRMS** (ESI, m/z) calcd for $C_{20}H_{19}BrFNO[M+H]^+$: 388.0704; found: 388.0707.

## 2-(2-fluoro-1-(4-iodobutyl)-1H-indol-3-yl)-1-phenylethan-1-one (9c)

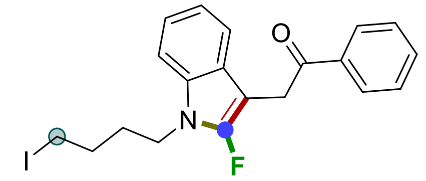

**9c**, 65%

The reaction was performed following the general procedure. The residue was purified by flash column chromatography (silica gel, petroleum ether: $CH_2Cl_2$ =10:1, v/v) to give the product as a yellow oil (56.6 mg, 65%).
**$^{1}$H NMR** (500 MHz, Chloroform-*d*) δ 8.10 – 8.04 (m, 2H), 7.59 – 7.53 (m, 1H), 7.52 – 7.41 (m, 3H), 7.23 – 7.17 (m, 2H), 7.14 (m, *J* = 8.1, 6.6, 1.7 Hz, 1H), 4.31 (s, 2H), 4.08 (t, *J* = 6.8 Hz, 2H), 3.14 (t, *J* = 6.7 Hz, 2H), 1.94 – 1.87 (m, 2H), 1.82 – 1.74 (m, 2H).
**$^{13}$C NMR** (126 MHz, Chloroform-*d*) δ 196.6, 149.7 (d, *J* = 265.9 Hz), 136.3, 133.1, 129.8, 128.6, 128.5, 125.5 (d, *J* = 6.0 Hz), 121.3 (d, *J* = 3.6 Hz), 120.5, 119.0 (d, *J* = 6.2 Hz), 109.0, 84.4 (d, *J* = 10.8 Hz), 41.0, 32.9 (d, *J* = 2.9 Hz), 30.5 (d, *J* = 11.3 Hz), 27.2, 5.5.
**$^{19}$F NMR** (471 MHz, Chloroform-*d*) δ -138.38.

**HRMS** (ESI, m/z) calcd for $C_{20}H_{19}FINO[M+H]^+$: 436.0568; found: 436.0567.

## 1-([1,1'-biphenyl]-4-yl)-2-(1-(4-bromobutyl)-2-fluoro-1H-indol-3-yl)ethan-1-one (9d)

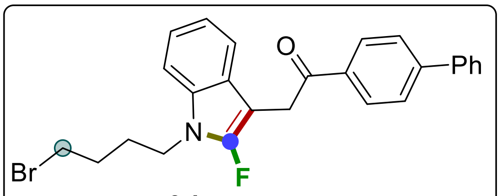


**9d**, 52%

The reaction was performed following the general procedure. The residue was purified by flash column chromatography (silica gel, petroleum ether: $CH_2Cl_2$ =10:1, v/v) to give the product as a yellow oil (48.3 mg, 52%).

**$^1$H NMR** (500 MHz, Chloroform-*d*) δ 8.14 (d, *J* = 8.3 Hz, 2H), 7.69 (d, *J* = 8.4 Hz, 2H), 7.65 – 7.60 (m, 2H), 7.54 (m, *J* = 7.6, 1.2 Hz, 1H), 7.48 (t, *J* = 7.5 Hz, 2H), 7.43 – 7.38 (m, 1H), 7.25 – 7.18 (m, 2H), 7.16 (m, *J* = 8.2, 6.6, 1.8 Hz, 1H), 4.35 (s, 2H), 4.10 (t, *J* = 6.8 Hz, 2H), 3.37 (t, *J* = 6.5 Hz, 2H), 2.01 – 1.92 (m, 2H), 1.90 – 1.78 (m, 2H).

**$^{13}$C NMR** (126 MHz, Chloroform-*d*) δ 196.2, 149.8 (d, *J* = 265.7 Hz), 145.8, 139.9, 135.0, 129.8, 129.1, 129.0, 128.3, 127.3 (d, *J* = 2.1 Hz), 125.6 (d, *J* = 5.8 Hz), 121.4 (d, *J* = 3.4 Hz), 120.5, 119.1 (d, *J* = 6.2 Hz), 84.4 (d, *J* = 10.8 Hz), 41.3, 33.0 (d, *J* = 2.7 Hz), 32.8, 29.8, 28.3.

**$^{19}$F NMR** (471 MHz, Chloroform-*d*) δ -138.34.

**HRMS** (ESI, m/z) calcd for $C_{26}H_{23}FNO[M+H]^+$: 464.1020; found: 464.1019.

## 2-(1-(4-bromobutyl)-2-fluoro-1H-indol-3-yl)-1-(4-isobutylphenyl)ethan-1-one (9e)

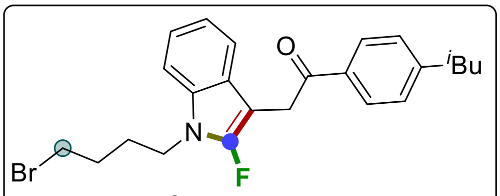


**9e**, 59%

The reaction was performed following the general procedure. The residue was purified by flash column chromatography (silica gel, petroleum ether: $CH_2Cl_2$ =10:1, v/v) to give the product as a yellow oil (52.3 mg, 59%).

**$^1$H NMR** (500 MHz, Chloroform-*d*) δ 8.01 (d, *J* = 8.3 Hz, 2H), 7.53 (d, *J* = 7.6 Hz, 1H), 7.25 (d, *J* = 8.2 Hz, 2H), 7.23 – 7.18 (m, 2H), 7.15 (m, *J* = 8.1, 6.6, 1.7 Hz, 1H), 4.31 (s, 2H), 4.09 (t, *J* = 6.7 Hz, 2H), 3.37 (t, *J* = 6.5 Hz, 2H), 2.54 (d, *J* = 7.2 Hz, 2H), 1.97 – 1.92 (m, 2H), 1.85 (dq, *J* = 10.4, 6.6 Hz, 2H), 0.93 (d, *J* = 6.6 Hz, 6H).

**$^{13}$C NMR** (126 MHz, Chloroform-*d*) δ 196.3, 149.8 (d, *J* = 265.6 Hz), 147.7, 134.2, 129.8, 129.4, 128.5, 125.6 (d, *J* = 5.9 Hz), 121.3 (d, *J* = 3.4 Hz), 120.5, 119.1 (d, *J* = 6.3 Hz), 109.0, 84.6 (d, *J* = 10.8 Hz), 45.4, 41.2, 32.8 (d, *J* = 2.3 Hz), 30.1, 29.8, 28.3, 22.4.

**$^{19}$F NMR** (471 MHz, Chloroform-*d*) δ -138.46.

**HRMS** (ESI, m/z) calcd for $C_{24}H_{27}BrFNO[M+H]^+$: 444.1333; found: 444.1331.

## 2-(1-(4-bromobutyl)-2-fluoro-1H-indol-3-yl)-1-(4-methoxyphenyl)ethan-1-one (9f)

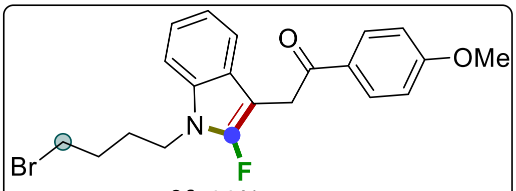


**9f**, 66%

The reaction was performed following the general procedure. The residue was purified by flash column chromatography (silica gel, petroleum ether: $CH_2Cl_2$ =10:1, v/v) to give the product as a yellow oil (55.1mg, 66%).

**$^1$H NMR** (500 MHz, Chloroform-*d*) δ 8.04 (d, *J* = 8.7 Hz, 2H), 7.51 (d, *J* = 7.8 Hz, 1H), 7.22 – 7.15 (m, 2H), 7.15 – 7.10 (m, 1H), 6.93 (d, *J* = 8.8 Hz, 2H), 4.25 (s, 2H), 4.09 (t, *J* = 6.8 Hz, 2H), 3.86 (s, 3H), 3.37 (t, *J* = 6.5 Hz, 2H), 1.95 (p, *J* = 6.7 Hz, 2H), 1.84 (dq, *J* = 13.1, 6.4 Hz, 2H).

**$^{13}$C NMR** (126 MHz, Chloroform-*d*) δ 195.2, 163.5, 149.7 (d, *J* = 265.5 Hz), 130.8, 129.6 (d, *J* = 44.9 Hz), 125.6 (d, *J* = 5.6 Hz), 121.3 (d, *J* = 3.5 Hz), 120.4, 119.3 (d, *J* = 6.3 Hz), 113.8, 108.9, 84.8 (d, *J* = 10.9 Hz), 55.5, 41.2, 32.8, 32.7 (d, *J* = 3.0 Hz), 29.8, 28.3.

**$^{19}$F NMR** (471 MHz, Chloroform-*d*) δ -138.60.

**HRMS** (ESI, m/z) calcd for $C_{21}H_{21}BrFNO_2[M+H]^+$: 418.0812; found: 418.0811.

## 2-(1-(4-bromobutyl)-2-fluoro-1H-indol-3-yl)-1-(4-bromophenyl)ethan-1-one (9g)

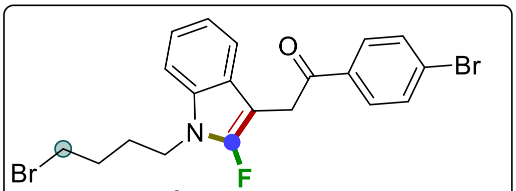


**9g**, 61%

The reaction was performed following the general procedure. The residue was purified by flash column chromatography (silica gel, petroleum ether: $CH_2Cl_2$ =10:1, v/v) to give the product as a yellow oil (56.7 mg, 61%).

**$^1$H NMR** (500 MHz, Chloroform-*d*) δ 8.63 (d, *J* = 1.8 Hz, 1H), 8.10 (m, *J* = 8.5, 1.8 Hz, 1H), 7.97 (d, *J* = 8.0 Hz, 1H), 7.88 (t, *J* = 8.7 Hz, 2H), 7.58 (m, *J* = 14.9, 9.4, 6.8, 1.3 Hz, 3H), 7.23 – 7.13 (m, 3H), 4.45 (s, 2H), 4.08 (t, *J* = 6.8 Hz, 2H), 3.30 (t, *J* = 6.5 Hz, 2H), 1.93 (p, *J* = 6.8 Hz, 2H), 1.86 – 1.74 (m, 2H).

**$^{13}$C NMR** (126 MHz, Chloroform-*d*) δ 196.6, 149.8 (d, *J* = 265.5 Hz), 135.6, 133.6, 132.5, 130.2, 129.8, 129.6, 128.5, 128.5, 127.8, 126. 8, 125.6 (d, *J* = 5.9 Hz), 124.3, 121.4 (d, *J* = 3.5 Hz), 120.5, 119.1 (d, *J* = 6.3 Hz), 109.0, 84.6 (d, *J* = 10.6 Hz), 41.3, 33.1 (d, *J* = 2.8 Hz), 32.8, 29.7, 28.2.

**$^{19}$F NMR** (471 MHz, Chloroform-*d*) δ -138.22.

**HRMS** (ESI, m/z) calcd for $C_{20}H_{18}BrFNO[M+K]^+$: 503.9371; found:503.9381.

## 2-(1-(4-bromobutyl)-2-fluoro-1H-indol-3-yl)-1-(4-iodophenyl)ethan-1-one (9h)

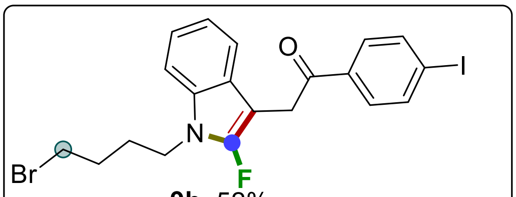


The reaction was performed following the general procedure. The residue was purified by flash column chromatography (silica gel, petroleum ether: $CH_2Cl_2$ =10:1, v/v) to give the product as a yellow oil (53.4 mg, 52%).

**$^{1}$H NMR** (500 MHz, Chloroform-*d*) δ 7.81 (d, *J* = 8.5 Hz, 2H), 7.75 (d, *J* = 8.5 Hz, 2H), 7.48 (dd, *J* = 7.8, 1.2 Hz, 1H), 7.23 – 7.17 (m, 2H), 7.14 (m, *J* = 8.1, 6.4, 1.9 Hz, 1H).

**$^{13}$C NMR** (126 MHz, Chloroform-*d*) δ 195.9, 149.7 (d, *J* = 265.7 Hz), 137.9, 135.5, 129.9, 129.8, 125.4 (d, *J* = 5.6 Hz), 121.5 (d, *J* = 3.5 Hz), 120.6, 118.9 (d, *J* = 6.2 Hz), 109.0, 101.1, 84.0 (d, *J* = 10.6 Hz), 41.3, 33.0 (d, *J* = 2.7 Hz), 32.7, 29.8, 28.2.

**$^{19}$F NMR** (471 MHz, Chloroform-*d*) δ -138.22.

**HRMS** (ESI, m/z) calcd for $C_{20}H_{18}FINO[M+H]^+$: 513.9673; found: 513.9674.

## 2-(1-(4-bromobutyl)-2-fluoro-1H-indol-3-yl)-1-(o-tolyl)ethan-1-one (9i)

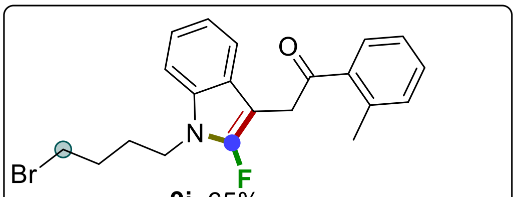


The reaction was performed following the general procedure. The residue was purified by flash column chromatography (silica gel, petroleum ether: $CH_2Cl_2$ =10:1, v/v) to give the product as a yellow oil (52.1 mg, 65%).

**$^{1}$H NMR** (500 MHz, Chloroform-*d*) δ 7.78 (dd, *J* = 7.7, 1.4 Hz, 1H), 7.47 (d, *J* = 7.6 Hz, 1H), 7.35 (td, *J* = 7.5, 1.4 Hz, 1H), 7.26 (s, 1H), 7.23 – 7.17 (m, 3H), 7.16 – 7.11 (m, 1H), 4.24 (s, 2H), 4.07 (t, *J* = 6.8 Hz, 2H), 3.35 (t, *J* = 6.5 Hz, 2H), 2.45 (s, 3H).

**$^{13}$C NMR** (126 MHz, Chloroform-*d*) δ 200.8, 149.9 (d, *J* = 265.8 Hz), 138.6, 137.4, 132.0, 131.2, 129.7, 128.4, 125.6, 125.6, 121.3 (d, *J* = 3.5 Hz), 120.5, 118.9 (d, *J* = 6.3 Hz), 109.0, 84.5 (d, *J* = 11.0 Hz), 41.2, 35.7 (d, *J* = 3.1 Hz), 32.8, 29.7, 28.2, 21.2.

**$^{19}$F NMR** (471 MHz, Chloroform-*d*) δ -138.61.

**HRMS** (ESI, m/z) calcd for $C_{21}H_{21}BrFNO[M+H]^+$: 402.0863; found: 402.0864.

## 2-(1-(4-bromobutyl)-2-fluoro-1H-indol-3-yl)-1-(2-(trifluoromethyl)phenyl)ethan-1-one (9j)

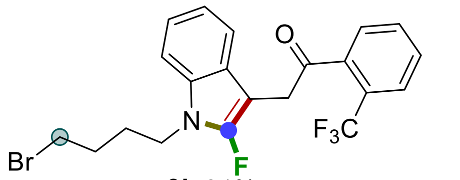

**9j**, 31%

The reaction was performed following the general procedure. The residue was purified by flash column chromatography (silica gel, petroleum ether: $CH_2Cl_2$ =10:1, v/v) to give the product as a yellow oil (28.2 mg, 31%).

**$^{1}$H NMR** (500 MHz, Chloroform-*d*) δ 7.73 (m, *J* = 6.7, 2.4 Hz, 1H), 7.59 – 7.53 (m, 2H), 7.47 (d, *J* = 7.9 Hz, 1H), 7.44 – 7.40 (m, 1H), 7.27 – 7.21 (m, 2H), 7.18 (m, *J* = 8.1, 6.4, 1.9 Hz, 1H), 4.21 (s, 2H), 4.10 (t, *J* = 6.8 Hz, 2H), 3.39 (t, *J* = 6.5 Hz, 2H), 2.01 – 1.91 (m, 2H), 1.86 – 1.75 (m, 2H).

**$^{13}$C NMR** (126 MHz, Chloroform-*d*) δ 200.8, 150.2 (d, *J* = 267.0 Hz), 139.7, 131.6, 129.8 (d, *J* = 36.6 Hz), 127.2, 126.7 (q, *J* = 4.8 Hz), 125.4 (d, *J* = 5.8 Hz), 121.4 (d, *J* = 3.4 Hz), 120.6, 118.7 (d, *J* = 6.3 Hz), 109.0, 83.2 (d, *J* = 11.0 Hz), 41.2, 37.1, 32.7, 29.7, 28.2.

**$^{19}$F NMR** (471 MHz, Chloroform-*d*) δ -57.95, -138.35.

**HRMS** (ESI, m/z) calcd for $C_{21}H_{18}BrF_4NO[M+H]^+$: 456.0581; found: 456.0583.

## 2-(1-(4-bromobutyl)-2-fluoro-1H-indol-3-yl)-1-(3-chlorophenyl)ethan-1-one (9k)

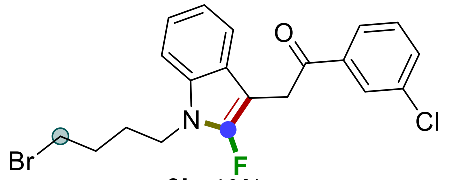

**9k**, 48%

The reaction was performed following the general procedure. The residue was purified by flash column chromatography (silica gel, petroleum ether: $CH_2Cl_2$ =10:1, v/v) to give the product as a yellow solid (40.4 mg, 48%). m.p. 167-169 °C.

**$^{1}$H NMR** (500 MHz, Chloroform-*d*) δ 8.03 (t, *J* = 1.9 Hz, 1H), 7.93 (m, *J* = 7.8, 1.4 Hz, 1H), 7.54 – 7.47 (m, 2H), 7.40 (t, *J* = 7.9 Hz, 1H), 7.24 – 7.18 (m, 2H), 7.15 (m, *J* = 8.1, 6.4, 1.9 Hz, 1H), 4.28 (s, 2H), 4.09 (t, *J* = 6.8 Hz, 2H), 3.37 (t, *J* = 6.5 Hz, 2H), 1.98 – 1.92 (m, 2H), 1.85 – 1.78 (m, 2H).

**$^{13}$C NMR** (126 MHz, Chloroform-*d*) δ 195.3, 149.7 (d, *J* = 265.8 Hz), 137.8, 134.9, 133.0, 130.0, 129.8, 128.6, 126.6, 125.4 (d, *J* = 5.8 Hz), 121.5 (d, *J* = 3.5 Hz), 120.60, 119.0 (d, *J* = 6.3 Hz), 109.1, 83.9 (d, *J* = 10.8 Hz), 41.3, 33.1 (d, *J* = 2.8 Hz), 3
2.8, 29.7, 28.2.

**$^{19}$F NMR** (471 MHz, Chloroform-*d*) δ -138.14.

**HRMS** (ESI, m/z) calcd for $C_{20}H_{18}FBrClNO[M+H]^+$: 422.0317; found: 422.0316.

## 1-(3-chlorophenyl)-2-(2-fluoro-1-(4-iodobutyl)-1H-indol-3-yl)ethan-1-one (9l)

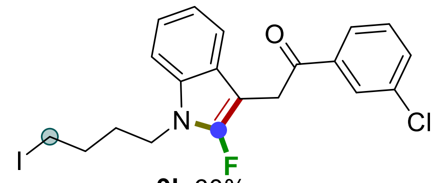


**9l**, 60%

The reaction was performed following the general procedure. The residue was purified by flash column chromatography (silica gel, petroleum ether: $CH_2Cl_2$ =10:1, v/v) to give the product as a yellow oil (56.3 mg, 60%).

**$^{1}$H NMR** (500 MHz, Chloroform-*d*) δ 8.04 (t, *J* = 1.9 Hz, 1H), 7.93 (m, *J* = 7.8, 1.4 Hz, 1H), 7.54 – 7.47 (m, 2H), 7.40 (t, *J* = 7.9 Hz, 1H), 7.25 – 7.18 (m, 2H), 7.16 (m, *J* = 8.2, 6.2, 2.2 Hz, 1H), 4.29 (s, 2H), 4.08 (t, *J* = 6.7 Hz, 2H), 3.14 (t, *J* = 6.7 Hz, 2H), 1.91 (m, *J* = 14.8, 6.7 Hz, 2H), 1.85 – 1.75 (m, 2H).

**$^{13}$C NMR** (126 MHz, Chloroform-*d*) δ 195.3, 149.7 (d, *J* = 265.9 Hz), 137.8, 134.9, 133.1, 130.0, 129.9, 128.6, 126.6, 125.4 (d, *J* = 5.8 Hz), 121.5 (d, *J* = 3.3 Hz), 120.61, 112.0 (d, *J* = 6.3 Hz), 109.1 (d, *J* = 1.6 Hz), 83.9 (d, *J* = 10.8 Hz), 41.1, 33.1 (d, *J* = 2.7 Hz), 30.5 (d, *J* = 11.0 Hz), 28.2, 5.6.

**$^{19}$F NMR** (471 MHz, Chloroform-*d*) δ -138.10.

**HRMS** (ESI, m/z) calcd for $C_{20}H_{18}ClFINO[M+H]^+$: 470.0178; found: 470.0174.

## 2-(1-(4-bromobutyl)-2-fluoro-1H-indol-3-yl)-1-(3,4,5-trimethoxyphenyl)ethan-1-one (9m)

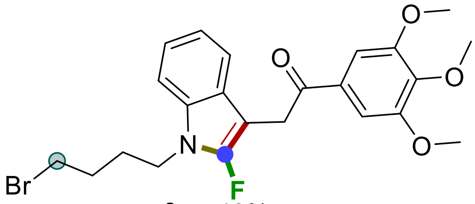


**9m**, 48%

The reaction was performed following the general procedure. The residue was purified by flash column chromatography (silica gel, petroleum ether: $CH_2Cl_2$ =10:1, v/v) to give the product as a yellow oil (45.8 mg, 48%).

**$^{1}$H NMR** (500 MHz, Chloroform-*d*) δ 7.56 (m, *J* = 7.5, 1.0 Hz, 1H), 7.33 (s, 2H), 7.23 – 7.17 (m, 2H), 7.14 (m, *J* = 8.1, 6.5, 1.8 Hz, 1H), 4.27 (s, 2H), 4.09 (t, *J* = 6.8 Hz, 2H), 3.90 (s, 3H), 3.88 (s, 6H), 3.36 (t, *J* = 6.5 Hz, 2H), 1.99 – 1.92 (m, 2H), 1.83 (m, *J* = 12.6, 9.1, 6.1 Hz, 2H).

**$^{13}$C NMR** (126 MHz, Chloroform-*d*) δ 195.3, 153.0, 149.6 (d, *J* = 265.2 Hz), 142.5, 131.3, 129.8, 125.4 (d, *J* = 6.0 Hz), 121.5 (d, *J* = 3.4 Hz), 120.6, 119.1 (d, *J* = 6.3 Hz), 109.0, 106.03, 84.6 (d, *J* = 10.7 Hz), 61.0, 56.2, 41.3, 33.0 (d, *J* = 2.7 Hz), 32.7, 29.8, 28.3.

**$^{19}$F NMR** (471 MHz, Chloroform-*d*) δ -138.51.

**HRMS** (ESI, m/z) calcd for $C_{23}H_{25}BrFNO_4[M+H]^+$: 478.1024; found: 478.1020.

## 2-(1-(4-bromobutyl)-2-fluoro-1H-indol-3-yl)-1-(naphthalen-2-yl)ethan-1-one (9n)

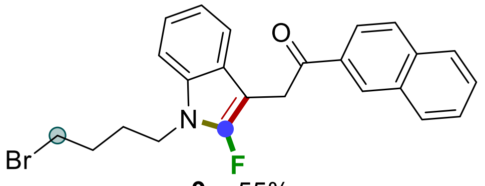


**9n**, 55%

The reaction was performed following the general procedure. The residue was purified by flash column chromatography (silica gel, petroleum ether: $CH_2Cl_2$ =10:1, v/v) to give the product as a yellow solid (48.1 mg, 55%). m.p. 139-141 ºC.

**$^{1}$H NMR** (500 MHz, Chloroform-*d*) δ 8.65 (d, *J* = 1.7 Hz, 1H), 8.12 (dd, *J* = 8.6, 1.8 Hz, 1H), 8.00 (dd, *J* = 8.2, 1.3 Hz, 1H), 7.90 (t, *J* = 8.7 Hz, 2H), 7.65 – 7.56 (m, 3H), 7.24 – 7.16 (m, 3H), 4.47 (s, 2H), 4.11 (t, *J* = 6.8 Hz, 2H), 3.33 (t, *J* = 6.5 Hz, 2H), 2.00 – 1.92 (m, 2H), 1.85 – 1.78 (m, **2H).**

**$^{13}$C NMR** (126 MHz, Chloroform-*d*) δ 196.6, 149.8 (d, *J* = 265.3 Hz), 135.6, 133.6, 132.5, 130.2 129.8, 129.6, 128.5 (d, *J* = 6.2 Hz), 127.8, 126.77, 125.6 (d, *J* = 5.9 Hz), 124.3, 121.38 (d, *J* = 3.6 Hz), 120.5, 119.1 (d, *J* = 6.3 Hz), 109.0, 84.6 (d, *J* = 10.9 Hz), 41.3, 33.1 (d, *J* = 2.7 Hz), 32.8, 29.7, 28.2.

**$^{19}$F NMR** (471 MHz, Chloroform-*d*) δ -138.24.

**HRMS** (ESI, m/z) calcd for $C_{24}H_{21}BrFNO[M+H]^+$: 438.0863; found: 438.0867.

## 2-(1-(4-bromobutyl)-2-fluoro-1H-indol-3-yl)-1-(furan-2-yl)ethan-1-one (9o)

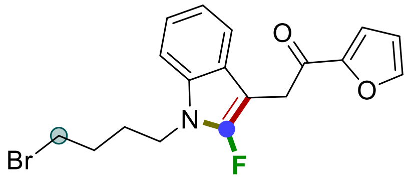


**9o**, 53%

The reaction was performed following the general procedure. The residue was purified by flash column chromatography (silica gel, petroleum ether: $CH_2Cl_2$ =10:1, v/v) to give the product as a yellow oil (40.0 mg, 53%).

**$^{1}$H NMR** (500 MHz, Chloroform-*d*) δ 7.59 (d, *J* = 1.7 Hz, 1H), 7.54 (dt, *J* = 7.7, 1.0 Hz, 1H), 7.27 – 7.25 (m, 1H), 7.23 – 7.17 (m, 2H), 7.14 (m, *J* = 8.1, 6.7, 1.6 Hz, 1H), 4.16 (s, 2H), 4.09 (t, *J* = 6.7 Hz, 2H), 3.38 (t, *J* = 6.5 Hz, 2H), 1.99 – 1.93 (m, 2H), 1.88 – 1.83 (m, 2H).

**$^{13}$C NMR** (126 MHz, Chloroform-*d*) δ 185.6, 152.2, 150.0 (d, *J* = 266.2 Hz), 146.6, 129.7, 125.5 (d, *J* = 5.7 Hz), 121.4 (d, *J* = 3.5 Hz), 120.5, 119.1 (d, *J* = 6.3 Hz), 117.5, 112.3, 108.9, 83.95 (d, *J* = 11.0 Hz), 41.3, 32.8, 32.7 (d, *J* = 2.6 Hz), 29.8, 28.3.

**$^{19}$F NMR** (471 MHz, Chloroform-*d*) δ -138.54.

**HRMS** (ESI, m/z) calcd for $C_{18}H_{17}B_rFNO[M+H]^+$: 378.0499; found: 378.0501.

## 2-(2-fluoro-1-(4-iodobutyl)-1H-indol-3-yl)-1-(furan-2-yl)ethan-1-one (9p)

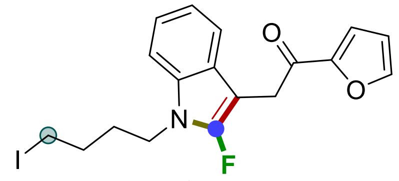


**9p**, 62%

The reaction was performed following the general procedure. The residue was purified by flash column chromatography (silica gel, petroleum ether: $CH_2Cl_2$ =10:1, v/v) to give the product as a yellow oil (52.7 mg, 62%).

**$^1$H NMR** (500 MHz, Chloroform-*d*) δ 7.60 (m, *J* = 1.8, 0.8 Hz, 1H), 7.55 (m, *J* = 7.6, 1.0 Hz, 1H), 7.26 (dd, *J* = 3.6, 0.8 Hz, 1H), 7.24 – 7.17 (m, 2H), 7.14 (m, *J* = 8.1, 6.6, 1.7 Hz, 1H), 6.53 (m, *J* = 3.5, 1.7 Hz, 1H), 4.17 (s, 2H), 4.07 (t, *J* = 6.7 Hz, 2H), 3.15 (t, *J* = 6.7 Hz, 2H), 1.91 (m, *J* = 8.9, 6.9, 5.0 Hz, 2H), 1.85 – 1.76 (m, 2H).

**$^{13}$C NMR** (126 MHz, Chloroform-*d*) δ 185.6, 152.2, 150.0 (d, *J* = 266.3 Hz), 146.5, 129.7, 125.5 (d, *J* = 5.8 Hz), 121.4 (d, *J* = 3.4 Hz), 120.5, 119.1 (d, *J* = 6.3 Hz), 117.6, 112.4, 109.0 (d, *J* = 1.7 Hz), 84.0 (d, *J* = 10.9 Hz), 41.0, 32.7 (d, *J* = 2.7 Hz), 30.5 (d, *J* = 8.2 Hz), 28.3, 5.6.

**$^{19}$F NMR** (471 MHz, Chloroform-*d*) δ -138.49.

**HRMS** (ESI, m/z) calcd for $C_{18}H_{17}FINO[M+H]^+$: 426.0361; found: 426.0360.

**2-(1-(4-bromobutyl)-2-fluoro-1H-indol-3-yl)-1-(thiophen-2-yl)ethan-1-one (9q)**

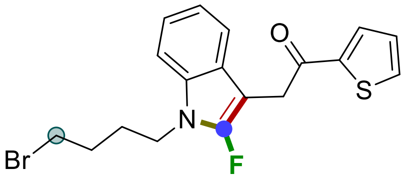


**9q**, 50%

The reaction was performed following the general procedure. The residue was purified by flash column chromatography (silica gel, petroleum ether: $CH_2Cl_2$ =10:1, v/v) to give the product as a yellow oil (39.3 mg, 50%).

**$^1$H NMR** (500 MHz, Chloroform-*d*) δ 7.85 (m, *J* = 3.8, 1.2 Hz, 1H), 7.61 (m, *J* = 4.9, 1.2 Hz, 1H), 7.56 (m, *J* = 7.8, 1.1 Hz, 1H), 7.24 – 7.18 (m, 2H), 7.17 – 7.11 (m, 2H), 4.24 (s, 2H), 4.09 (t, *J* = 6.8 Hz, 2H), 3.37 (t, *J* = 6.5 Hz, 2H), 1.99 – 1.93 (m, 2H), 1.85 (mm, *J* = 12.8, 9.1, 6.3 Hz, 2H).

**$^{13}$C NMR** (126 MHz, Chloroform-*d*) δ 189.6, 149.8 (d, *J* = 266.0 Hz), 143.4, 133.8, 132.3, 129.8, 128.1, 125.5 (d, *J* = 5.8 Hz), 121.5 (d, *J* = 3.6 Hz), 120.6, 119.2 (d, *J* = 6.3 Hz), 109.0 (d, *J* = 1.7 Hz), 84.5 (d, *J* = 10.8 Hz), 41.3, 33.8 (d, *J* = 2.8 Hz), 32.8, 29.8, 28.3.

**$^{19}$F NMR** (471 MHz, Chloroform-*d*) δ -138.42.

**HRMS** (ESI, m/z) calcd for $C_{18}H_{17}BrFNOS[M+H]^+$: 393.0198; found: 393.0197.

2-(1-(4-bromobutyl)-2-fluoro-5-methyl-1H-indol-3-yl)-1-phenylethan-1-one **(9r)**

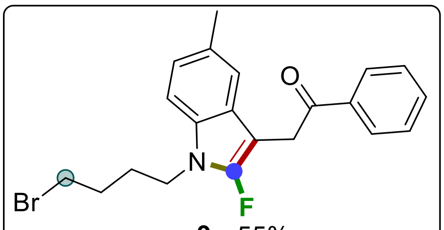

**9r**, 55%

The reaction was performed following the general procedure. The residue was purified by flash column chromatography (silica gel, petroleum ether: $CH_2Cl_2$ =10:1, v/v) to give the product as a yellow oil (42.6 mg, 55%).

**$^{1}$H NMR** (500 MHz, Chloroform-*d*) δ 8.10 (m, *J* = 8.3, 1.3 Hz, 2H), 7.61 – 7.56 (m, 1H), 7.49 (t, *J* = 7.7 Hz, 2H), 7.35 – 7.32 (m, 1H), 7.13 (d, *J* = 8.2 Hz, 1H), 7.04 (m, *J* = 8.3, 1.6 Hz, 1H), 4.32 (s, 2H), 4.08 (t, *J* = 6.7 Hz, 2H), 3.38 (t, *J* = 6.5 Hz, 2H), 2.46 (s, 3H), 2.00 – 1.92 (m, 2H), 1.87 – 1.80 (m, 2H).

**$^{13}$C NMR** (126 MHz, Chloroform-*d*) δ 196.7, 149.8 (d, *J* = 265.5 Hz), 136.4, 133.1, 129.9, 128.6, 128.5, 128.0, 125.7 (d, *J* = 5.9 Hz), 122.8 (d, *J* = 3.5 Hz), 118.9 (d, *J* = 6.0 Hz), 108.8, 83.9 (d, *J* = 10.8 Hz), 41.2, 33.0 (d, *J* = 2.8 Hz), 32.8, 29.73, 28.2, 21.6.

**$^{19}$F NMR** (471 MHz, Chloroform-*d*) δ -138.22.

**HRMS** (ESI, m/z) calcd for $C_{20}H_{19}BrFNO$ $[M+NA]^+$: 410.0526; found: 410.0524.

## 2-(1-(4-bromobutyl)-2-fluoro-6-methyl-1H-indol-3-yl)-1-phenylethan-1-one (9s)

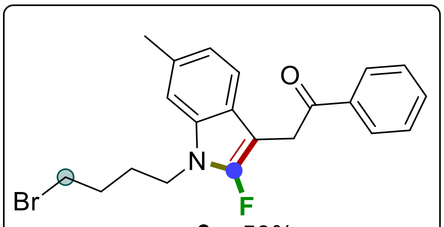

**9s**, 59%

The reaction was performed following the general procedure. The residue was purified by flash column chromatography (silica gel, petroleum ether: $CH_2Cl_2$ =10:1, v/v) to give the product as a yellow oil (47.3 mg, 59%).

**$^{1}$H NMR** (500 MHz, Chloroform-*d*) δ 8.05 (d, *J* = 7.0 Hz, 2H), 7.56 – 7.51 (m, 1H), 7.45 (m, *J* = 8.4, 7.0 Hz, 2H), 7.38 (d, *J* = 8.0 Hz, 1H), 7.00 (s, 1H), 6.96 (dd, *J* = 8.1, 1.4 Hz, 1H), 4.28 (s, 2H), 4.05 (t, *J* = 6.7 Hz, 2H), 3.36 (t, *J* = 6.5 Hz, 2H), 2.45 (s, 3H), 1.98 – 1.90 (m, 2H), 1.87 – 1.77 (m, 2H).

**$^{13}$C NMR** (126 MHz, Chloroform-*d*) δ 196.7, 149.3 (d, *J* = 264.9 Hz), 136.4, 133.0, 131.2 (d, *J* = 3.5 Hz), 130.0, 128.6 128.5, 123.2 (d, *J* = 5.8 Hz), 122.0, 118.8 (d, *J* = 6.2 Hz), 109.0, 84.1 (d, *J* = 10.7 Hz), 41.1, 33.1 (d, *J* = 3.0 Hz), 32.8, 29.8, 28.2, 21.9.

**$^{19}$F NMR** (471 MHz, Chloroform-*d*) δ -139.28.

**HRMS** (ESI, m/z) calcd for $C_{20}H_{21}BrFNO[M+H]^+$: 402.0863; found: 402.0863.

## 1-(1-(4-bromobutyl)-2-fluoro-6-methyl-1H-indol-3-yl)-4-phenylbutan-2-one (9t)

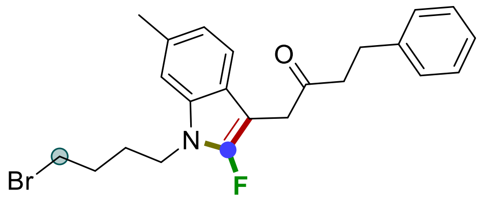

**9t**, 67%

The reaction was performed following the general procedure. The residue was purified by flash column chromatography (silica gel, petroleum ether: $CH_2Cl_2$ =10:1, v/v) to give the product as a yellow oil (56.1 mg, 67%).

**$^{1}$H NMR** (500 MHz, Chloroform-*d*) δ 7.41 (d, *J* = 7.8 Hz, 1H), 7.25 (t, *J* = 7.2 Hz, 4H), 7.23 – 7.19 (m, 1H), 7.19 – 7.16 (m, 1H), 7.16 – 7.13 (m, 2H), 4.12 (t, *J* = 6.8 Hz, 2H), 3.72 (s, 2H), 3.40 (t, *J* = 6.4 Hz, 2H), 2.95 – 2.88 (m, 2H), 2.87 – 2.79 (m, 2H), 2.05 – 1.95 (m, 2H), 1.94 – 1.83 (m, 2H).

**$^{13}$C NMR** (126 MHz, Chloroform-*d*) δ 207.2, 150.0 (d, *J* = 266.0 Hz), 141.0, 129.7, 128.4, 128.3, 126.1, 125.4 (d, *J* = 5.9 Hz), 121.5 (d, *J* = 3.4 Hz), 120.6, 118.7 (d, *J* = 6.3 Hz), 109.0, 84.1 (d, *J* = 11.1 Hz), 42.8, 41.3, 37.1 (d, *J* = 2.8 Hz), 32.7, 29.8, 29.8, 28.3.

**$^{19}$F NMR** (471 MHz, Chloroform-*d*) δ -139.10.

**HRMS** (ESI, m/z) calcd for $C_{22}H_{27}BrFNO[M+K]^+$: 454.0579; found: 454.0577.

### 1-(1-(4-bromobutyl)-2-fluoro-1H-indol-3-yl)nonan-2-one (9u)

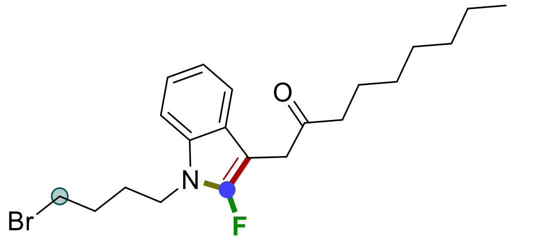

**9u**, 43%

The reaction was performed following the general procedure. The residue was purified by flash column chromatography (silica gel, petroleum ether: $CH_2Cl_2$ =10:1, v/v) to give the product as a yellow oil (35.2 mg, 43%).

**$^{1}$H NMR** (500 MHz, Chloroform-*d*) δ 7.43 (d, *J* = 7.8 Hz, 1H), 7.26 – 7.19 (m, 2H), 7.14 (mm, *J* = 8.0, 6.8, 1.4 Hz, 1H), 4.11 (t, *J* = 6.8 Hz, 2H), 3.70 (s, 2H), 3.39 (t, *J* = 6.5 Hz, 2H), 2.47 (t, *J* = 7.4 Hz, 2H), 1.97 (mm, *J* = 14.0, 7.8, 3.9 Hz, 2H), 1.91 – 1.84 (m, 2H), 1.56 (p, *J* = 7.5 Hz, 2H), 1.25 (d, *J* = 19.2 Hz, 8H), 0.86 (t, *J* = 7.0 Hz, 3H).

**$^{13}$C NMR** (126 MHz, Chloroform-*d*) δ 208.4, 145.0 (d, *J* = 266.1 Hz), 129.7, 125.5 (d, *J* = 5.9 Hz), 121.4 (d, *J* = 3.4 Hz), 120.5, 118.7 (d, *J* = 6.4 Hz), 109.0, 84.4 (d, *J* = 11.1 Hz), 41.4, 41.26, 36.9 (d, *J* = 2.8 Hz), 32.7, 31.6, 29.8, 29.1, 29.1, 28.3, 23.9, 22.6, 14.1.

**$^{19}$F NMR** (471 MHz, Chloroform-*d*) δ -139.25.

**HRMS** (ESI, m/z) calcd for $C_{21}H_{29}BrFNO[M+H]^+$: 410.1489; found: 410.1487

### 2-(-2-fluoro-1-methyl-1H-indol-3-yl)-1- ferroceneyl -1-one (11a)

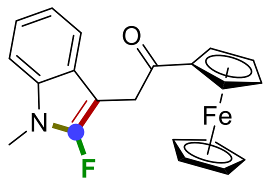

**11a**, 55%

The reaction was performed following the general procedure. The residue was purified by flash column chromatography (silica gel, petroleum ether: $CH_2Cl_2$ =10:1, v/v) to give the product as a yellow oil (41.3 mg, 55%).

**$^1$H NMR** (500 MHz, Chloroform-*d*) δ 7.61 – 7.55 (m, 1H), 7.23 – 7.18 (m, 2H), 7.16 (m, *J* = 5.5, 2.9 Hz, 1H), 4.89 (t, *J* = 2.0 Hz, 2H), 4.51 (t, *J* = 2.0 Hz, 2H), 4.15 (s, 5H), 4.05 (s, 2H), 3.66 (s, 3H).

**$^{13}$C NMR** (126 MHz, Chloroform-*d*) δ 200.9, 149.6 (d, *J* = 333.6 Hz), 130.5, 125.6, 121.2 (d, *J* = 3.5 Hz), 120.5, 119.1 (d, *J* = 6.4 Hz), 108.8, 85.0 (d, *J* = 10.9 Hz), 78.3, 72.3, 69.9, 69.6, 34.0 (d, *J* = 3.0 Hz), 28.0.

**$^{19}$F NMR** (471 MHz, Chloroform-*d*) δ -139.24.

**HRMS** (ESI, m/z) calcd for $C_{21}H_{18}FFeNO[M+H]^+$: 376.0795; found: 376.0797.

## 4-(2-(2-fluoro-1-methyl-1H-indol-3-yl)acetyl)phenyl (tert-butoxycarbonyl)-D-valinate (11b)

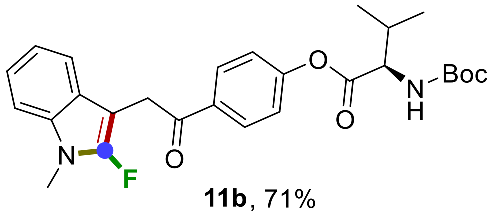

**11b**, 71%

The reaction was performed following the general procedure. The residue was purified by flash column chromatography (silica gel, petroleum ether: $CH_2Cl_2$ =10:1, v/v) to give the product as a yellow oil (68.5 mg, 71%).

**$^1$H NMR** (500 MHz, Chloroform-*d*) δ 8.14 – 8.07 (m, 2H), 7.52 – 7.47 (m, 1H), 7.24 – 7.16 (m, 4H), 7.13 (m, *J* = 8.1, 5.1, 3.2 Hz, 1H), 5.04 (m, *J* = 32.5, 8.3 Hz, 1H), 4.46 (m, *J* = 9.0, 4.9 Hz, 1H), 4.28 (s, 2H), 3.63 (s, 3H), 2.33 (s, 1H), 1.47 (s, 9H), 1.09 (d, *J* = 6.8 Hz, 3H), 1.02 (d, *J* = 6.9 Hz, 3H).

**$^{13}$C NMR** (126 MHz, Chloroform-*d*) δ 195.3, 170.7, 155.7, 154.1, 149.9 (d, *J* = 265.8 Hz), 134.1, 130.2, 125.3 (d, *J* = 5.7 Hz), 121.7, 121.3 (d, *J* = 3.5 Hz), 120.5, 118.8 (d, *J* = 6.4 Hz), 108.9, 83.8 (d, *J* = 10.8 Hz), 80.2, 58.8, 33.0 (d, *J* = 2.9 Hz), 31.3, 28.3, 28.0 (d, *J* = 2.0 Hz), 19.1, 17.7.

**$^{19}$F NMR** (471 MHz, Chloroform-*d*) δ -138.80.

**HRMS** (ESI, m/z) calcd for $C_{27}H_{31}FN_2O_5[M+H]^+$:483.2290 ; found: 483.2287

**1-(tert-butyl) 2-(4-(2-(2-fluoro-1-methyl-1H-indol-3-yl)acetyl)phenyl) (S)-pyrrolidine-1,2-dicarboxylate (11c)**

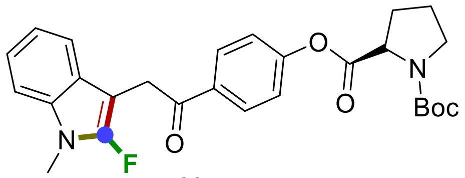


**11c**, 75%

The reaction was performed following the general procedure. The residue was purified by flash column chromatography (silica gel, petroleum ether: $CH_2Cl_2$ =10:1, v/v) to give the product as a yellow oil (72.0 mg, 75%).

**$^1$H NMR** (500 MHz, Chloroform-*d*) δ 8.10 (m, *J* = 14.4, 8.3 Hz, 2H), 7.48 (d, *J* = 7.8 Hz, 1H), 7.23 – 7.10 (m, 5H), 4.57 – 4.41 (m, 1H), 4.29 (s, 2H), 3.64 (s, 3H), 3.61 – 3.41 (m, 2H), 2.42 – 2.14 (m, 2H), 2.07 – 1.94 (m, 2H), 1.47 (d, *J* = 12.3 Hz, 9H).

**$^{13}$C NMR** (126 MHz, Chloroform-*d*) δ 195.4 (d, *J* = 19.6 Hz), 171.1, 154.5, 154.2, 153.7, 149.9 (d, *J* = 265.8 Hz), 134.0 130.6, 130.3, 130.1, 125.3, 120.5 (d, *J* = 2.9 Hz), 118.8 (d, *J* = 6.0 Hz), 108.9, 83.8, 80.3 (d, *J* = 26.8 Hz), 59.2, 59.1, 46.7, 46.5, 33.0 (d, *J* = 3.2 Hz), 31.1, 30.0, 28.4, 28.0, 24.6, 23.8.

**$^{19}$F NMR** (471 MHz, Chloroform-*d*) δ -138.80.

**HRMS** (ESI, m/z) calcd for $C_{27}H_{29}FN_2O_5[M+H]^+$: 481.2133; found: 481.2131.

## 2-(2-fluoro-1-methyl-1H-indol-3-yl)-1-((3S,8S,9S,10R,14S,17R)-3-hydroxy-10-methyl-2,3,4,7,8,9,10,11,12,13,14,15,16,17-tetradecahydro-1H-cyclopenta[a]phenanthren-17-yl)ethan-1-one (11d)

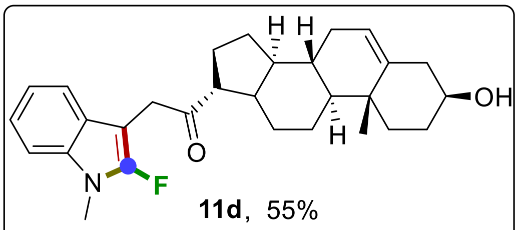


**11d**, 55%

The reaction was performed following the general procedure. The residue was purified by flash column chromatography (silica gel, petroleum ether: $CH_2Cl_2$ =10:1, v/v) to give the product as a yellow solid (49.4 mg, 55%). m.p. 141-143 °C.

**$^1$H NMR** (500 MHz, Chloroform-*d*) δ 7.39 (d, *J* = 7.8 Hz, 1H), 7.22 – 7.17 (m, 2H), 7.12 (ddd, *J* = 8.1, 5.8, 2.4 Hz, 1H), 5.34 (m, *J* = 4.9, 2.5 Hz, 1H), 3.76 – 3.69 (m, 2H), 3.64 (s, 3H), 3.56 – 3.48 (m, 1H), 2.71 (t, *J* = 9.0 Hz, 1H), 2.40 – 2.06 (m, 5H), 2.00 (m, *J* = 14.9, 5.2, 2.7 Hz, 1H), 1.89 – 1.82 (m, 2H), 1.71 – 1.59 (m, 5H), 1.51 (m, *J* = 14.1, 8.7, 4.0 Hz, 5H), 1.27 – 1.21 (m, 2H), 1.17 – 1.07 (m, 2H), 1.02 (s, 4H), 0.71 (s, 3H).

**$^{13}$C NMR** (126 MHz, Chloroform-*d*) δ 208.2, 150.2 (d, *J* = 266.0 Hz), 140.8, 130.5, 125.5 (d, *J* = 5.9 Hz), 121.4, 121.2 (d, *J* = 3.6 Hz), 120.4, 118.7 (d, *J* = 6.4 Hz), 108.8 (d, *J* = 1.8 Hz), 84.2 (d, *J*

= 11.1 Hz), 71.7, 61.3, 57.0, 50.1, 44.4, 42.3, 38.9, 38.2 (d, *J* = 3.1 Hz), 37.3, 36.6, 28.0 (d, *J* = 2.1 Hz), 24.6, 23.5, 19.4, 13.5.

**$^{19}$F NMR** (471 MHz, Chloroform-*d*) δ -139.31.

**HRMS** (ESI, m/z) calcd for $C_{29}H_{36}FNO_2[M+H]^+$: 450.2803; found: 450.2804.

## 1-((1r,3R,5S,7s)-adamantan-1-yl)-2-(2-fluoro-1-methyl-1H-indol-3-yl)ethan-1-one (11e)

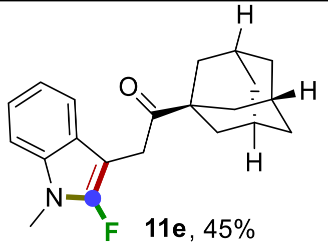

**11e**, 45%

The reaction was performed following the general procedure. The residue was purified by flash column chromatography (silica gel, petroleum ether: $CH_2Cl_2$ =10:1, v/v) to give the product as a yellow solid (29.3 mg, 45%). m.p. 109-111 °C.

**$^1$H NMR** (500 MHz, Chloroform-*d*) δ 7.39 (d, *J* = 7.7 Hz, 1H), 7.21 (m, *J* = 3.9, 1.4 Hz, 2H), 7.14 (m, *J* = 8.1, 5.3, 3.1 Hz, 1H), 3.82 (s, 2H), 3.63 (s, 3H), 2.12 (s, 3H), 1.98 (s, 6H), 1.83 – 1.73 (m, 6H).

**$^{13}$C NMR** (126 MHz, Chloroform-*d*) δ 211.8, 1503 (d, *J* = 265.5 Hz), 130.6, 125.6 (d, *J* = 6.3 Hz), 121.0 (d, *J* = 3.6 Hz), 118.7 (d, *J* = 6.3 Hz), 108.8 (d, *J* = 1.8 Hz), 84.3 (d, *J* = 10.9 Hz), 46.7, 38.4, 36.6, 29.9 (d, *J* = 3.1 Hz), 28.1, 27.9 (d, *J* = 1.9 Hz).

**$^{19}$F NMR** (471 MHz, Chloroform-*d*) δ -139.58.

**HRMS** (ESI, m/z) calcd for $C_{21}H_{24}FNO[M+H]^+$: 326.1915; found: 326.1913.

## (8S,9S,10R,14S,17R)-17-(2-(2-fluoro-1-methyl-1H-indol-3-yl)acetyl)-10-methyl-1,2,6,7,8,9,10,11,12,13,14,15,16,17-tetradecahydro-3H-cyclopenta[a]phenanthren-3-one (11f)

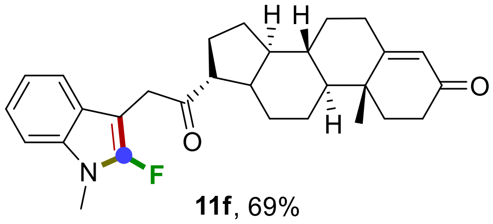

**11f**, 69%

The reaction was performed following the general procedure. The residue was purified by flash column chromatography (silica gel, petroleum ether: $CH_2Cl_2$ =10:1, v/v) to give the product as a yellow oil (61.7 mg, 69%).

**$^1$H NMR** (500 MHz, Chloroform-*d*) δ 7.38 (d, *J* = 7.8 Hz, 1H), 7.20 (m, *J* = 5.3, 1.7 Hz, 2H), 7.15 – 7.12 (m, 1H), 5.74 (d, *J* = 1.7 Hz, 1H), 3.70 (d, *J* = 7.6 Hz, 1H), 3.64 (s, 3H), 2.71 (t, *J* = 9.0 Hz, 1H), 2.46 – 2.35 (m, 3H), 2.35 – 2.24 (m, 4H), 2.22 – 2.12 (m, 2H), 2.04 (m, *J* = 13.4, 5.1, 3.2 Hz,

1H), 1.85 (m, $J$ = 12.9, 5.6, 2.8 Hz, 1H), 1.69 – 1.65 (m, 2H), 1.51 – 1.45 (m, 2H), 1.23 – 1.12 (m, 6H), 1.08 – 1.02 (m, 1H), 0.99 (m, $J$ = 11.8, 3.9 Hz, 1H), 0.90 (dd, $J$ = 14.9, 7.7 Hz, 1H), 0.74 (s, 3H).

**$^{13}$C NMR** (126 MHz, Chloroform-*d*) δ 208.0, 199.5, 171.1, 150.2 (d, $J$ = 265.7 Hz), 130.5, 129.6, 125.8, 125.5, 123.9, 121.2, 121.2, 120.4, 118.7, 118.6, 108.9, 84.1, 84.0, 61.1, 56.1, 53.7, 44.7, 38.7, 38.61, 38.23, 38.20, 35.75, 35.59, 33.97, 32.8, 32.0, 28.0, 28.0, 24.5, 23.9, 23.5, 21.1, 19.7, 17.4, 13.6.

**$^{19}$F NMR** (471 MHz, Chloroform-*d*) δ -139.33.

**HRMS** (ESI, m/z) calcd for $C_{29}H_{34}FNO_2[M+H]^+$: 448.2646; found: 448.2649.

## (E)-3,7-dimethylocta-2,6-dien-1-yl 2-(2-fluoro-1-methyl-1H-indol-3-yl)acetate (11g)

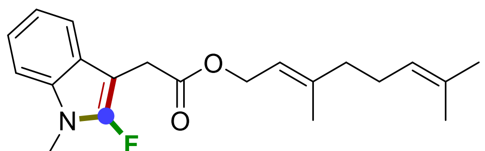


**11g**, 48%

The reaction was performed following the general procedure. The residue was purified by flash column chromatography (silica gel, petroleum ether: $CH_2Cl_2$ =10:1, v/v) to give the product as a yellow oil (32.9 mg, 48%).

**$^{1}$H NMR** (500 MHz, Chloroform-*d*) δ 7.50 (d, $J$ = 7.8 Hz, 1H), 7.22 – 7.18 (m, 2H), 7.14 (m, $J$ = 8.1, 5.4, 2.9 Hz, 1H), 5.35 (m, $J$ = 7.1, 1.3 Hz, 1H), 5.13 – 5.03 (m, 1H), 4.63 (d, $J$ = 7.1 Hz, 2H), 3.69 (s, 2H), 3.64 (s, 3H), 2.12 – 2.01 (m, 4H), 1.68 (m, $J$ = 2.9, 1.4 Hz, 6H), 1.61 (d, $J$ = 1.3 Hz, 3H).

**$^{13}$C NMR** (126 MHz, Chloroform-*d*) δ 171.3, 150.2 (d, $J$ = 266.5 Hz), 142.3, 131.8, 130.5, 125.3 (d, $J$ = 5.8 Hz), 123.8, 121.2 (d, $J$ = 3.5 Hz), 120.3, 118.7 (d, $J$ = 6.5 Hz), 118.3, 108.8 (d, $J$ = 1.8 Hz), 83.9 (d, $J$ = 10.6 Hz), 61.9, 39.5, 28.3 (d, $J$ = 2.5 Hz), 27.9 (d, $J$ = 2.0 Hz), 26.3, 25.7, 17.7, 16.5.

**$^{19}$F NMR** (471 MHz, Chloroform-*d*) δ -139.43.

**HRMS** (ESI, m/z) calcd for $C_{21}H_{26}FNO_2[M+H]^+$: 344.2020; found: 344.2019.

## 2-((1R,5S)-6,6-dimethylbicyclo[3.1.1]hept-2-en-3-yl)ethyl 2-(2-fluoro-1-methyl-1H-indol-3-yl)acetate (11h)

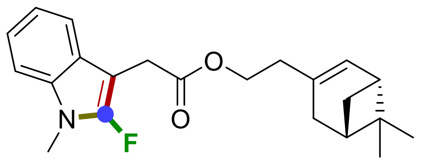


**11h**, 72%

The reaction was performed following the general procedure. The residue was purified by flash column chromatography (silica gel, petroleum ether: $CH_2Cl_2$ =10:1, v/v) to give the product as a yellow oil (51.1 mg, 72%).

**$^1$H NMR** (500 MHz, Chloroform-*d*) δ 7.54 (d, *J* = 7.7 Hz, 1H), 7.24 – 7.20 (m, 2H), 7.18 (m, *J* = 8.1, 5.3, 3.0 Hz, 1H), 5.24 – 5.20 (m, 1H), 4.20 – 4.08 (m, 2H), 3.69 (s, 2H), 3.64 (s, 3H), 2.37 – 2.27 (m, 3H), 2.25 – 2.12 (m, 2H), 2.10 – 2.02 (m, 2H), 1.28 (s, 3H), 1.12 (d, *J* = 8.5 Hz, 1H), 0.80 (s, 3H).

**$^{13}$C NMR** (126 MHz, Chloroform-*d*) δ 171.3, 150.2 (d, *J* = 266.6 Hz), 144.0, 130.5, 125.4 (d, *J* = 5.5 Hz), 121.2 (d, *J* = 3.5 Hz), 120.4, 118.9, 118.7 (d, *J* = 6.4 Hz), 108.9, 83.9 (d, *J* = 11.0 Hz), 63.1, 45.6, 40.7, 38.0, 36.0, 31.6, 31.4, 28.3 (d, *J* = 2.3 Hz), 27.9 (d, *J* = 2.0 Hz), 26.3, 21.1.

**$^{19}$F NMR** (471 MHz, Chloroform-*d*) δ -139.57.

**HRMS** (ESI, m/z) calcd for $C_{22}H_{26}FNO_2$[M+H]$^+$: 356.2020; found: 356.2018.

## (1R,2S,5R)-2-isopropyl-5-methylcyclohexyl 2-(2-fluoro-1-methyl-1H-indol-3-yl)acetate (11i)

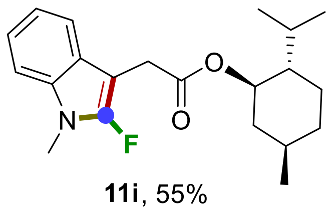


**11i**, 55%

The reaction was performed following the general procedure. The residue was purified by flash column chromatography (silica gel, petroleum ether: $CH_2Cl_2$ =10:1, v/v) to give the product as a yellow oil (38.0 mg, 55%).

**$^1$H NMR** (500 MHz, Chloroform-*d*) δ 7.51 – 7.47 (m, 1H), 7.22 – 7.19 (m, 2H), 7.14 (m, *J* = 8.1, 5.3, 3.0 Hz, 1H), 4.69 (m, *J* = 10.9, 4.4 Hz, 1H), 3.66 (d, *J* = 2.4 Hz, 2H), 3.64 (s, 3H), 2.03 – 1.98 (m, 1H), 1.77 (m, *J* = 7.0, 2.7 Hz, 1H), 1.69 – 1.63 (m, 2H), 1.36 (m, *J* = 14.4, 10.9, 3.2 Hz, 2H), 1.09 – 0.98 (m, 2H), 0.89 (d, *J* = 6.6 Hz, 3H), 0.82 (d, *J* = 7.0 Hz, 3H), 0.69 (d, *J* = 6.9 Hz, 3H).

**$^{13}$C NMR** (126 MHz, Chloroform-*d*) δ 170.9, 150.2 (d, *J* = 266.5 Hz), 130.5, 125.3 (d, *J* = 5.7 Hz), 121.1 (d, *J* = 3.6 Hz), 120.2, 118.7 (d, *J* = 6.4 Hz), 108.7 (d, *J* = 2.0 Hz), 84.1 (d, *J* = 11.1 Hz), 74.8, 47.1, 40.8, 34.3, 31.4, 28.6 (d, *J* = 2.4 Hz), 27.9 (d, *J* = 2.1 Hz), 26.2, 23.5, 22.0, 20.7, 16.3.

**$^{19}$F NMR** (471 MHz, Chloroform-*d*) δ -139.62.

**HRMS** (ESI, m/z) calcd for $C_{21}H_{26}FNO_2$[M+H]$^+$: 346.2177; found: 346.2174.

## (R)-3,7-dimethyloct-6-en-1-yl 2-(2-fluoro-1-methyl-1H-indol-3-yl)acetate (11j)

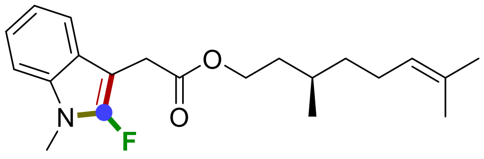


**11j**, 66%

The reaction was performed following the general procedure. The residue was purified by flash column chromatography (silica gel, petroleum ether: $CH_2Cl_2$ =10:1, v/v) to give the product as a yellow oil (45.6 mg, 66%).

**$^1$H NMR** (500 MHz, Chloroform-*d*) δ 7.54 – 7.50 (m, 1H), 7.24 – 7.20 (m, 2H), 7.16 (m, *J* = 8.1, 4.1 Hz, 1H), 5.09 (m, *J* = 7.1, 2.9, 1.4 Hz, 1H), 4.20 – 4.12 (m, 2H), 3.69 (s, 2H), 3.64 (s, 3H), 1.97 (m, *J* = 27.5, 7.7 Hz, 2H), 1.71 (s, 3H), 1.62 (s, 3H), 1.55 – 1.43 (m, 2H), 1.37 – 1.28 (m, 2H), 1.21 – 1.13 (m, 1H), 0.90 (d, *J* = 6.5 Hz, 3H).

**$^{13}$C NMR** (126 MHz, Chloroform-*d*) δ 171.4 (d, *J* = 1.9 Hz), 150.2 (d, *J* = 266.5 Hz), 131.3, 130.5, 125.3 (d, *J* = 5.6 Hz), 124.6, 121.2 (d, *J* = 3.5 Hz), 120.3, 118.7 (d, *J* = 6.4 Hz), 108.8 (d, *J* = 1.8 Hz), 83.9 (d, *J* = 10.8 Hz), 63.5, 37.0, 35.5, 29.5, 28.3 (d, *J* = 2.4 Hz), 27.9 (d, *J* = 2.1 Hz), 25.8, 25.4, 19.4, 17.7.

**$^{19}$F NMR** (471 MHz, Chloroform-*d*) δ -139.49.

**HRMS** (ESI, m/z) calcd for $C_{21}H_{28}FNO_2[M+H]^+$: 346.2177; found: 346.2181.

## (3aS,5R,6R,6aS)-5-((S)-2,2-dimethyl-1,3-dioxolan-4-yl)-2,2-dimethyltetrahydrofuro[2,3-d][1,3]dioxol-6-yl 2-(2-fluoro-1-methyl-1H-indol-3-yl)acetate (11k)

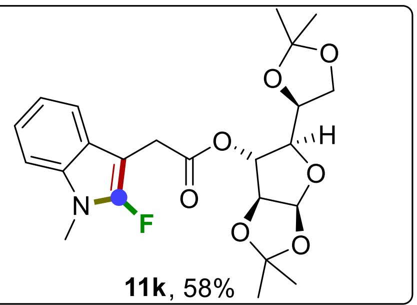


**11k**, 58%

The reaction was performed following the general procedure. The residue was purified by flash column chromatography (silica gel, petroleum ether: $CH_2Cl_2$ =10:1, v/v) to give the product as a yellow oil (52.1 mg, 58%).

**$^1$H NMR** (500 MHz, Chloroform-*d*) δ 7.49 (m, *J* = 7.9, 1.0 Hz, 1H), 7.23 – 7.20 (m, 2H), 7.14 (m, *J* = 8.1, 4.9, 3.4 Hz, 1H), 5.80 (d, *J* = 3.6 Hz, 1H), 5.29 (d, *J* = 3.0 Hz, 1H), 4.44 (d, *J* = 3.6 Hz, 1H), 4.26 (m, *J* = 8.9, 7.2 Hz, 1H), 4.20 (m, *J* = 7.9, 3.0 Hz, 1H), 4.13 (m, *J* = 7.9, 5.5 Hz, 1H), 3.96 (m, *J* = 5.5, 2.0 Hz, 2H), 3.72 (s, 2H), 3.64 (s, 3H), 1.50 (s, 3H), 1.38 (s, 3H), 1.27 (s, 3H), 1.24 (s, 3H).

**$^{13}$C NMR** (126 MHz, Chloroform-*d*) δ 169.9, 150.2 (d, *J* = 266.9 Hz), 130.5, 125.0 (d, *J* = 5.5 Hz), 121.4 (d, *J* = 3.5 Hz), 120.4, 118.5 (d, *J* = 6.3 Hz), 112.3, 109.3, 108.9, 105.1, 83.3, 79.9, 76.5, 72.4, 67.12, 65.7 (d, *J* = 5.6 Hz), 28.2 (d, *J* = 2.4 Hz), 28.0 (d, *J* = 2.1 Hz), 26.8, 26.8, 26.21, 25.0.

**$^{19}$F NMR** (471 MHz, Chloroform-*d*) δ -139.49.

**HRMS** (ESI, m/z) calcd for $C_{23}H_{28}FNO_7[M+H]^+$: 450.1923; found: 450.1916.

## (3S,8S,9S,10R,13R,14S,17R)-10,13-dimethyl-17-((R)-6-methylheptan-2-yl)-2,3,4,7,8,9,10,11,12,13,14,15,16,17-tetradecahydro-1H-cyclopenta[a]phenanthren-3-yl 2-(2-fluoro-1-methyl-1H-indol-3-yl)acetate (11l)

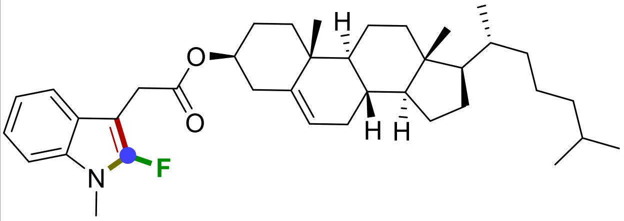

**11l**, 55%

The reaction was performed following the general procedure. The residue was purified by flash column chromatography (silica gel, petroleum ether: $CH_2Cl_2$ =10:1, v/v) to give the product as a yellow solid (63.3 mg, 55%). m.p. 149-151 ºC.

**$^{1}$H NMR** (500 MHz, Chloroform-*d*) δ 7.54 (d, *J* = 7.7 Hz, 1H), 7.22 (d, *J* = 3.5 Hz, 2H), 7.17 (m, *J* = 8.1, 4.3 Hz, 1H), 5.37 (m, *J* = 3.5, 1.7 Hz, 1H), 4.66 (m, *J* = 10.8, 6.5, 4.3 Hz, 1H), 3.68 (s, 2H), 3.64 (s, 3H), 2.39 – 2.32 (m, 2H), 2.00 (m, *J* = 24.6, 13.9, 2.8 Hz, 2H), 1.87 (m, *J* = 12.6, 8.7, 4.5 Hz, 3H), 1.65 – 1.44 (m, 7H), 1.42 – 1.33 (m, 3H), 1.32 – 1.09 (m, 8H), 1.03 (s, 5H), 0.94 (d, *J* = 6.6 Hz, 4H), 0.89 (m, *J* = 6.6, 2.3 Hz, 6H), 0.70 (s, 3H).

**$^{13}$C NMR** (126 MHz, Chloroform-*d*) δ 170.7, 150.2 (d, *J* = 266.5 Hz), 139.7, 130.5, 125.3 (d, *J* = 5.6 Hz), 122.7, 121.2 (d, *J* = 3.5 Hz), 120.3, 118.8 (d, *J* = 6.3 Hz), 108.8, 84.0 (d, *J* = 10.6 Hz), 74.6, 56.7, 56.2, 50.0, 42.3, 39.8, 39.6, 38.1, 37.0, 36.6, 36.2, 35.9, 31.9 (d, *J* = 6.5 Hz), 28.7 (d, *J* = 2.4 Hz), 28.3, 28.1, 27.9, 27.8, 24.3, 23.9, 22.9, 22.6, 21.1, 19.4, 18.8, 11.9.

**$^{19}$F NMR** (471 MHz, Chloroform-*d*) δ -139.44.

**HRMS** (ESI, m/z) calcd for $C_{38}H_{54}FNO_2[M+K]^+$: 614.3770; found: 614.3780.

## 4-(2-(2-fluoro-1-methyl-1H-indol-3-yl)acetyl)phenyl (R)-2-(4-isobutylphenyl)propanoate (11m)

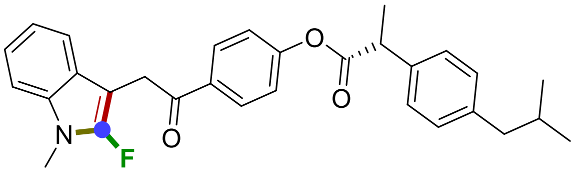

**11m**, 85%

The reaction was performed following the general procedure. The residue was purified by flash column chromatography (silica gel, petroleum ether: $CH_2Cl_2$ =10:1, v/v) to give the product as a yellow oil (80.1 mg, 85%).

**$^{1}$H NMR** (500 MHz, Chloroform-*d*) δ 8.13 – 8.06 (m, 2H), 7.52 (dt, *J* = 7.8, 1.0 Hz, 1H), 7.34 (d, *J* = 8.2 Hz, 2H), 7.24 – 7.16 (m, 5H), 7.13 (d, *J* = 8.7 Hz, 2H), 4.29 (s, 2H), 3.99 (q, *J* = 7.1 Hz, 1H), 3.62 (s, 3H), 2.52 (d, *J* = 7.2 Hz, 2H), 1.92 (dt, *J* = 13.5, 6.8 Hz, 1H), 1.65 (d, *J* = 7.2 Hz, 3H), 0.97 (d, *J* = 6.6 Hz, 6H).

**$^{13}$C NMR** (126 MHz, Chloroform-*d*) δ 195.4, 172.7, 154.6, 150.0 (d, *J* = 265.8 Hz), 141.1, 137.0, 133.8, 130.6, 130.1, 129.7, 127.3, 125.3 (d, *J* = 5.8 Hz), 121.7, 121.3 (d, *J* = 3.5 Hz), 120.5, 118.8 (d, *J* = 6.2 Hz), 113.8, 108.9 (d, *J* = 1.8 Hz), 83.9 (d, *J* = 10.6 Hz), 45.4, 45.1, 32.9 (d, *J* = 2.7 Hz), 30.3, 27.9 (d, *J* = 1.9 Hz), 22.5, 18.5.

**$^{19}$F NMR** (471 MHz, Chloroform-*d*) δ -138.78.
**HRMS** (ESI, m/z) calcd for $C_{30}H_{30}FNO_3[M+K]^+$: 510.1841; found: 510.1840.

## 4-(2-(2-fluoro-1-methyl-1H-indol-3-yl)acetyl)phenyl (R)-2-(6-methoxynaphthalen-2-yl)propanoate (11n)

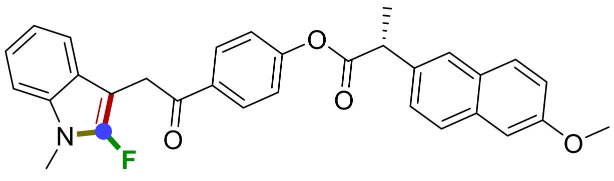

**11n**, 82%

The reaction was performed following the general procedure. The residue was purified by flash column chromatography (silica gel, petroleum ether: $CH_2Cl_2$ =10:1, v/v) to give the product as a yellow oil (81.2 mg, 82%).
**$^1$H NMR** (500 MHz, Chloroform-*d*) δ 8.09 (d, *J* = 8.3 Hz, 2H), 7.84 – 7.78 (m, 3H), 7.57 – 7.51 (m, 2H), 7.26 – 7.17 (m, 5H), 7.13 (d, *J* = 8.3 Hz, 2H), 4.30 (s, 2H), 4.16 (q, *J* = 7.1 Hz, 1H), 3.96 (s, 3H), 3.63 (s, 3H), 1.76 (d, *J* = 7.2 Hz, 3H).
**$^{13}$C NMR** (126 MHz, Chloroform-*d*) δ 195.4, 172.7, 157.9, 154.6, 150.0 (d, *J* = 265.9 Hz), 134.810, 134.9, 133.9, 130.6, 130.1, 129.4, 129.0, 127.8, 126.3, 126.1, 125.3 (d, *J* = 5.7 Hz), 121.7, 121.3 (d, *J* = 3.5 Hz), 120.5, 119.3, 118.8 (d, *J* = 6.3 Hz), 108.9, 105.7, 83.9 (d, *J* = 10.6 Hz), 55.4, 45.7, 32.9 (d, *J* = 2.8 Hz), 28.0 18.5.
**$^{19}$F NMR** (471 MHz, Chloroform-*d*) δ -138.72.
**HRMS** (ESI, m/z) calcd for $C_{31}H_{26}FNO_4[M+H]^+$: 496.1919; found: 496.1917.

## 4-(2-(2-fluoro-1-methyl-1H-indol-3-yl)acetyl)phenyl 4-(N,N-dipropylsulfamoyl)benzoate (11o)

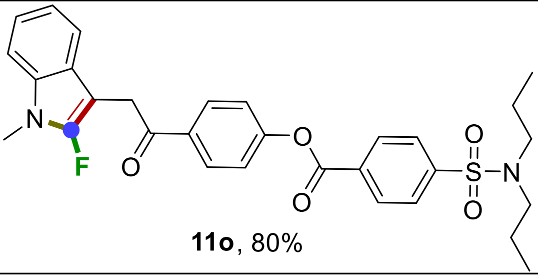

**11o**, 80%

The reaction was performed following the general procedure. The residue was purified by flash column chromatography (silica gel, petroleum ether: $CH_2Cl_2$ =10:1, v/v) to give the product as a yellow oil (88.0 mg, 80%).
**$^1$H NMR** (500 MHz, Chloroform-*d*) δ 8.34 (d, *J* = 8.4 Hz, 2H), 8.21 – 8.17 (m, 2H), 8.00 – 7.96 (m, 2H), 7.52 (d, *J* = 7.8 Hz, 1H), 7.37 – 7.33 (m, 2H), 7.23 (M, *J* = 4.5, 1.9 Hz, 2H), 7.19 – 7.15 (m, 1H), 4.34 (s, 2H), 3.67 (s, 3H), 3.18 – 3.14 (m, 4H), 1.59 (q, *J* = 7.5 Hz, 4H), 0.90 (d, *J* = 7.4 Hz, 6H).

**$^{13}$C NMR** (126 MHz, Chloroform-*d*) δ 195.3, 163.4, 154.2, 150.0 (d, $J$ = 265.8 Hz), 145.3, 134.3, 132.3, 130.9, 130.6, 130.3, 127.3, 125.3 (d, $J$ = 5.8 Hz), 121.8, 121.3 (d, $J$ = 3.4 Hz), 120.5, 118.77 (d, $J$ = 6.3 Hz), 108.9, 83.8 (d, $J$ = 10.9 Hz), 45.0, 33.0 (d, $J$ = 2.7 Hz), 28.0 (d, $J$ = 2.0 Hz), 22.0, 11.2.
**$^{19}$F NMR** (471 MHz, Chloroform-*d*) δ -138.77.
**HRMS** (ESI, m/z) calcd for $C_{30}H_{31}FN_2O_5S[M+H]^+$: 551.2010; found: 551.2017.

## 2-(5-chloro-2-fluoro-1-methyl-1H-indol-3-yl)-1-phenylethan-1-one (13a)

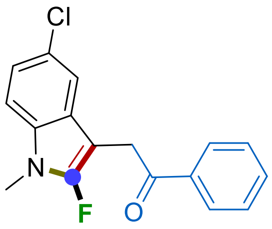

**13a**, 80%

The reaction was performed following the general procedure. The residue was purified by flash column chromatography (silica gel, petroleum ether: $CH_2Cl_2$ =10:1, v/v) to give the product as a yellow solid (48.1 mg, 80%). m.p. 150-152 °C.
**$^1$H NMR** (500 MHz, Chloroform-*d*) δ 8.07 (d, $J$ = 7.5 Hz, 2H), 7.61 – 7.55 (m, 1H), 7.53 – 7.43 (m, 3H), 7.16 – 7.06 (m, 2H), 4.28 (s, 2H), 3.60 (s, 3H).
**$^{13}$C NMR** (126 MHz, Chloroform-*d*) δ 196.2, 150.6 (d, $J$ = 267.3 Hz), 136.2, 133.3, 129.0, 128.7, 128.5, 126.5, 126.1, 121.5 (d, $J$ = 3.4 Hz), 118.5 (d, $J$ = 6.3 Hz), 110.1 (d, $J$ = 2.1 Hz), 84.2 (d, $J$ = 11.3 Hz), 32.6 (d, $J$ = 2.8 Hz), 28.1 (d, $J$ = 1.9 Hz).
**$^{19}$F NMR** (471 MHz, Chloroform-*d*) δ -136.30.
**HRMS** (ESI, m/z) calcd for $C_{17}H_{13}ClFNO[M+H]^+$: 302.0742; found: 302.0741.

## 2-(5-chloro-2-fluoro-1-methyl-1H-indol-3-yl)-1-(thiophen-2-yl)ethan-1-one (13b)

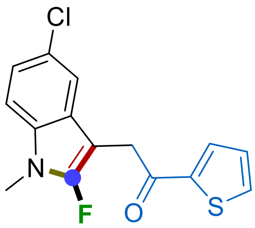

**13b**, 75%

The reaction was performed following the general procedure. The residue was purified by flash column chromatography (silica gel, petroleum ether: $CH_2Cl_2$ =10:1, v/v) to give the product as a yellow solid (46.1 mg, 75%). m.p. 139-141 °C.
**$^1$H NMR** (500 MHz, Chloroform-*d*) δ 7.85 (m, $J$ = 3.8, 1.2 Hz, 1H), 7.64 (m, $J$ = 5.0, 1.2 Hz, 1H), 7.49 (d, $J$ = 1.9 Hz, 1H), 7.16 – 7.09 (m, 3H), 4.19 (s, 2H), 3.62 (s, 3H).
**$^{13}$C NMR** (126 MHz, Chloroform-*d*) δ 189.2, 150.6 (d, $J$ = 267.3 Hz), 143.3, 134.0, 132.4, 128.9, 128.2, 126.4 (d, $J$ = 5.8 Hz), 126.3, 121.6 (d, $J$ = 3.6 Hz), 118.6 (d, $J$ = 6.3 Hz), 110.0 (d, $J$ = 2.0 Hz), 84.2 (d, $J$ = 11.5 Hz), 33.4 (d, $J$ = 2.8 Hz), 28.2 (d, $J$ = 2.2 Hz).

**$^{19}$F NMR** (471 MHz, Chloroform-*d*) δ -136.35.

**HRMS** (ESI, m/z) calcd for $C_{15}H_{11}ClFNOS[M+H]^+$: 308.0307; found: 308.0305.

## 1-(5-chloro-2-fluoro-1-methyl-1H-indol-3-yl)-3-methylpentan-2-one (13c)

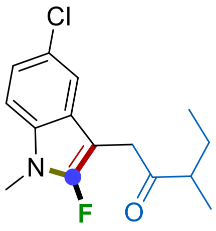

**13c**, 71%

The reaction was performed following the general procedure. The residue was purified by flash column chromatography (silica gel, petroleum ether: $CH_2Cl_2$ =10:1, v/v) to give the product as a yellow oil (39.9 mg, 71%).

**$^1$H NMR** (500 MHz, Chloroform-*d*) δ 7.35 (d, *J* = 1.9 Hz, 1H), 7.17 – 7.09 (m, 2H), 3.71 (s, 2H), 3.62 (s, 3H), 2.62 (q, *J* = 6.9 Hz, 1H), 1.74 (m, *J* = 14.5, 7.3 Hz, 1H), 1.50 – 1.39 (m, 1H), 1.11 (d, *J* = 6.9 Hz, 3H), 0.87 (t, *J* = 7.4 Hz, 3H).

**$^{13}$C NMR** (126 MHz, Chloroform-*d*) δ 211.0, 150.8 (d, *J* = 267.5 Hz), 128.9, 126.5 (d, *J* = 6.3 Hz), 126.1, 121.5 (d, *J* = 3.6 Hz), 118.2 (d, *J* = 6.4 Hz), 110.0 (d, *J* = 2.0 Hz), 84.2 (d, *J* = 11.6 Hz), 46.8, 35.1 (d, *J* = 3.0 Hz), 28.1 (d, *J* = 2.1 Hz), 26.1, 16.1, 11.7.

**$^{19}$F NMR** (471 MHz, Chloroform-*d*) δ -136.95.

**HRMS** (ESI, m/z) calcd for $C_{15}H_{17}ClFNO$ $[M+H]^+$: 282.1055; found: 282.1051.

## 2-(5-chloro-2-fluoro-1-methyl-1H-indol-3-yl)-1-cyclopropylethan-1-one (13d)

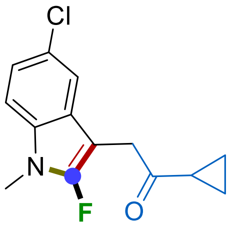

**13d**, 79%

The reaction was performed following the general procedure. The residue was purified by flash column chromatography (silica gel, petroleum ether: $CH_2Cl_2$ =10:1, v/v) to give the product as a yellow solid (41.9 mg, 79%). m.p. 129-131 °C.

**$^1$H NMR** (500 MHz, Chloroform-*d*) δ 7.38 (d, *J* = 1.9 Hz, 1H), 7.15 – 7.08 (m, 2H), 3.81 (s, 2H), 3.61 (s, 3H), 2.03 (m, *J* = 8.1, 4.5 Hz, 1H), 1.08 – 1.04 (m, 2H), 0.87 (m, *J* = 7.3, 3.7 Hz, 2H).

**$^{13}$C NMR** (126 MHz, Chloroform-*d*) δ 207.5, 150.8 (d, *J* = 267.7 Hz), 128.9, 126.5 (d, *J* = 6.3 Hz), 126.1, 121.5 (d, *J* = 3.5 Hz), 118.2 (d, *J* = 6.4 Hz), 110.1 (d, *J* = 1.9 Hz), 84.3 (d, *J* = 11.7 Hz), 37.1 (d, *J* = 2.7 Hz), 28.1 (d, *J* = 2.1 Hz), 19.6, 11.4 (d, *J* = 3.6 Hz).

**$^{19}$F NMR** (471 MHz, Chloroform-*d*) δ -136.84.

**HRMS** (ESI, m/z) calcd for $C_{14}H_{13}FNOCl[M+H]^+$: 266.0742; found: 266.0738.

## 2-(5-chloro-2-fluoro-1-methyl-1H-indol-3-yl)-1- ferroceneyl -1-one (13e)

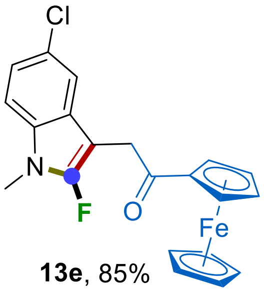


**13e**, 85%

The reaction was performed following the general procedure. The residue was purified by flash column chromatography (silica gel, petroleum ether: $CH_2Cl_2$ =10:1, v/v) to give the product as a yellow solid (69.5 mg, 85%). m.p. 125-127 $^oC$.

**$^1$H NMR** (500 MHz, Chloroform-*d*) δ 7.54 (d, *J* = 2.0 Hz, 1H), 7.17 – 7.06 (m, 2H), 4.88 (t, *J* = 2.0 Hz, 2H), 4.53 (t, *J* = 2.0 Hz, 2H), 4.18 (s, 5H), 4.00 (s, 2H), 3.62 (s, 3H).

**$^{13}$C NMR** (126 MHz, Chloroform-*d*) δ 200.5, 150.5 (d, *J* = 266.7 Hz), 128.9, 126.7 (d, *J* = 6.2 Hz), 126.1, 121.5 (d, *J* = 3.6 Hz), 118.8 (d, *J* = 6.4 Hz), 110.1, 85.1 (d, *J* = 11.4 Hz), 78.1, 72.5, 69.9, 69.6, 33.6 (d, *J* = 3.0 Hz), 28.2.

**$^{19}$F NMR** (471 MHz, Chloroform-*d*) δ -136.62.

**HRMS** (ESI, m/z) calcd for $C_{21}H_{17}ClFFeNO[M+H]^+$: 410.0405; found: 410.0409.

## 2-(5-bromo-1-ethyl-2-fluoro-1H-indol-3-yl)-1-phenylethan-1-one (16)

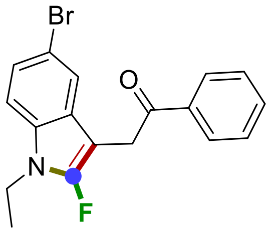


**16**, 35%

The reaction was performed following the general procedure. The residue was purified by flash column chromatography (silica gel, petroleum ether: $CH_2Cl_2$ =10:1, v/v) to give the product as a yellow solid (25.1 mg, 35%). m.p. 158-160 $^oC$.

**$^1$H NMR** (500 MHz, Chloroform-*d*) δ 8.14 – 8.06 (m, 2H), 7.66 – 7.56 (m, 2H), 7.51 (t, *J* = 7.7 Hz, 2H), 7.34 – 7.23 (m, 1H), 7.12 (d, *J* = 8.6 Hz, 1H), 4.31 (s, 2H), 4.10 (q, *J* = 7.3 Hz, 2H), 1.39 (t, *J* = 7.2 Hz, 3H).

**$^{13}$C NMR** (126 MHz, Chloroform-*d*) δ 196.2, 150.1 (d, *J* = 267.4 Hz), 136.3, 133.3, 128.7, 128.5, 128.2, 127.3 (d, *J* = 6.2 Hz), 124.0 (d, *J* = 3.6 Hz), 121.6 (d, *J* = 6.3 Hz), 113.5, 110.5 (d, *J* = 2.0 Hz), 84.2 (d, *J* = 11.3 Hz), 37.2, 32.6 (d, *J* = 2.8 Hz), 15.0.

**$^{19}$F NMR** (471 MHz, Chloroform-*d*) δ -136.46.

**HRMS** (ESI, m/z) calcd for $C_{18}H_{15}BrFNO[M+H]^+$: 360.0394; found: 360.0392.

## 2-(2-fluoro-1-methyl-1H-indol-3-yl)-1-phenylethan-1-one-2-d (3a-D)

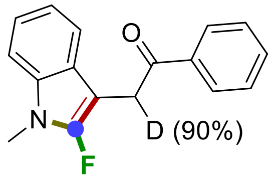


**3a-D**, 92%

The reaction was performed following the general procedure. The residue was purified by flash column chromatography (silica gel, petroleum ether: $CH_2Cl_2$ =10:1, v/v) to give the product as a yellow oil (49.3 mg, 92%).

**$^1$H NMR** (500 MHz, Chloroform-*d*) δ 8.09 (m, *J* = 8.4, 1.4 Hz, 2H), 7.59 – 7.54 (m, 1H), 7.52 (d, *J* = 7.8 Hz, 1H), 7.47 (m, *J* = 8.3, 7.0 Hz, 2H), 7.22 – 7.19 (m, 2H), 7.15 (m, *J* = 8.1, 4.7, 3.6 Hz, 1H), 4.30 (t, *J* = 2.2 Hz, 1.1H), 3.63 (s, 3H).

**$^{13}$C NMR** (126 MHz, Chloroform-*d*) δ 196.7, 150.0 (d, *J* = 266.1 Hz), 136.4, 133.1, 130.6, 128.6, 128.5, 125.4 (d, *J* = 6.0 Hz), 121.2 (d, *J* = 3.6 Hz), 120.4, 118.9 (d, *J* = 6.3 Hz), 108.8, 84.0, 32.9 (d, *J* = 3.0 Hz), 32.8 – 32.3 (m), 28.0.

**$^{19}$F NMR** (471 MHz, Chloroform-*d*) δ -138.89.

## 1-(difluoromethyl)-1H-benzo[d]imidazole (19) (CAS: 84941-15-1)[4]

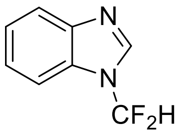


**19**, 78%

The reaction was performed following the general procedure. The residue was purified by flash column chromatography (silica gel, petroleum ether: $CH_2Cl_2$ =10:1, v/v) to give the product as a yellow oil (78.3 mg, 78%).

**$^1$H NMR** (500 MHz, Chloroform-*d*) δ 8.10 (s, 1H), 7.84 – 7.80 (m, 1H), 7.60 – 7.56 (m, 1H), 7.38 – 7.34 (m, 2H), 7.33 (s, 1H).

**$^{13}$C NMR** (126 MHz, Chloroform-*d*) δ 143.9, 139.2, 130.5, 124.8, 124.2, 120.9, 111.1, 109.0 (t, *J* = 249.7 Hz).

**$^{19}$F NMR** (471 MHz, Chloroform-*d*) δ -93.86.

## 2-phenylquinoline (22) (CAS: 612-96-4)[5]

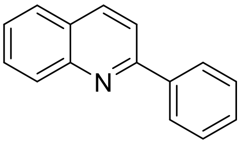


**22,** 86%

The reaction was performed following the general procedure. The residue was purified by flash column chromatography (silica gel, petroleum ether: $CH_2Cl_2$ =10:1, v/v) to give the product as a yellow oil (35.3 mg, 86%).

**$^1$H NMR** (500 MHz, Chloroform-*d*) δ 8.25 – 8.15 (m, 4H), 7.88 (d, *J* = 8.5 Hz, 1H), 7.83 (d, *J* = 8.1 Hz, 1H), 7.74 (t, *J* = 7.7 Hz, 1H), 7.59 – 7.51 (m, 3H), 7.48 (t, *J* = 7.3 Hz, 1H).

**$^{13}$C NMR** (126 MHz, Chloroform-*d*) δ 157.41, 148.32, 139.73, 136.81, 129.78, 129.70, 129.36, 128.89, 127.61, 127.50, 127.22, 126.32, 119.05.

## 2.5 NMR spectroscopic data

**3-(2-(dimethylamino)phenyl)-1-phenylprop-2-en-1-one (1a)**

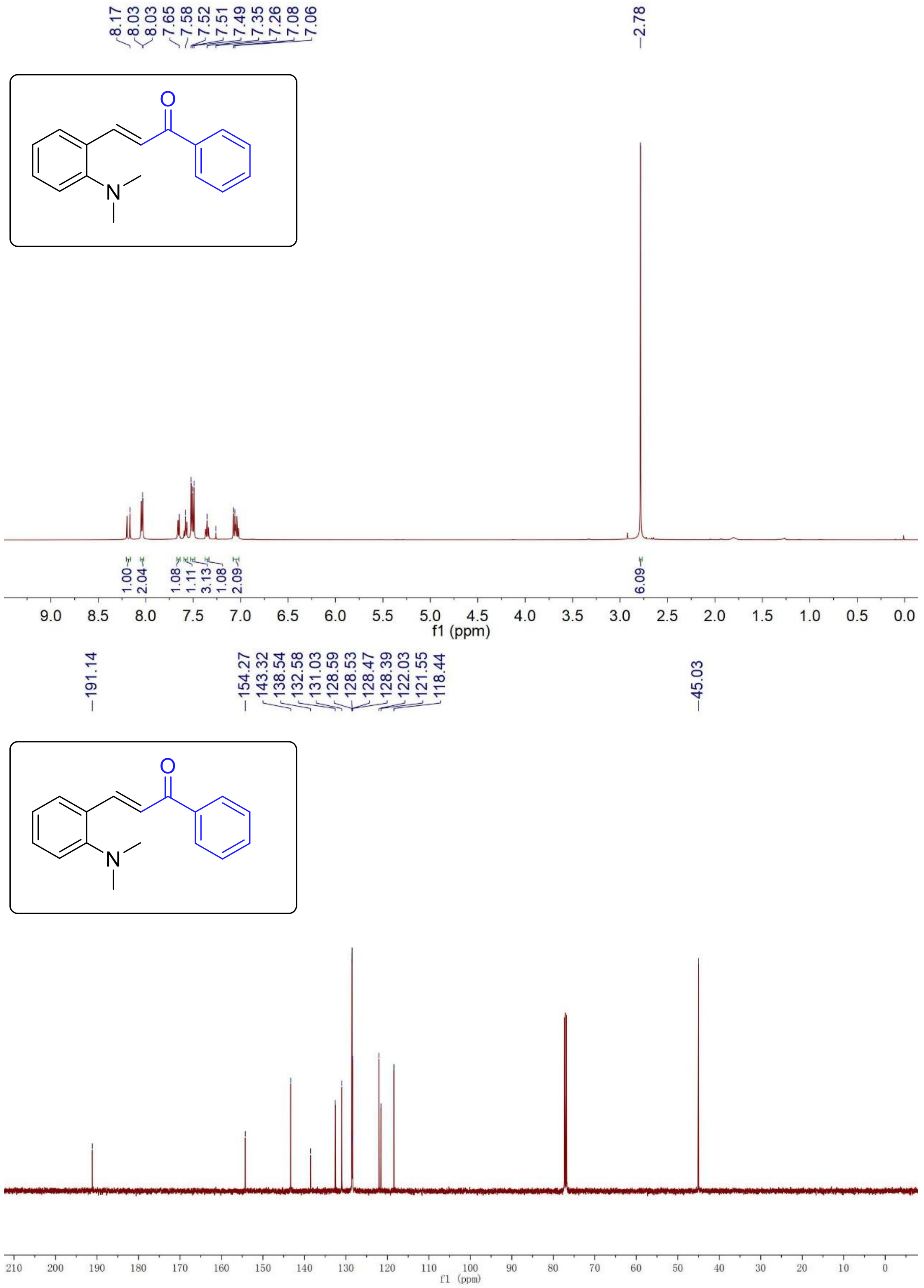


Supplementary Figure 1. $^{1}$H NMR and $^{13}$C NMR spectrum of **1a**.

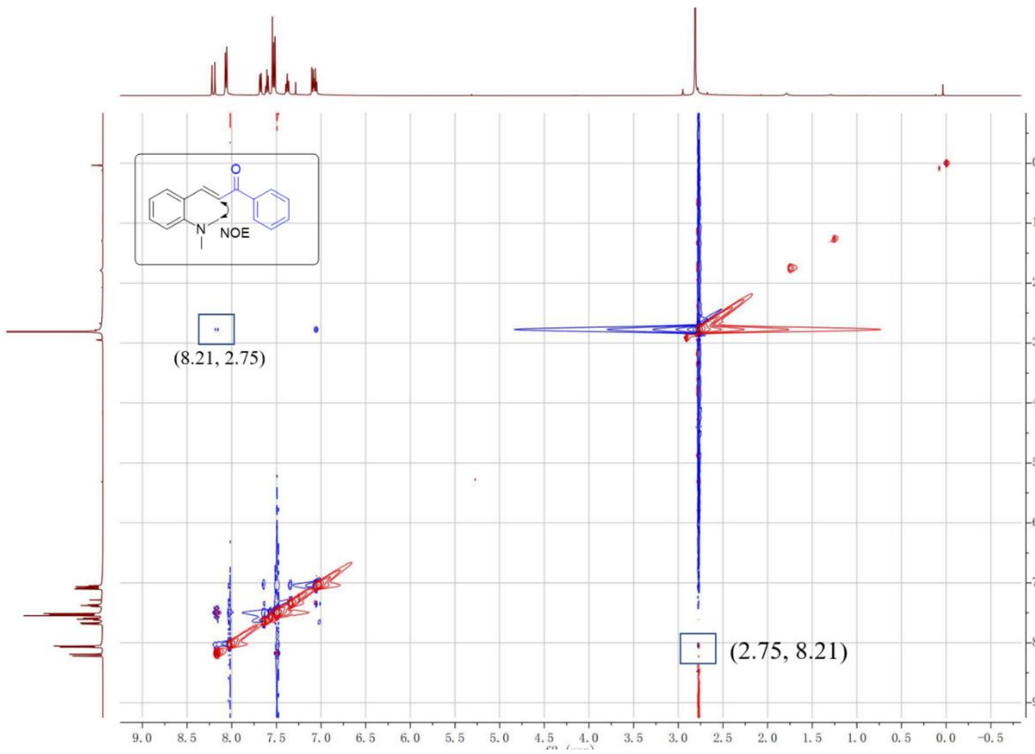


Supplementary Figure 2. $^1$H-$^1$HNOESYspectrum of **1a**.

**(E)-1-([1,1'-biphenyl]-4-yl)-3-(2-(dimethylamino)phenyl)prop-2-en-1-one (1b)**

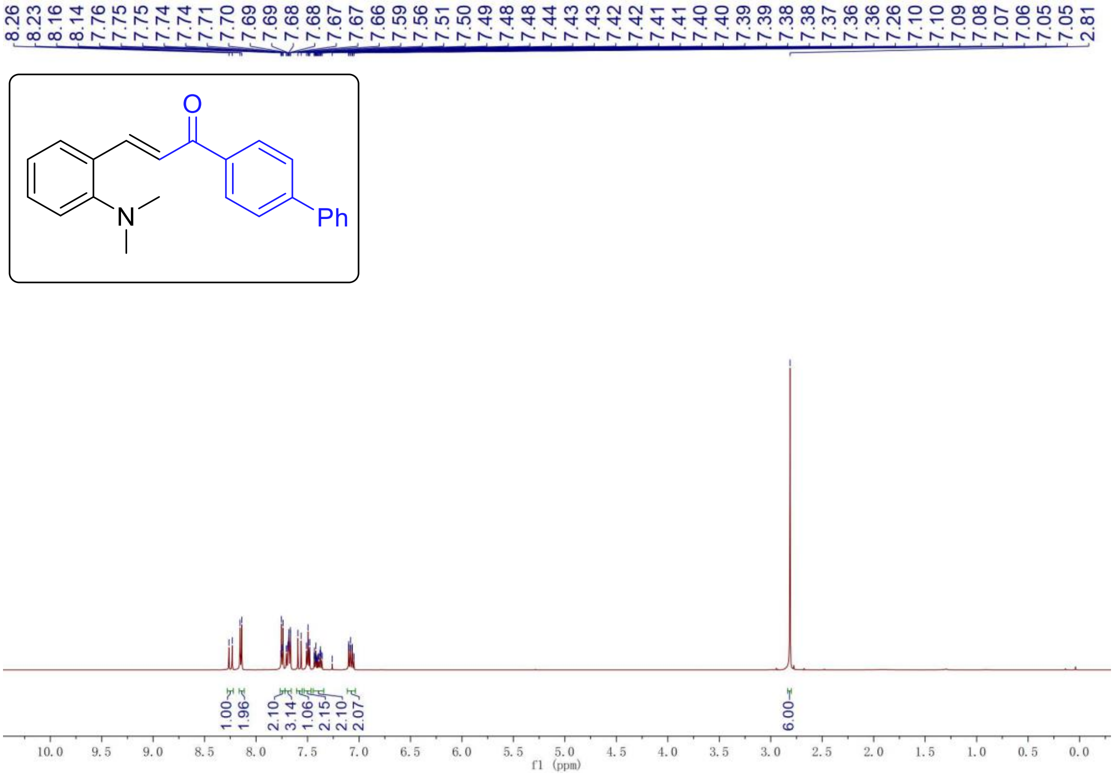

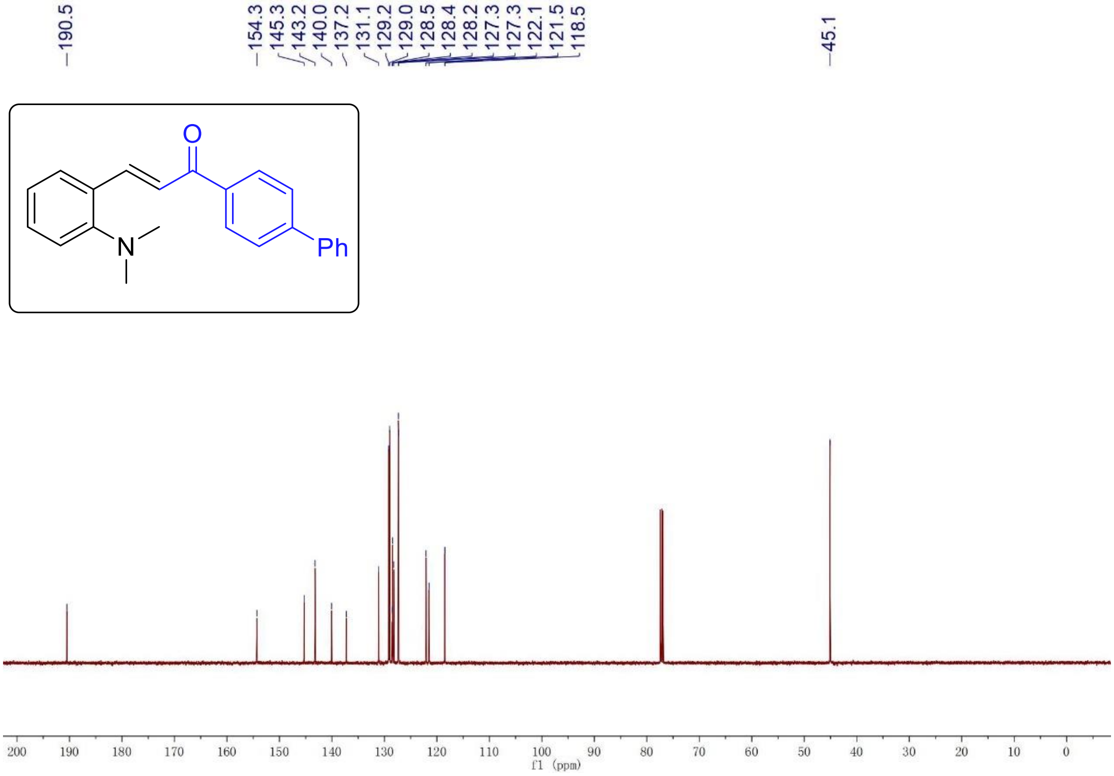

Supplementary Figure 3. $^{1}H$ NMR and $^{13}C$ NMR spectrum of **1b**.

## (E)-4-(3-(2-(dimethylamino)phenyl)acryloyl)benzoate (1c)

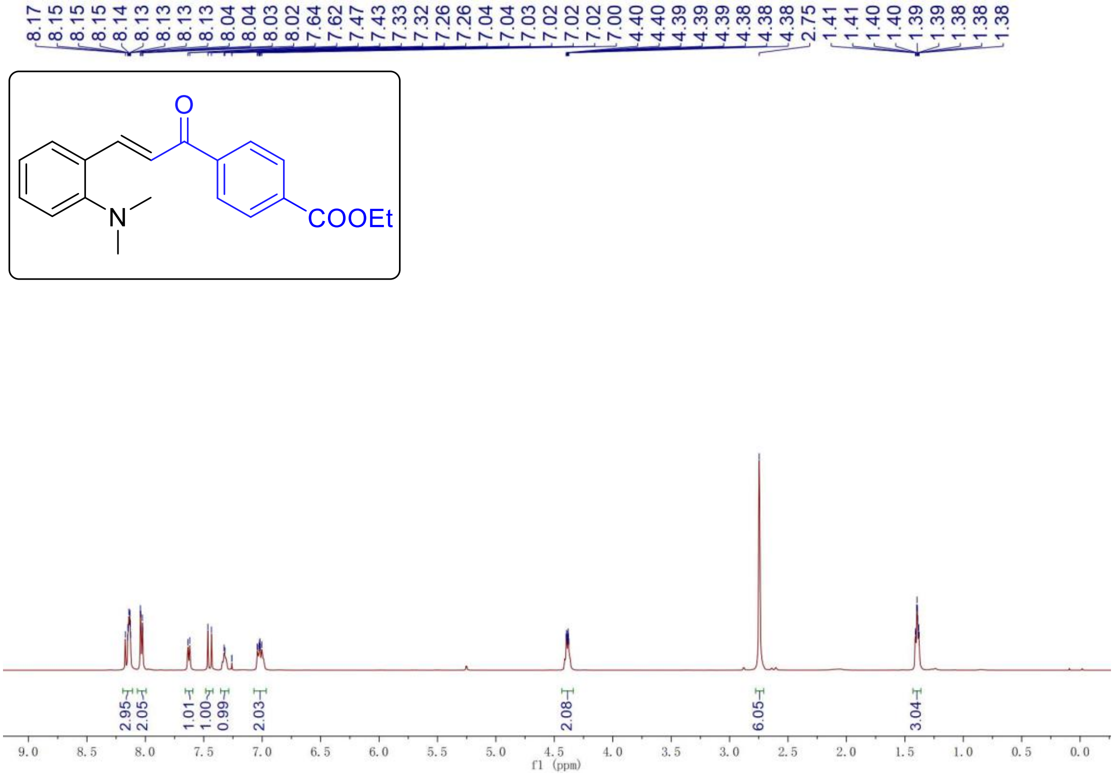


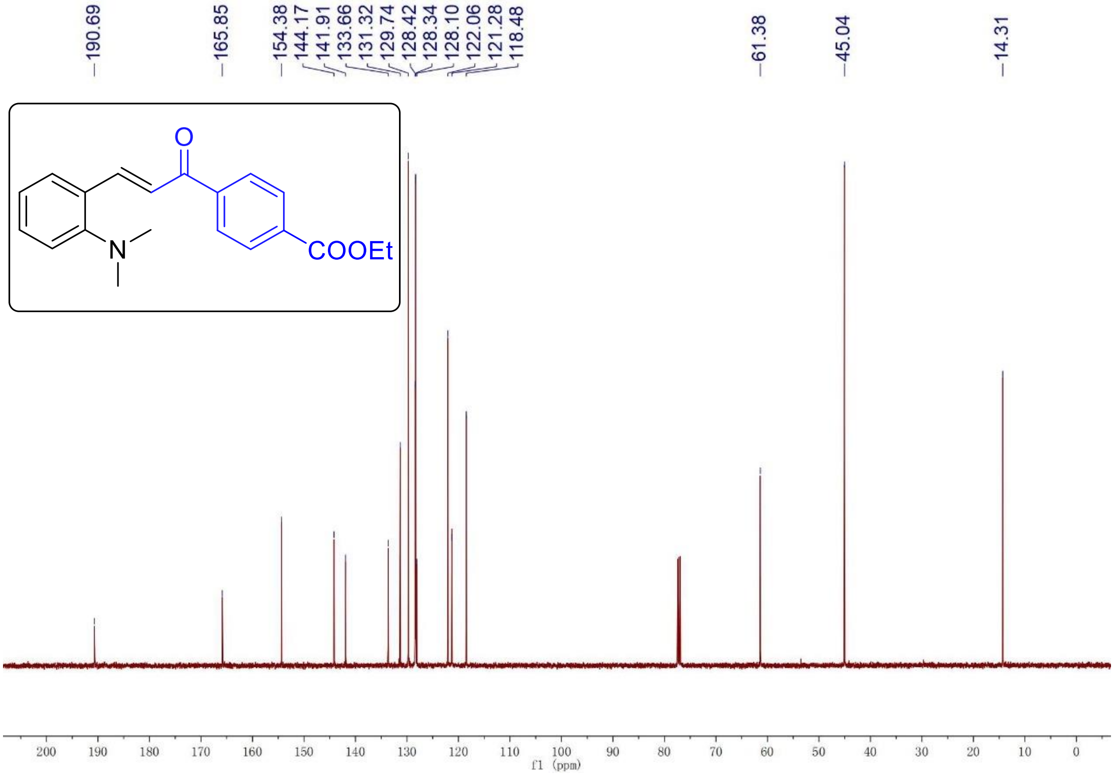


Supplementary Figure 4. $^{1}$H NMR and $^{13}$C NMR spectrum of **1c**.

**(E)-4-(3-(2-(dimethylamino)phenyl)acryloyl)benzonitrile (1d)**

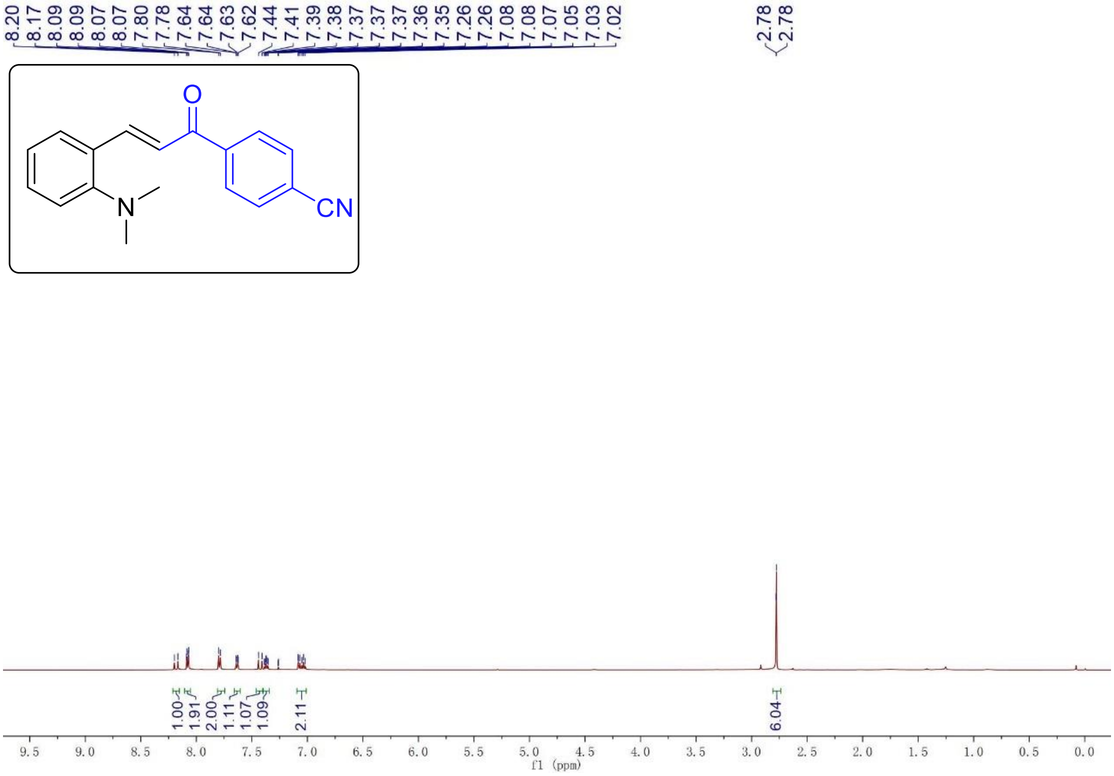


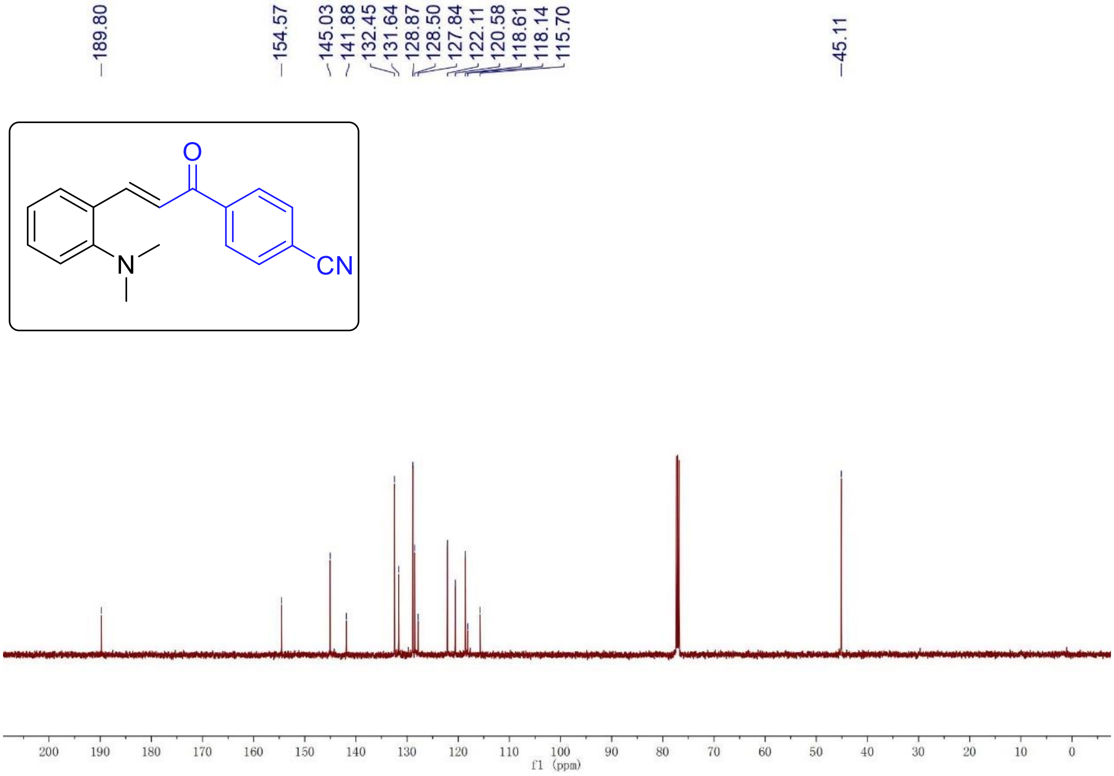


Supplementary Figure 5. $^{1}$H NMR and $^{13}$C NMR spectrum of **1d**.

**(E)-3-(2-(dimethylamino)phenyl)-1-(4-(methylthio)phenyl)prop-2-en-1-one (1e)**

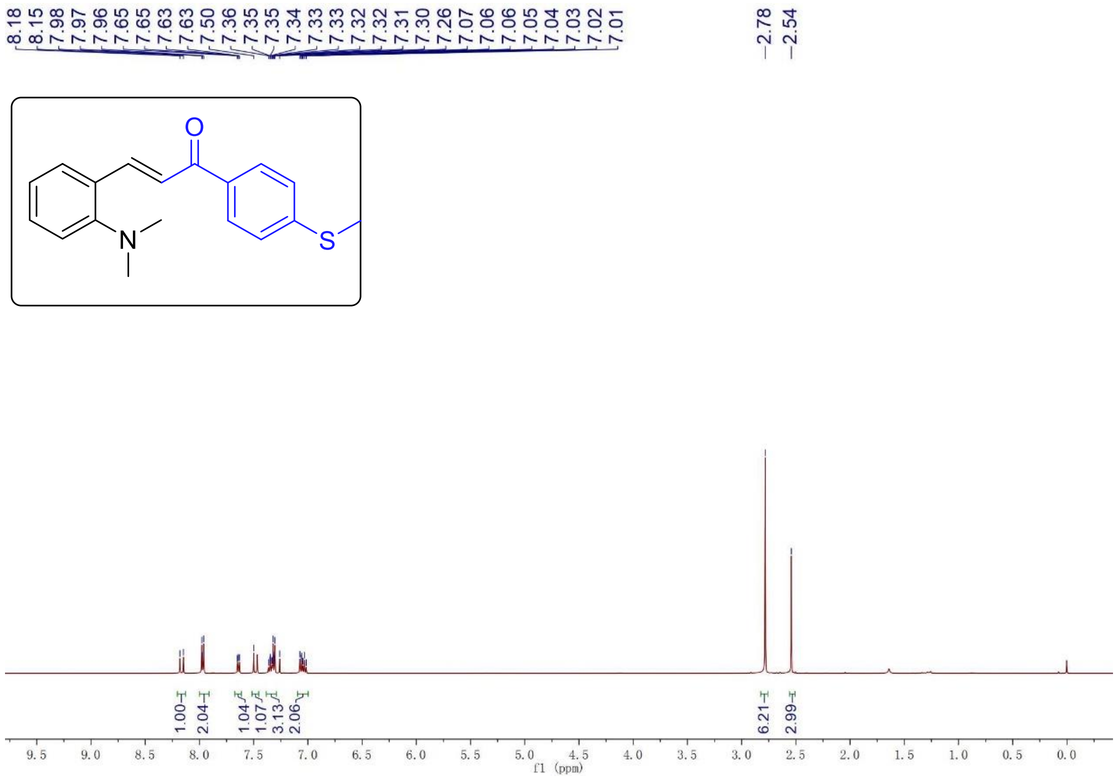


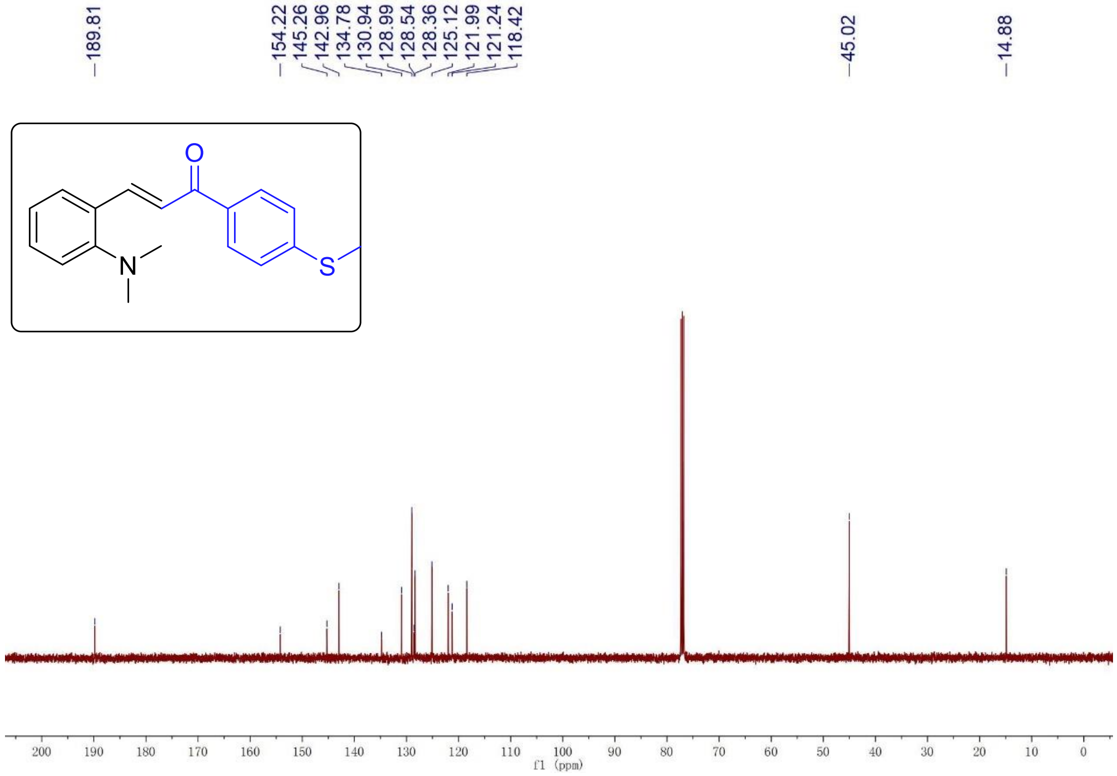


Supplementary Figure 6. $^{1}$H NMR and $^{13}$C NMR spectrum of **1e**.

**(E)-1-(4-(diethylamino)phenyl)-3-(2-(dimethylamino)phenyl)prop-2-en-1-one (1f)**

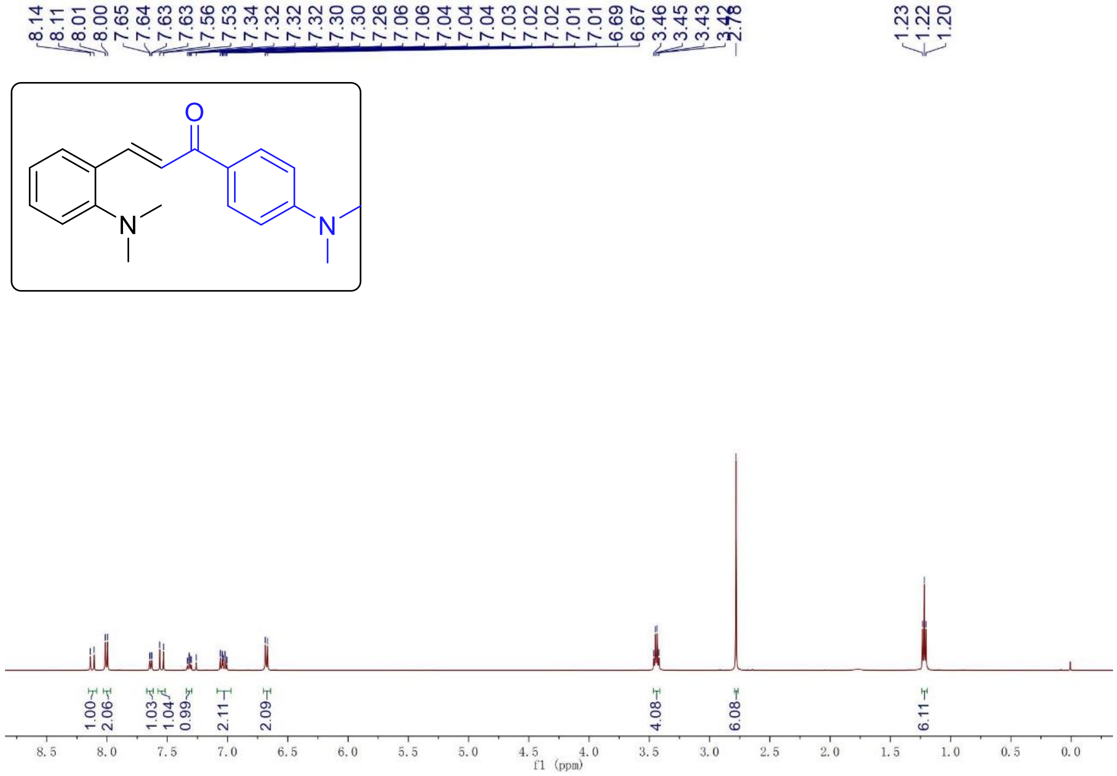


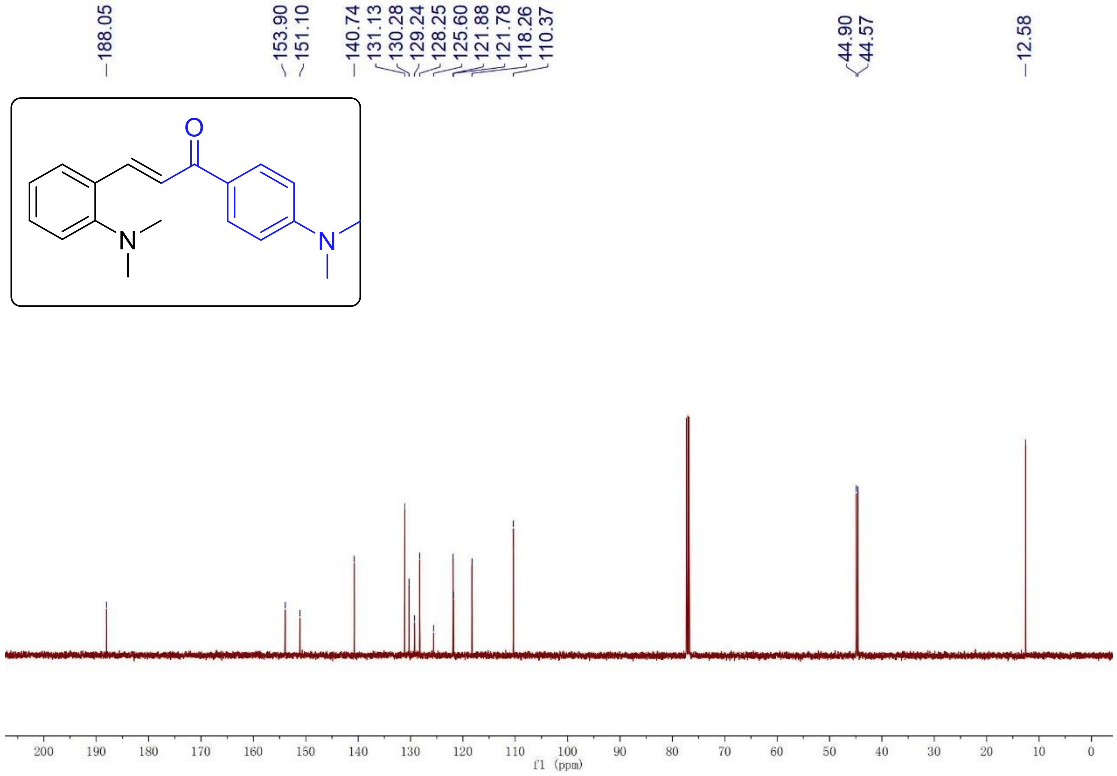


Supplementary Figure 7. $^{1}$H NMR and $^{13}$C NMR spectrum of **1f**.

**(E)-3-(2-(dimethylamino)phenyl)-1-(4-methoxyphenyl)prop-2-en-1-one (1g)**

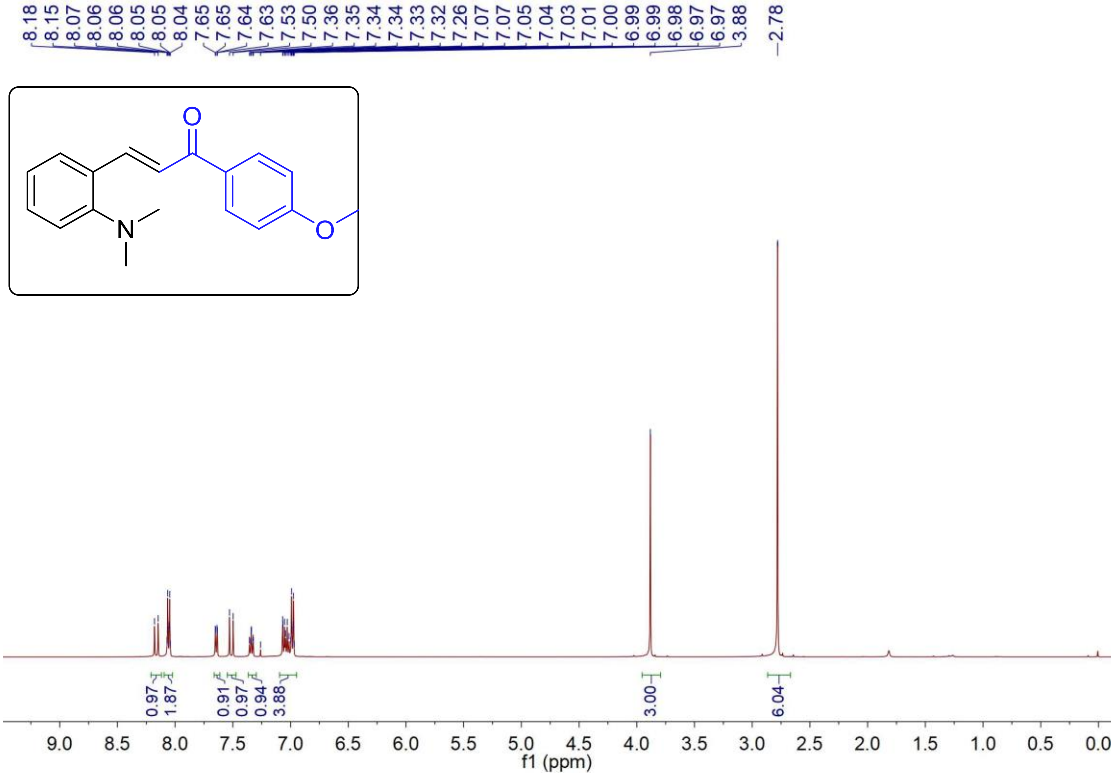


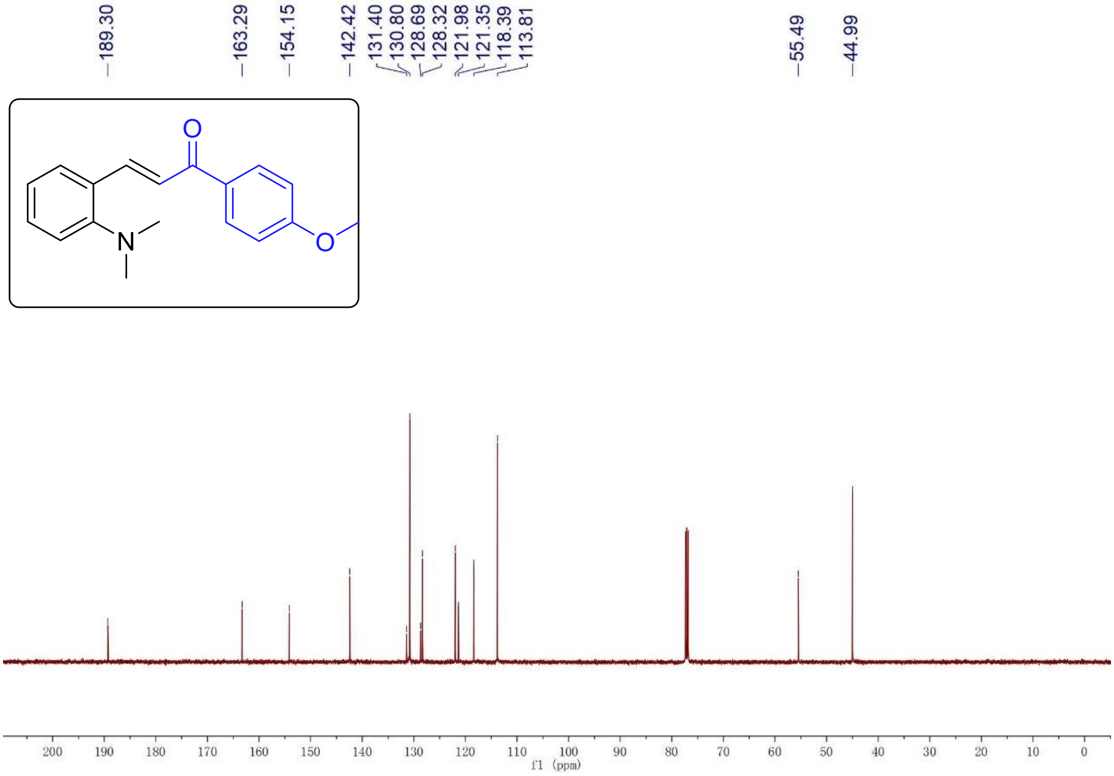


Supplementary Figure 8. $^{1}$H NMR and $^{13}$C NMR spectrum of **1g**.

**(E)-3-(2-(dimethylamino)phenyl)-1-(3,4-dimethylphenyl)prop-2-en-1-one (1h)**

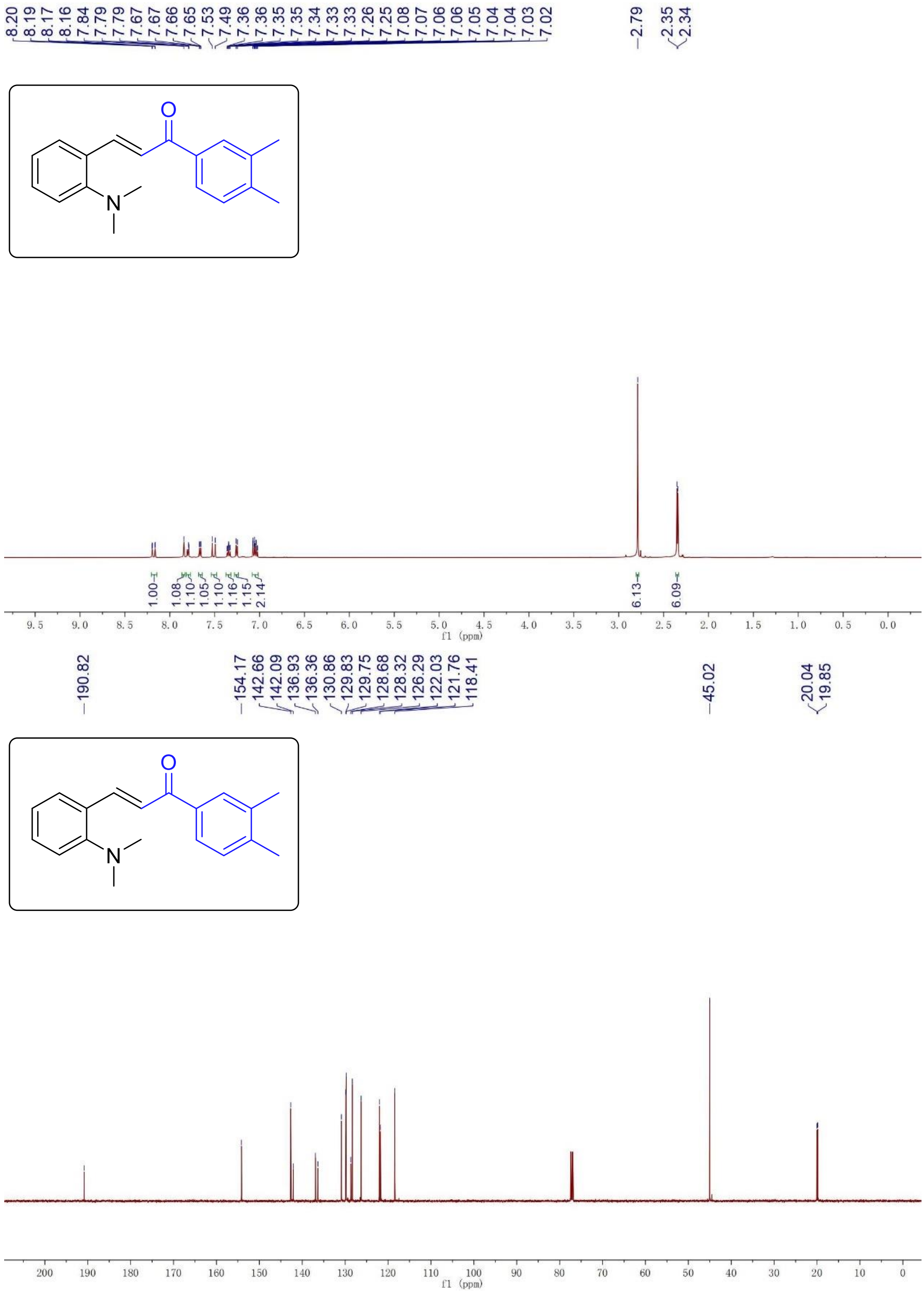


Supplementary Figure 9. $^{1}$H NMR and $^{13}$C NMR spectrum of **1h**.

**(E)-3-(2-(dimethylamino)phenyl)-1-(o-tolyl)prop-2-en-1-one (1i)**

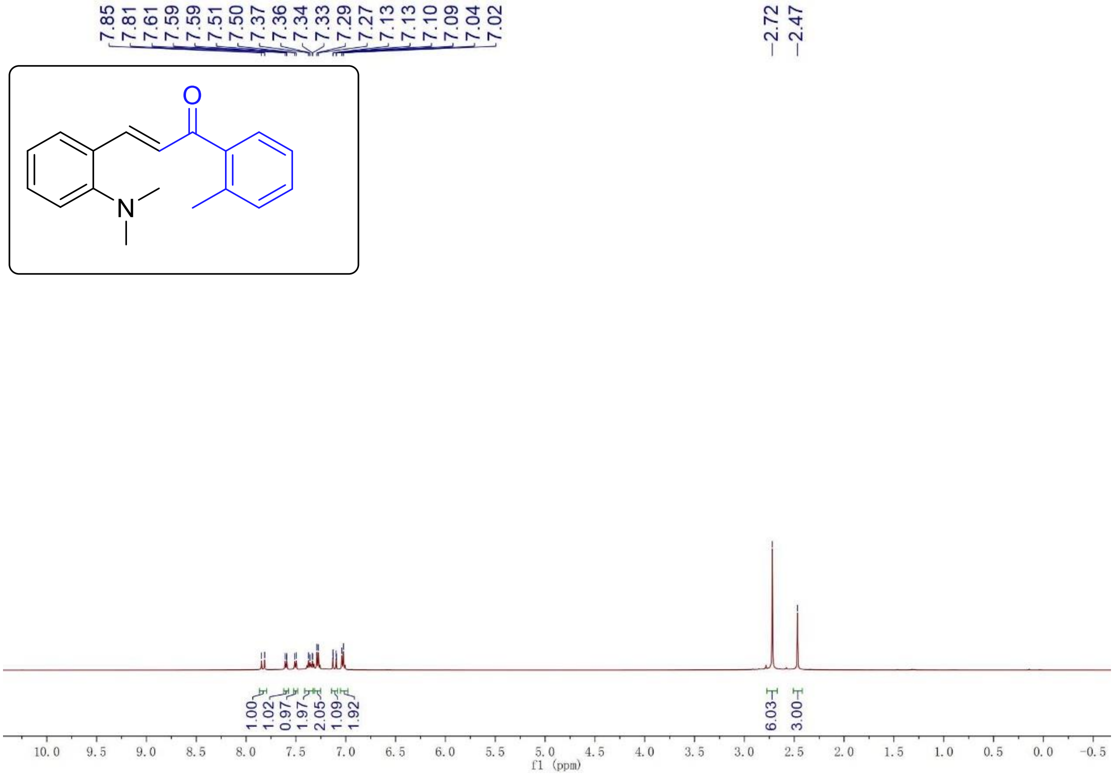


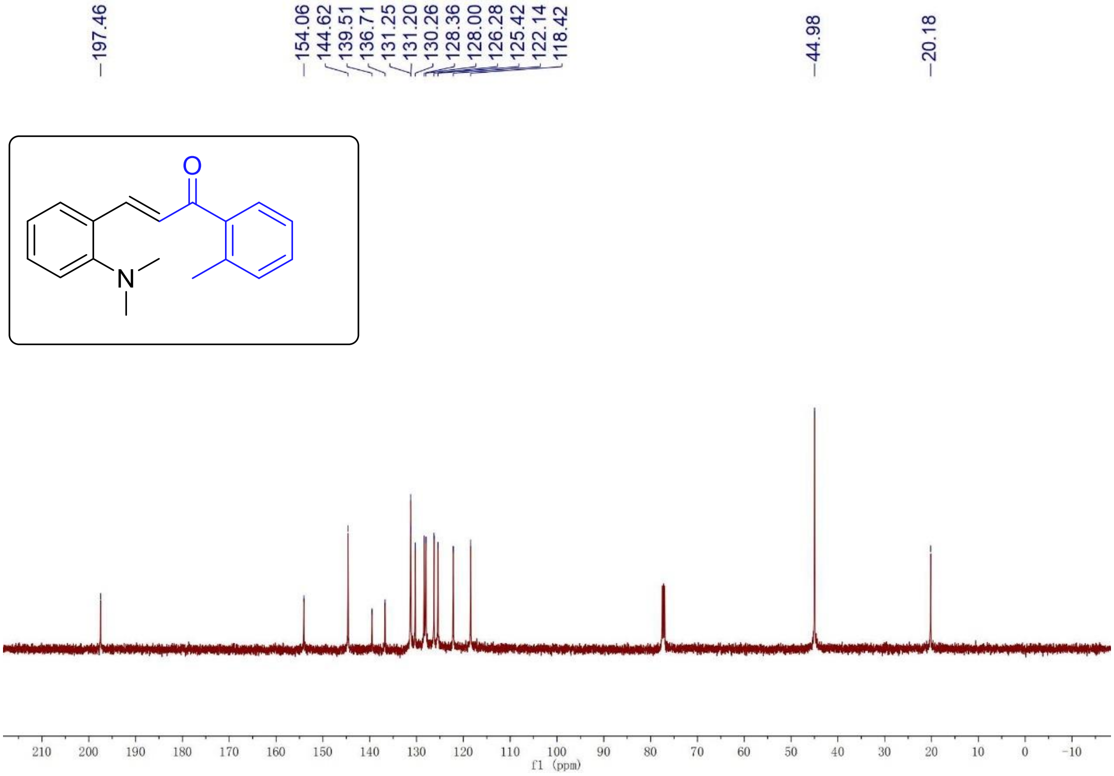


Supplementary Figure 10. $^{1}$H NMR and $^{13}$C NMR spectrum of **1i**.

**(E)-3-(2-(dimethylamino)phenyl)-1-(4-iodophenyl)prop-2-en-1-one (1j)**

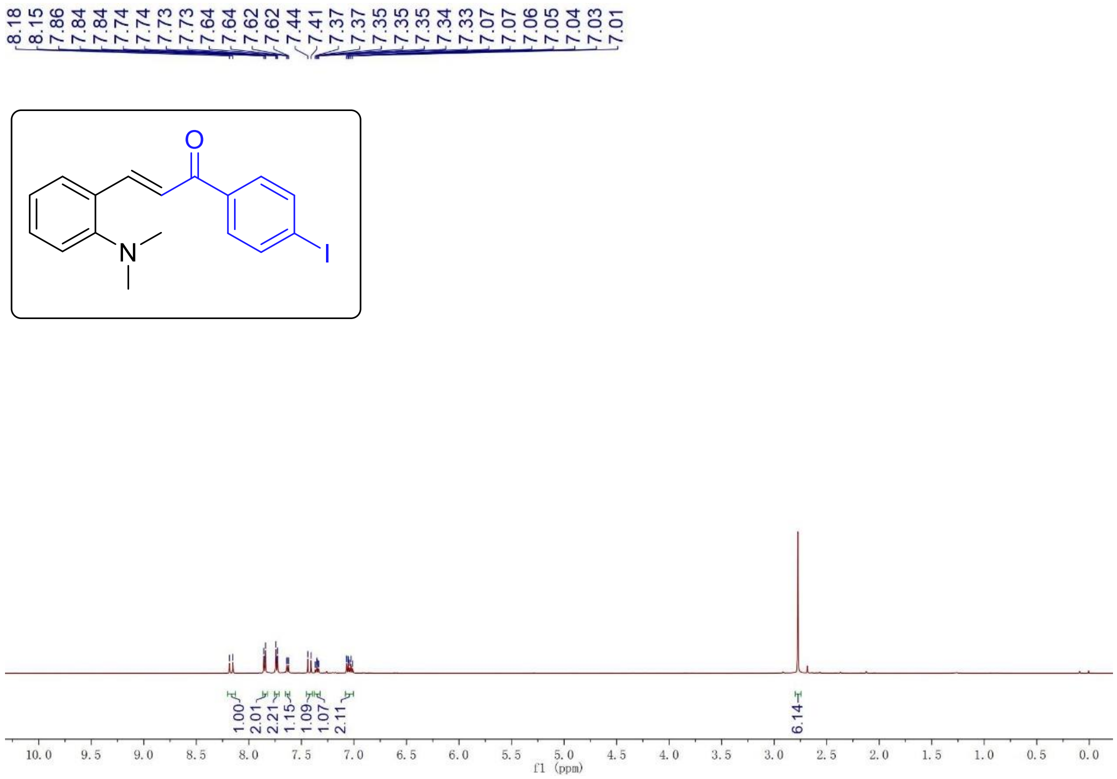

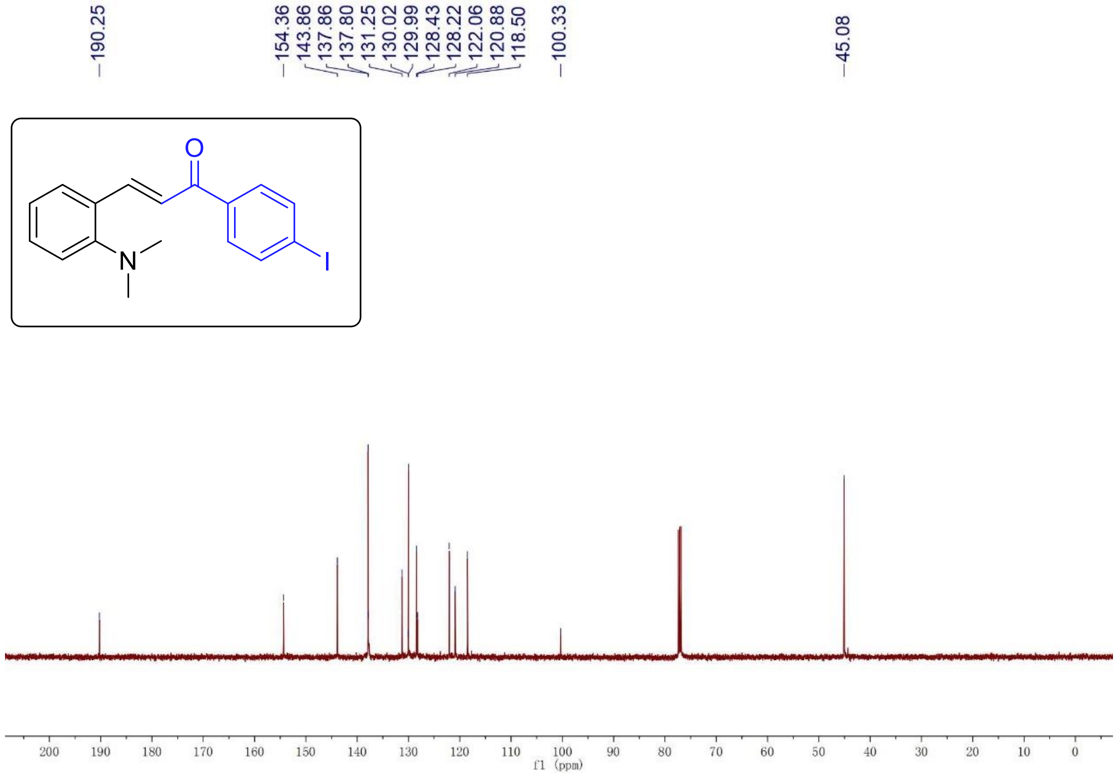

Supplementary Figure 11. $^{1}$H NMR and $^{13}$C NMR spectrum of **1j**.

**(E)-3-(2-(dimethylamino)phenyl)-1-(naphthalen-2-yl)prop-2-en-1-one (1k)**

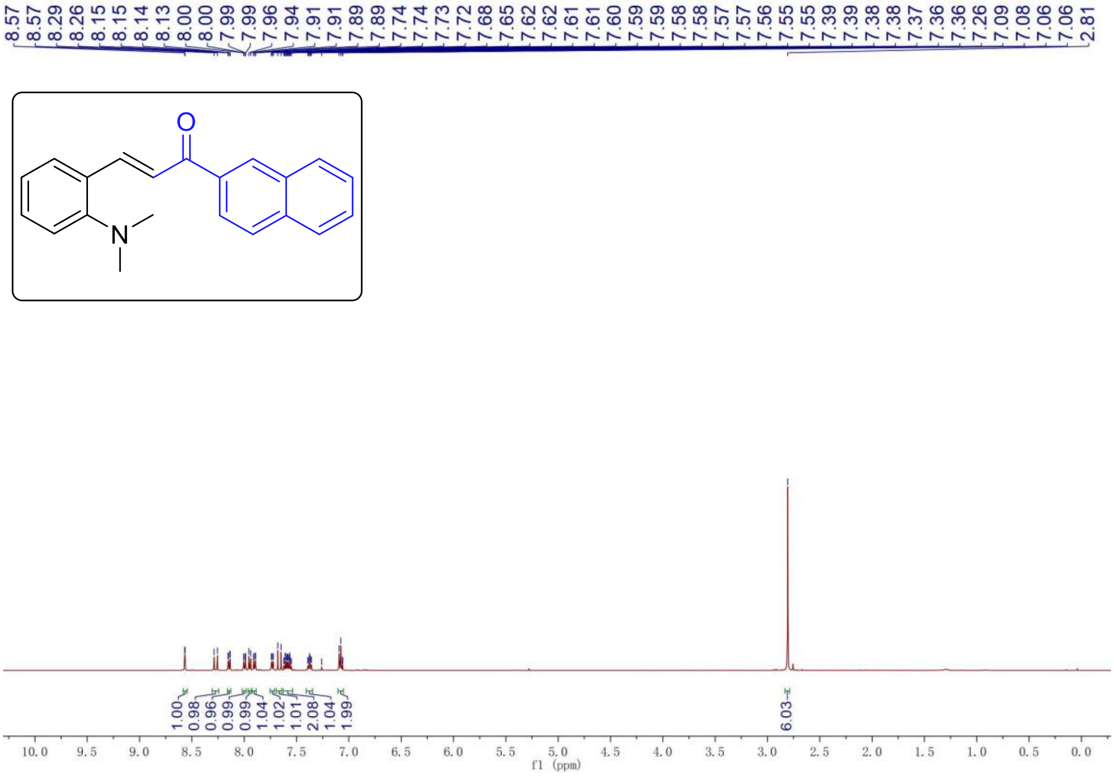


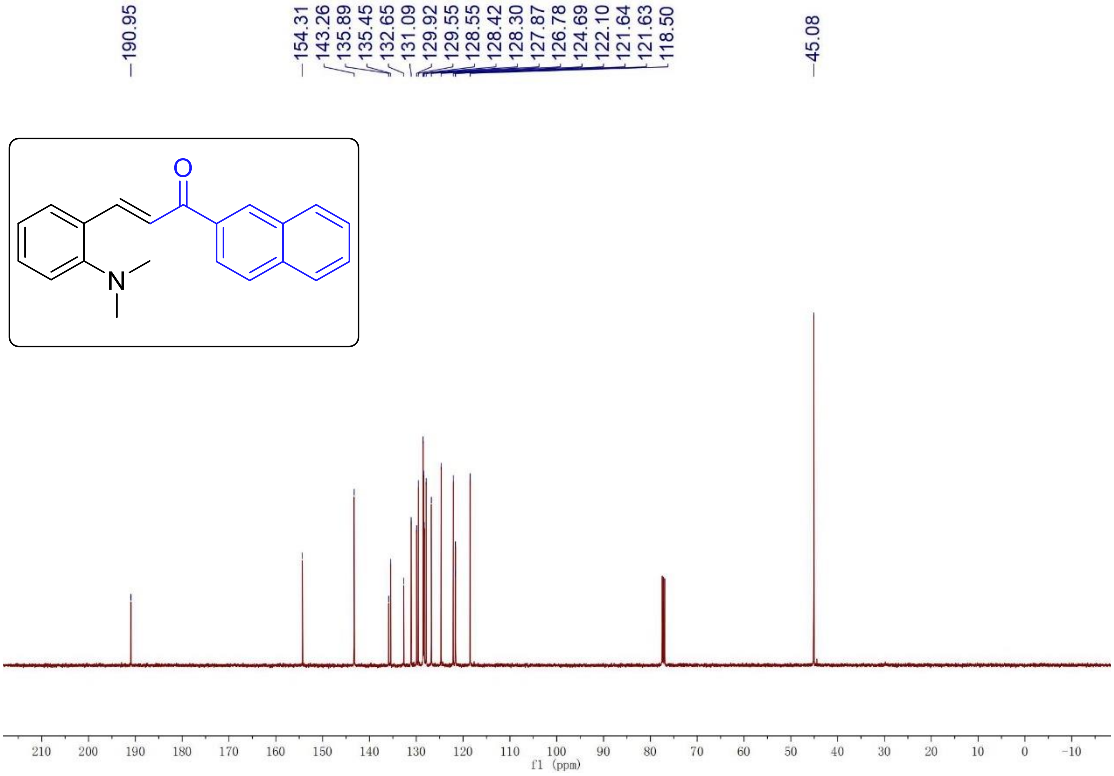


Supplementary Figure 12. $^{1}$H NMR and $^{13}$C NMR spectrum of **1k**.

**(E)-3-(2-(dimethylamino)phenyl)-1-(furan-2-yl)prop-2-en-1-one (1l)**

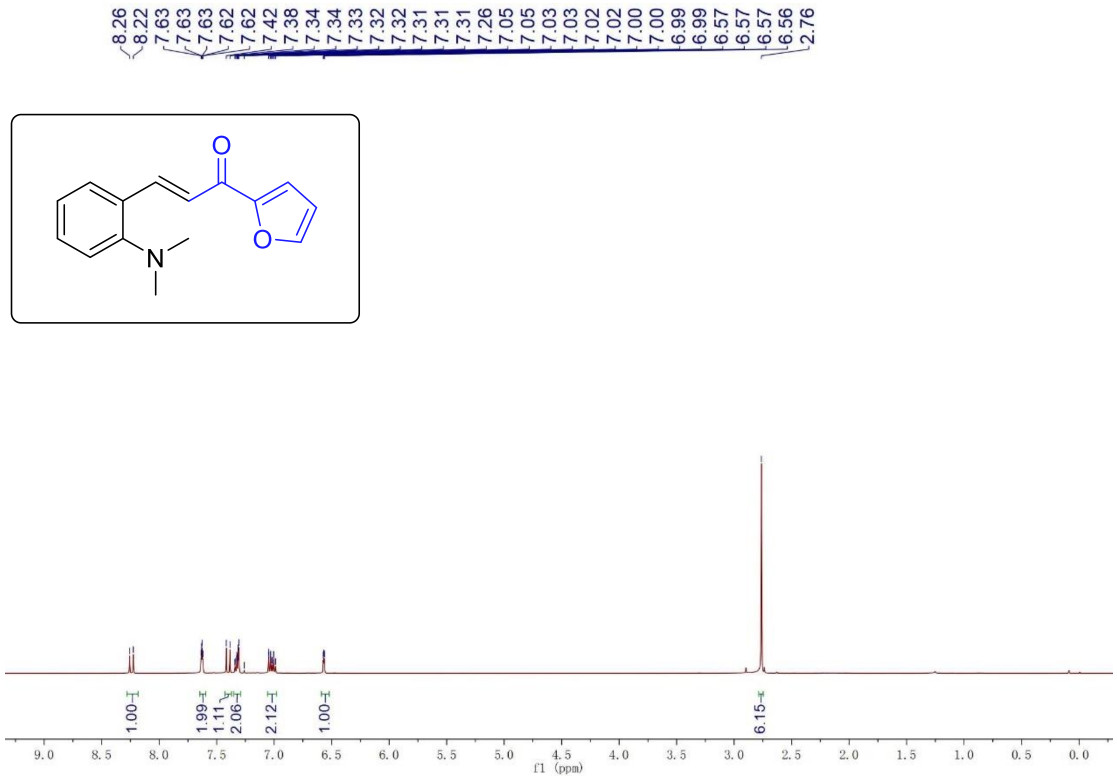

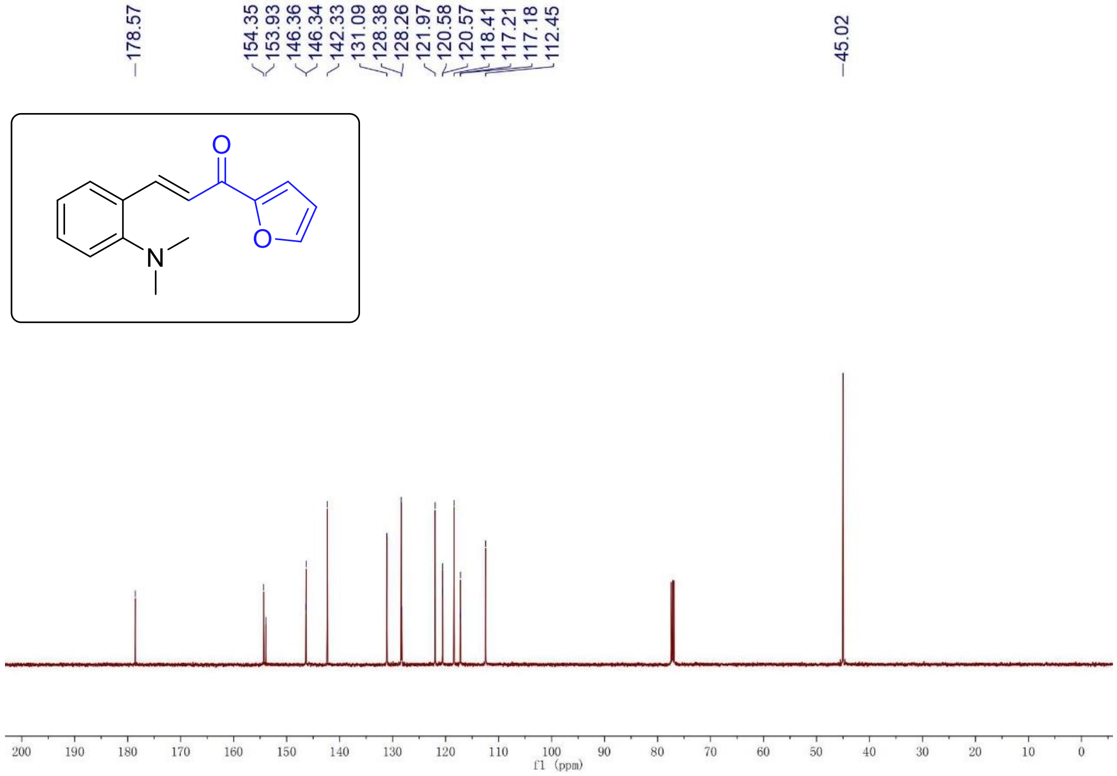

Supplementary Figure 13. $^{1}$H NMR and $^{13}$C NMR spectrum of **1l**.

**(E)-3-(2-(dimethylamino)phenyl)-1-(thiophen-3-yl)prop-2-en-1-one (1m)**

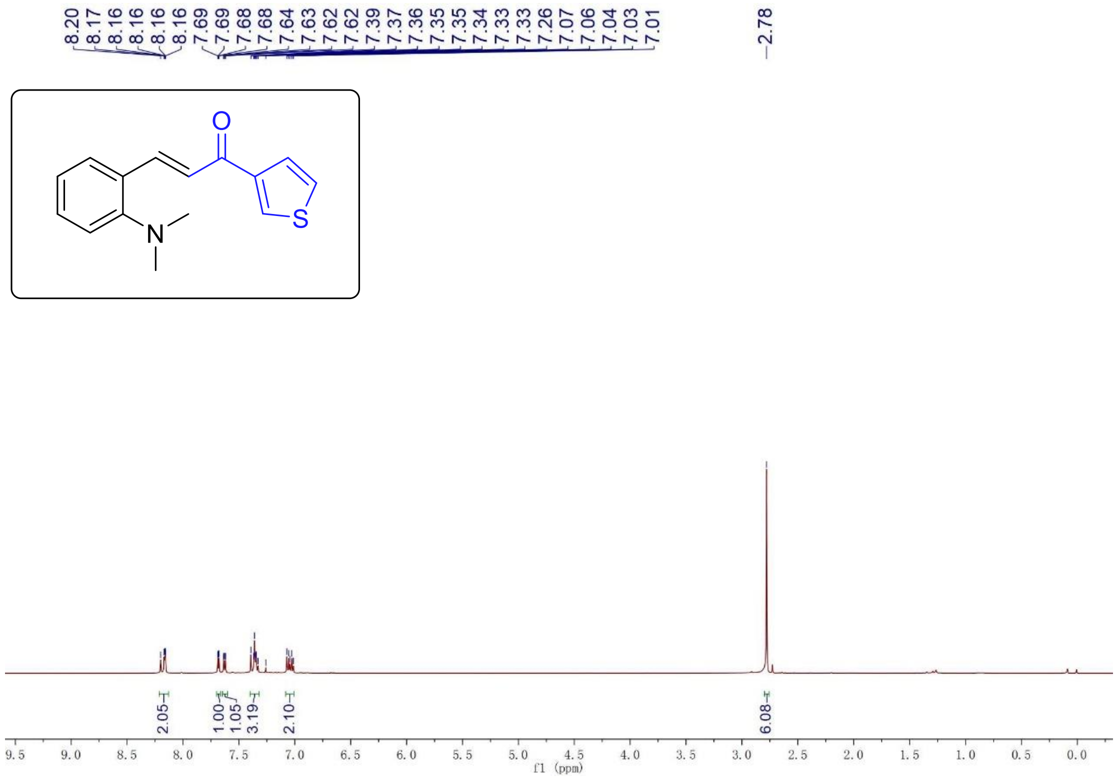


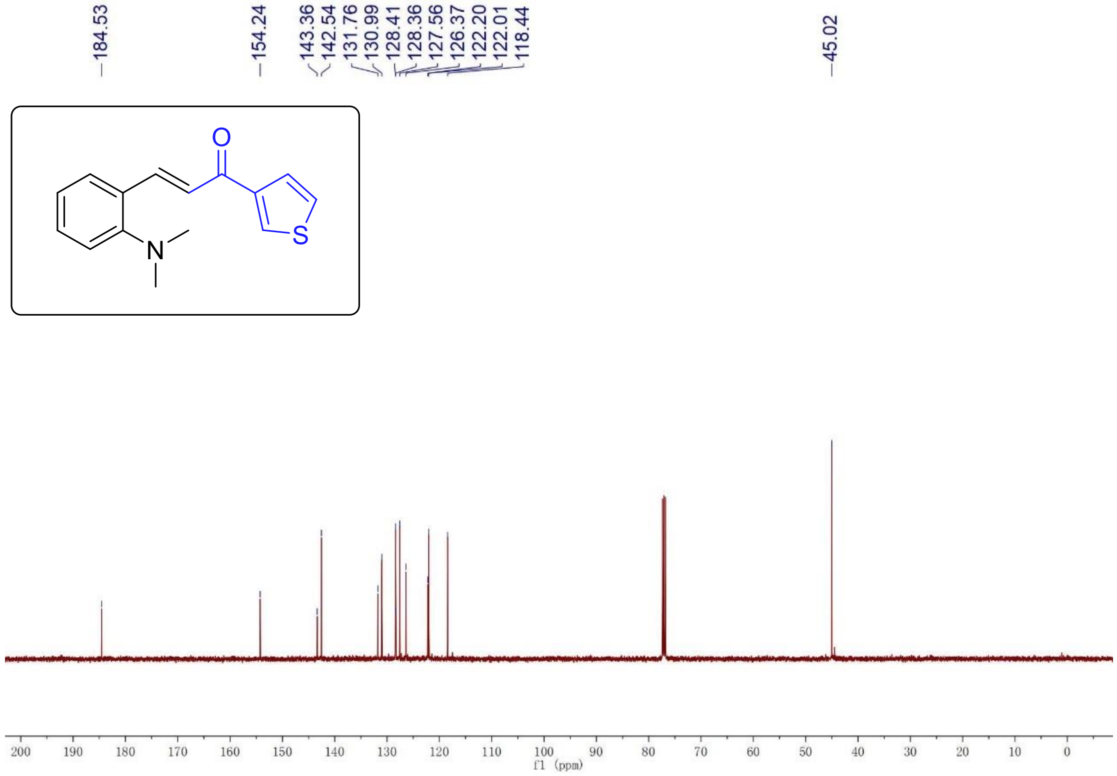


Supplementary Figure 14. $^{1}H$ NMR and $^{13}C$ NMR spectrum of **1m**.

**(E)-1,1'-(1,3-phenylene)bis(3-(2-(dimethylamino)phenyl)prop-2-en-1-one) (1n)**

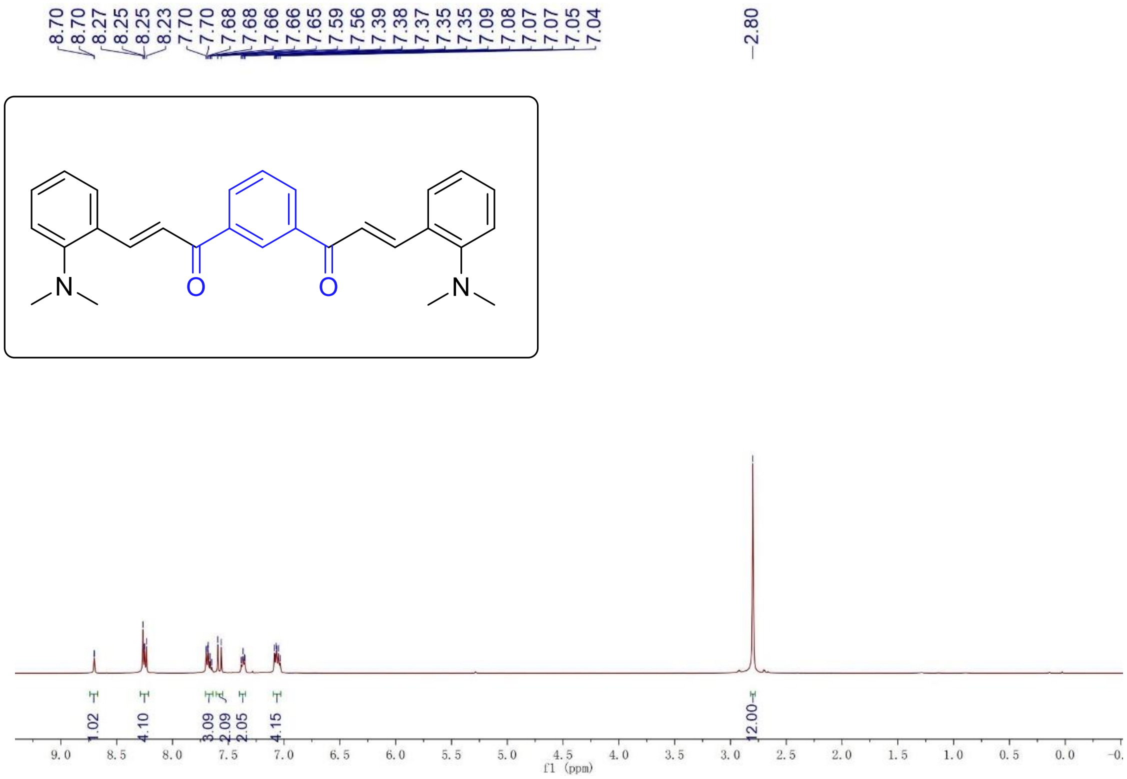

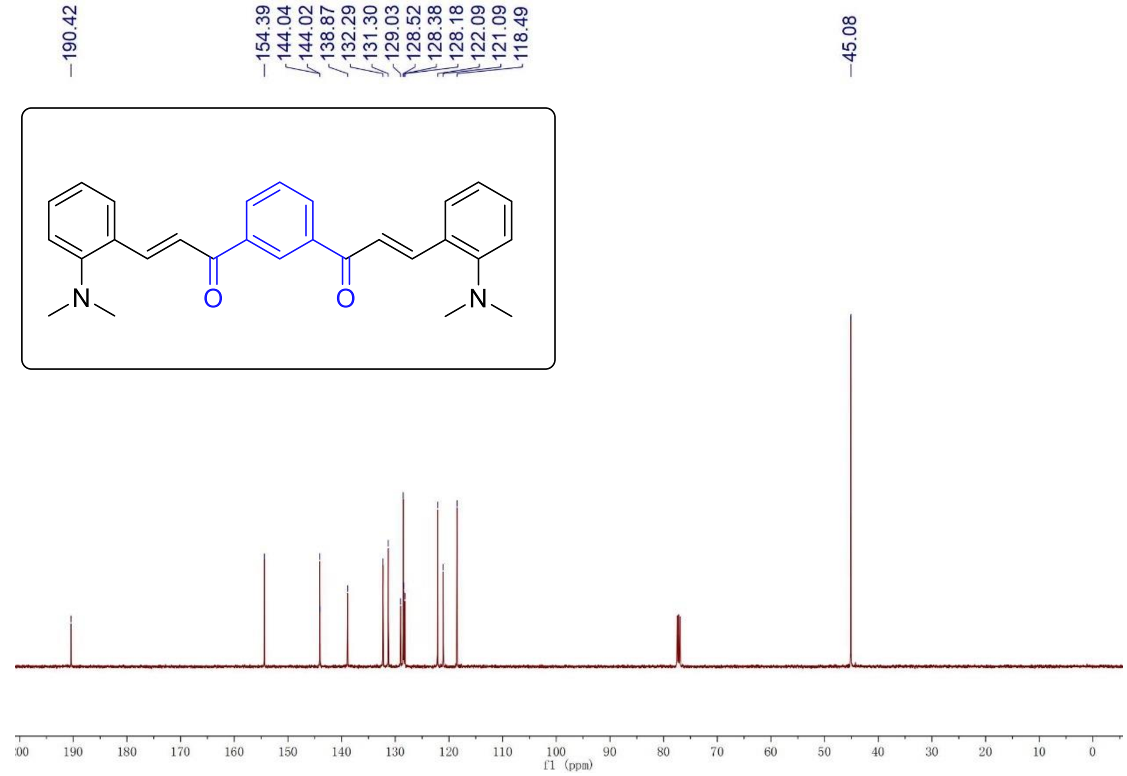

Supplementary Figure 15. $^{1}$H NMR and $^{13}$C NMR spectrum of **1n**.

**(E)-2-(2-(dimethylamino)benzylidene)-2,3-dihydro-1H-inden-1-one (1o)**

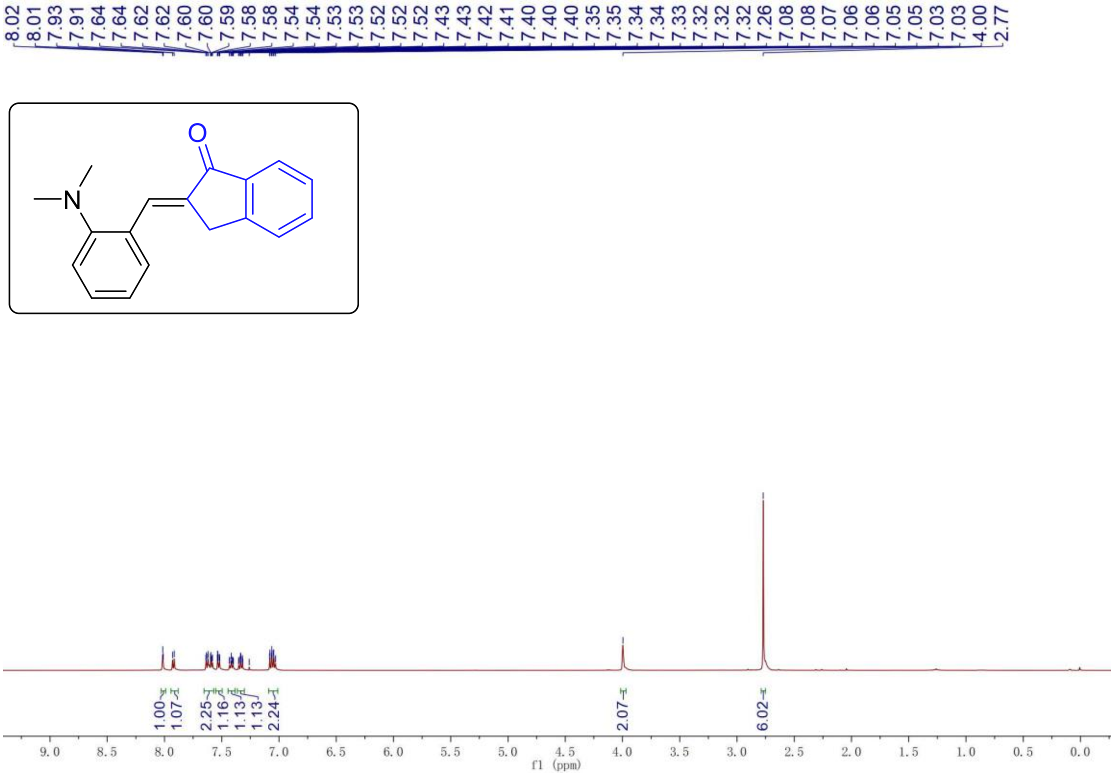


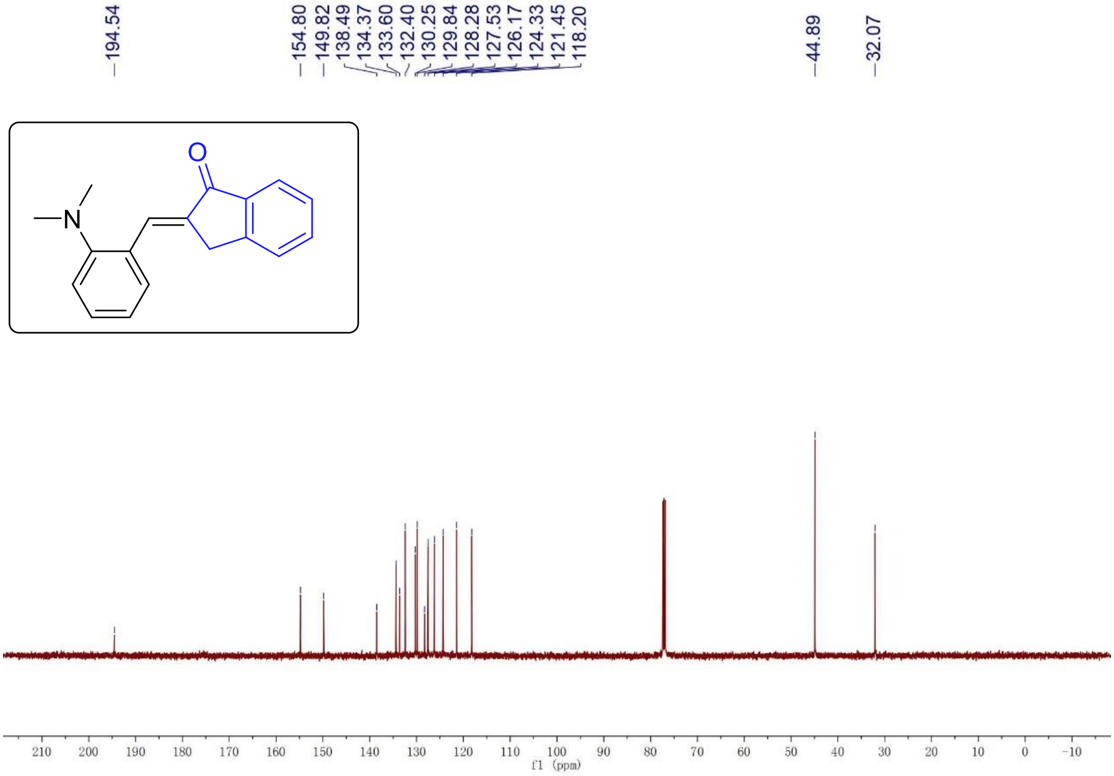


Supplementary Figure 16. $^{1}$H NMR and $^{13}$C NMR spectrum of **1o**.

**(E)-2-(2-(dimethylamino)benzylidene)-3,4-dihydronaphthalen-1(2H)-one (1p)**

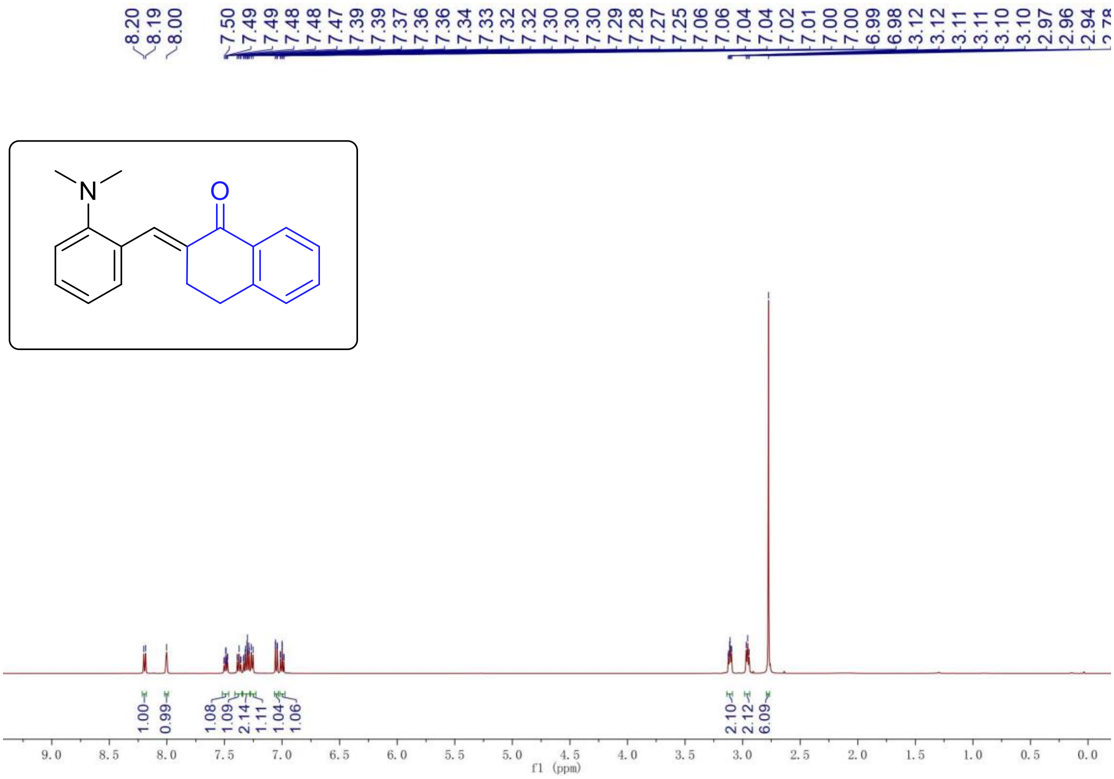


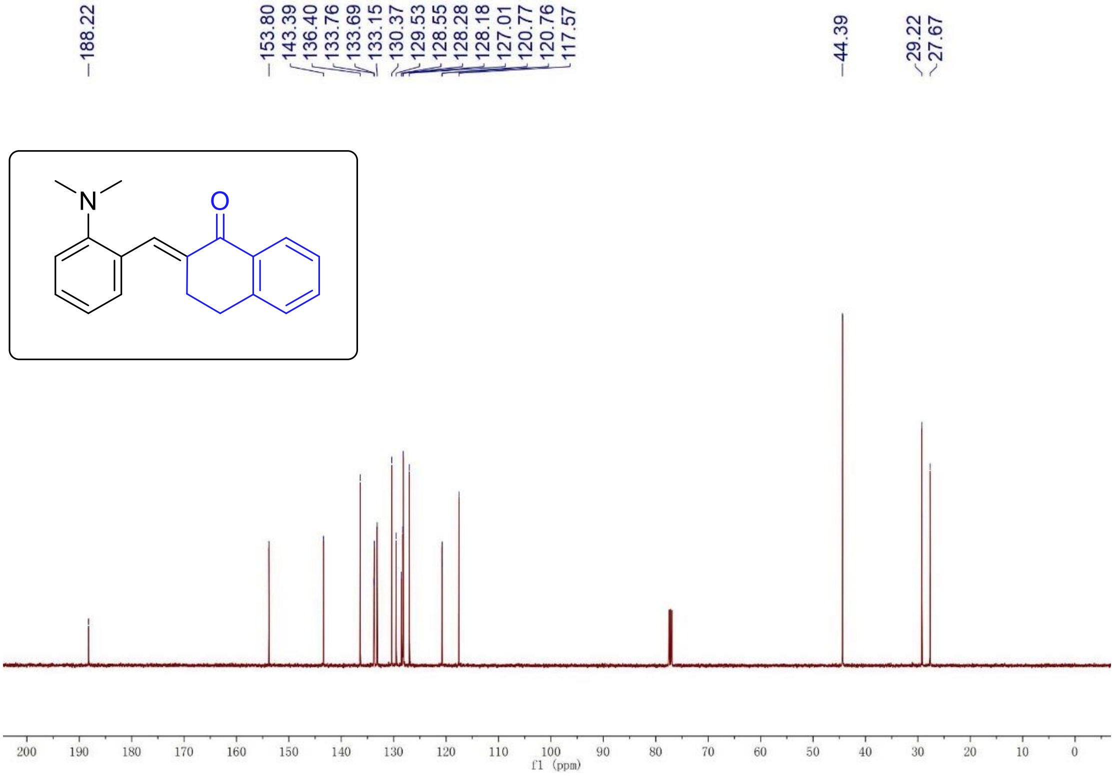


Supplementary Figure 17. $^{1}$H NMR and $^{13}$C NMR spectrum of **1p**.

**(E)-3-(2-(dimethylamino)benzylidene)chroman-4-one (1q)**

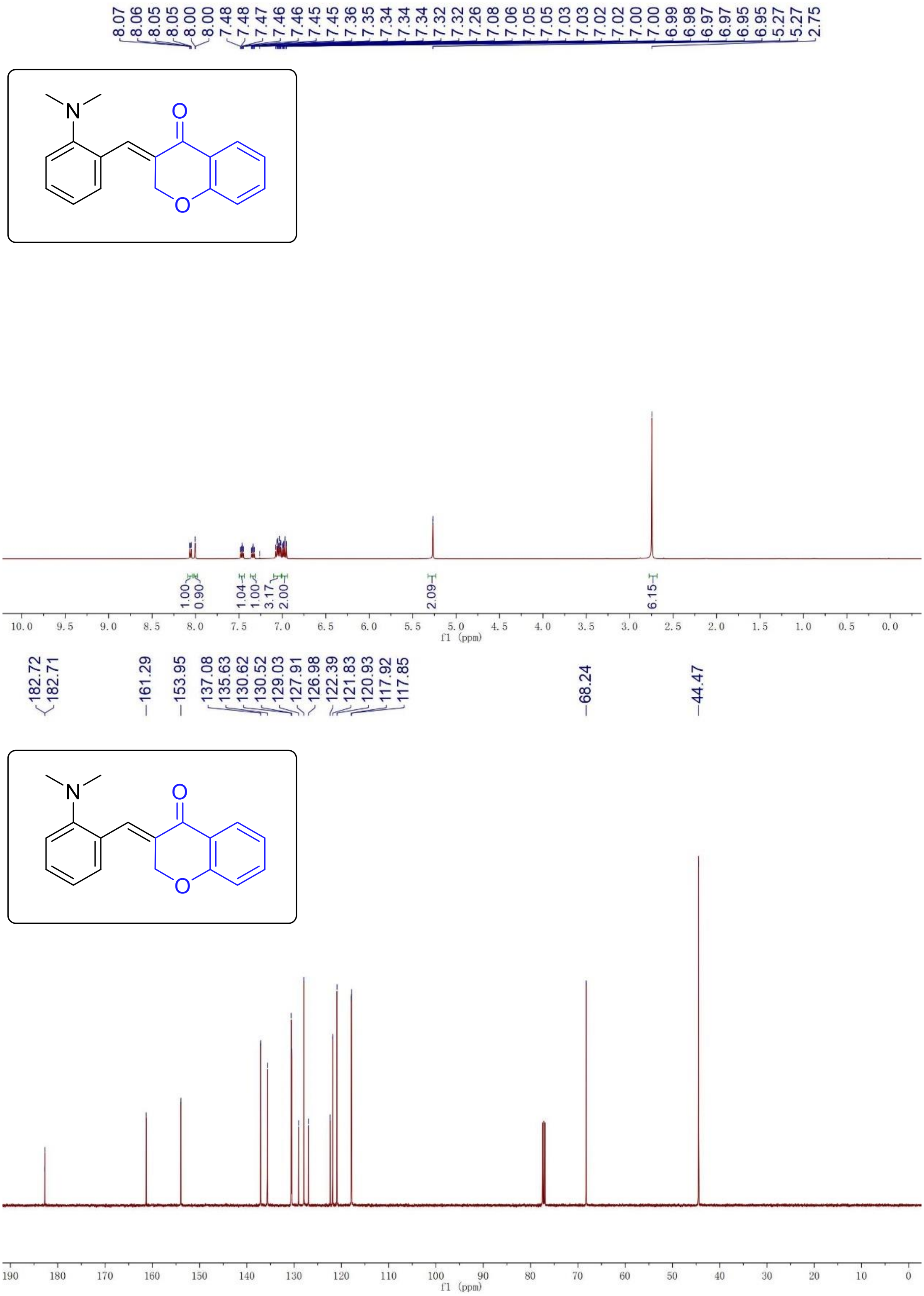


Supplementary Figure 18. $^{1}H$ NMR and $^{13}C$ NMR spectrum of **1q**.

**(E)-6-(2-(dimethylamino)benzylidene)-6,7,8,9-tetrahydro-5H-benzo[7]annulen-5-one (1r)**

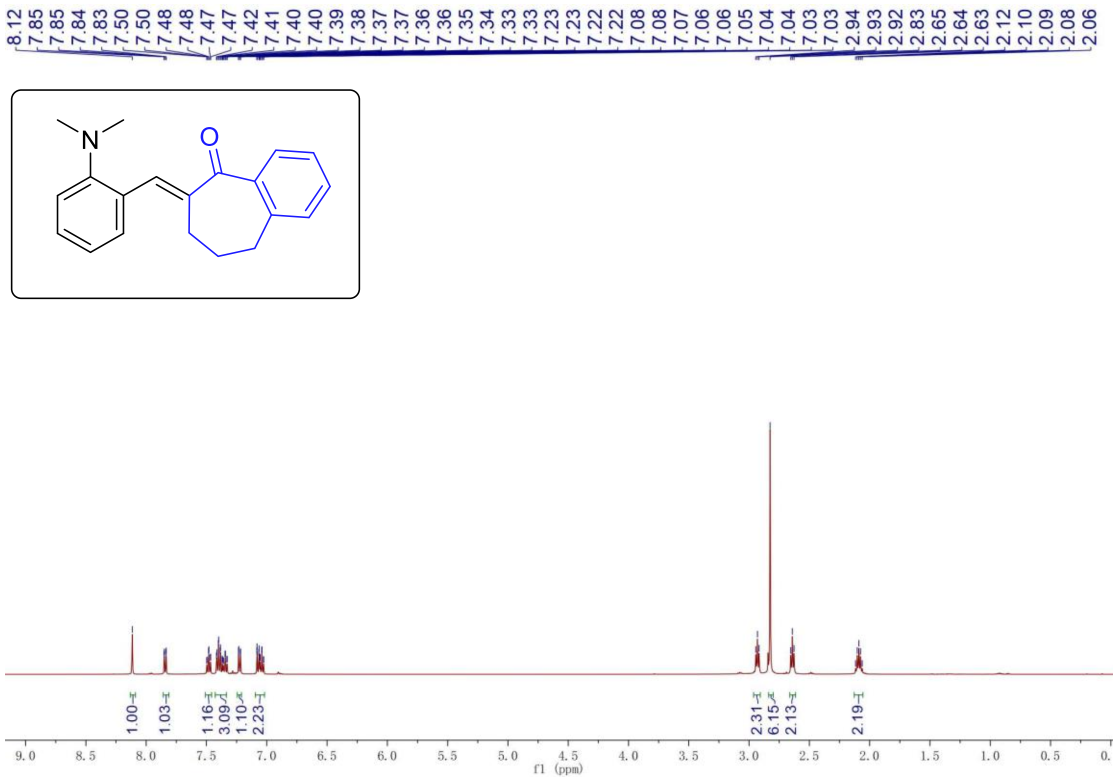


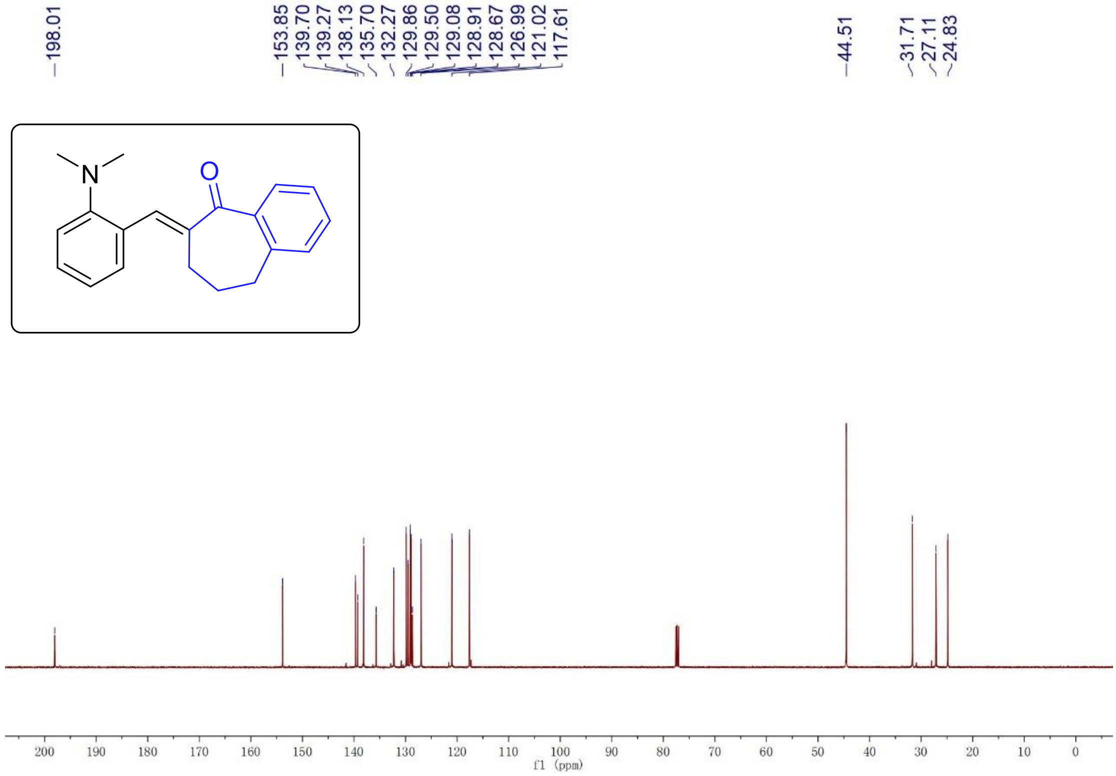


Supplementary Figure 19. $^{1}H$ NMR and $^{13}C$ NMR spectrum of **1r**.

## (E)-4-(2-(dimethylamino)phenyl)but-3-en-2-one (1s)

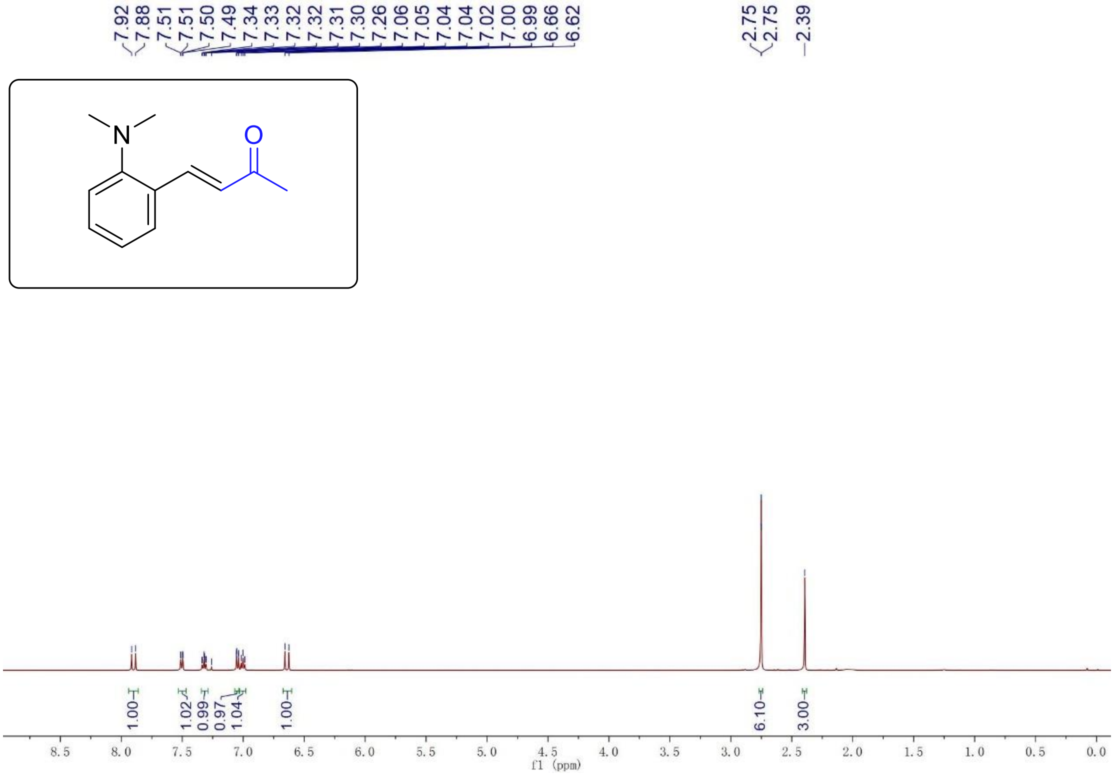


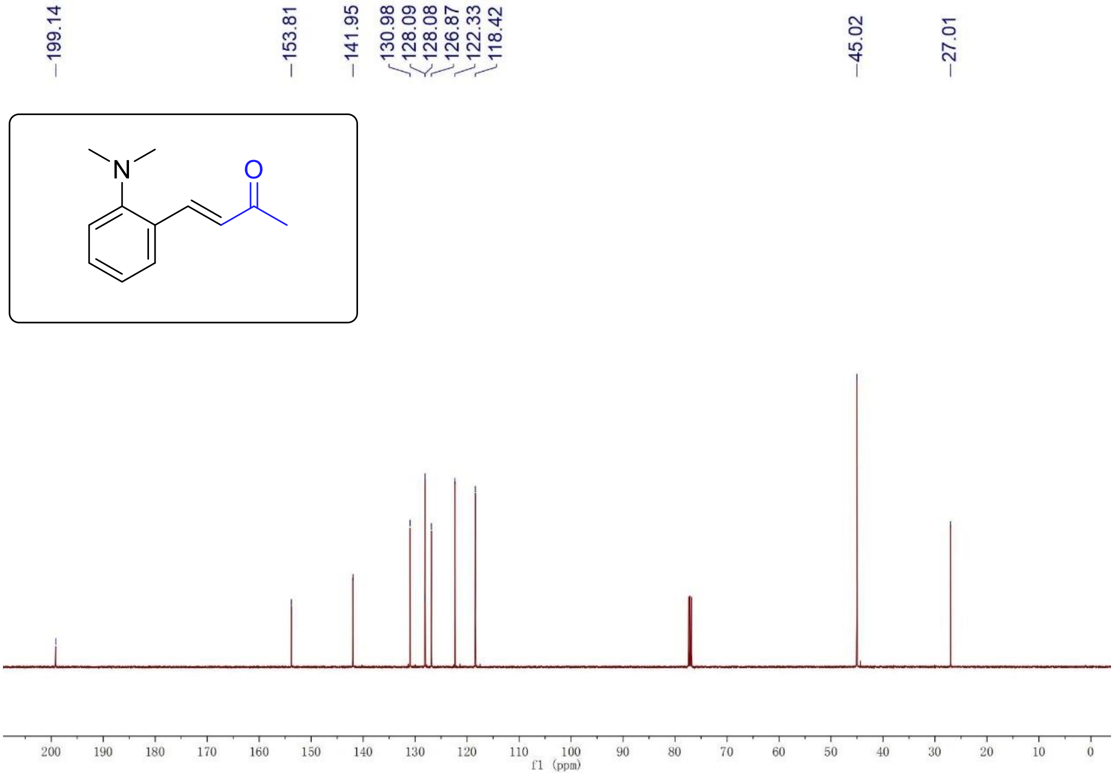


Supplementary Figure 20. $^{1}$H NMR and $^{13}$C NMR spectrum of **1s**.

**(E)-1-(2-(dimethylamino)phenyl)-4-methylpent-1-en-3-one (1t)**

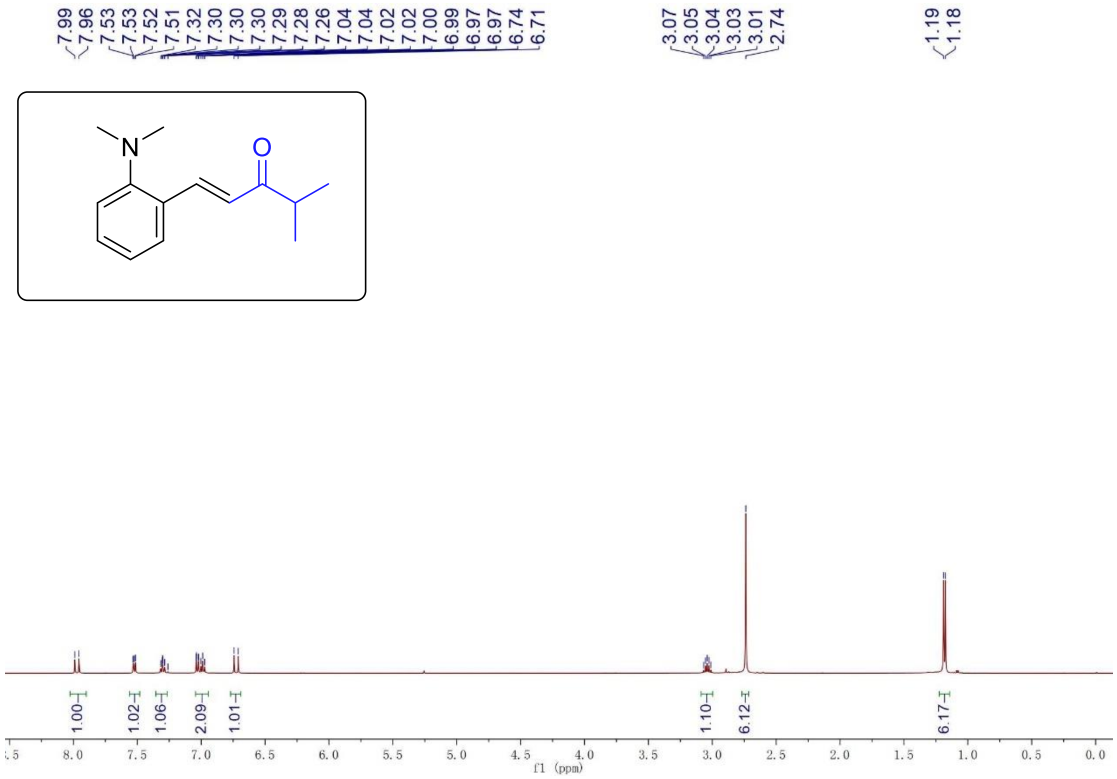


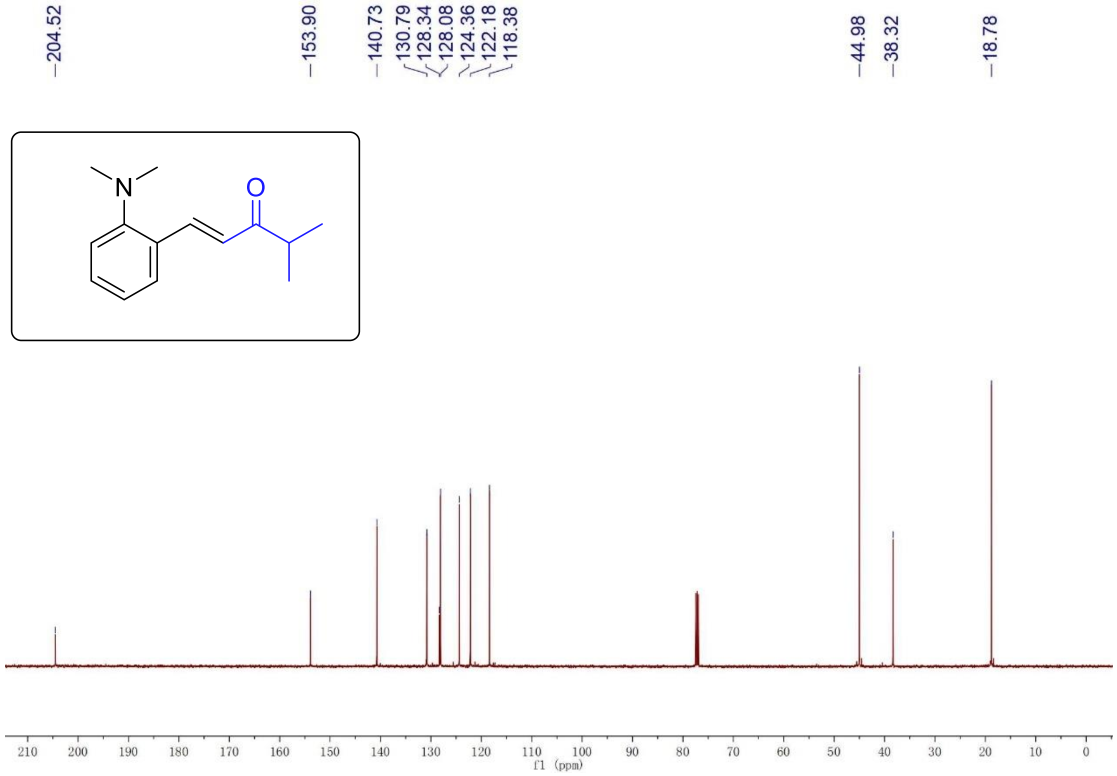


Supplementary Figure 21. $^{1}$H NMR and $^{13}$C NMR spectrum of **1t**.

**(E)-1-(2-(dimethylamino)phenyl)-4,4-dimethylpent-1-en-3-one (1u)**

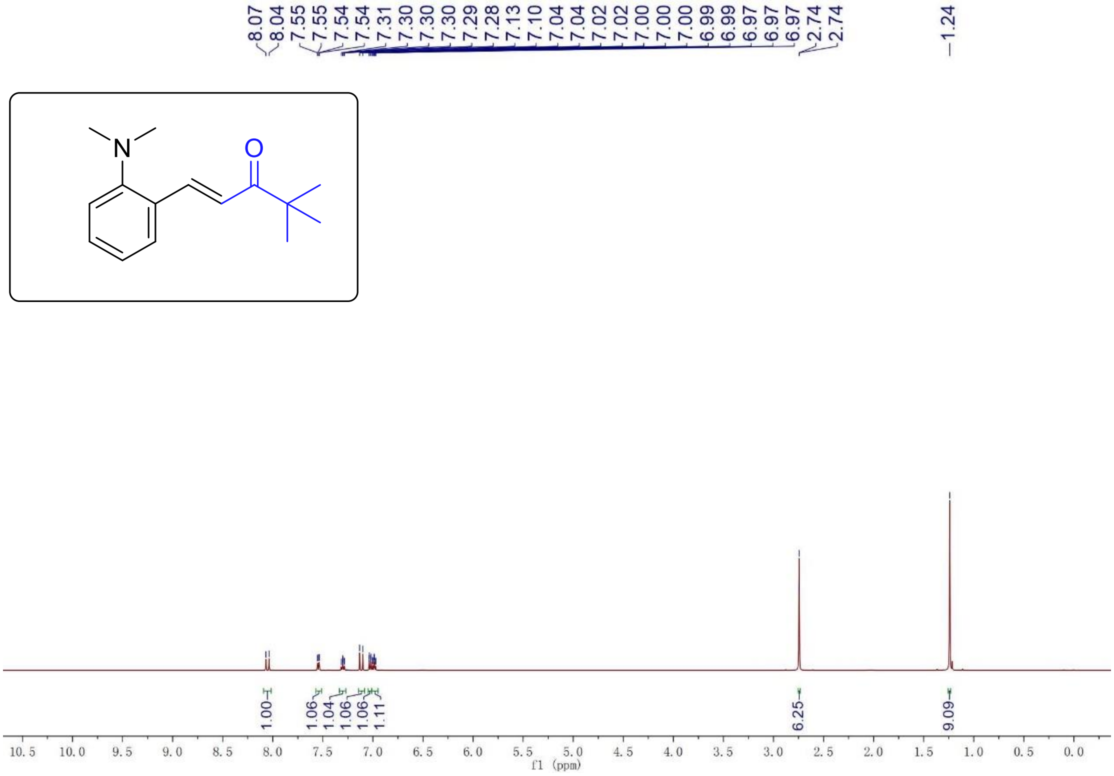

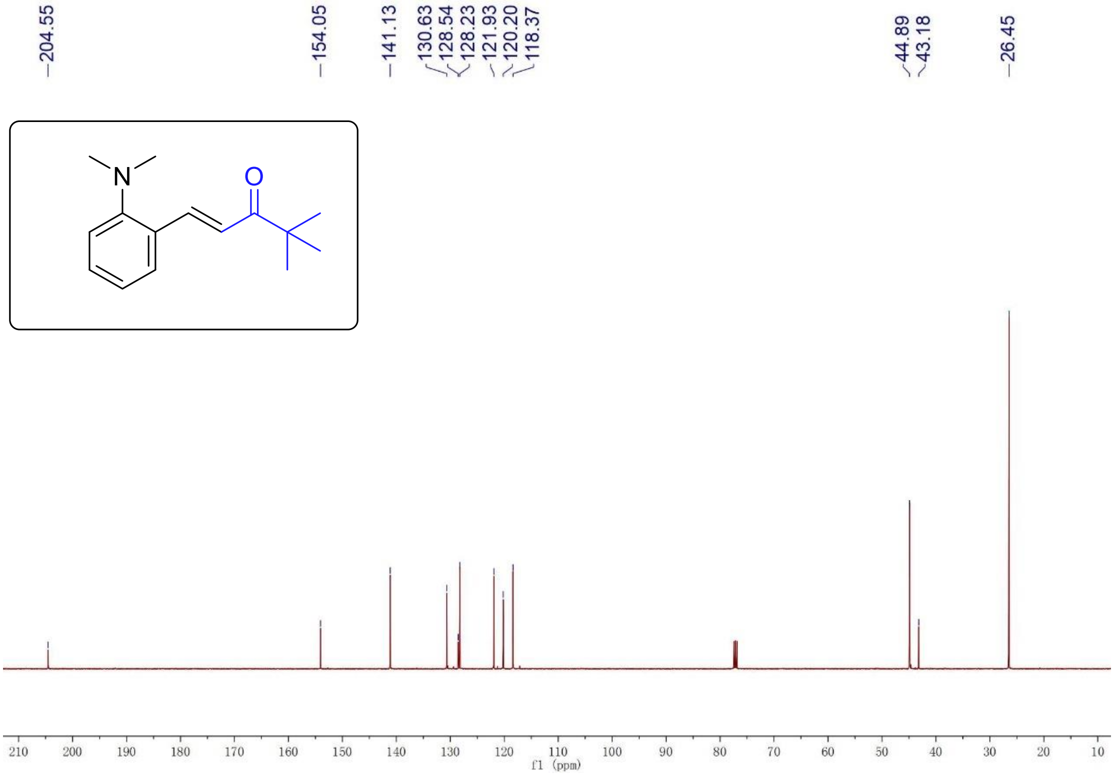

Supplementary Figure 22. $^{1}H$ NMR and $^{13}C$ NMR spectrum of **1u**.

**(E)-1-(2-(dimethylamino)phenyl)-2-methylpent-1-en-3-one (1v)**

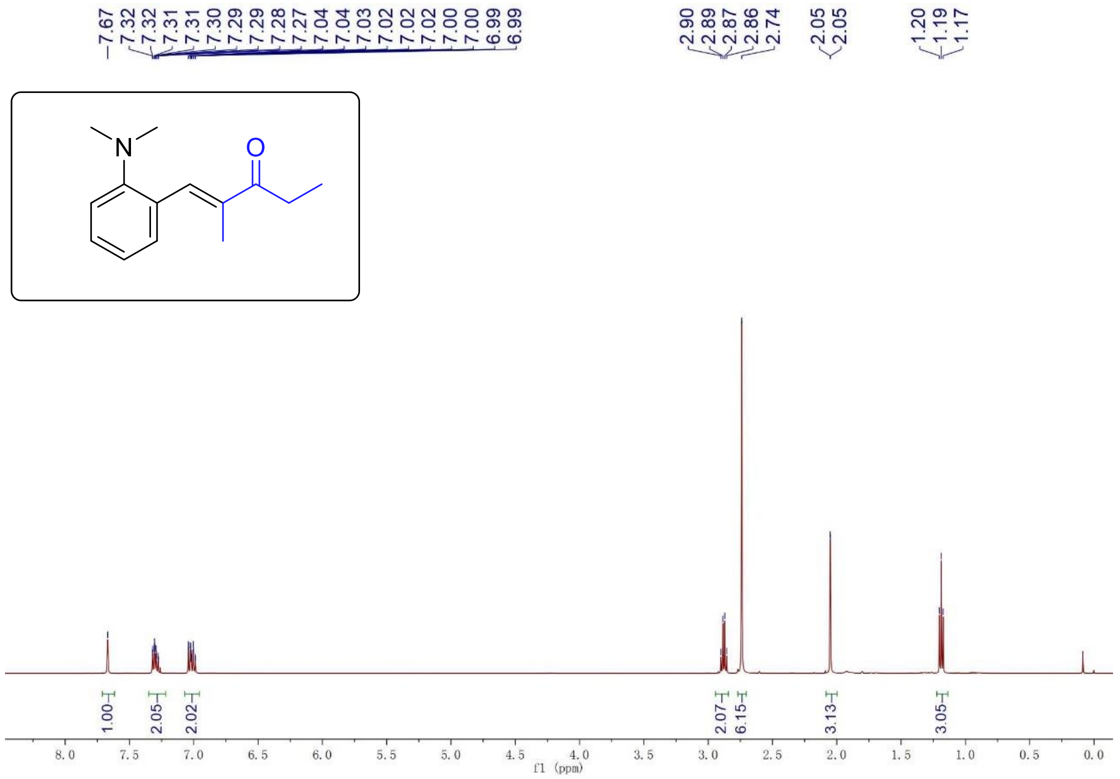


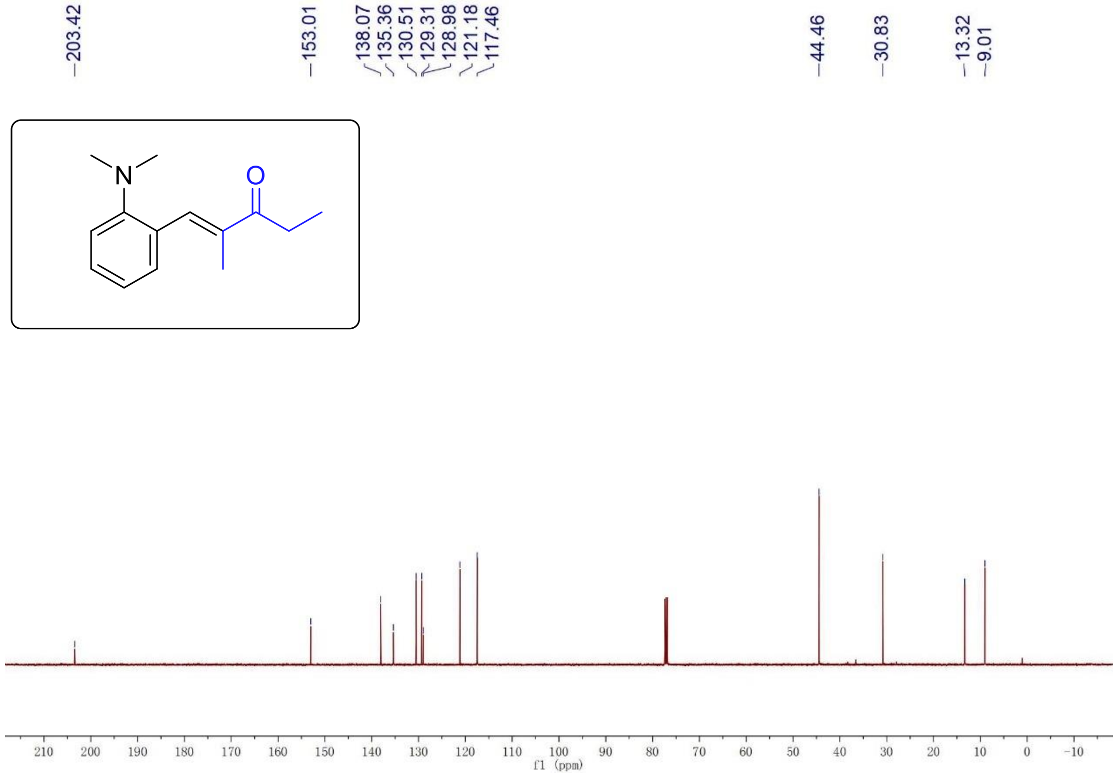


Supplementary Figure 23. $^{1}$H NMR and $^{13}$C NMR spectrum of **1v**.

**(E)-1-(2-(dimethylamino)phenyl)-2,4-dimethylpent-1-en-3-one (1w)**

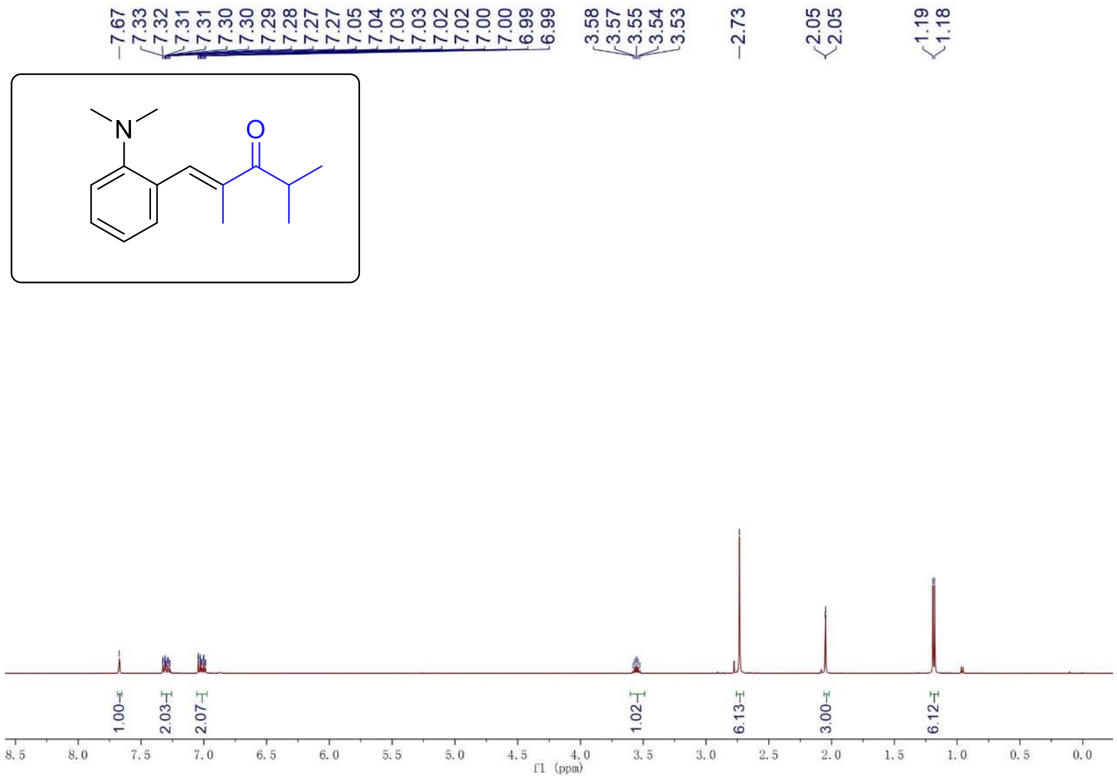


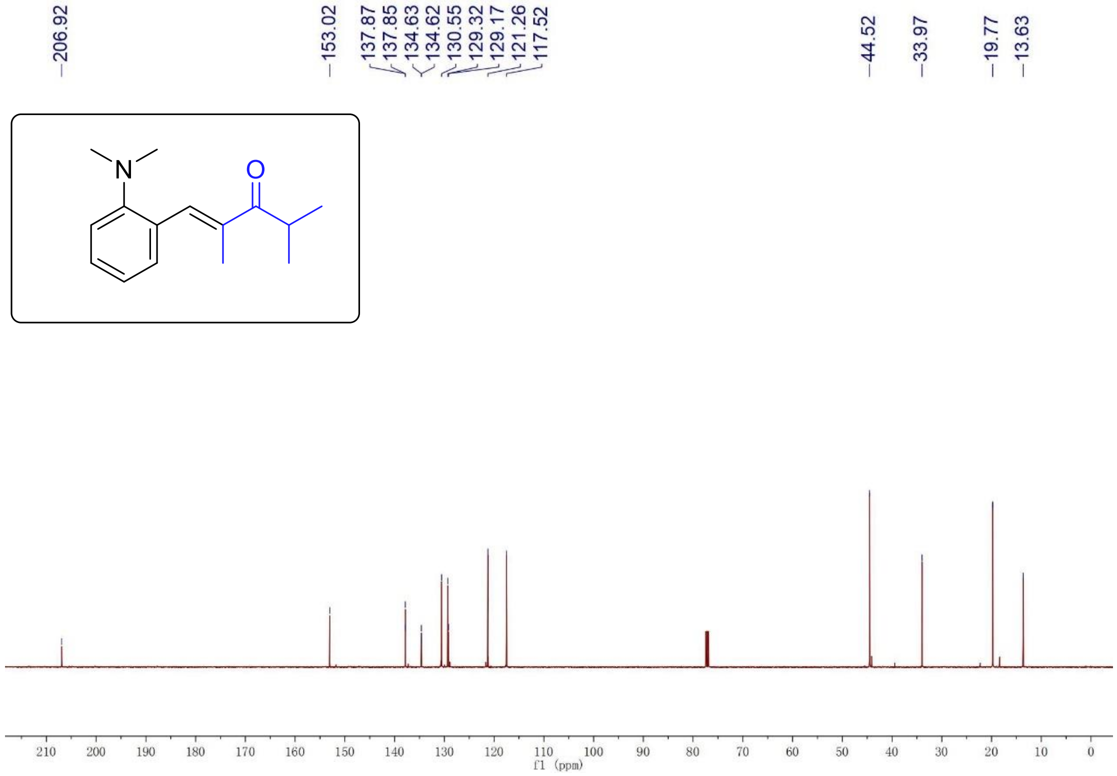


Supplementary Figure 24. $^{1}$H NMR and $^{13}$C NMR spectrum of **1w**.

**(E)-1-(2-(dimethylamino)phenyl)dec-1-en-3-one (1x)**

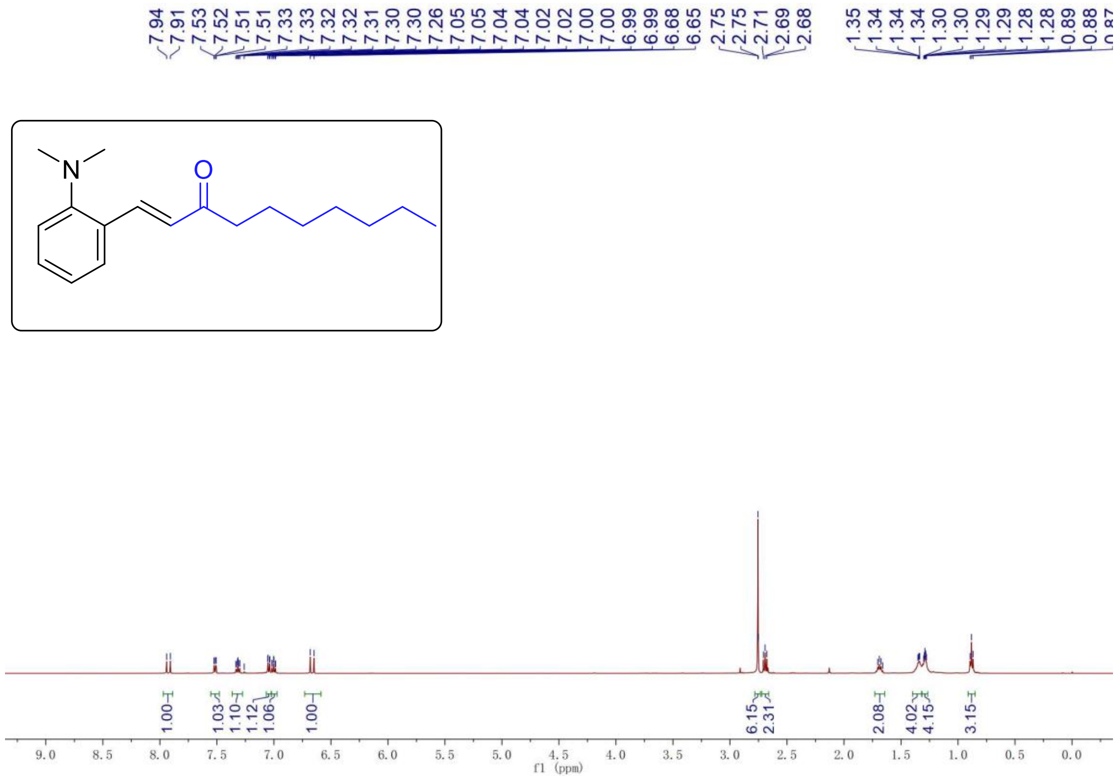


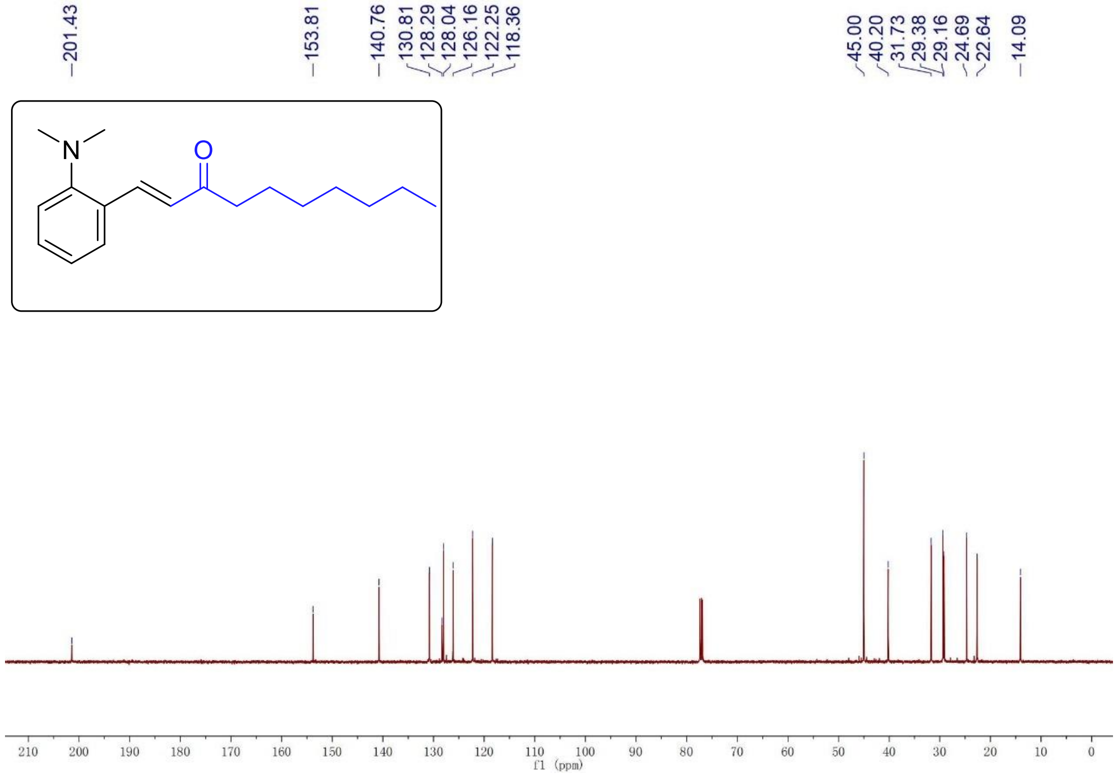


Supplementary Figure 25. $^{1}$H NMR and $^{13}$C NMR spectrum of **1x**.

**(E)-1-cyclopropyl-3-(2-(dimethylamino)phenyl)prop-2-en-1-one (1y)**

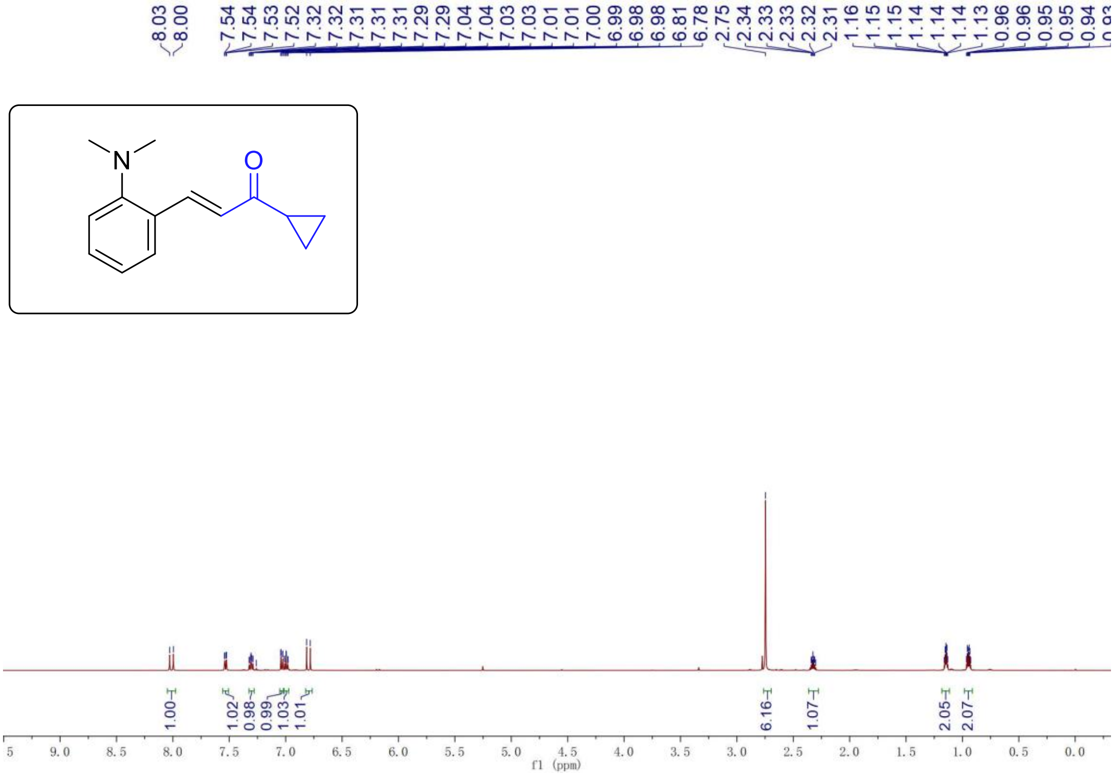

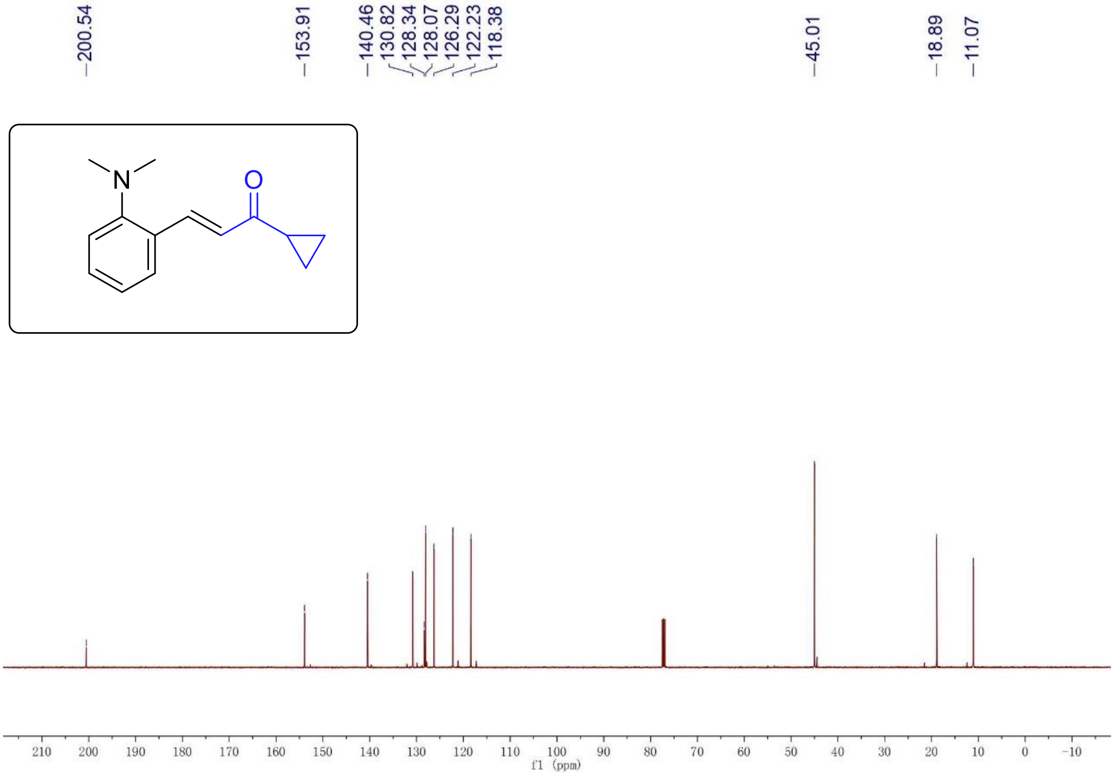

Supplementary Figure 26. $^1$H NMR and $^{13}$C NMR spectrum of **1y**.

**(E)-1-cyclohexyl-3-(2-(dimethylamino)phenyl)prop-2-en-1-one (1z)**

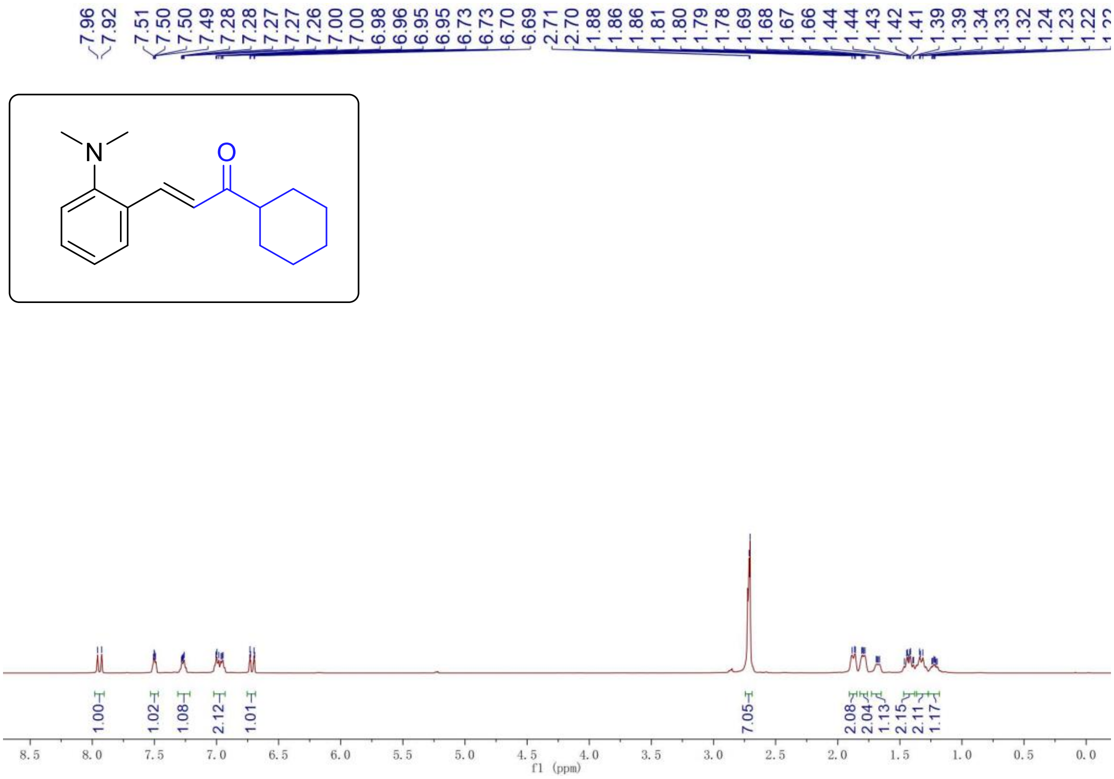

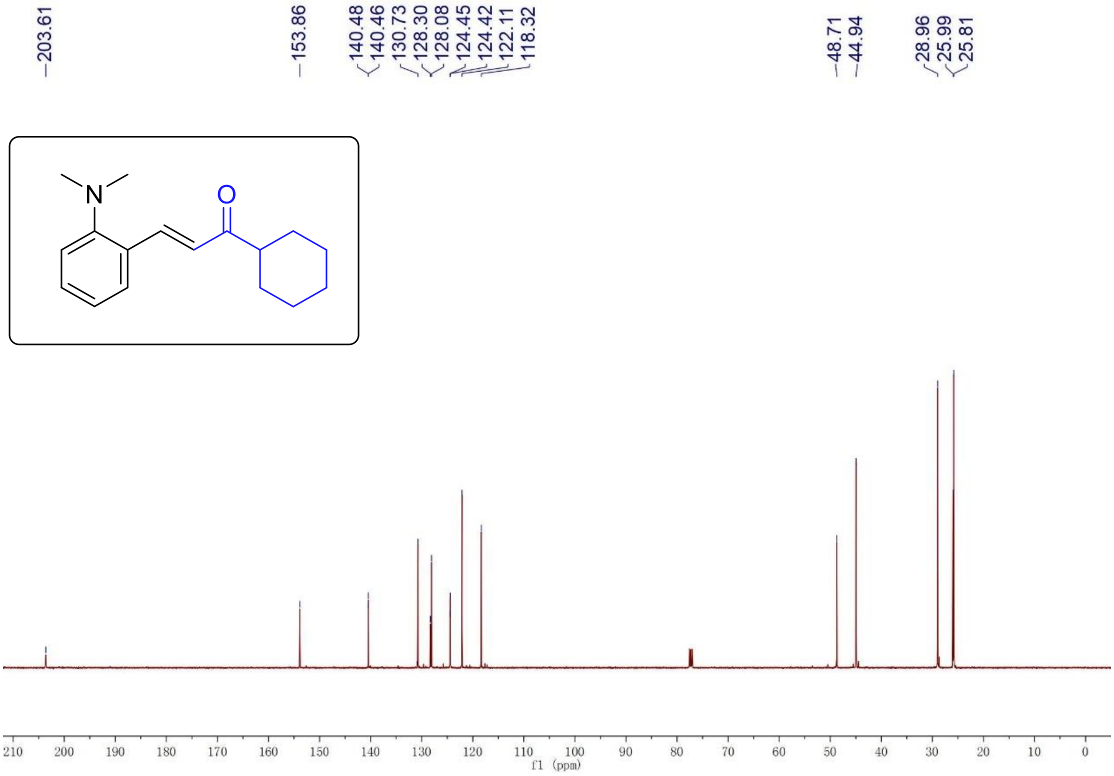

Supplementary Figure 27. $^{1}$H NMR and $^{13}$C NMR spectrum of **1z**.

**E)-1-((1S,4S)-bicyclo[2.2.1]hept-5-en-2-yl)-3-(2-(dimethylamino)phenyl)prop-2-en-1-one (1aa)**

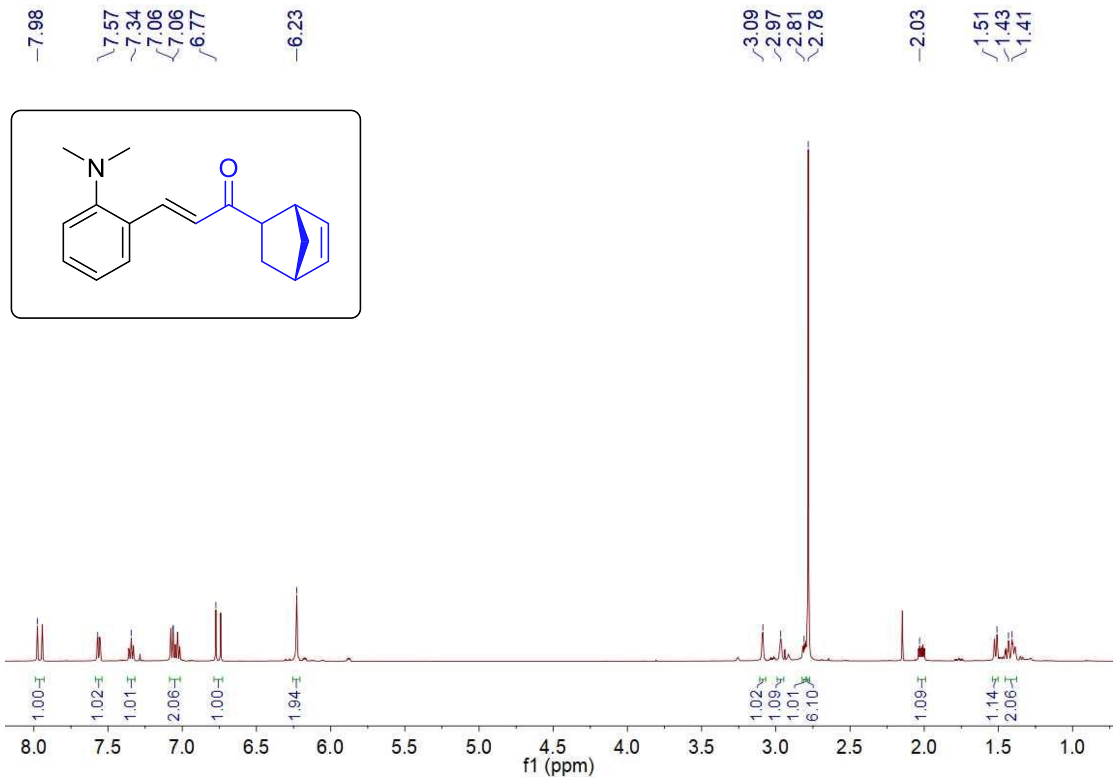


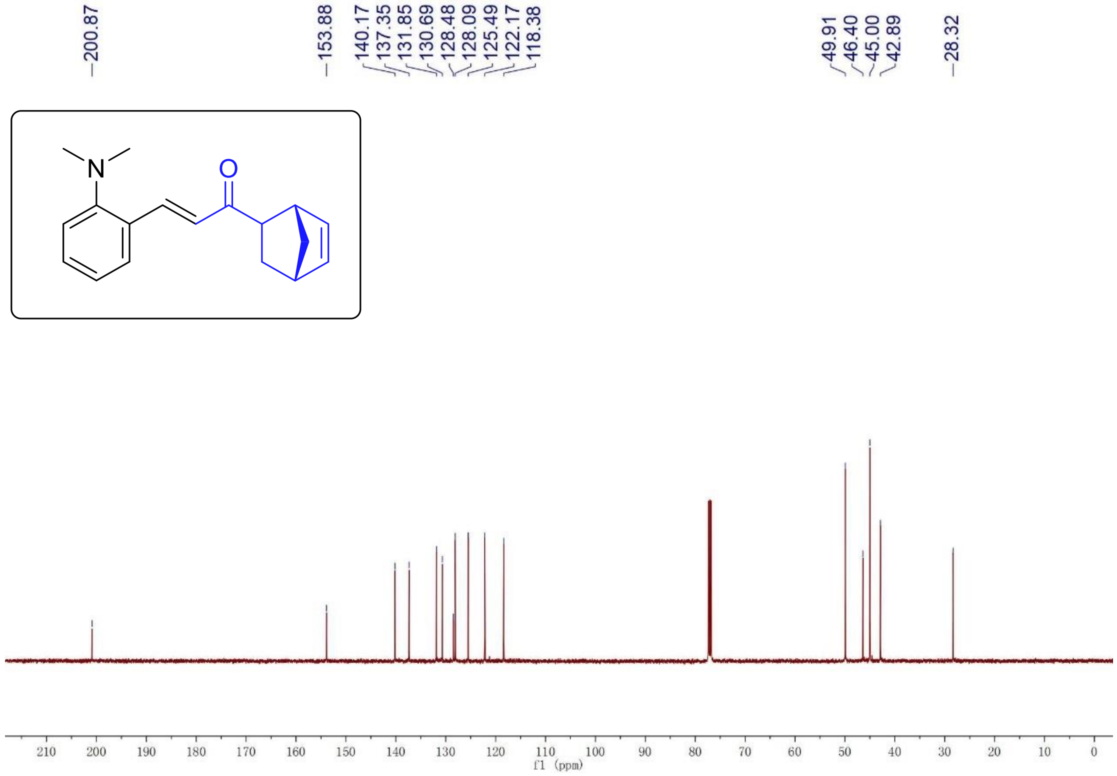


Supplementary Figure 28. $^{1}$H NMR and $^{13}$C NMR spectrum of **1aa**.

**E)-2-(2-(dimethylamino)benzylidene)cyclobutan-1-one (1ab)**

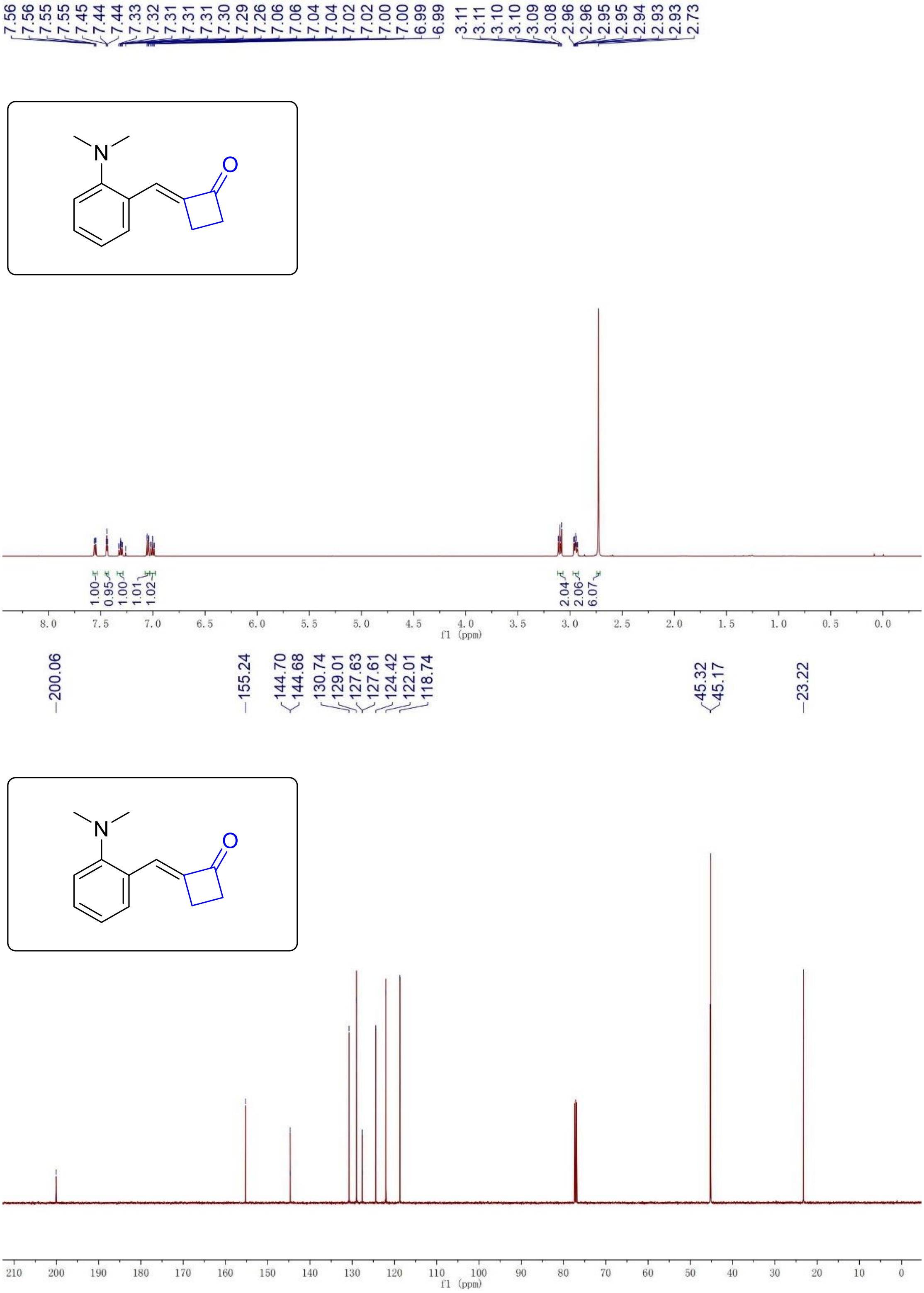


Supplementary Figure 29. $^{1}H$ NMR and $^{13}C$ NMR spectrum of **1ab**.

**(E)-3-(2-(dimethylamino)benzylidene)tetrahydro-4H-pyran-4-one (1ac)**

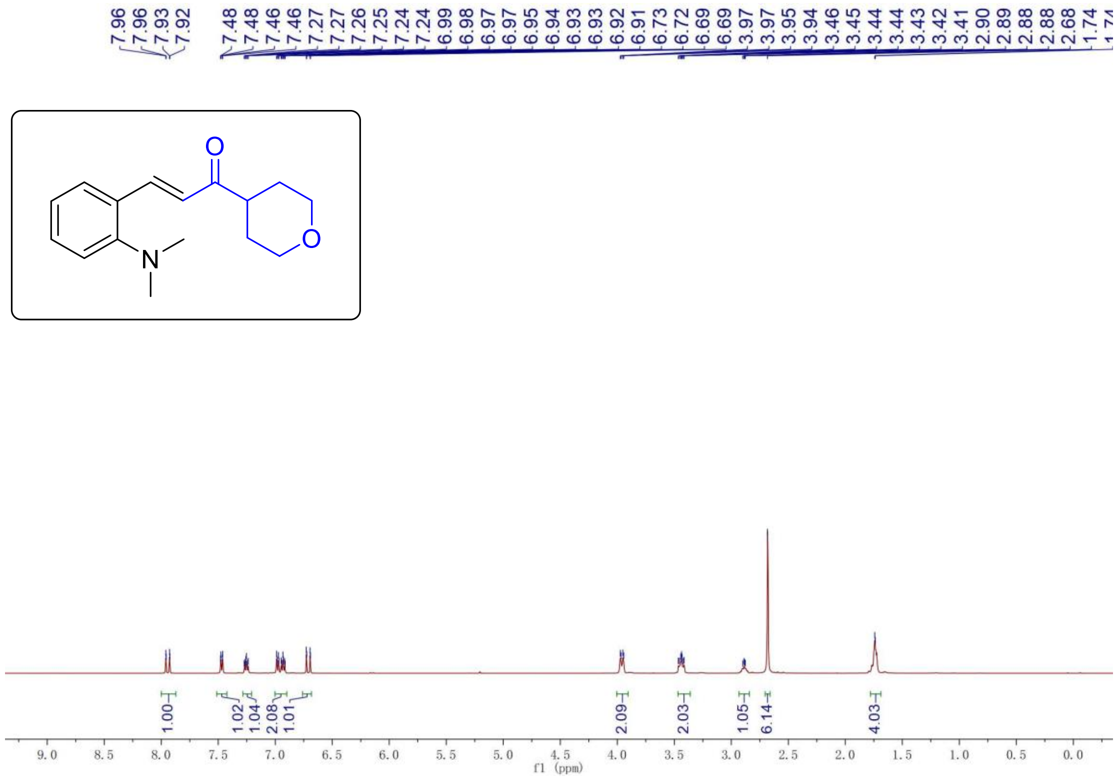


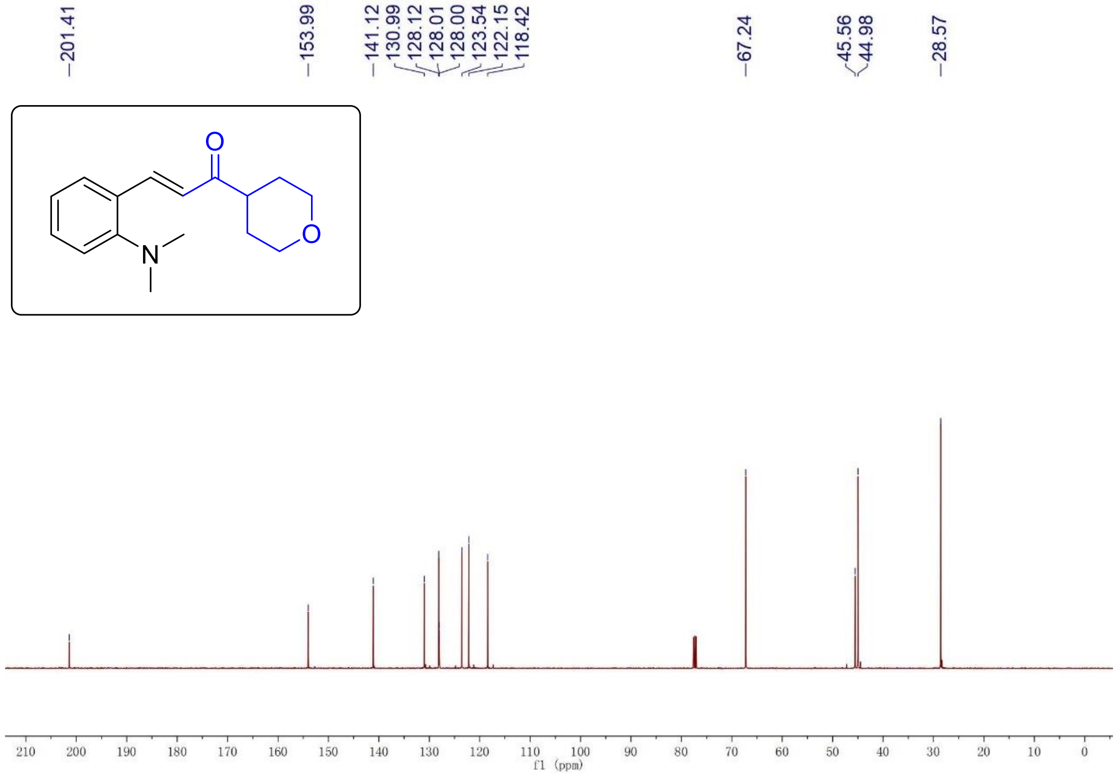


Supplementary Figure 30. $^1$H NMR and $^{13}$C NMR spectrum of **1ac**.

**(E)-2-(2-(dimethylamino)benzylidene)cyclooctan-1-one (1ad)**

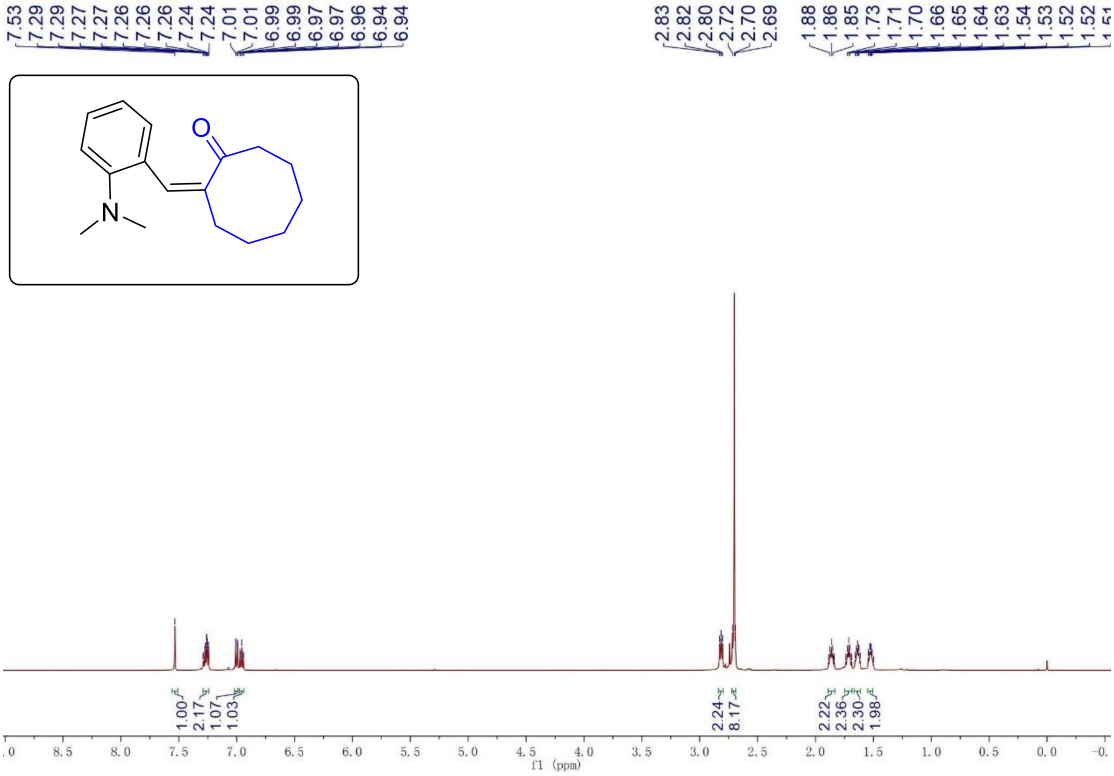


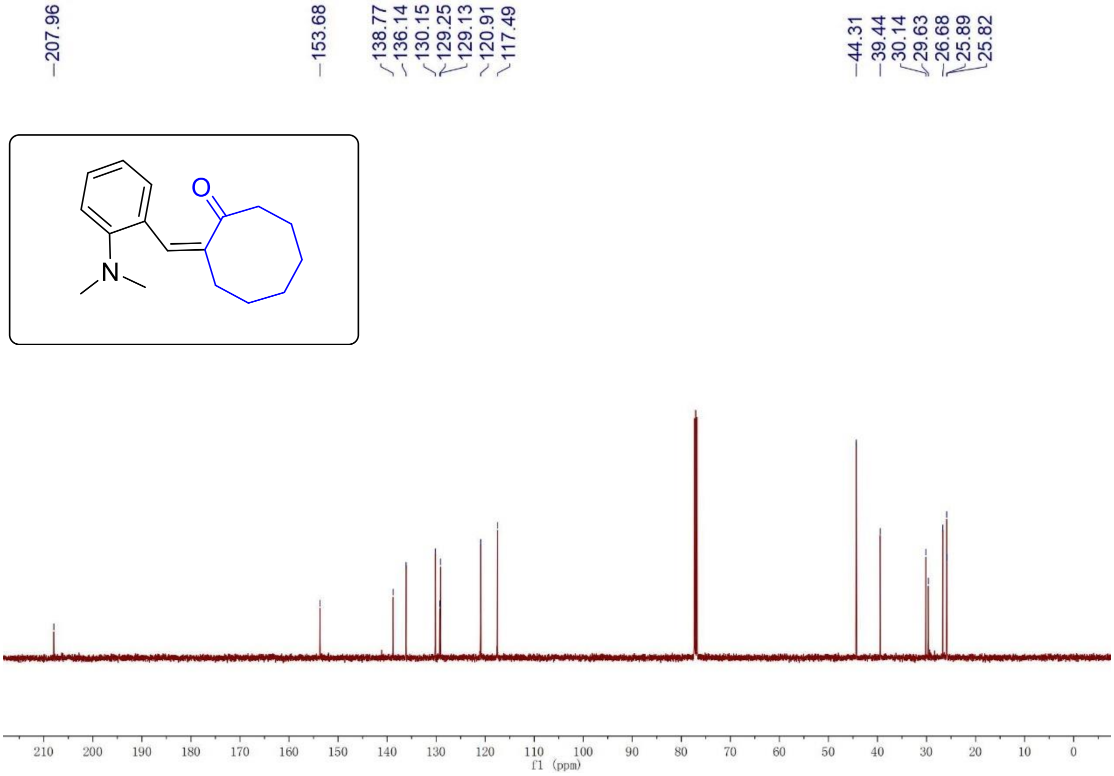


Supplementary Figure 31. $^{1}$H NMR and $^{13}$C NMR spectrum of **1ad**.

**(1R,4S)-3-((E)-2-(dimethylamino)benzylidene)bicyclo[2.2.1]heptan-2-one (1ae)**

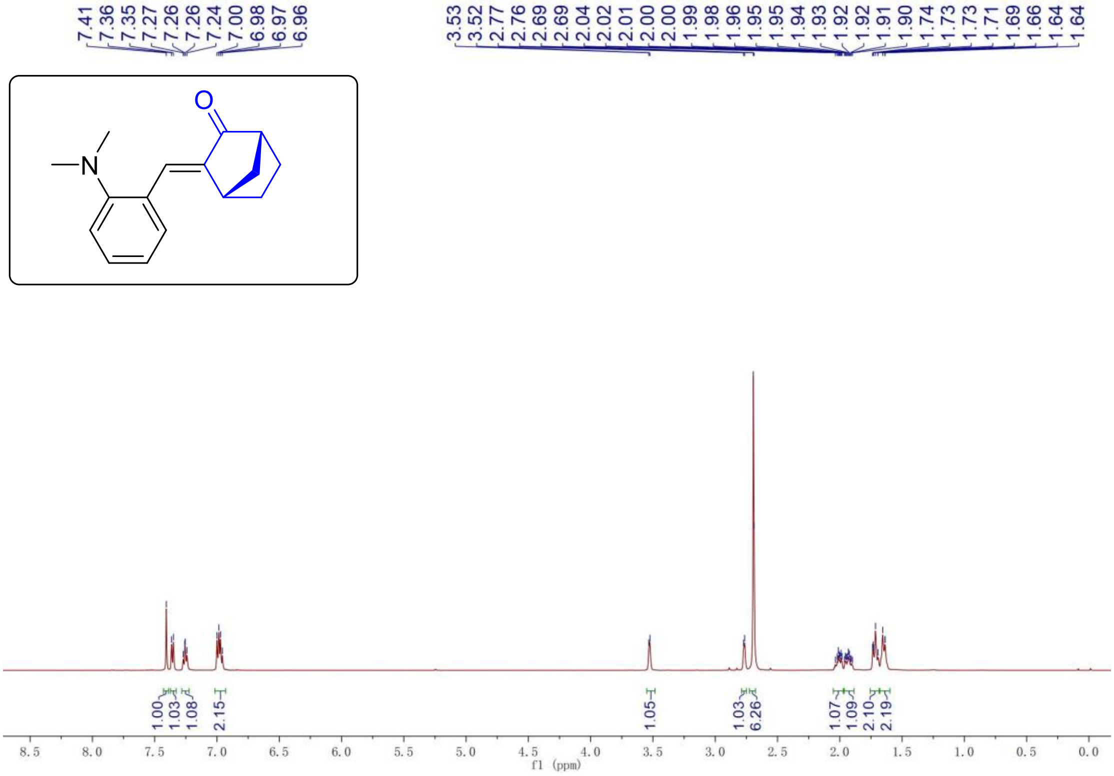


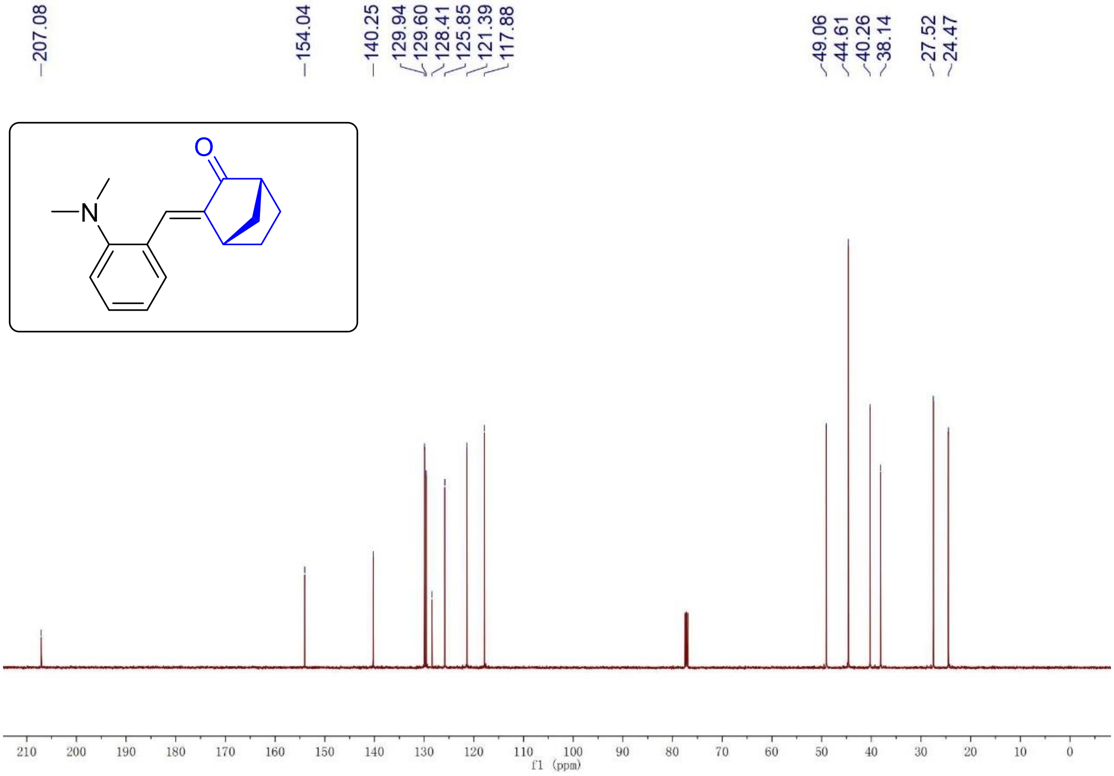


Supplementary Figure 32. $^{1}$H NMR and $^{13}$C NMR spectrum of **1ae**.

**(1E,4E)-1,5-bis(2-(dimethylamino)phenyl)penta-1,4-dien-3-one (1af)**

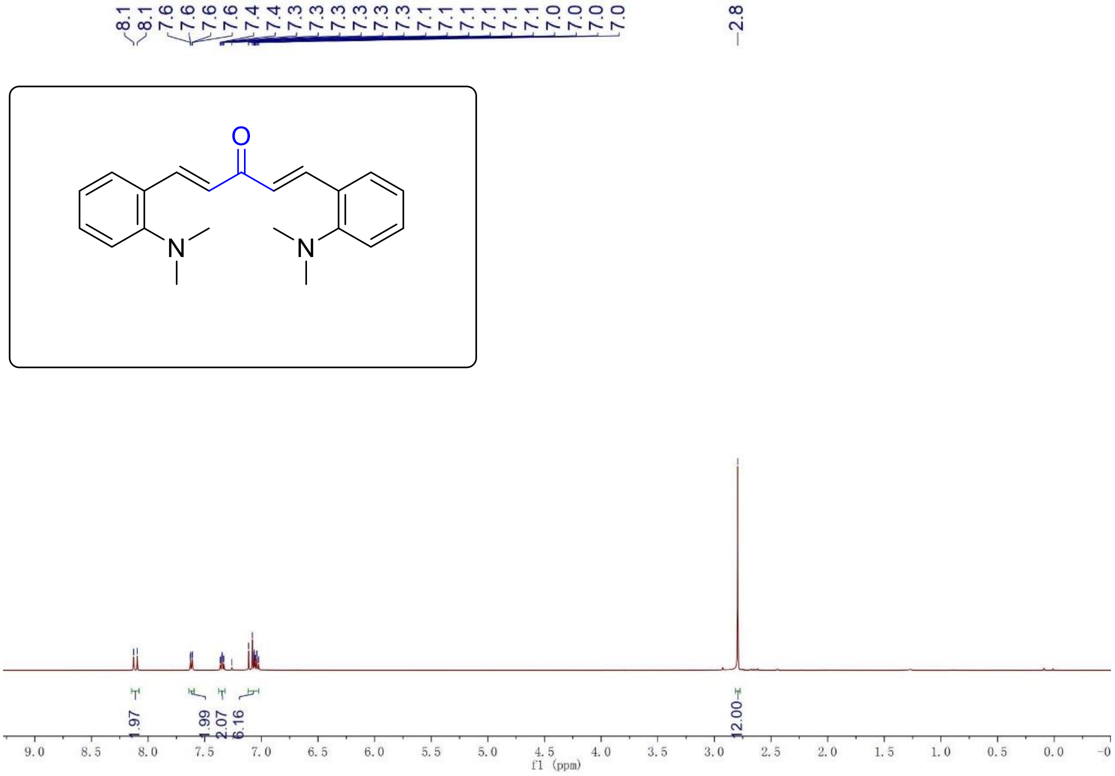


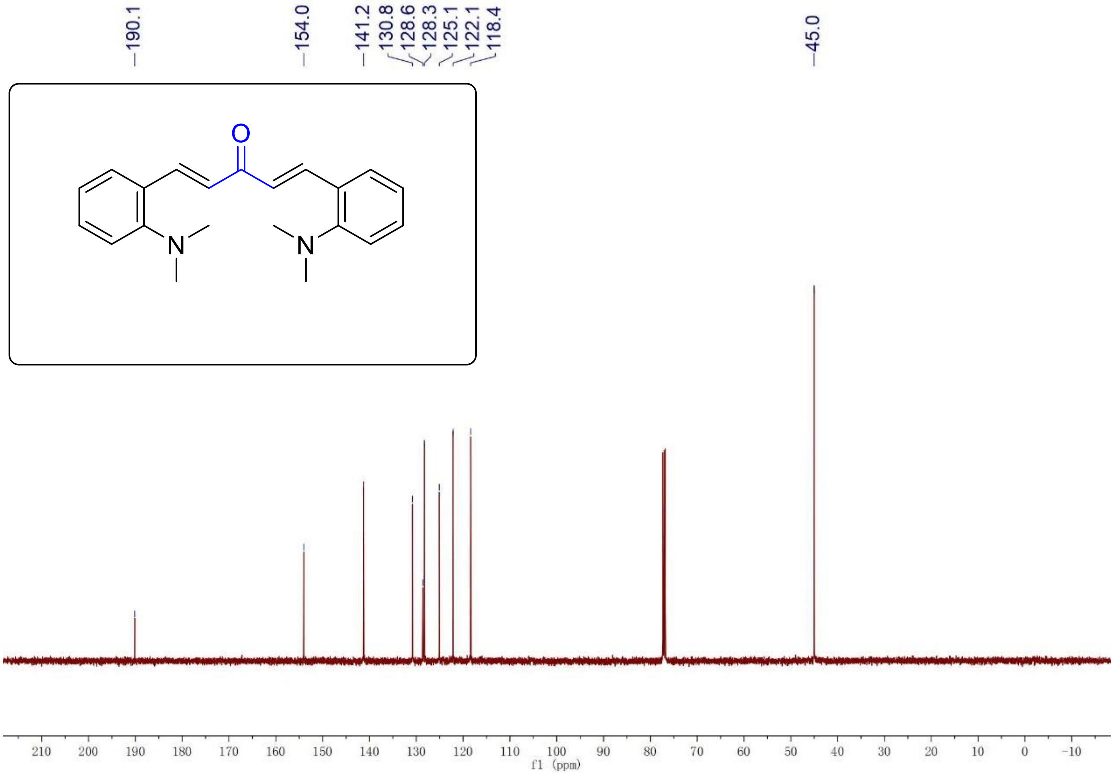


Supplementary Figure 33. $^{1}$H NMR and $^{13}$C NMR spectrum of **1af**.

**(E)-3-(2-(dimethylamino)-5-methylphenyl)-1-phenylprop-2-en-1-one (1ag)**

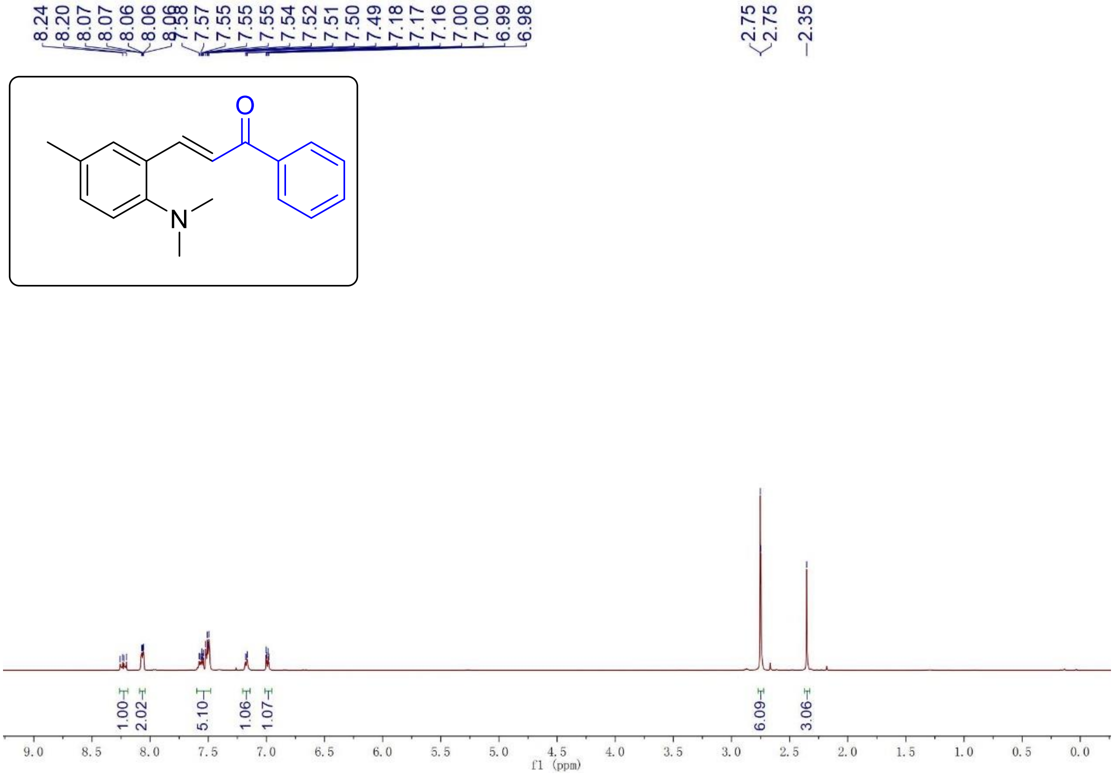


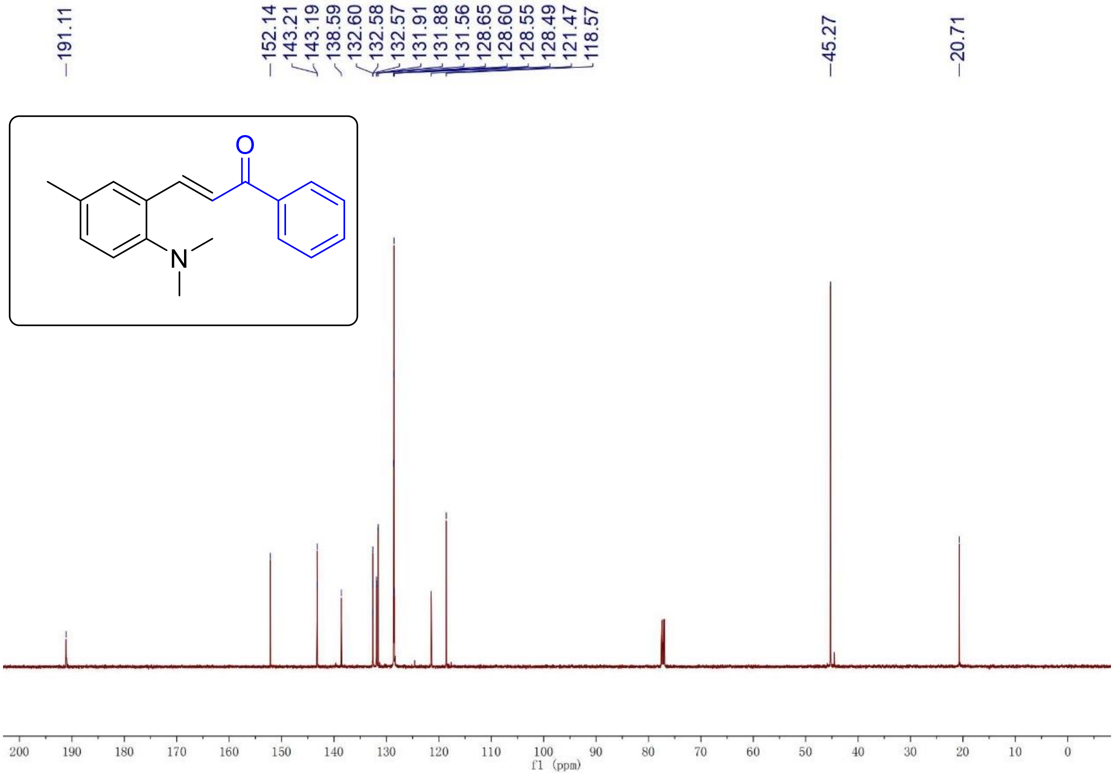


Supplementary Figure 34. $^{1}$H NMR and $^{13}$C NMR spectrum of **1ag**.

**(E)-3-(2-(dimethylamino)-5-(trifluoromethyl)phenyl)-1-phenylprop-2-en-1-one (1ah)**

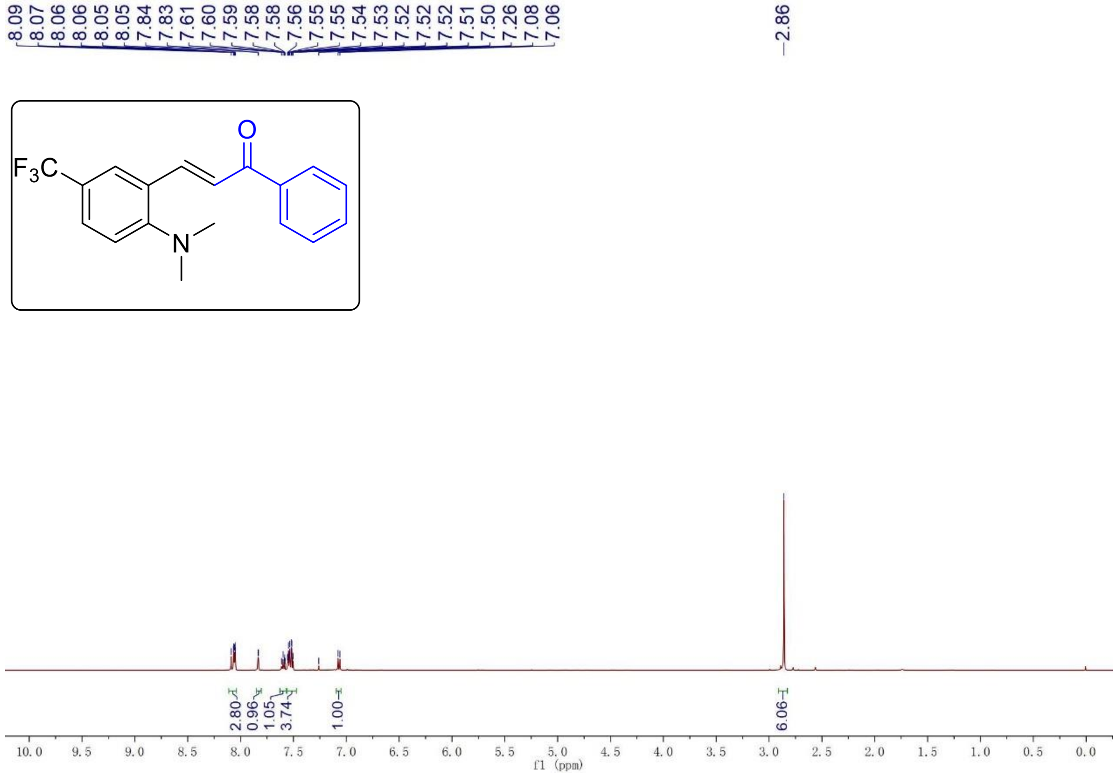


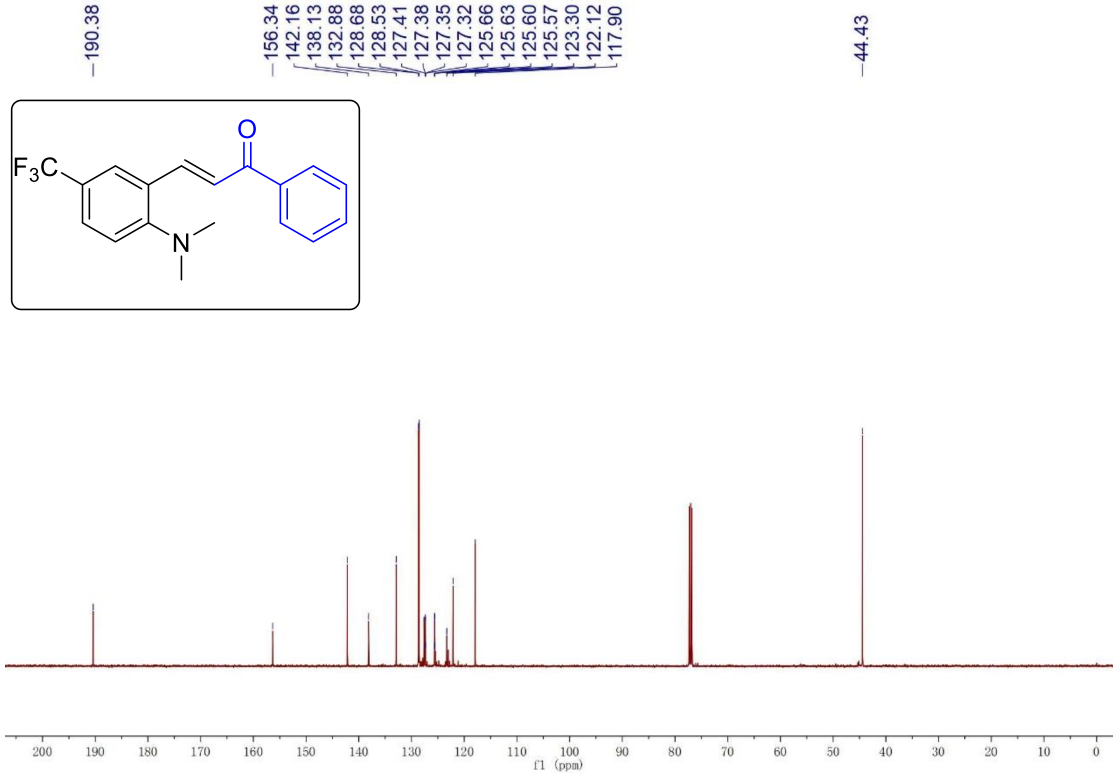

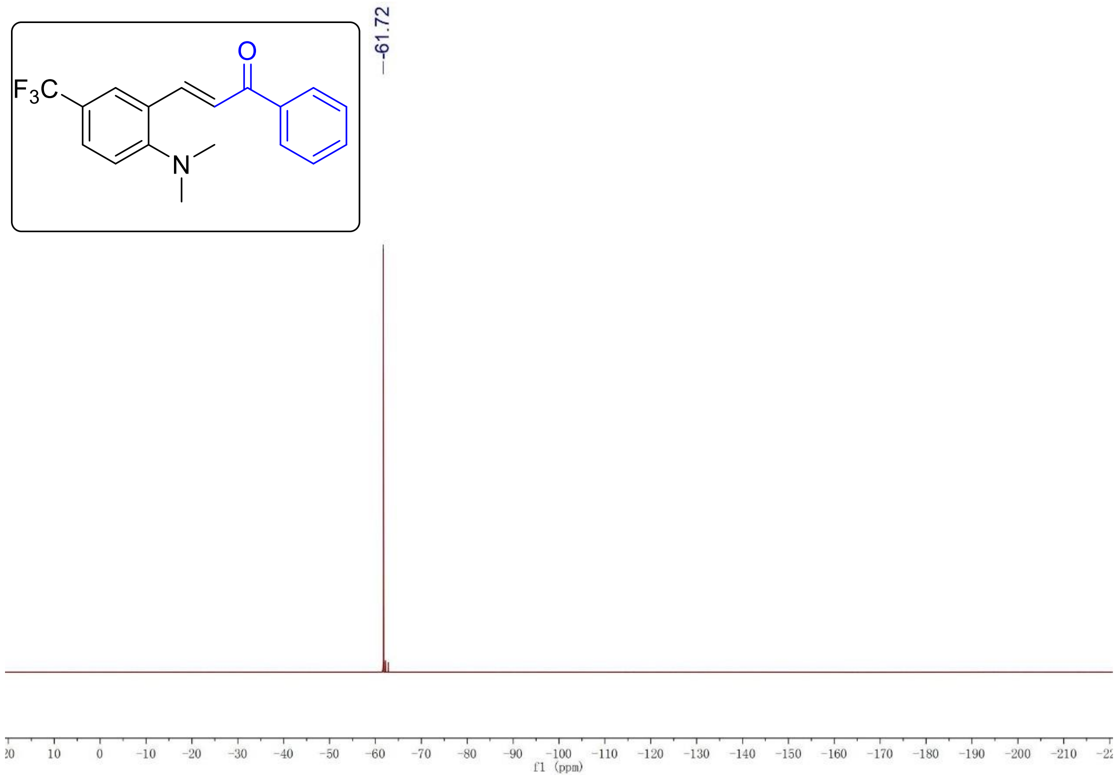


Supplementary Figure 35. $^{1}H$ NMR , $^{13}C$ NMR and $^{19}F$ NMR spectrum of **1ah**.

**(E)-3-(5-bromo-2-(dimethylamino)phenyl)-1-phenylprop-2-en-1-one (1ai)**

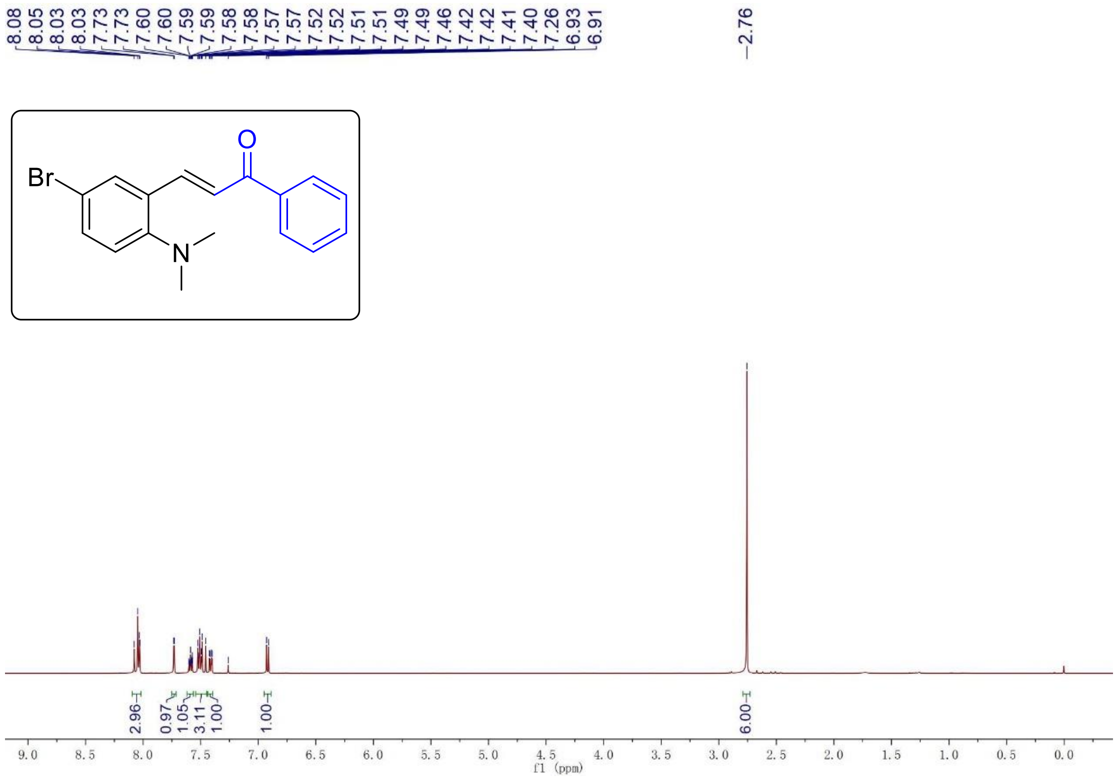


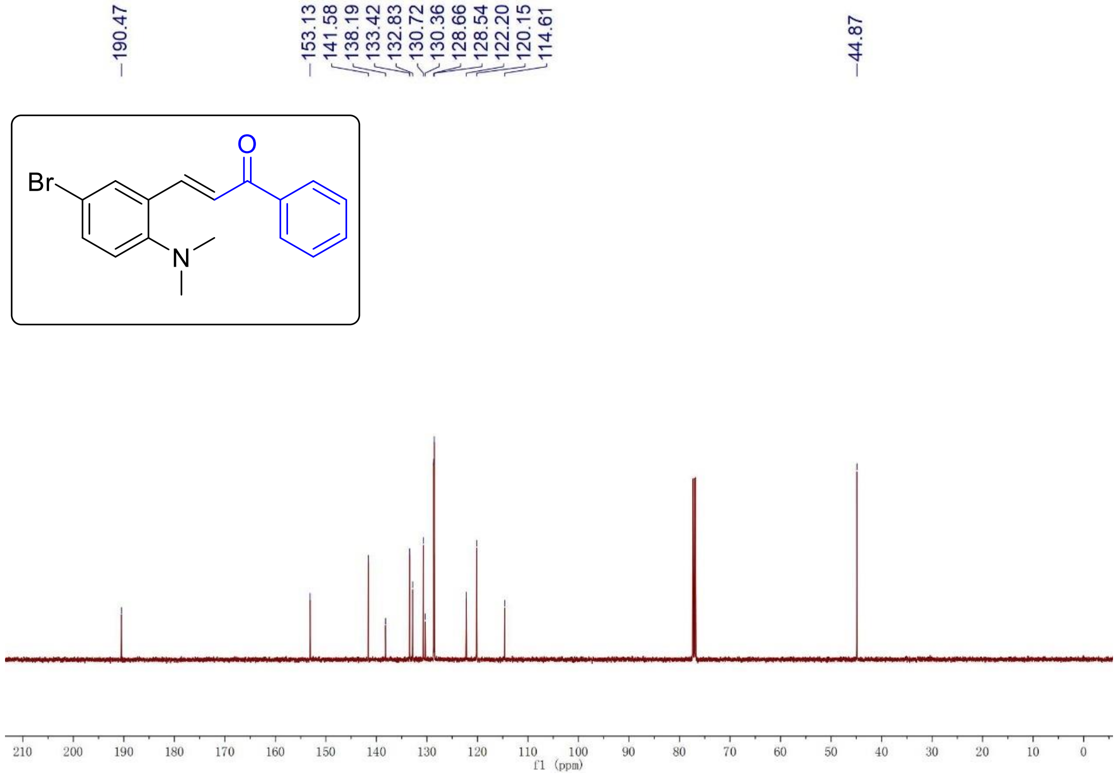


Supplementary Figure 36. $^{1}$H NMR and $^{13}$C NMR spectrum of **1ai**.

**(E)-3-(2-(dimethylamino)-4-methylphenyl)-1-phenylprop-2-en-1-one (1aj)**

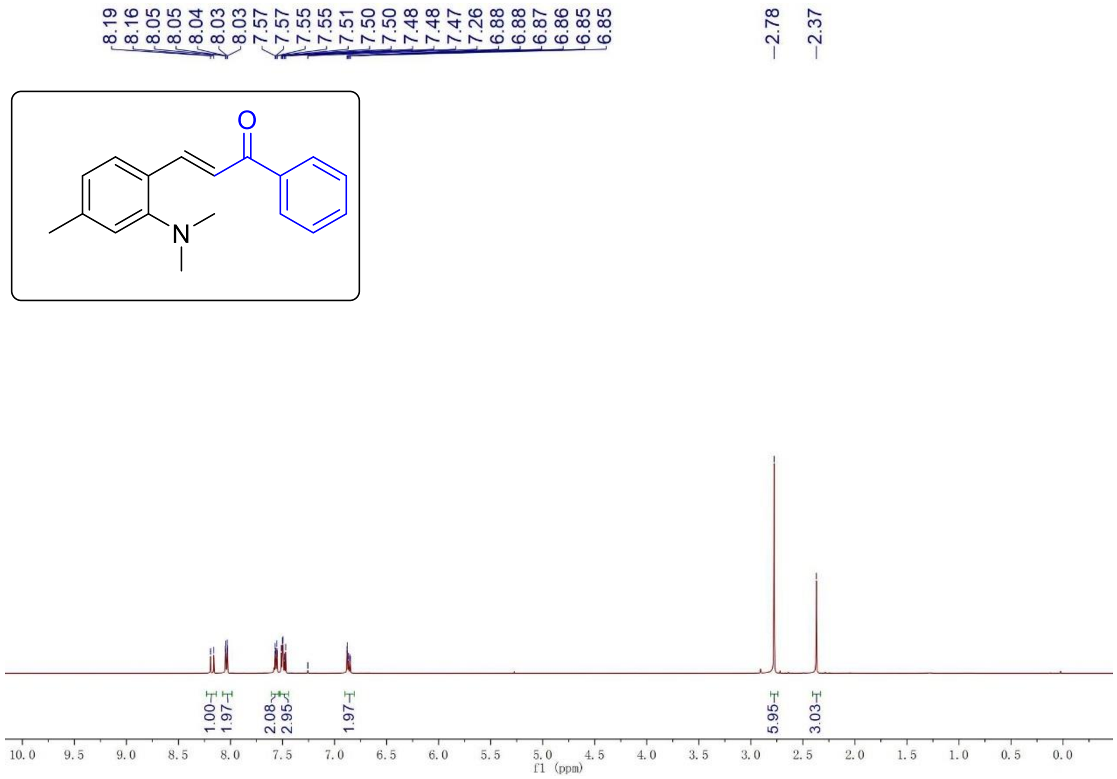

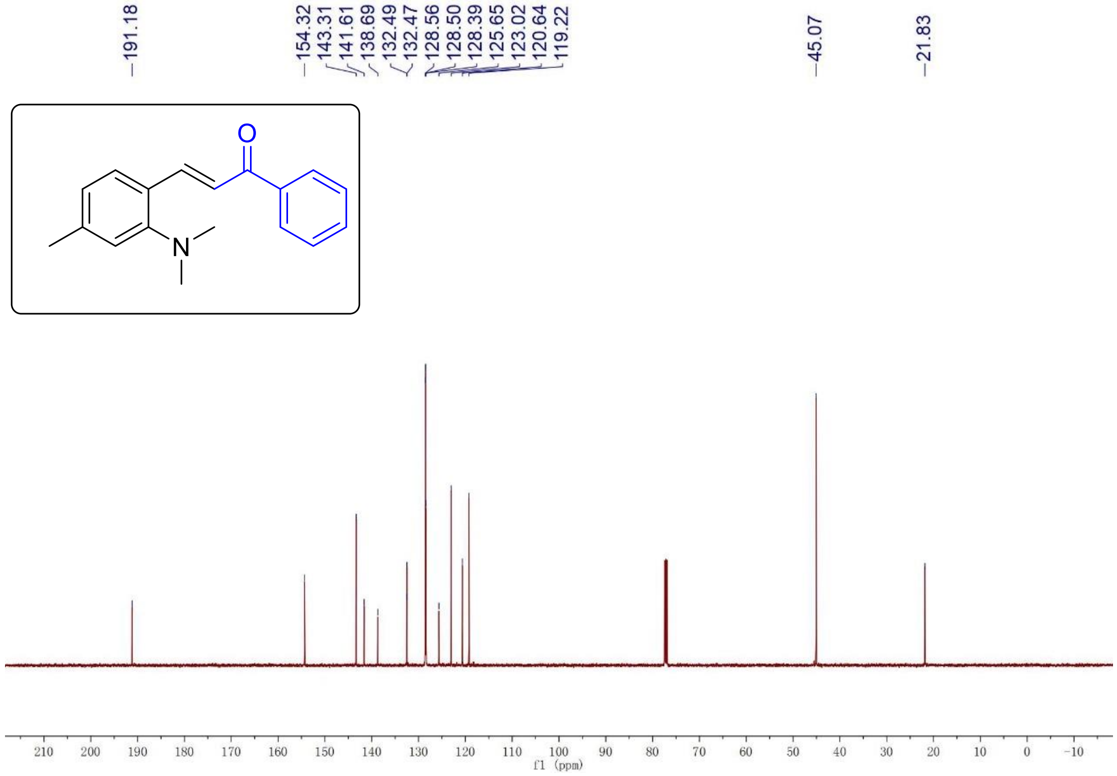

Supplementary Figure 37. $^{1}$H NMR and $^{13}$C NMR spectrum of **1aj**.

**(E)-3-(2-(dimethylamino)-4-methoxyphenyl)-1-phenylprop-2-en-1-one (1ak)**

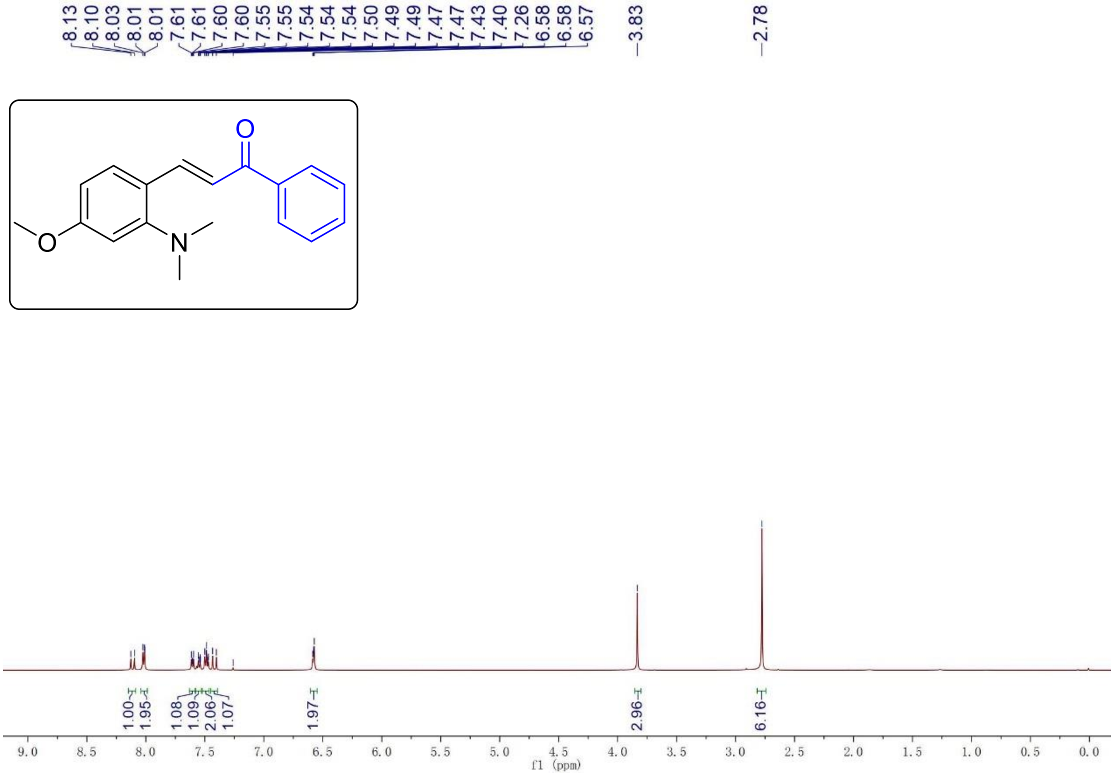


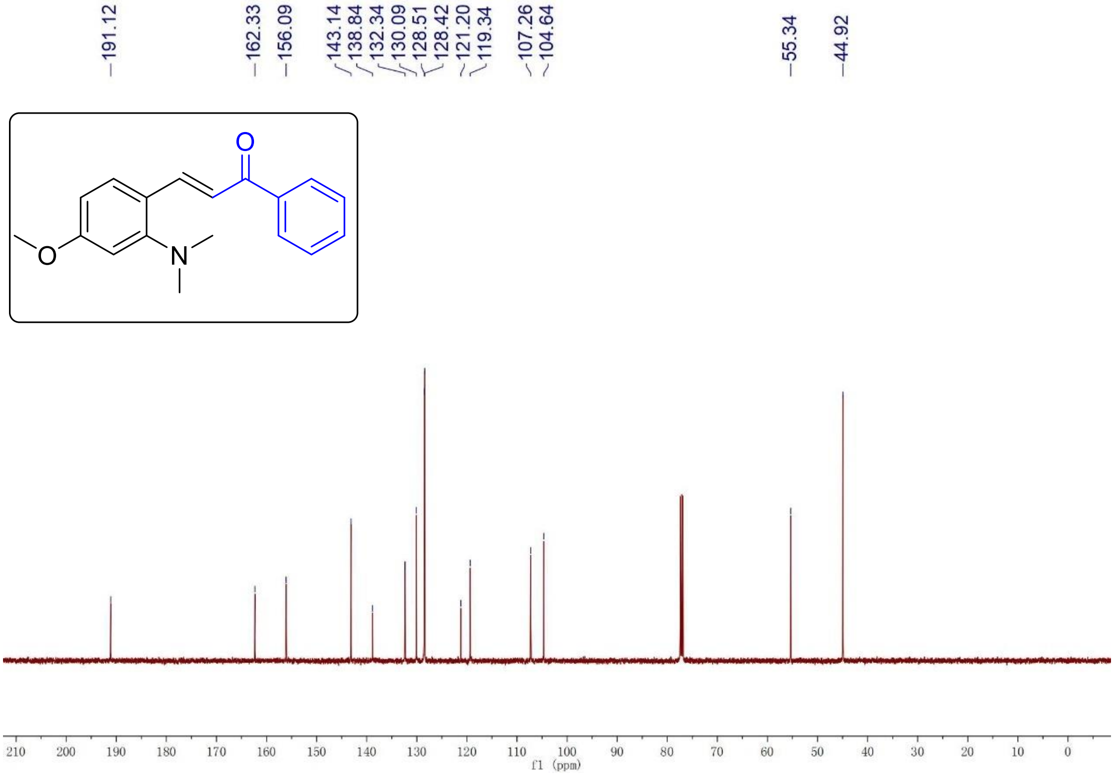


Supplementary Figure 38. $^{1}$H NMR and $^{13}$C NMR spectrum of **1ak**.

**(E)-3-(4-bromo-2-(dimethylamino)phenyl)-1-phenylprop-2-en-1-one (1al)**

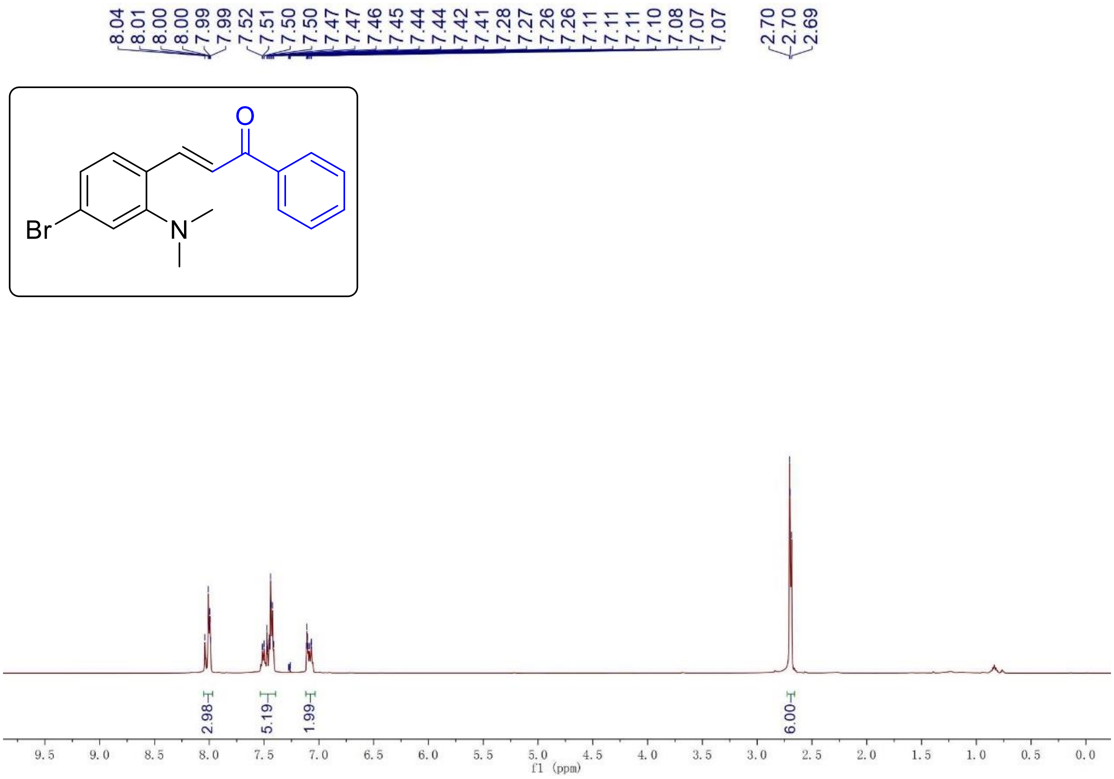


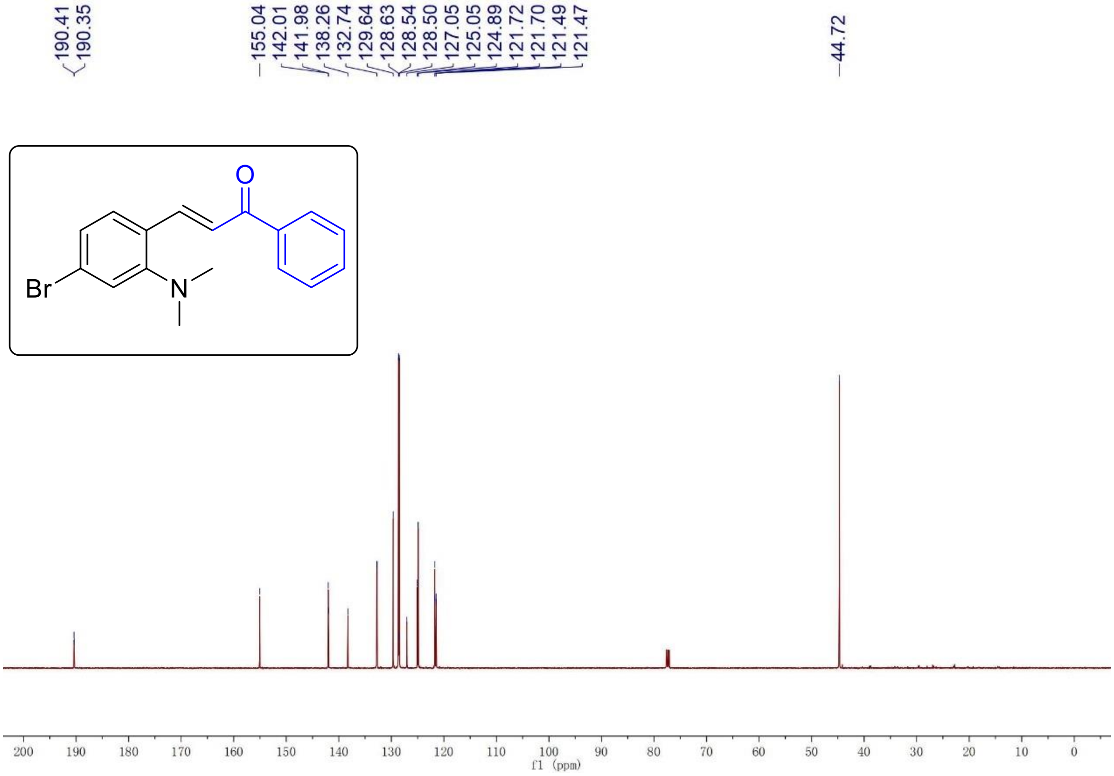


Supplementary Figure 39. $^{1}$H NMR and $^{13}$C NMR spectrum of **1al**.

**(E)-3-(2-(dimethylamino)-3-fluorophenyl)-1-phenylprop-2-en-1-one(1am)**

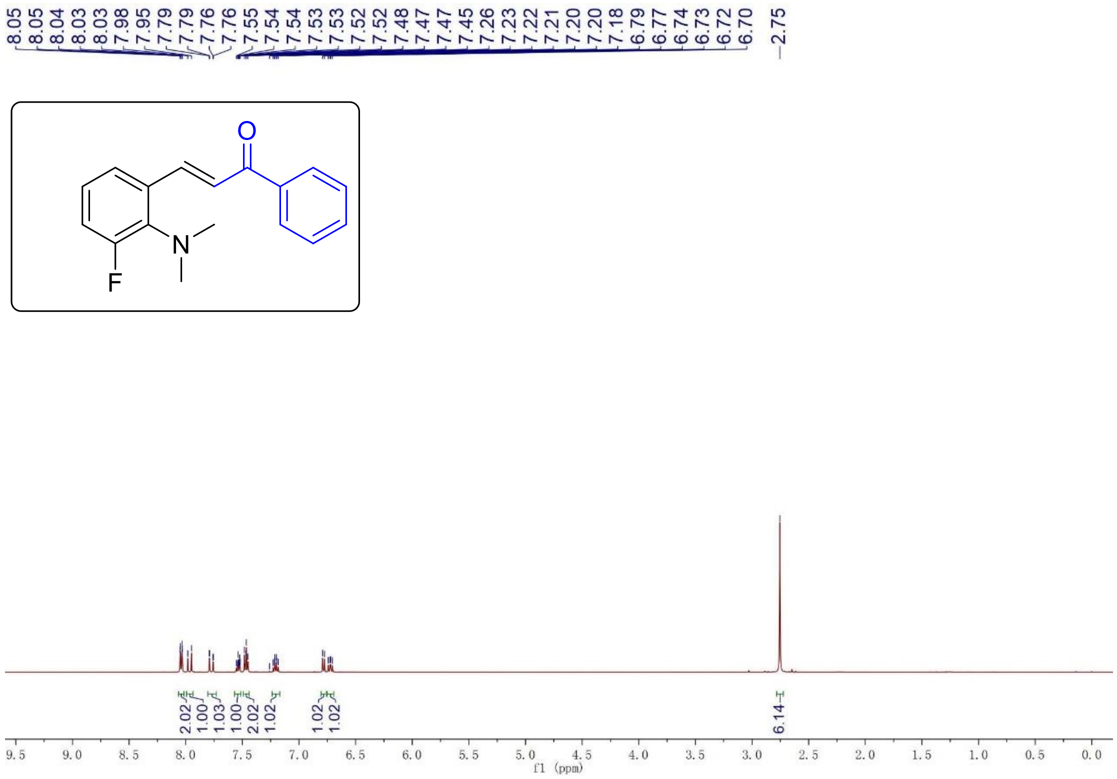

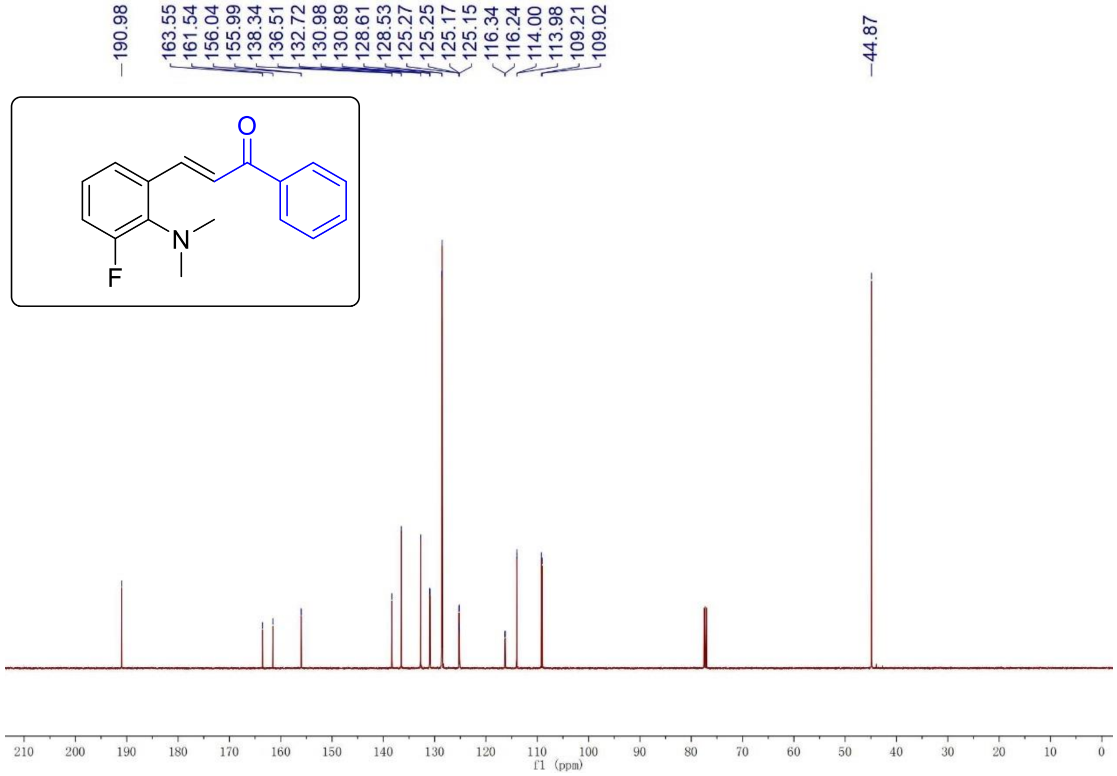

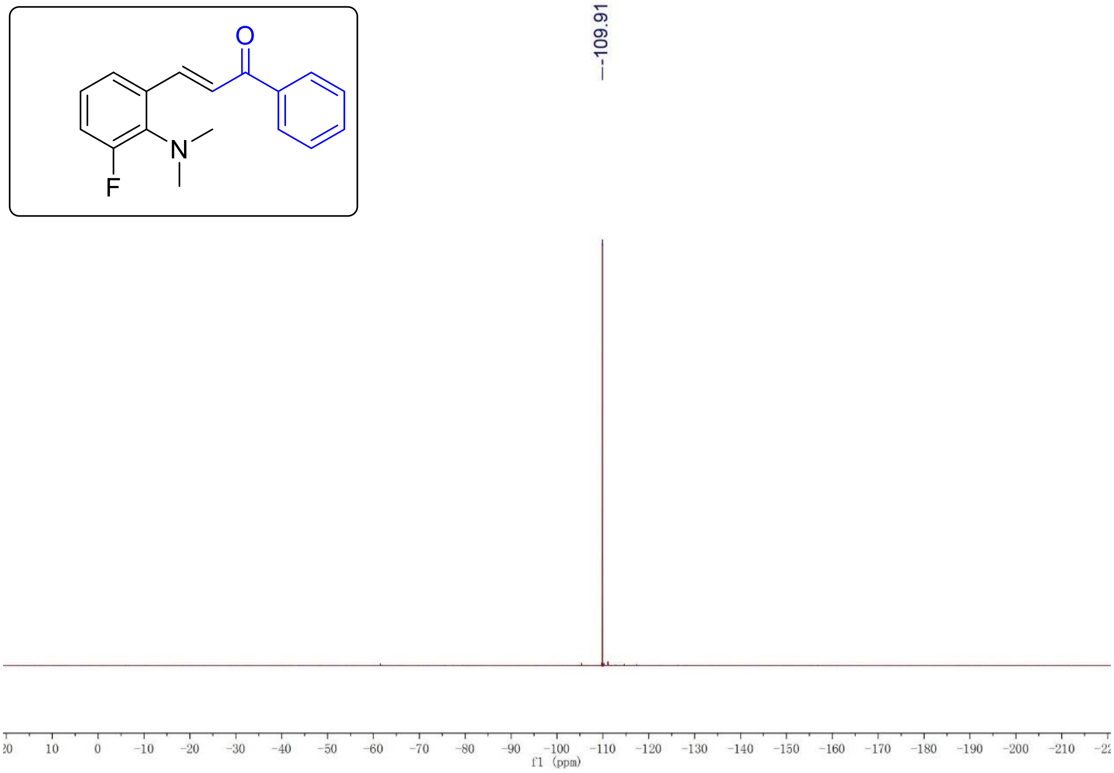


Supplementary Figure 40. $^{1}H$ NMR $^{13}C$ NMR and $^{19}F$ NMR spectrum of **1am**.

**(E)-3-(3-chloro-2-(dimethylamino)phenyl)-1-phenylprop-2-en-1-one (1an) 2:1**

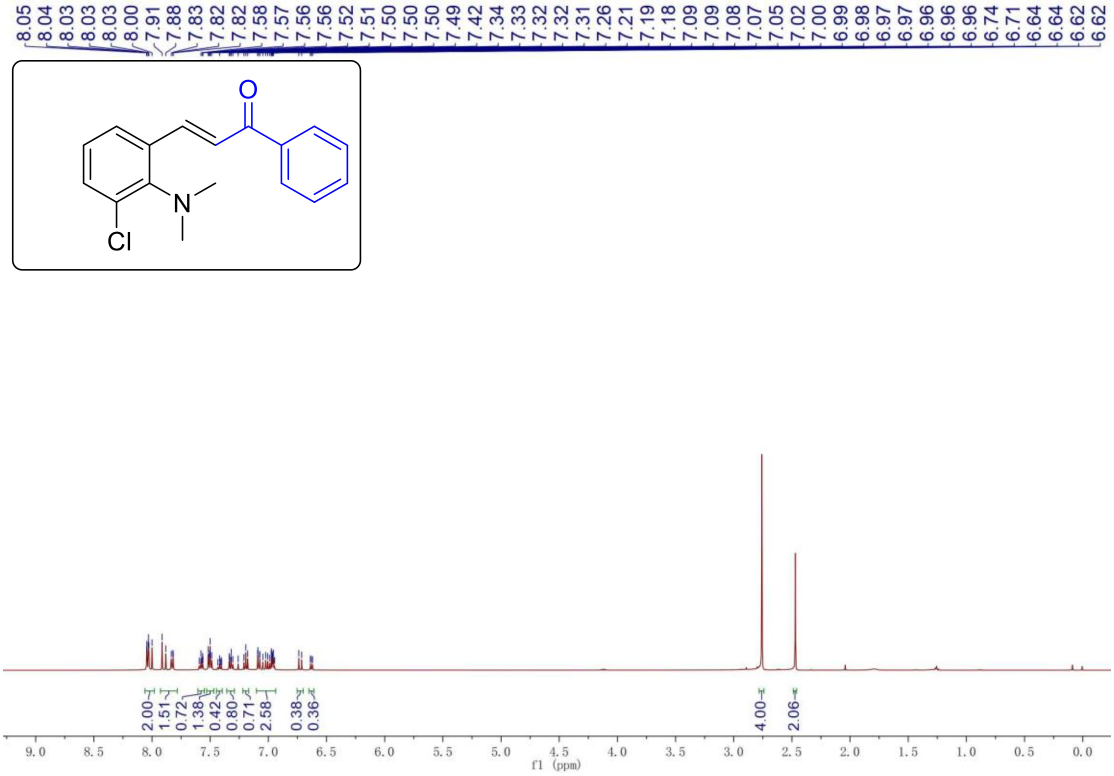

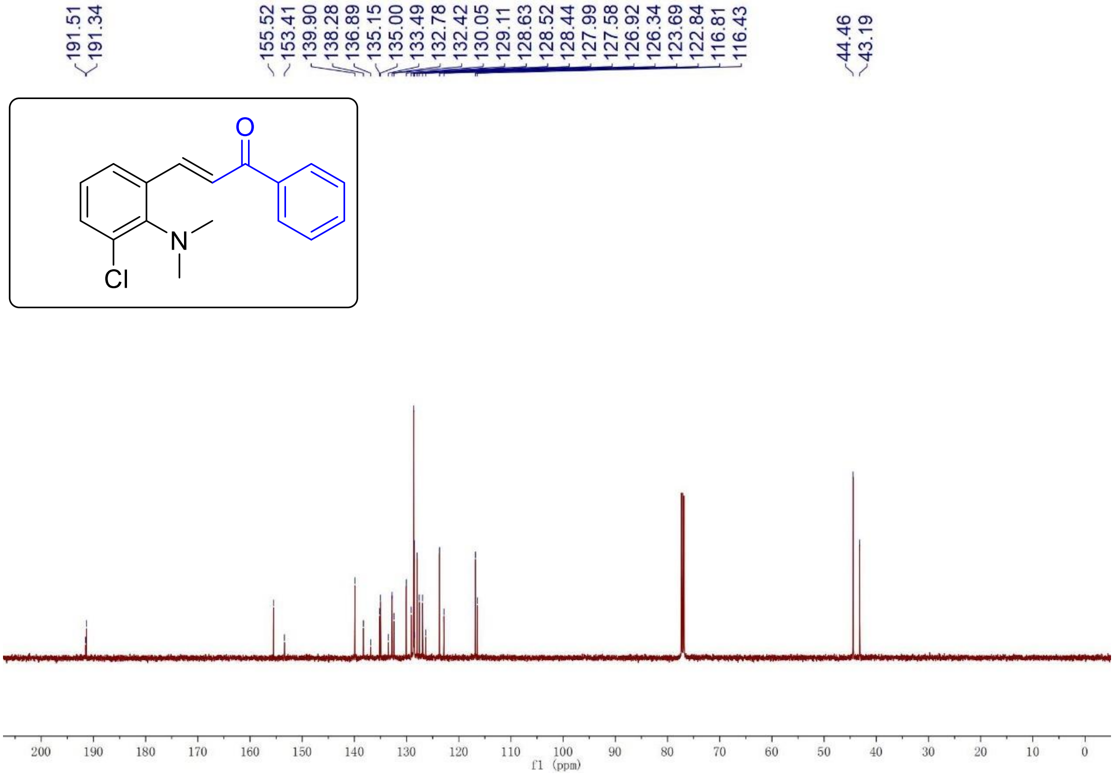

Supplementary Figure 41. $^{1}$H NMR and $^{13}$C NMR spectrum of **1an**.

**(E)-3-(2-(ethyl(methyl)amino)phenyl)-1-phenylprop-2-en-1-one (1ao)**

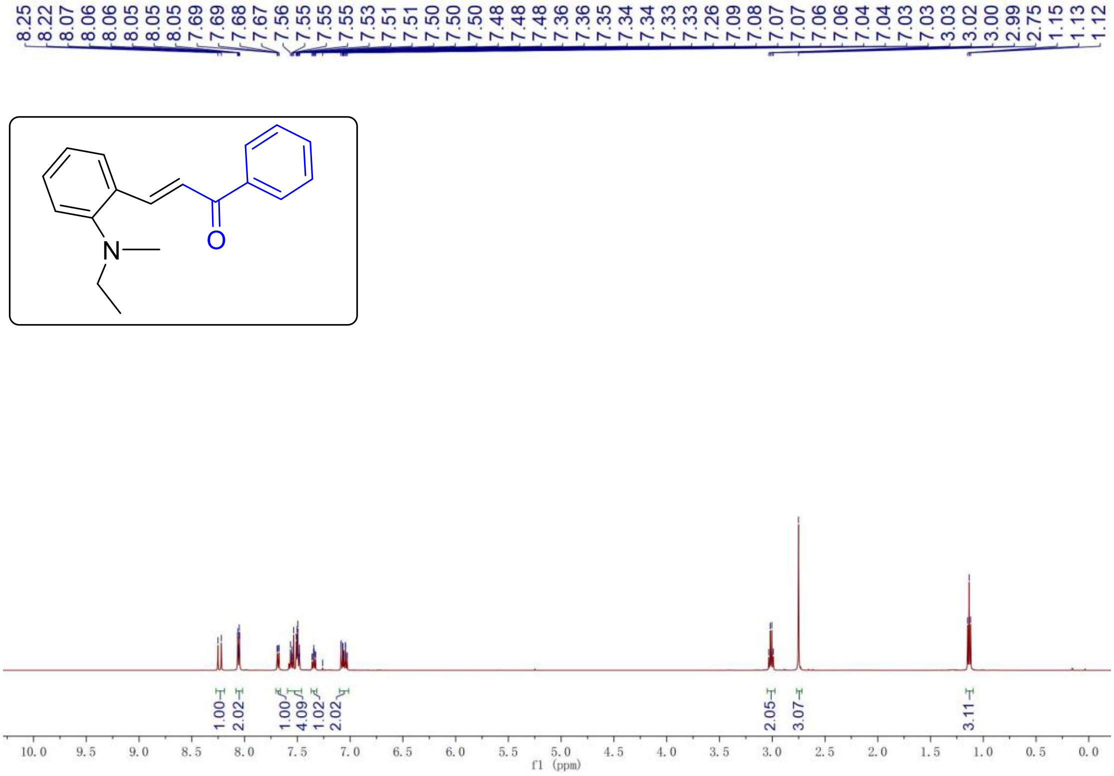


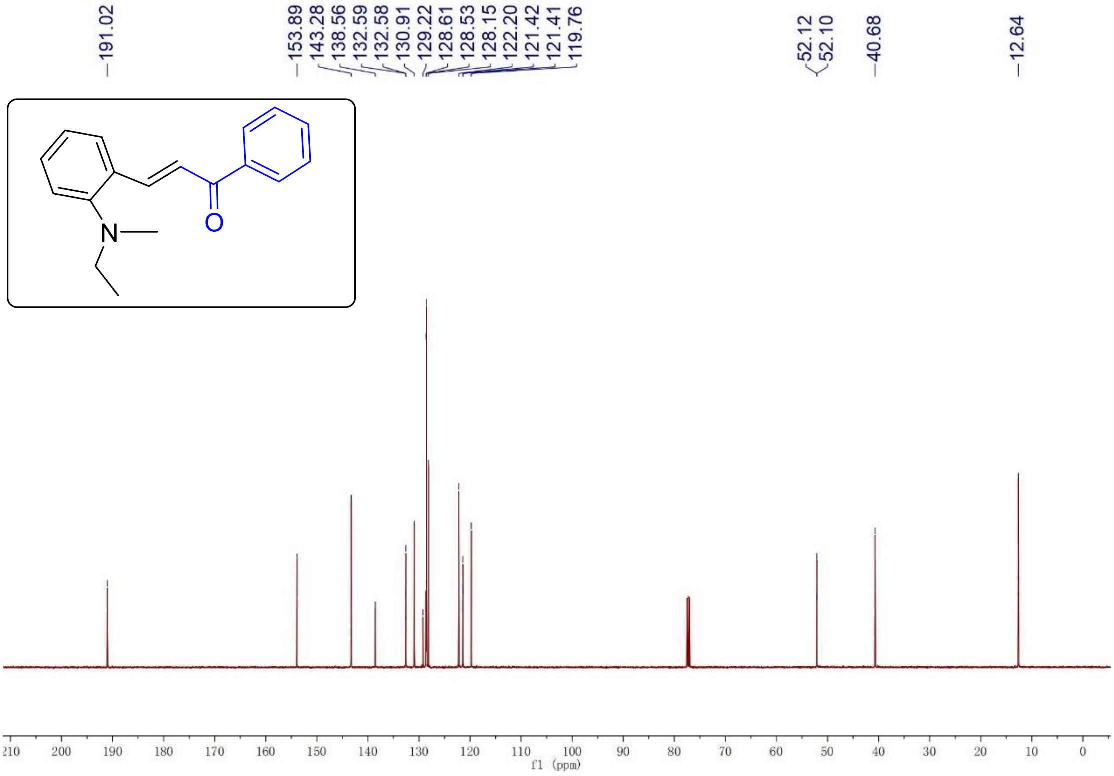


Supplementary Figure 42. $^{1}$H NMR and $^{13}$C NMR spectrum of **1ao**.

**(E)-3-(2-(allyl(methyl)amino)phenyl)-1-phenylprop-2-e (1ap)**

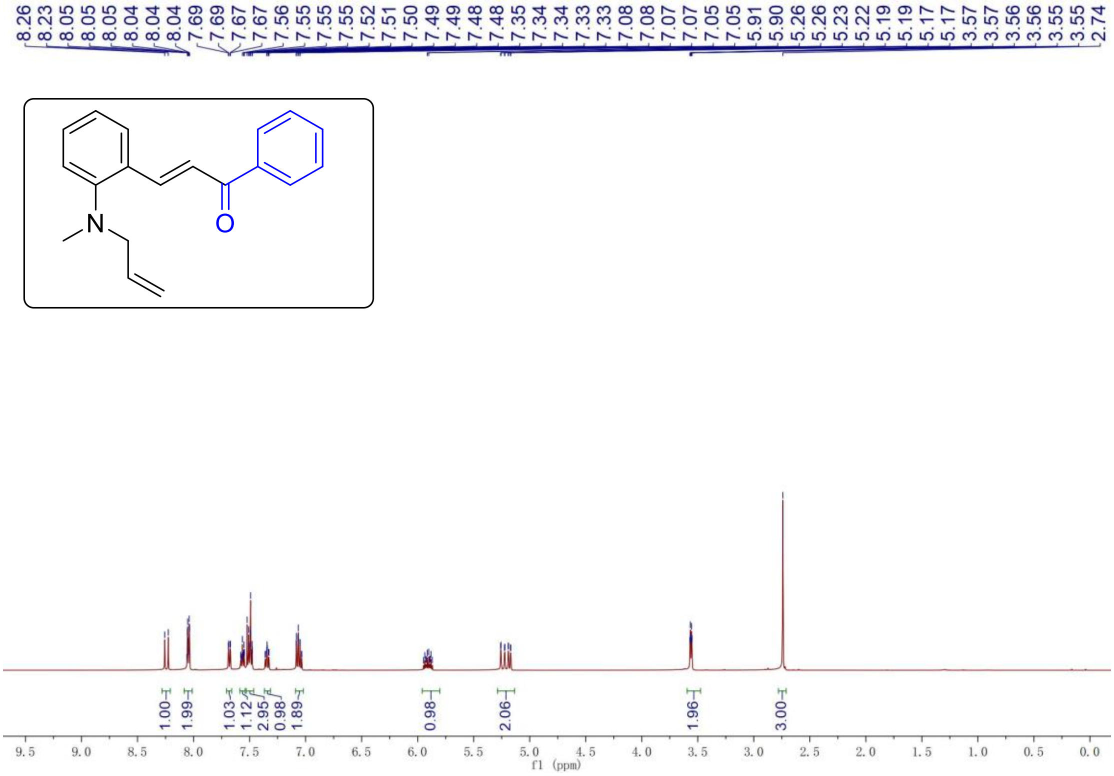


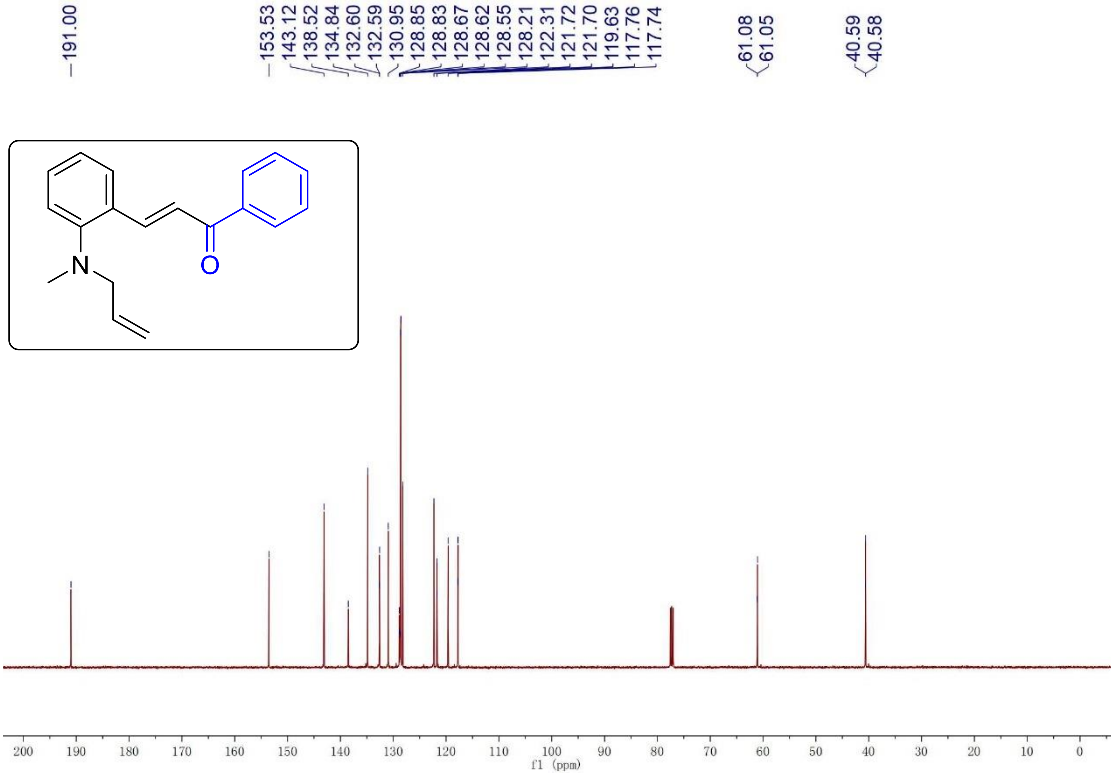


Supplementary Figure 43. $^{1}$H NMR and $^{13}$C NMR spectrum of **1ap**.

**methyl (E)-3-(2-(dimethylamino)phenyl)acrylate (4a)**

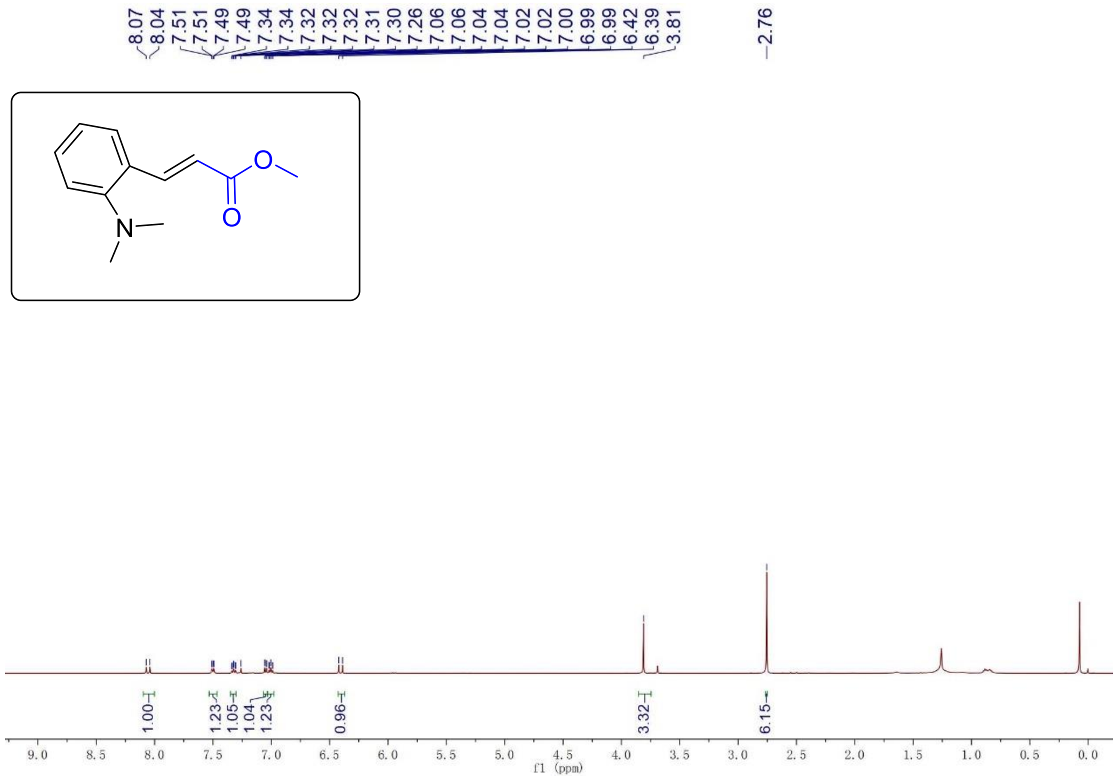


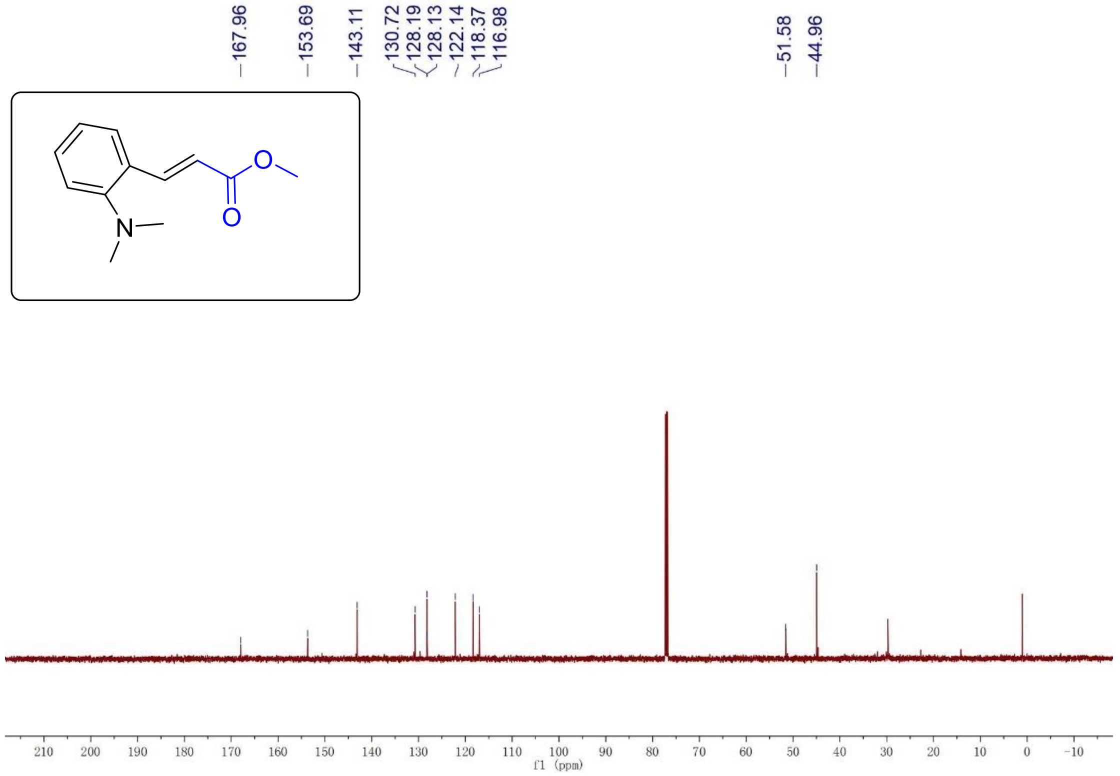


Supplementary Figure 44. $^{1}$H NMR and $^{13}$C NMR spectrum of **4a**.

**ethyl (E)-3-(2-(dimethylamino)phenyl)acrylate (4b)**

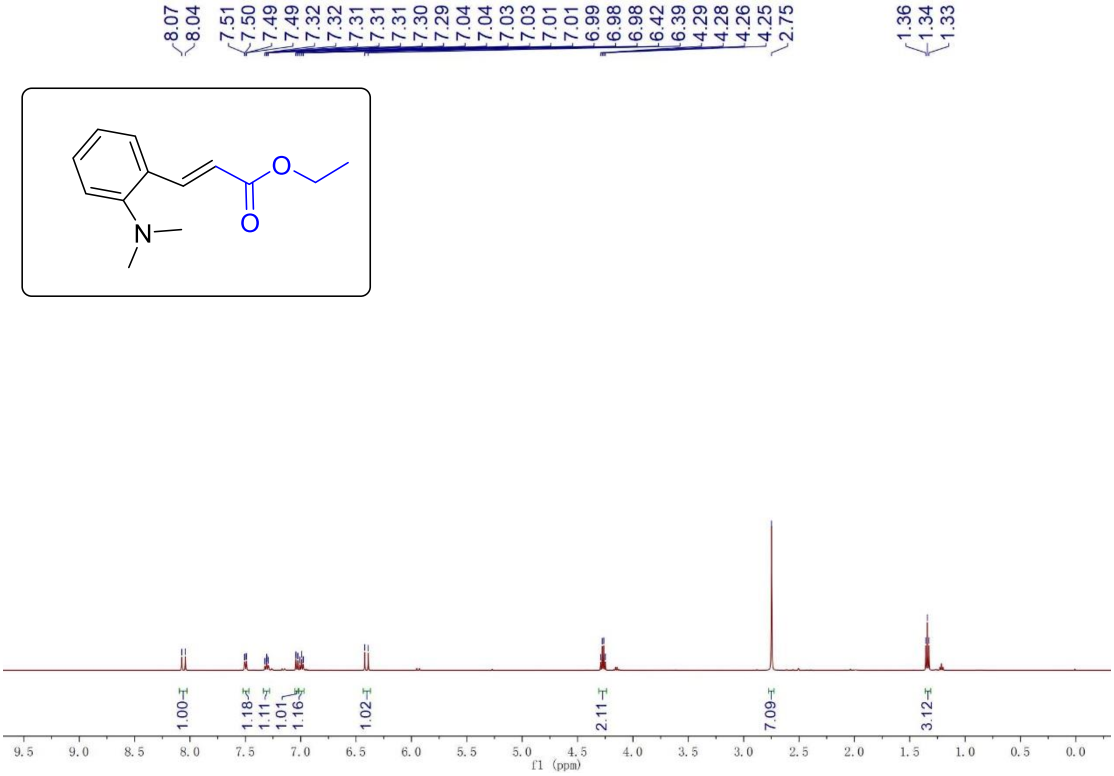

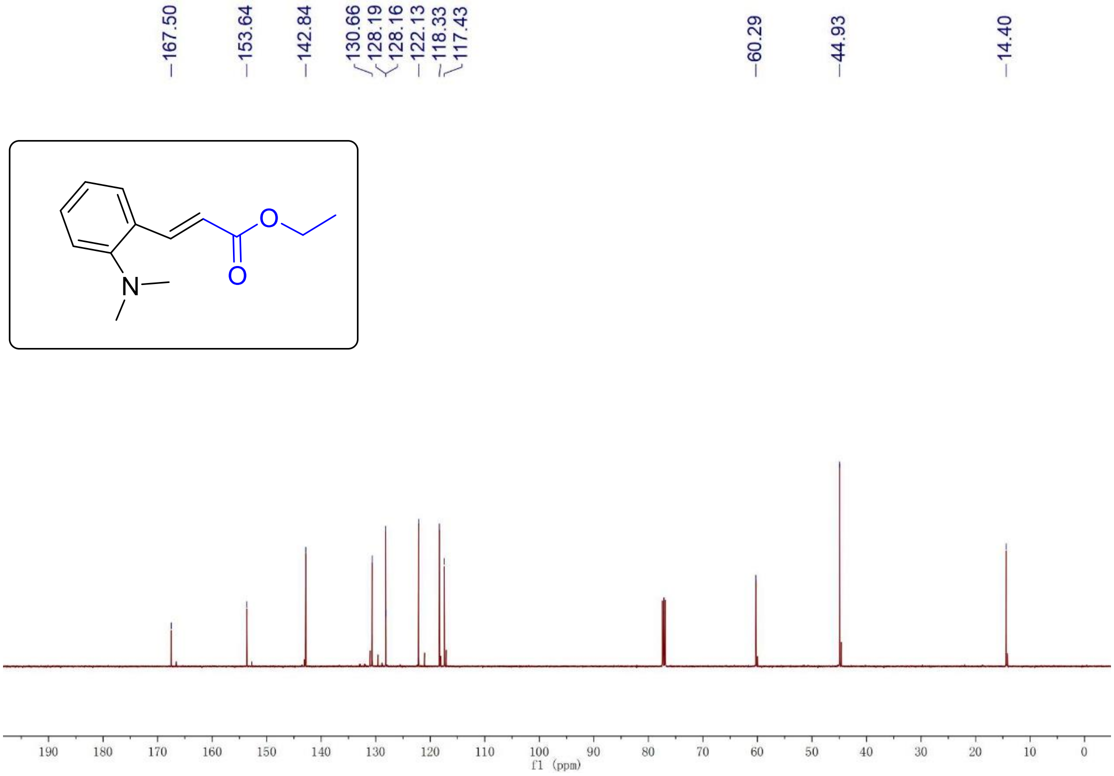

Supplementary Figure 45. $^{1}$H NMR and $^{13}$C NMR spectrum of **4b**.

**tert-butyl (E)-3-(2-(dimethylamino)phenyl)acrylate (4c)**

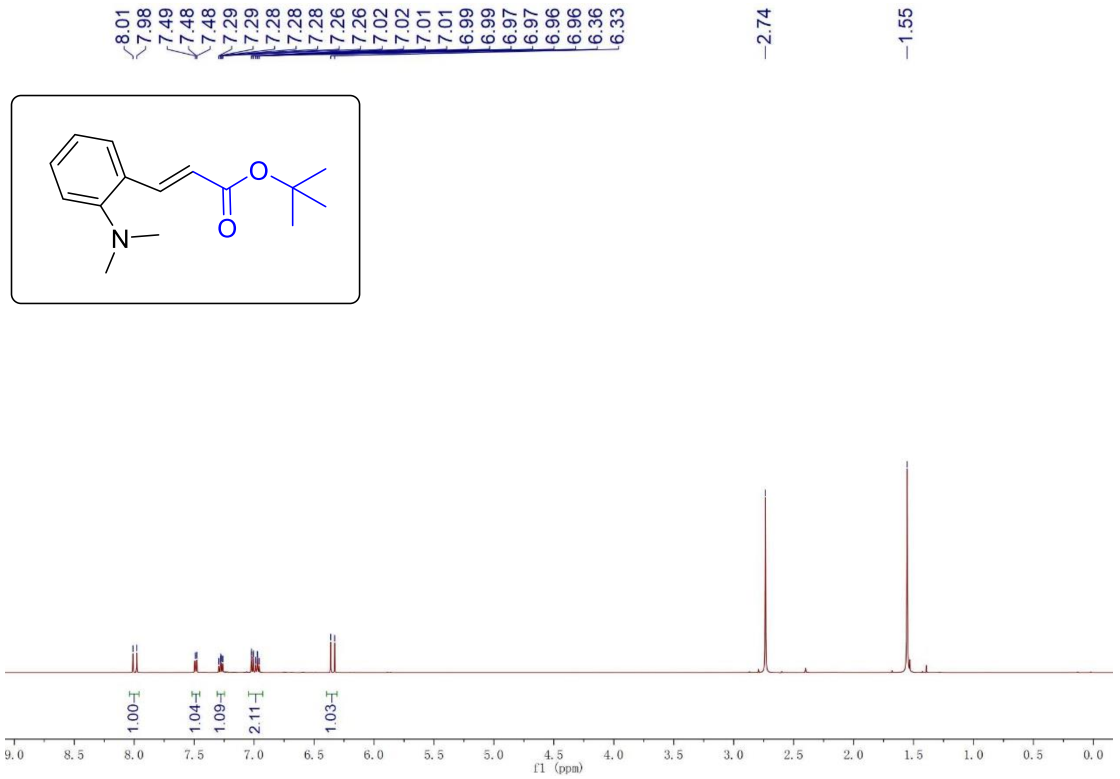


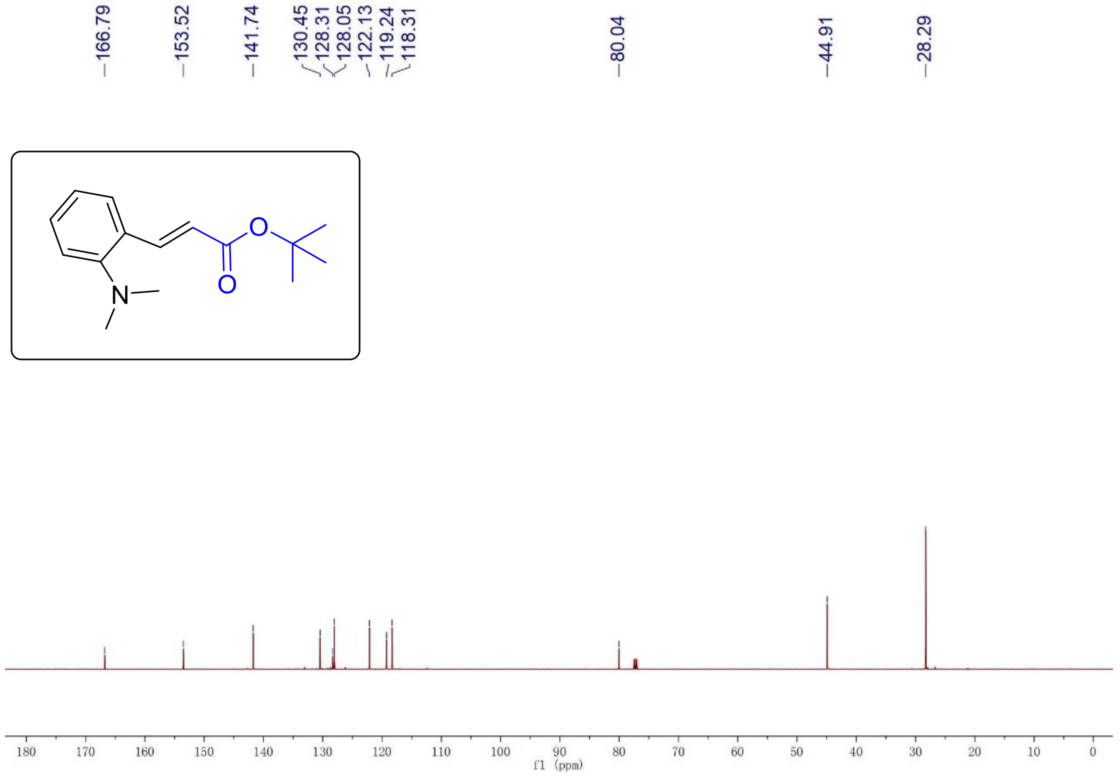


Supplementary Figure 46. $^{1}$H NMR and $^{13}$C NMR spectrum of **4c**.

**benzyl (E)-3-(2-(dimethylamino)phenyl)acrylate (4d)**

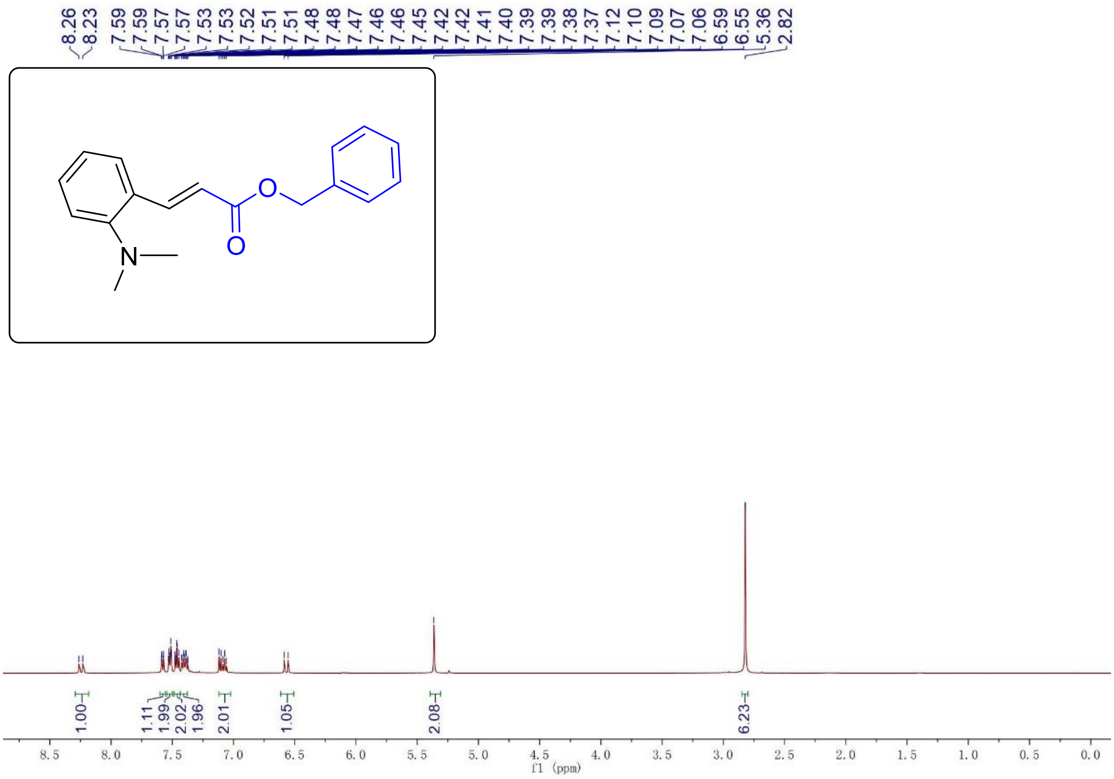


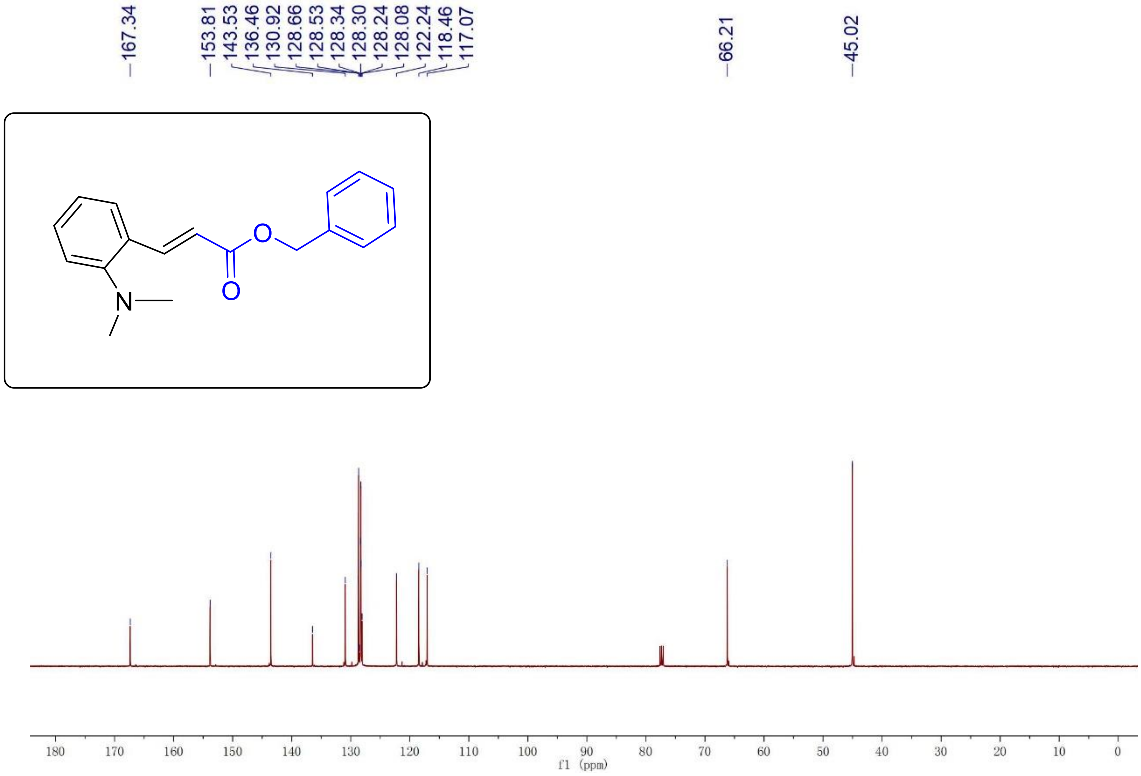


Supplementary Figure 47. $^{1}$H NMR and $^{13}$C NMR spectrum of **4d**.

**(E)-3-(2-(dimethylamino)phenyl)acrylonitrile (4e) 2:1**

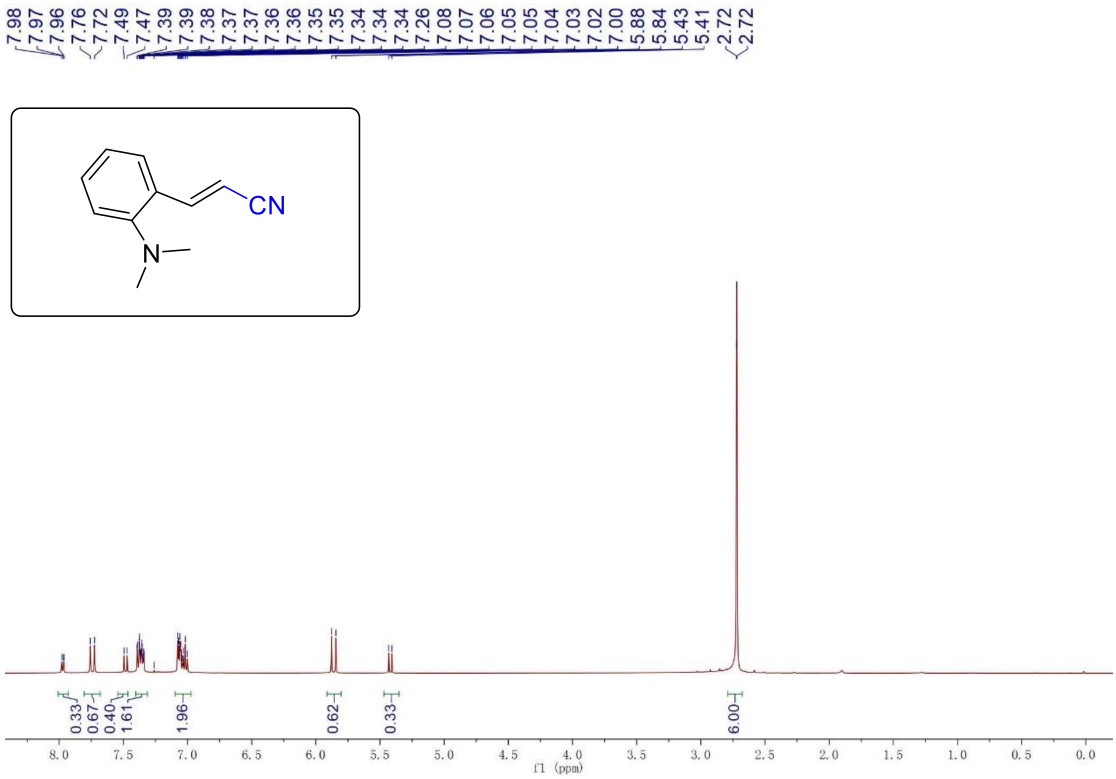

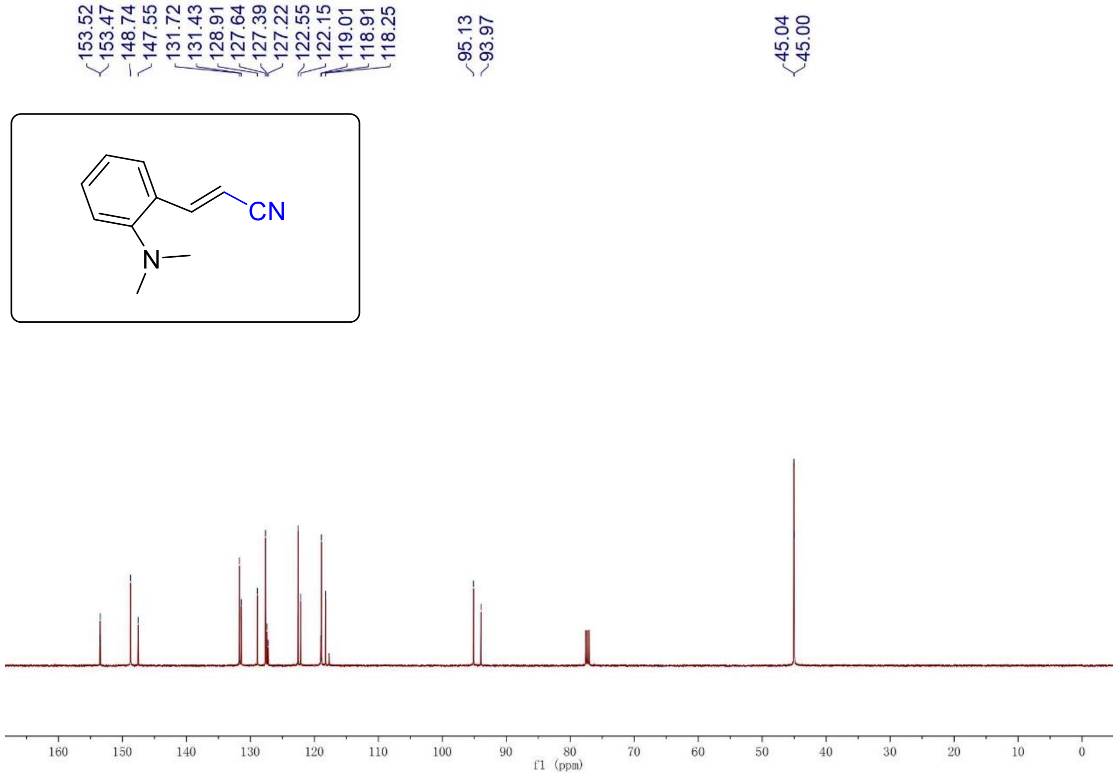

Supplementary Figure 48. $^{1}H$ NMR and $^{13}C$ NMR spectrum of **4e**.

**2,6-di-tert-butyl-4-(2-(dimethylamino)benzylidene)cyclohexa-2,5-dien-1-ol (6a)**

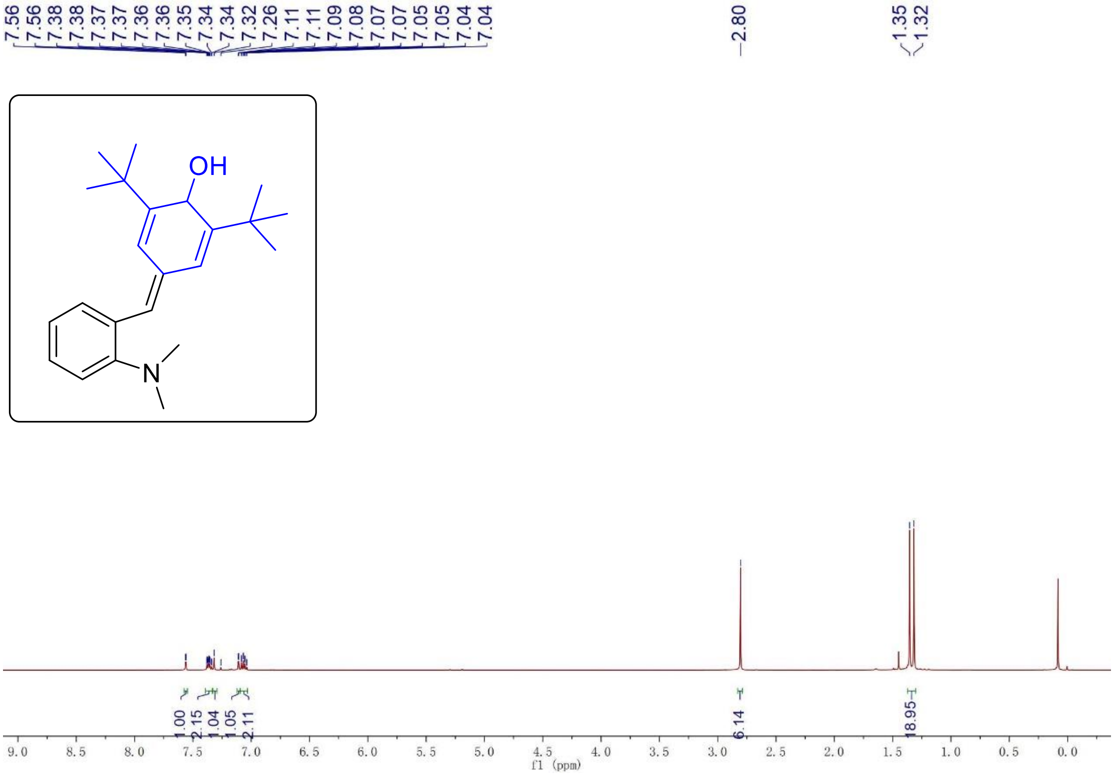


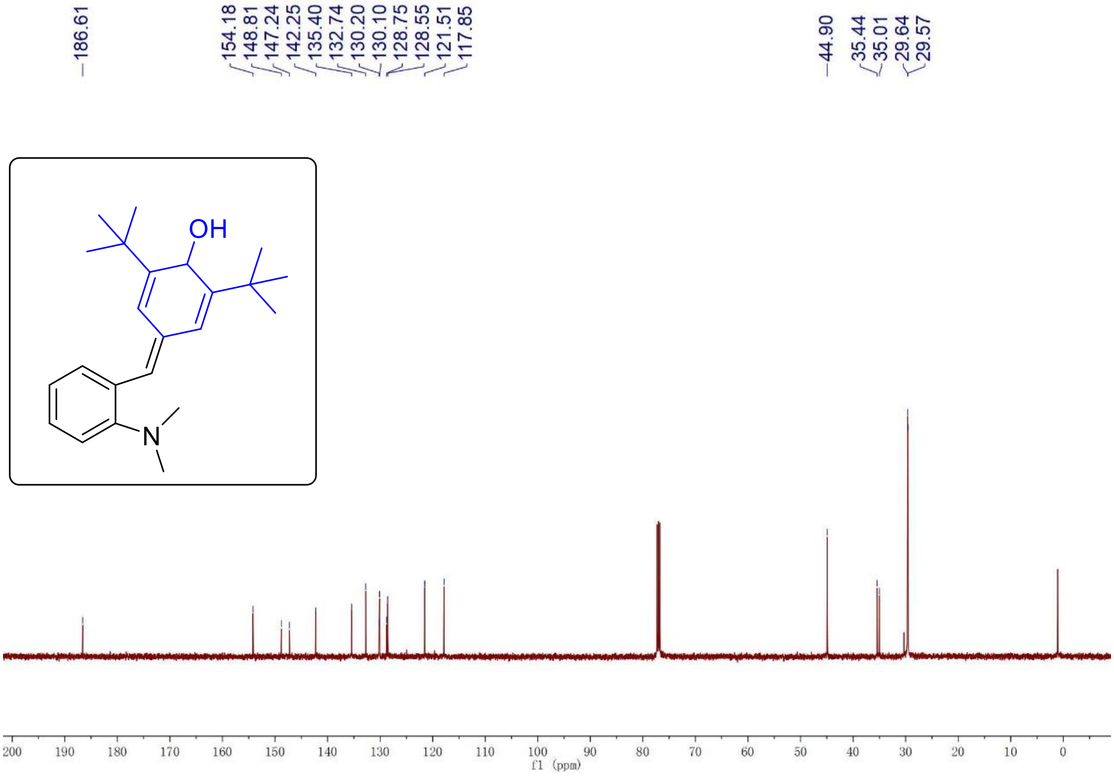


Supplementary Figure 49. $^1$H NMR and $^{13}$C NMR spectrum of **6a**.

**2,6-di-tert-butyl-4-(2-(dimethylamino)-5-methylbenzylidene)cyclohexa-2,5-dien-1-ol (6b)**

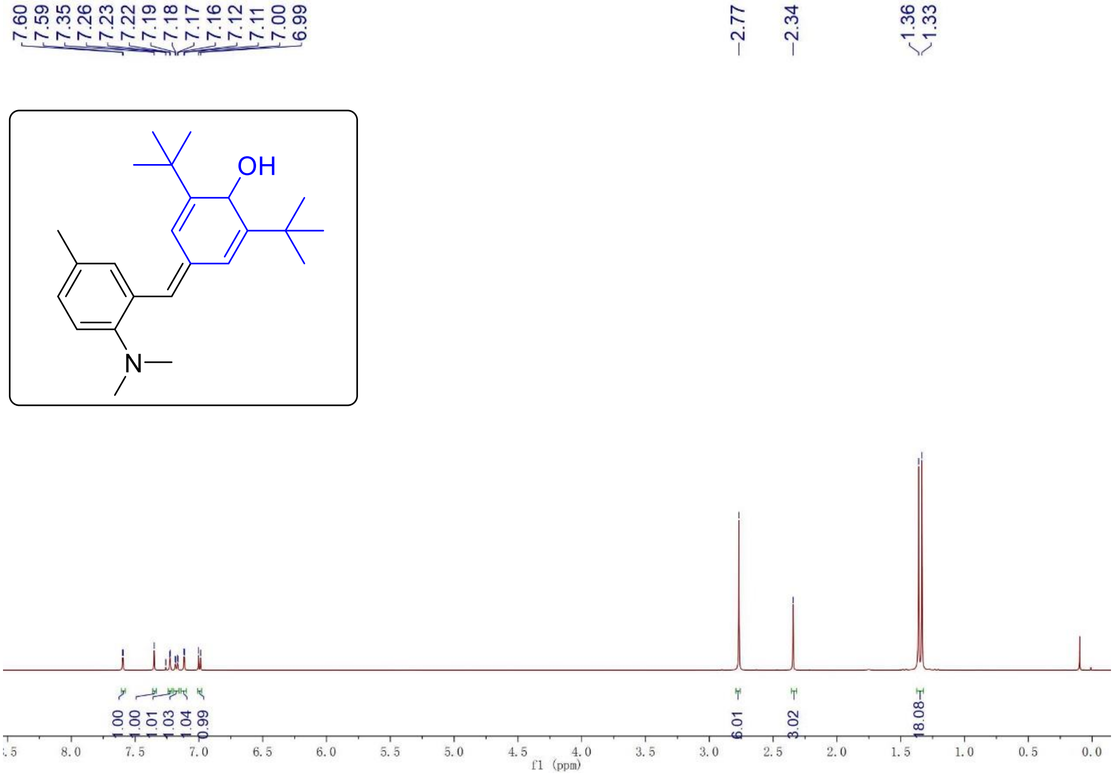


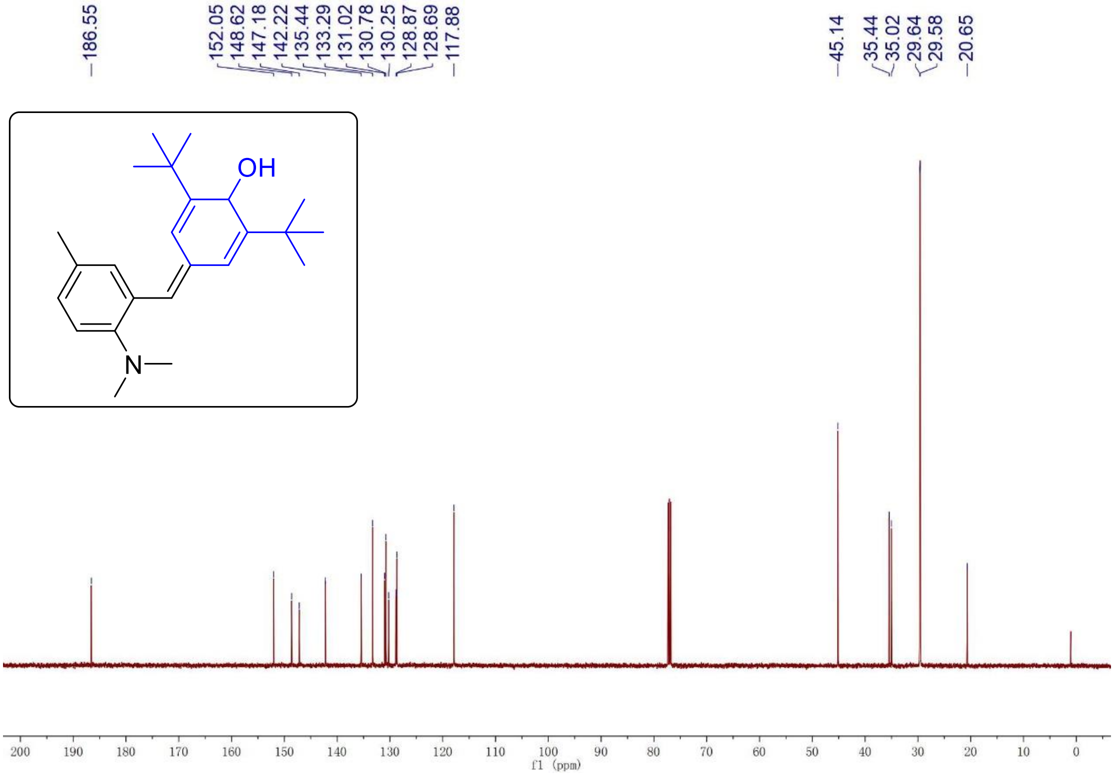


Supplementary Figure 50. $^1$H NMR and $^{13}$C NMR spectrum of **6b**.

**4-(5-bromo-2-(dimethylamino)benzylidene)-2,6-di-tert-butylcyclohexa-2,5-dien-1-one (6c)**

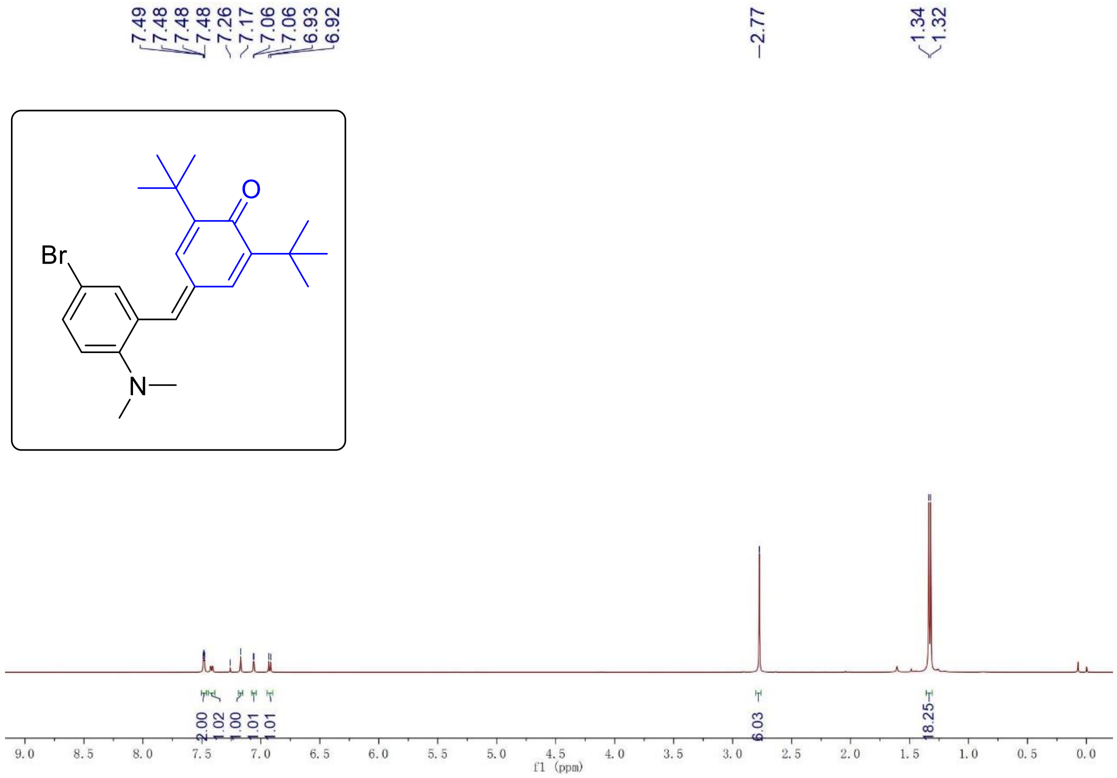


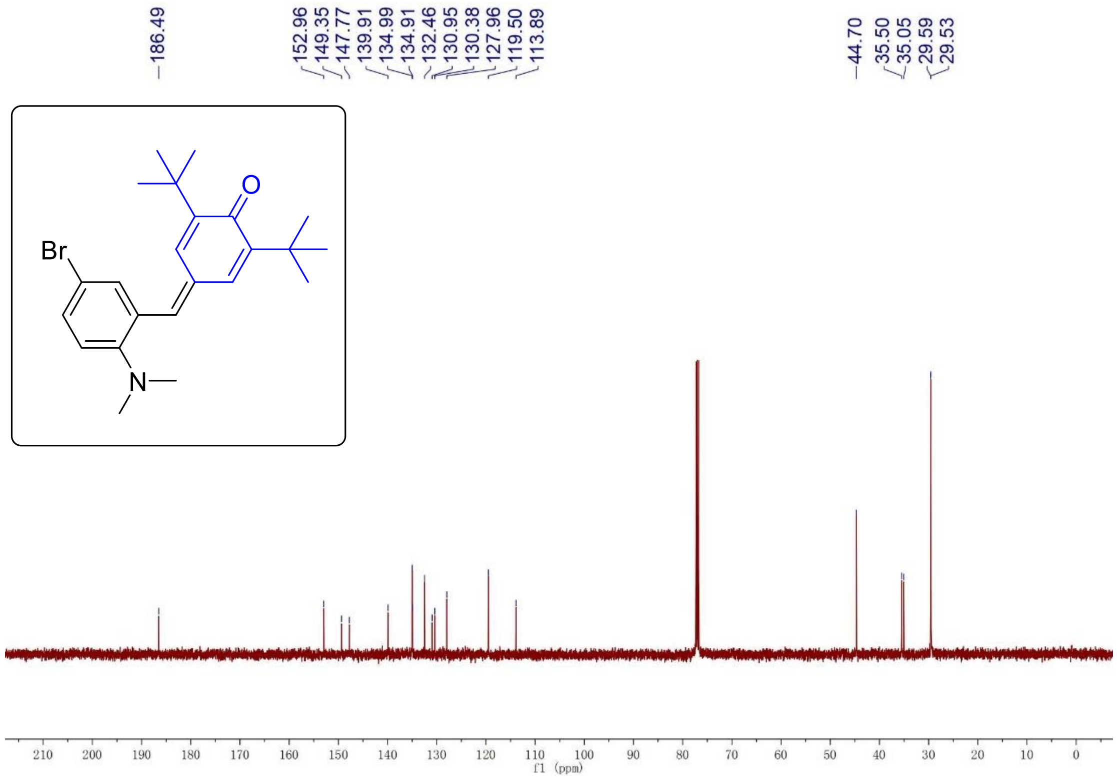


Supplementary Figure 51. $^{1}$H NMR and $^{13}$C NMR spectrum of **6c**.

**2,6-di-tert-butyl-4-(2-chloro-6-(dimethylamino)benzylidene)cyclohexa-2,5-dien-1-one (6d)**

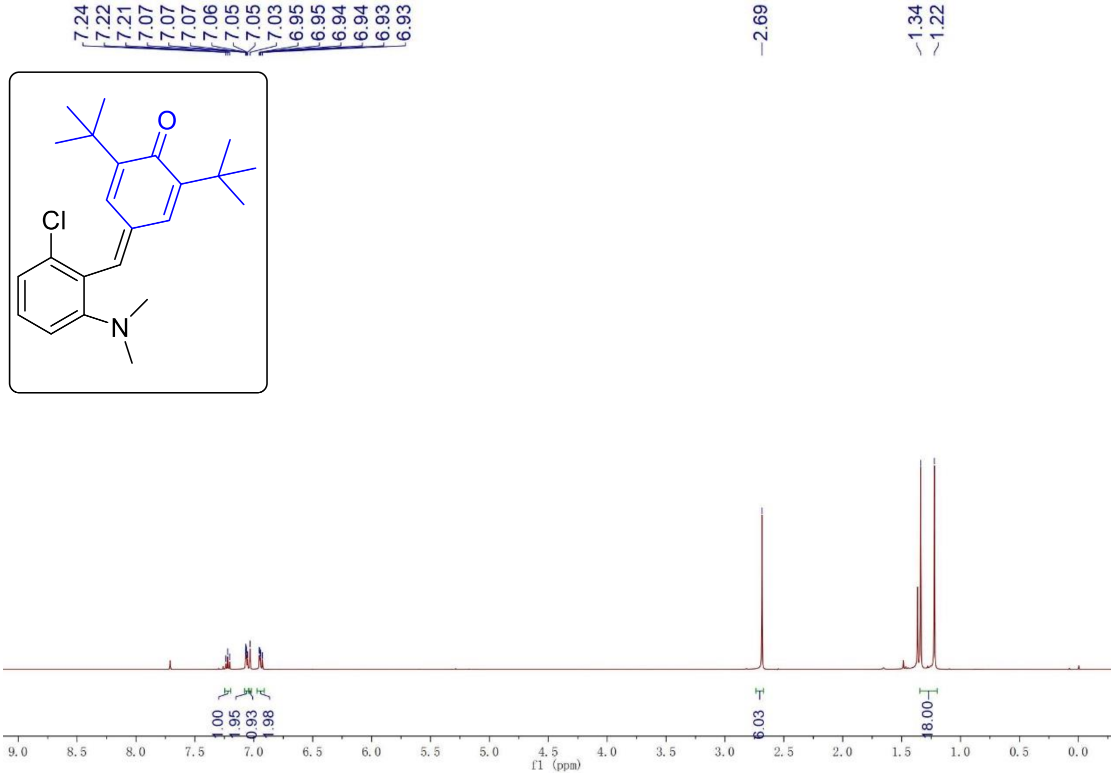


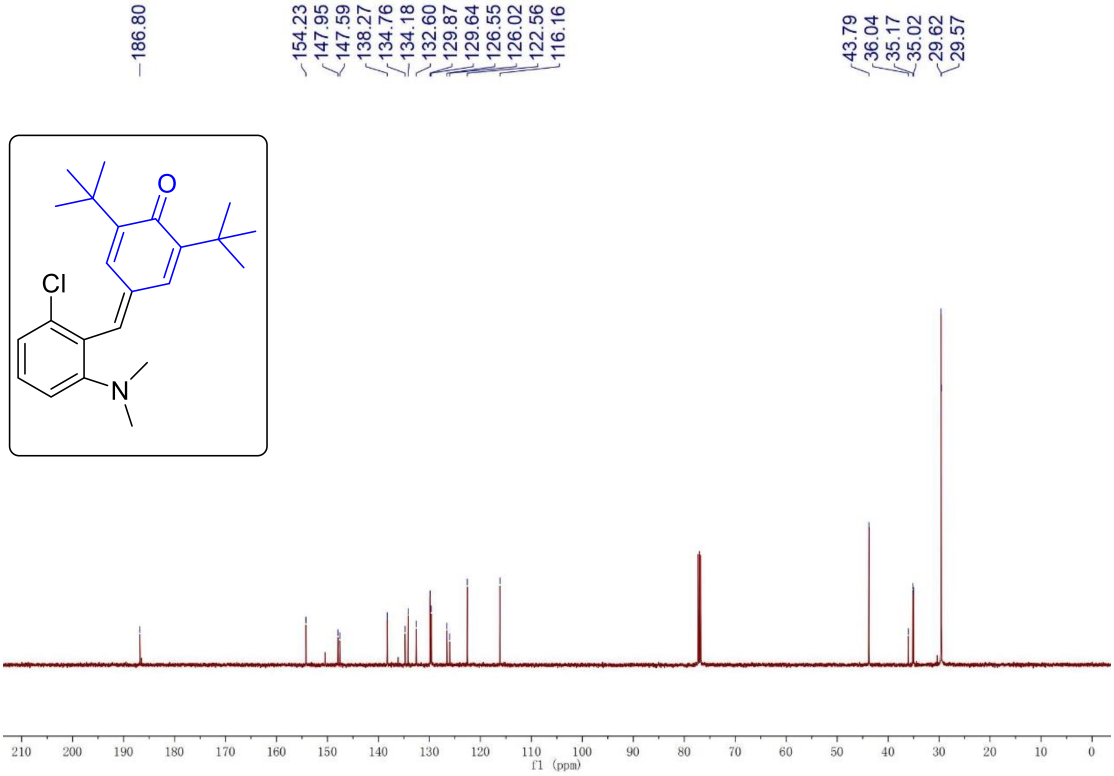


Supplementary Figure 52. $^{1}$H NMR and $^{13}$C NMR spectrum of **6d**.

**2,6-di-tert-butyl-4-(2-(dimethylamino)-3-fluorobenzylidene)cyclohexa-2,5-dien-1-one (6e)**

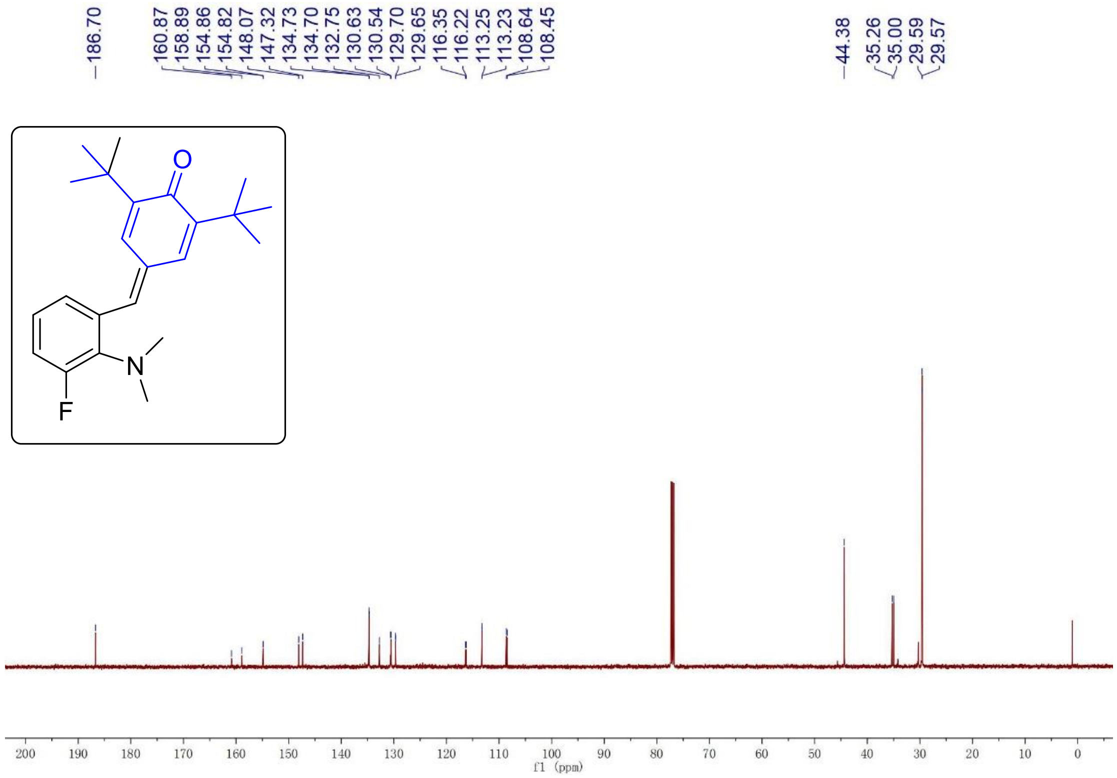


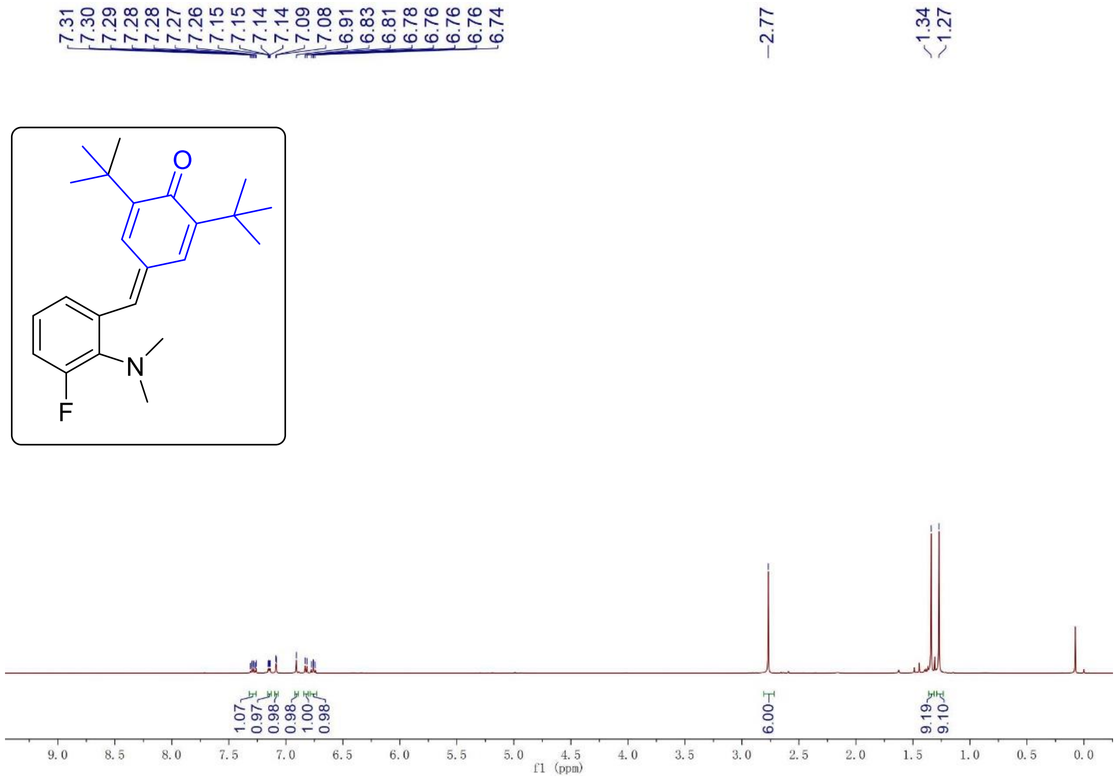

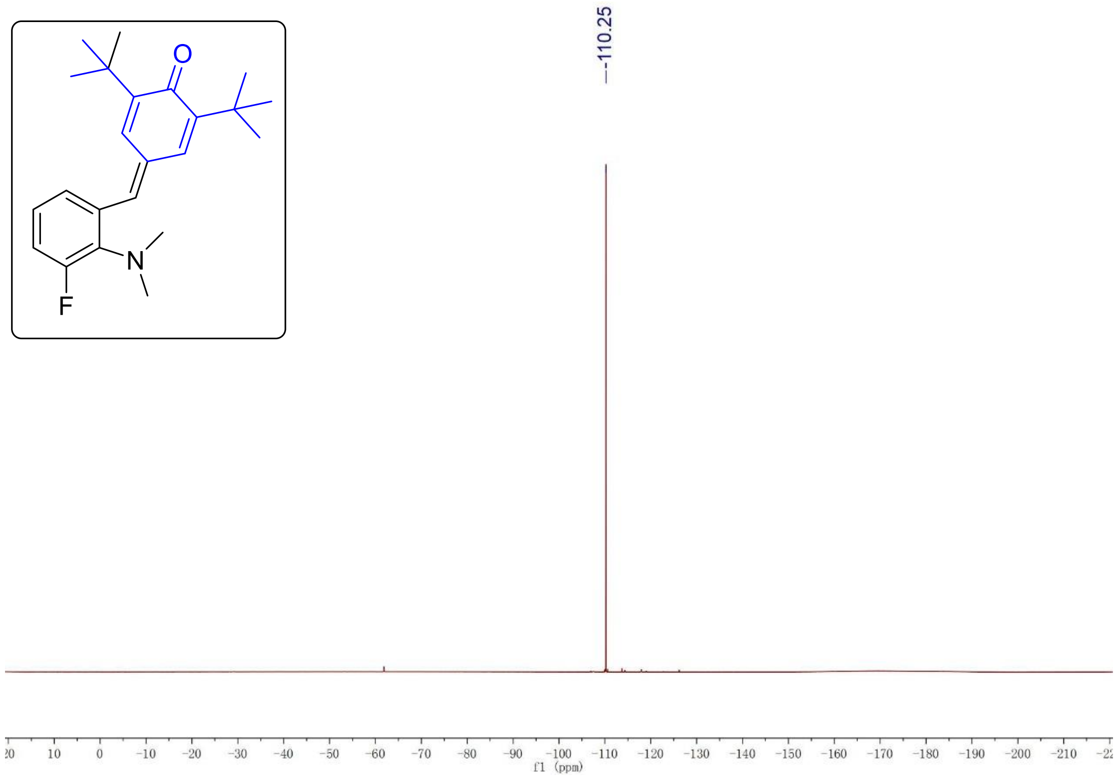


Supplementary Figure 53. $^{1}$H NMR $^{13}$C NMR and $^{19}$F NMRspectrum of **6e**.

**(E)-3-(2-(azetidin-1-yl)phenyl)-1-phenylprop-2-en-1-one (8a)**

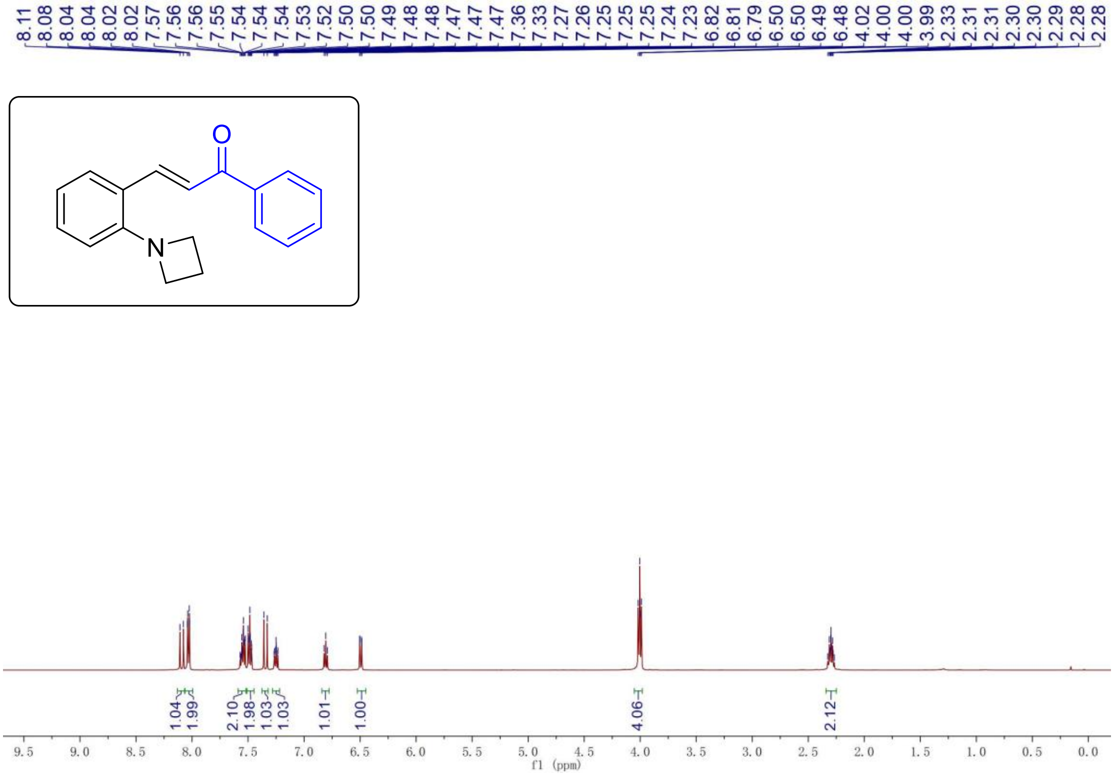


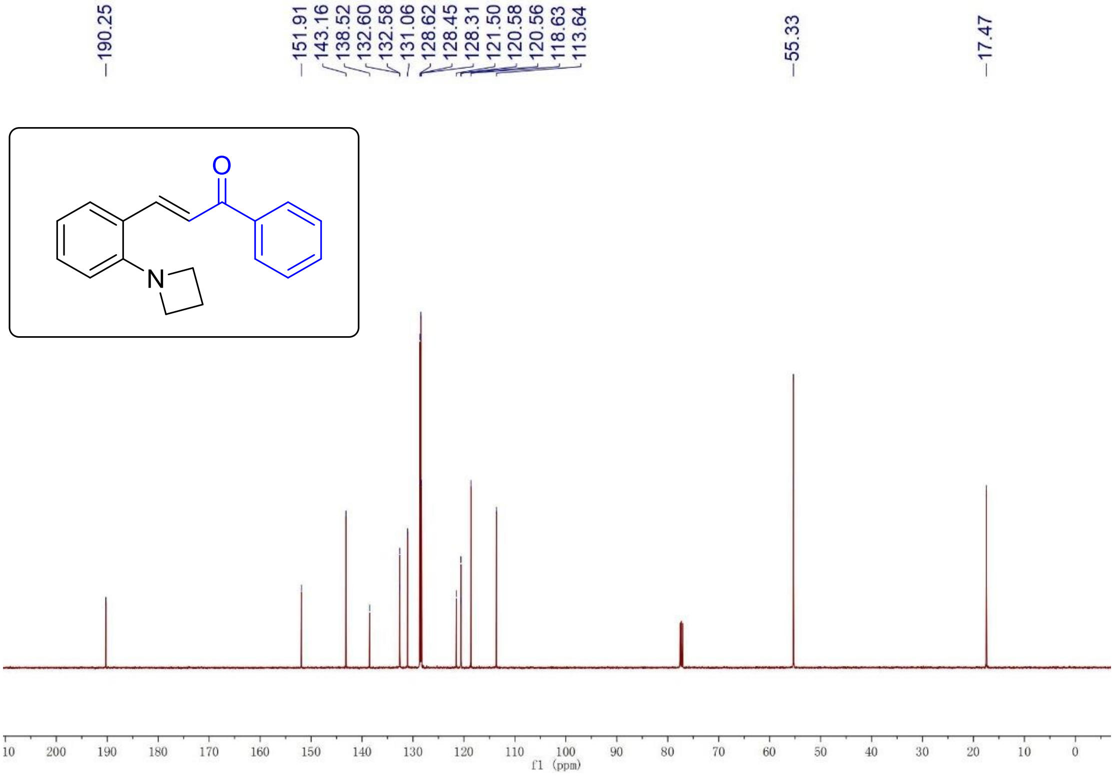


Supplementary Figure 54. $^{1}$H NMR and $^{13}$C NMR spectrum of **8a**.

## (E)-1-phenyl-3-(2-(pyrrolidin-1-yl)phenyl)prop-2-en-1-one (8b)

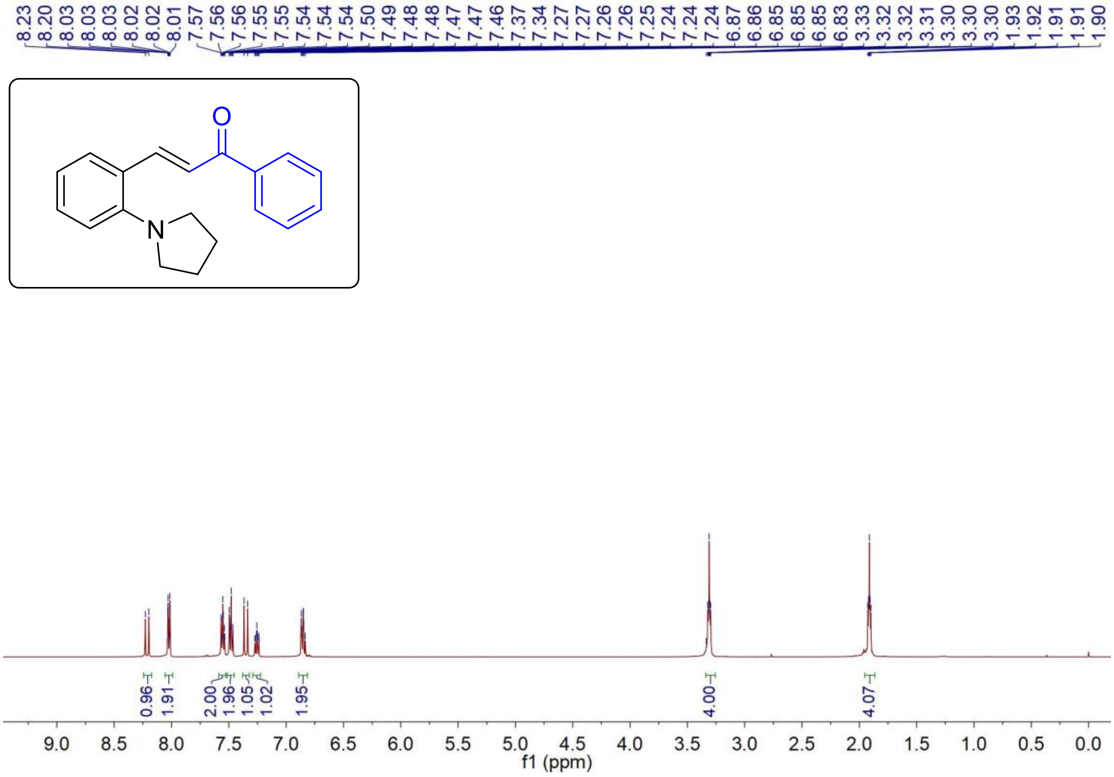


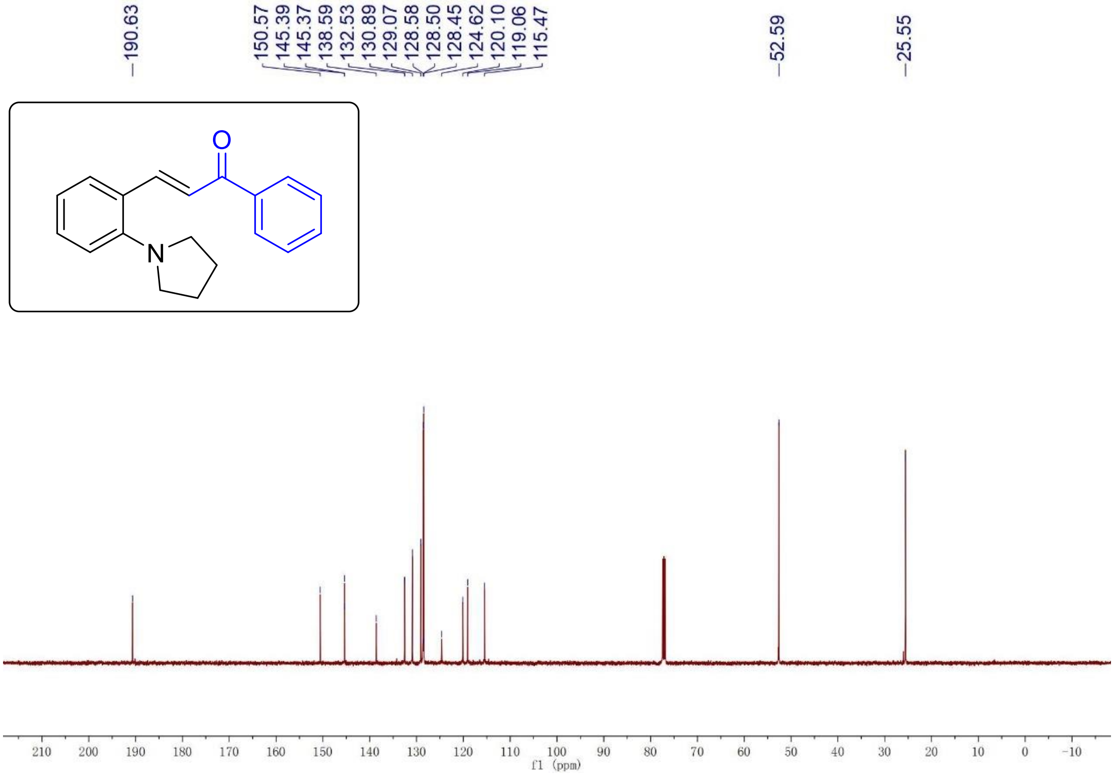


Supplementary Figure 55. $^{1}$H NMR and $^{13}$C NMR spectrum of **8b**.

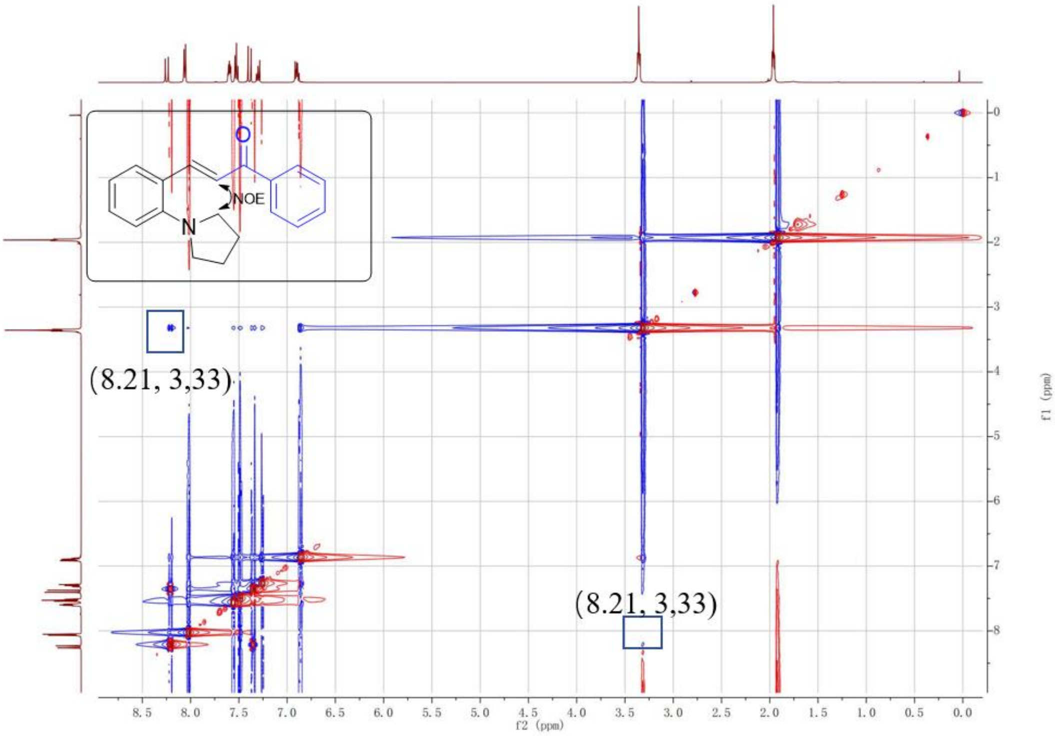


Supplementary Figure 56. $^{1}$H - $^{1}$H NOESY NMR spectrum of **8b**.

**(E)-1-([1,1'-biphenyl]-4-yl)-3-(2-(pyrrolidin-1-yl)phenyl)prop-2-en-1-one (8d)**

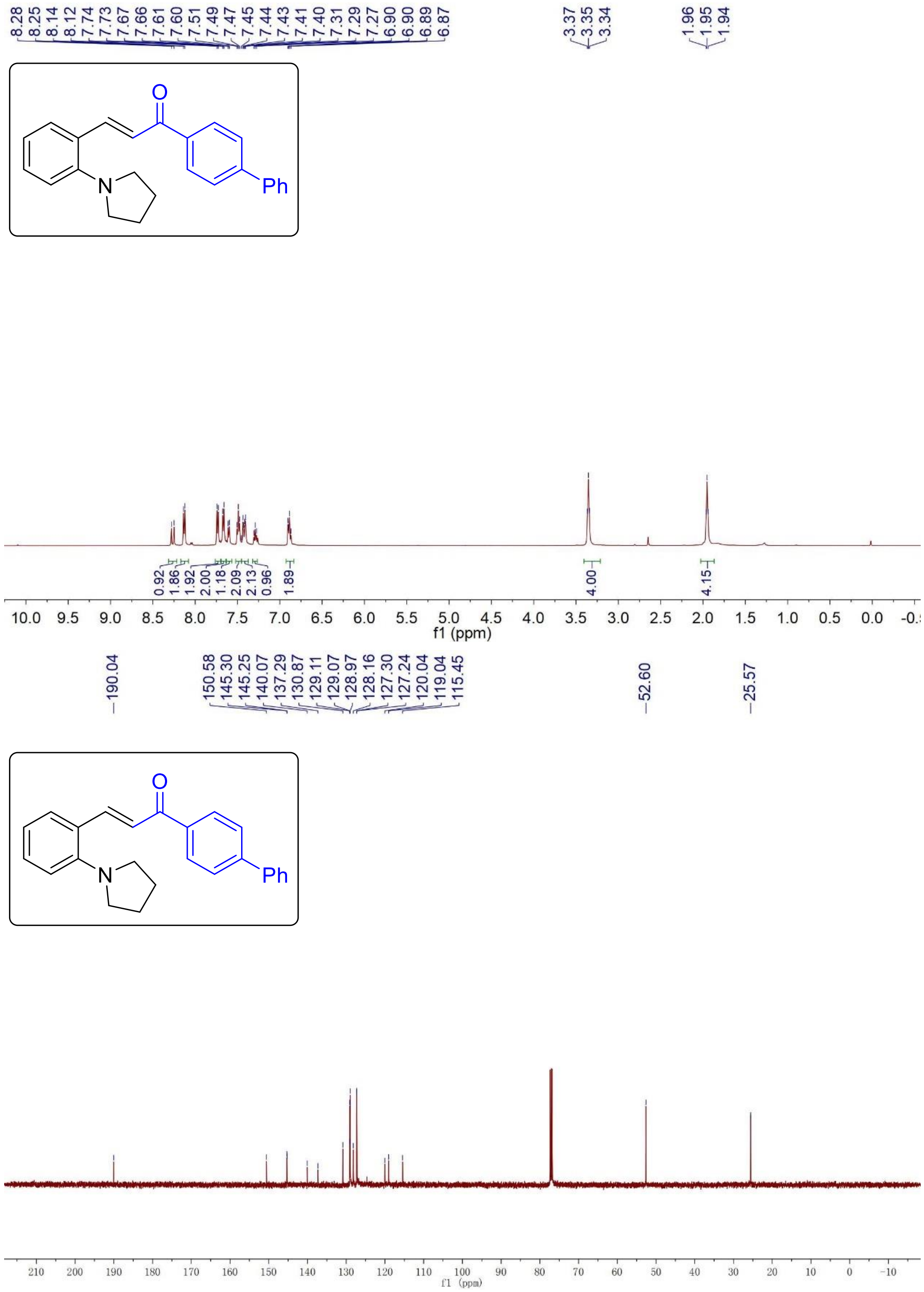

Supplementary Figure 57. $^1$H NMR and $^{13}$C NMR spectrum of **8d**.

**(E)-1-(4-isobutylphenyl)-3-(2-(pyrrolidin-1-yl)phenyl)prop-2-en-1-one (8e)**

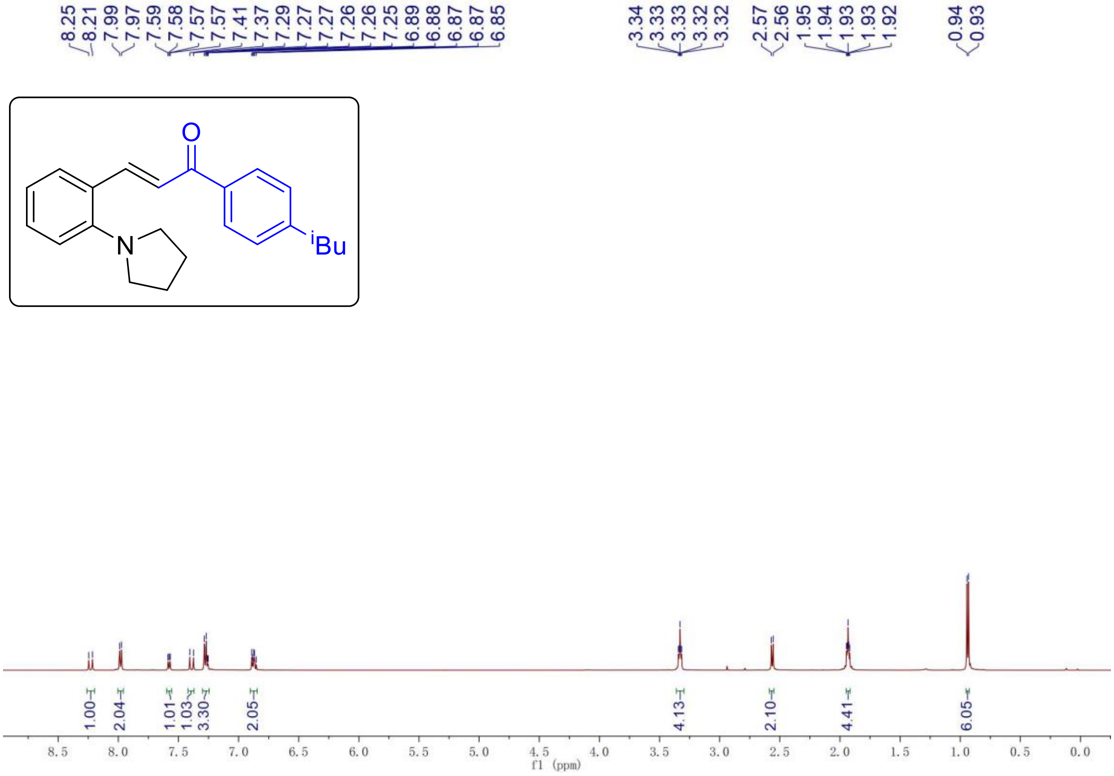


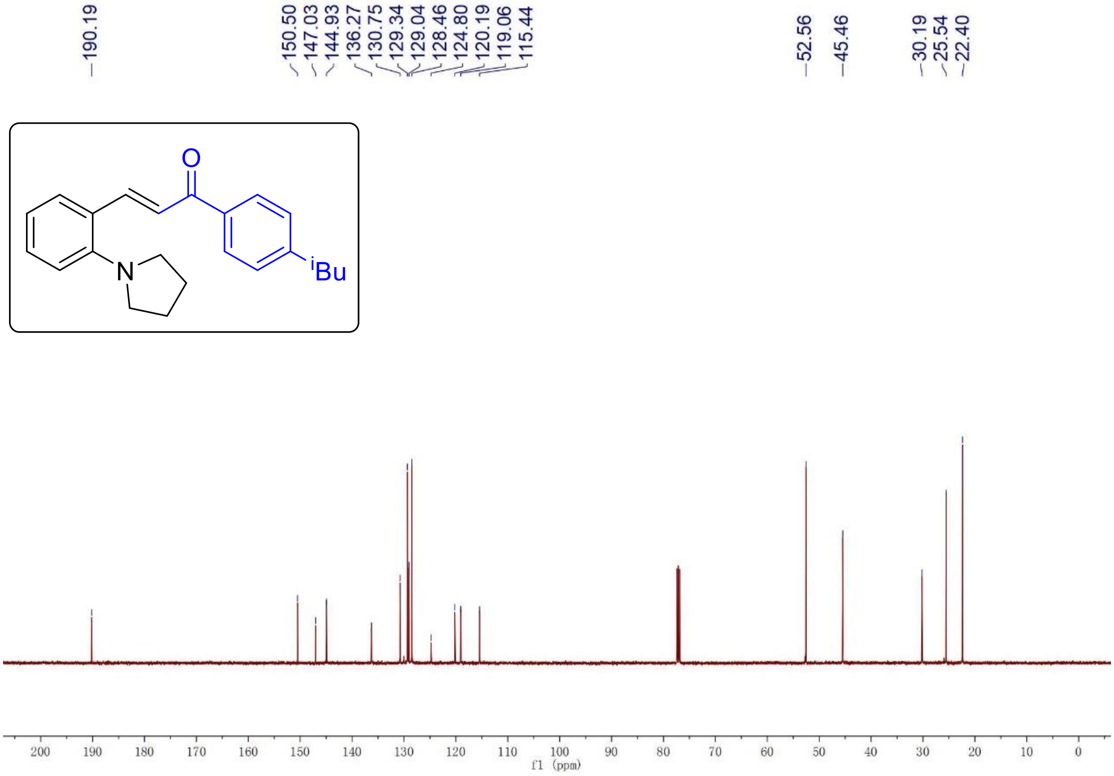


Supplementary Figure 58. $^1$H NMR and $^{13}$C NMR spectrum of **8e**.

**(E)-1-(4-methoxyphenyl)-3-(2-(pyrrolidin-1-yl)phenyl)prop-2-en-1-one (8f)**

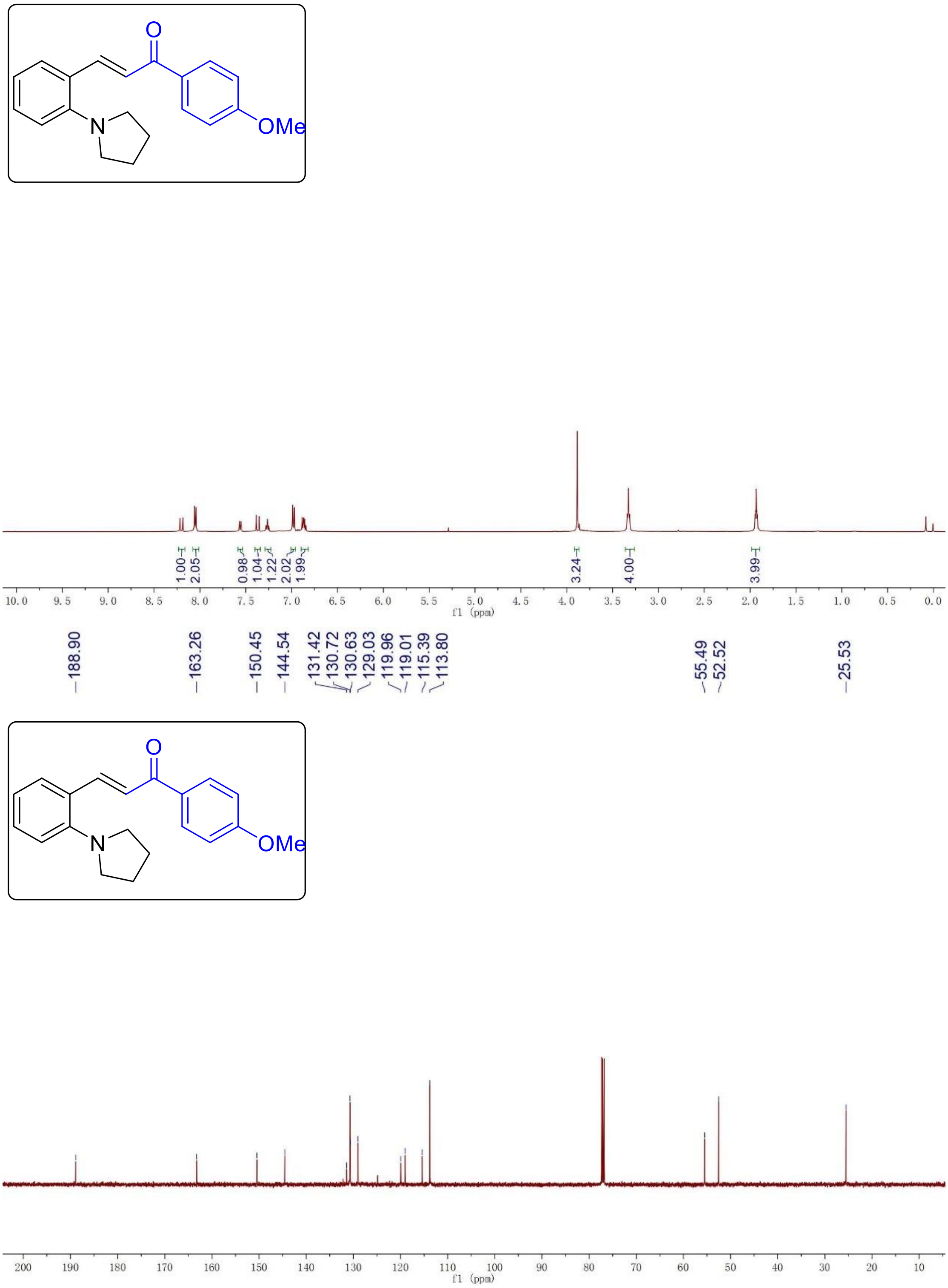


Supplementary Figure 59. $^{1}$H NMR and $^{13}$C NMR spectrum of **8f**.

**(E)-1-(4-bromophenyl)-3-(2-(pyrrolidin-1-yl)phenyl)prop-2-en-1-one (8g)**

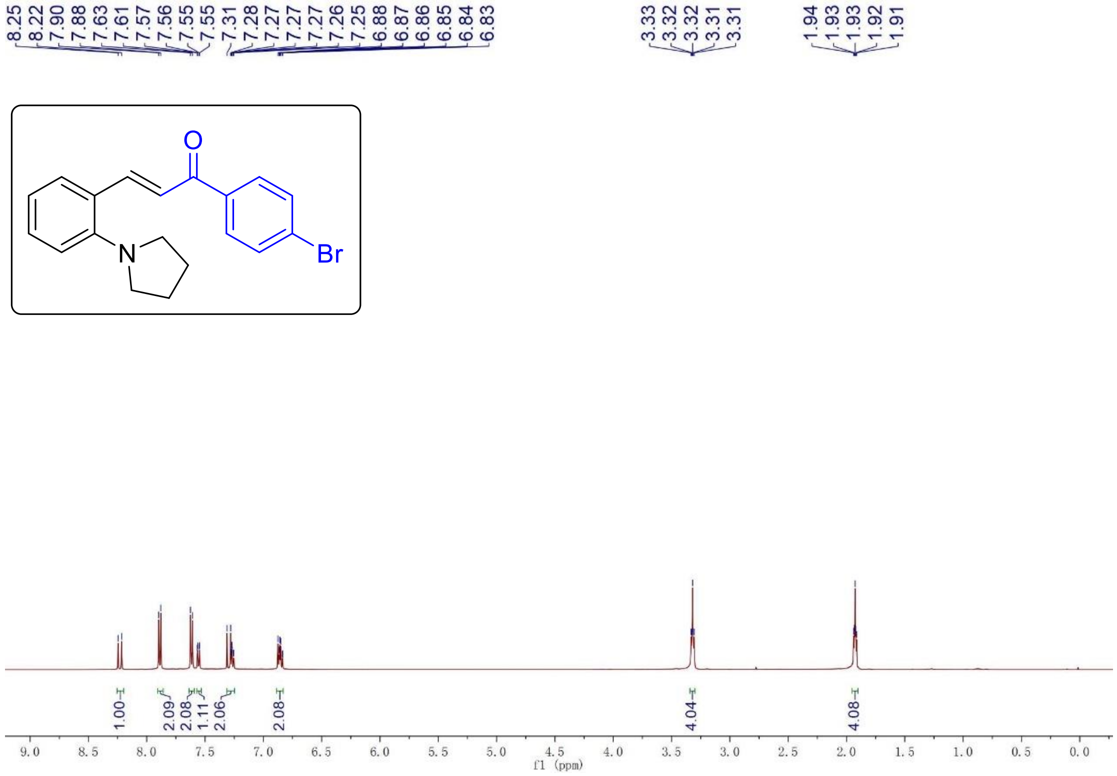


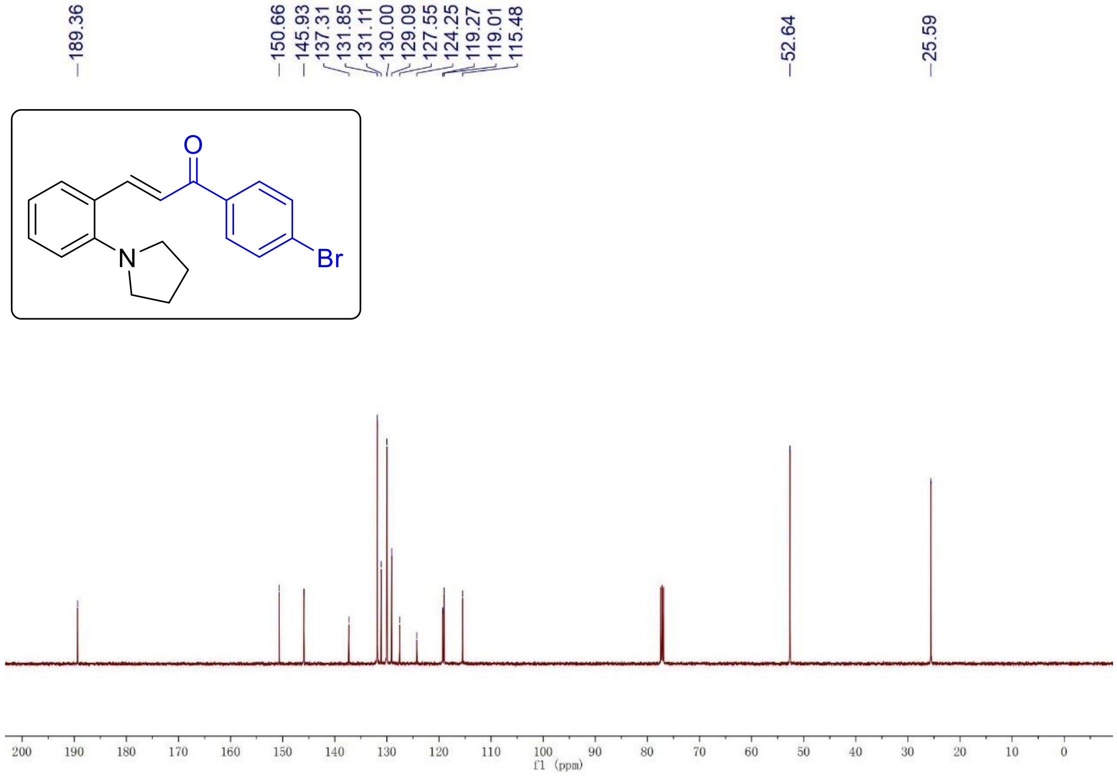


Supplementary Figure 60. $^{1}$H NMR and $^{13}$C NMR spectrum of **8g**.

**(E)-1-(4-iodophenyl)-3-(2-(pyrrolidin-1-yl)phenyl)prop-2-en-1-one (8h)**

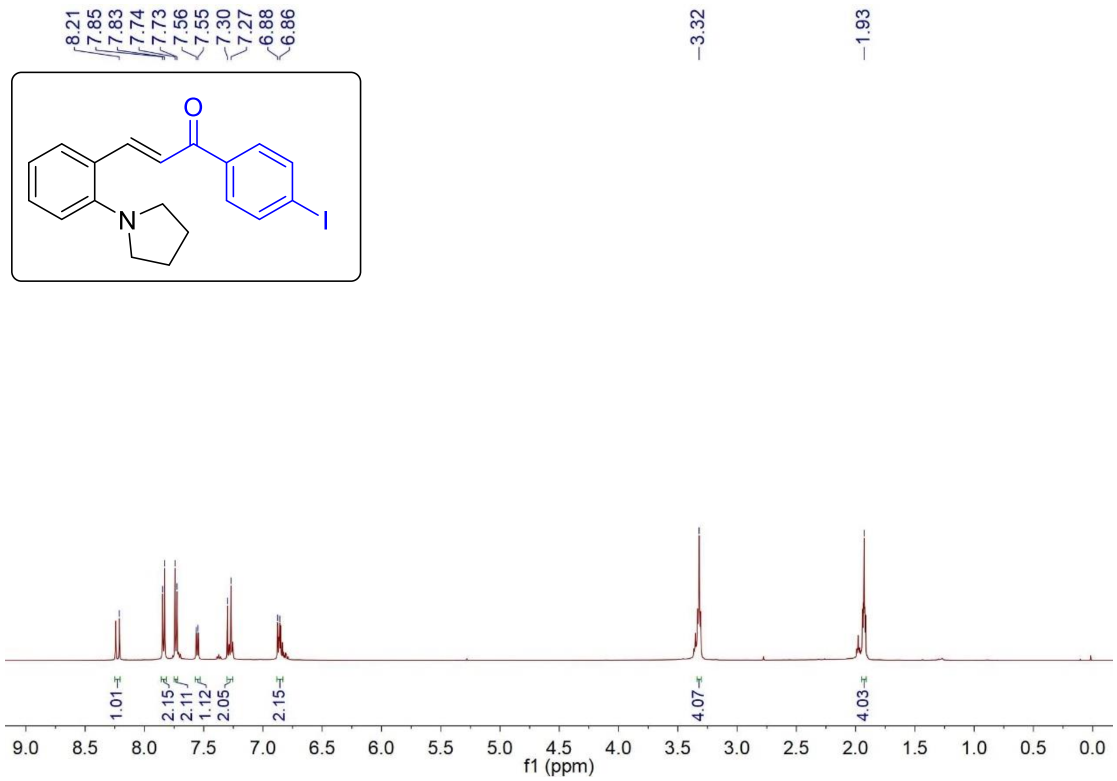


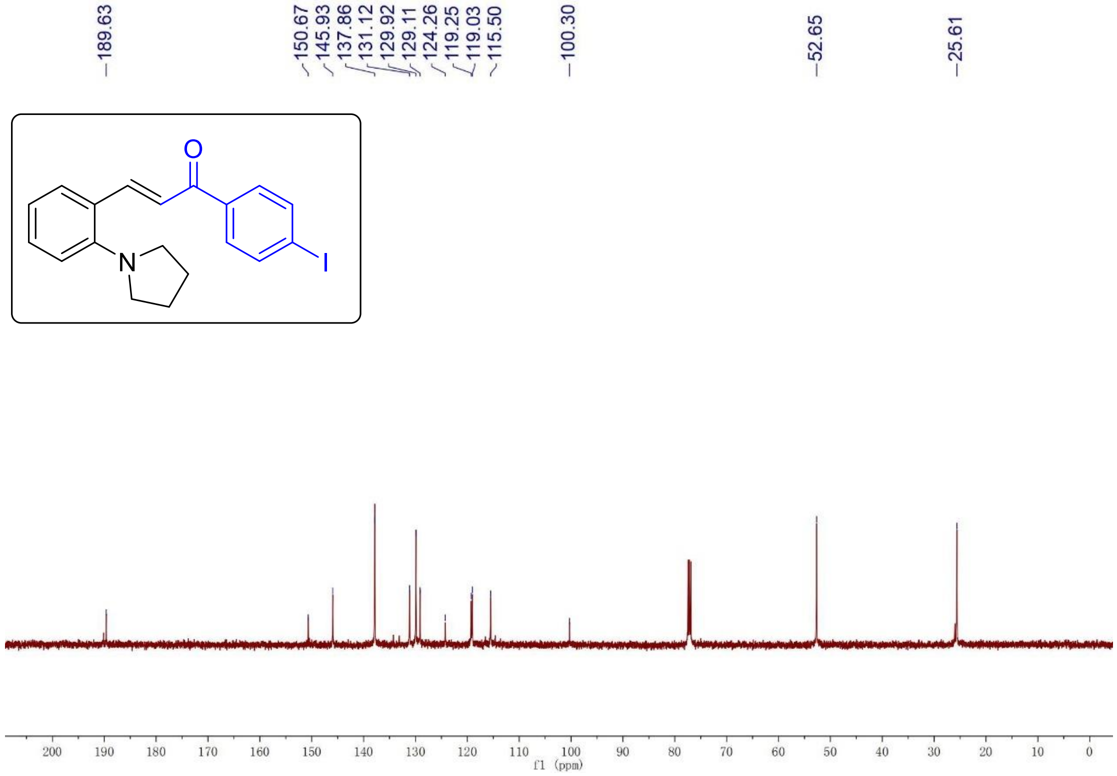


Supplementary Figure 61. $^{1}$H NMR and $^{13}$C NMR spectrum of **8h**.

**(E)-3-(2-(pyrrolidin-1-yl)phenyl)-1-(o-tolyl)prop-2-en-1-one (8i)**

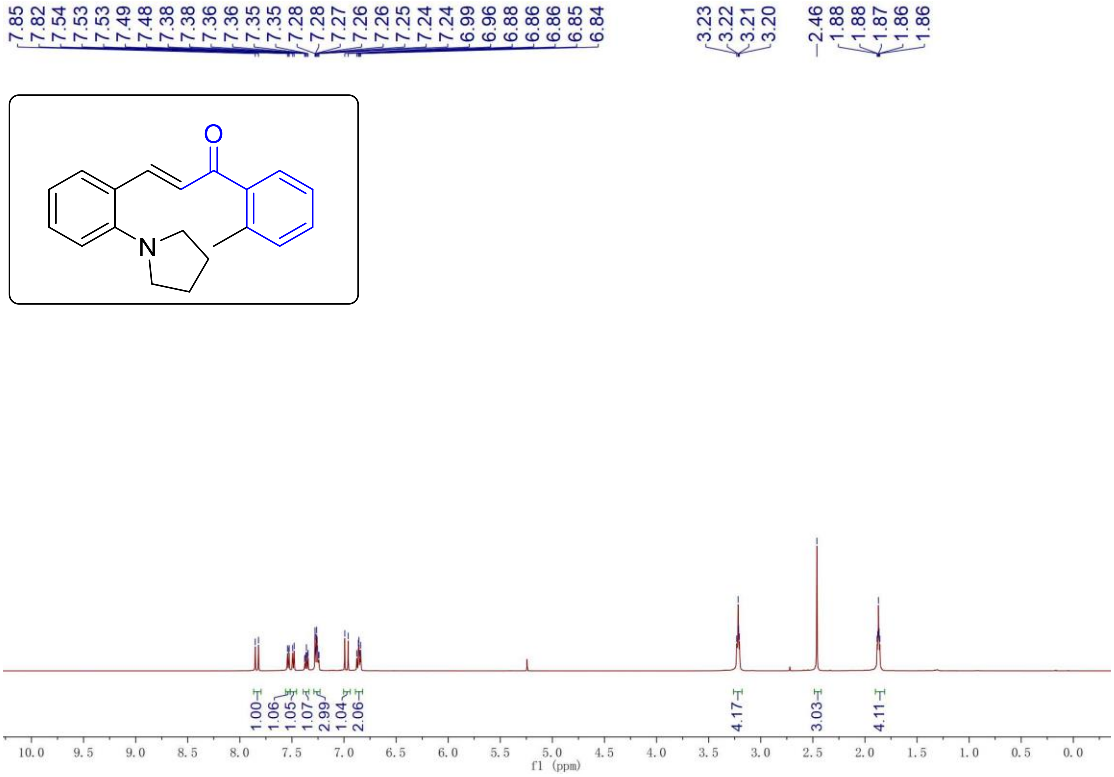

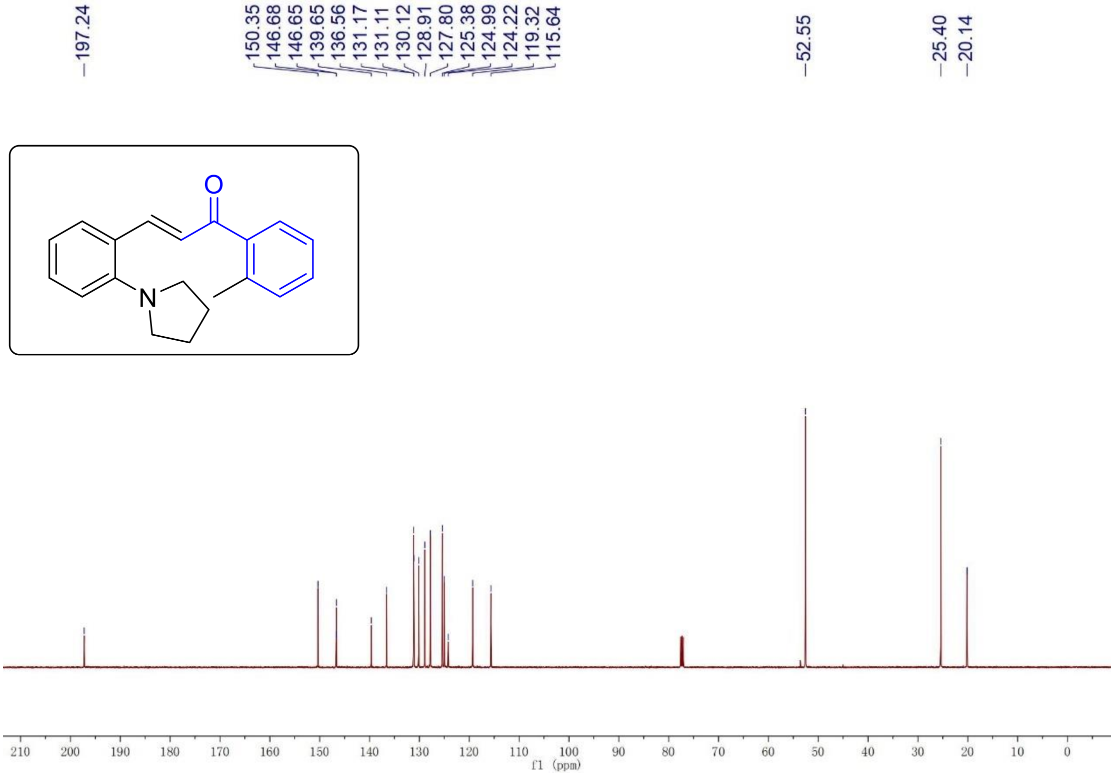

Supplementary Figure 62. $^{1}$H NMR and $^{13}$C NMR spectrum of **8i**.

**(E)-3-(2-(pyrrolidin-1-yl)phenyl)-1-(2-(trifluoromethyl)phenyl)prop-2-en-1-one (8j)**

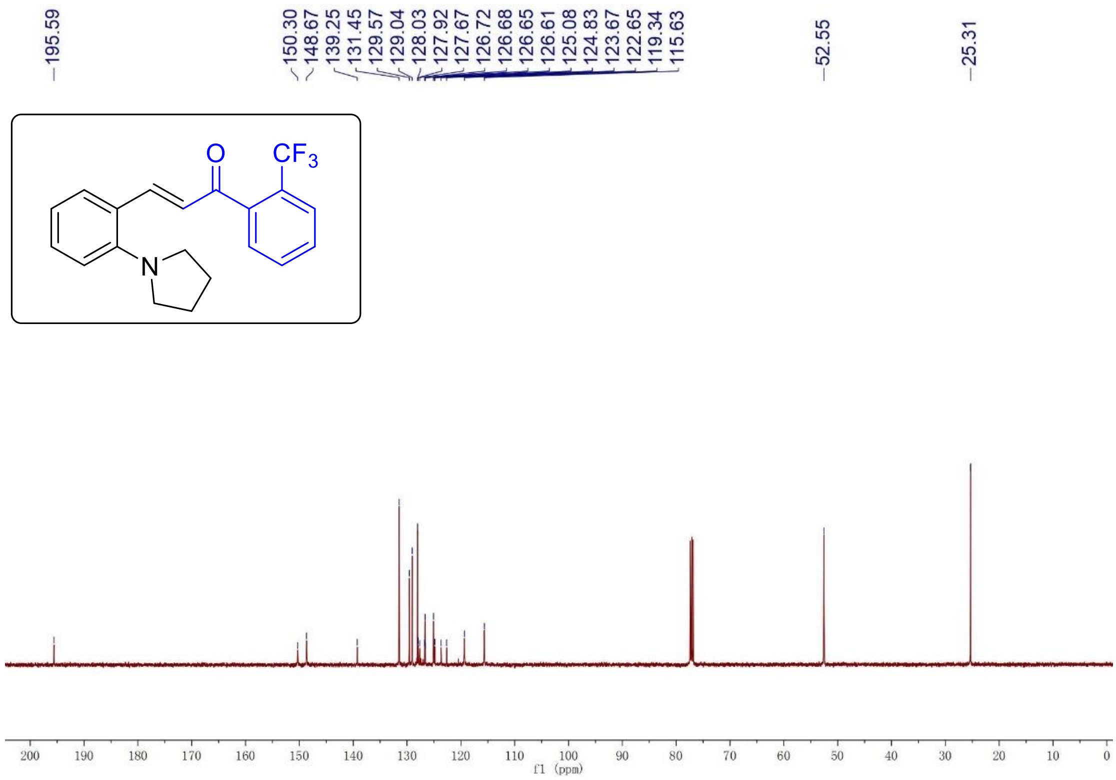

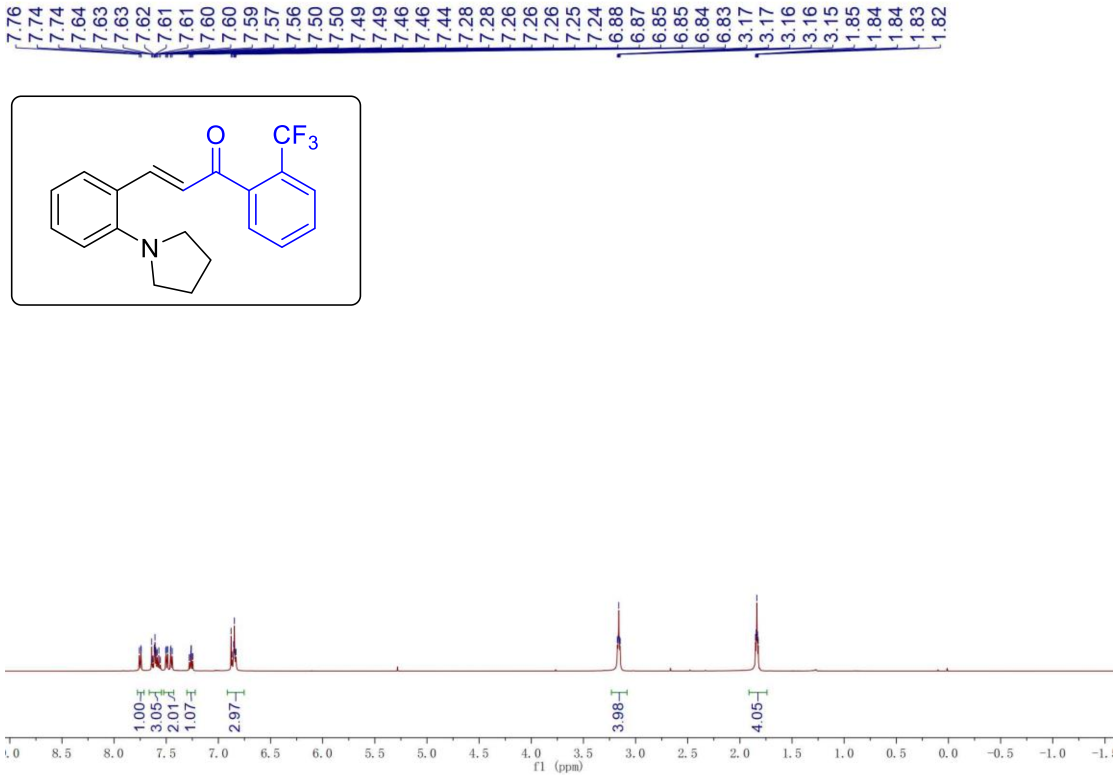

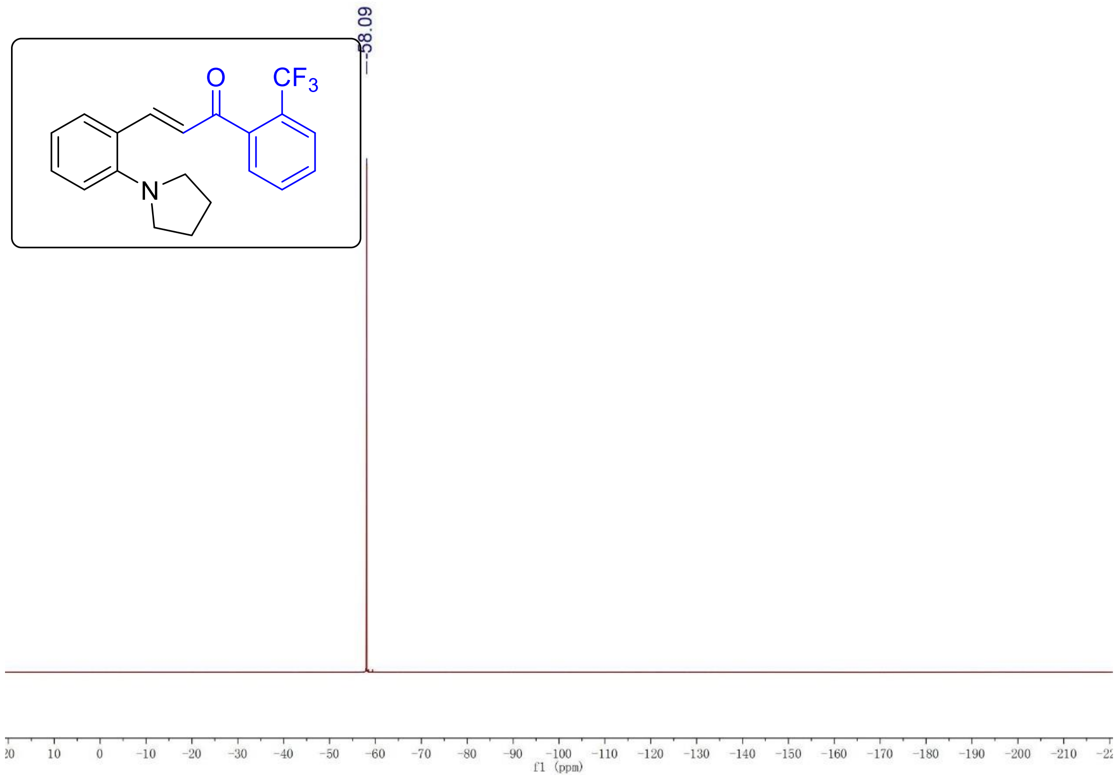


Supplementary Figure 63. $^{1}H$ NMR, $^{13}C$ NMR and $^{19}F$ NMR spectrum of **8j**.

## (E)-1-(3-chlorophenyl)-3-(2-(pyrrolidin-1-yl)phenyl)prop-2-en-1-one (8k)

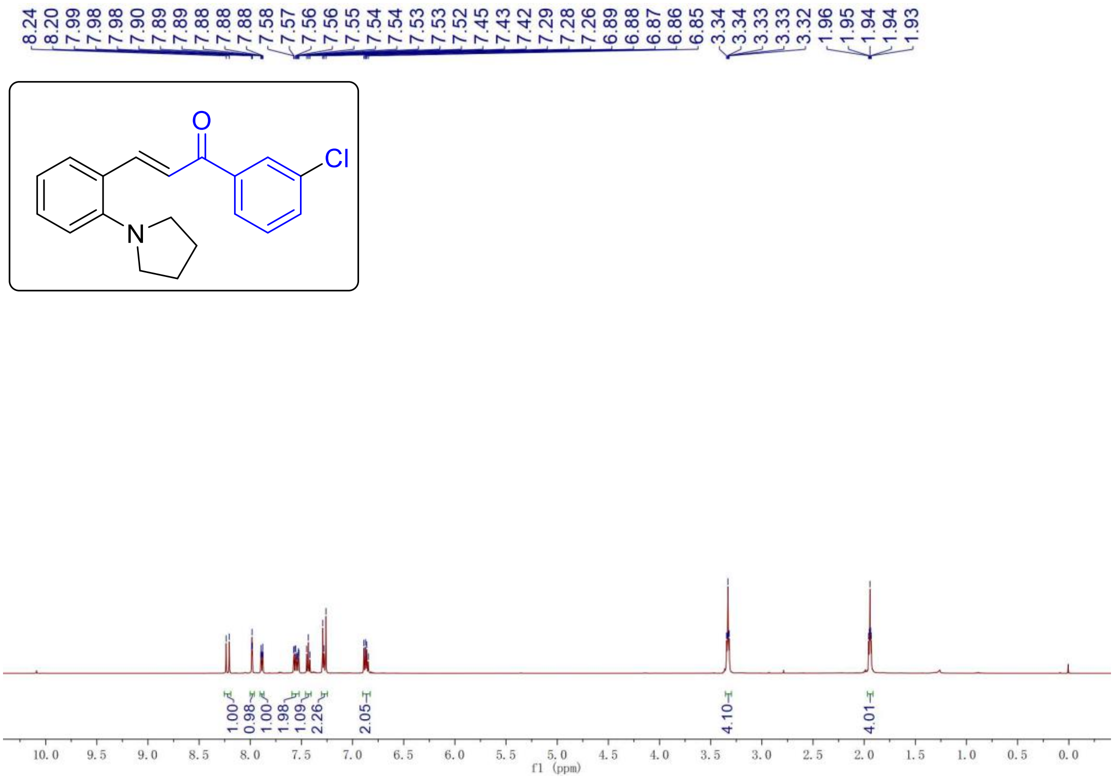


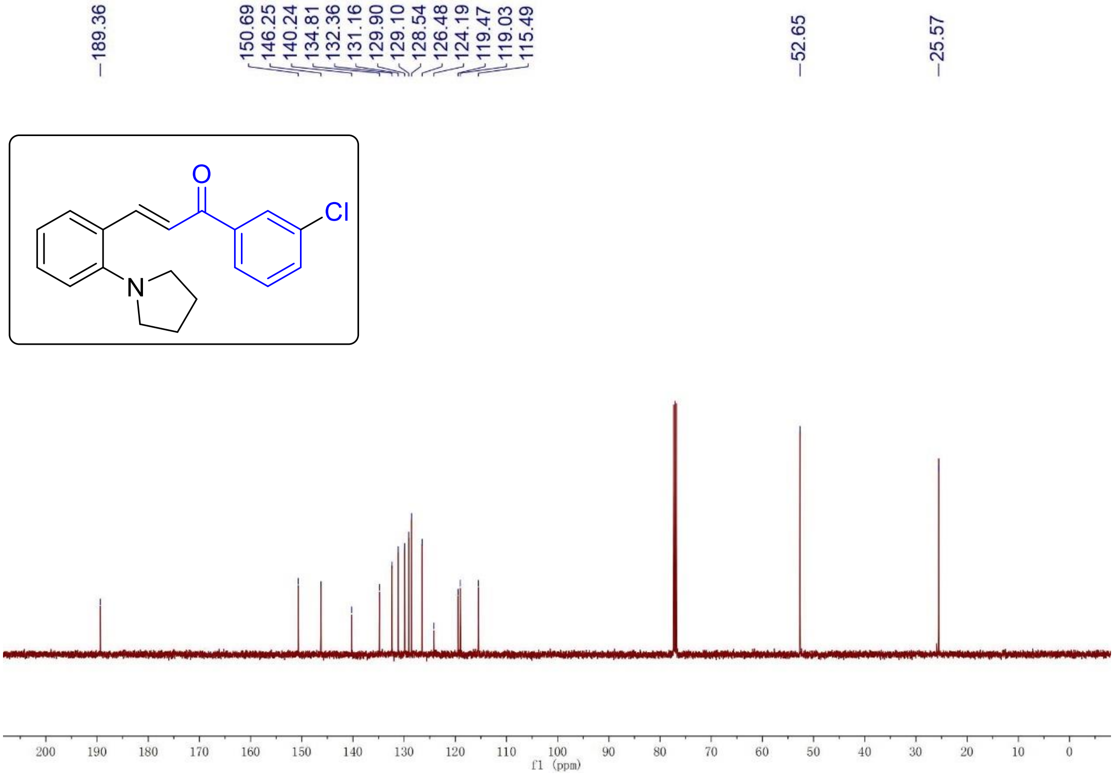


Supplementary Figure 64. $^{1}H$ NMR and $^{13}C$ NMR spectrum of **8k**.

**(E)-3-(2-(pyrrolidin-1-yl)phenyl)-1-(3,4,5-trimethoxyphenyl)prop-2-en-1-one (8m)**

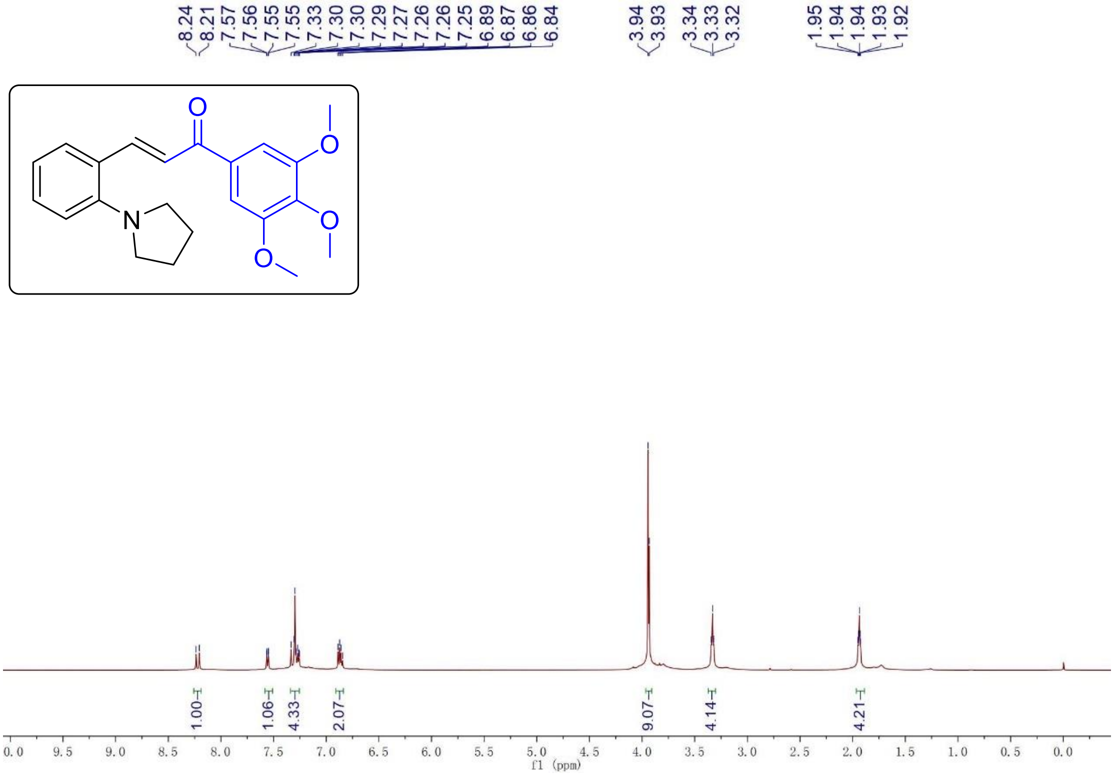


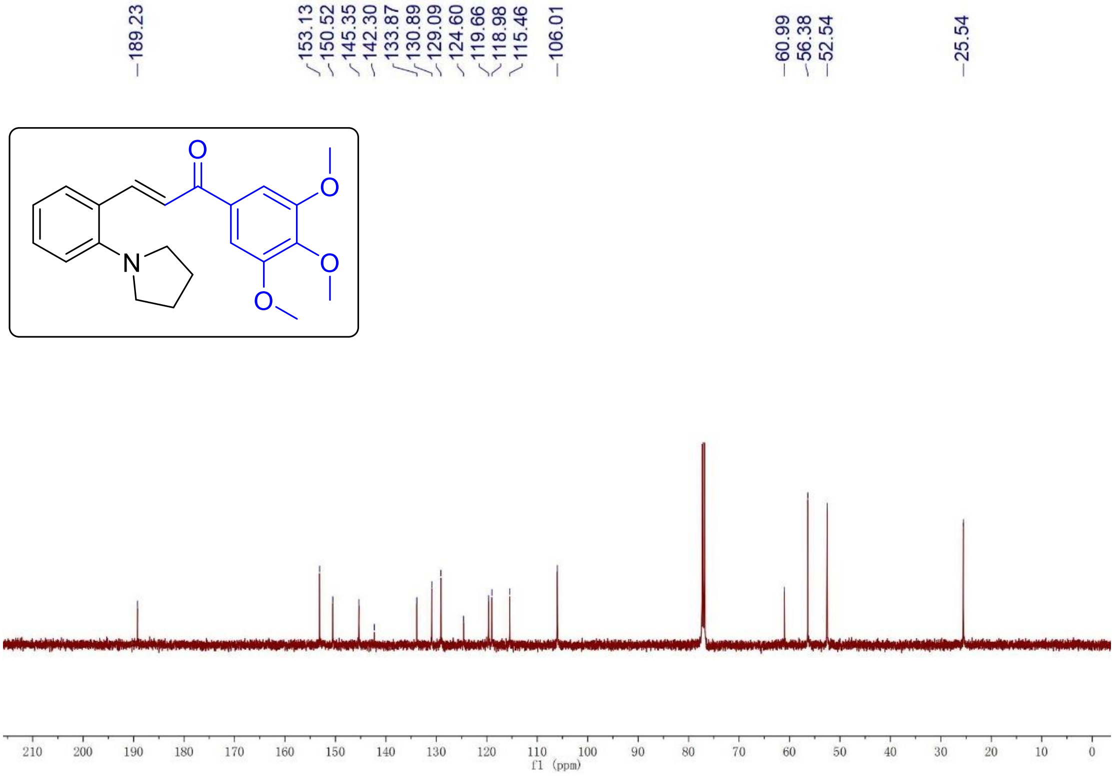


Supplementary Figure 65. $^{1}$H NMR and $^{13}$C NMR spectrum of **8m**.

**(E)-1-(naphthalen-2-yl)-3-(2-(pyrrolidin-1-yl)phenyl)prop-2-en-1-one (8n)**

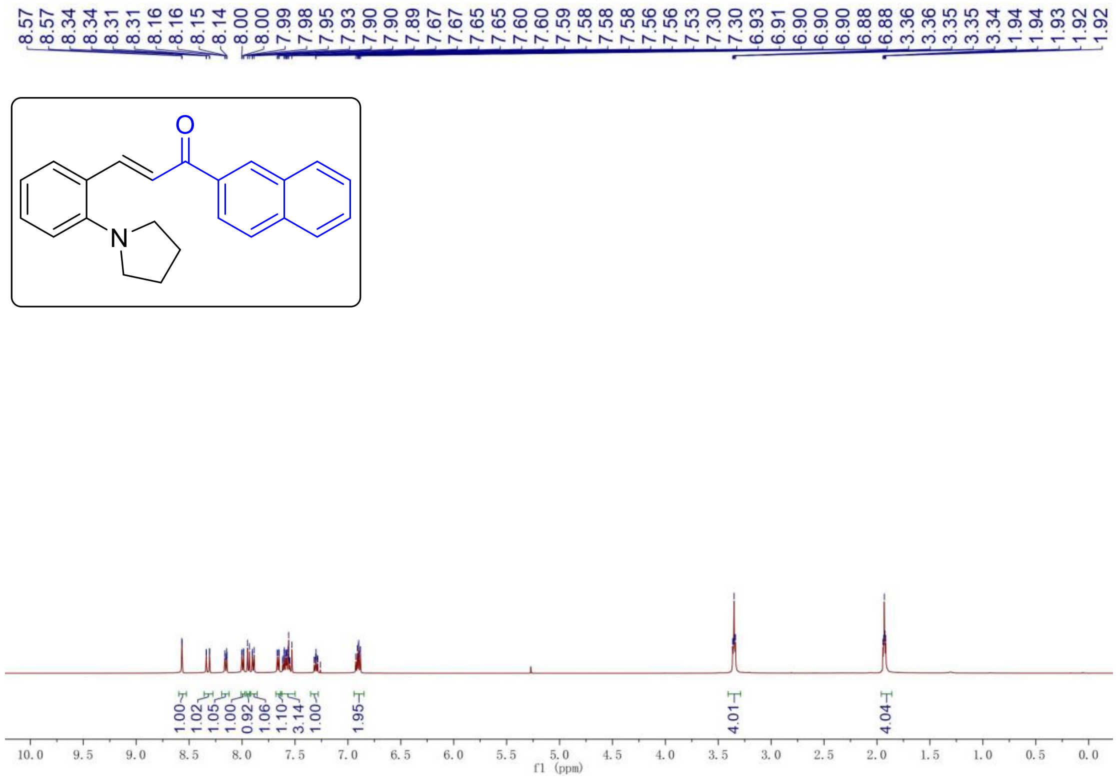


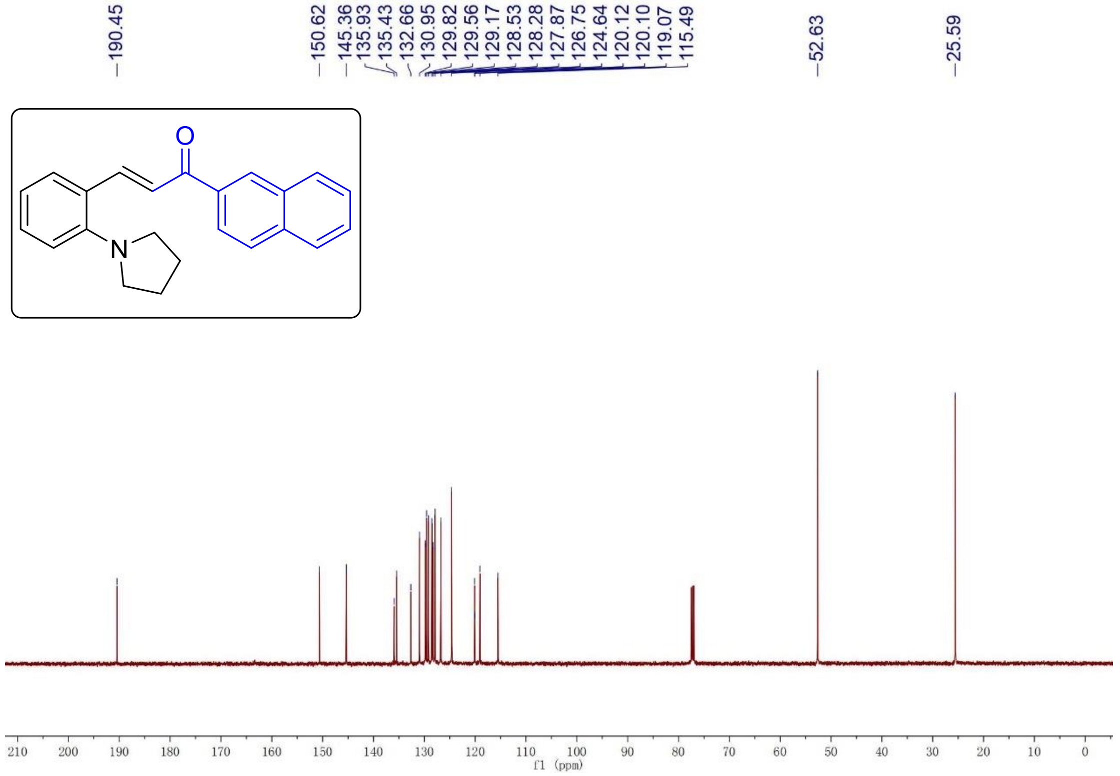


Supplementary Figure 66. $^{1}$H NMR and $^{13}$C NMR spectrum of **8n**.

## (E)-1-(furan-2-yl)-3-(2-(pyrrolidin-1-yl)phenyl)prop-2-en-1-one (8o)

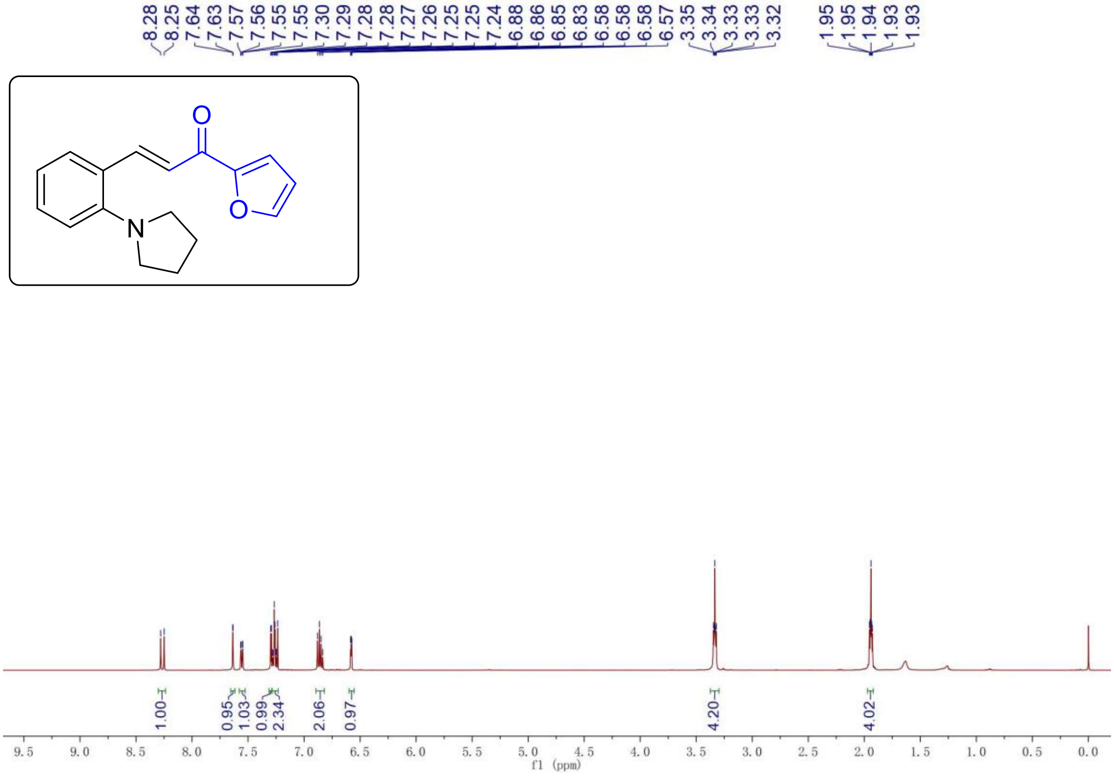


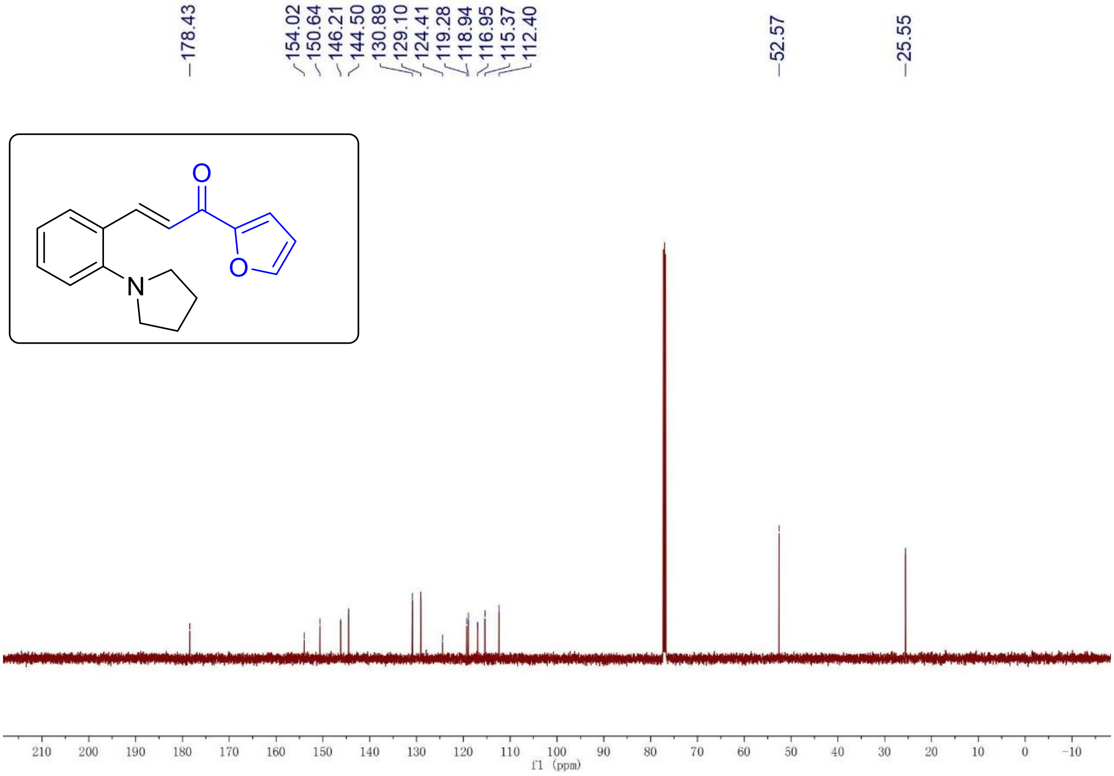


Supplementary Figure 67. $^{1}$H NMR and $^{13}$C NMR spectrum of **8o**.

**(E)-3-(2-(pyrrolidin-1-yl)phenyl)-1-(thiophen-2-yl)prop-2-en-1-one (8q)**

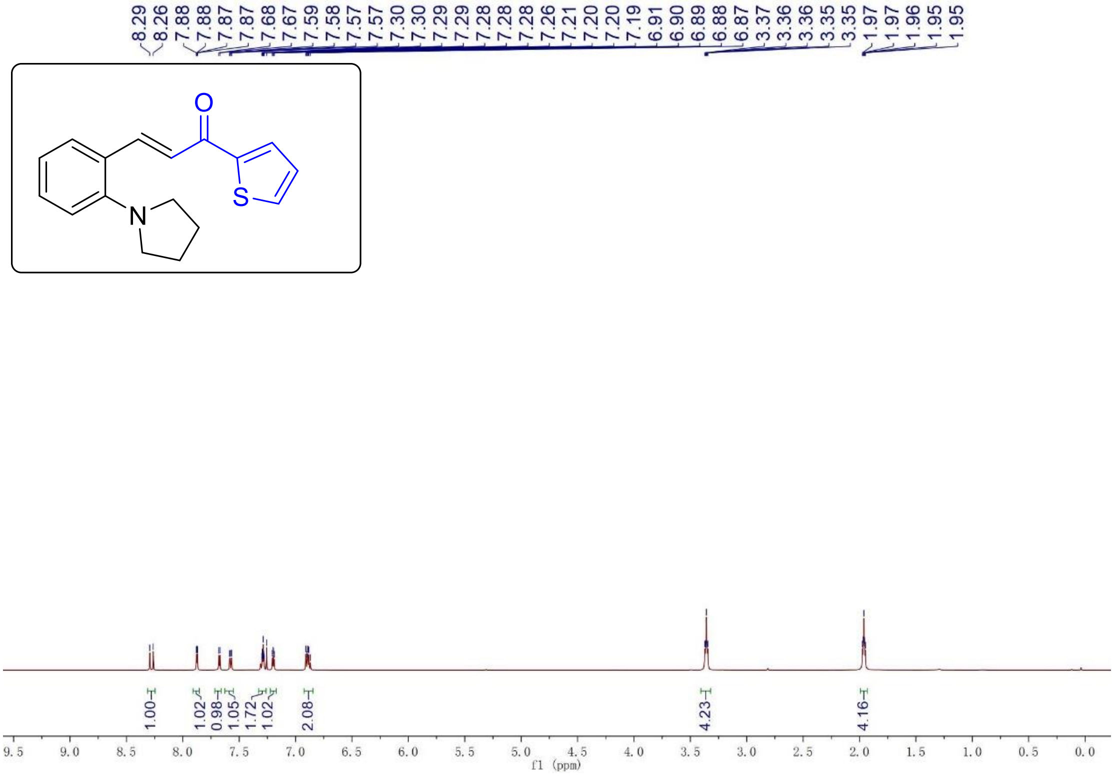


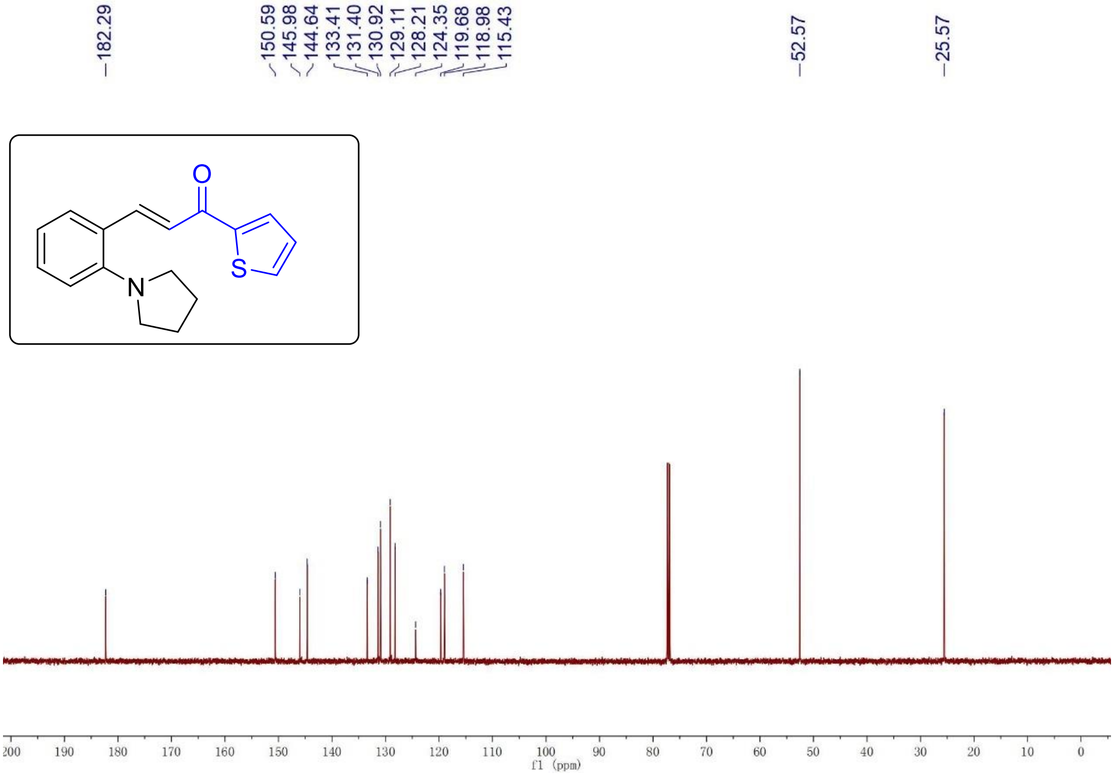


Supplementary Figure 68. $^{1}$H NMR and $^{13}$C NMR spectrum of **8q**.

**(E)-3-(5-methyl-2-(pyrrolidin-1-yl)phenyl)-1-phenylprop-2-en-1-one (8r)**

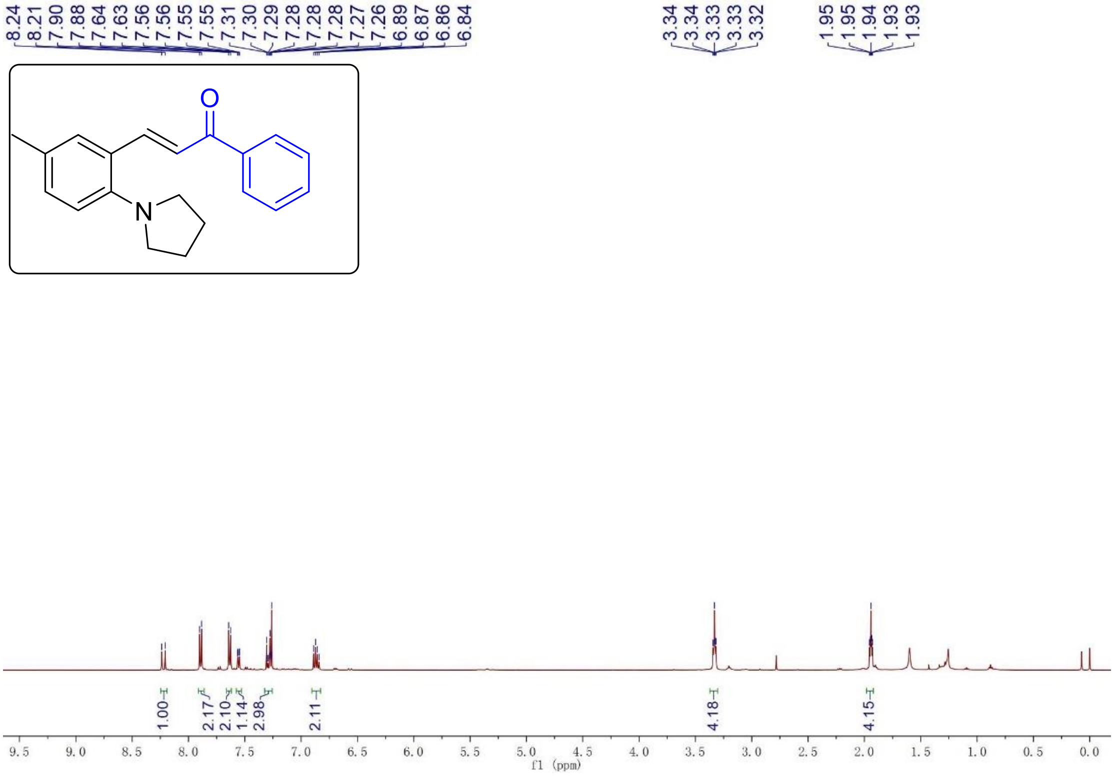


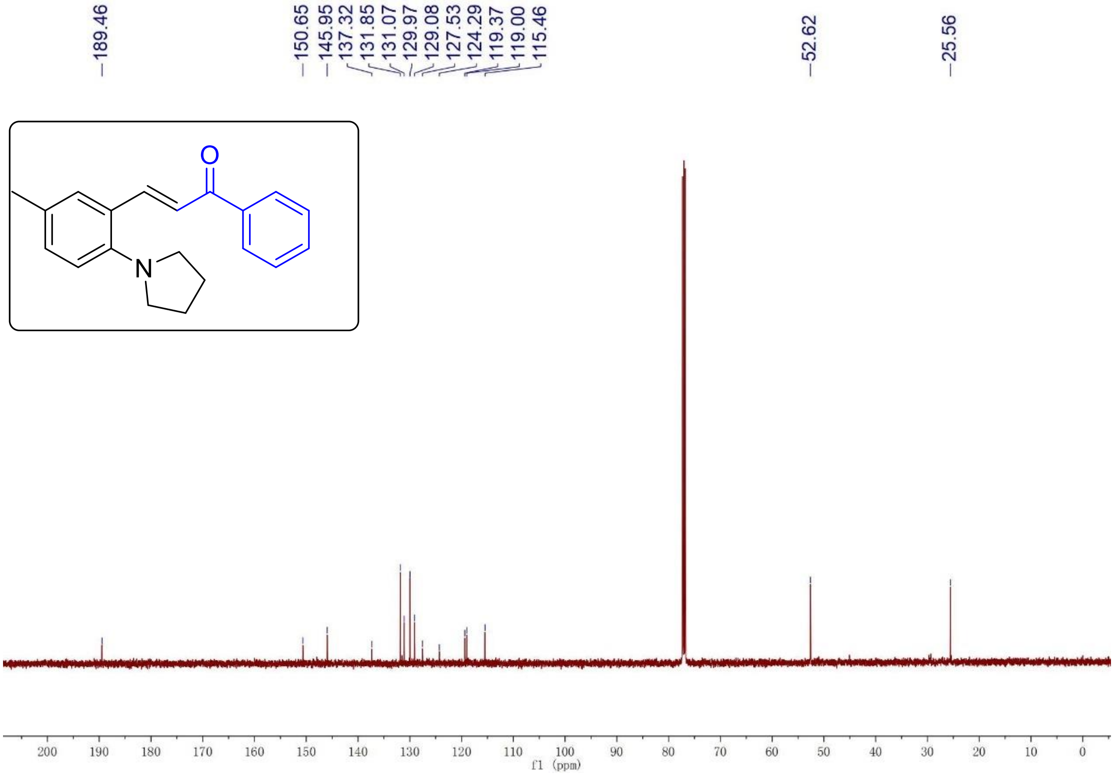


Supplementary Figure 69. $^{1}$H NMR and $^{13}$C NMR spectrum of **8r**.

**(E)-3-(4-methyl-2-(pyrrolidin-1-yl)phenyl)-1-phenylprop-2-en-1-one (8s)**

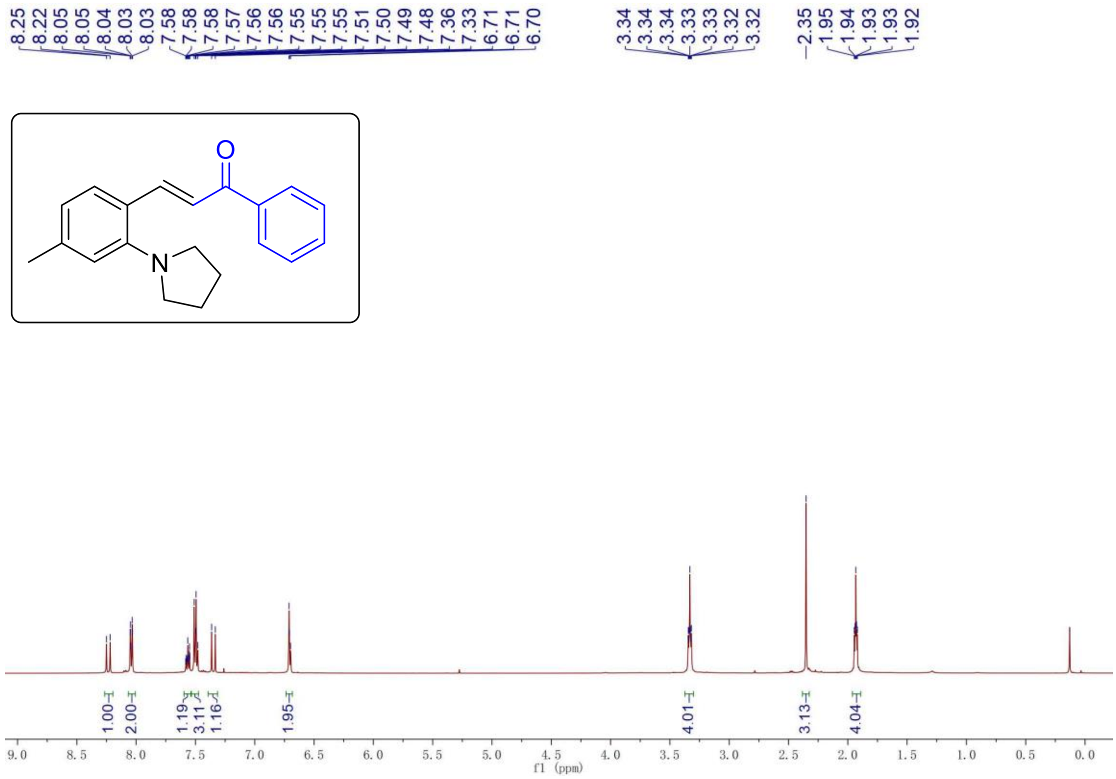


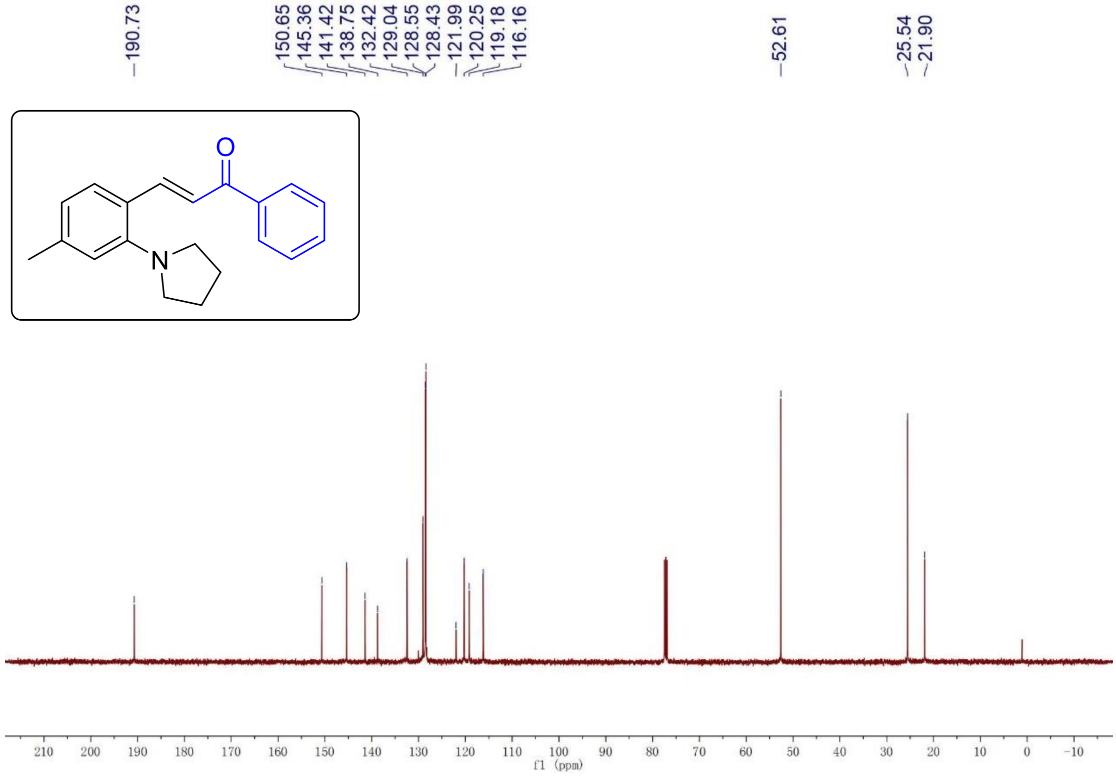


Supplementary Figure 70. $^{1}$H NMR and $^{13}$C NMR spectrum of **8s**.

**(E)-1-(2-(dimethylamino)phenyl)-5-phenylpent-1-en-3-one (8t)**

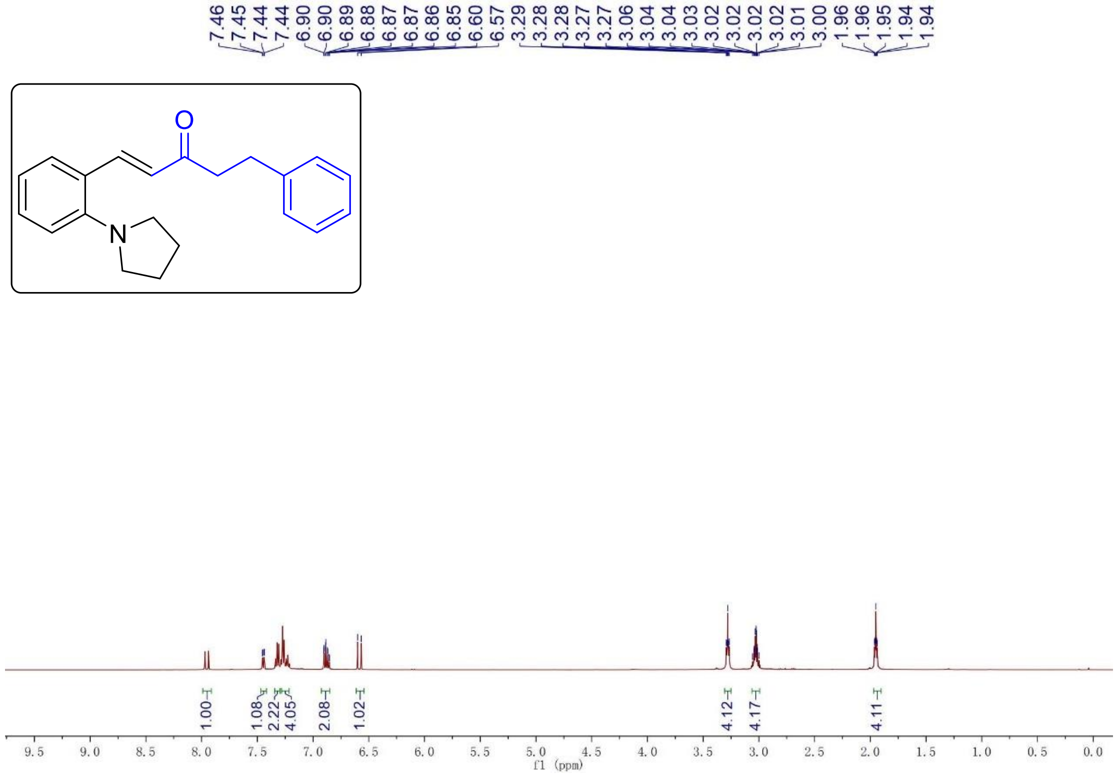


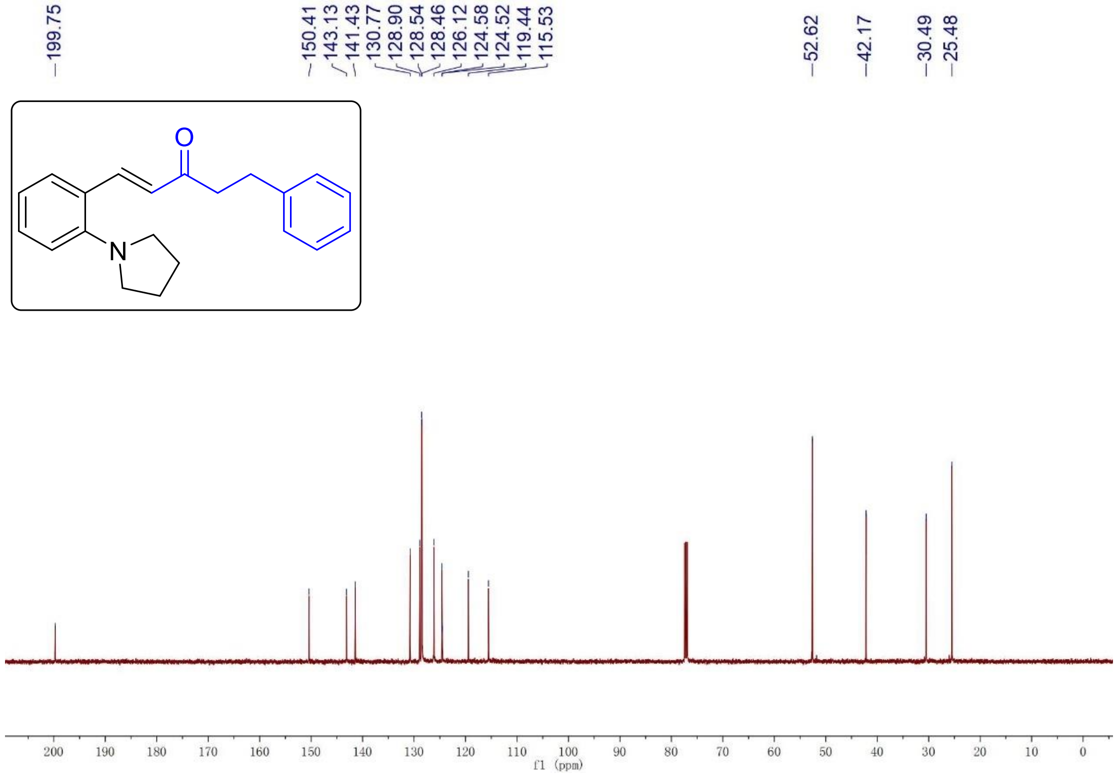


Supplementary Figure 71. $^{1}$H NMR and $^{13}$C NMR spectrum of **8t**.

**(E)-1-(2-(dimethylamino)phenyl)dec-1-en-3-one (8u)**

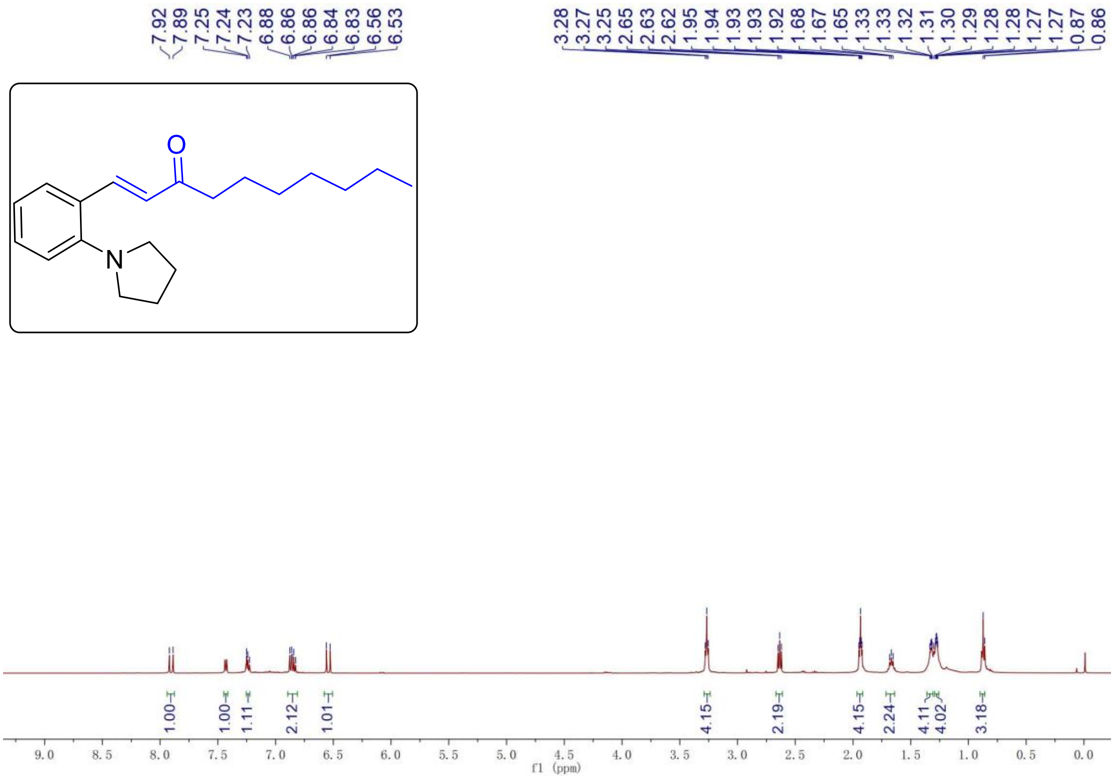


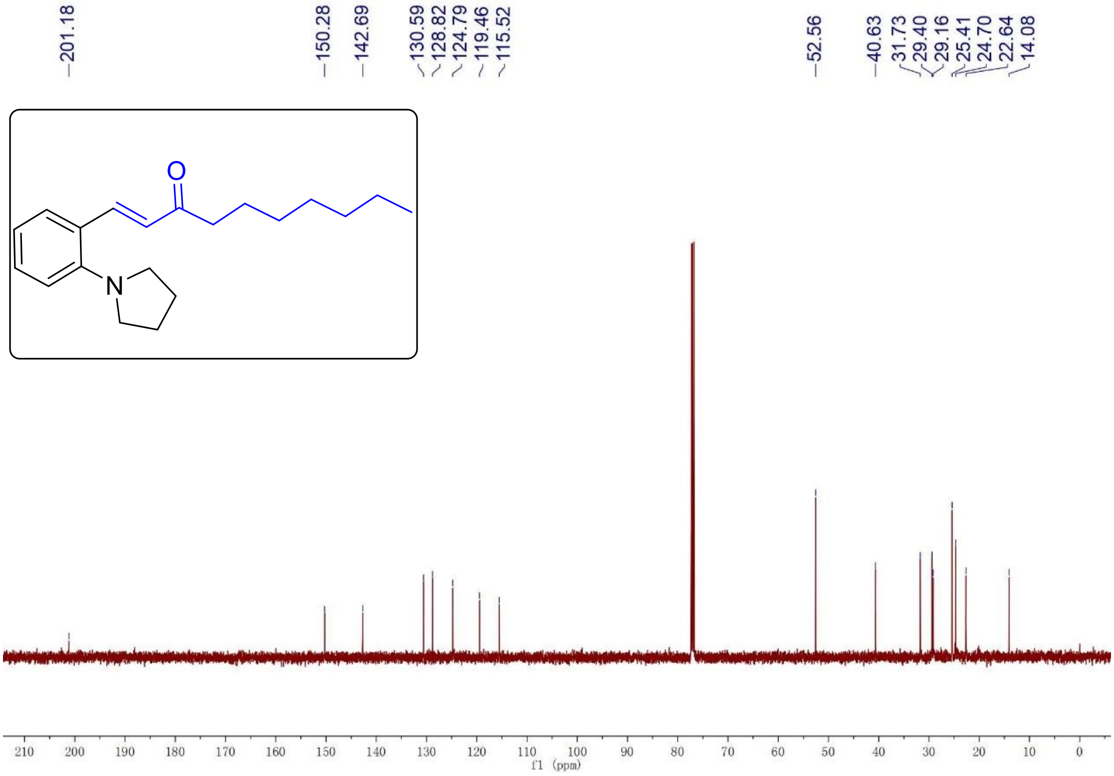


Supplementary Figure 72. $^{1}$H NMR and $^{13}$C NMR spectrum of **8u**.

**(E)-1-acetylferroceneyl-3-(2-(dimethylamino)phenyl)prop-2-en-1-one (1z) (10a)**

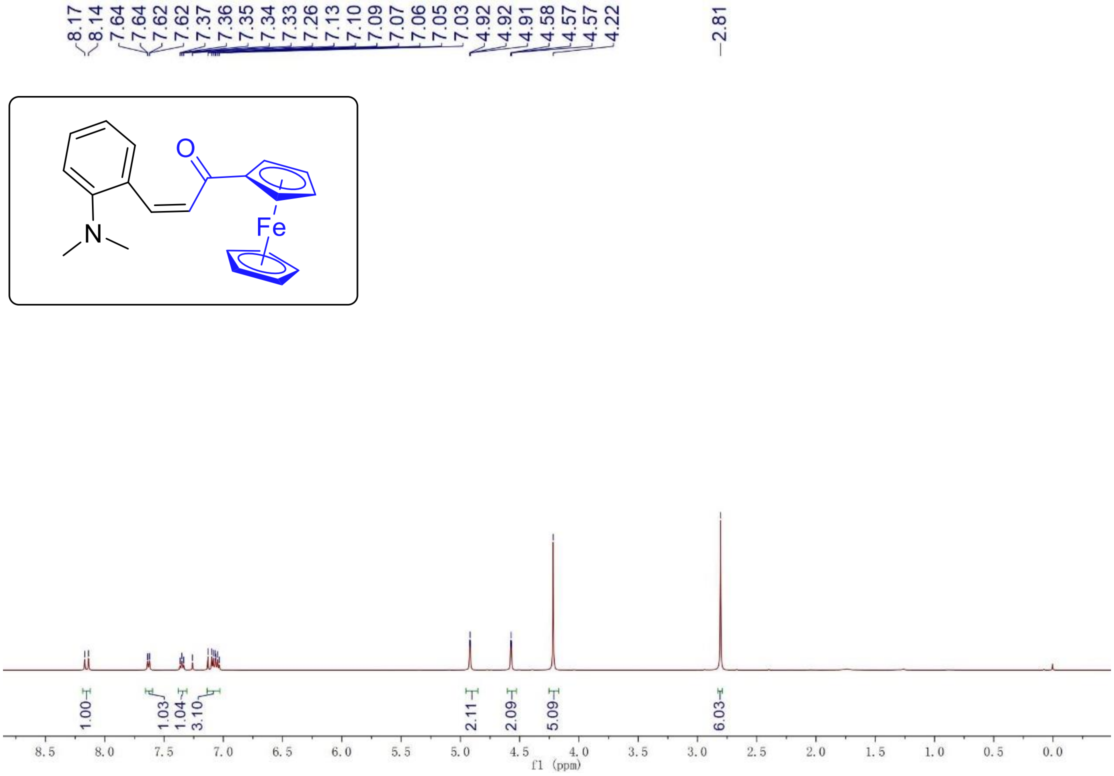


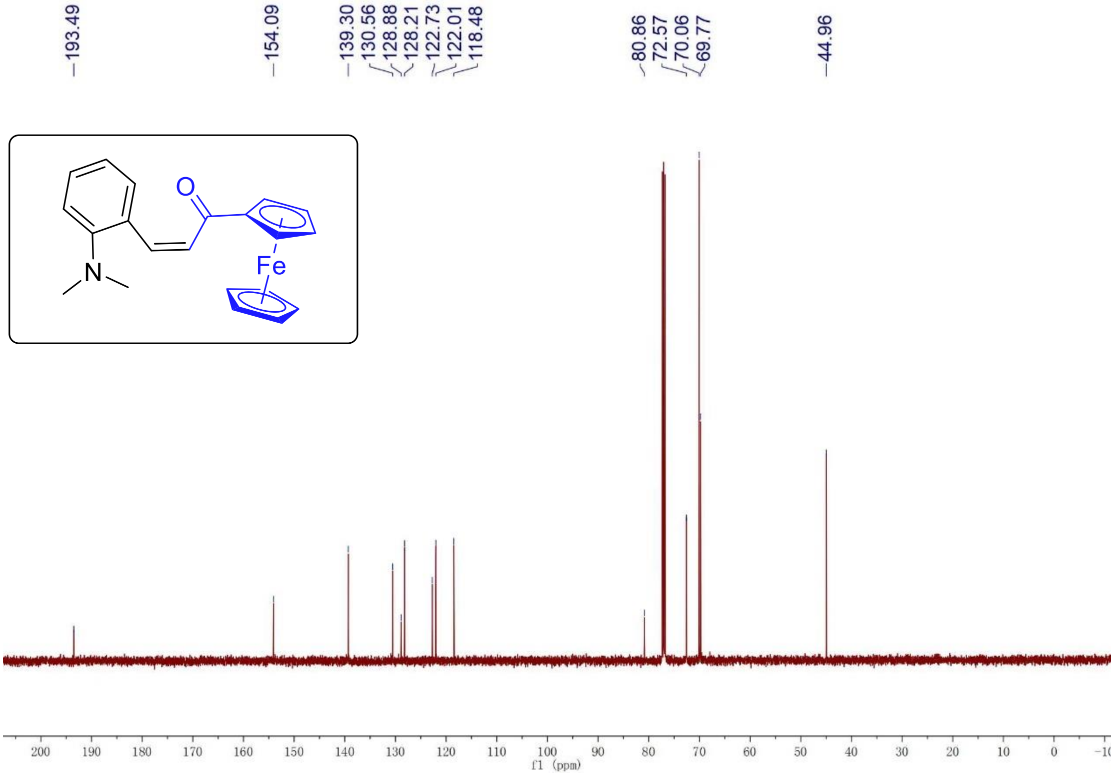


Supplementary Figure 73. $^{1}$H NMR and $^{13}$C NMR spectrum of **10a**.

**(E)-4-(3-(2-(dimethylamino)phenyl)acryloyl)phenyl(tert-butoxycarbonyl)-D-valinate (10b)**

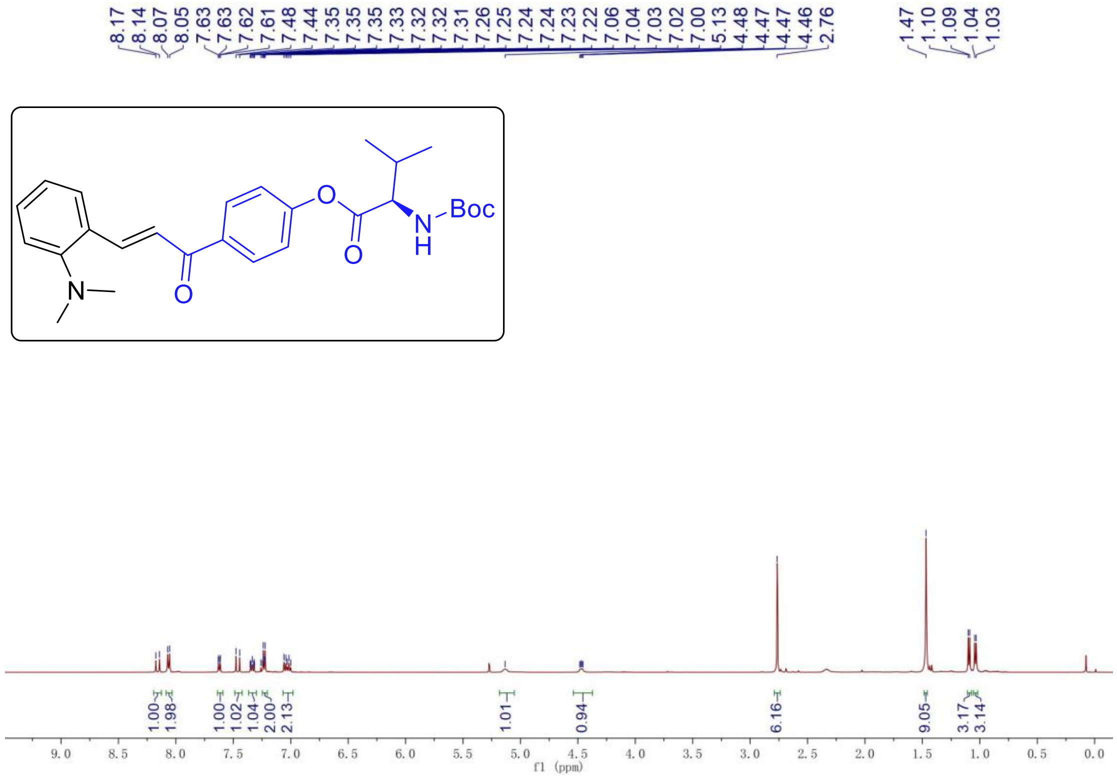


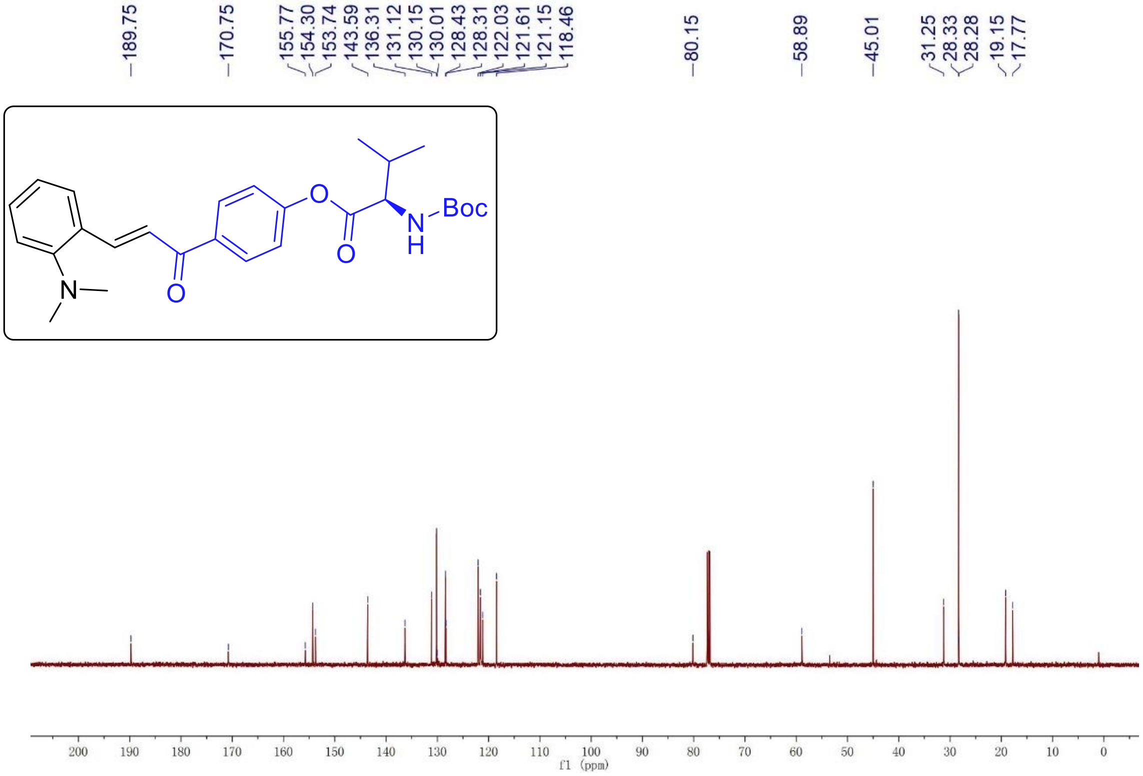


Supplementary Figure 74. $^{1}$H NMR and $^{13}$C NMR spectrum of **10b**.

**(E)-1-(tert-butyl)2-(4-(3-(2-(dimethylamino)phenyl)acryloyl)phenyl)(S)-pyrrolidine-1,2-dicarboxylate (10c)**

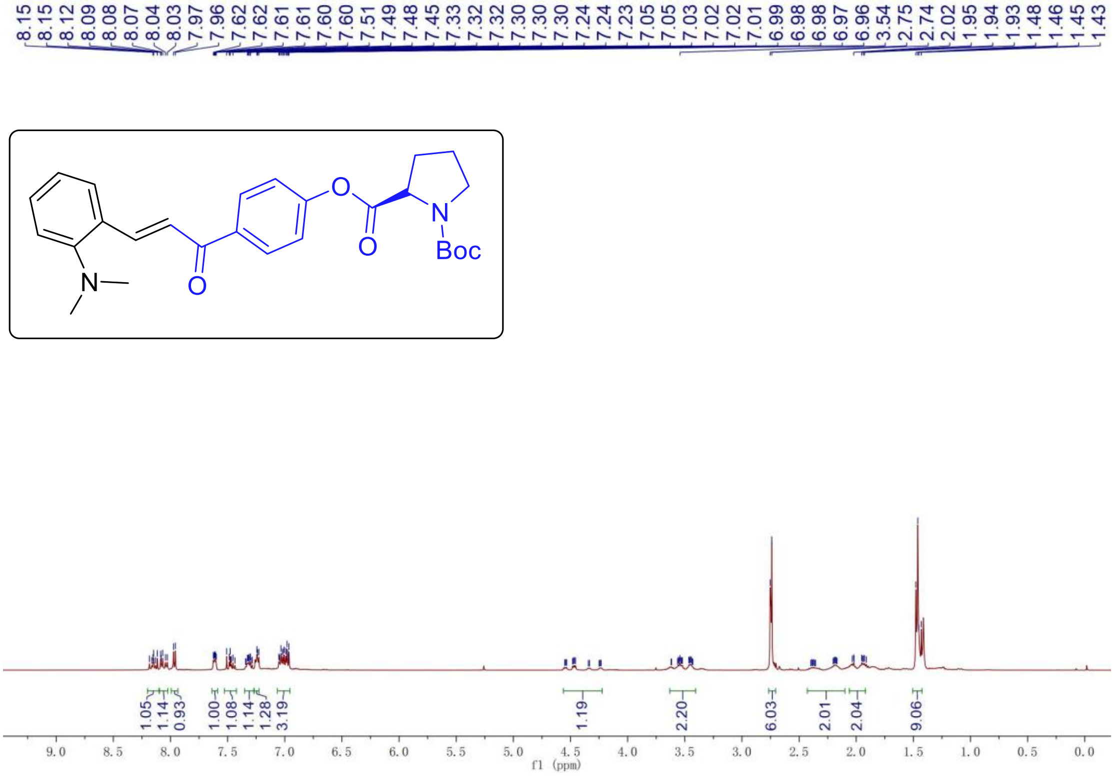


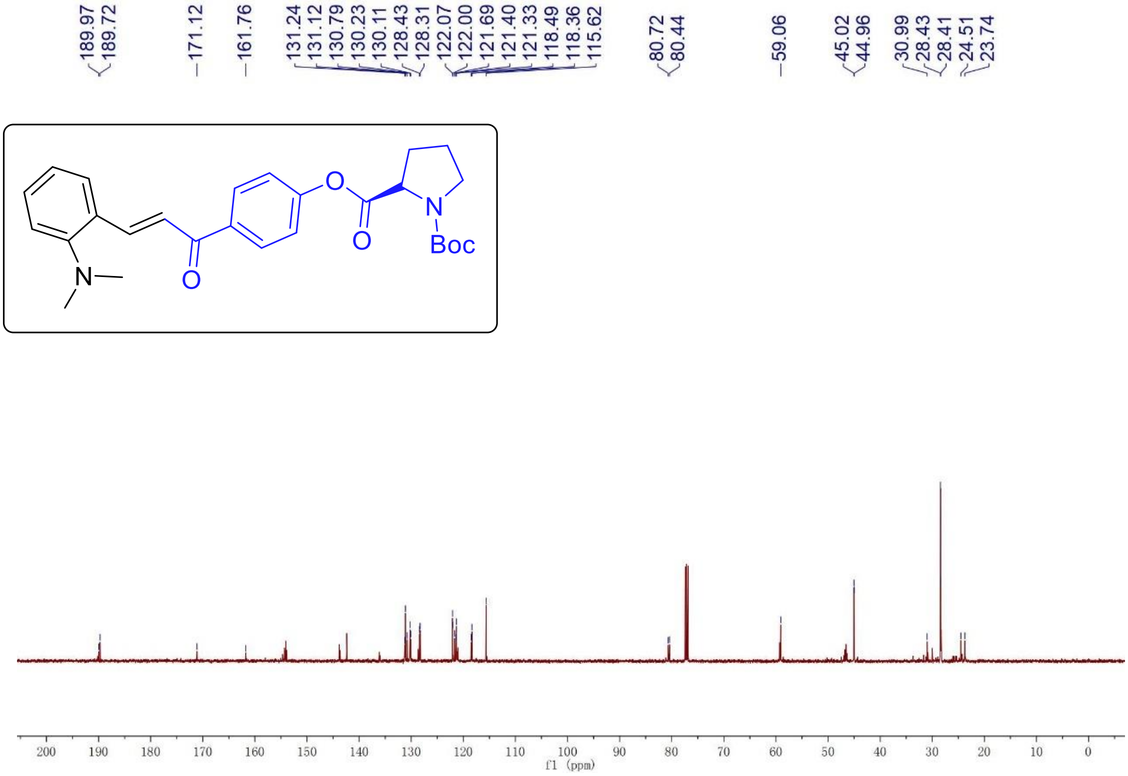


Supplementary Figure 75. $^{1}$H NMR and $^{13}$C NMR spectrum of **10c**.

**(E)-3-(2-(dimethylamino)phenyl)-1-((3S,8S,9S,10R,14S,17R)-3-hydroxy-10-methyl-2,3,4,7,8,9,10,11,12,13,14,15,16,17-tetradecahydro-1H-cyclopenta[a]phenanthren-17-yl)prop-2-en-1-one (10d)**

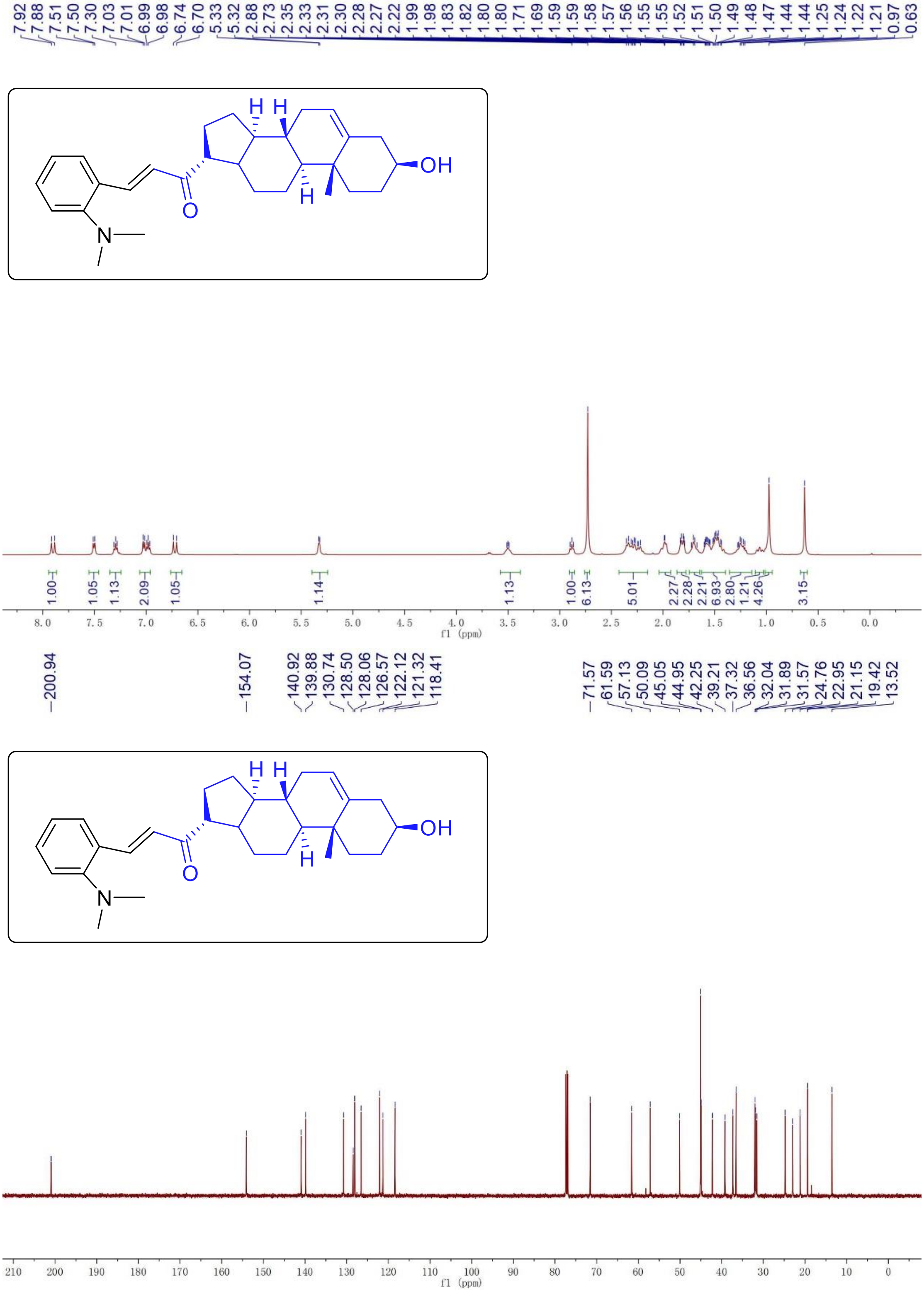


Supplementary Figure 76. $^1$H NMR and $^{13}$C NMR spectrum of **10d**.

**(E)-1-((1r,3R,5S,7s)-adamantan-1-yl)-3-(2-(dimethylamino)phenyl)prop-2-en-1-one (10e)**

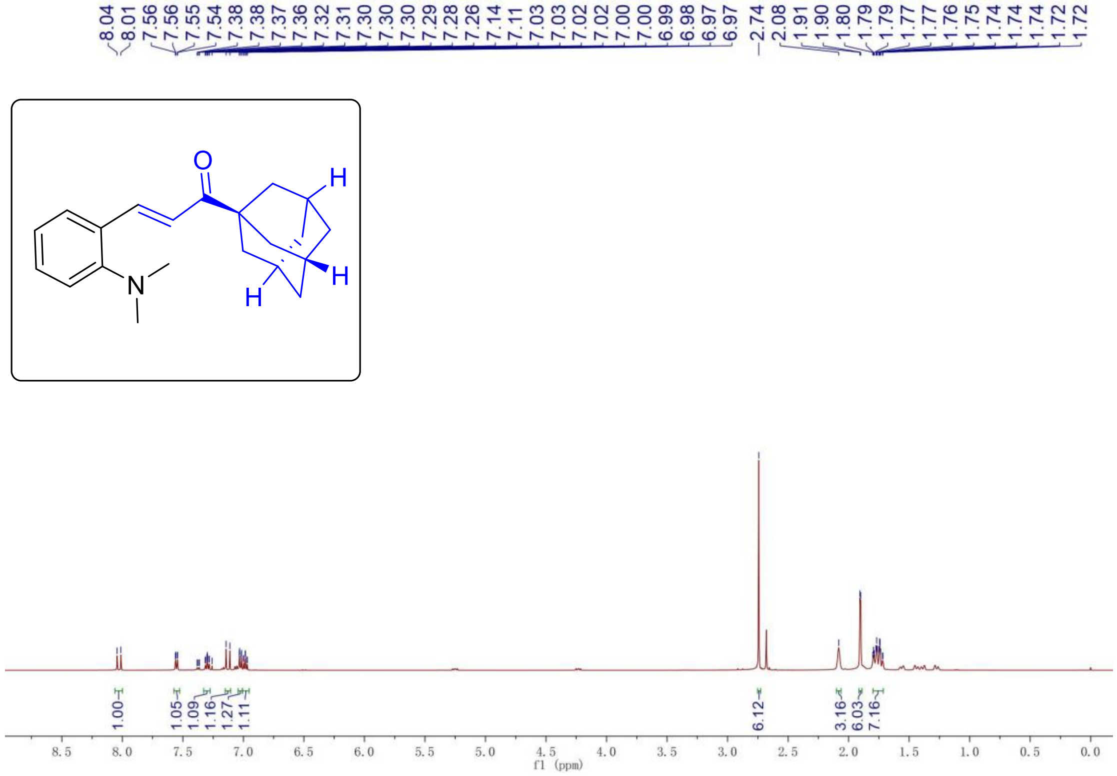


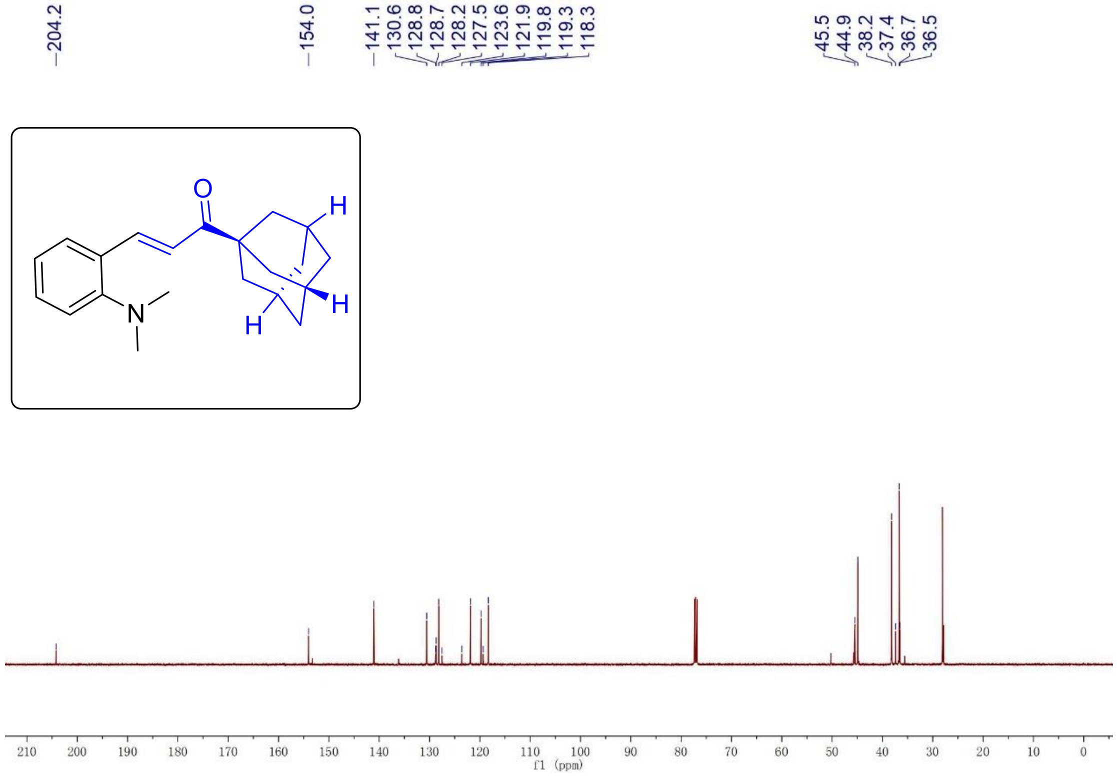


Supplementary Figure 77. $^{1}$H NMR and $^{13}$C NMR spectrum of **10e**.

**(8S,9S,10R,14S,17R)-17-((E)-3-(2-(dimethylamino)phenyl)acryloyl)-10-methyl-1,2,6,7,8,9,10,11,12,13,14,15,16,17-tetradecahydro-3H-cyclopenta[a]phenanthren-3-one (10f)**

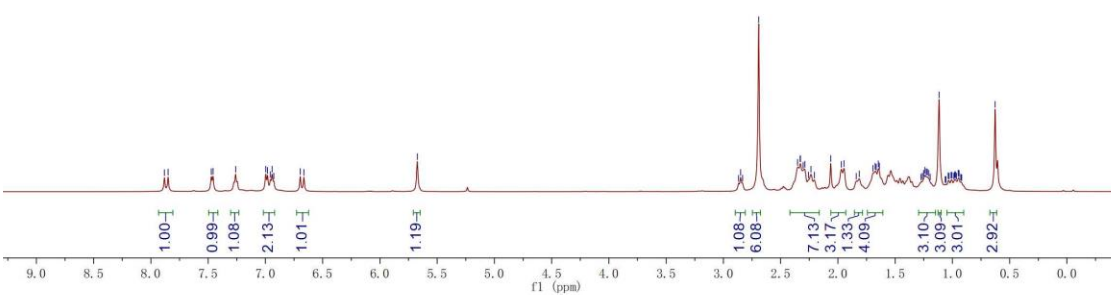


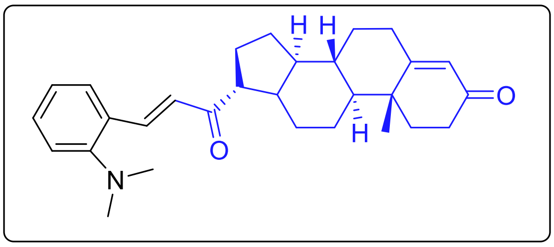

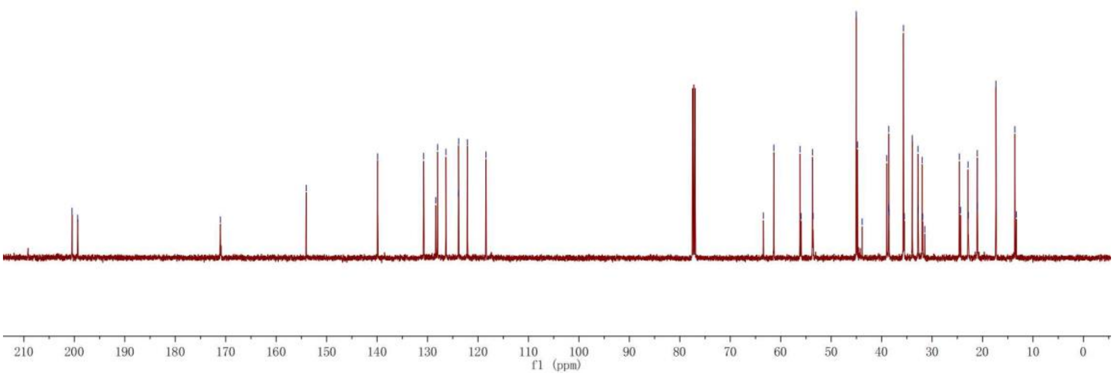


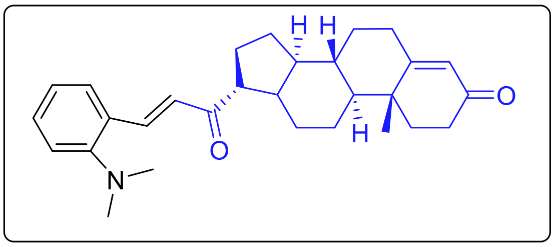

Supplementary Figure 78. $^{1}$H NMR and $^{13}$C NMR spectrum of **10f**.

**(E)-3,7-dimethylocta-2,6-dien-1-yl (E)-3-(2-(dimethylamino)phenyl)acrylate (10g)**

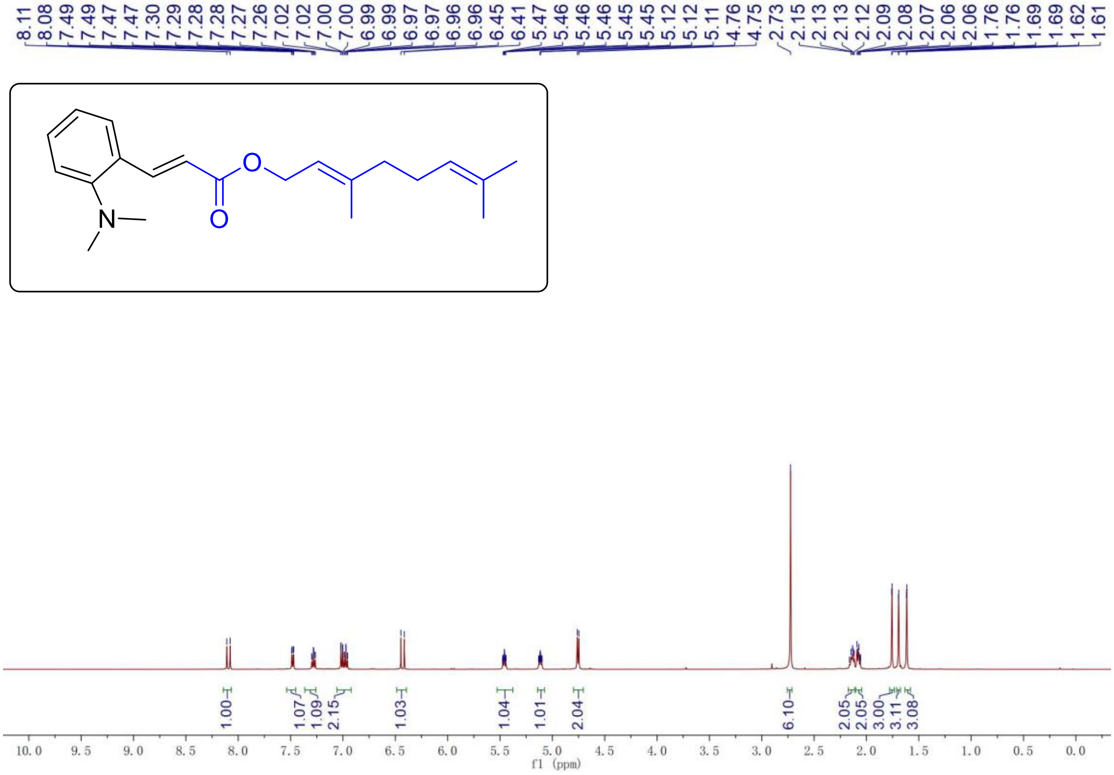


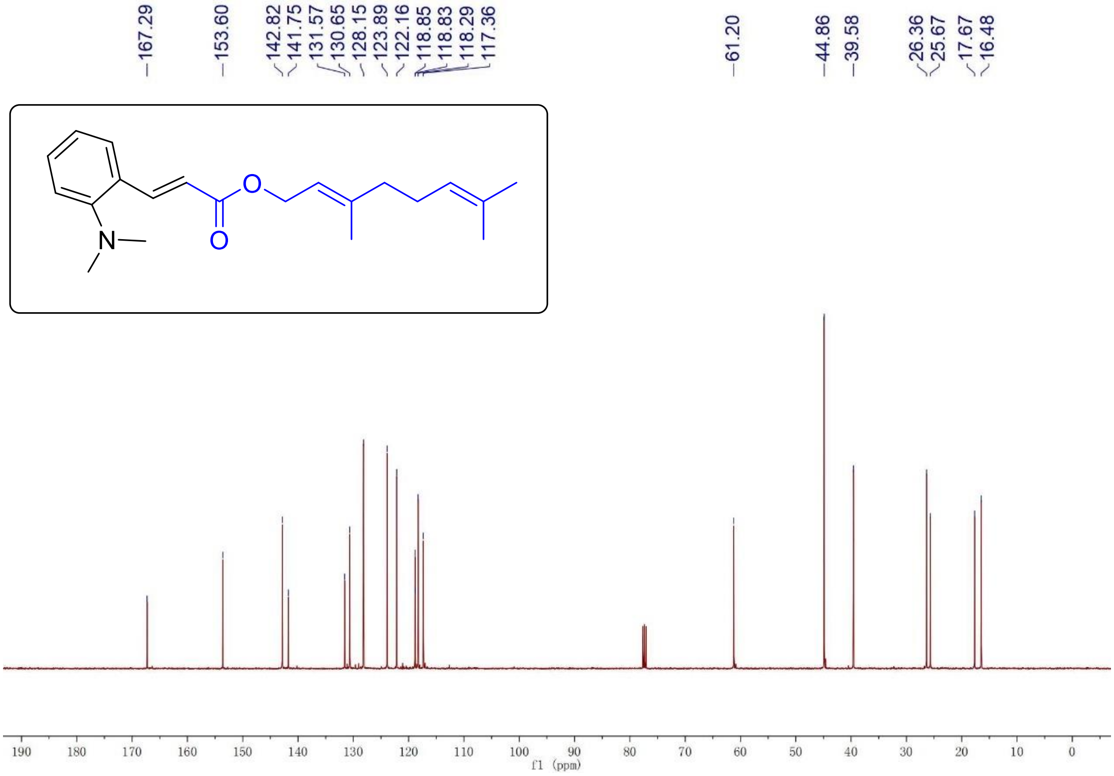


Supplementary Figure 79. $^{1}$H NMR and $^{13}$C NMR spectrum of **10g**.

**2-((1R,5S)-6,6-dimethylbicyclo[3.1.1]hept-2-en-3-yl)ethyl(E)-3-(2-(dimethylamino)phenyl)acrylate (10h)**

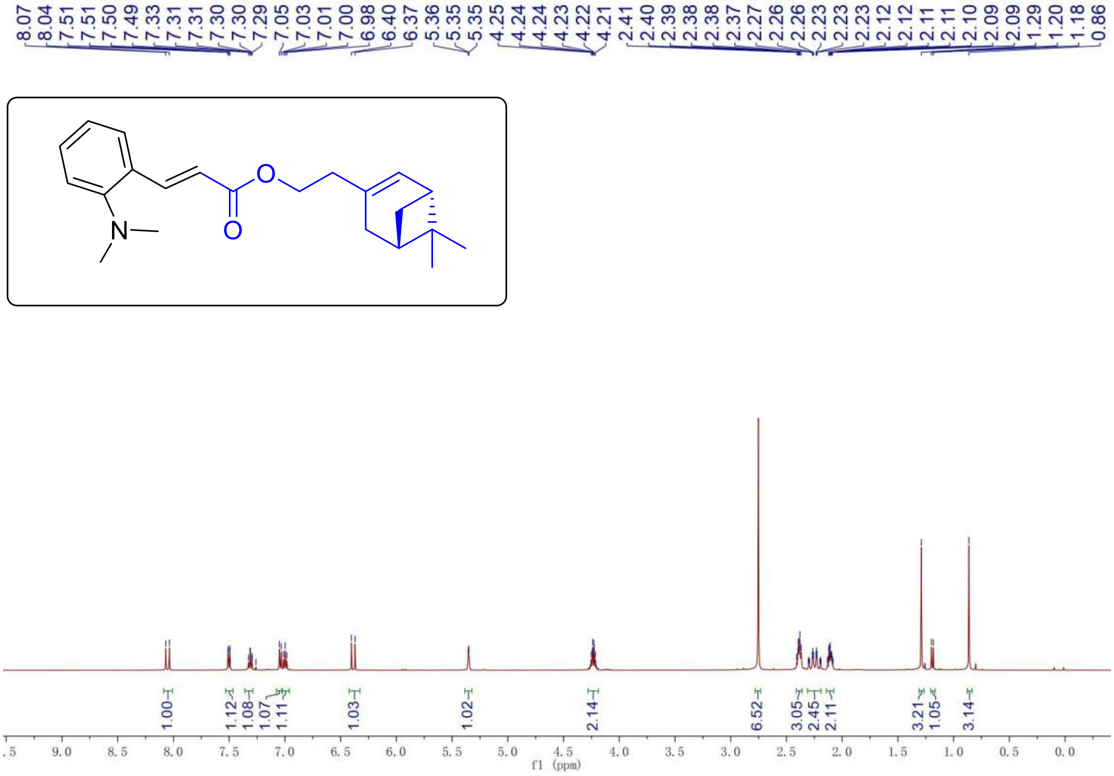


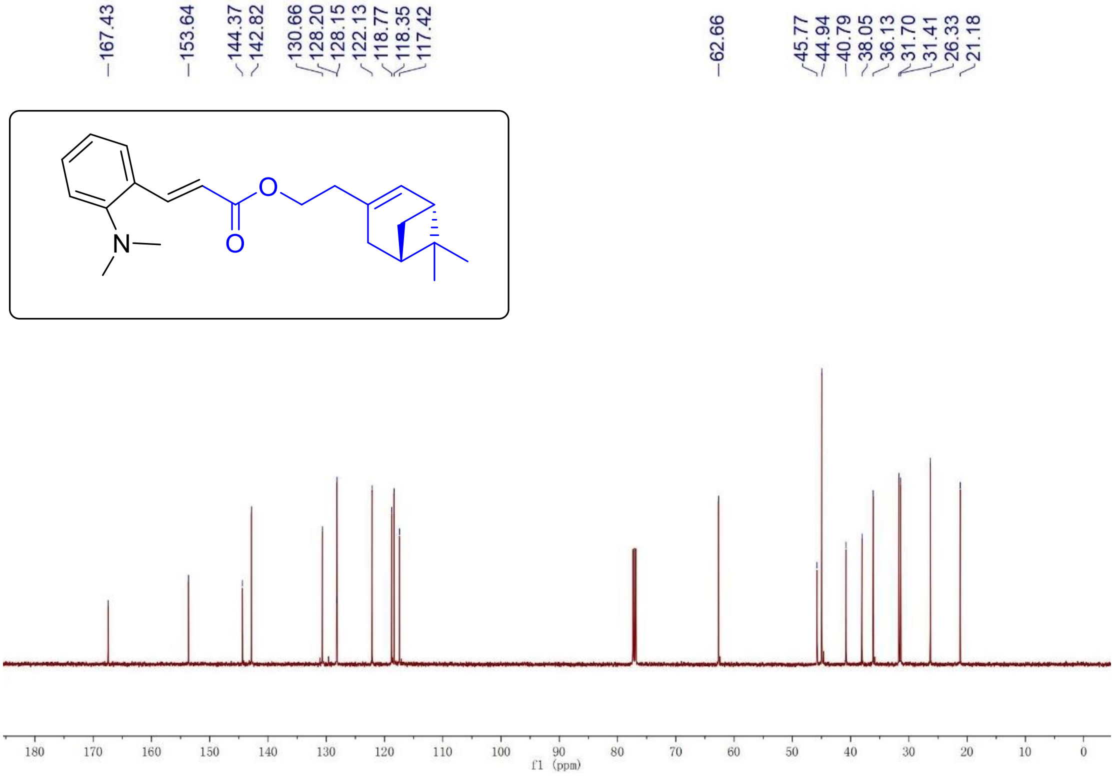


Supplementary Figure 80. $^1$H NMR and $^{13}$C NMR spectrum of **10h**.

**(1R,2S,5R)-2-isopropyl-5-methylcyclohexyl(E)-3-(2-(dimethylamino)phenyl)acrylate (10i)**

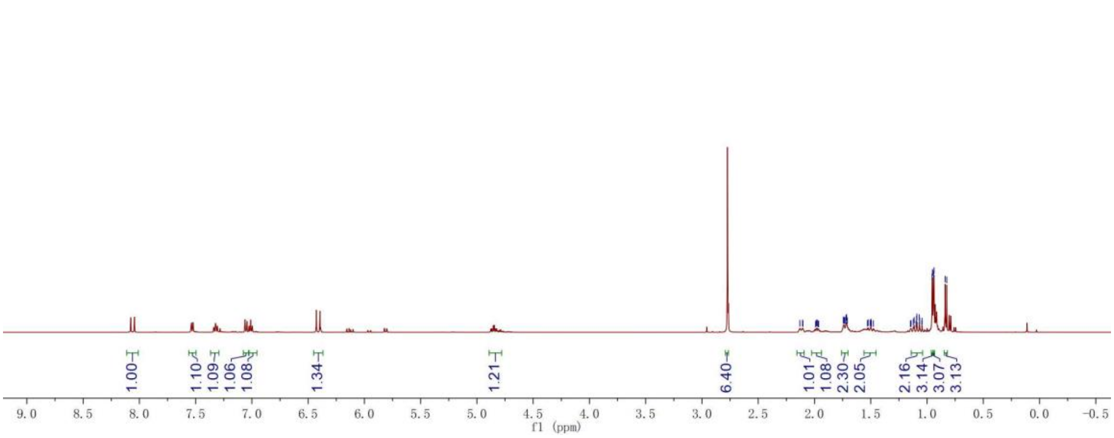


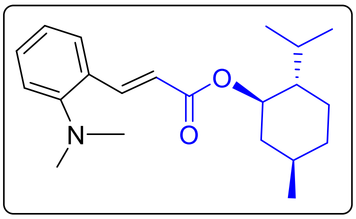


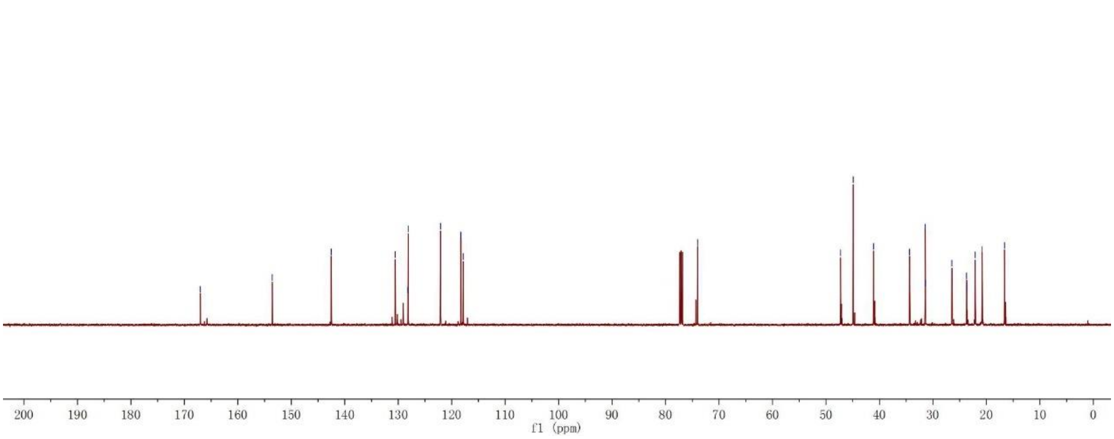


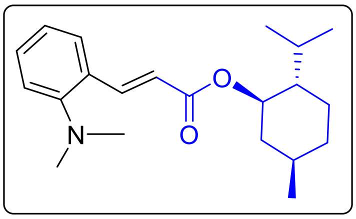


Supplementary Figure 81. $^{1}$H NMR and $^{13}$C NMR spectrum of **10i**.

**(R)-3,7-dimethyloct-6-en-1-yl (E)-3-(2-(dimethylamino)phenyl)acrylate (10j)**

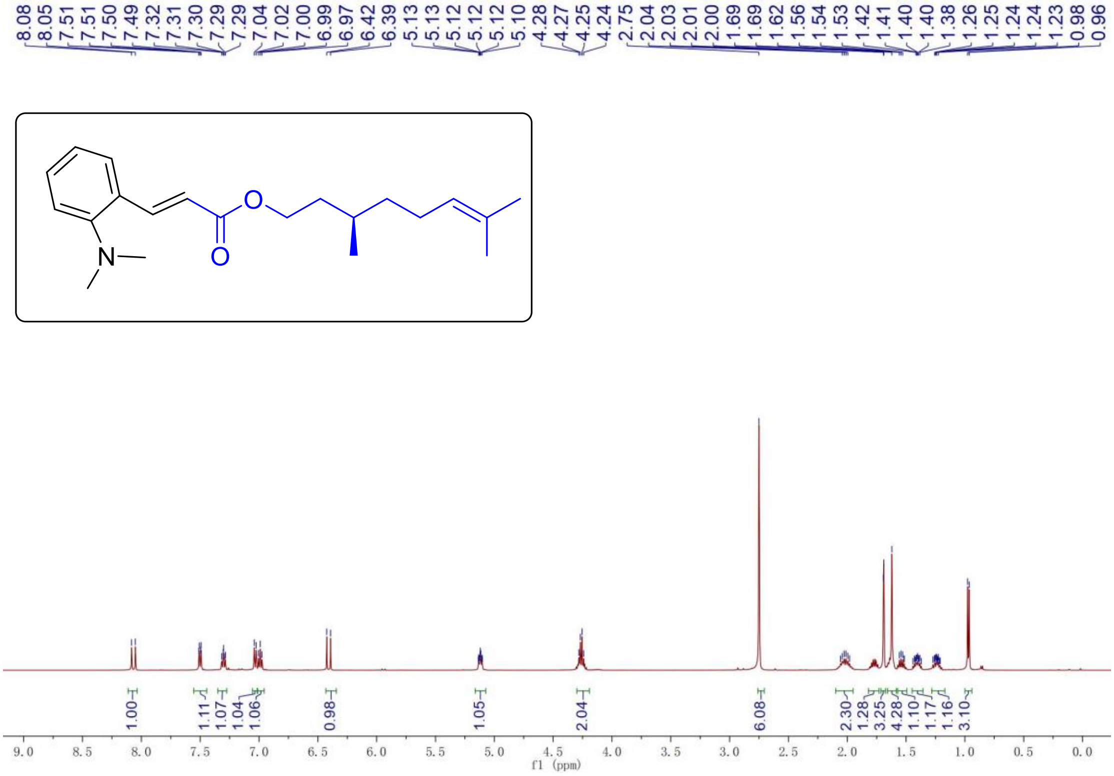


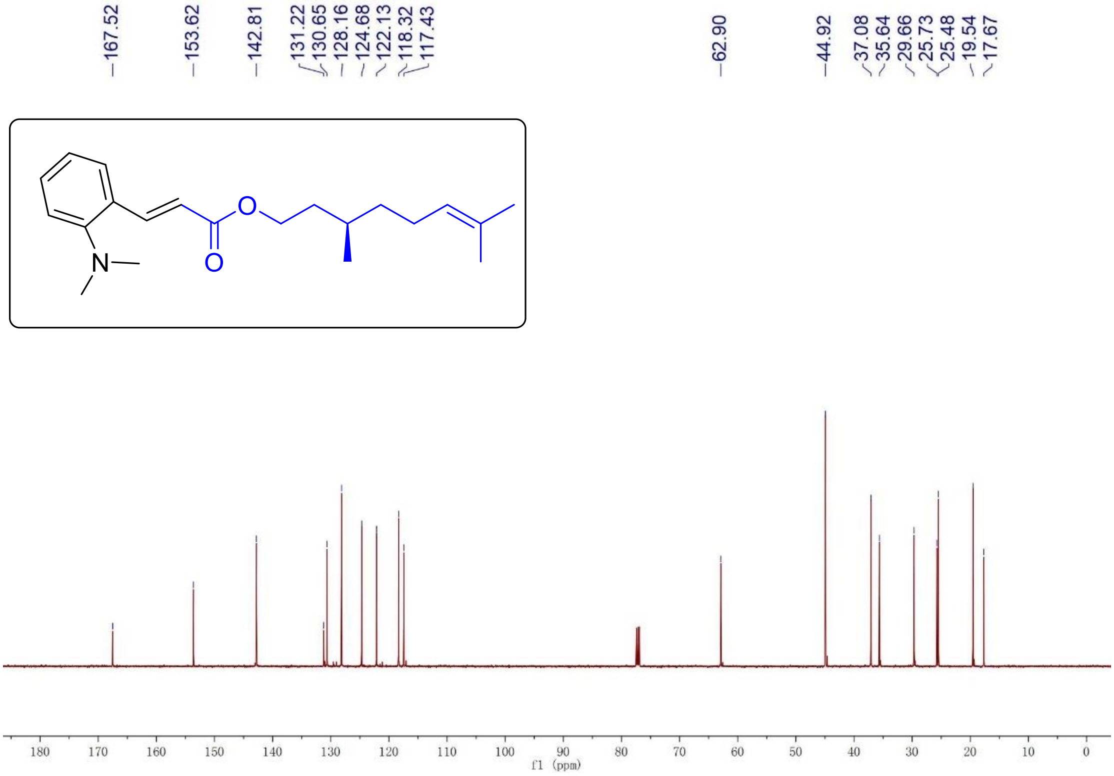


Supplementary Figure 82. $^{1}$H NMR and $^{13}$C NMR spectrum of **10j**.

**(3aS,5R,6R,6aS)-5-((S)-2,2-dimethyl-1,3-dioxolan-4-yl)-2,2-dimethyltetrahydrofuro[2,3-d][1,3]dioxol-6-yl(E)-3-(2-(dimethylamino)phenyl)acrylate (10k)**

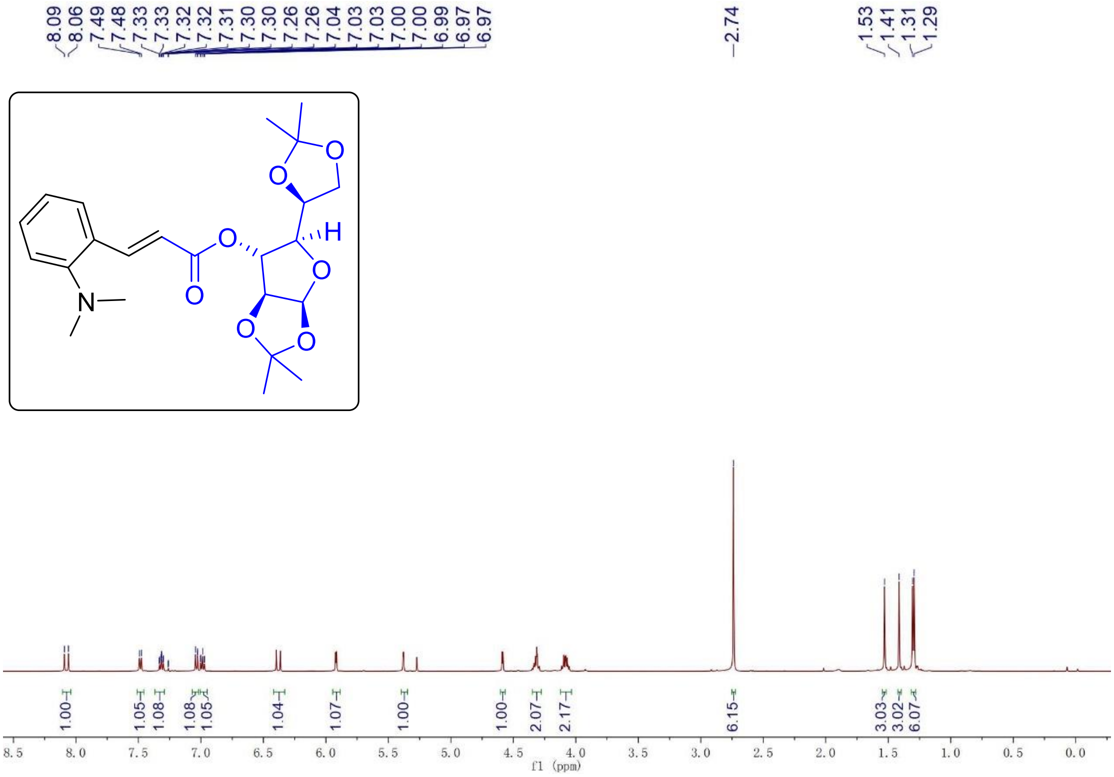


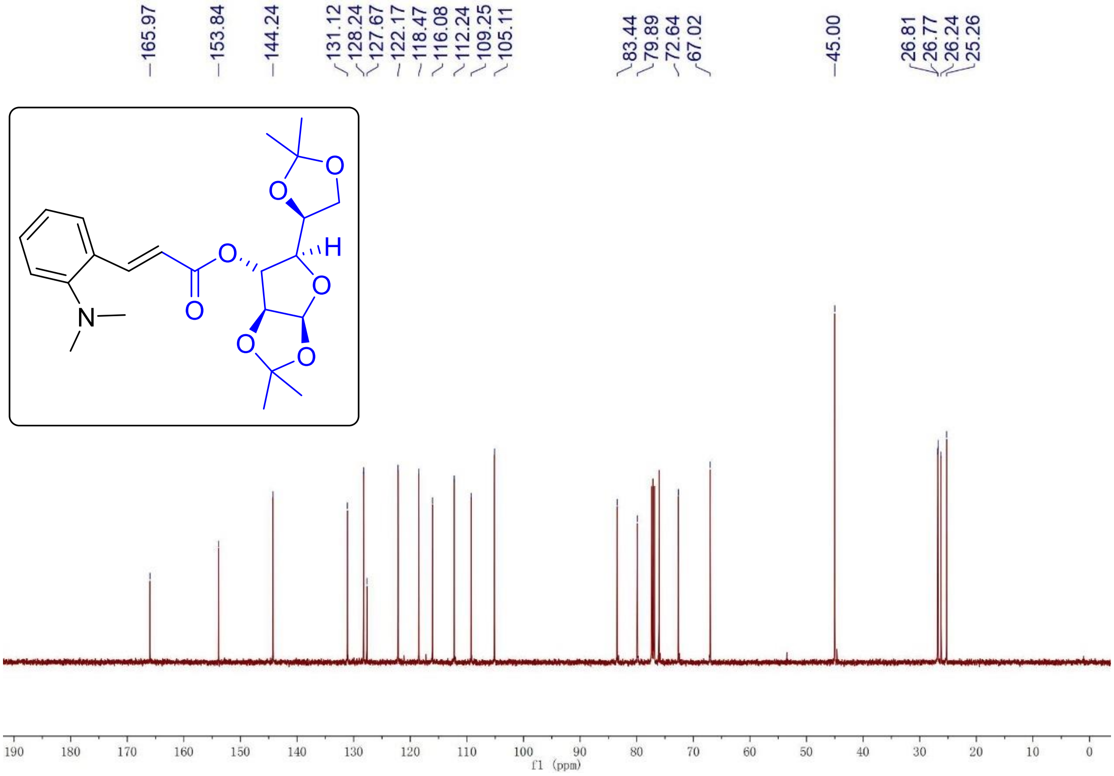


Supplementary Figure 83. $^{1}$H NMR and $^{13}$C NMR spectrum of **10k**.

**(3S,8S,9S,10R,13R,14S,17R)-10,13-dimethyl-17-((R)-6-methylheptan-2-yl)-2,3,4,7,8,9,10,11,12,13,14,15,16,17-tetradecahydro-1H-cyclopenta[a]phenanthren-3-yl (E)-3-(2-(dimethylamino)phenyl)acrylate (10l)**

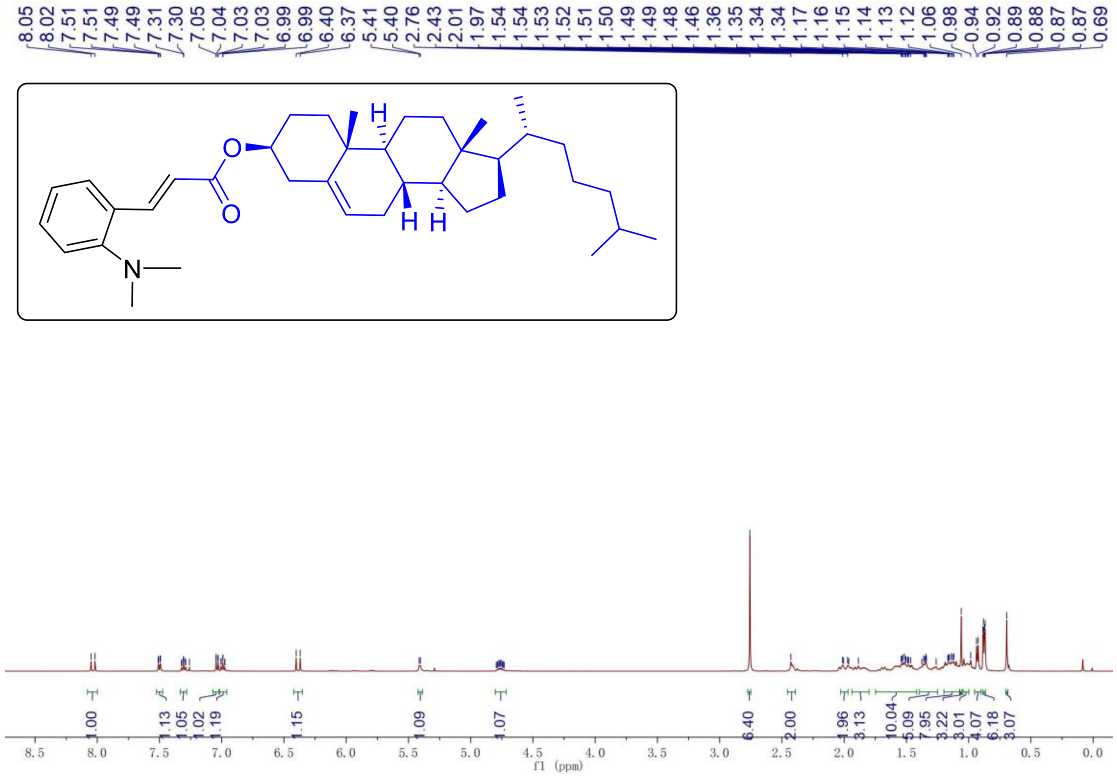


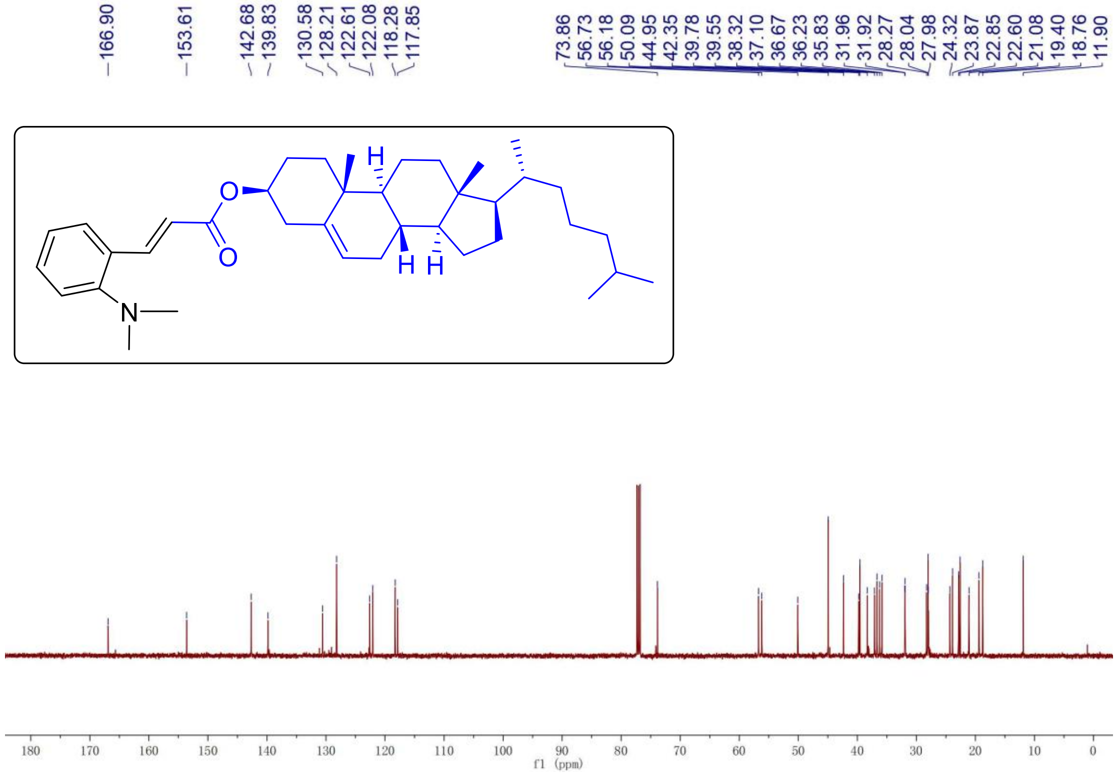


Supplementary Figure 84. $^{1}H$ NMR and $^{13}C$ NMR spectrum of **10l**.

**(E)-4-(3-(2-(dimethylamino)phenyl)acryloyl)phenyl(R)-2-(4-isobutylphenyl)propanoate (10m)**

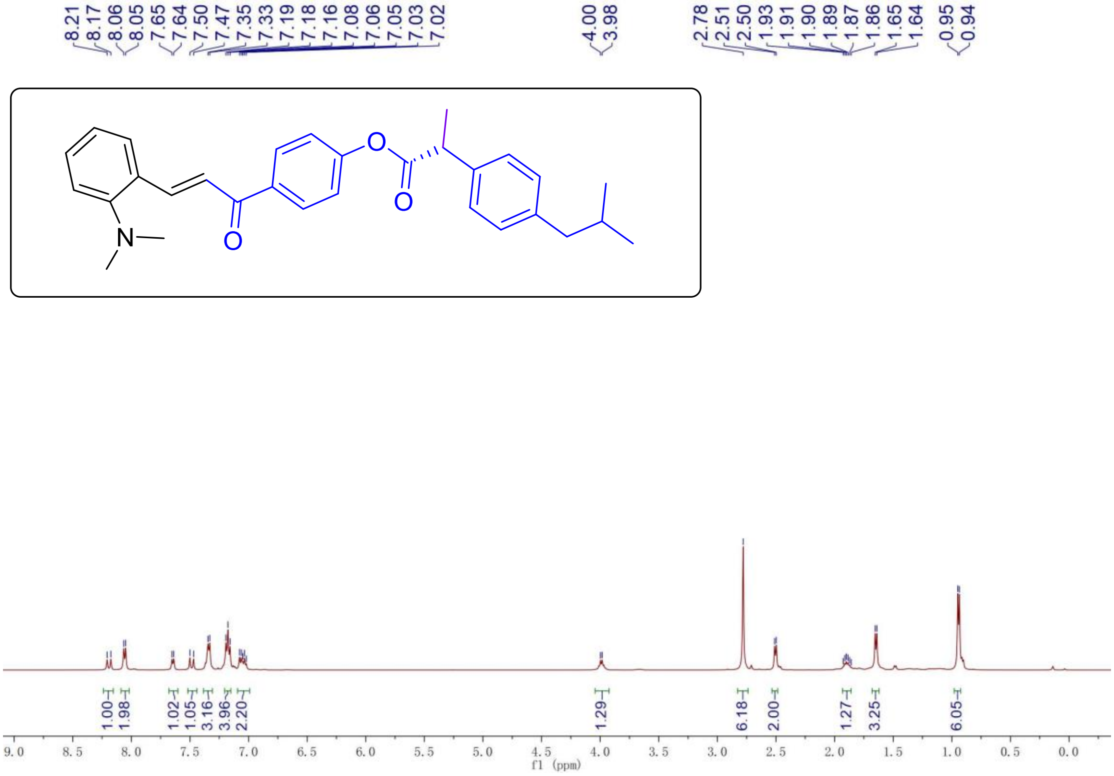


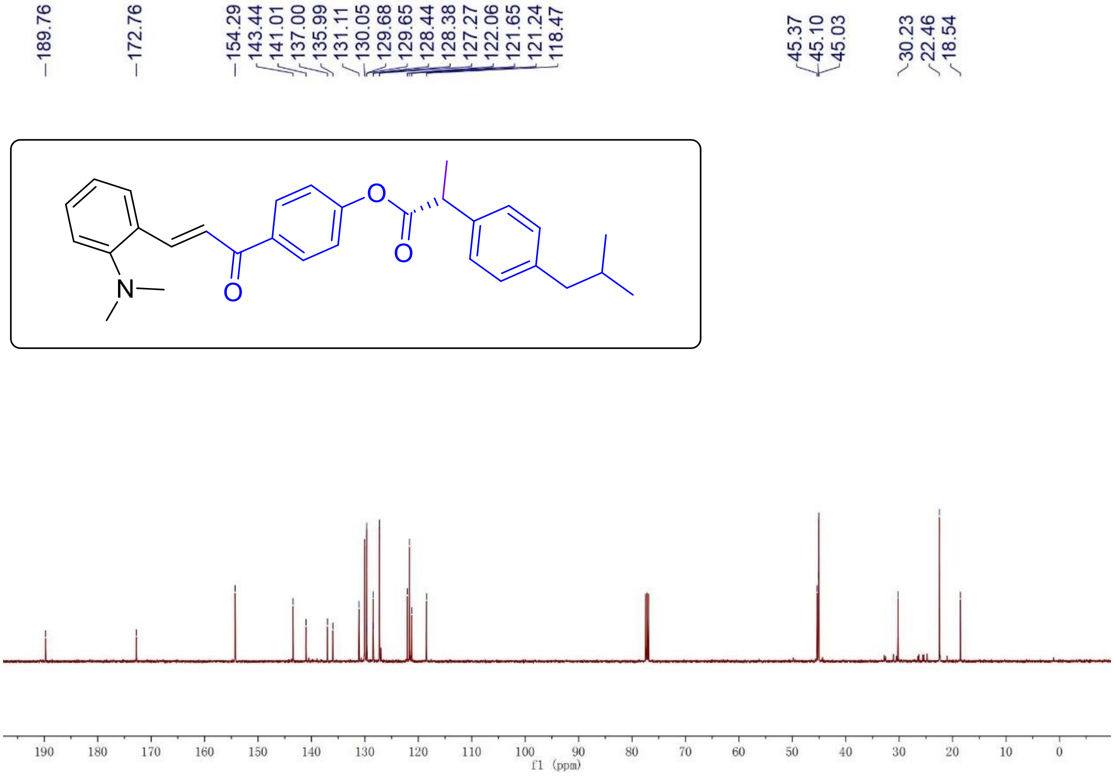


Supplementary Figure 85. $^{1}$H NMR and $^{13}$C NMR spectrum of **10m**.

**(E)-4-(3-(2-(dimethylamino)phenyl)acryloyl)phenyl (R)-2-(6-methoxynaphthalen-2-yl)propanoate (10n)**

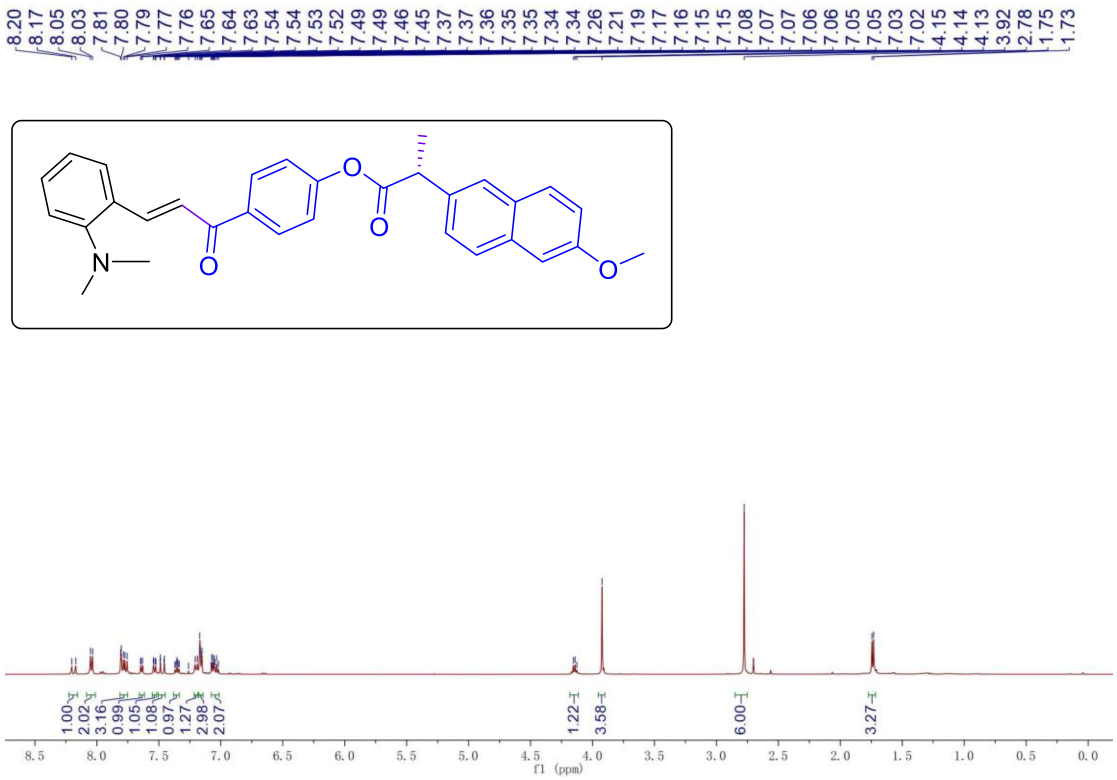

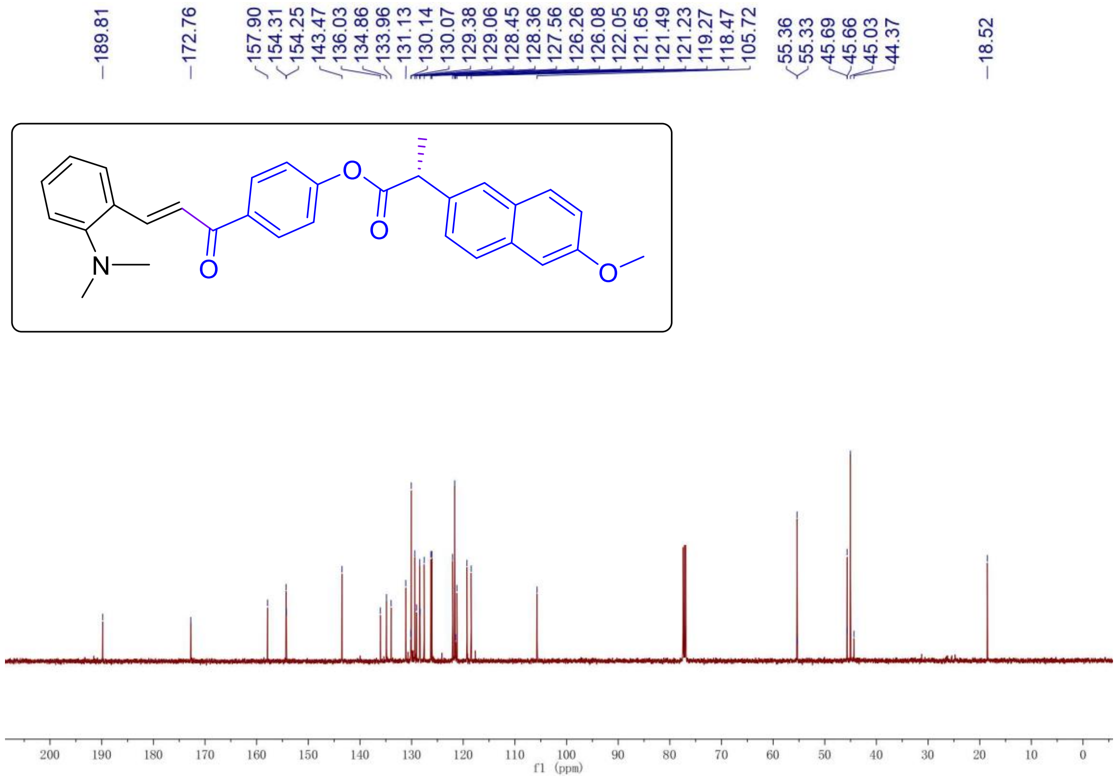

Supplementary Figure 86. $^{1}$H NMR and $^{13}$C NMR spectrum of **10n**.

**(E)-4-(3-(2-(dimethylamino)phenyl)acryloyl)phenyl4-(N,N-dipropylsulfamoyl)benzoate (10o)**

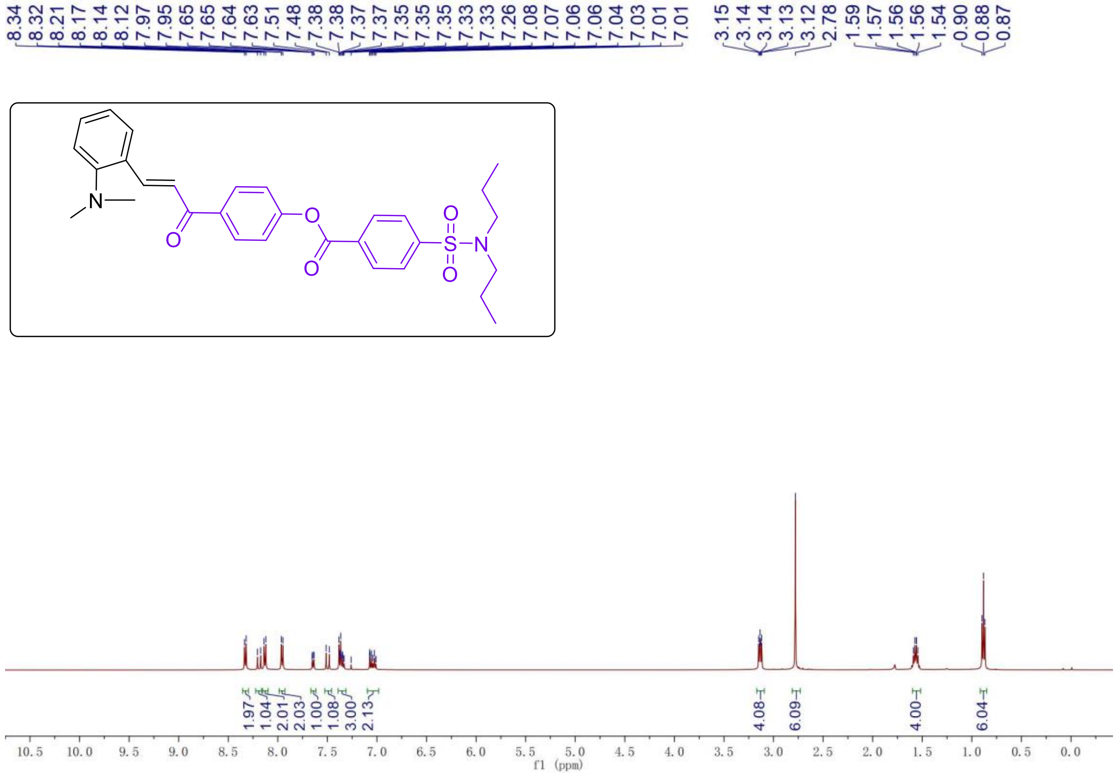

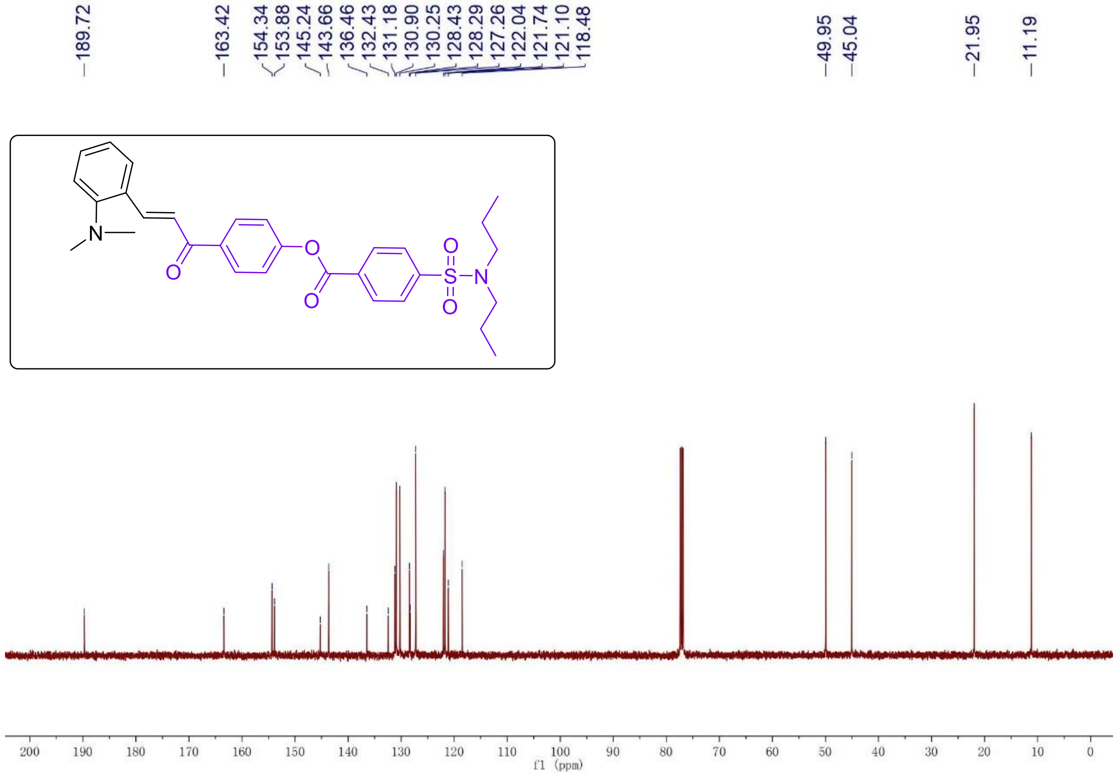

Supplementary Figure 87. $^{1}$H NMR and $^{13}$C NMR spectrum of **10o**.

**(E)-3-(5-chloro-2-(dimethylamino)phenyl)-1-phenylprop-2-en-1-one (12a)**

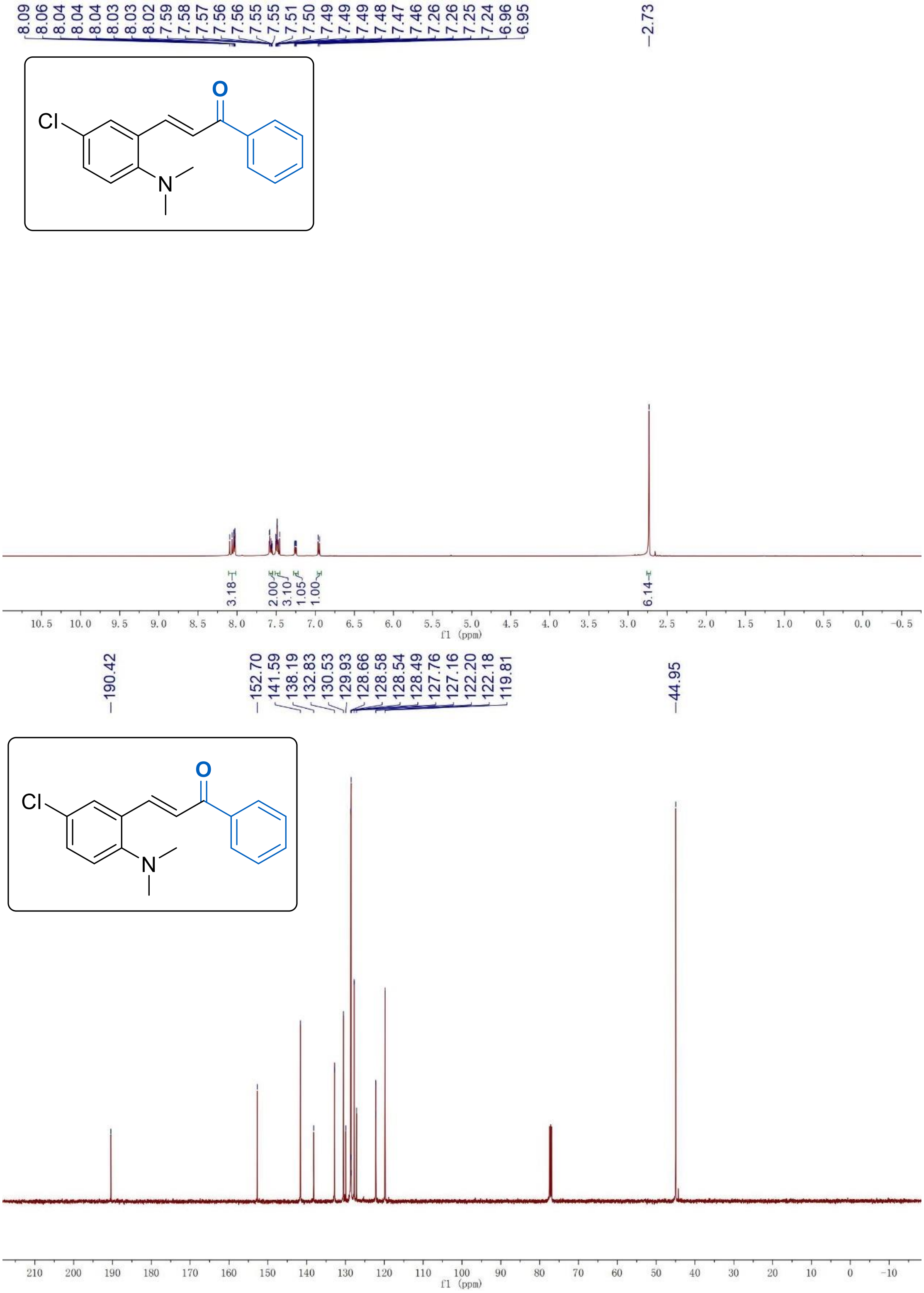


Supplementary Figure 88. $^{1}H$ NMR and $^{13}C$ NMR spectrum of **12a**.

**(E)-3-(5-chloro-2-(dimethylamino)phenyl)-1-(thiophen-2-yl)prop-2-en-1-one (12b)**

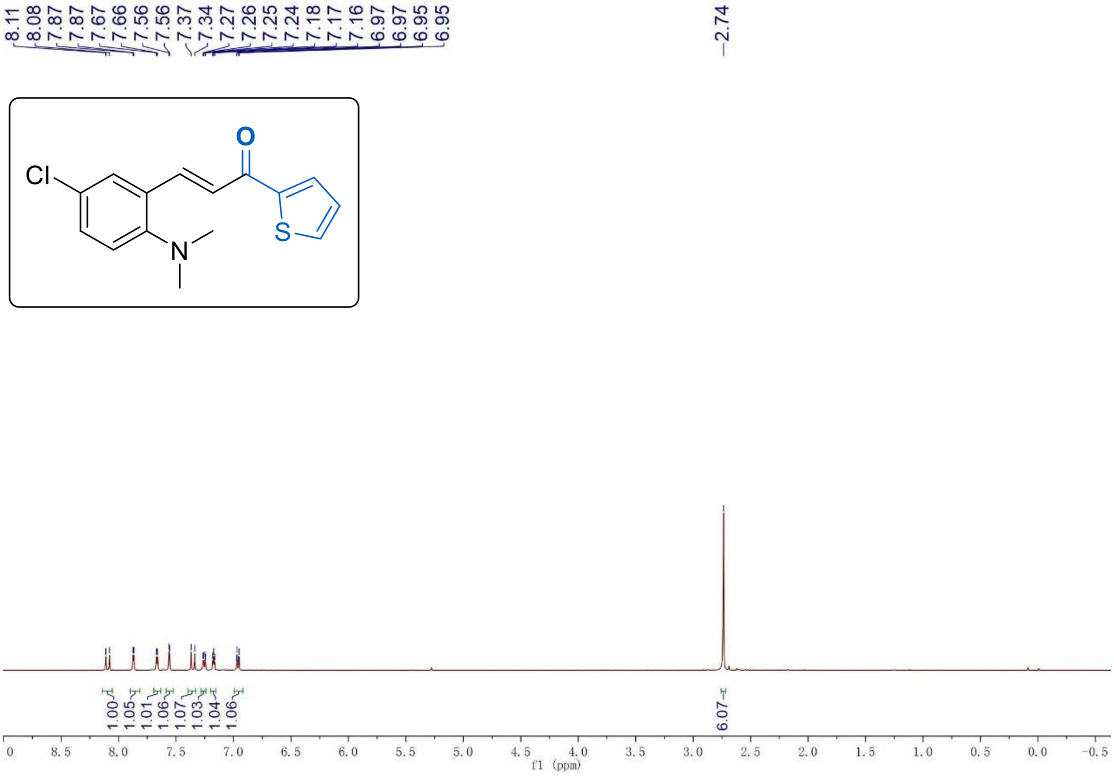


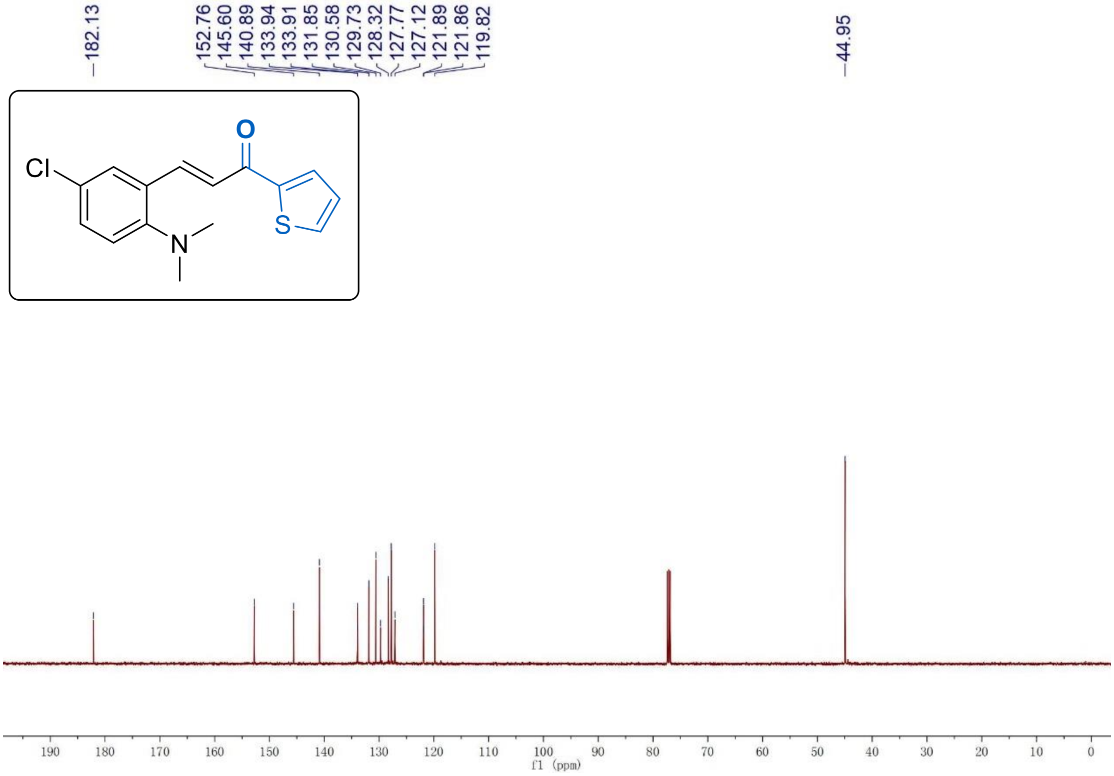


Supplementary Figure 89. $^{1}$H NMR and $^{13}$C NMR spectrum of **12b**.

## (E)-3-(5-chloro-2-(dimethylamino)phenyl)-1-cyclopropylprop-2-en-1-one (12c)

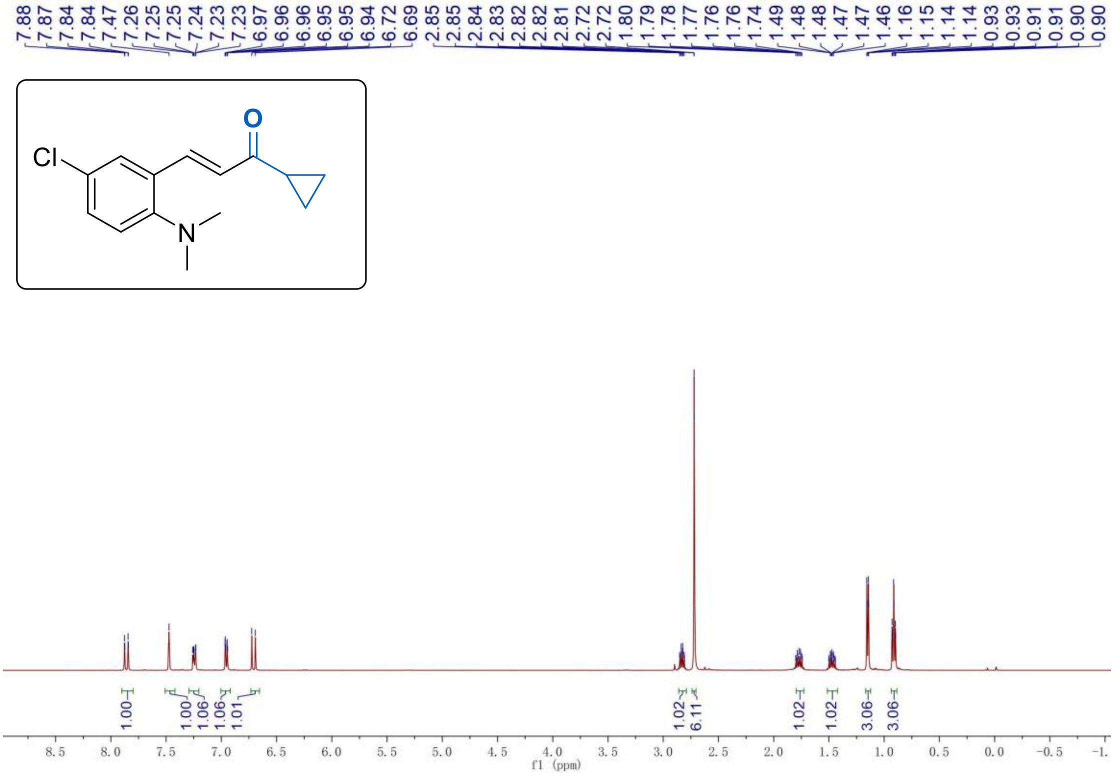


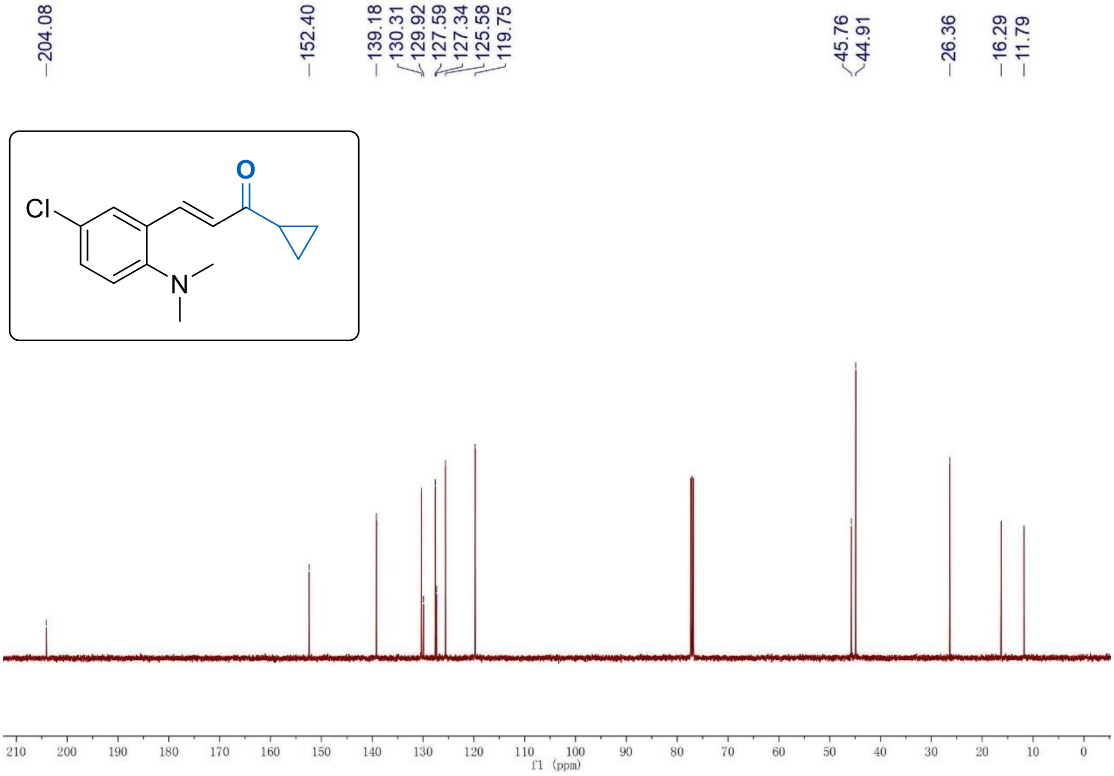


Supplementary Figure 90. $^{1}$H NMR and $^{13}$C NMR spectrum of **12c**.

**(E)-1-(5-chloro-2-(dimethylamino)phenyl)-4-methylhex-1-en-3-one (12d)**

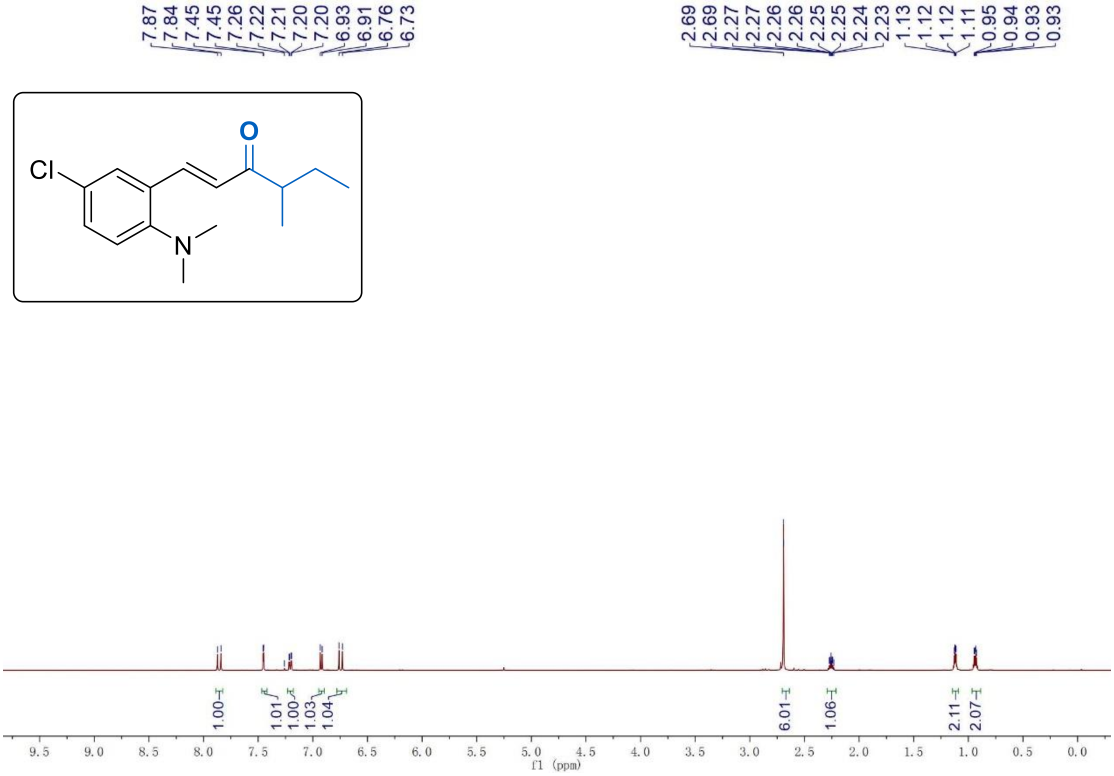

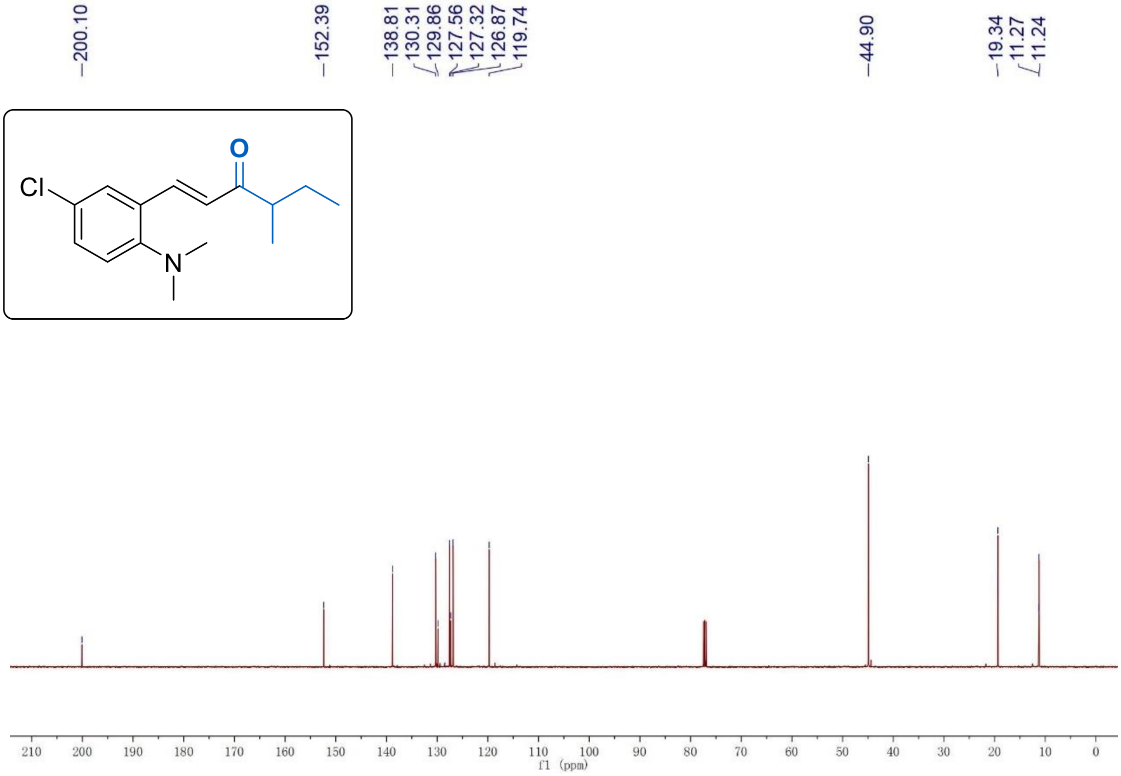

Supplementary Figure 91. $^{1}$H NMR and $^{13}$C NMR spectrum of **12d**.

**(E)-3-(5-chloro-2-(dimethylamino)phenyl)-1-phenylprop-Acetylferrocene (12e)**

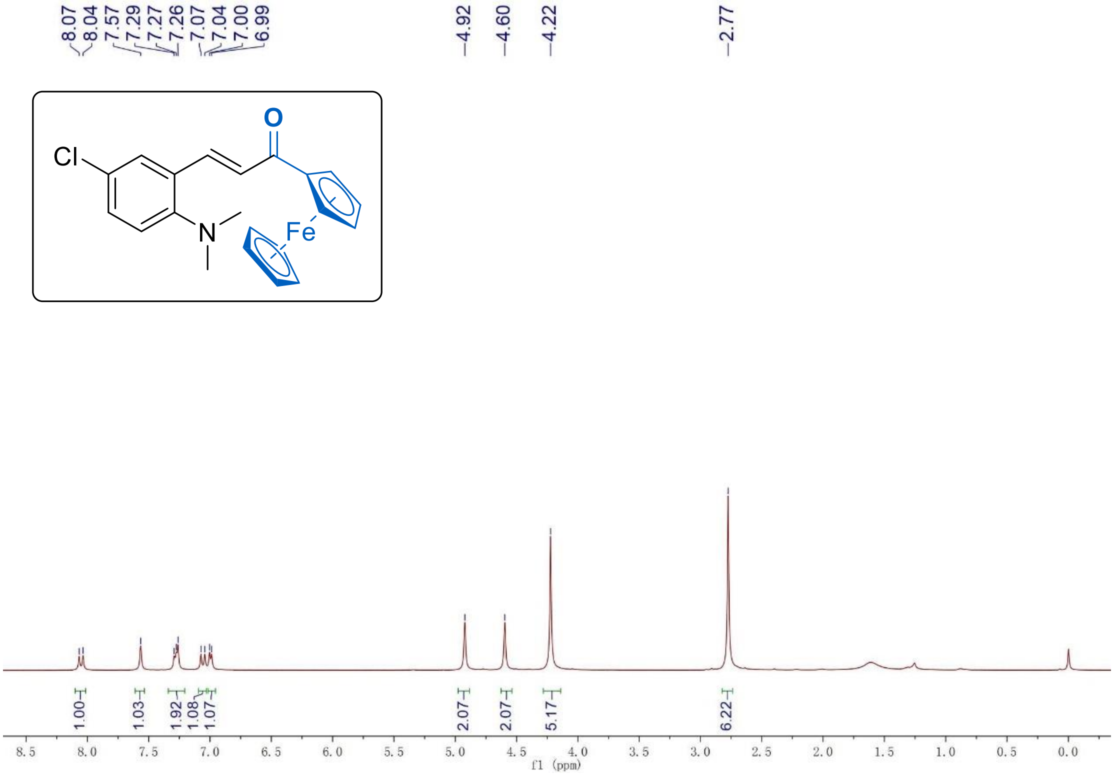


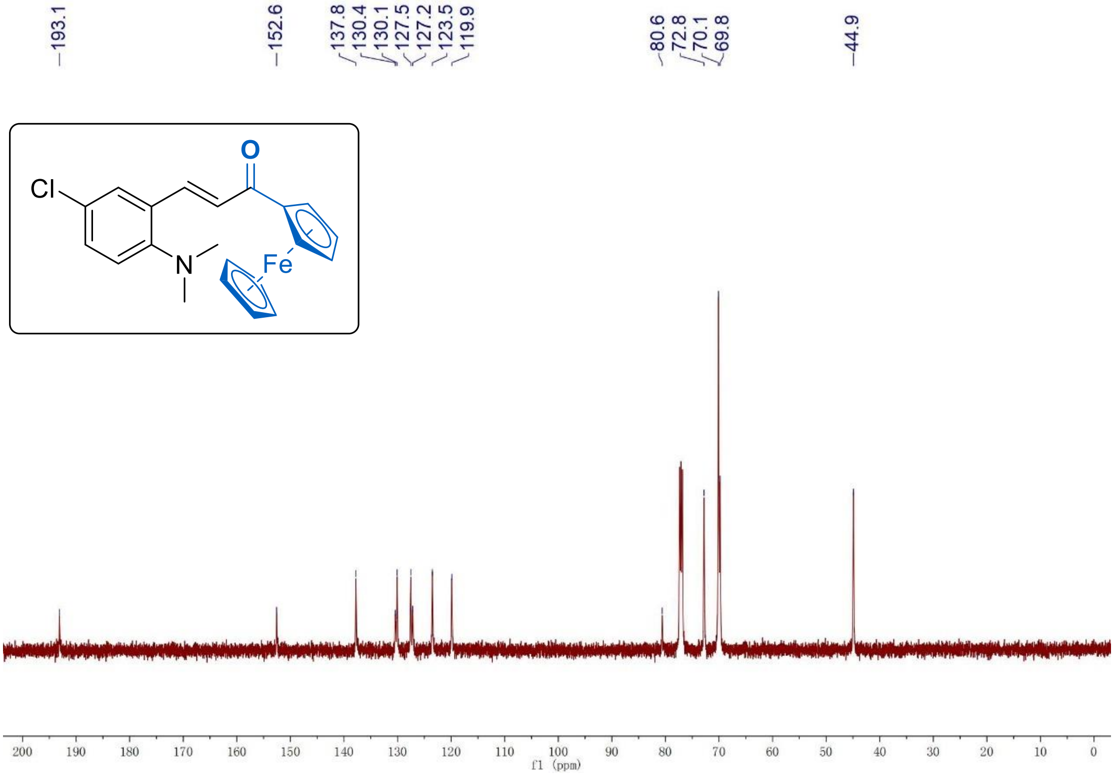


Supplementary Figure 92. $^{1}$H NMR and $^{13}$C NMR spectrum of **12e**.

**(E)-3-(5-bromo-2-(ethyl(methyl)amino)phenyl)-1-phenylprop-2-en-1-one (15)**

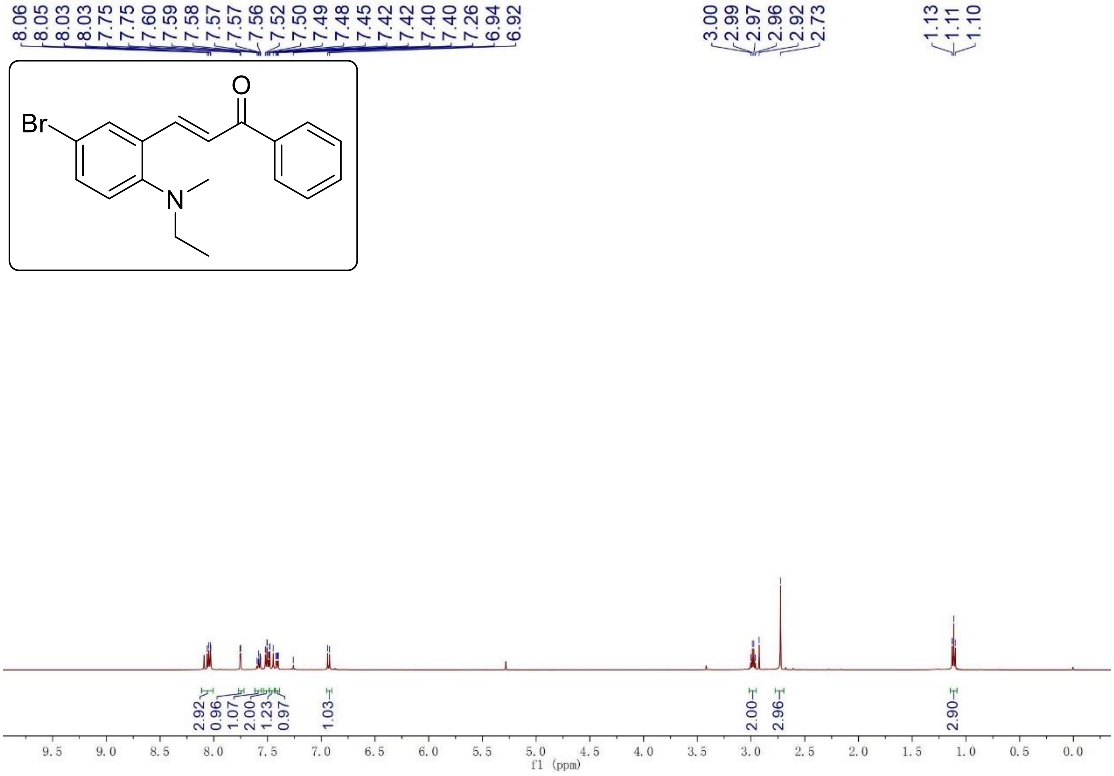

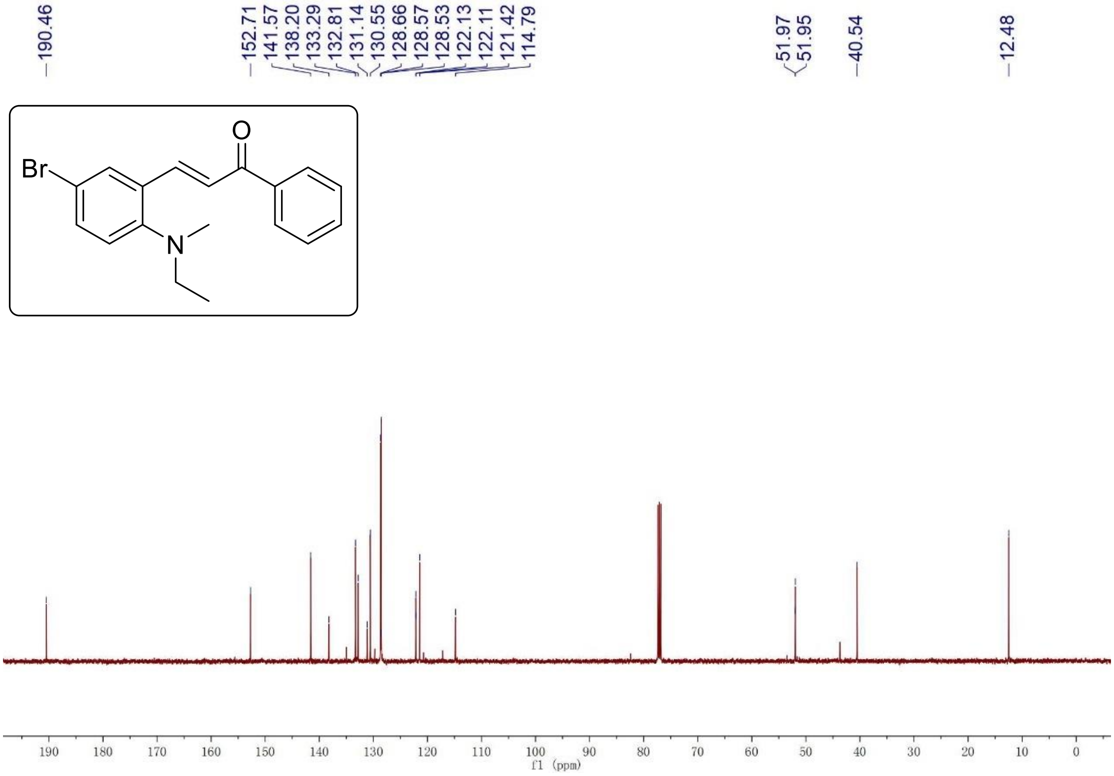

Supplementary Figure 93. $^{1}H$ NMR and $^{13}C$ NMR spectrum of **15**.

**2-(2-fluoro-1-methyl-1H-indol-3-yl)-1-phenylethan-1-one (3a)**

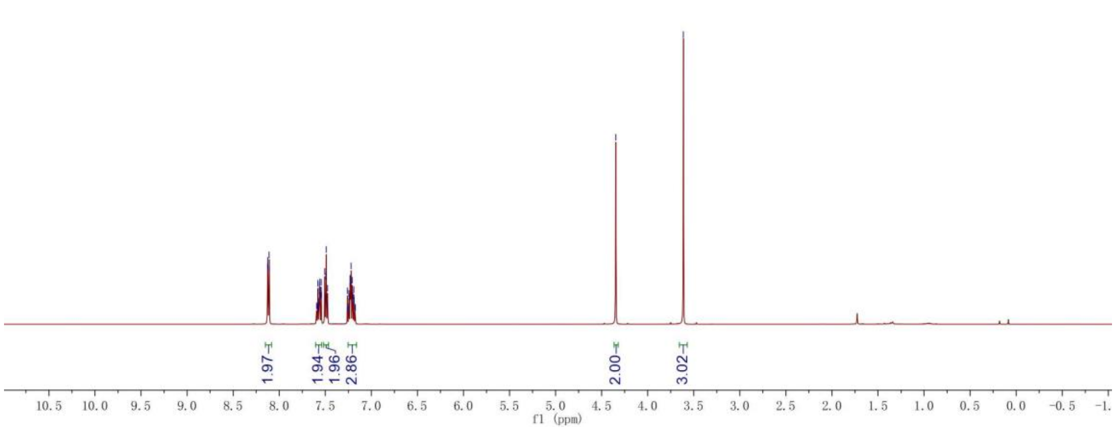


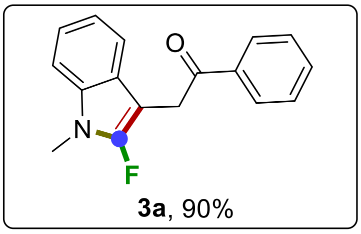


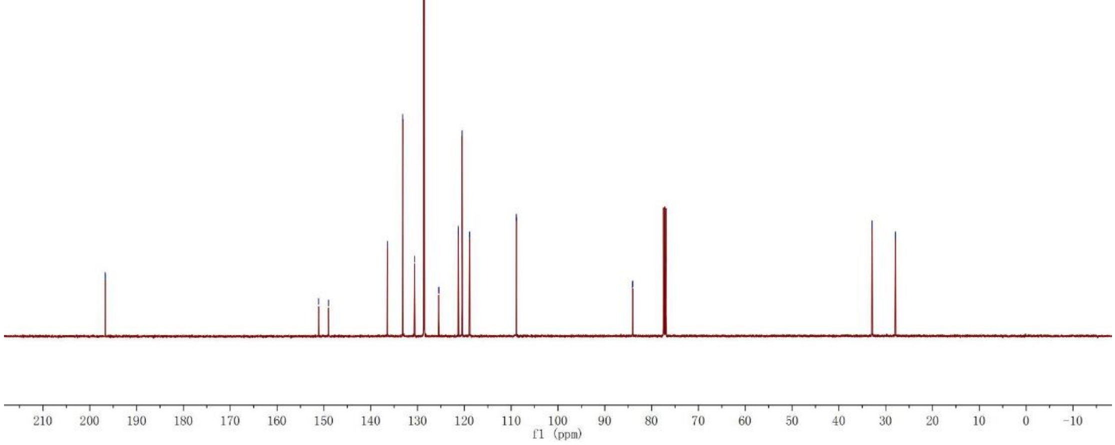


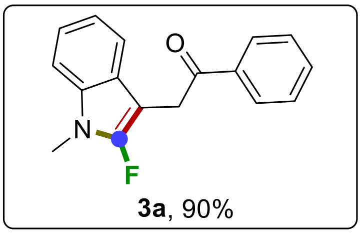

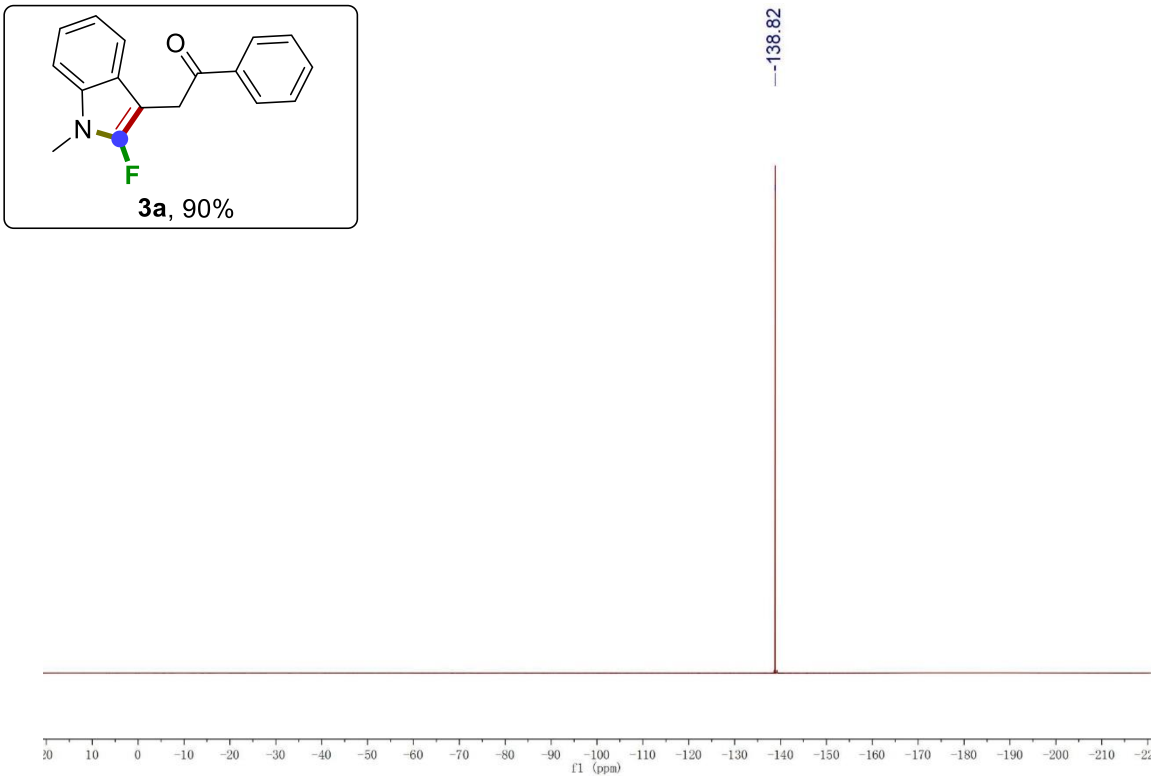


Supplementary Figure 94. $^{1}H$ NMR, $^{13}C$ NMR and $^{19}F$ NMR spectrum of **3a**.

## 1-([1,1'-biphenyl]-4-yl)-2-(2-fluoro-1-methyl-1H-indol-3-yl)ethan-1-one (3b)

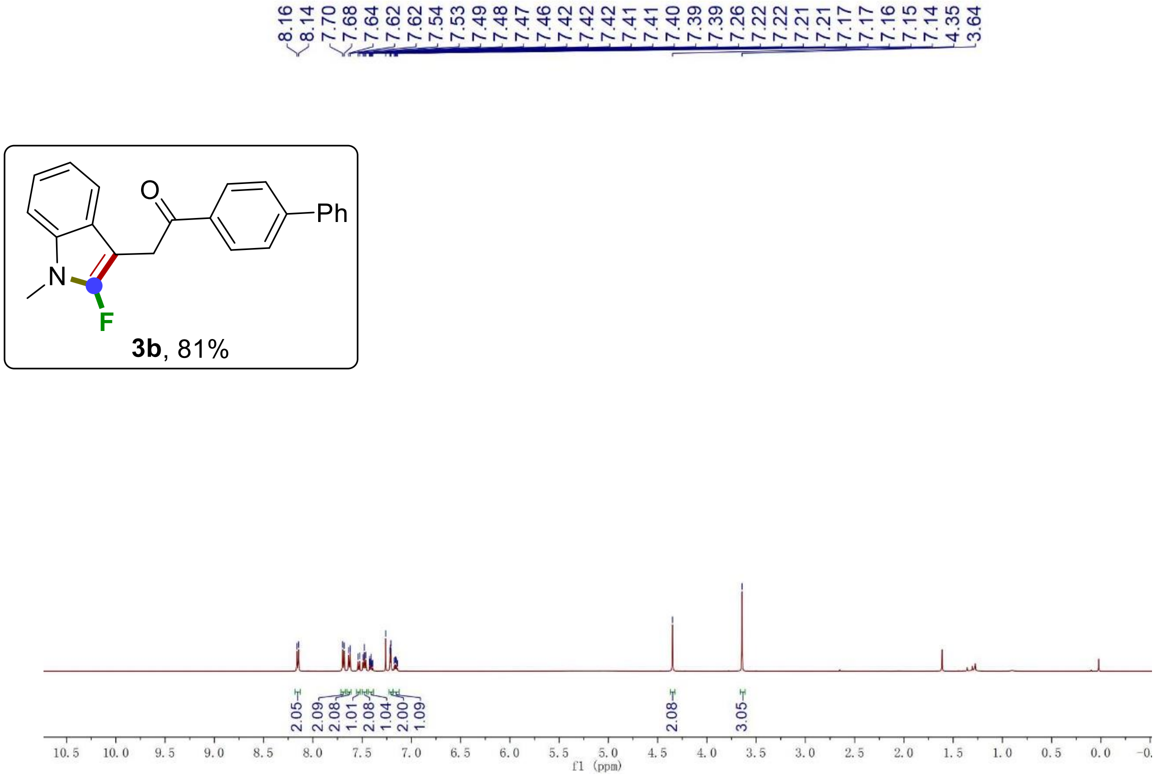


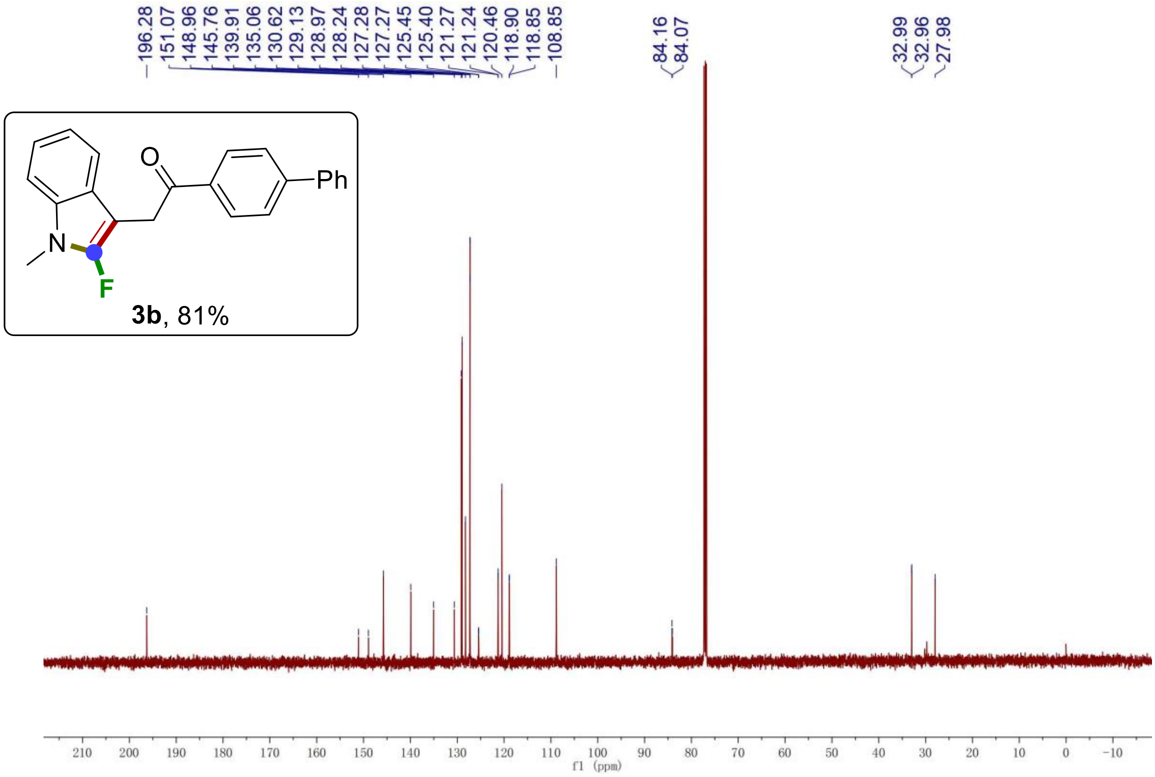

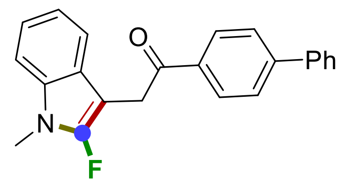


**3b**, 81%

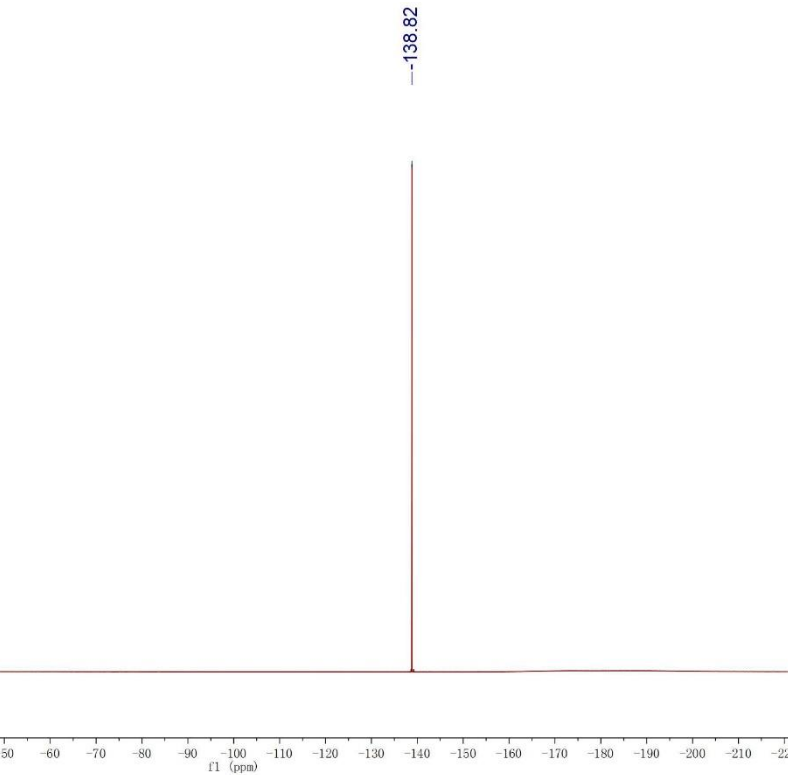


Supplementary Figure 95. $^{1}$H NMR, $^{13}$C NMR and $^{19}$F NMR spectrum of **3b**.

**ethyl 4-(2-(2-fluoro-1-methyl-1H-indol-3-yl)acetyl)benzoate (3c)**

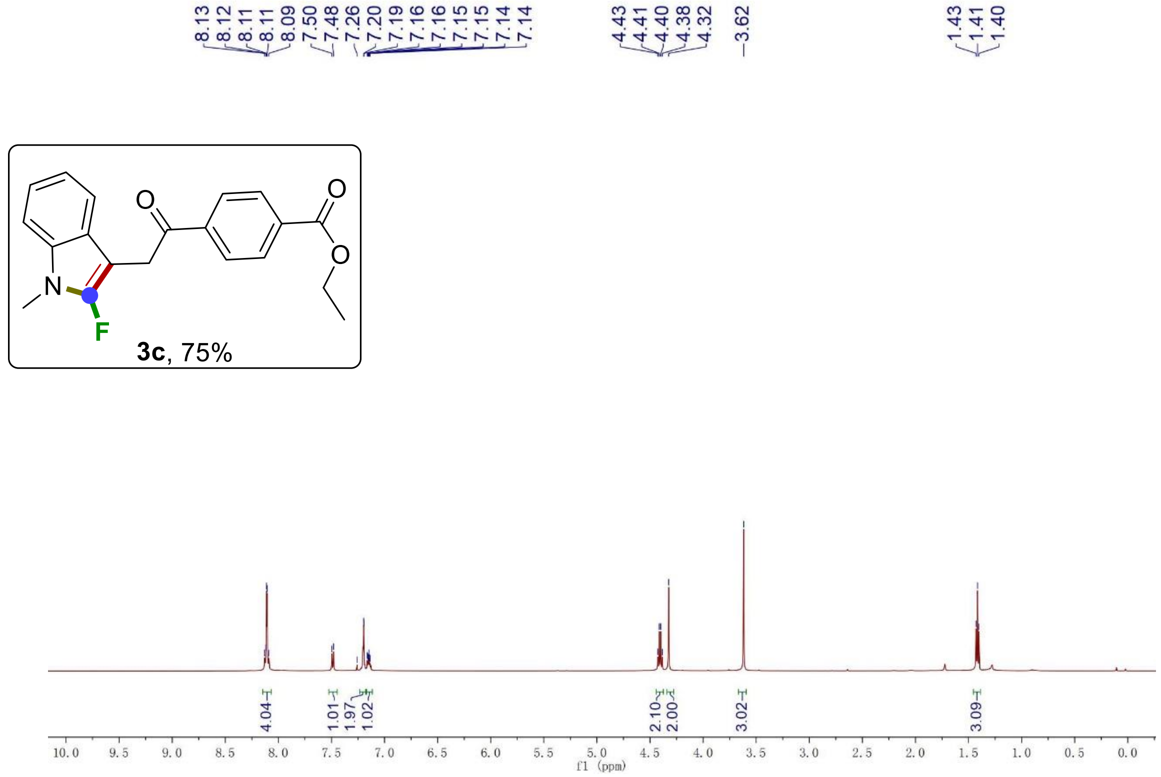


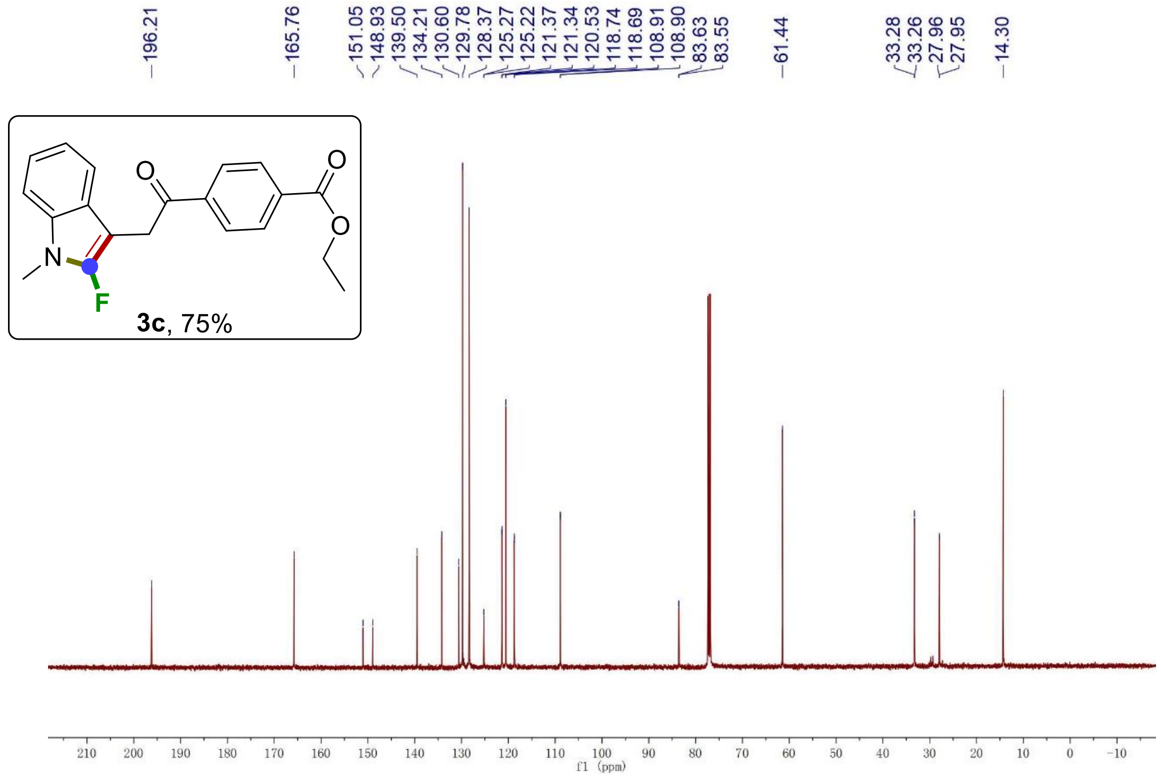

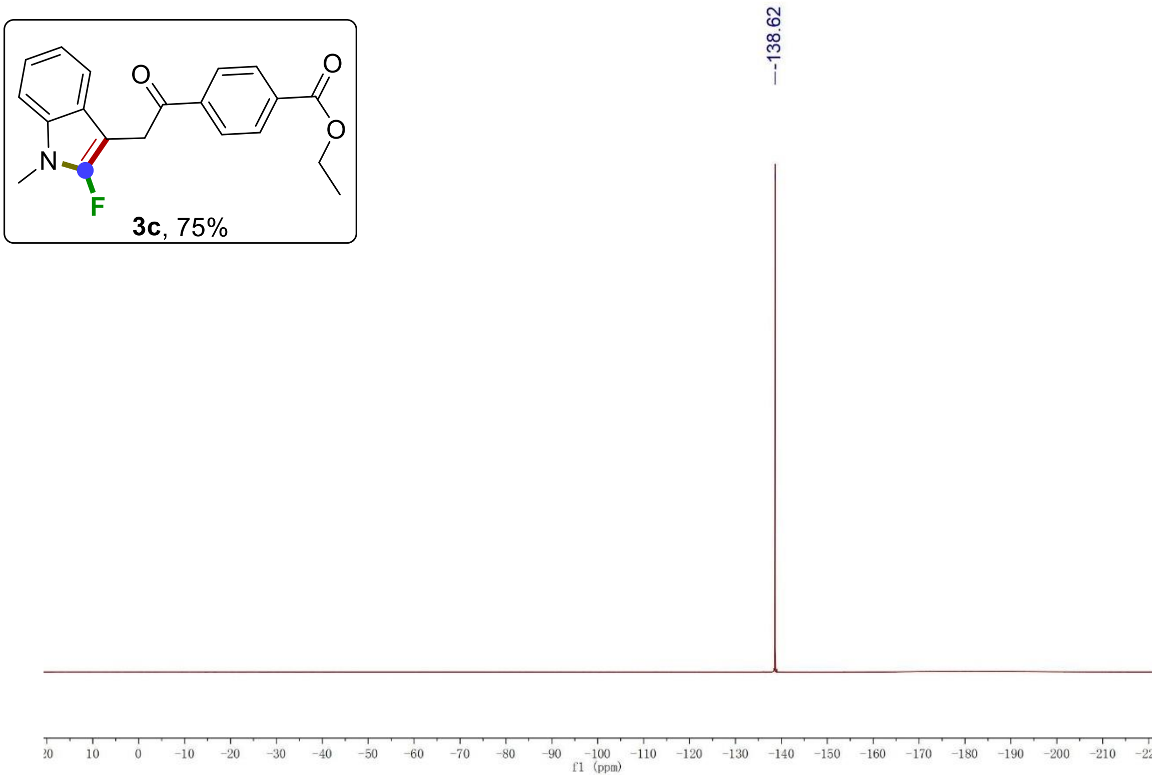


Supplementary Figure 95. $^{1}$H NMR, $^{13}$C NMR and $^{19}$F NMR spectrum of **3c**.

**4-(2-(2-fluoro-1-methyl-1H-indol-3-yl)acetyl)benzonitrile (3d)**

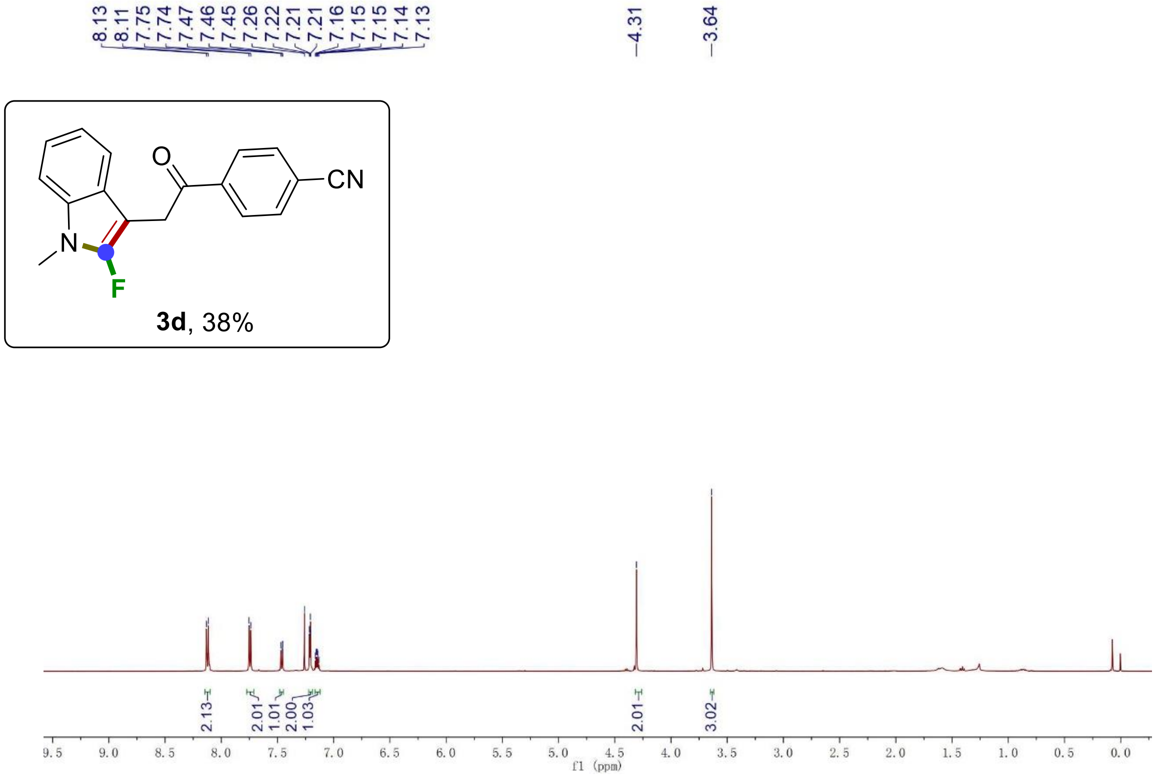


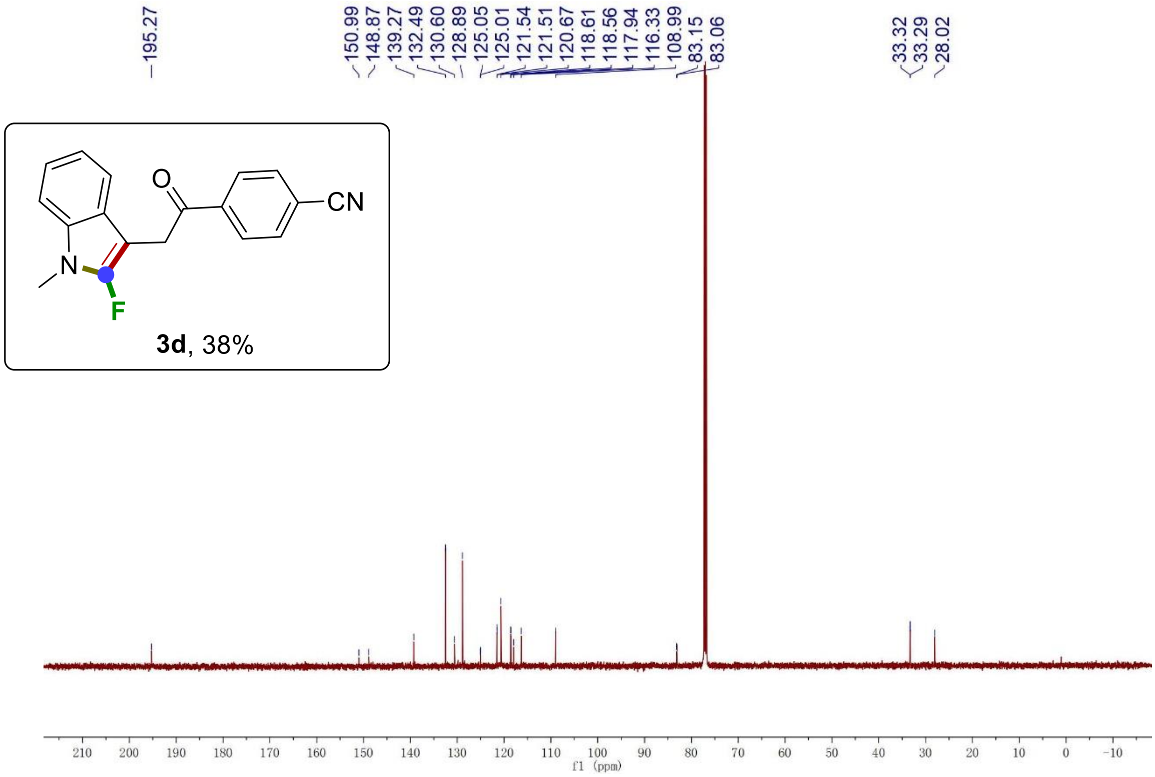

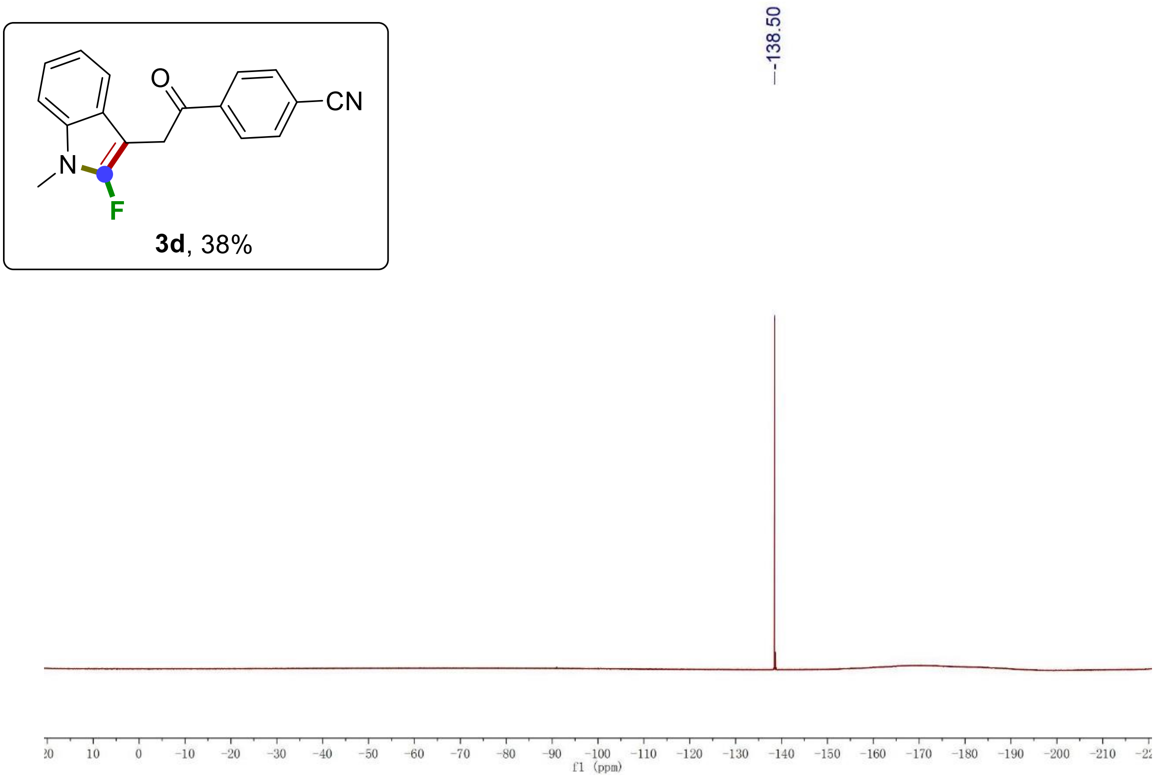


Supplementary Figure 96. $^{1}H$ NMR, $^{13}C$ NMR and $^{19}F$ NMR spectrum of **3d**.

## 2-(2-fluoro-1-methyl-1H-indol-3-yl)-1-(4-(methylthio)phenyl)ethan-1-one (3e)

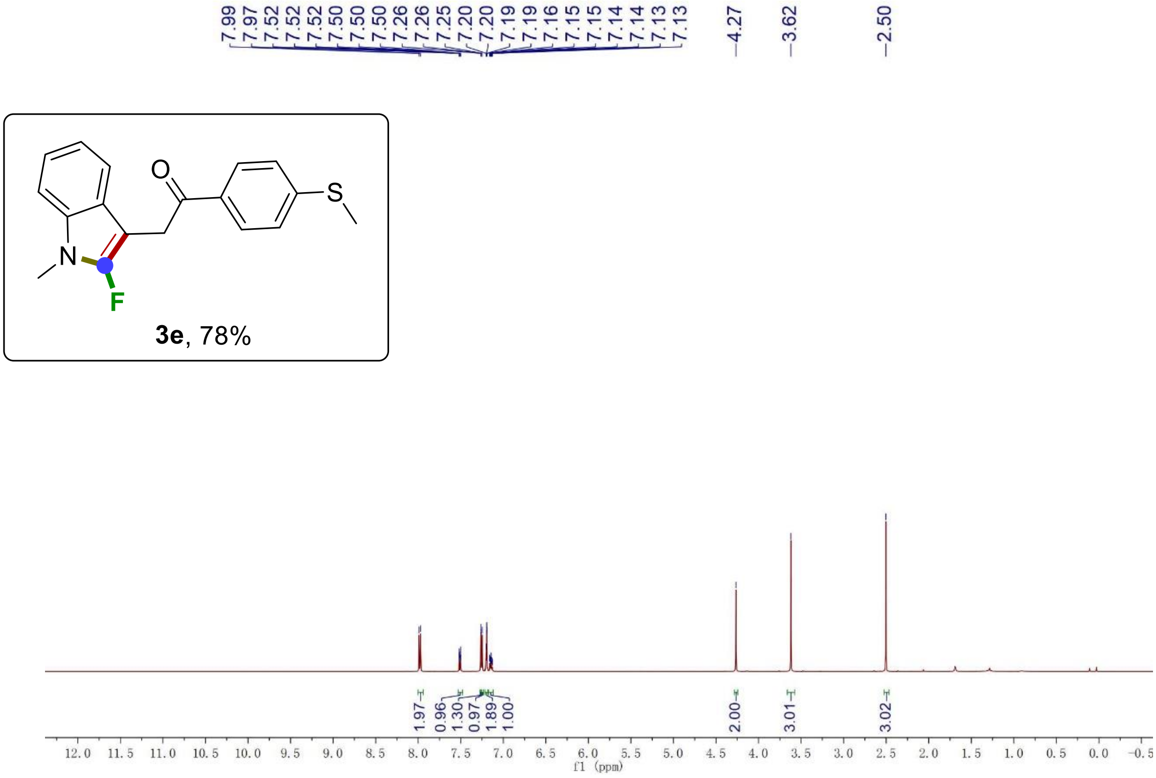


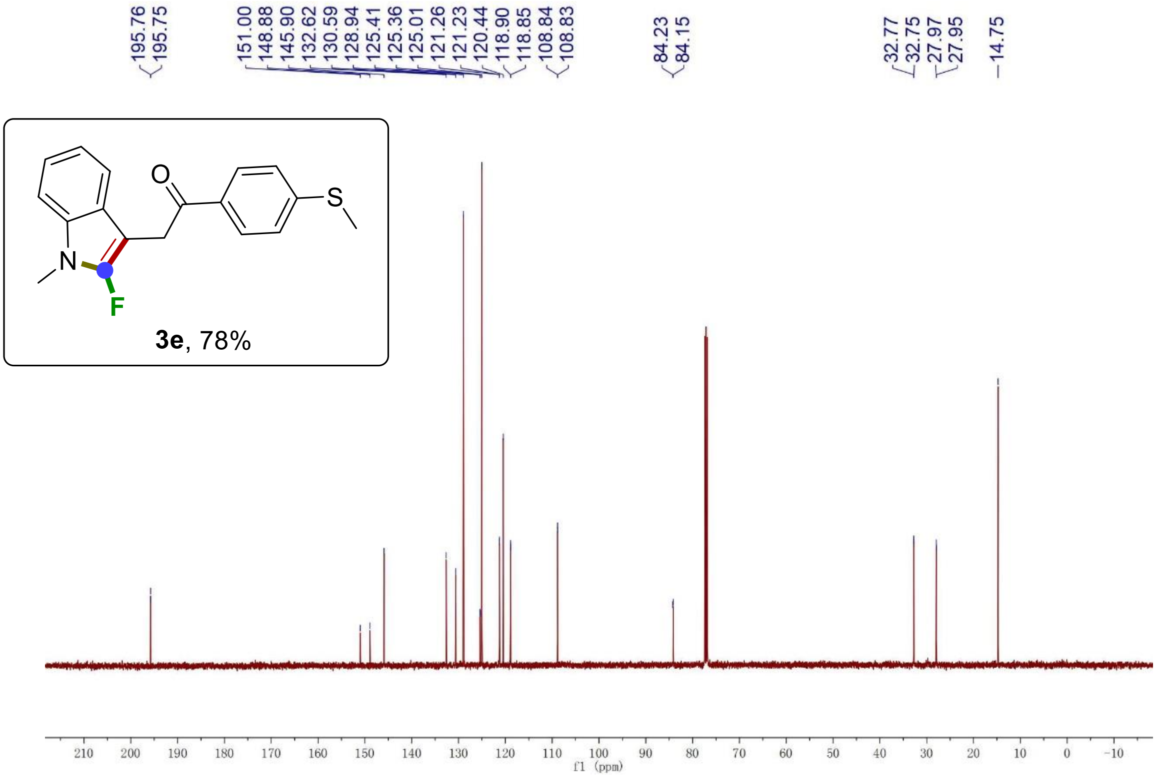

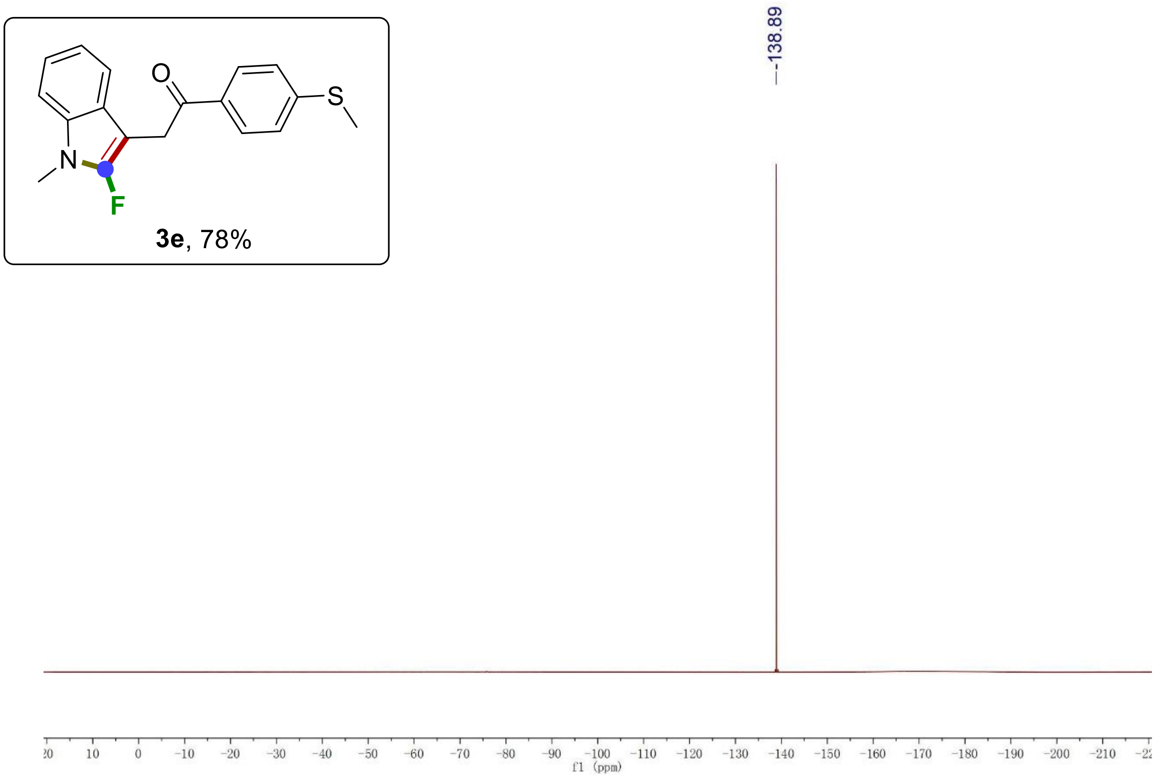


Supplementary Figure 97. $^{1}H$ NMR, $^{13}C$ NMR and $^{19}F$ NMR spectrum of **3e**.

## 1-(4-(diethylamino)phenyl)-2-(2-fluoro-1-methyl-1H-indol-3-yl)ethan-1-one (3f)

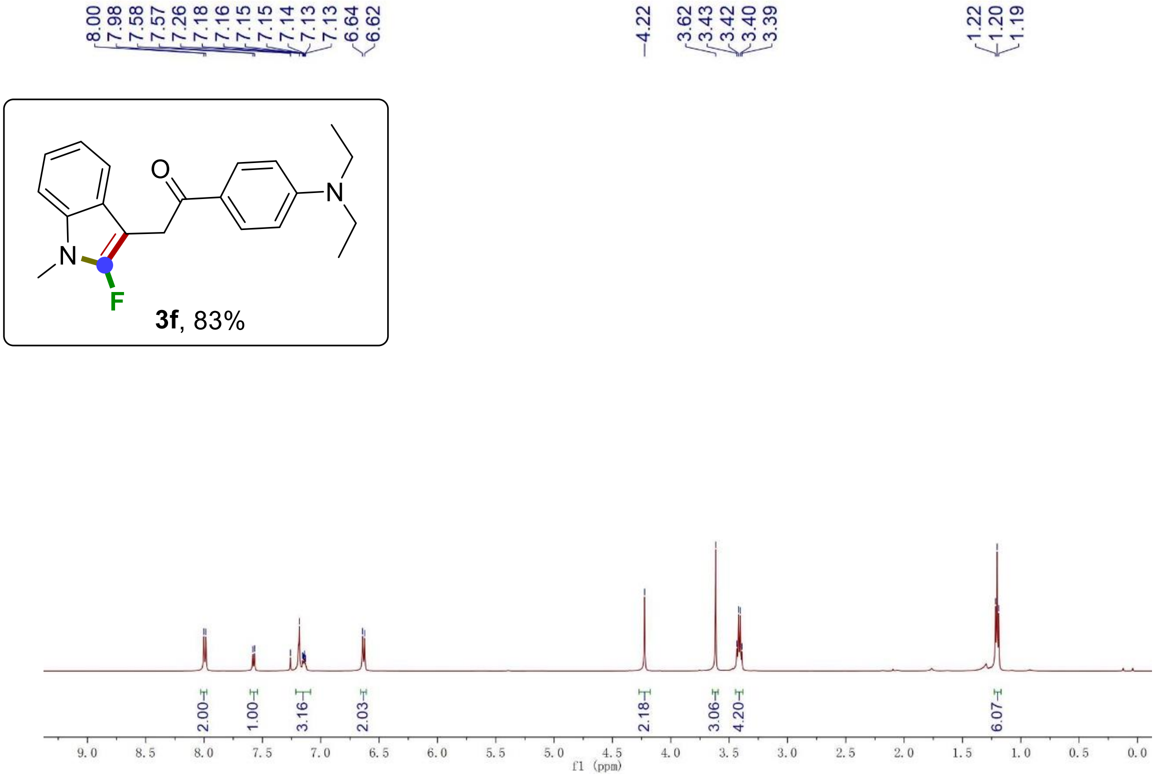


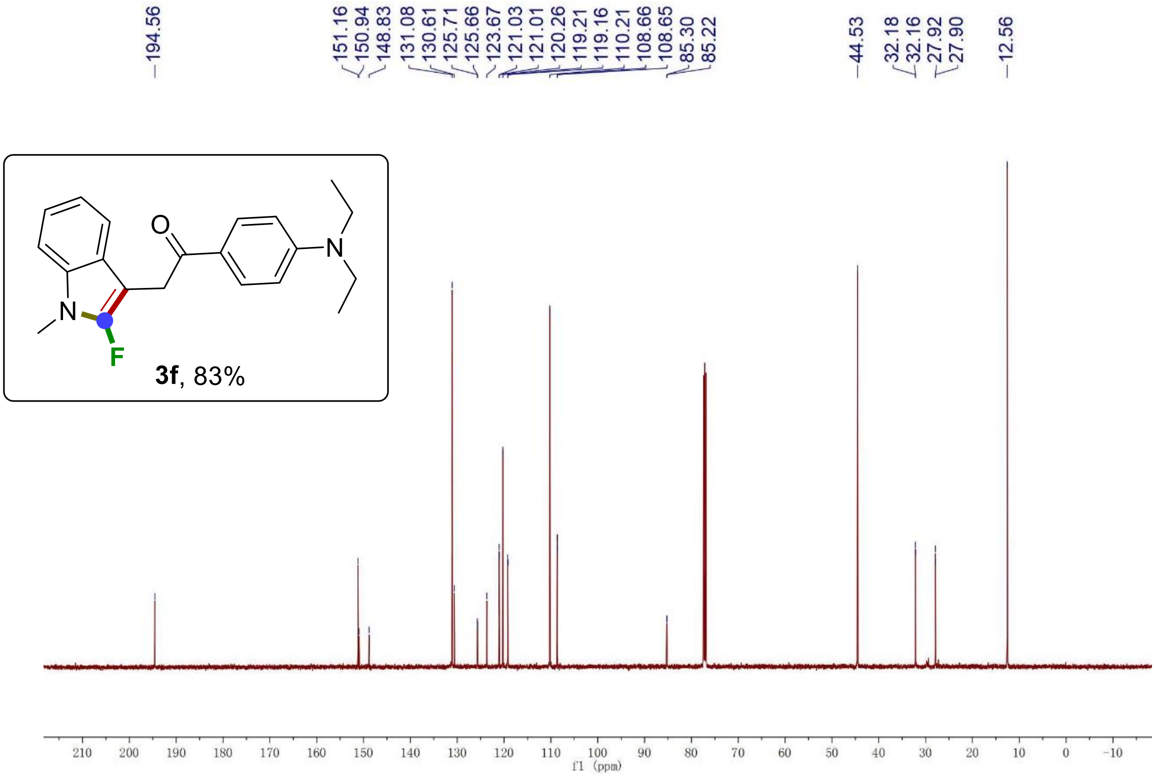

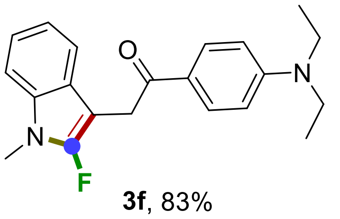

**3f**, 83%

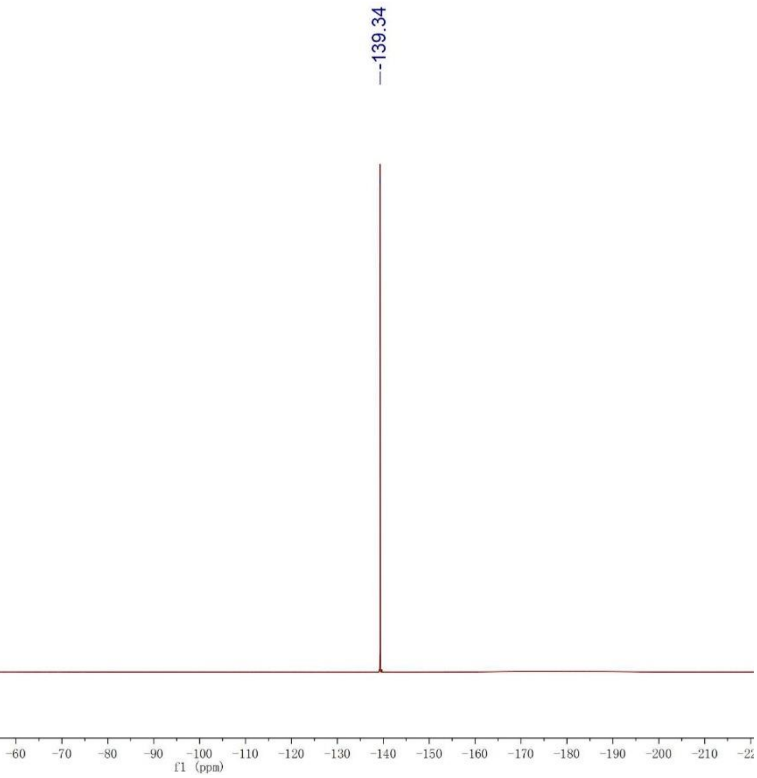


Supplementary Figure 98. $^{1}$H NMR, $^{13}$C NMR and $^{19}$F NMR spectrum of **3f**.

**2-(2-fluoro-1-methyl-1H-indol-3-yl)-1-(4-methoxyphenyl)ethan-1-one (3g)**

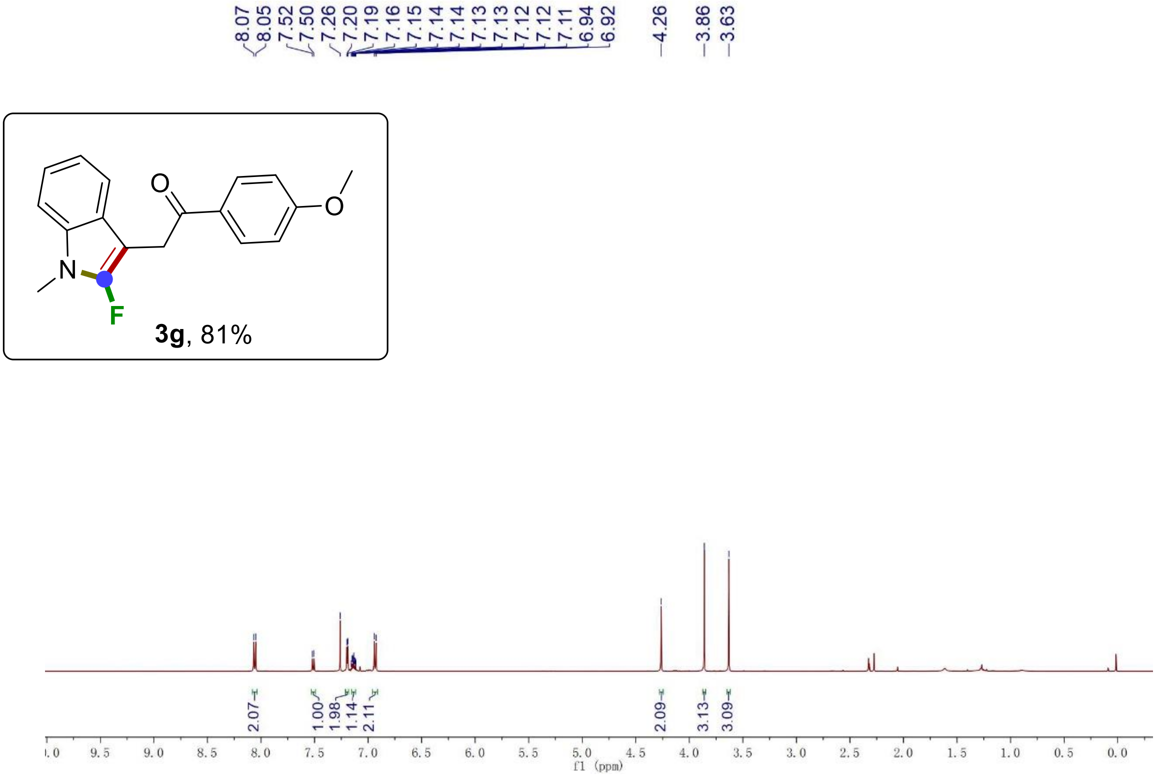


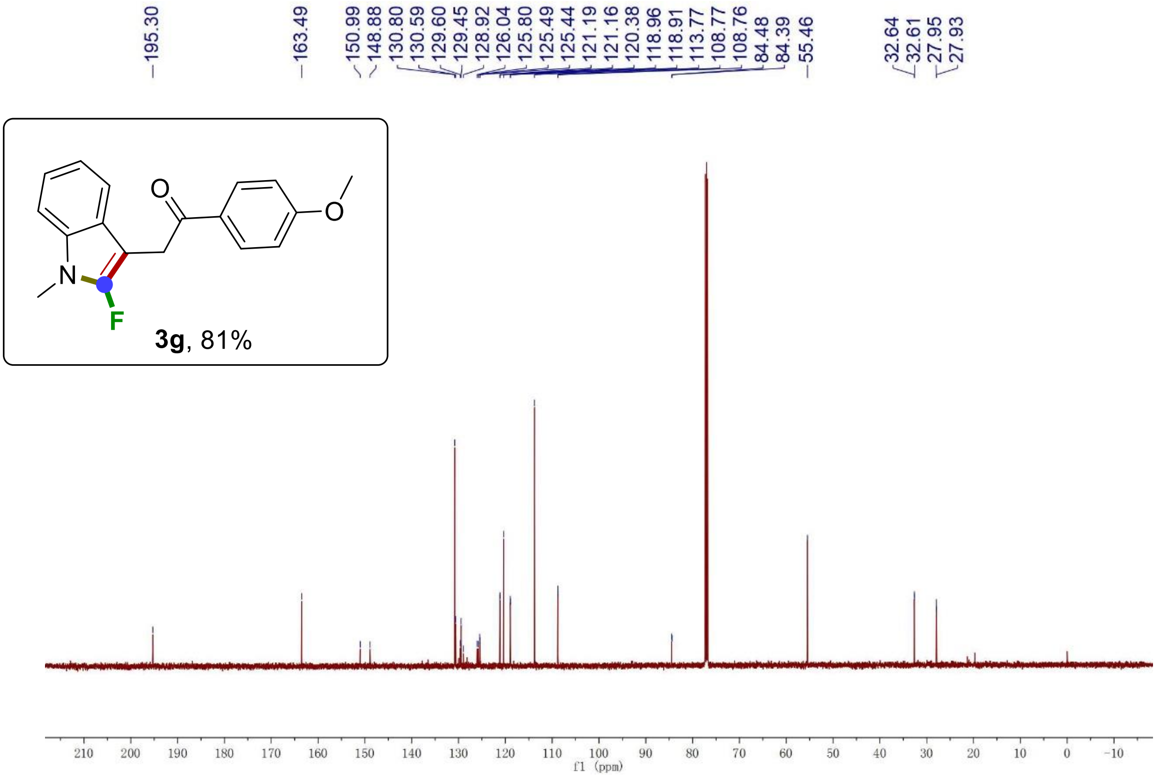

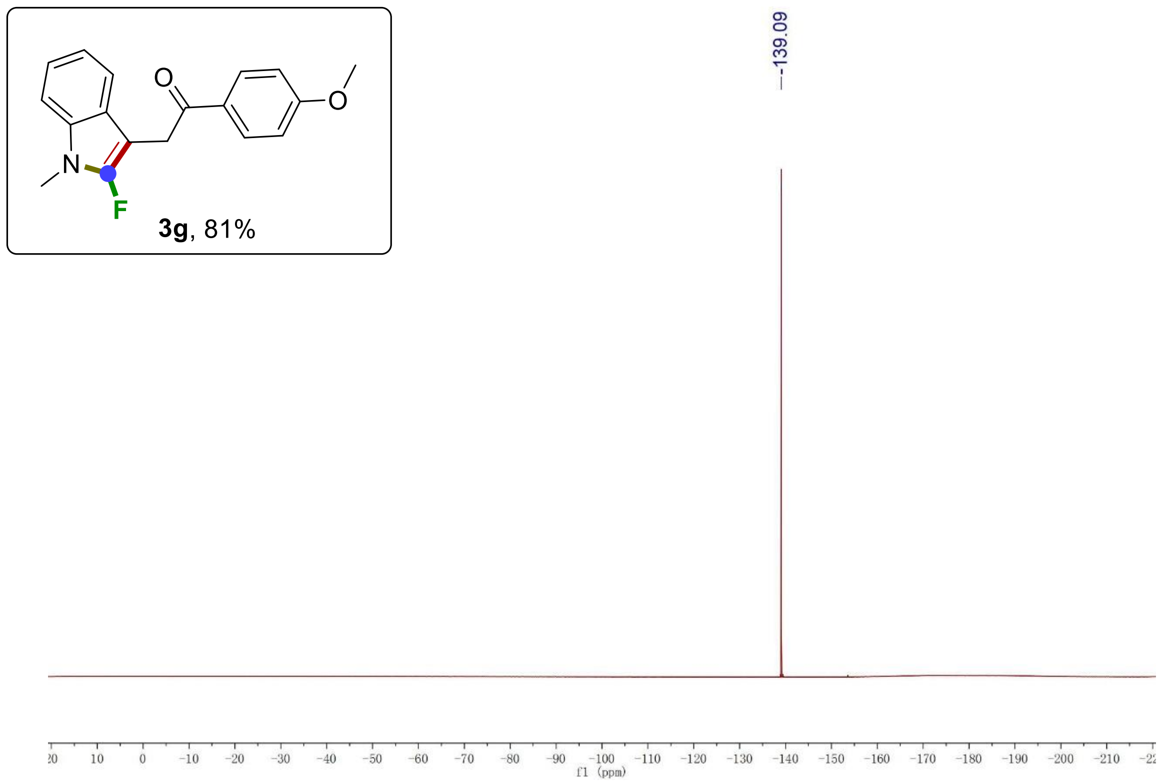


Supplementary Figure 99. $^{1}H$ NMR, $^{13}C$ NMR and $^{19}F$ NMR spectrum of **3g**.

## 1-(3,4-dimethylphenyl)-2-(2-fluoro-1-methyl-1H-indol-3-yl)ethan-1-one (3h)

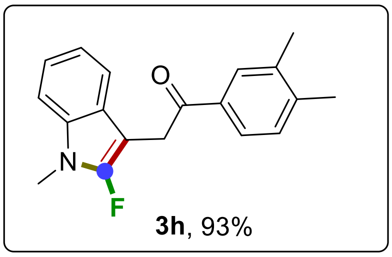


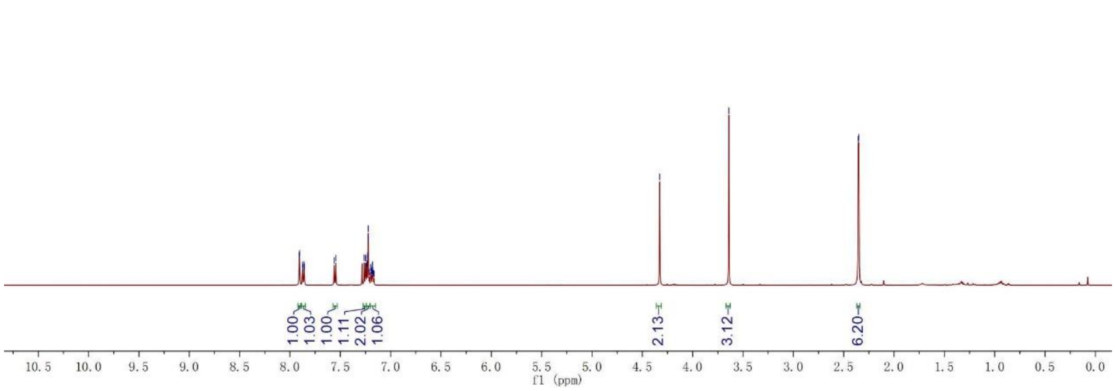


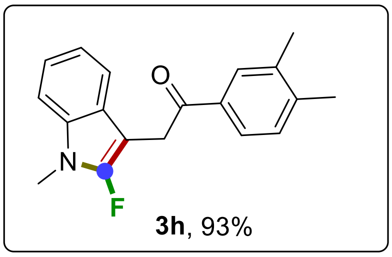


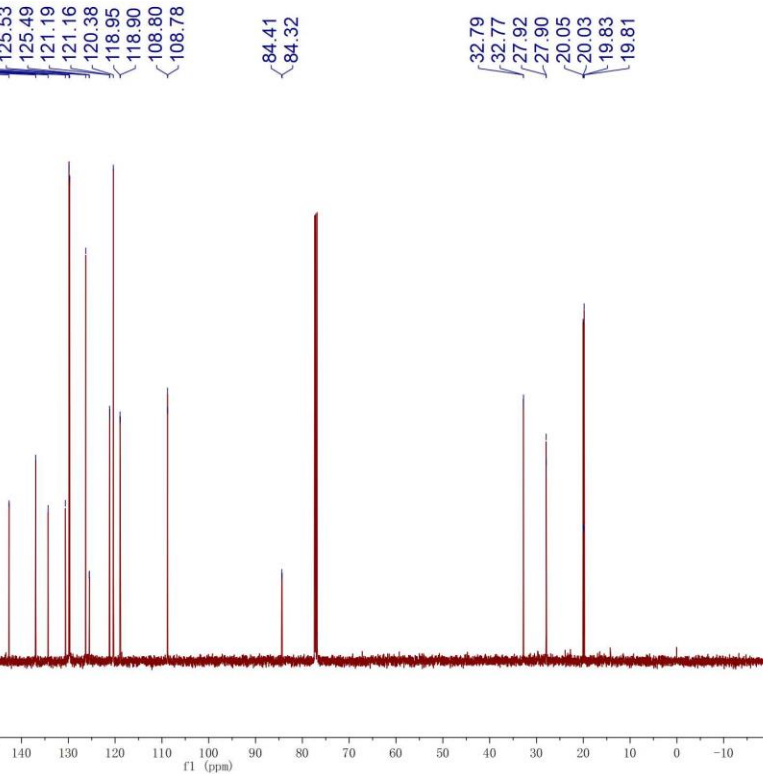

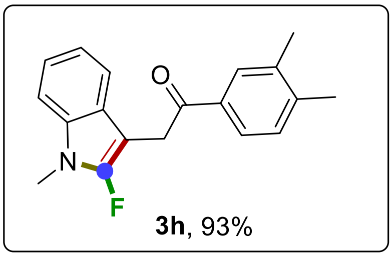

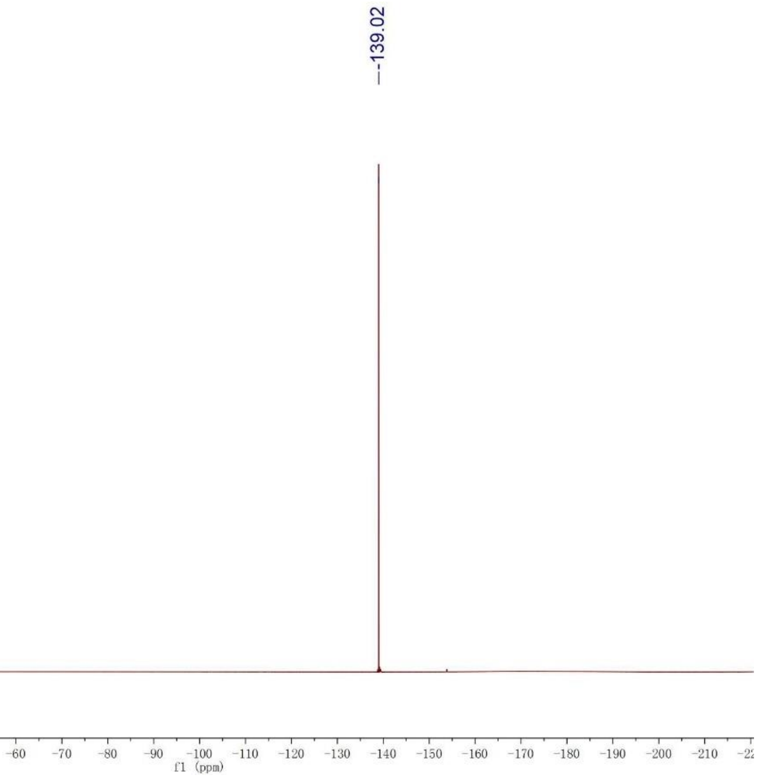


Supplementary Figure 100. $^{1}$H NMR, $^{13}$C NMR and $^{19}$F NMR spectrum of **3h**.

## 2-(2-fluoro-1-methyl-1H-indol-3-yl)-1-(m-tolyl)ethan-1-one (3i)

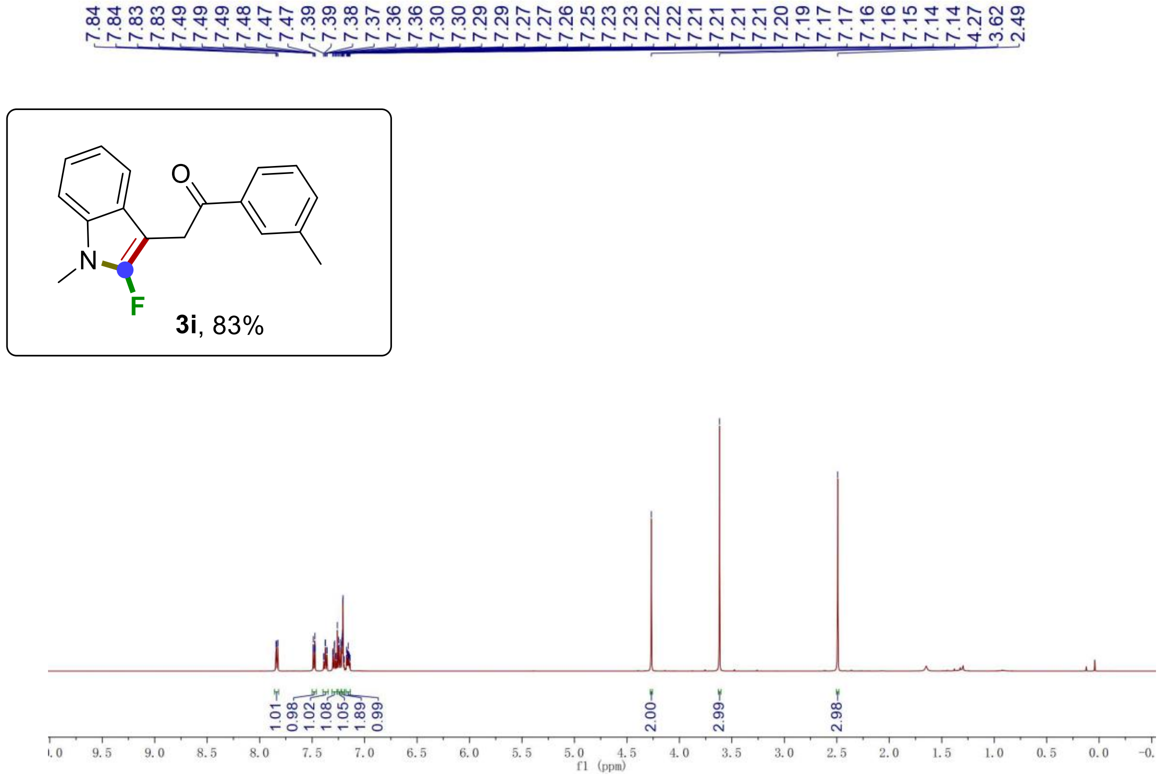


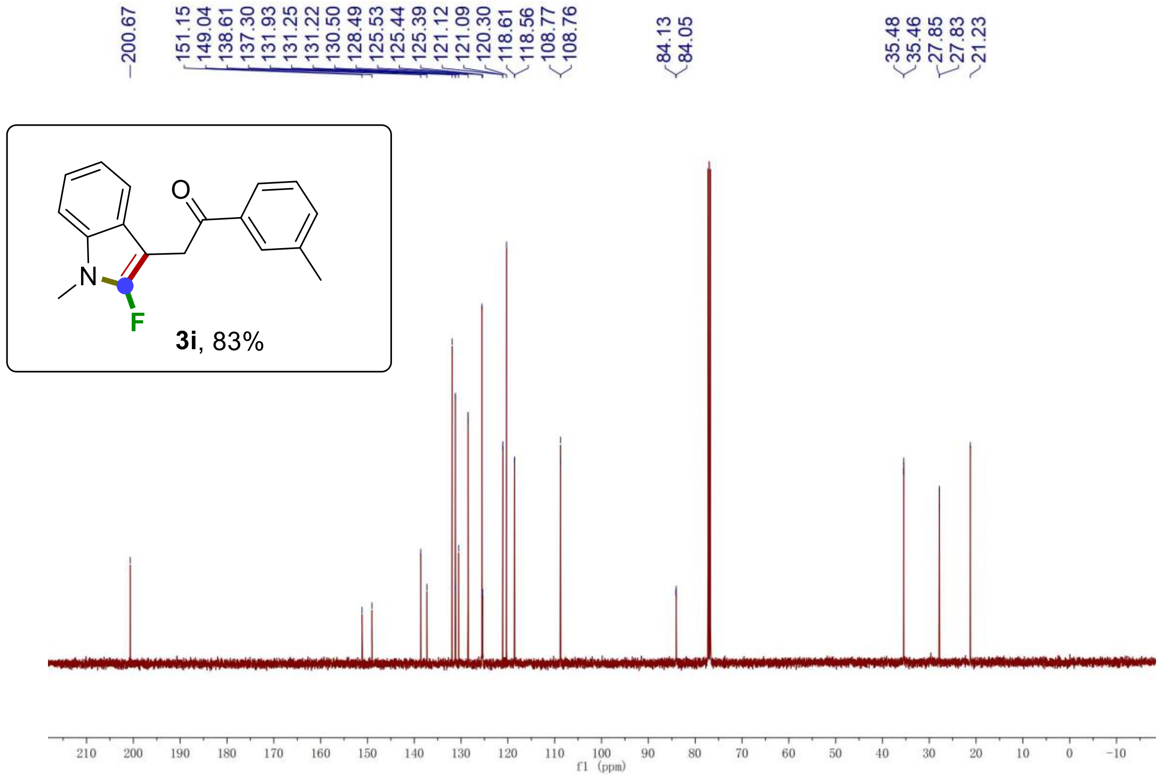

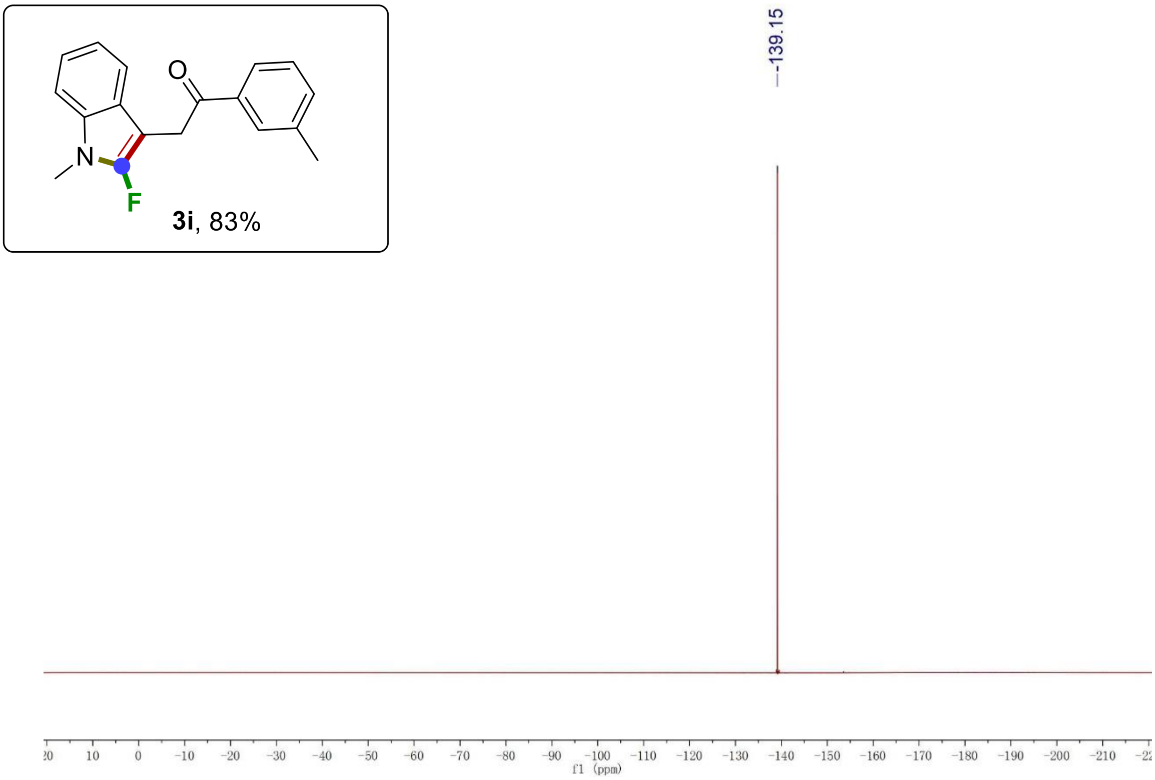


Supplementary Figure 101. $^{1}H$ NMR, $^{13}C$ NMR and $^{19}F$ NMR spectrum of **3i**.

**2-(2-fluoro-1-methyl-1H-indol-3-yl)-1-(4-iodophenyl)ethan-1-one (3j)**

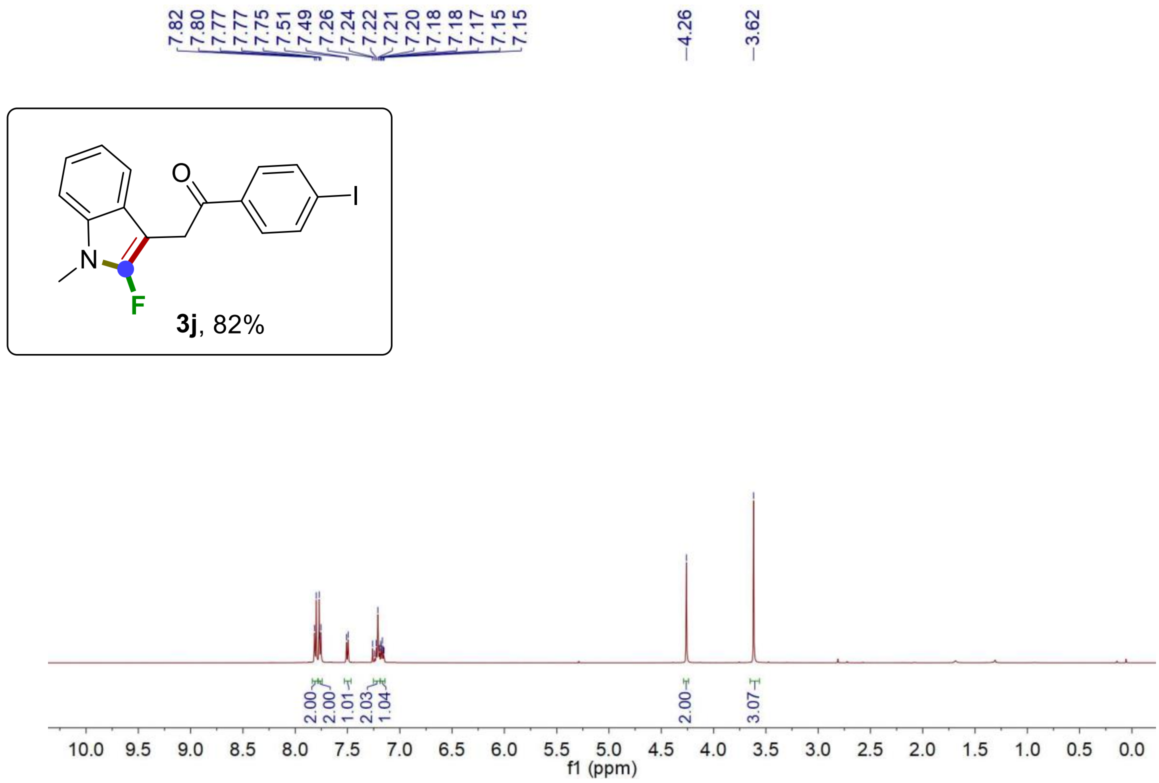


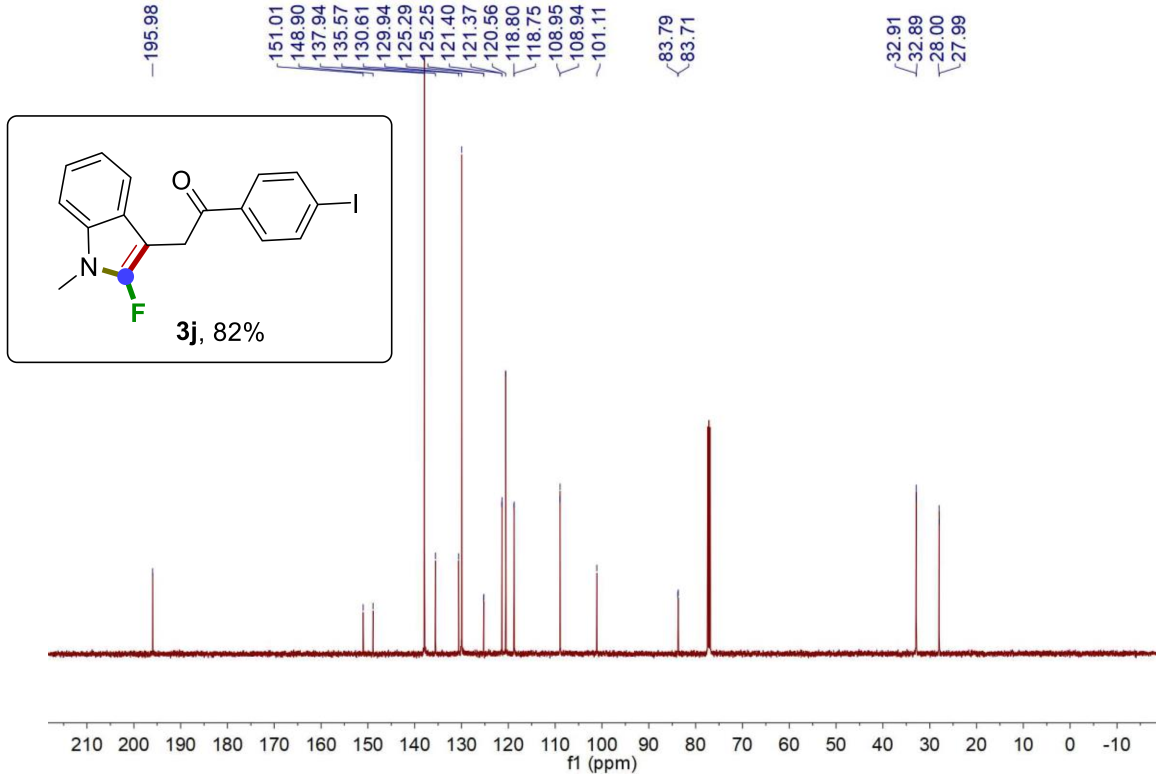

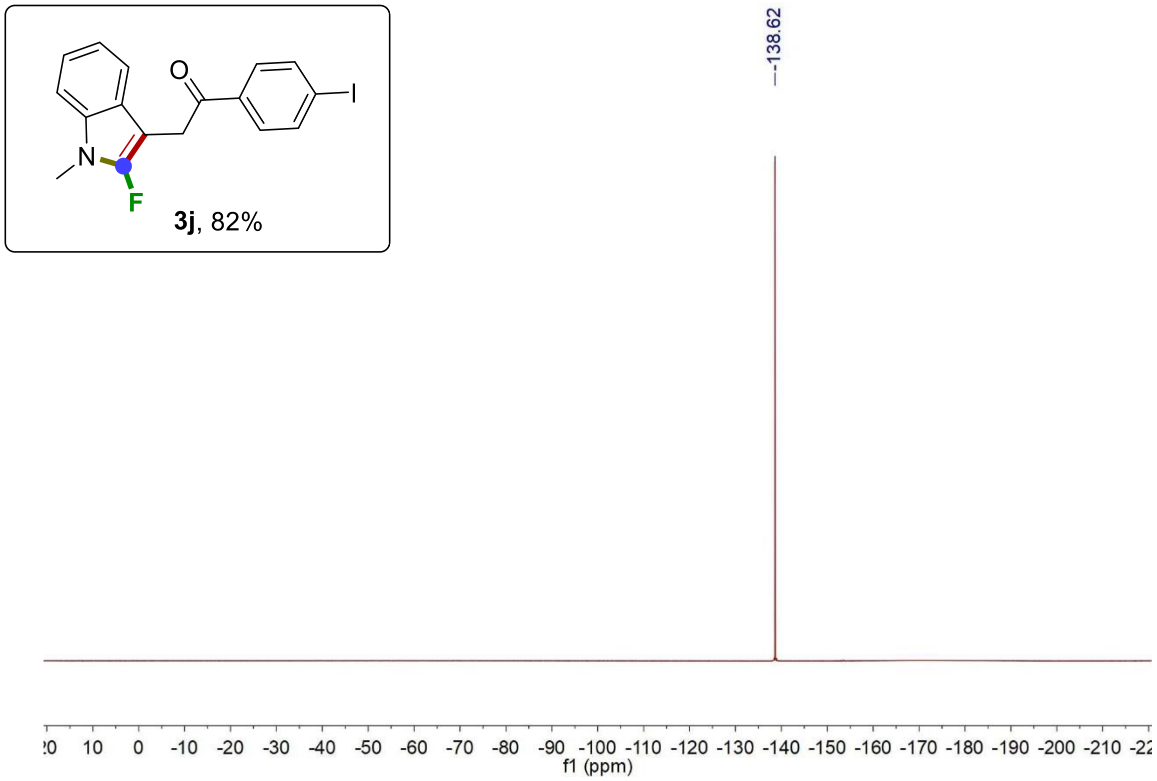


Supplementary Figure 102. $^{1}H$ NMR, $^{13}C$ NMR and $^{19}F$ NMR spectrum of **3j**.

**2-(2-fluoro-1-methyl-1H-indol-3-yl)-1-(naphthalen-2-yl)ethan-1-one (3k)**

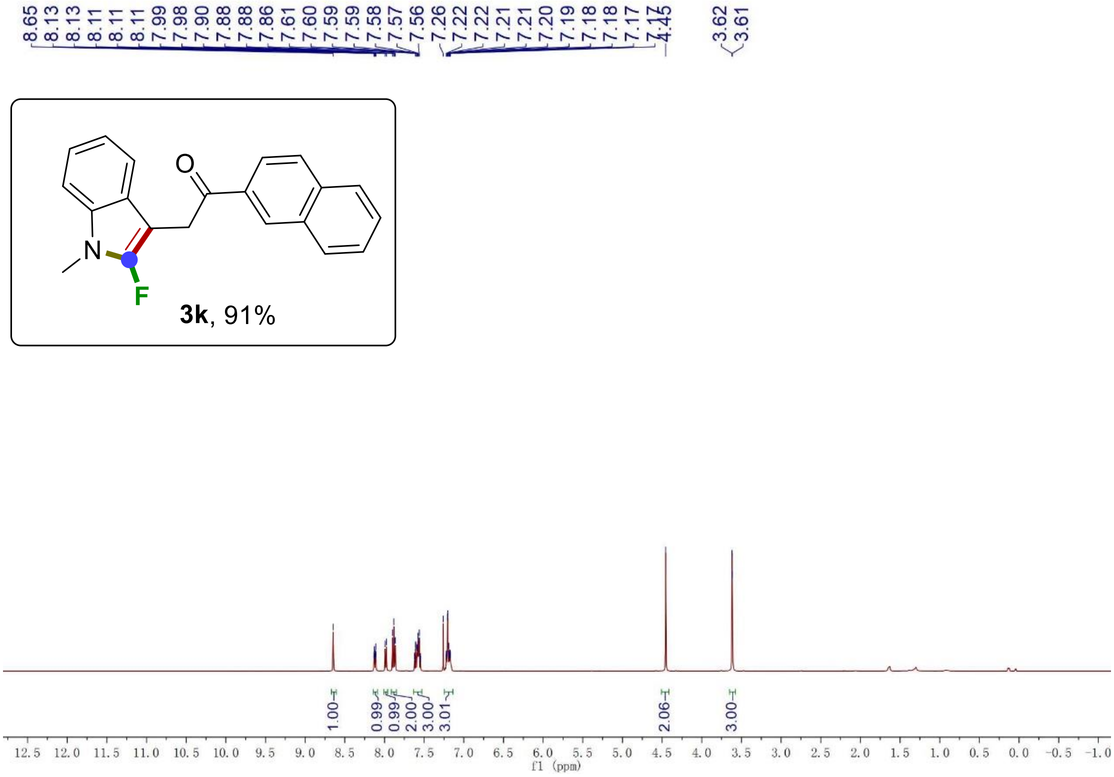


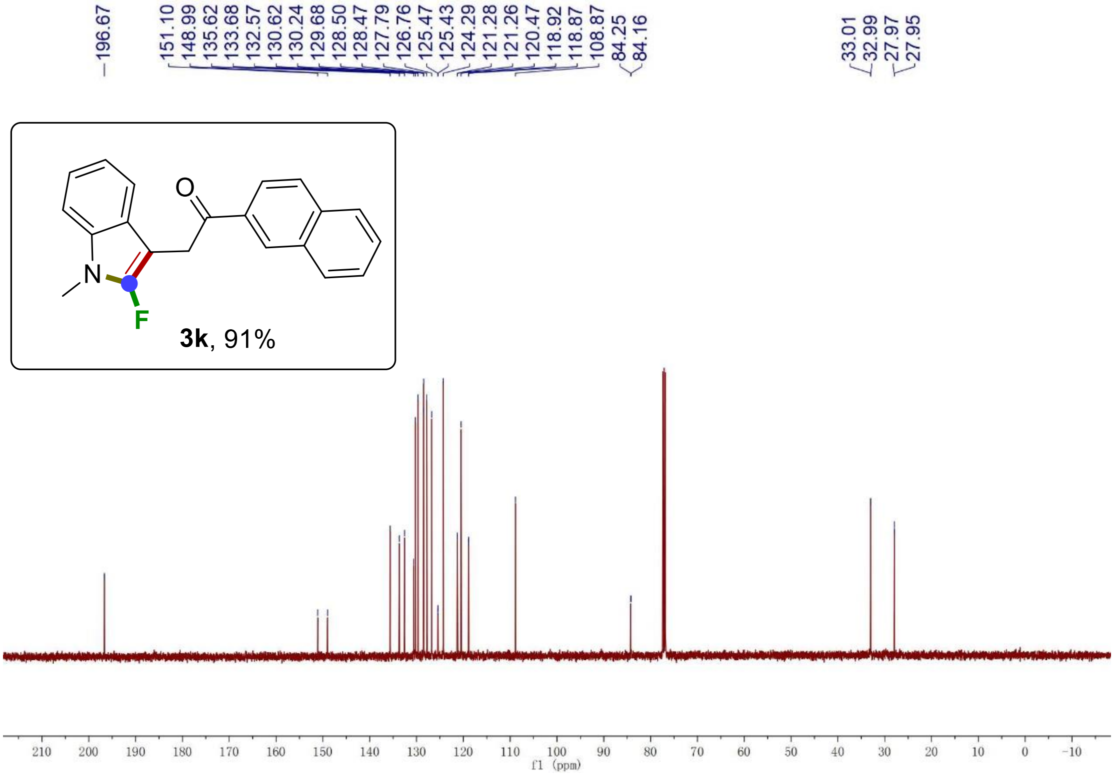

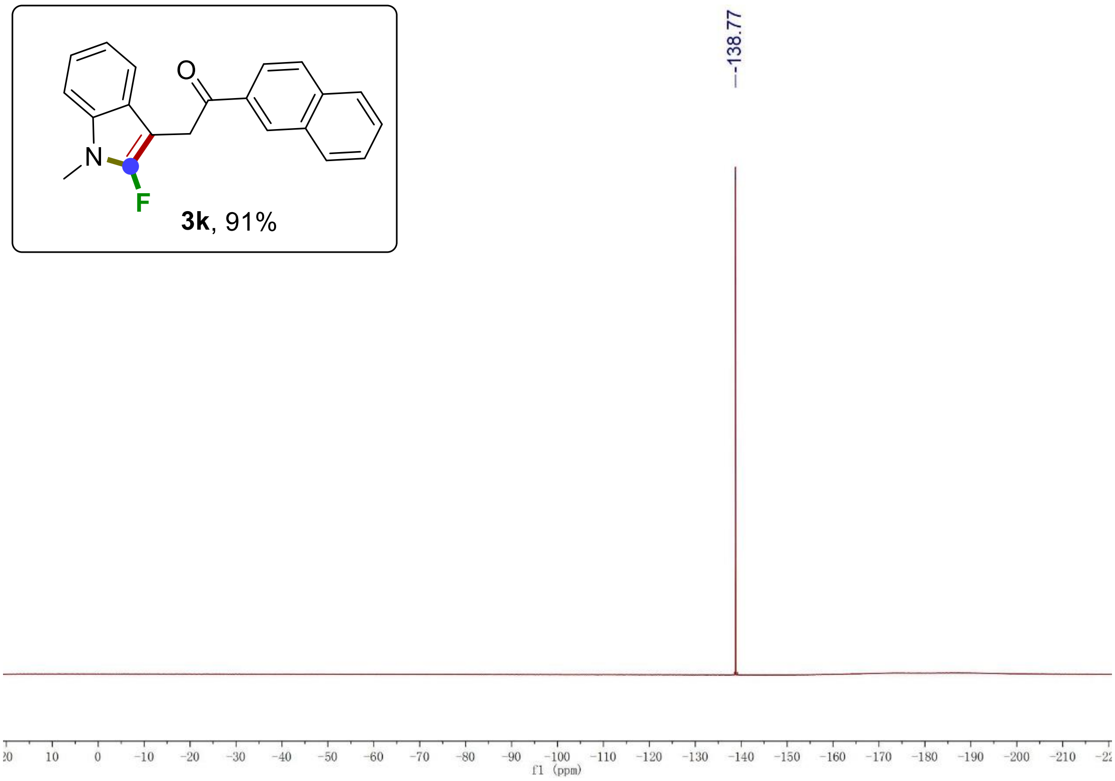


Supplementary Figure 103. $^{1}H$ NMR, $^{13}C$ NMR and $^{19}F$ NMR spectrum of **3k**.

## 2-(2-fluoro-1-methyl-1H-indol-3-yl)-1-(furan-2-yl)ethan-1-one (3l)

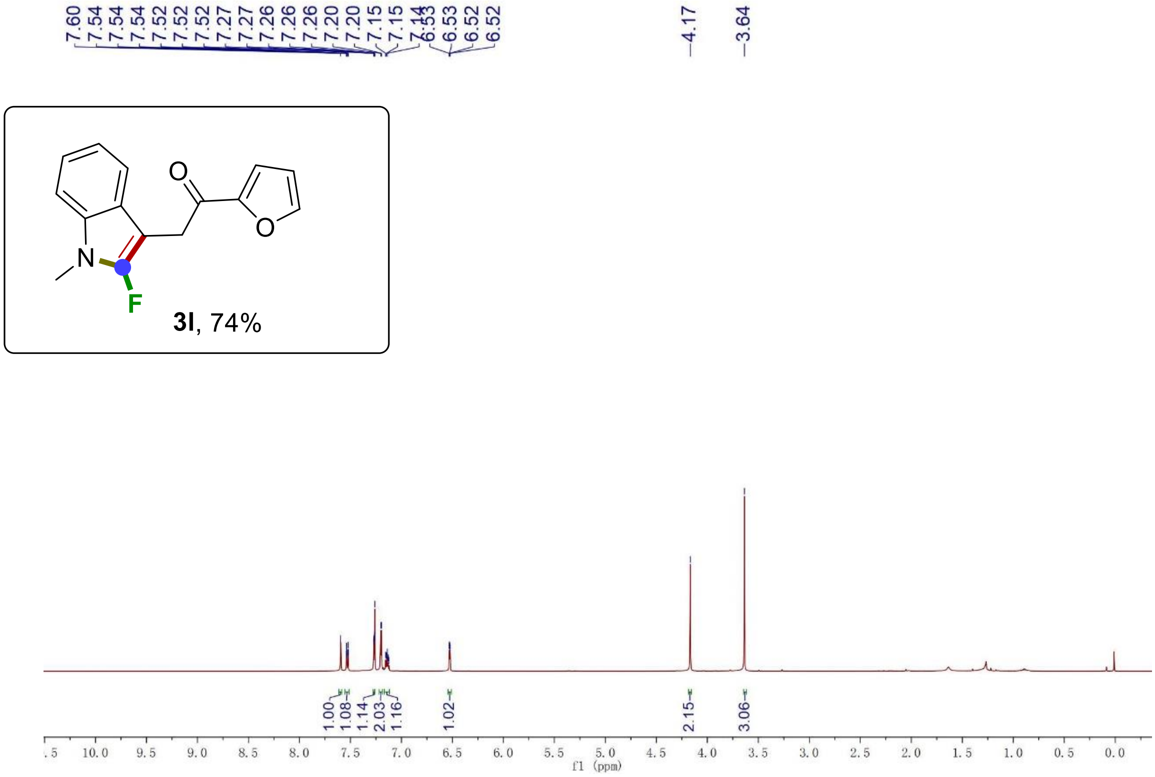


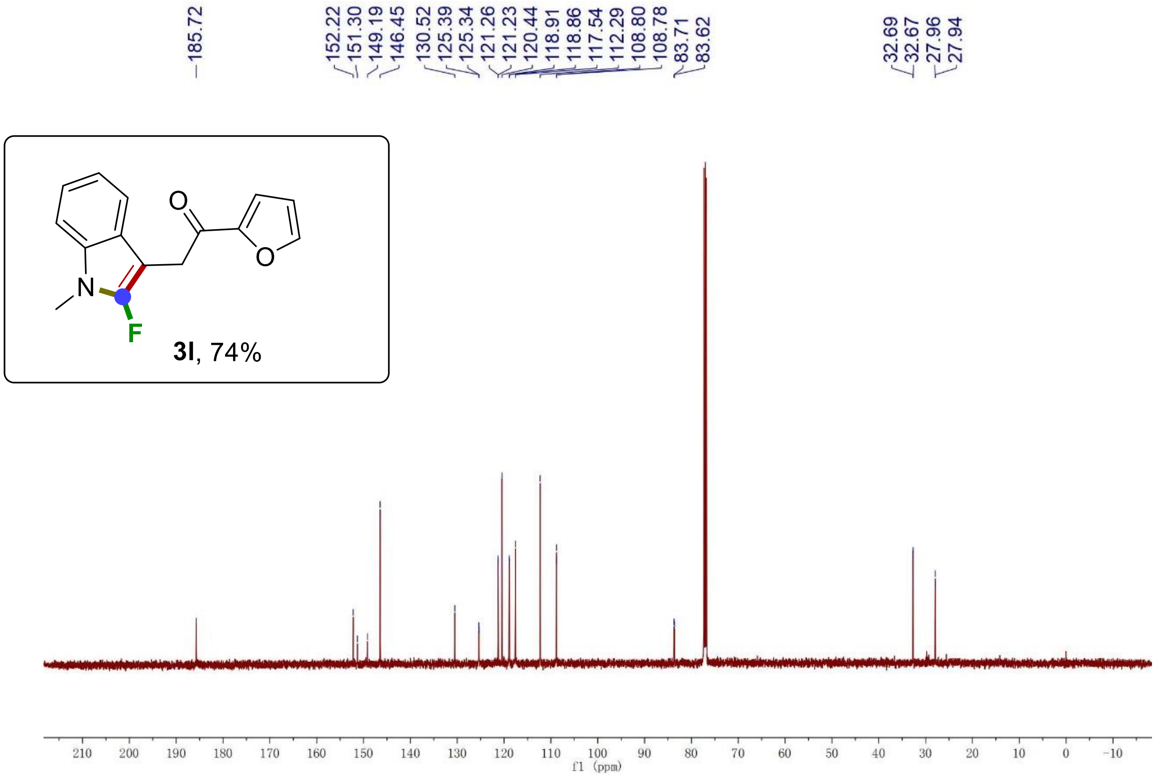

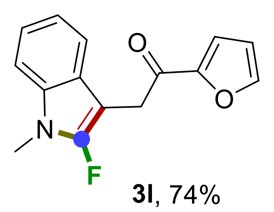


**3l**, 74%

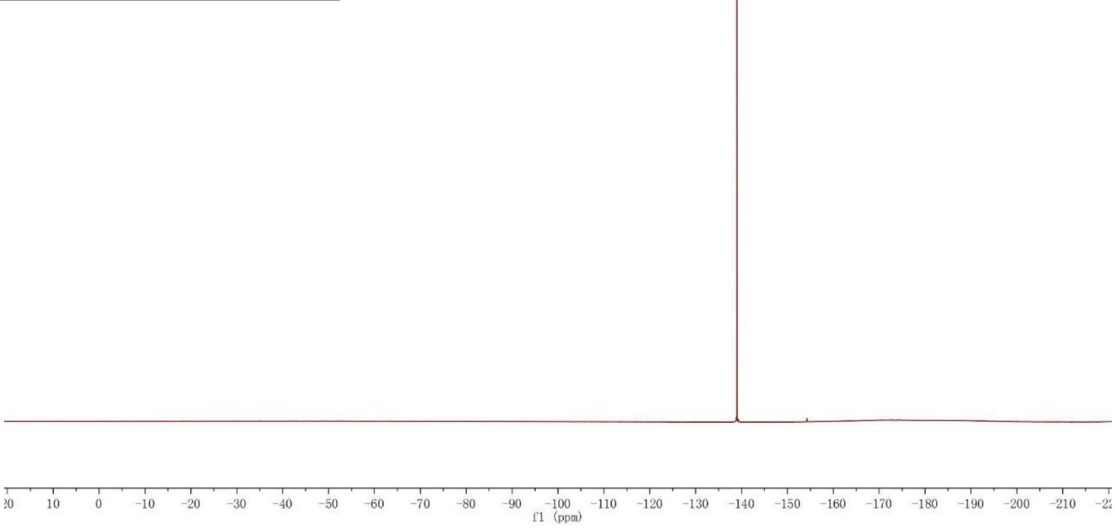


Supplementary Figure 104. $^{1}H$ NMR, $^{13}C$ NMR and $^{19}F$ NMR spectrum of **3l**.

## 2-(2-fluoro-1-methyl-1H-indol-3-yl)-1-(thiophen-3-yl)ethan-1-one (3m)

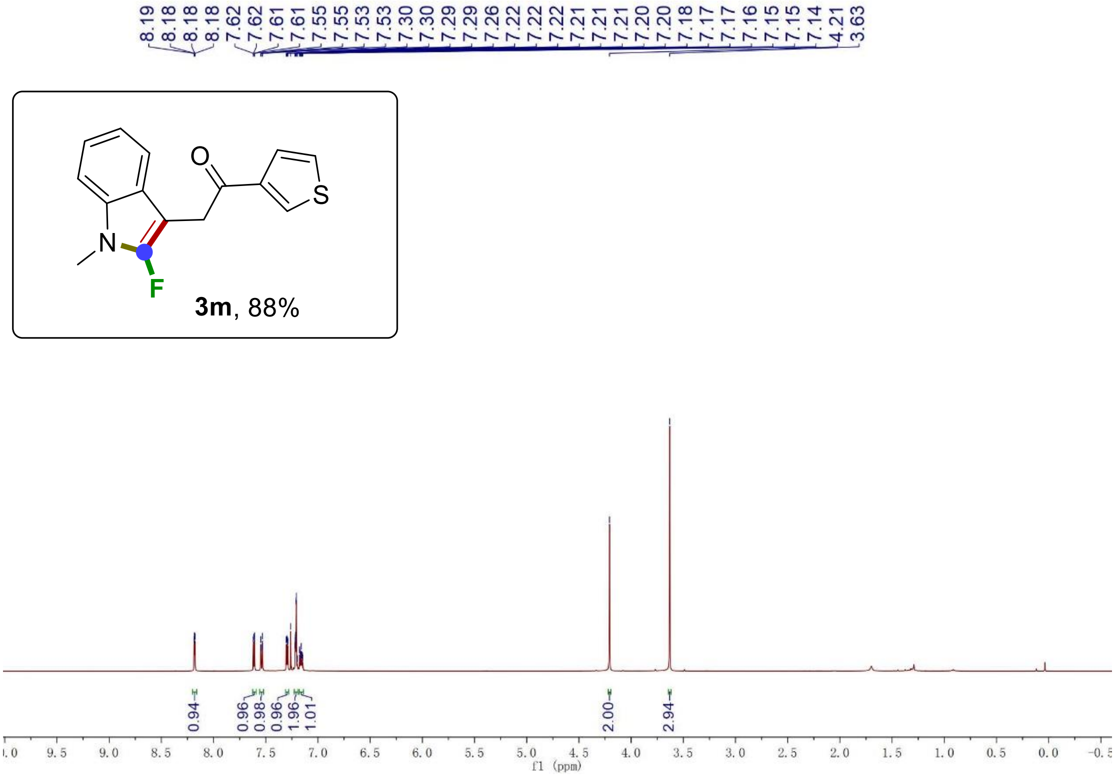


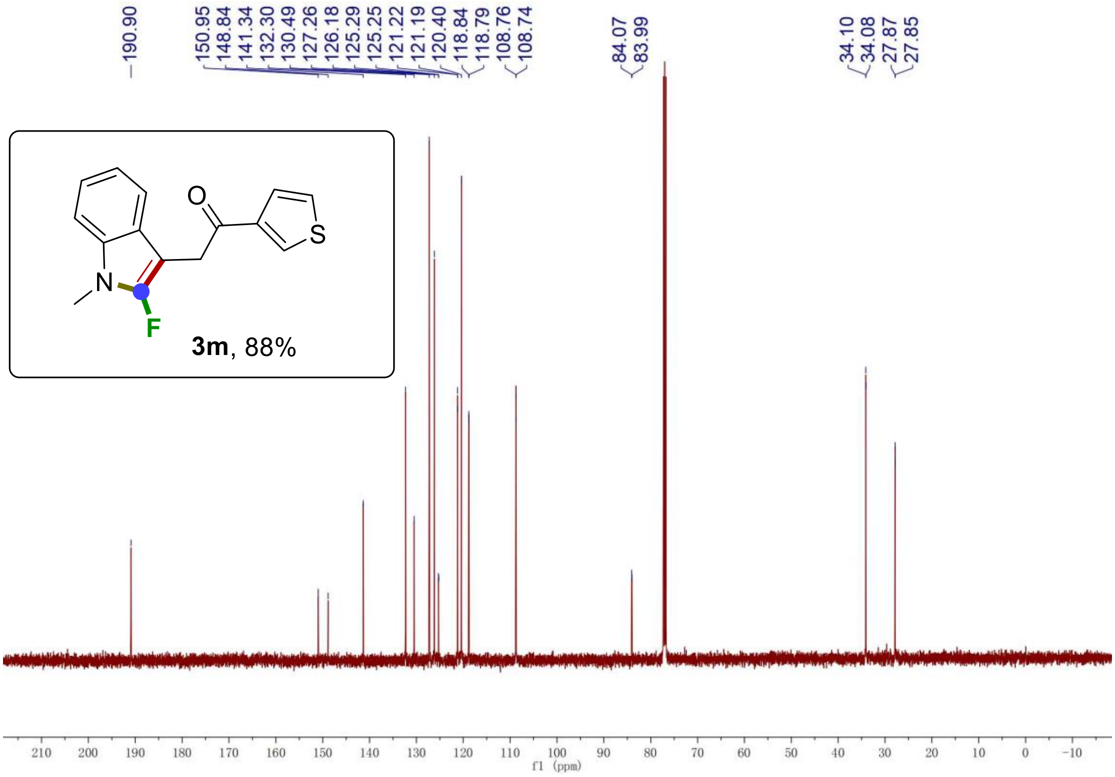

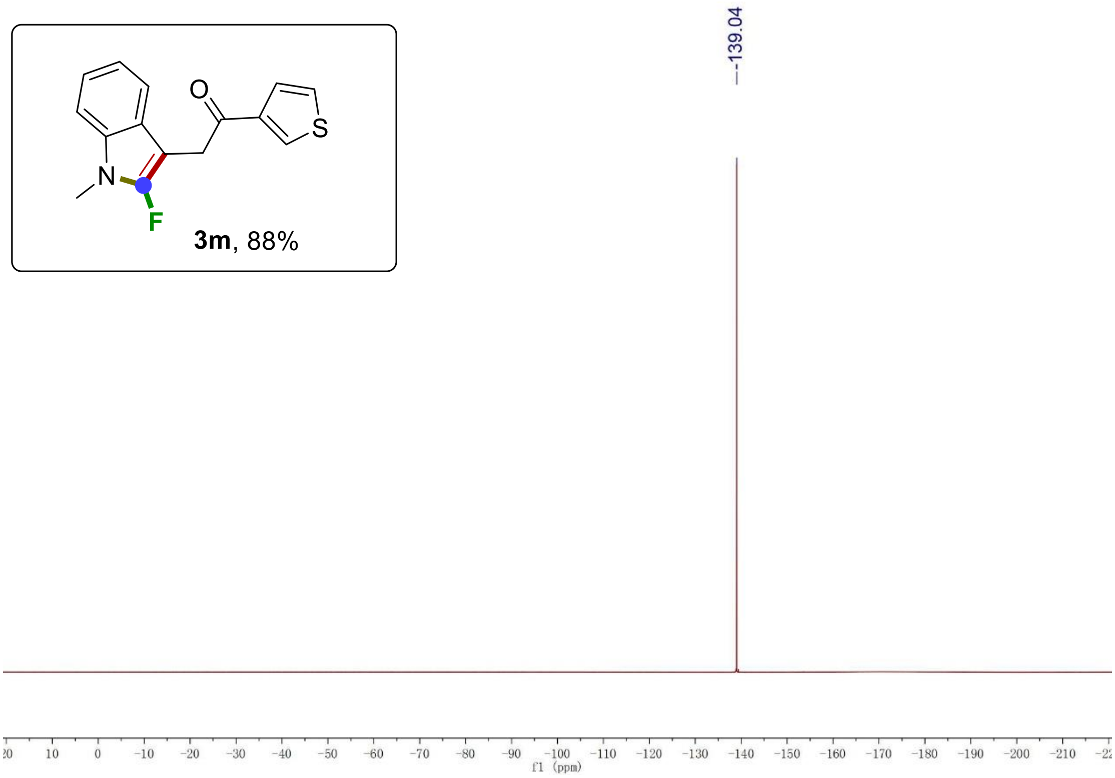


Supplementary Figure 105. $^{1}H$ NMR, $^{13}C$ NMR and $^{19}F$ NMR spectrum of **3m**.

## 1,1'-(1,3-phenylene)bis(2-(2-fluoro-1-methyl-1H-indol-3-yl)ethan-1-one) (3n)

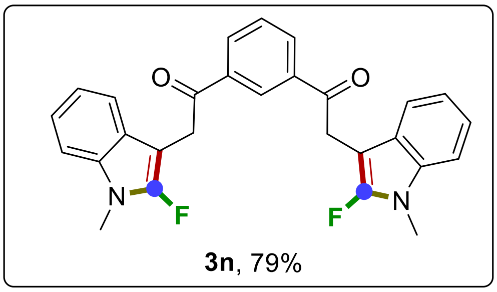


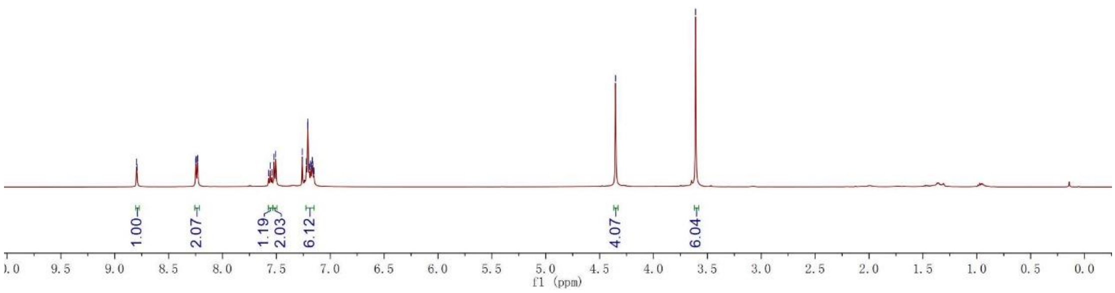


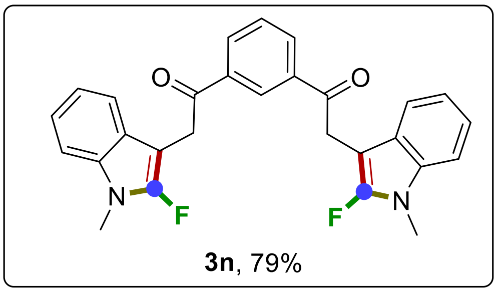


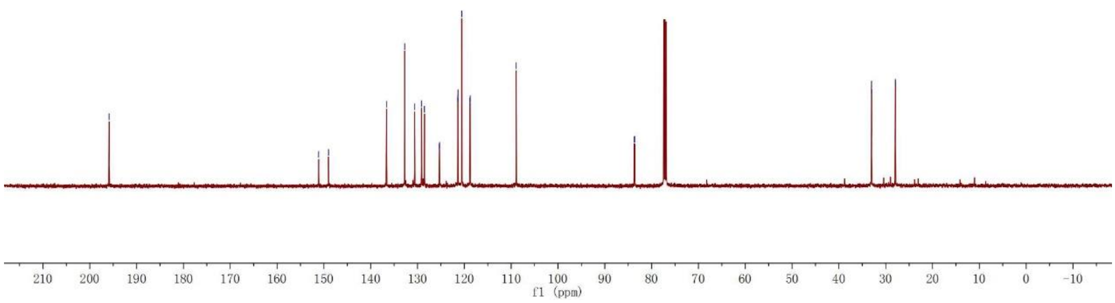

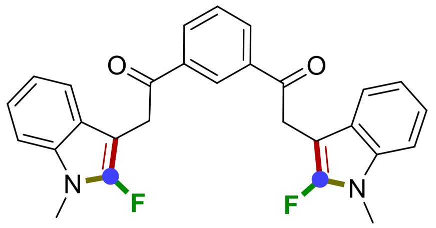


**3n**, 79%

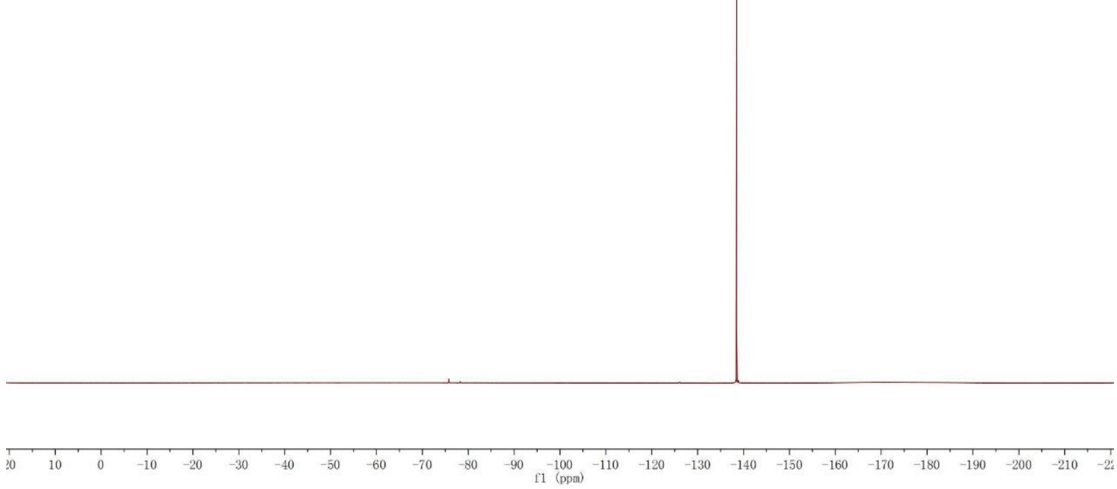


Supplementary Figure 106. $^{1}H$ NMR, $^{13}C$ NMR and $^{19}F$ NMR spectrum of **3n**.

## 2-(2-fluoro-1-methyl-1H-indol-3-yl)-2,3-dihydro-1H-inden-1-one (3o)

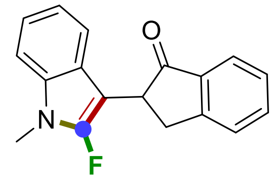


**3o**, 75%

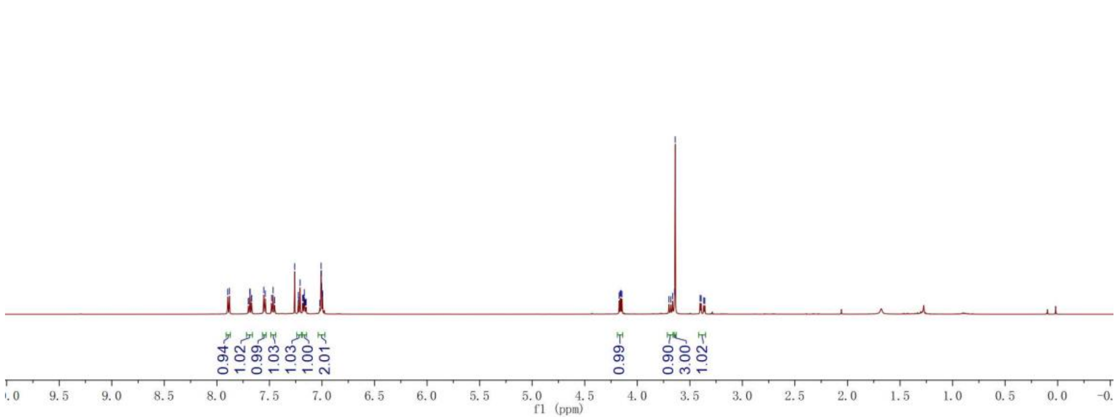


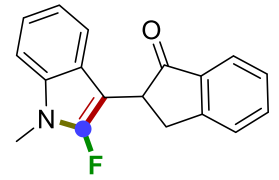


**3o**, 75%

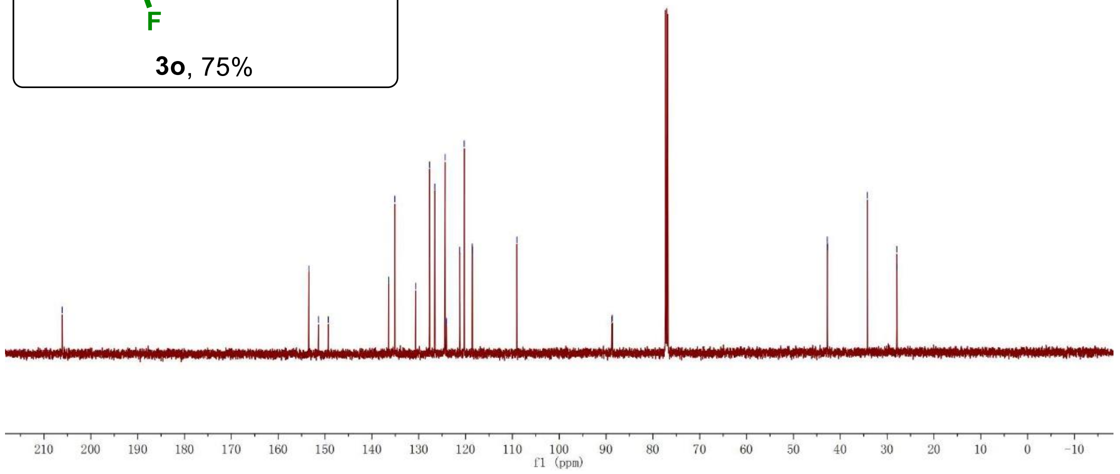

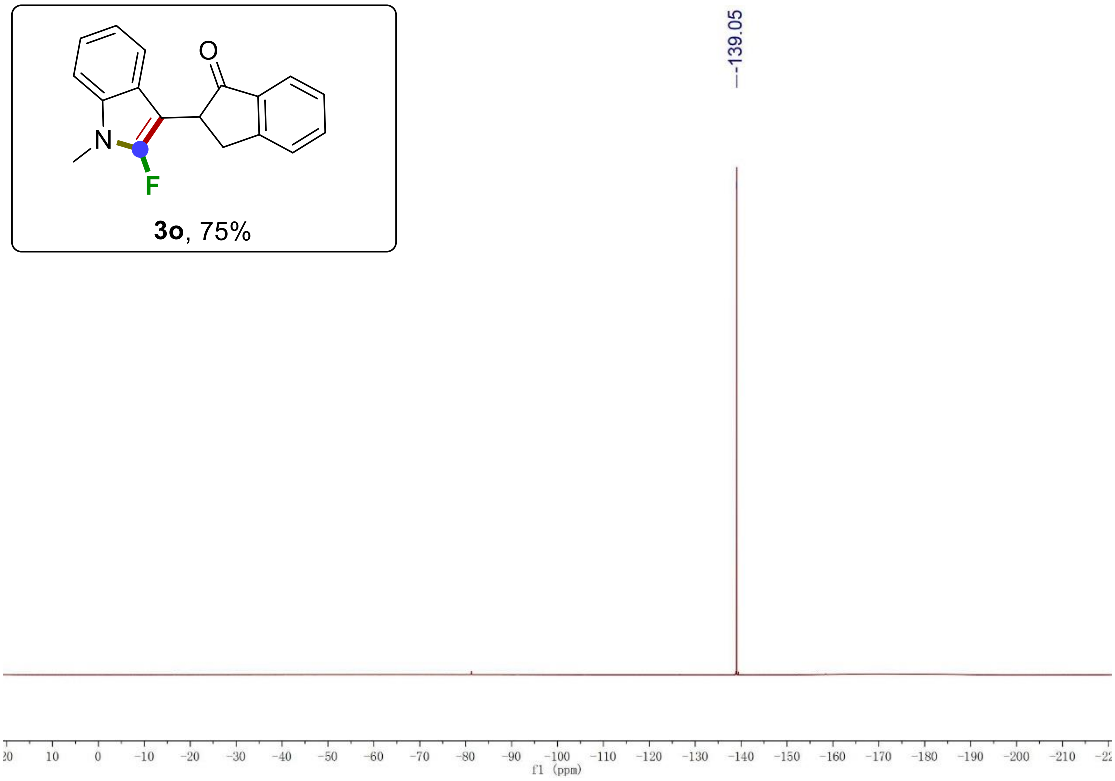


Supplementary Figure 107. $^{1}H$ NMR, $^{13}C$ NMR and $^{19}F$ NMR spectrum of **3o**.

## 2-(2-fluoro-1-methyl-1H-indol-3-yl)-3,4-dihydronaphthalen-1(2H)-one (3p)

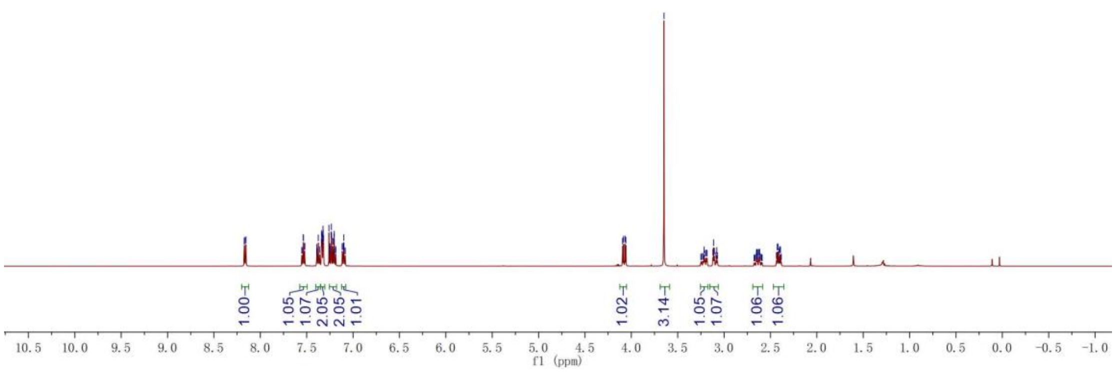


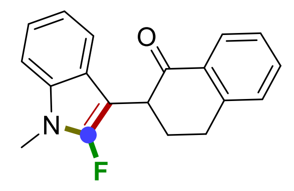


**3p**, 79%

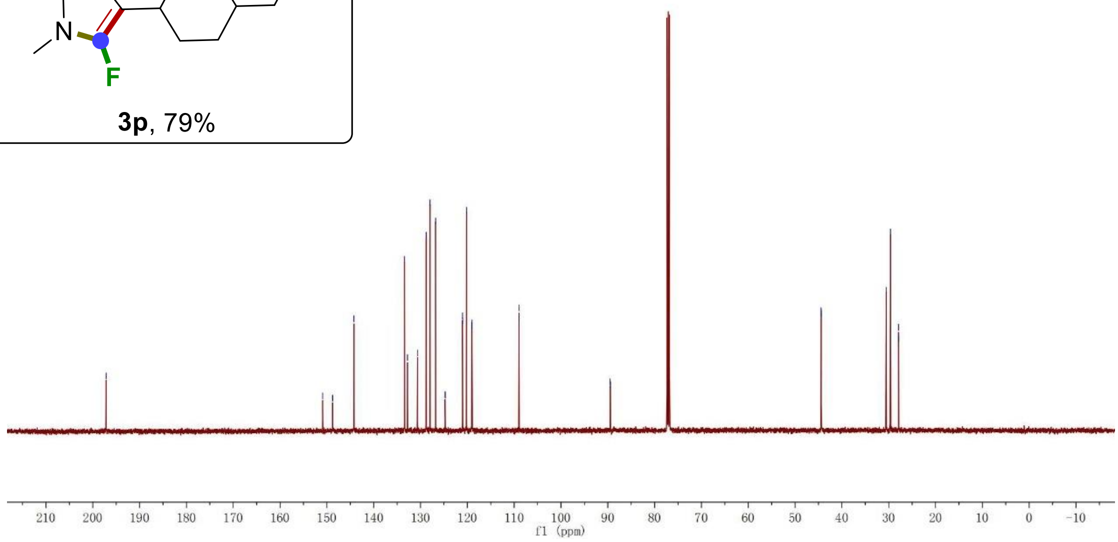


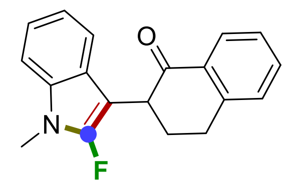


**3p**, 79%

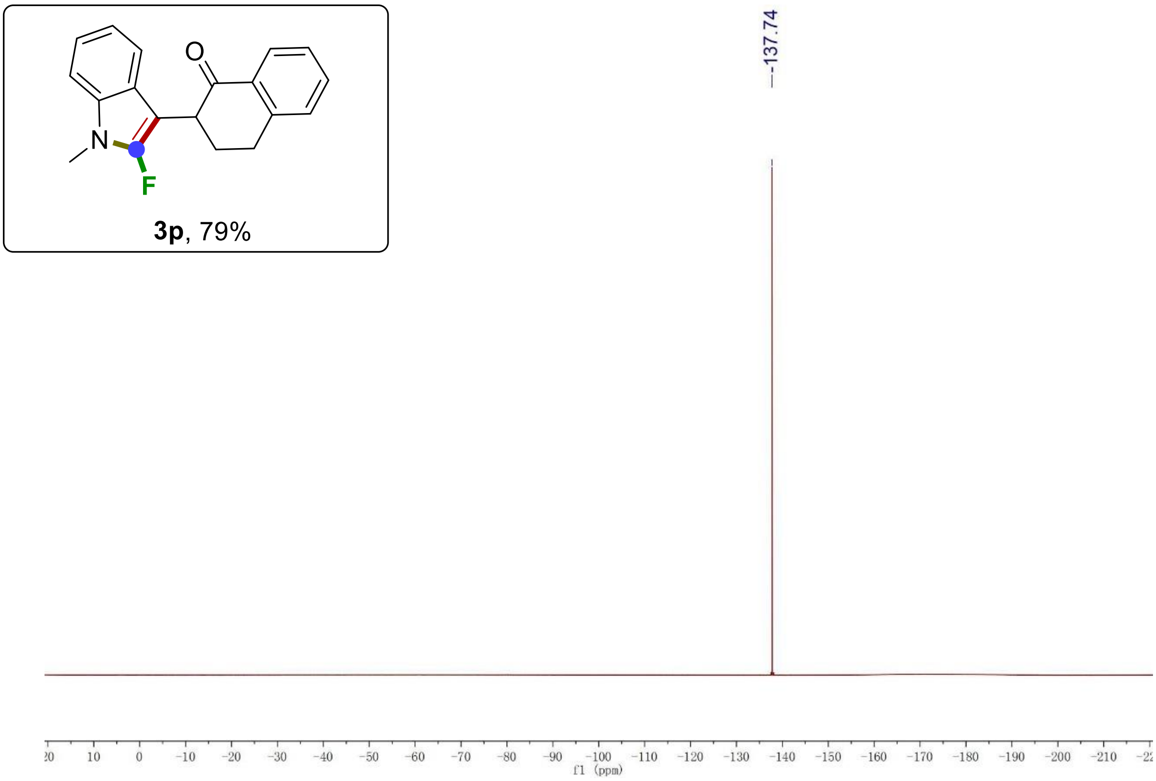


Supplementary Figure 108. $^{1}H$ NMR, $^{13}C$ NMR and $^{19}F$ NMR spectrum of **3p**.

## 3-(2-fluoro-1-methyl-1H-indol-3-yl)chroman-4-one (3q)

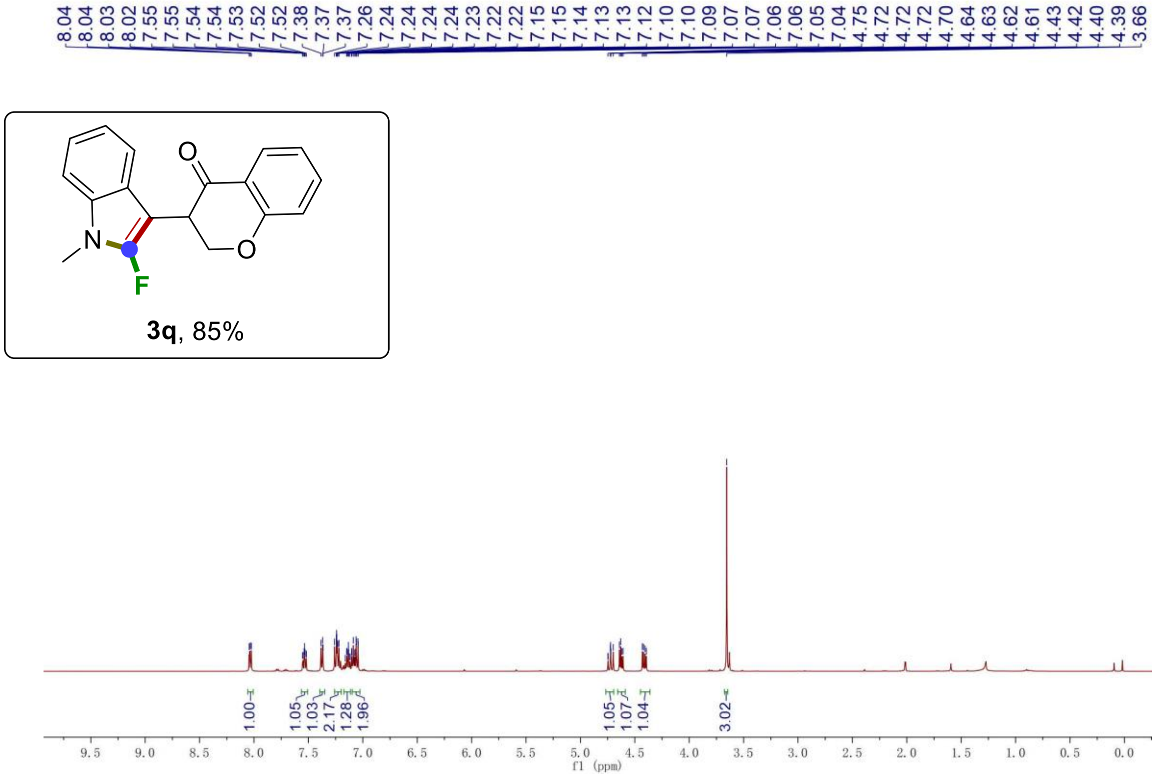


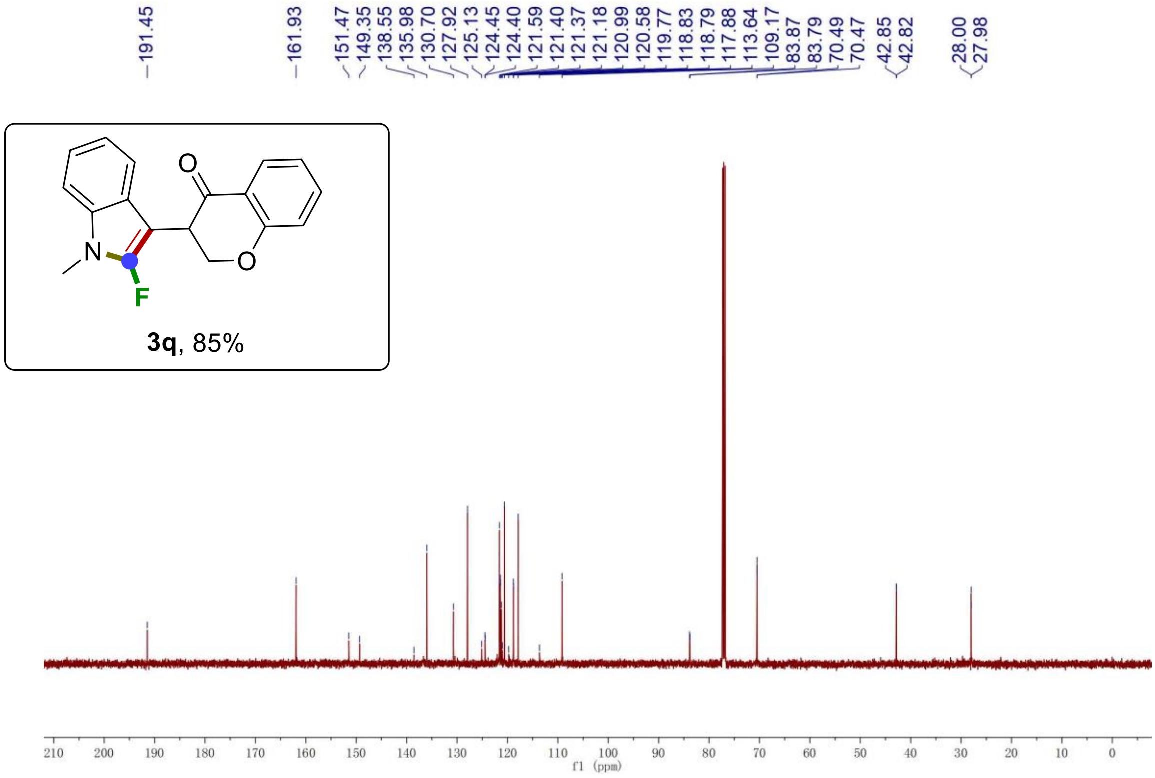

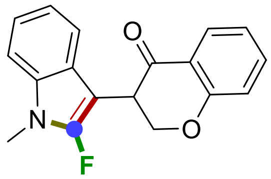

**3q**, 85%

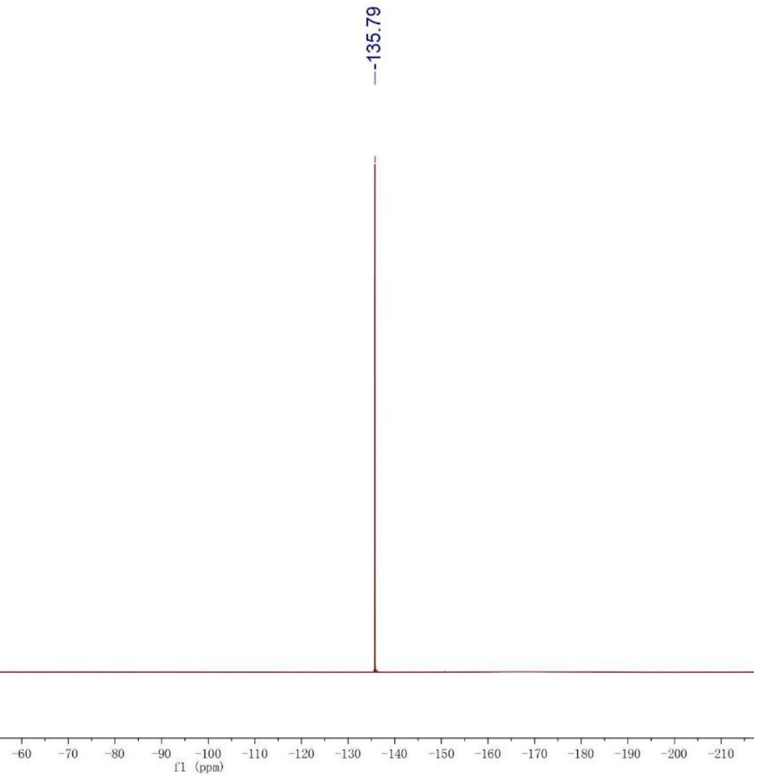


Supplementary Figure 109. $^{1}H$ NMR, $^{13}C$ NMR and $^{19}F$ NMR spectrum of **3q**.

**6-(2-fluoro-1-methyl-1H-indol-3-yl)-6,7,8,9-tetrahydro-5H-benzo[7]annulen-5-one (3r)**

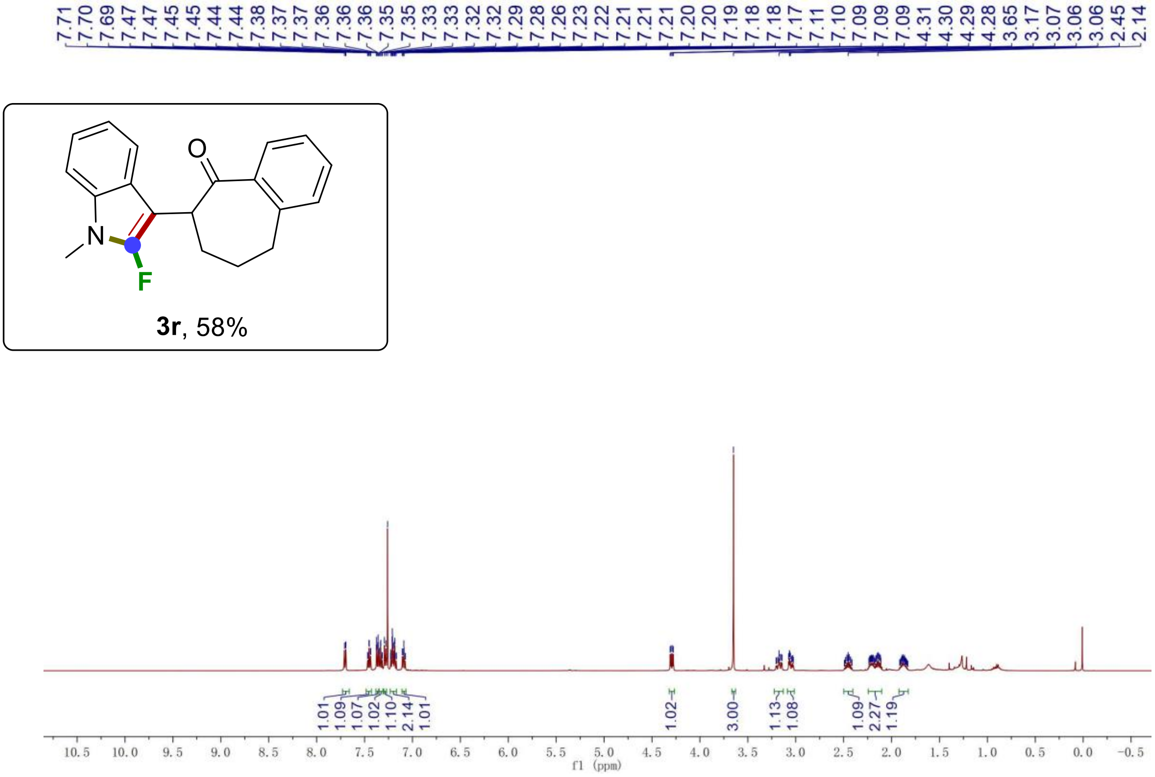


**3r**, 58%

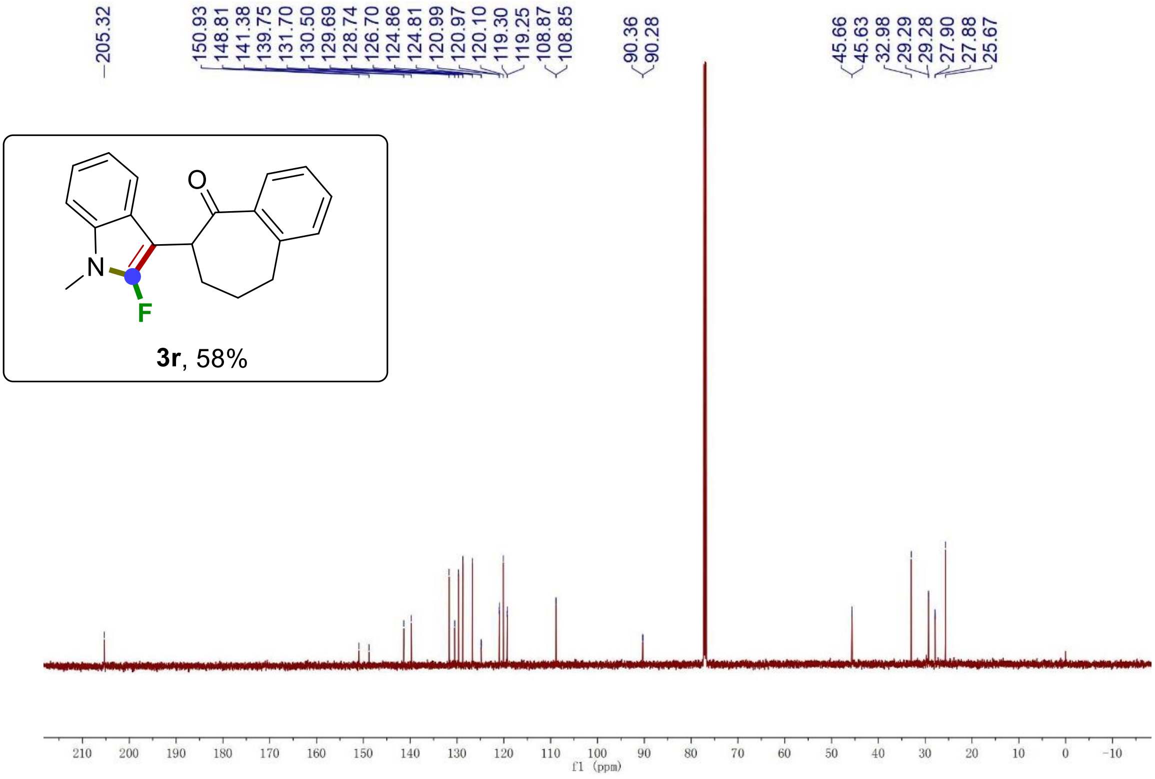


**3r**, 58%

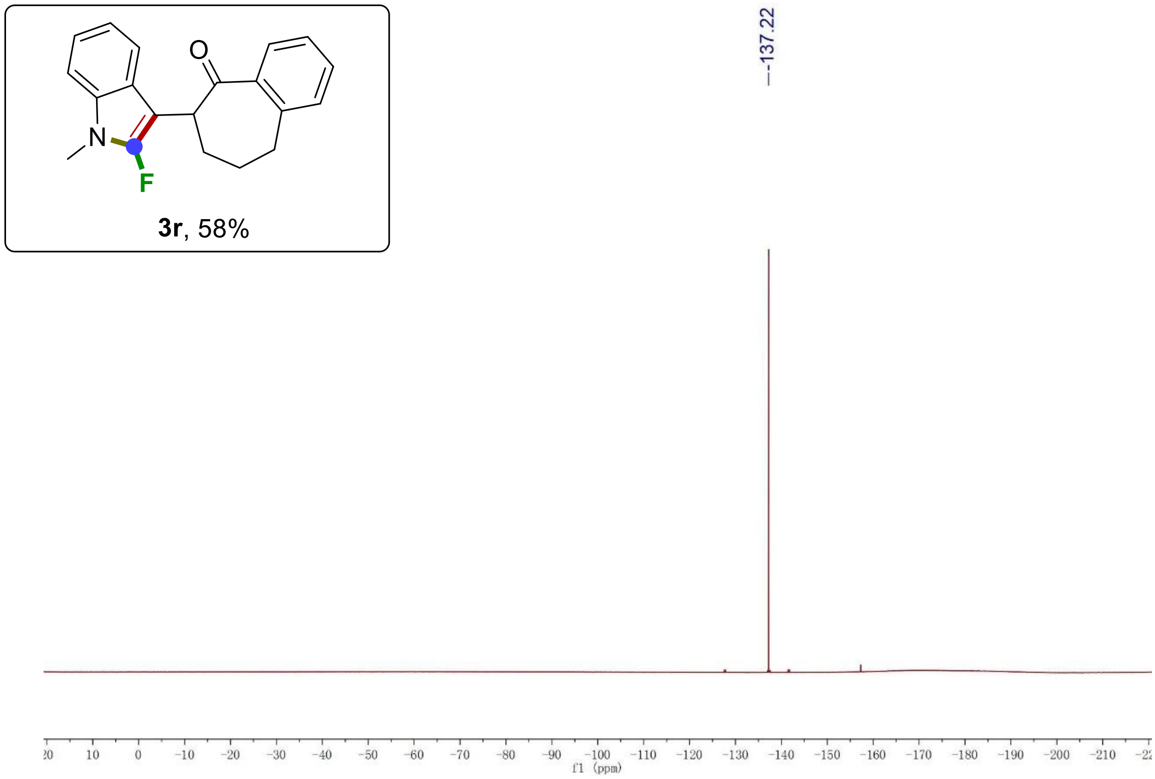


Supplementary Figure 110. $^{1}H$ NMR, $^{13}C$ NMR and $^{19}F$ NMR spectrum of **3r**.

1-(2-fluoro-1-methyl-1H-indol-3-yl)propan-2-one (3s)

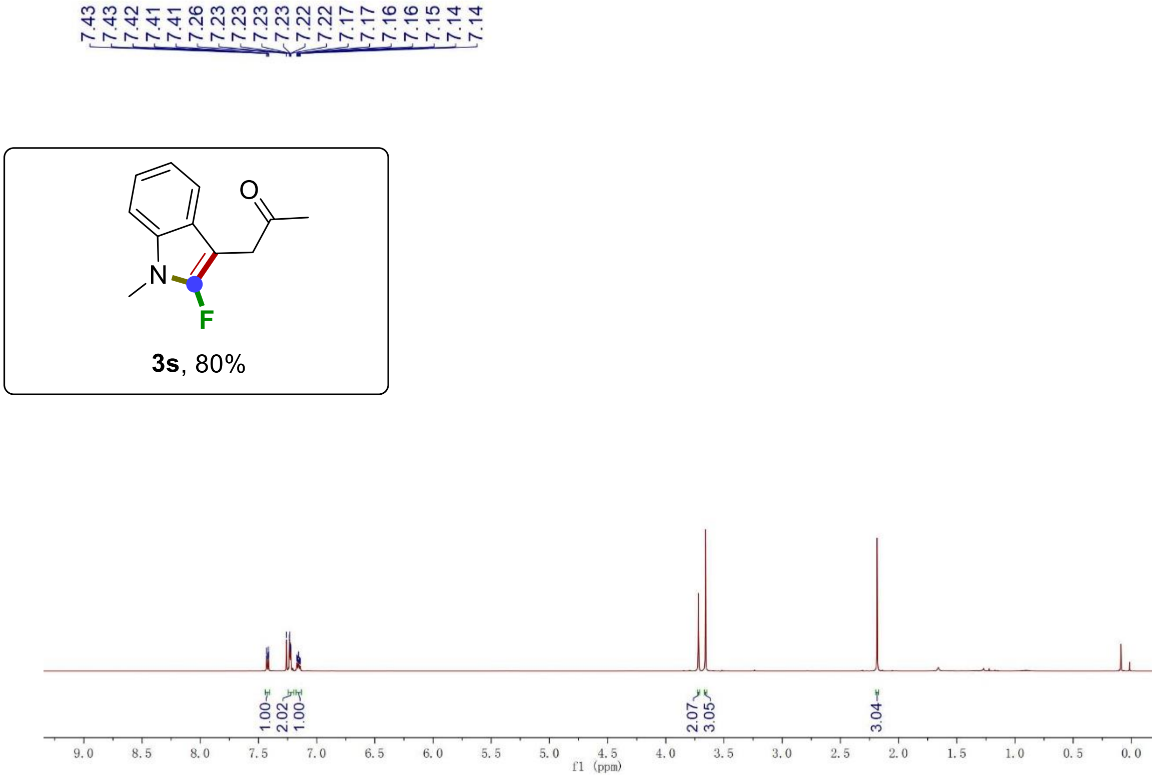


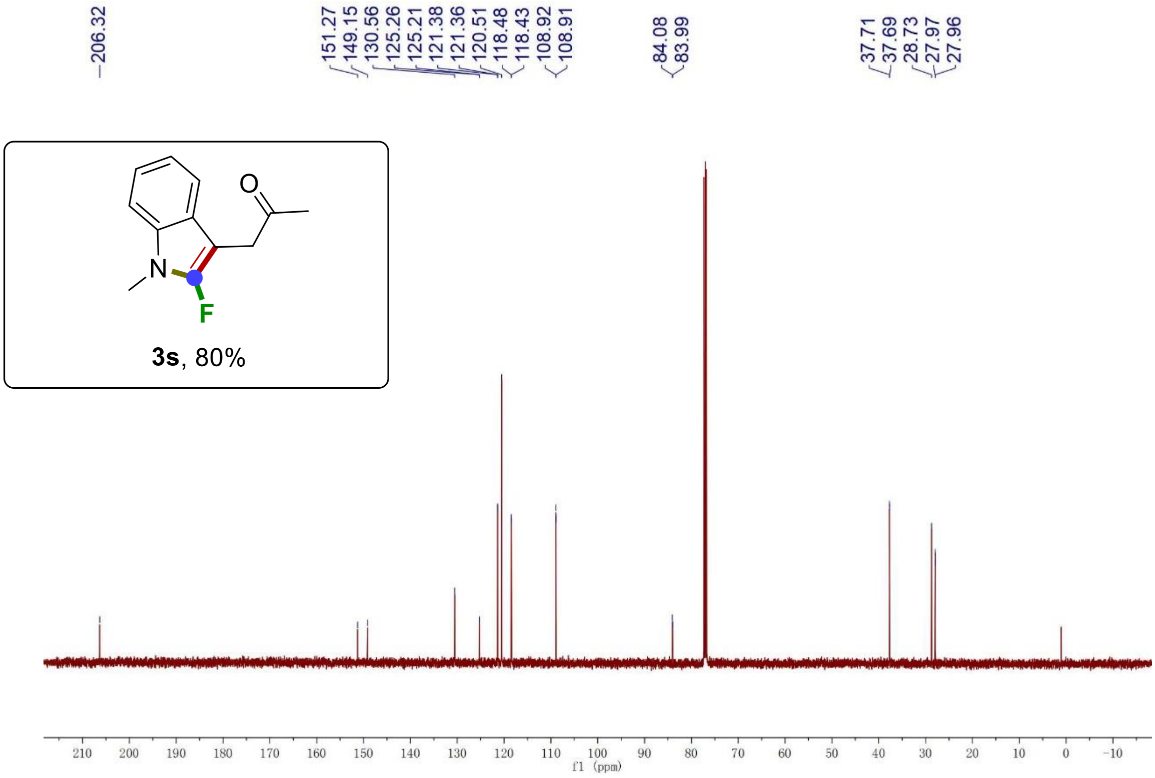

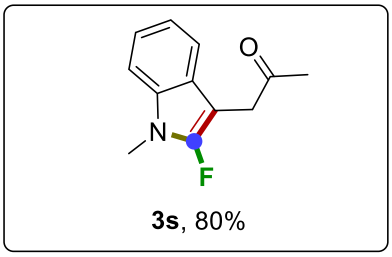


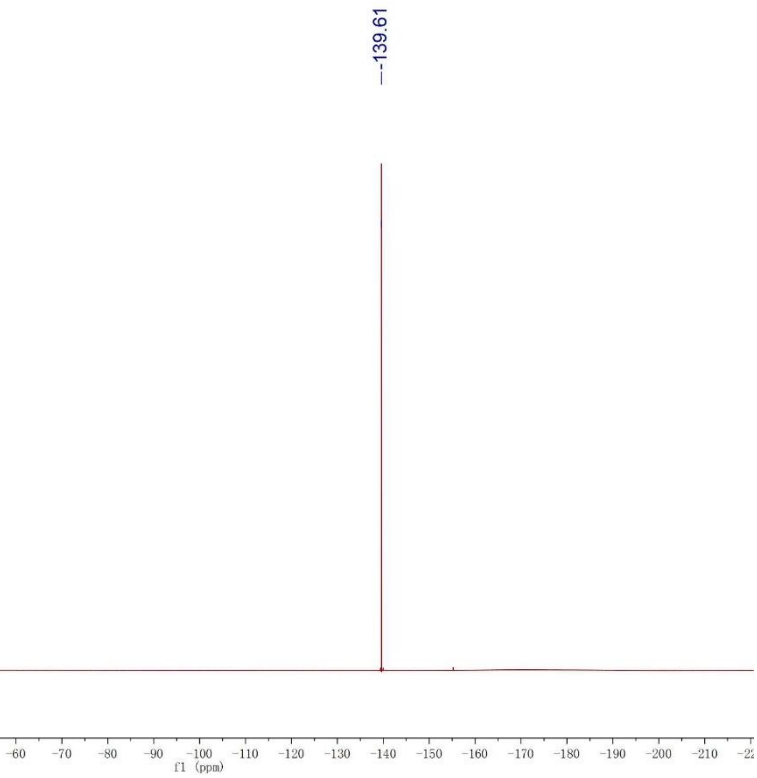


Supplementary Figure 111. $^{1}H$ NMR, $^{13}C$ NMR and $^{19}F$ NMR spectrum of **3s**.

## 1-(2-fluoro-1-methyl-1H-indol-3-yl)-3-methylbutan-2-one (3t)

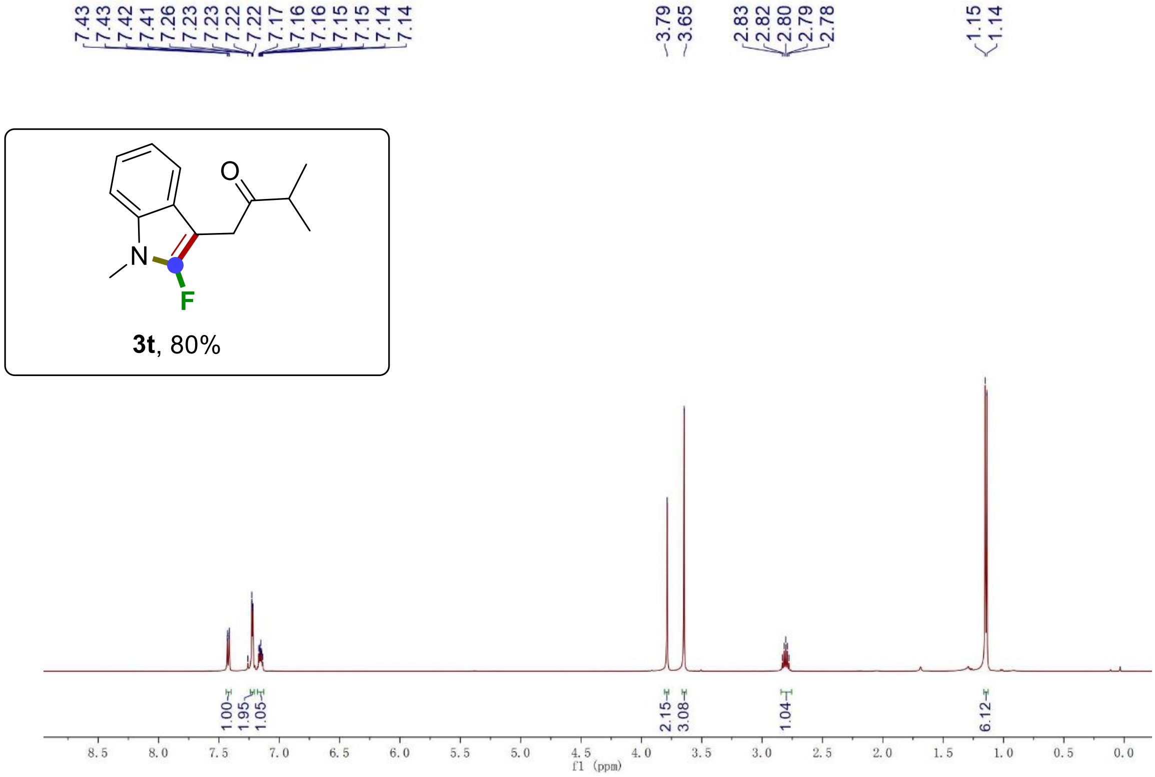


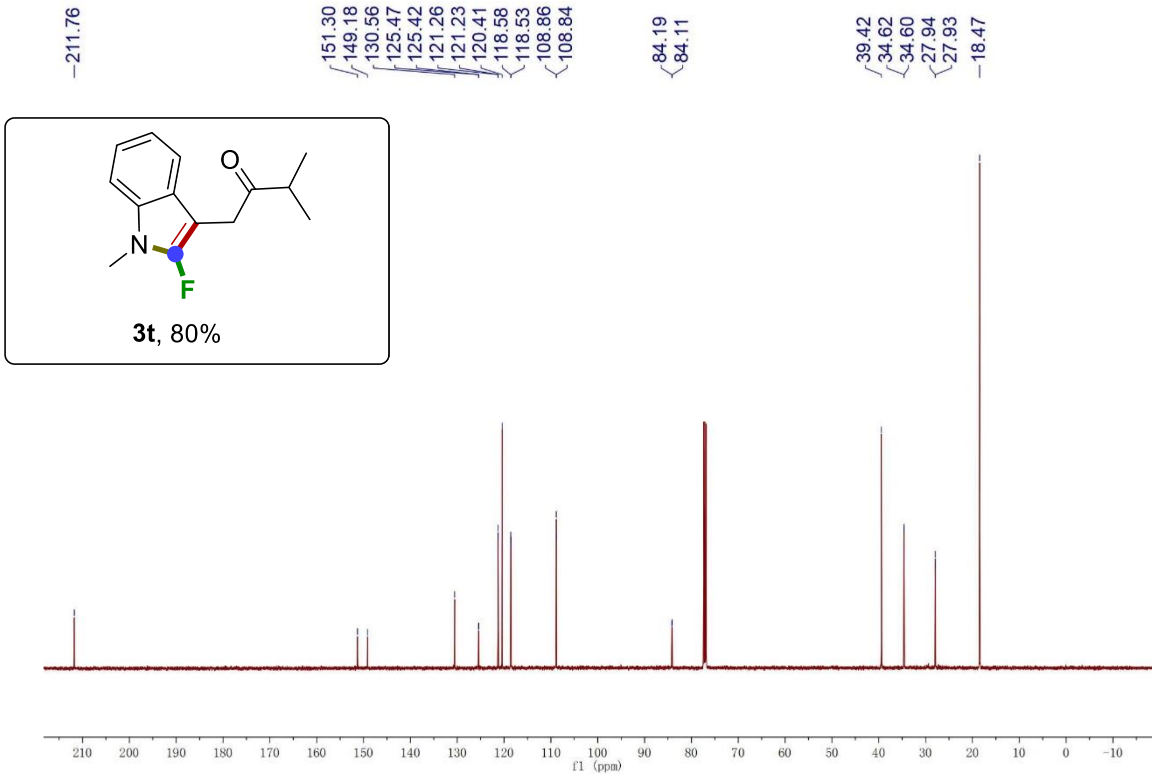

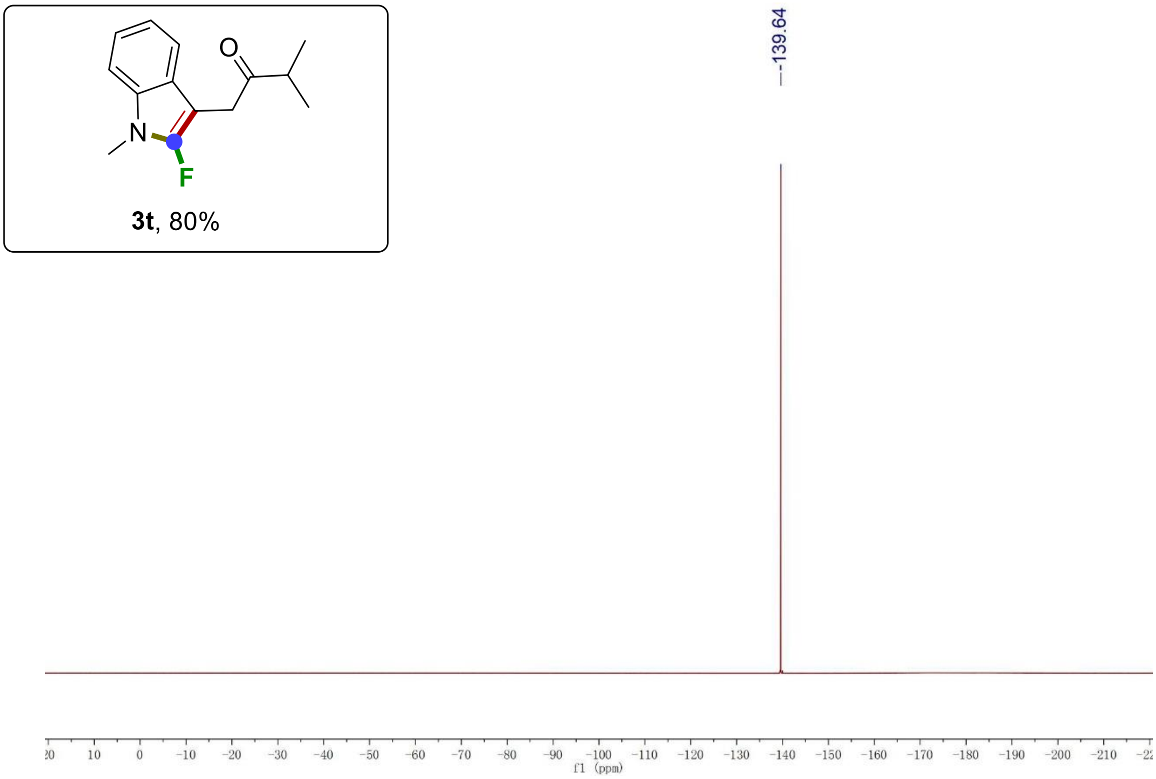


Supplementary Figure 112. $^{1}$H NMR, $^{13}$C NMR and $^{19}$F NMR spectrum of **3t**.

**1-(2-fluoro-1-methyl-1H-indol-3-yl)-3,3-dimethylbutan-2-one (3u)**

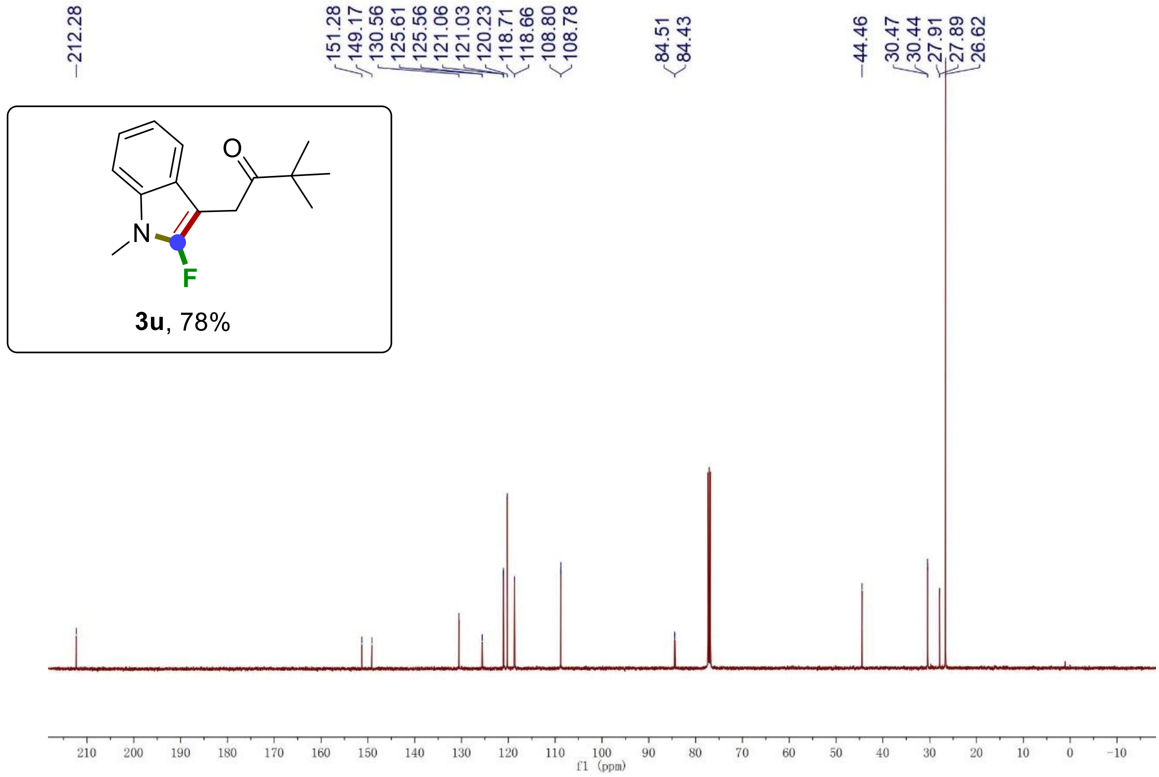


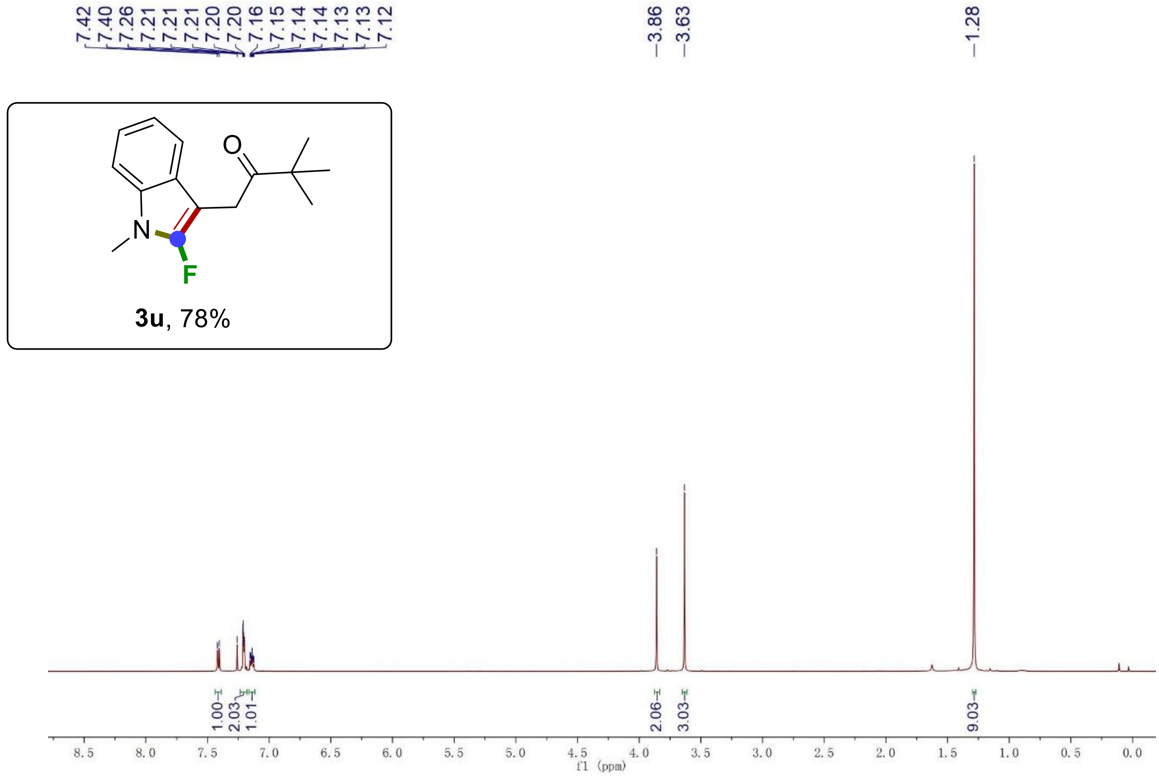

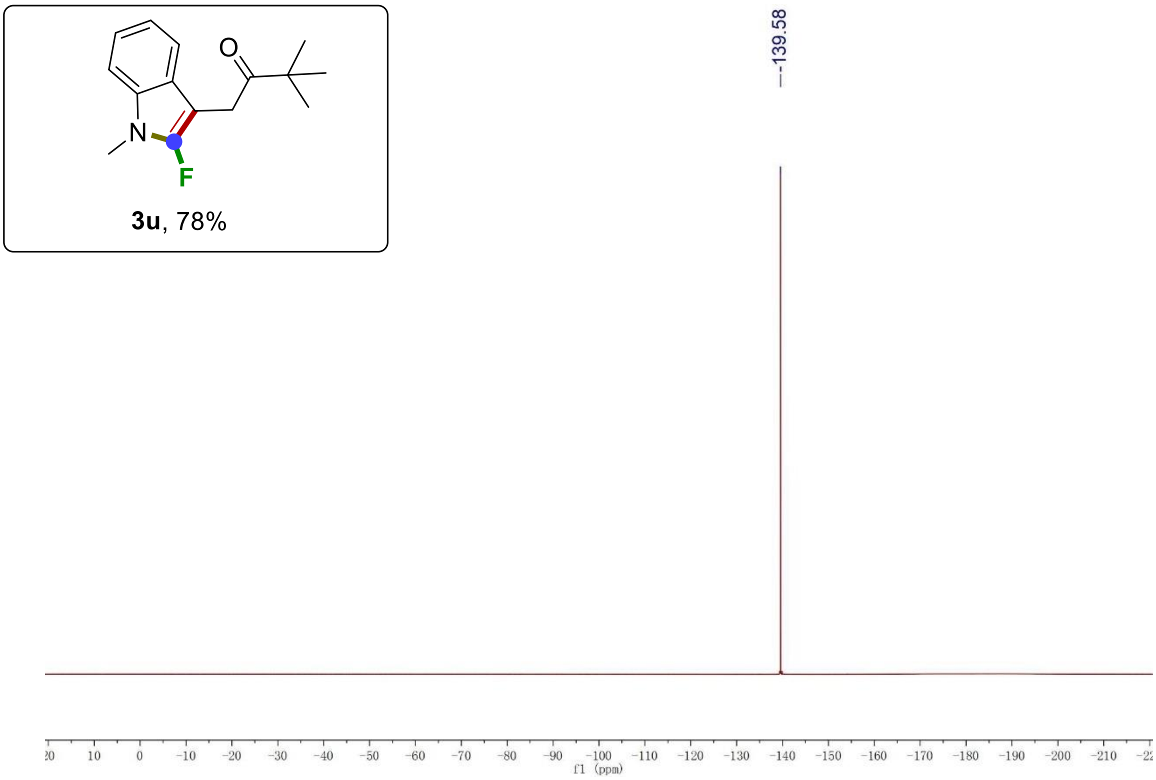


Supplementary Figure 113. $^{1}$H NMR, $^{13}$C NMR and $^{19}$F NMR spectrum of **3u**.

# 2-(2-fluoro-1-methyl-1H-indol-3-yl)pentan-3-one (3v)

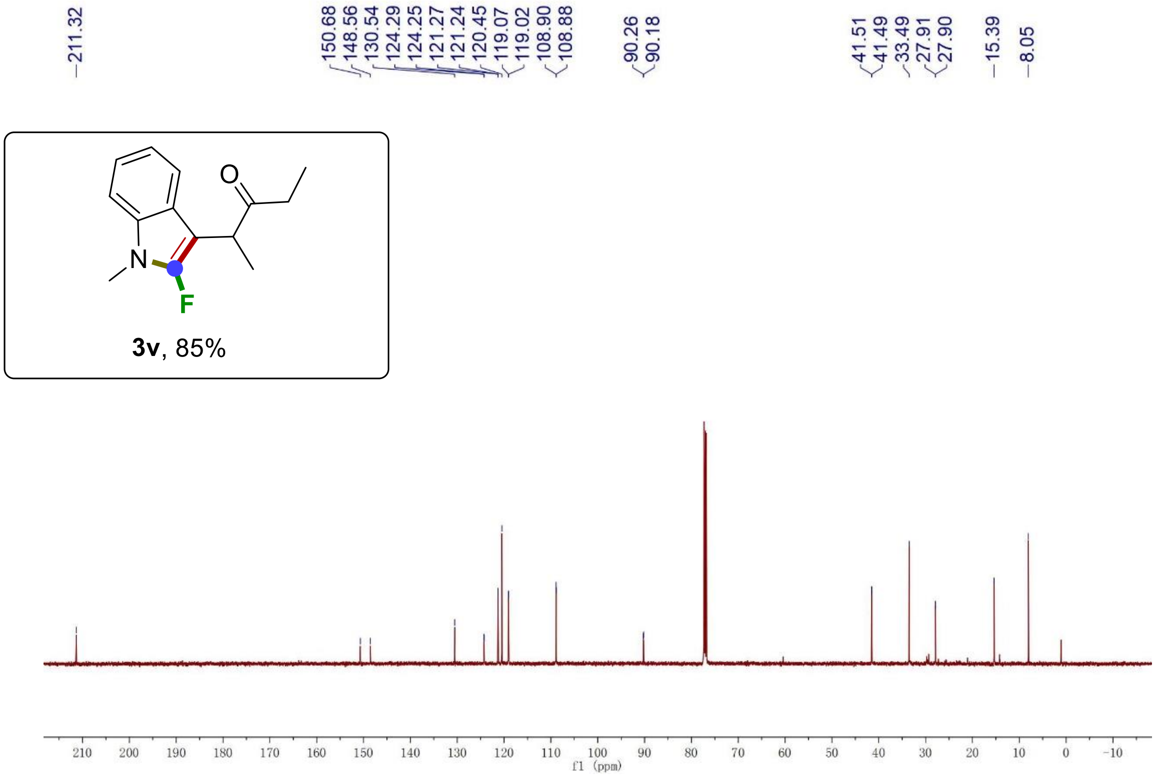


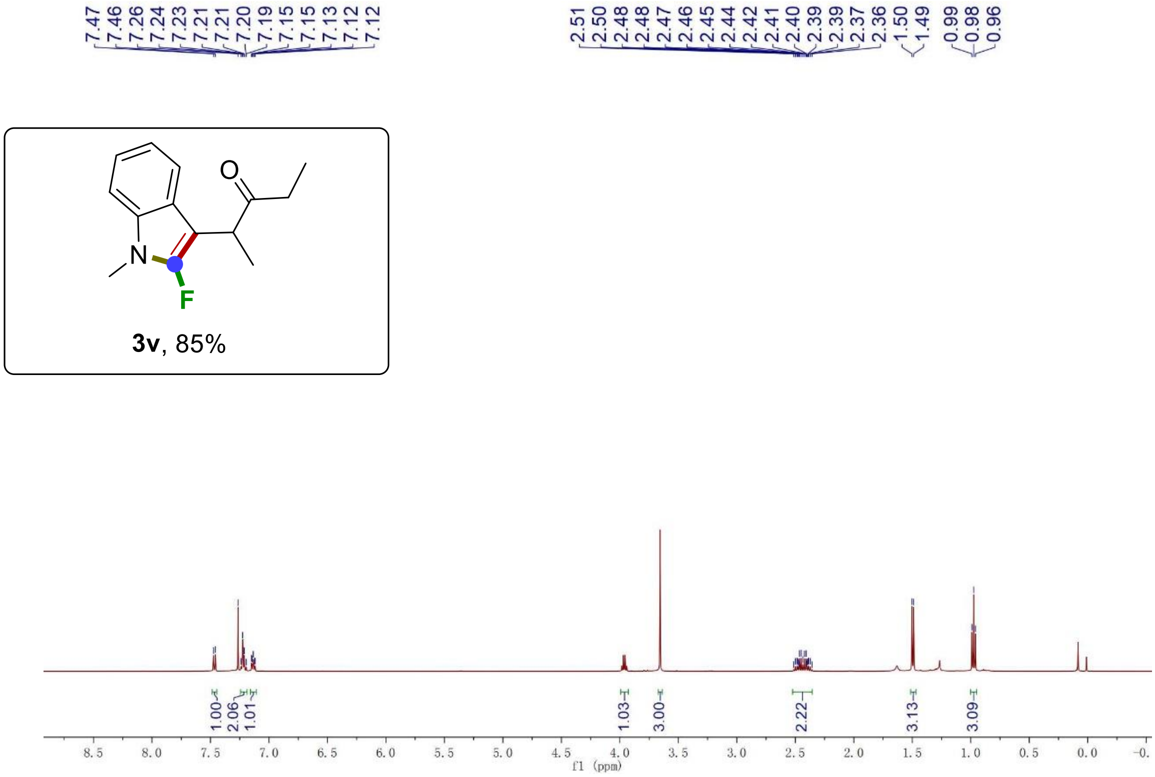

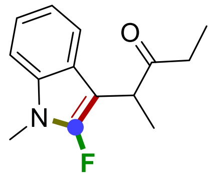


**3v**, 85%

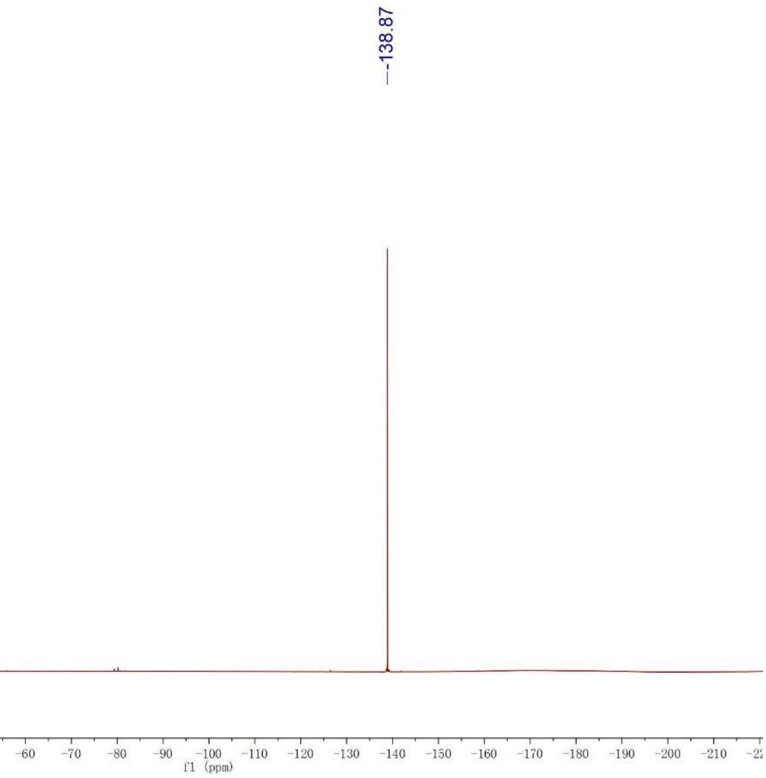


Supplementary Figure 114. $^{1}$H NMR, $^{13}$C NMR and $^{19}$F NMR spectrum of **3v**

**2-(2-fluoro-1-methyl-1H-indol-3-yl)-4-methylpentan-3-one (3w)**

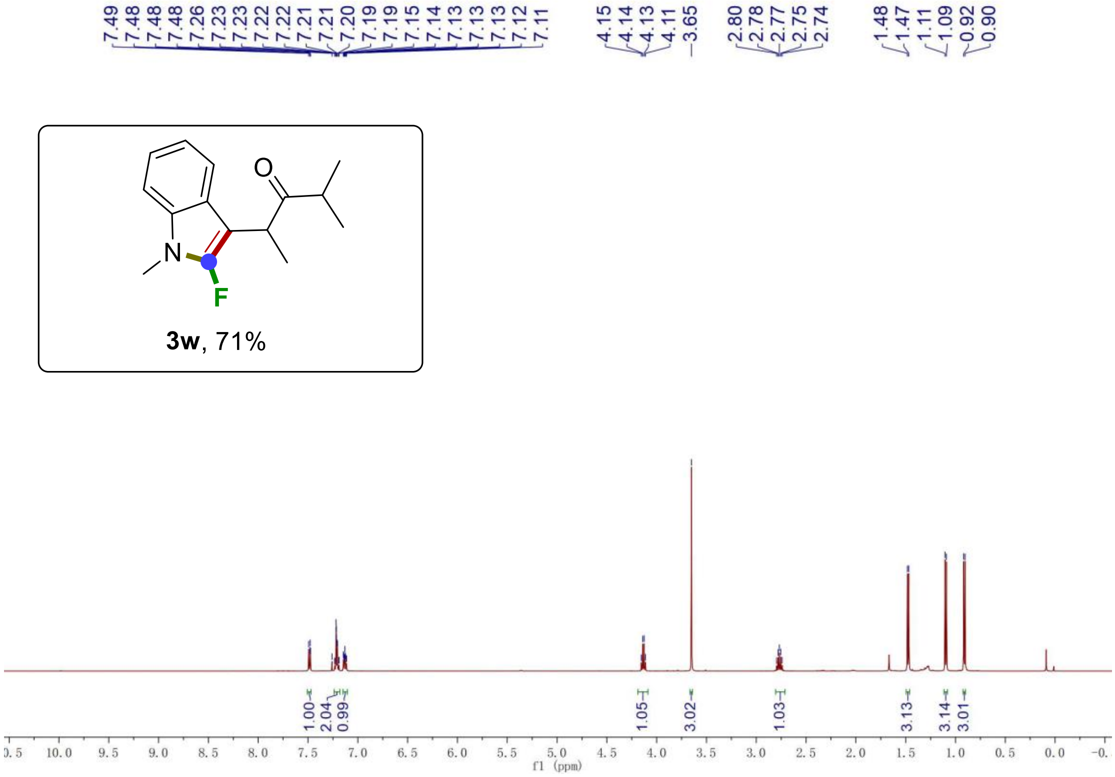

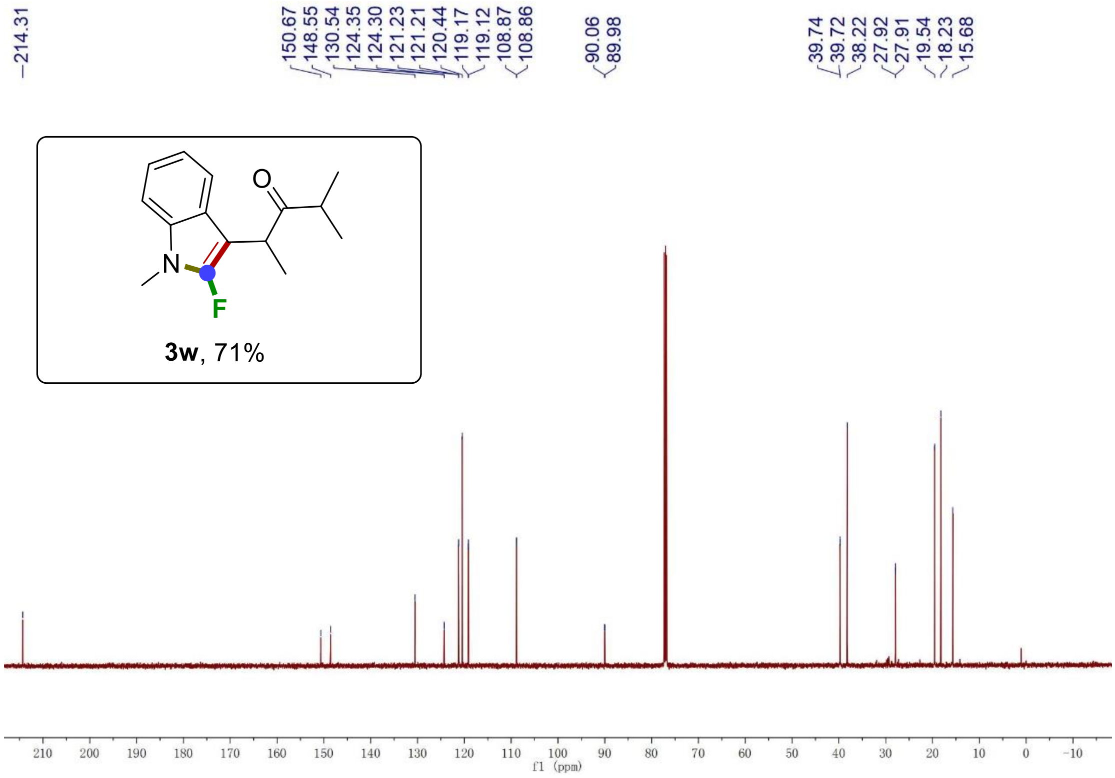

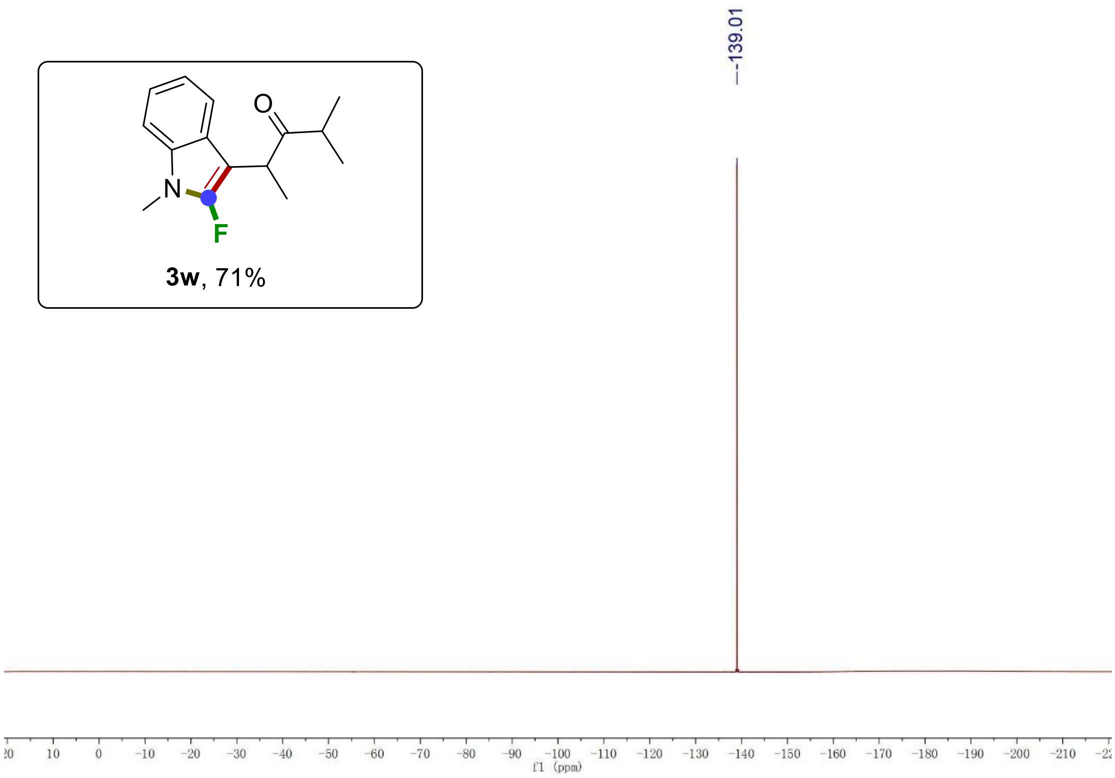


Supplementary Figure 115. $^{1}H$ NMR, $^{13}C$ NMR and $^{19}F$ NMR spectrum of **3w**.

## 1-(2-fluoro-1-methyl-1H-indol-3-yl)nonan-2-one (3x)

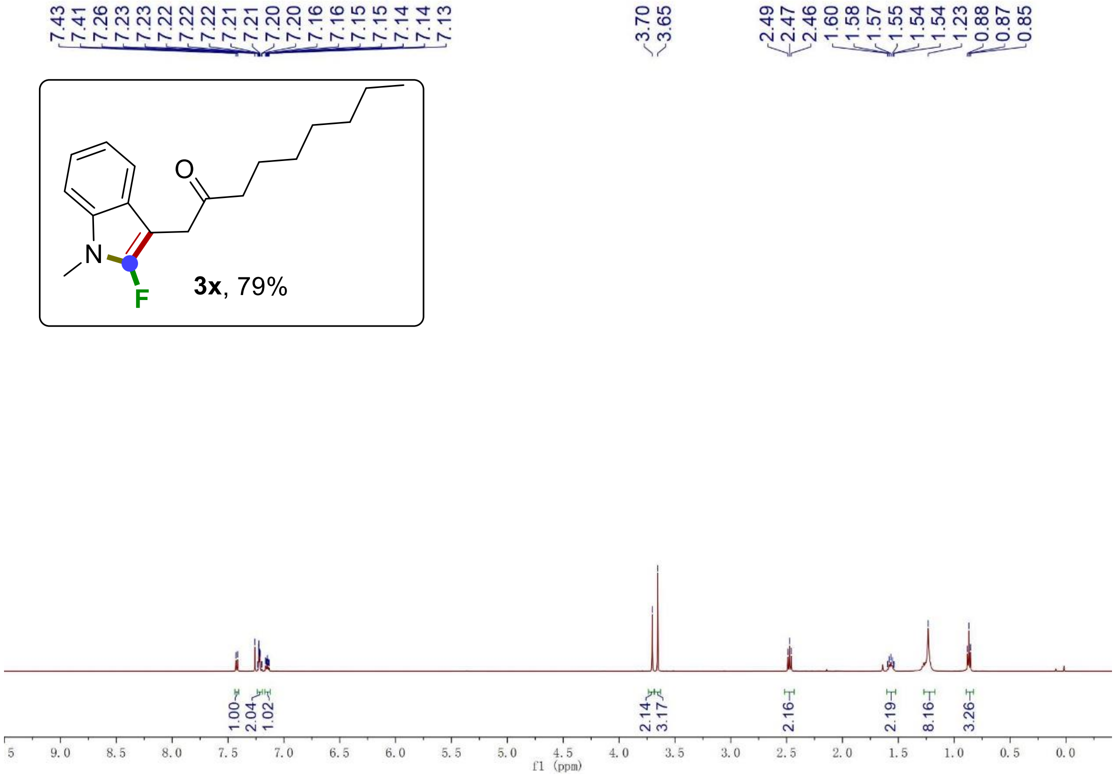


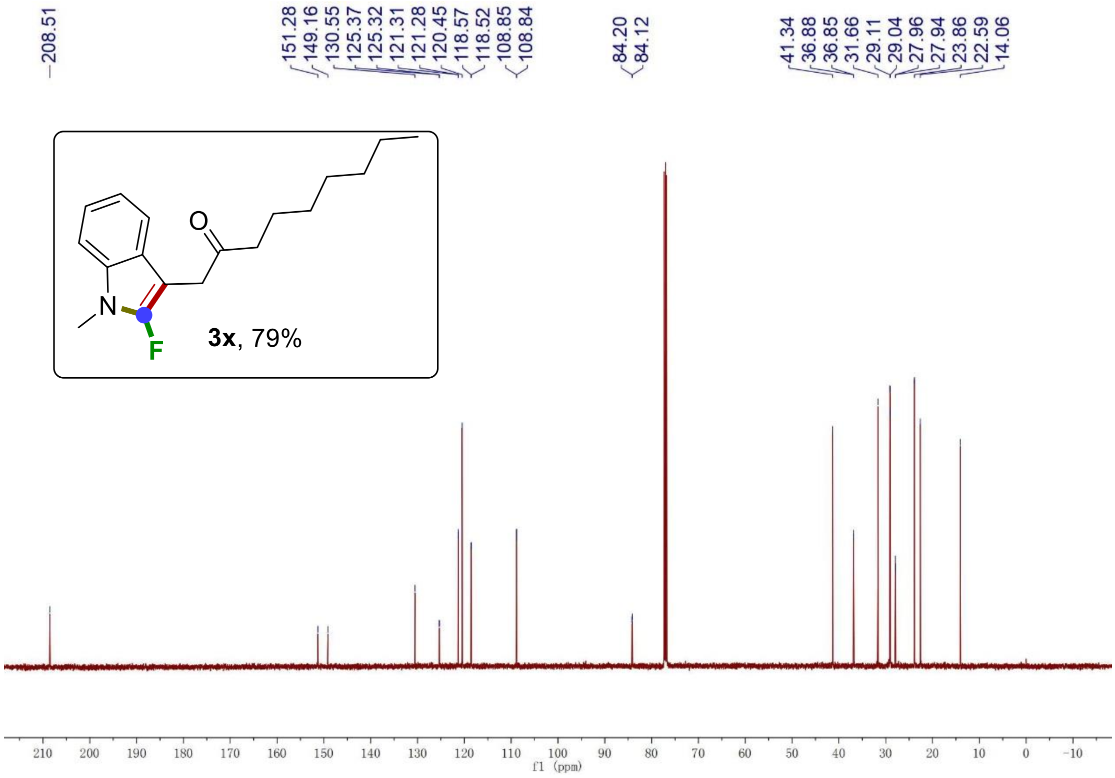

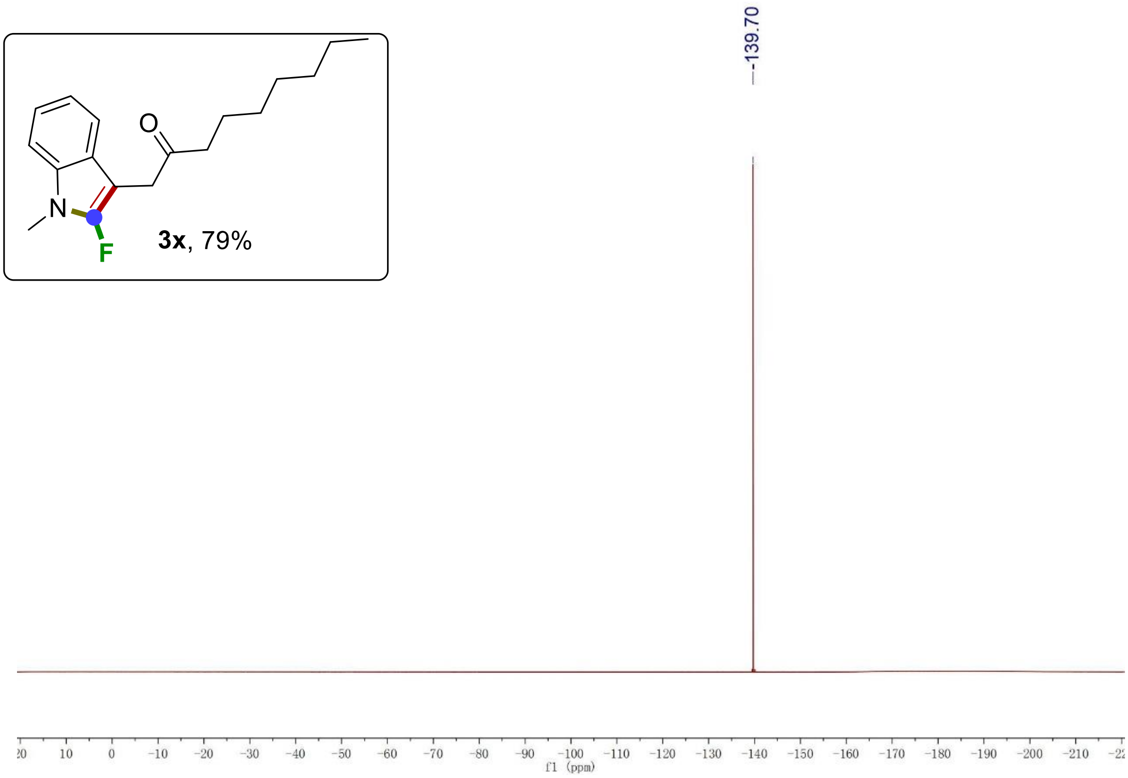


Supplementary Figure 116. $^{1}H$ NMR, $^{13}C$ NMR and $^{19}F$ NMR spectrum of **3x**.

**cyclopropyl-2-(2-fluoro-1-methyl-1H-indol-3-yl)ethan-1-one (3y)**

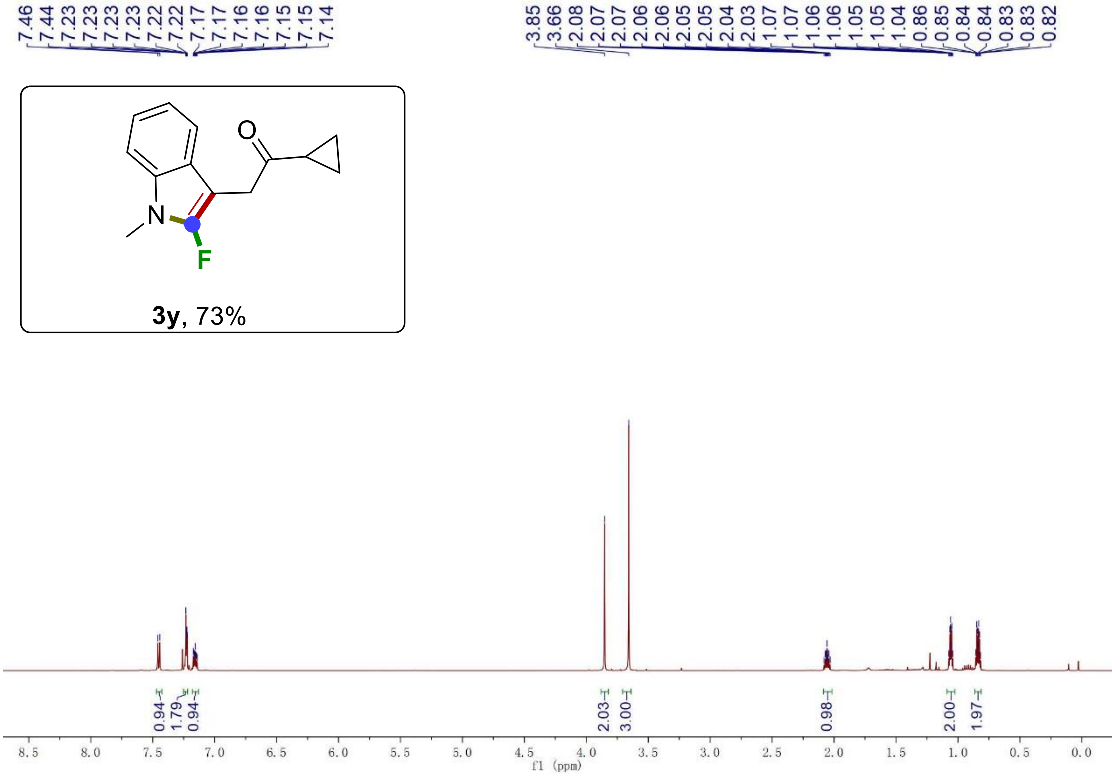


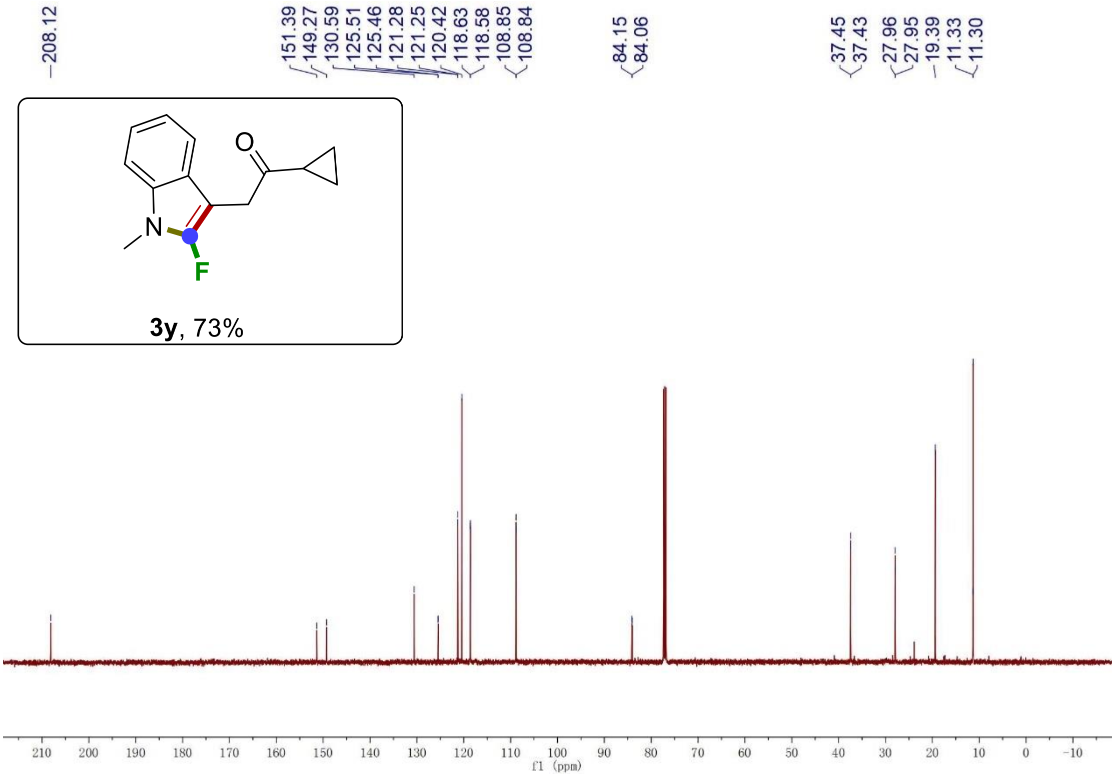

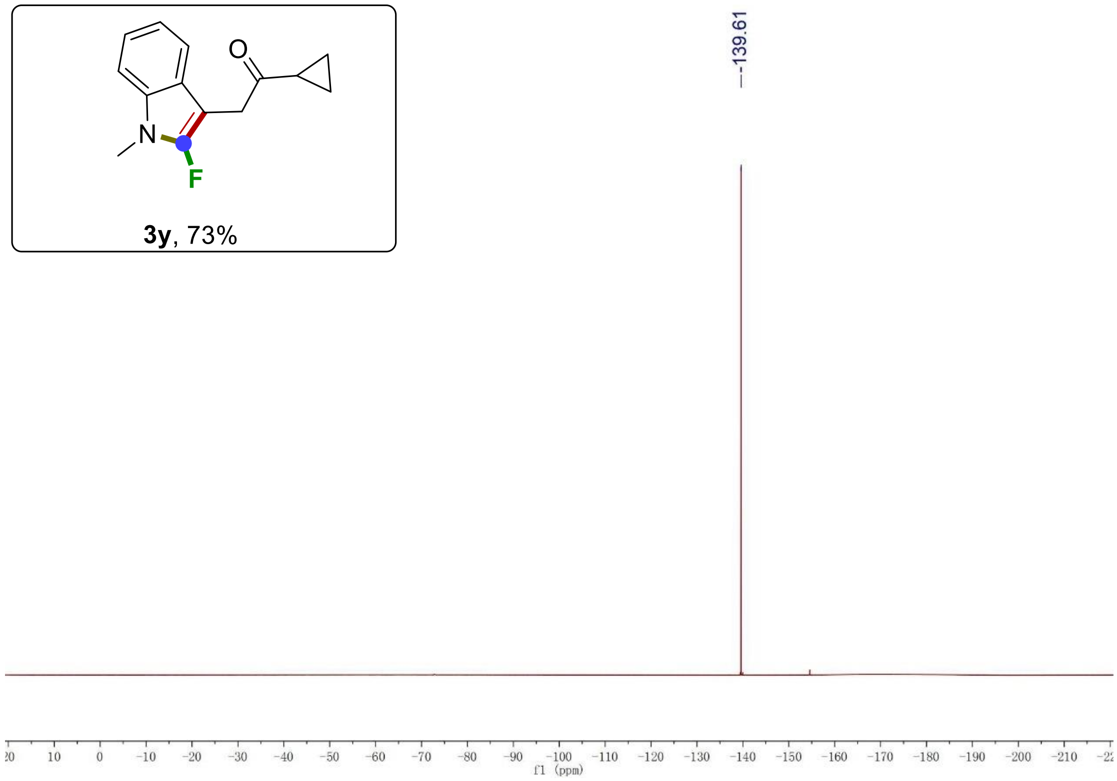


Supplementary Figure 117. $^{1}H$ NMR, $^{13}C$ NMR and $^{19}F$ NMR spectrum of **3y**.

## 1-cyclohexyl-2-(2-fluoro-1-methyl-1H-indol-3-yl)ethan-1-one (3z)

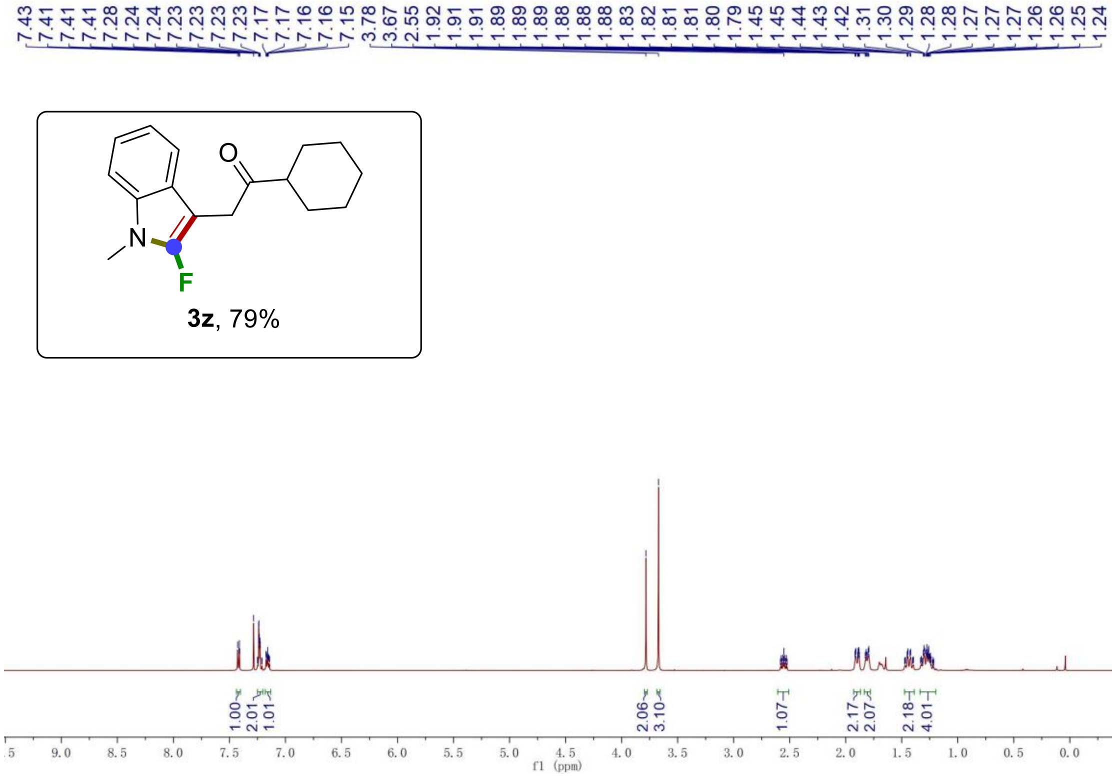


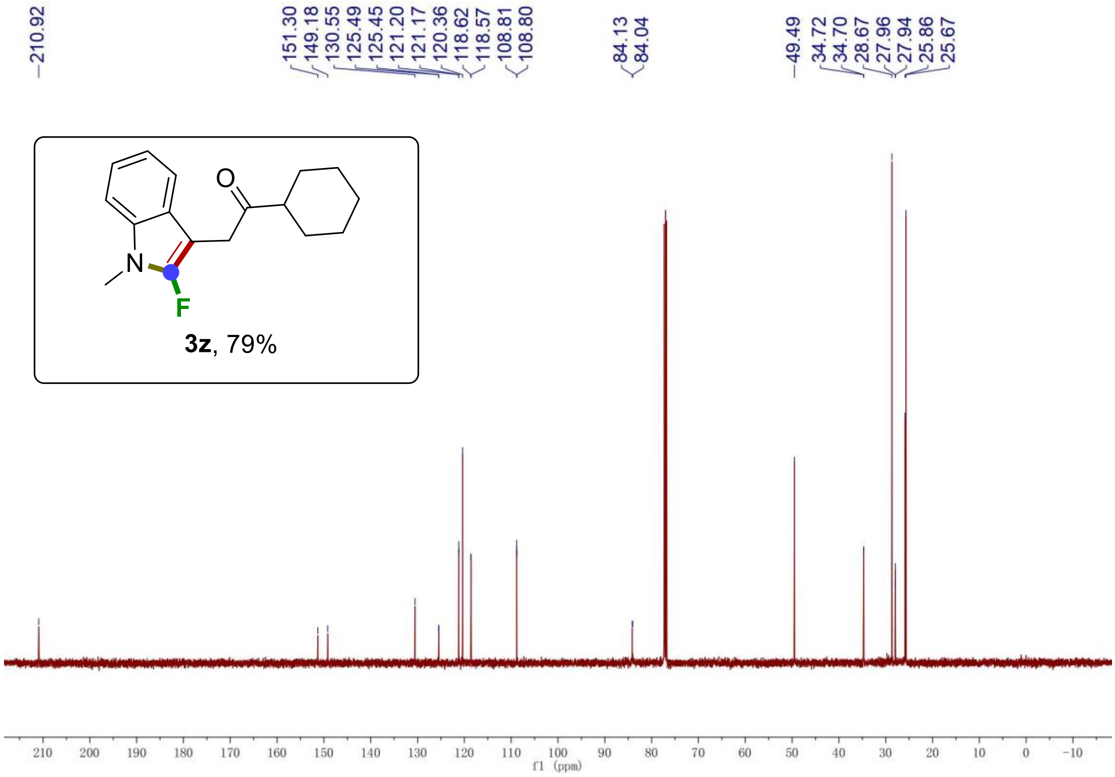

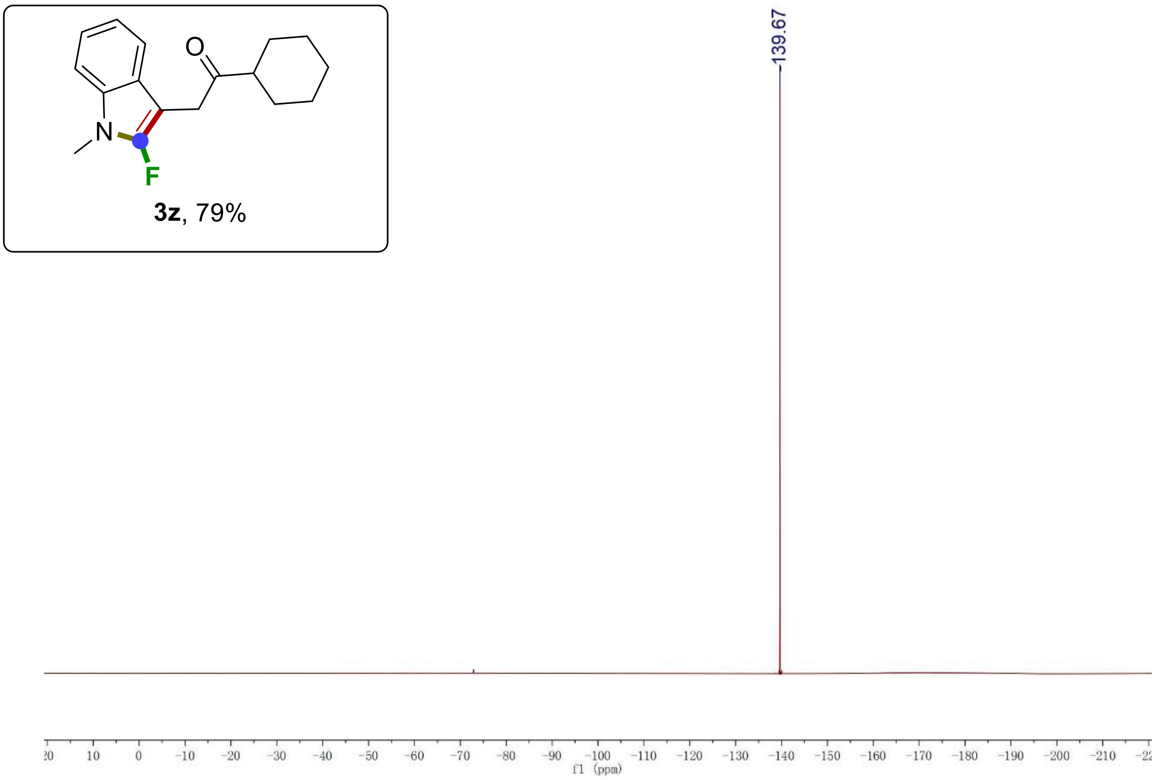


Supplementary Figure 118. $^{1}H$ NMR, $^{13}C$ NMR and $^{19}F$ NMR spectrum of **3z**.

**1-((1S,4S)-bicyclo[2.2.1]hept-5-en-2-yl)-2-(2-fluoro-1-methyl-1H-indol-3-yl)ethan-1-one (3aa)**

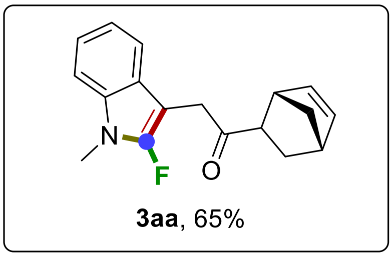

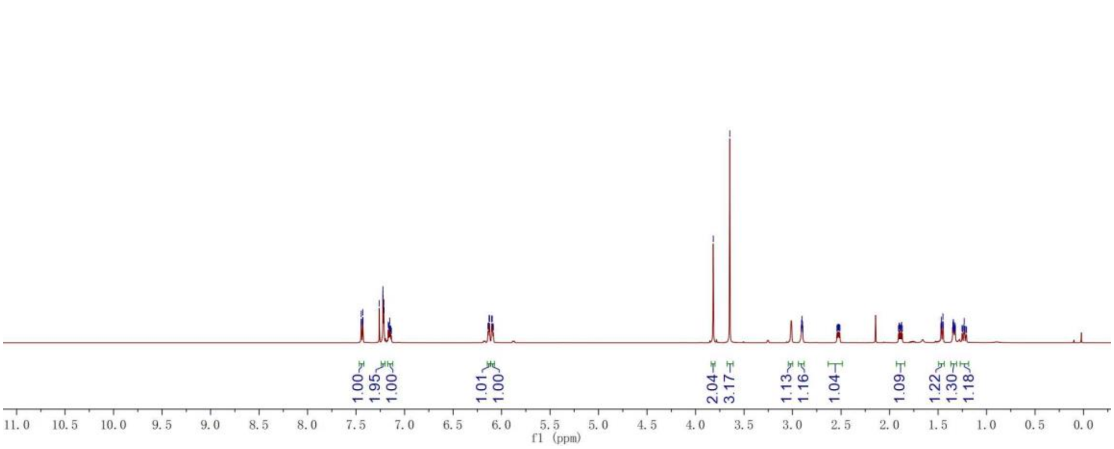

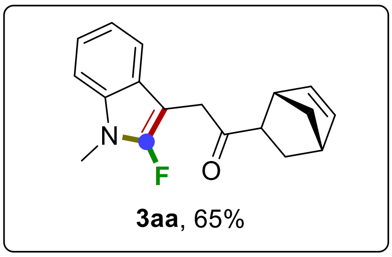

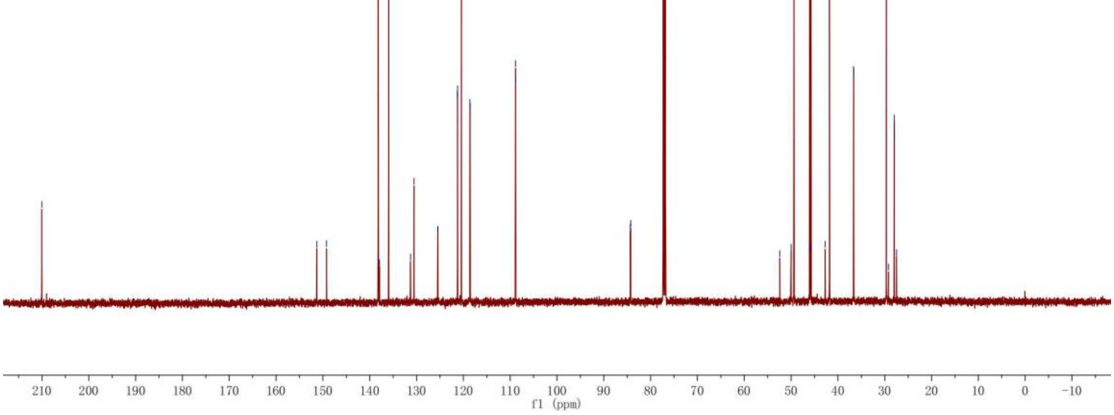

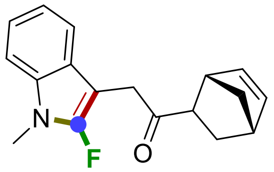

3aa, 65%

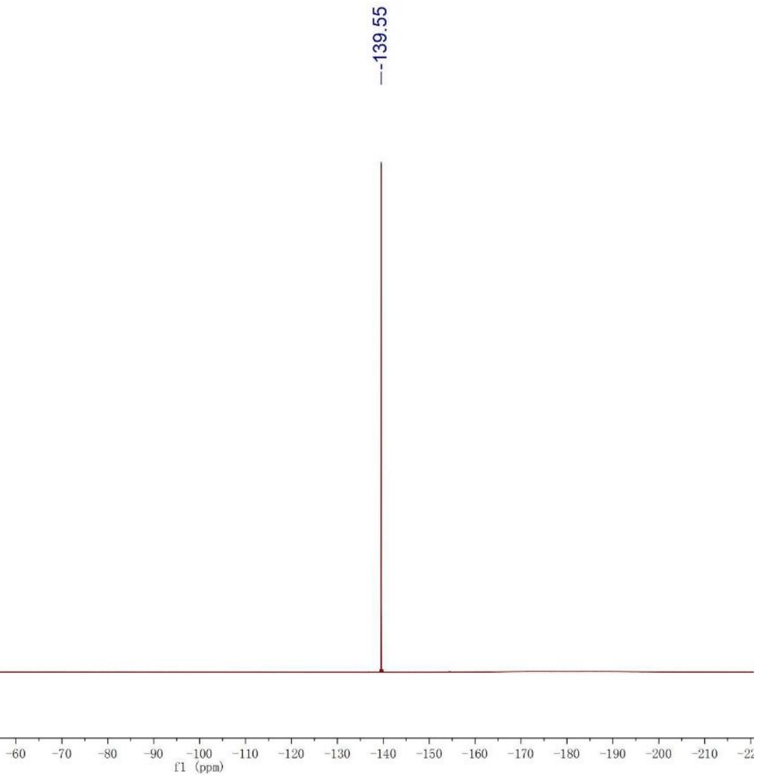


Supplementary Figure 119. $^{1}H$ NMR, $^{13}C$ NMR and $^{19}F$ NMR spectrum of **3aa**.

## 2-(2-fluoro-1-methyl-1H-indol-3-yl)cyclobutan-1-one (3ab)

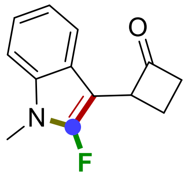


**3ab**, 52%

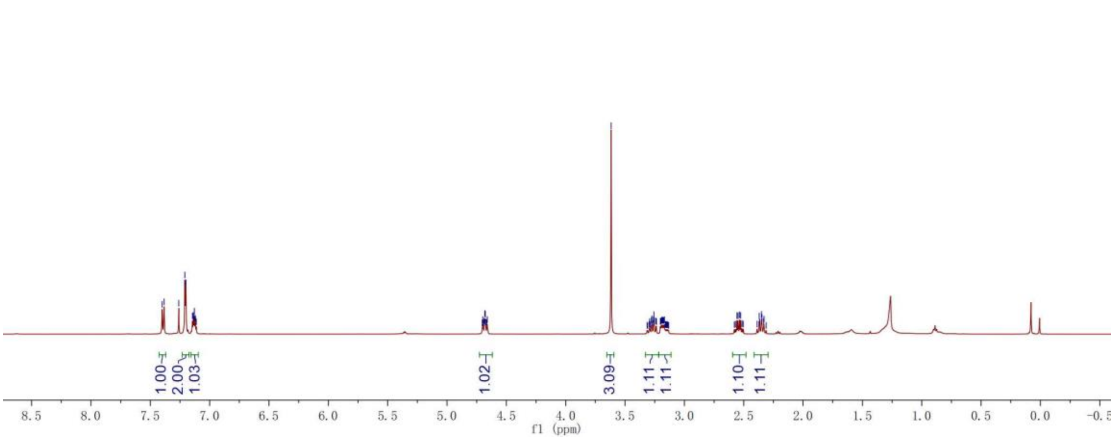


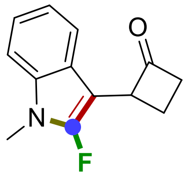


**3ab**, 52%

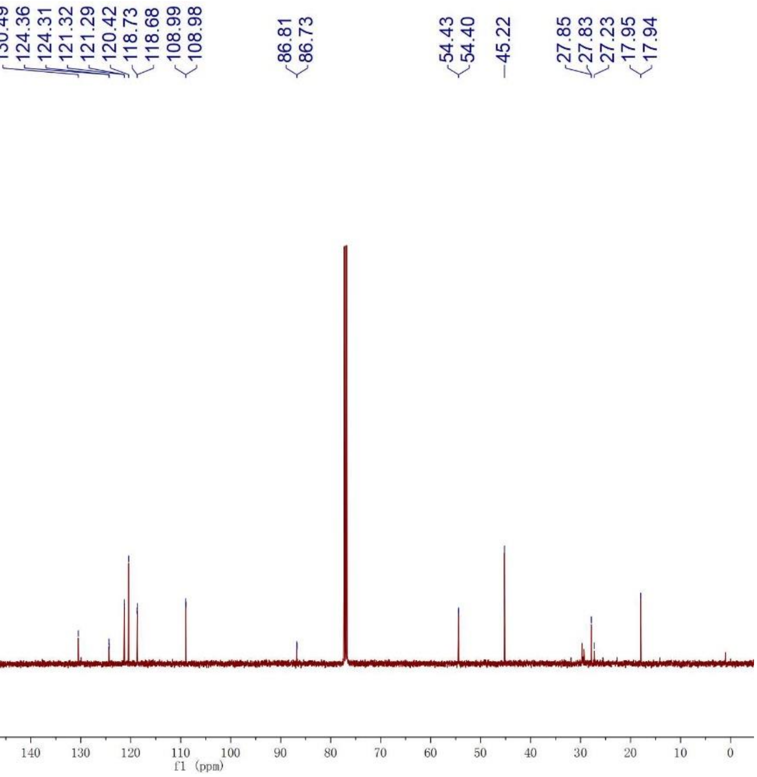

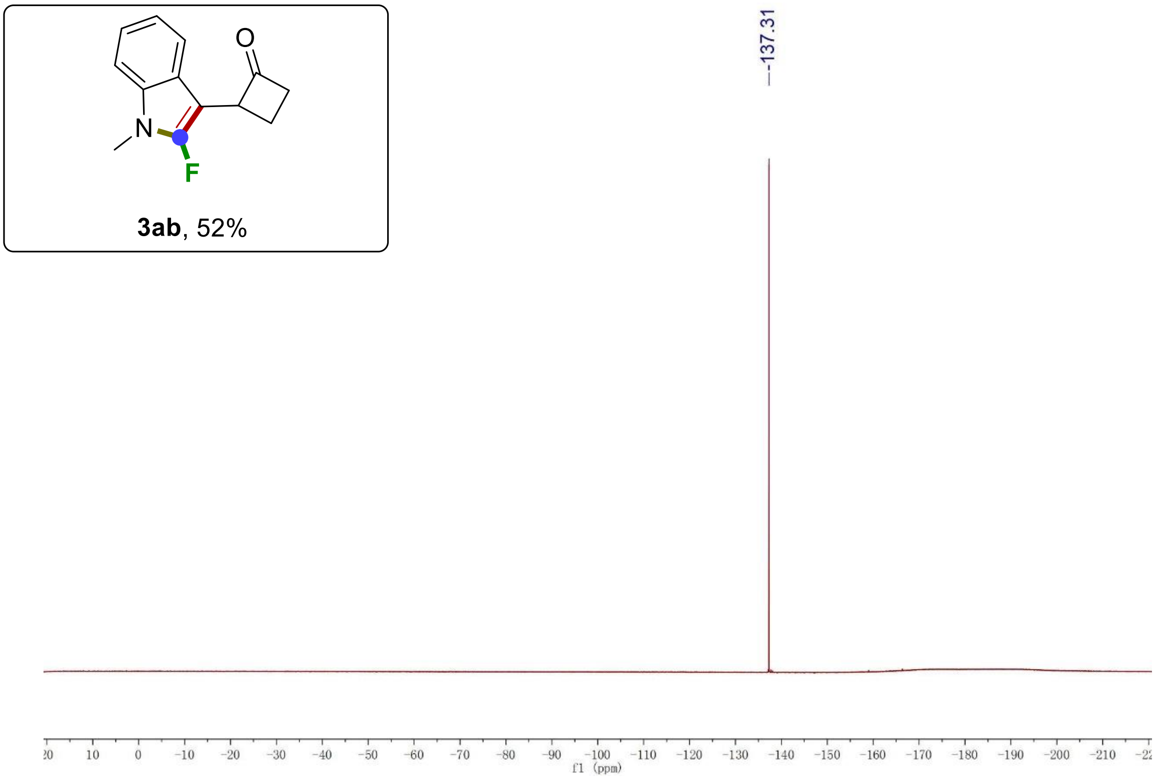


Supplementary Figure 120. $^{1}$H NMR, $^{13}$C NMR and $^{19}$F NMR spectrum of **3ab**.

## 3-(2-fluoro-1-methyl-1H-indol-3-yl)tetrahydro-4H-pyran-4-one (3ac)

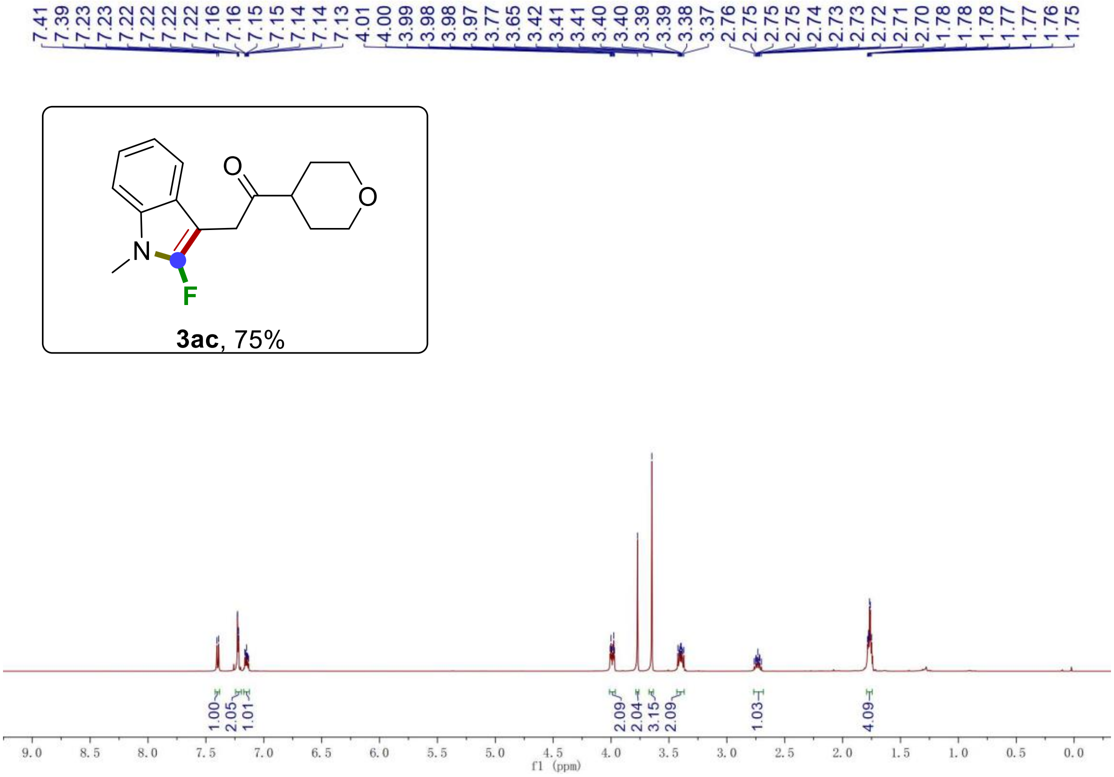


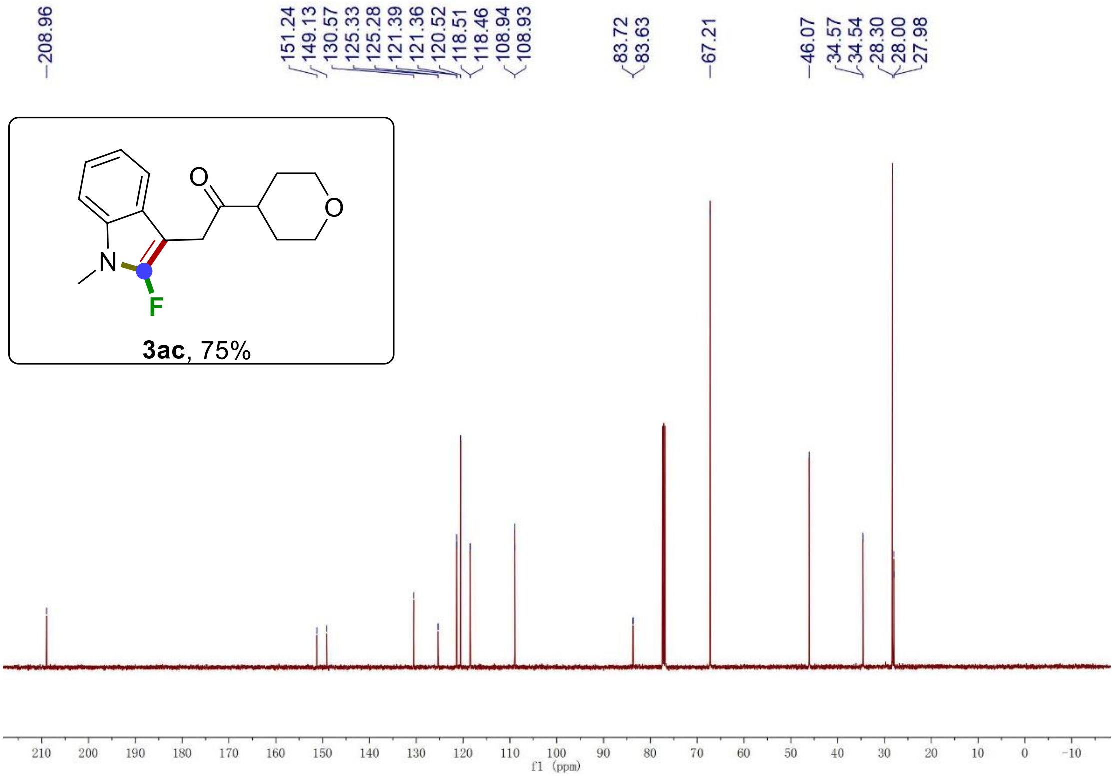

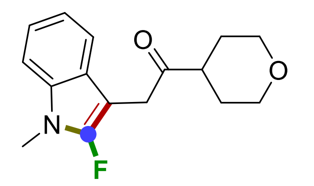


**3ac**, 75%

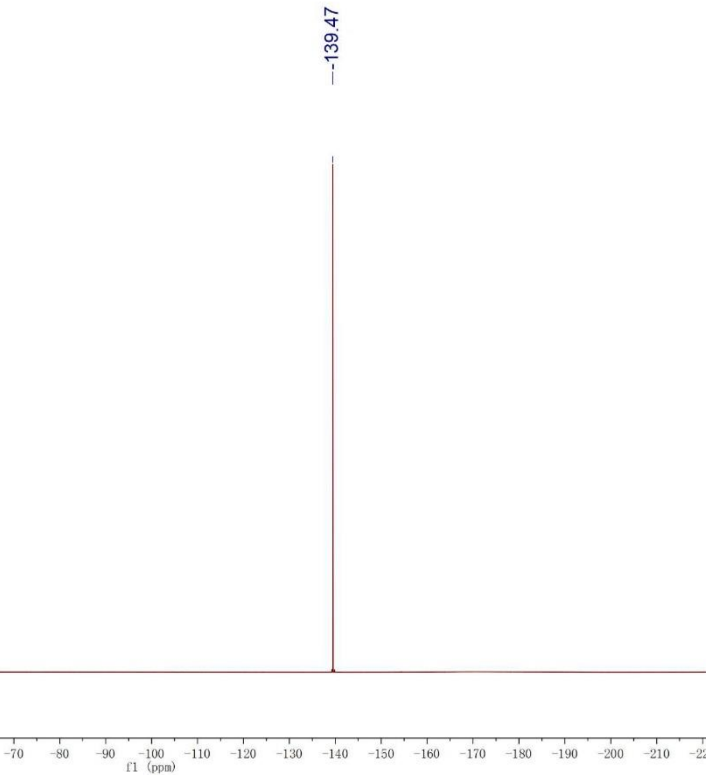


Supplementary Figure 121. $^{1}H$ NMR, $^{13}C$ NMR and $^{19}F$ NMR spectrum of **3ac**.

**2-(2-fluoro-1-methyl-1H-indol-3-yl)cyclooctan-1-one (3ad)**

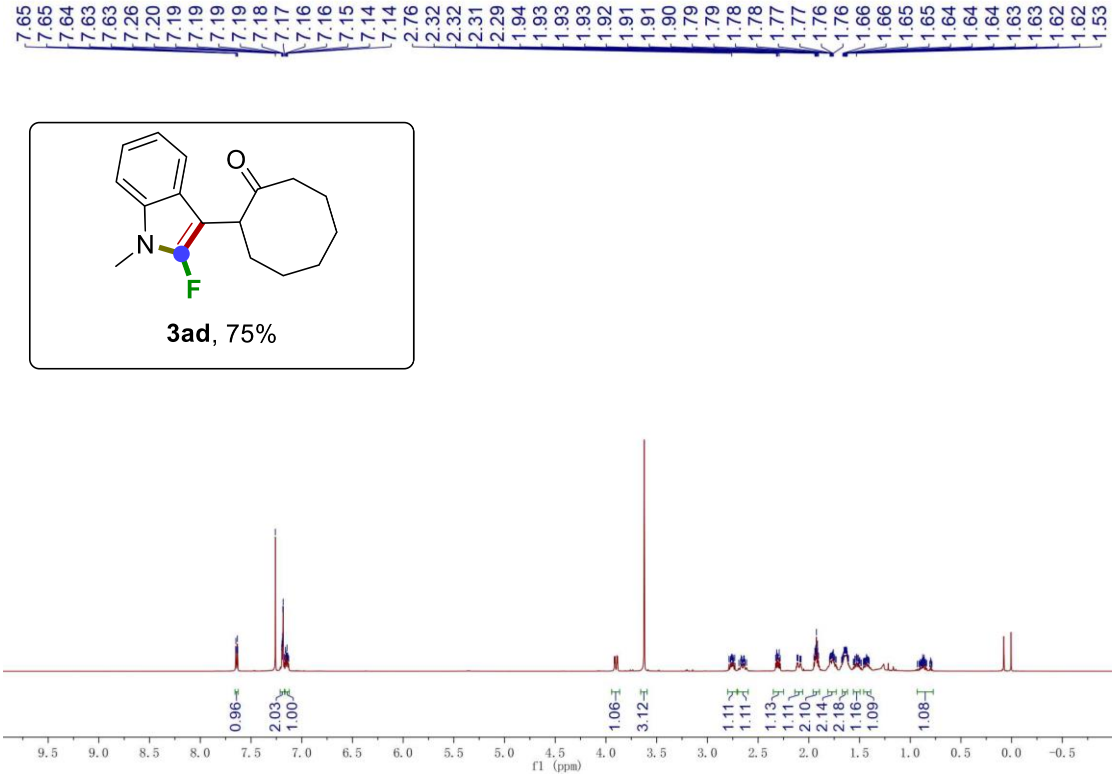


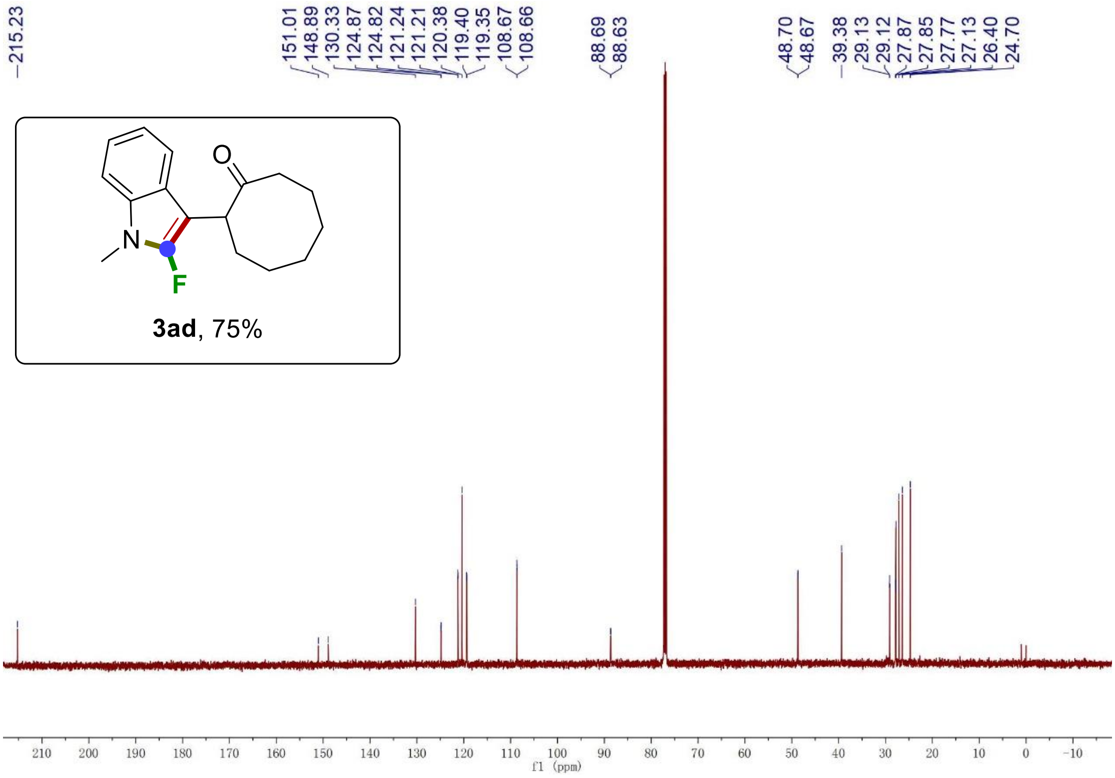

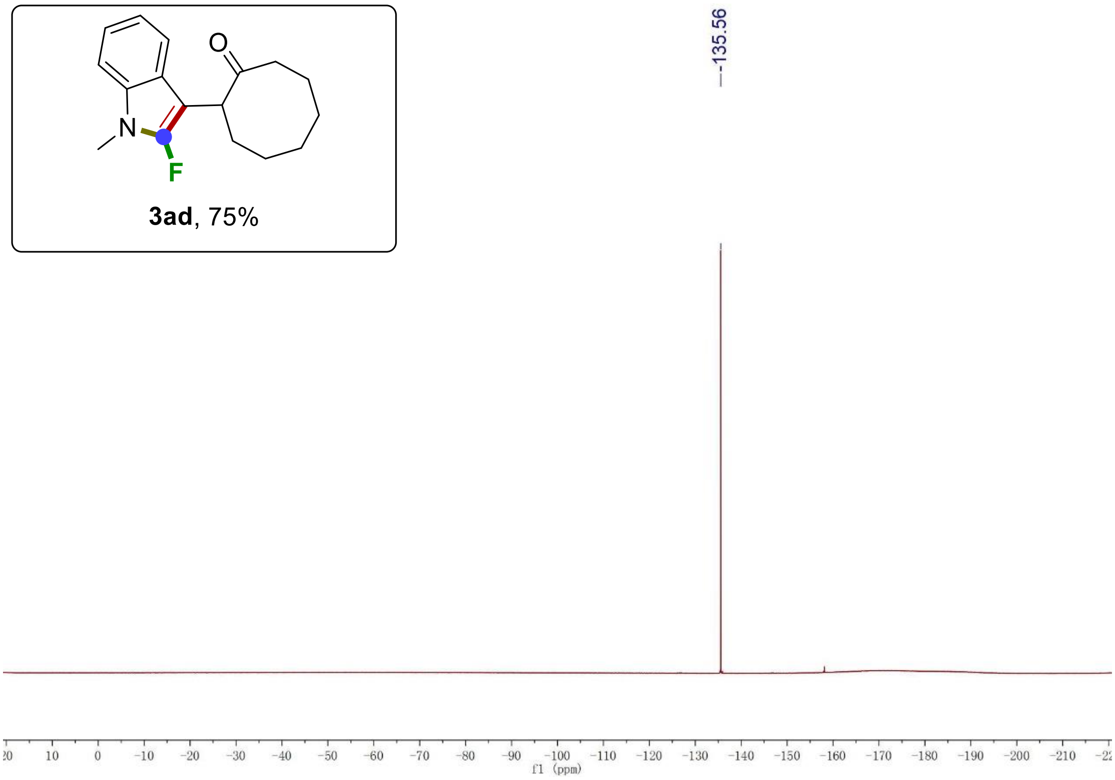


Supplementary Figure 122. $^{1}H$ NMR, $^{13}C$ NMR and $^{19}F$ NMR spectrum of **3ad**.

**(1R,4S)-3-(2-fluoro-1-methyl-1H-indol-3-yl)bicyclo[2.2.1]heptan-2-one (3ae)**

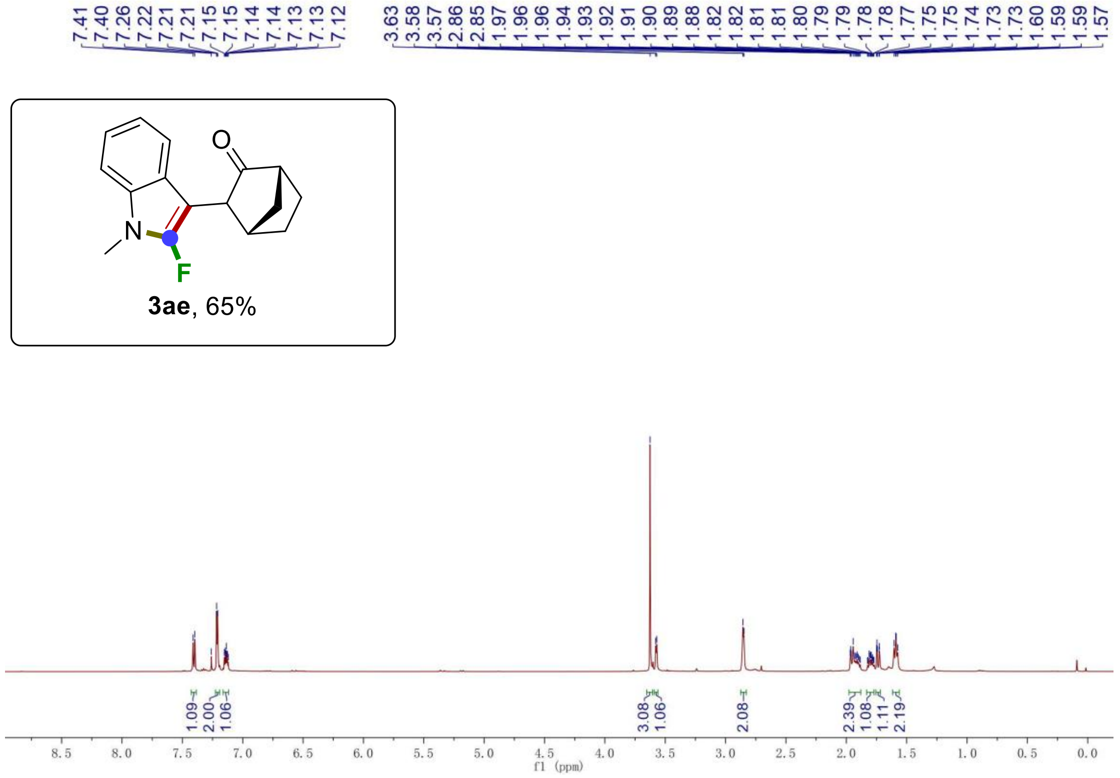


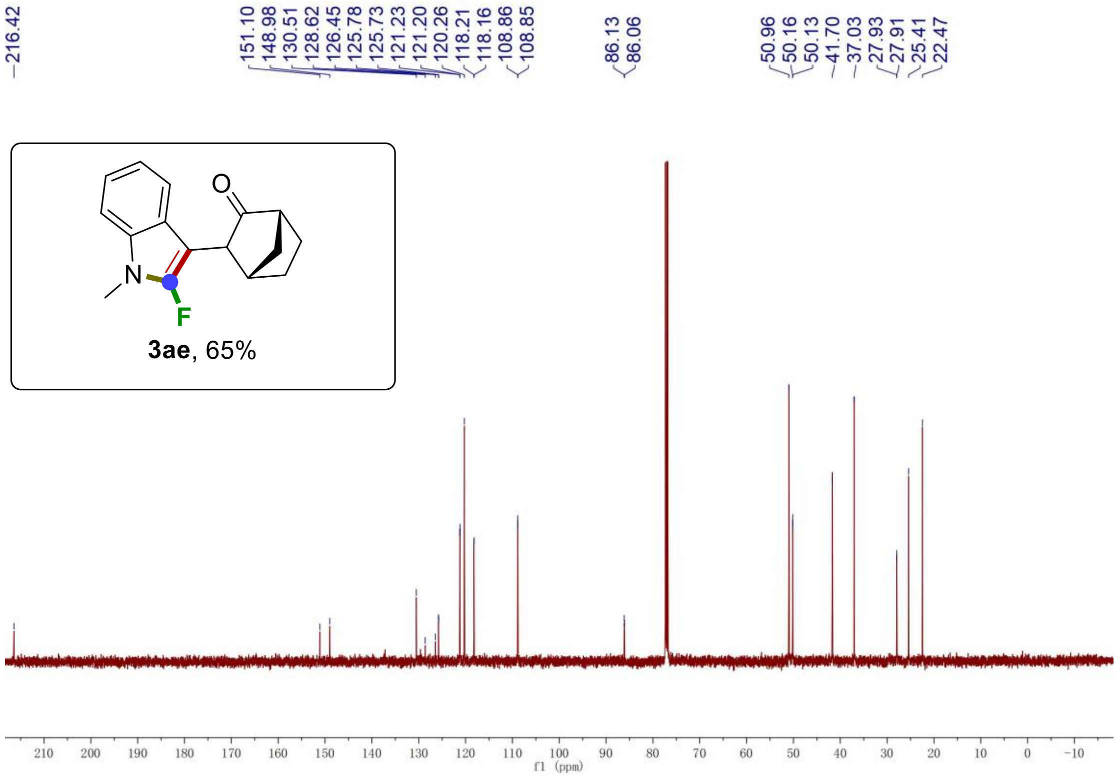

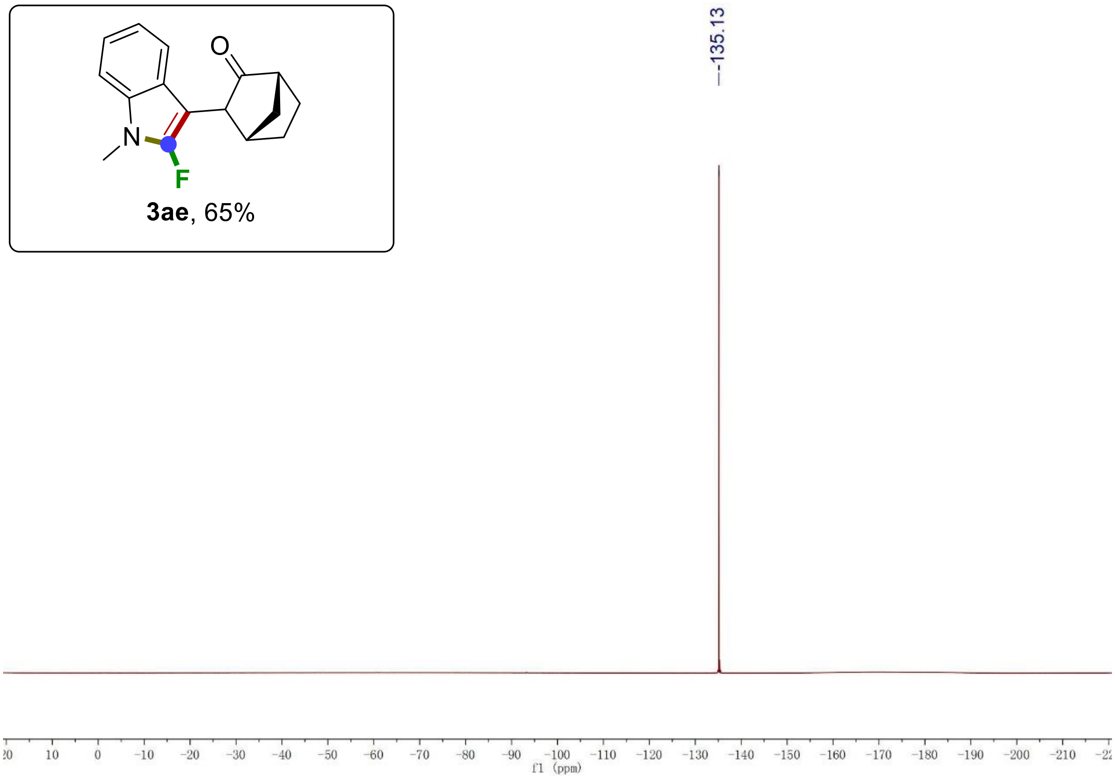


Supplementary Figure 123. $^{1}H$ NMR, $^{13}C$ NMR and $^{19}F$ NMR spectrum of **3ae**.

## 1,3-bis(2-fluoro-1-methyl-1H-indol-3-yl)propan-2-one (3af)

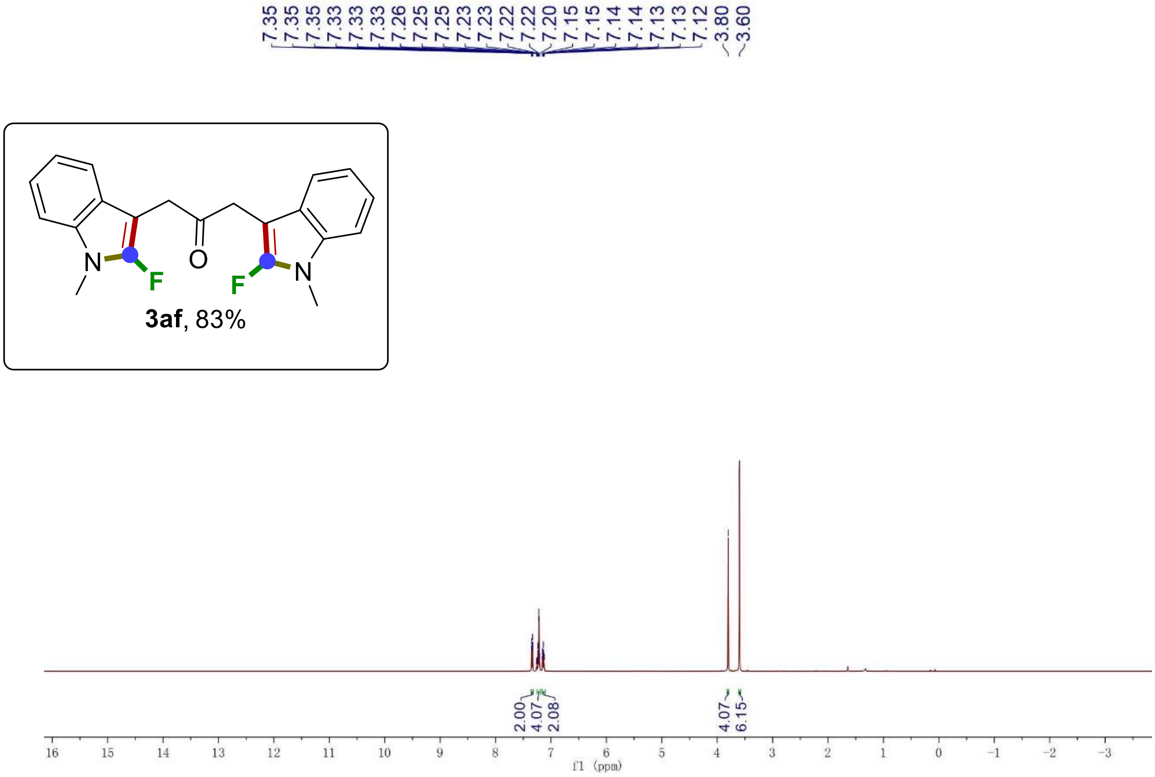


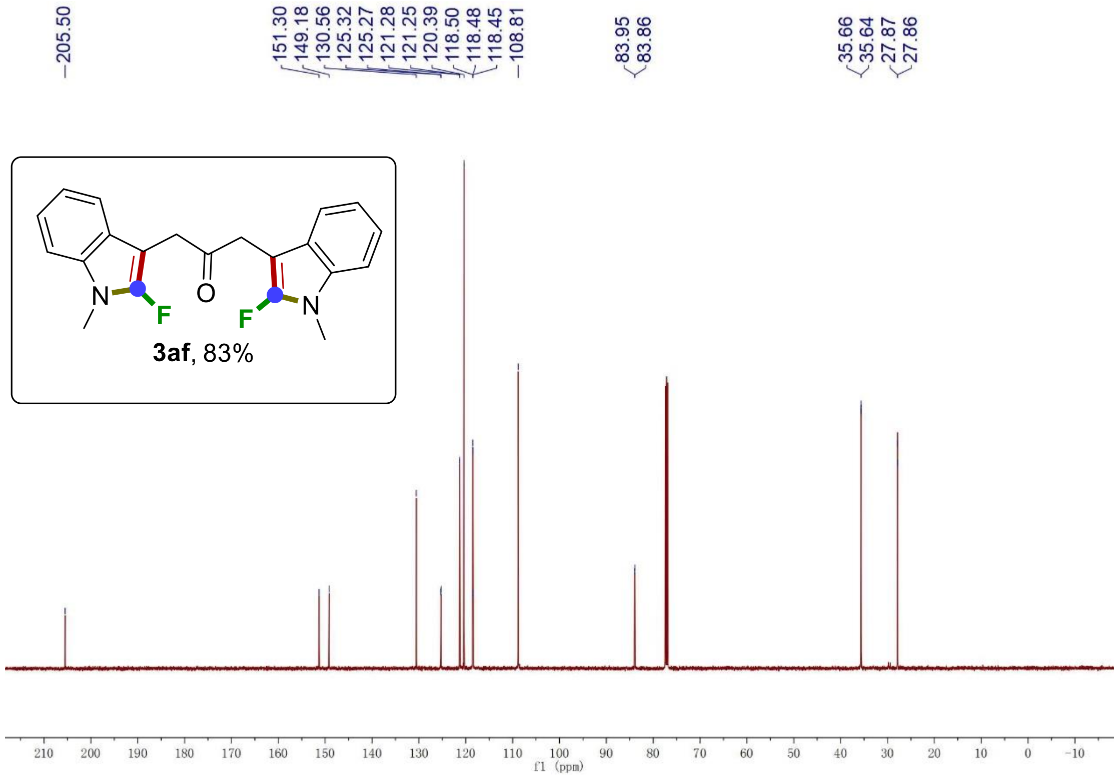

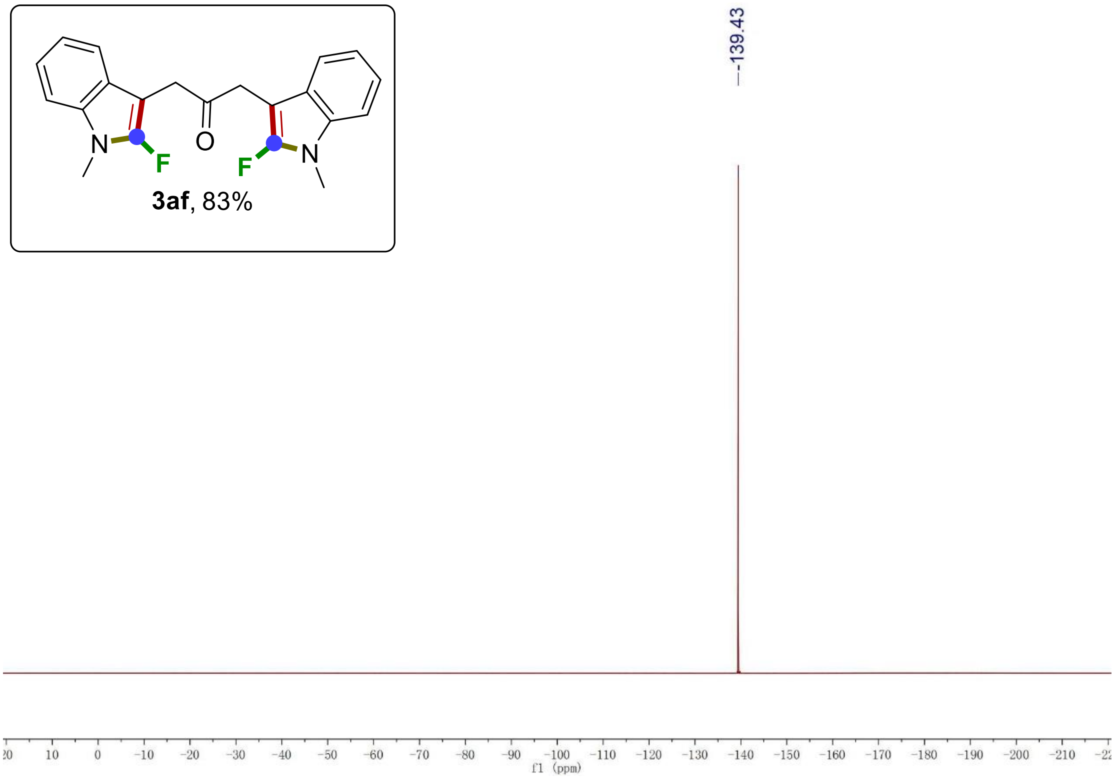


Supplementary Figure 124. $^{1}H$ NMR, $^{13}C$ NMR and $^{19}F$ NMR spectrum of **3af**.

# 2-(2-fluoro-1,5-dimethyl-1H-indol-3-yl)-1-phenylethan-1-one (3ag)

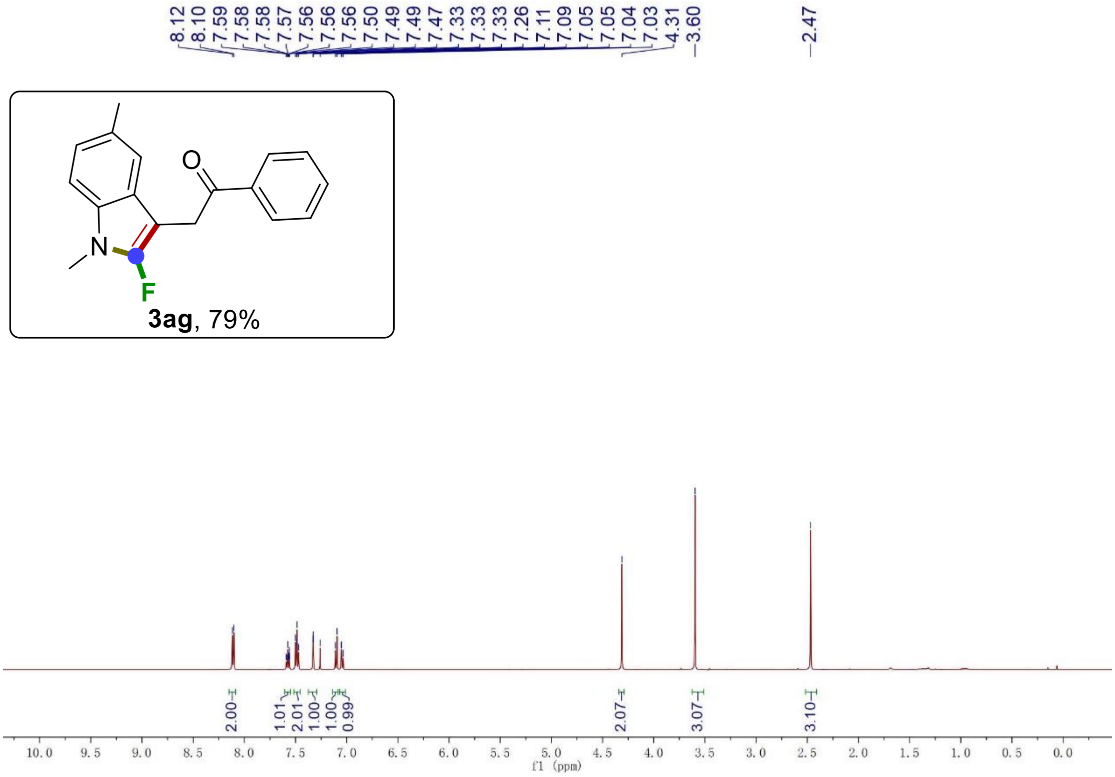


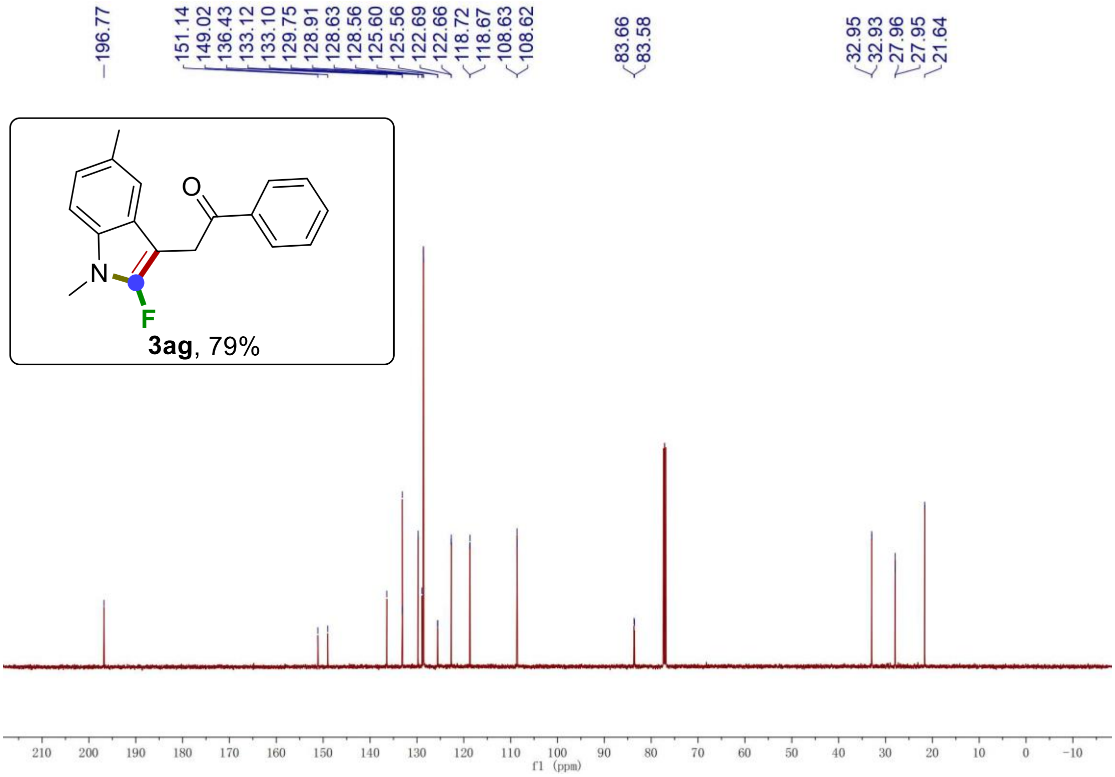

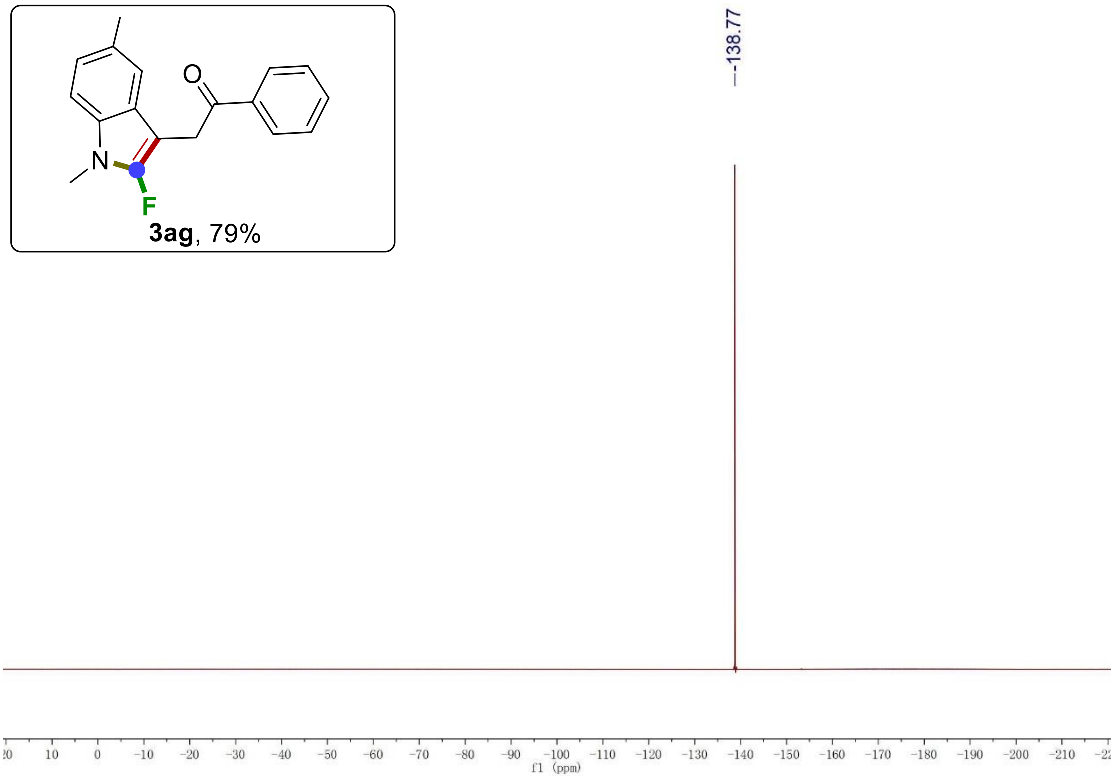


Supplementary Figure 125. $^{1}H$ NMR, $^{13}C$ NMR and $^{19}F$ NMR spectrum of **3ag**.

**2-(2-fluoro-1-methyl-5-(trifluoromethyl)-1H-indol-3-yl)-1-phenylethan-1-one (3ah)**

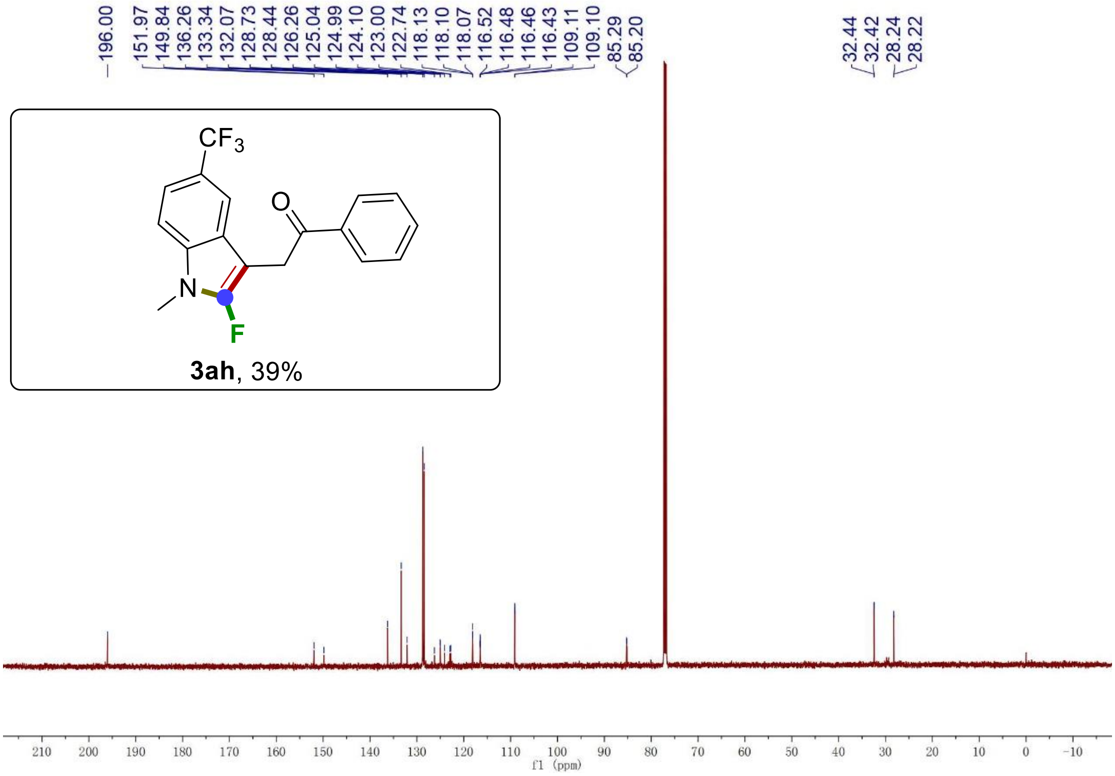


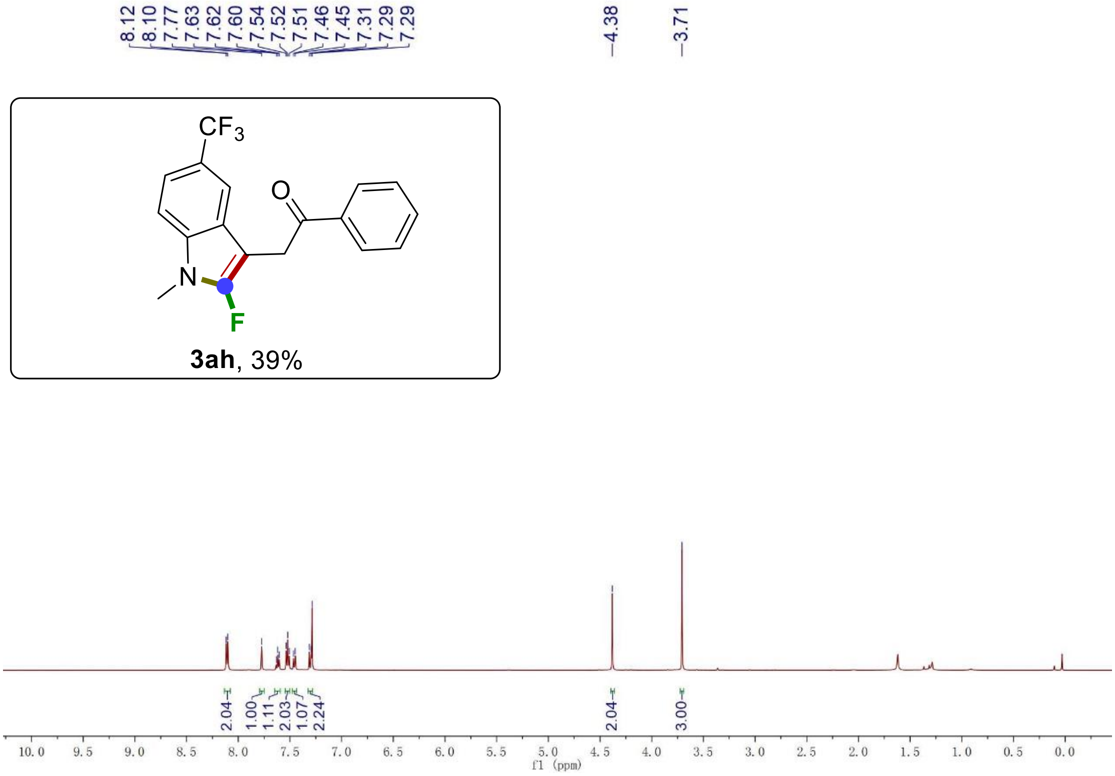

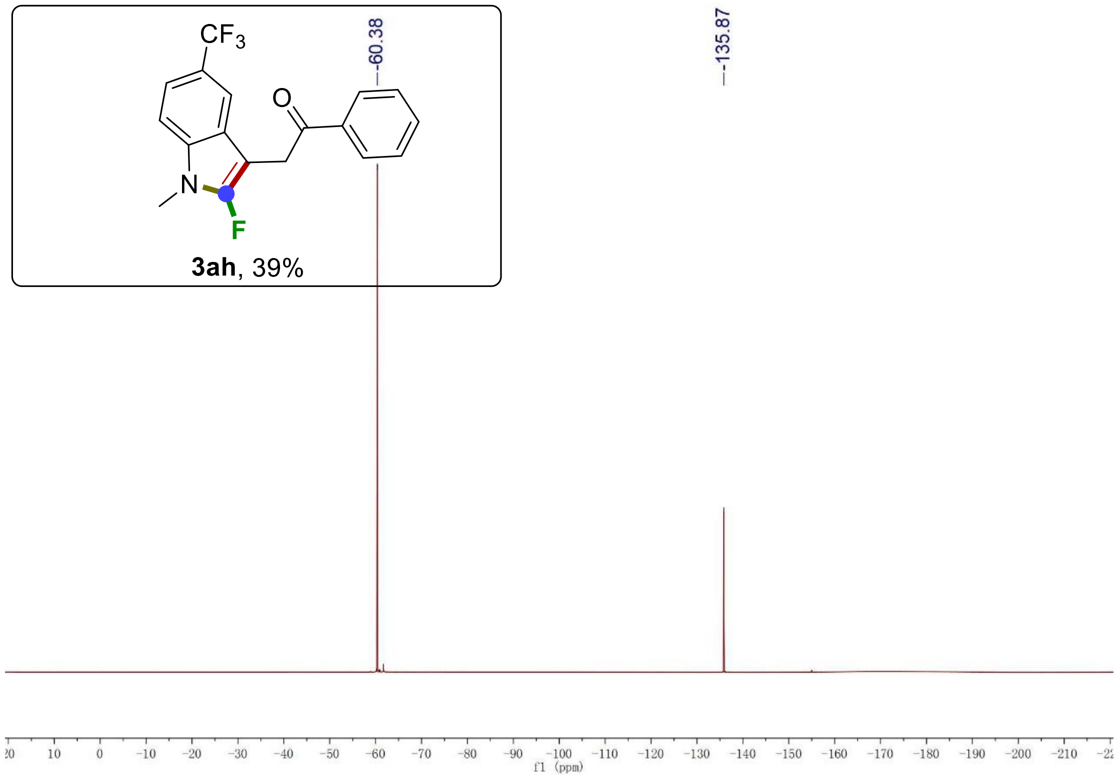


Supplementary Figure 126. $^{1}H$ NMR, $^{13}C$ NMR and $^{19}F$ NMR spectrum of **3ah**.

**2-(5-bromo-2-fluoro-1-methyl-1H-indol-3-yl)-1-phenylethan-1-one (3ai)**

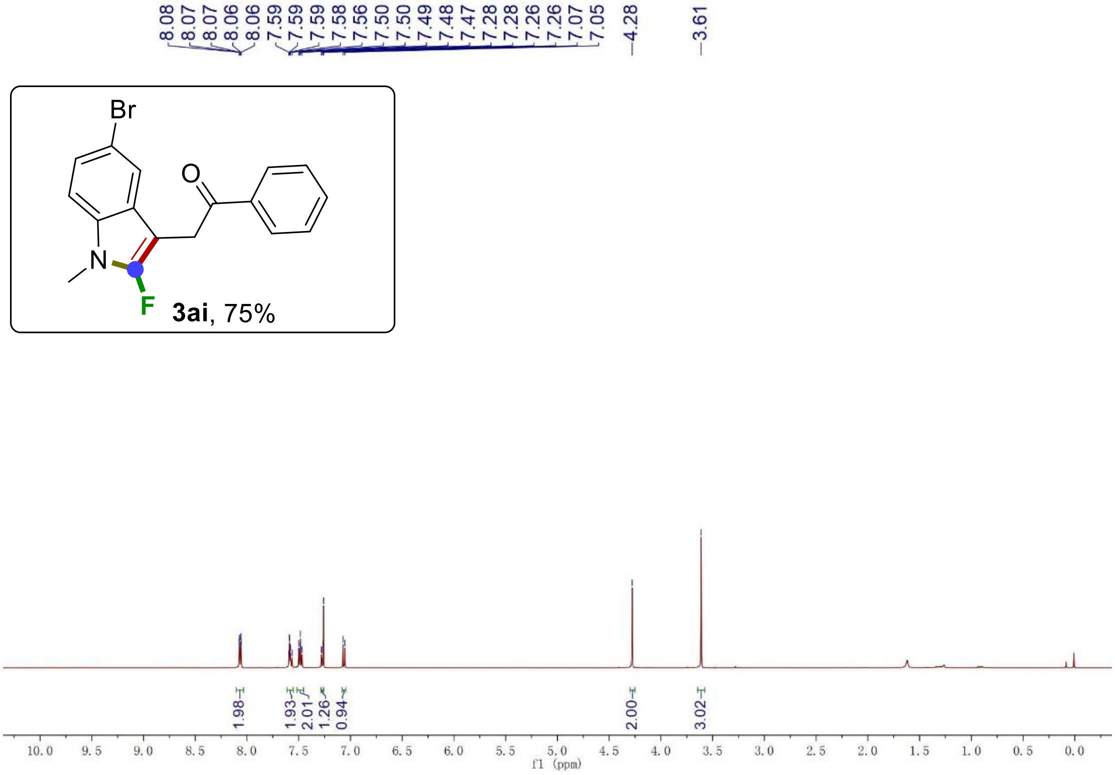


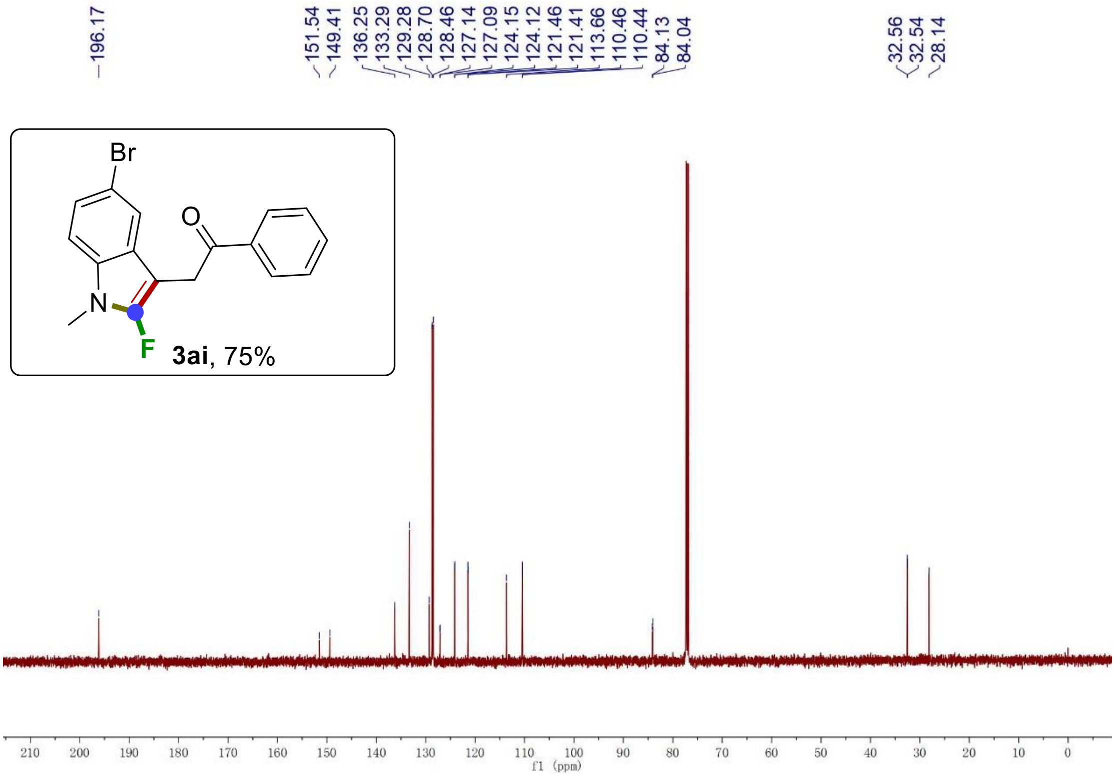

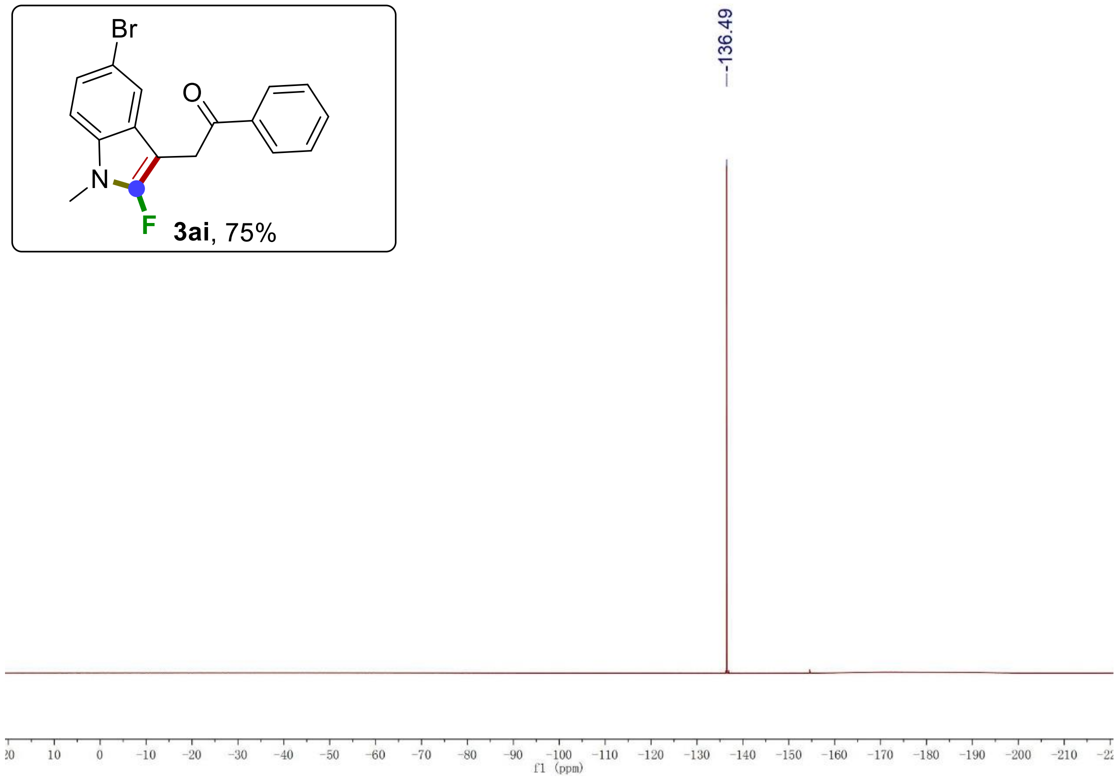


Supplementary Figure 127. $^{1}H$ NMR, $^{13}C$ NMR and $^{19}F$ NMR spectrum of **3ai**.

**2-(2-fluoro-1,6-dimethyl-1H-indol-3-yl)-1-phenylethan-1-one (3aj)**

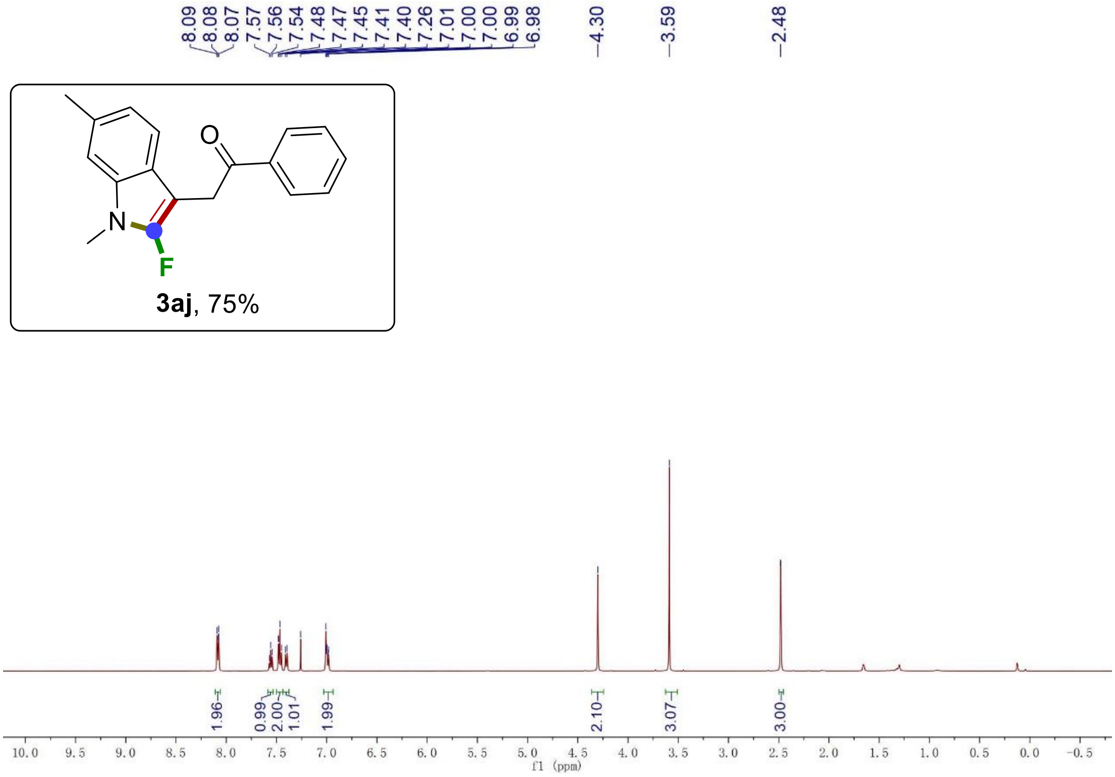


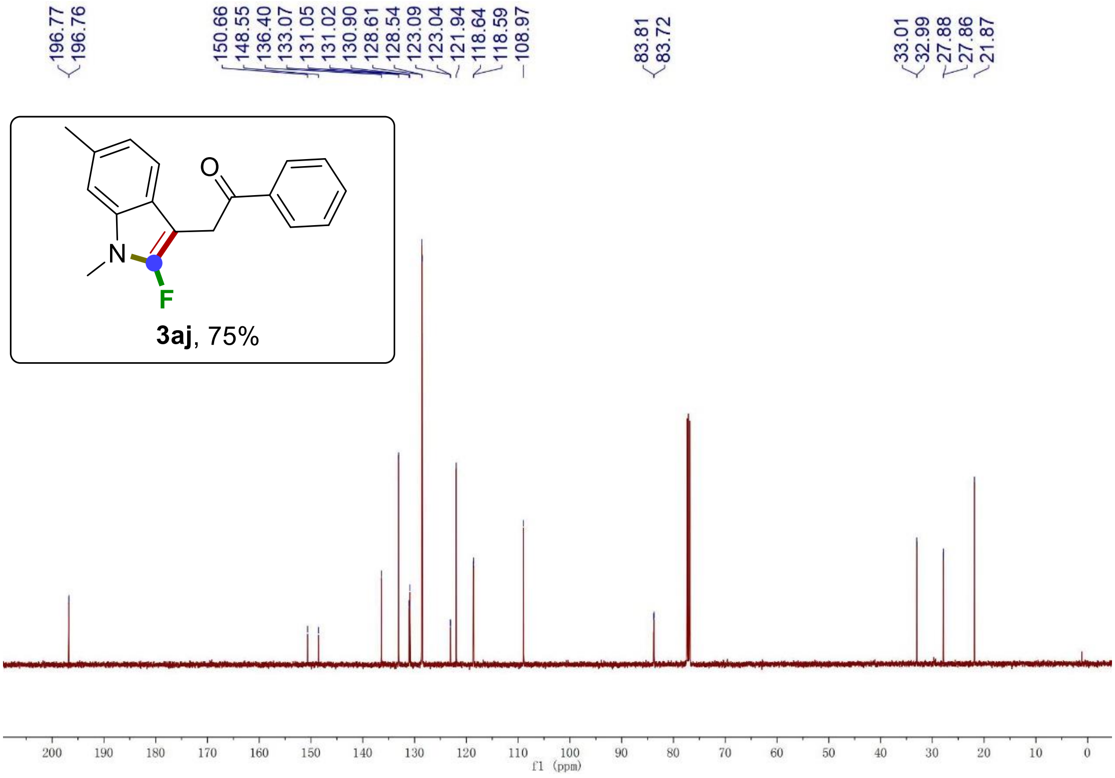

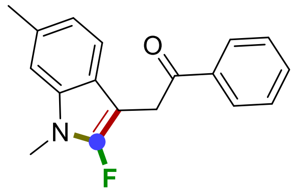


**3aj**, 75%

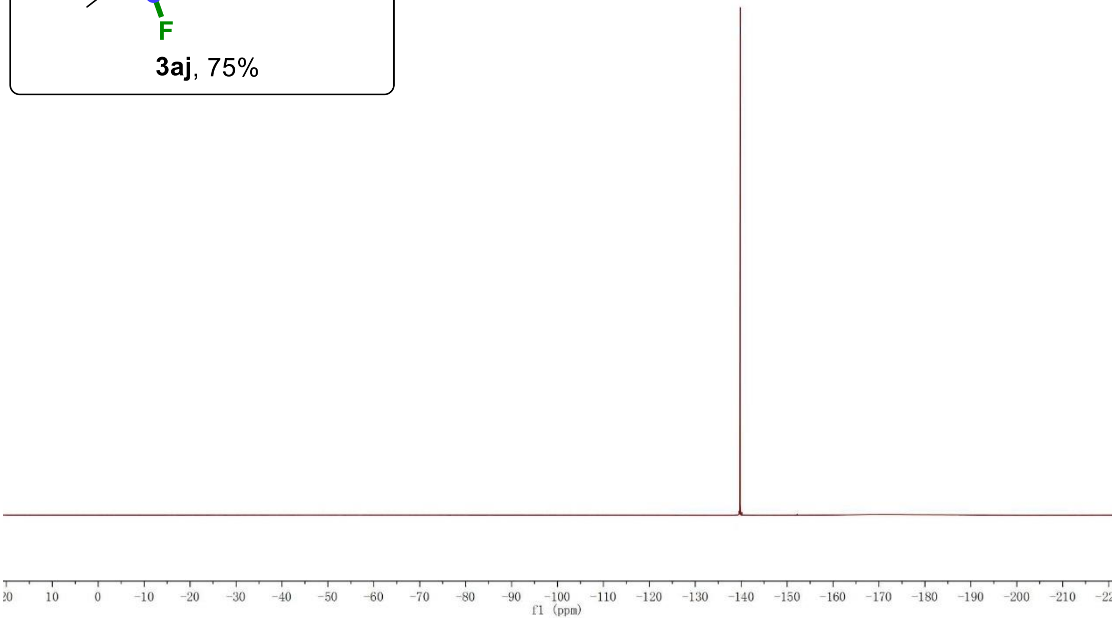


Supplementary Figure 128. $^{1}H$ NMR, $^{13}C$ NMR and $^{19}F$ NMR spectrum of **3aj**.

## 2-(2-fluoro-6-methoxy-1-methyl-1H-indol-3-yl)-1-phenylethan-1-one (3ak)

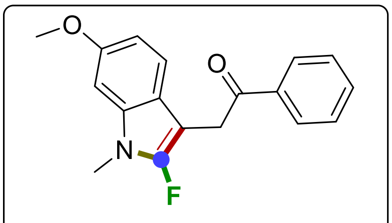


**3ak**, 81%

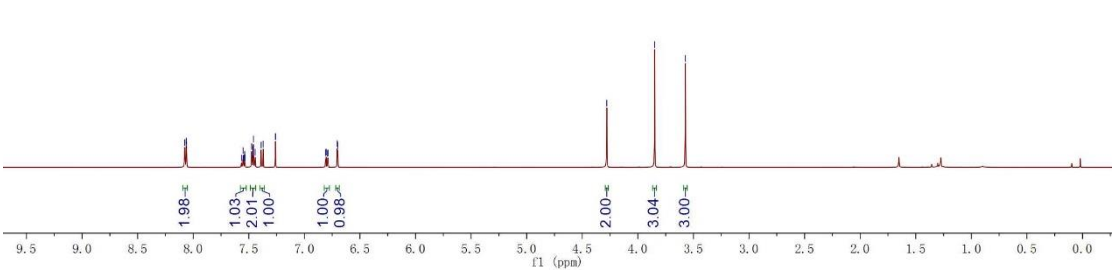



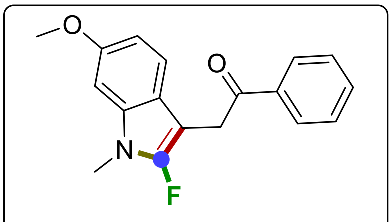


**3ak**, 81%


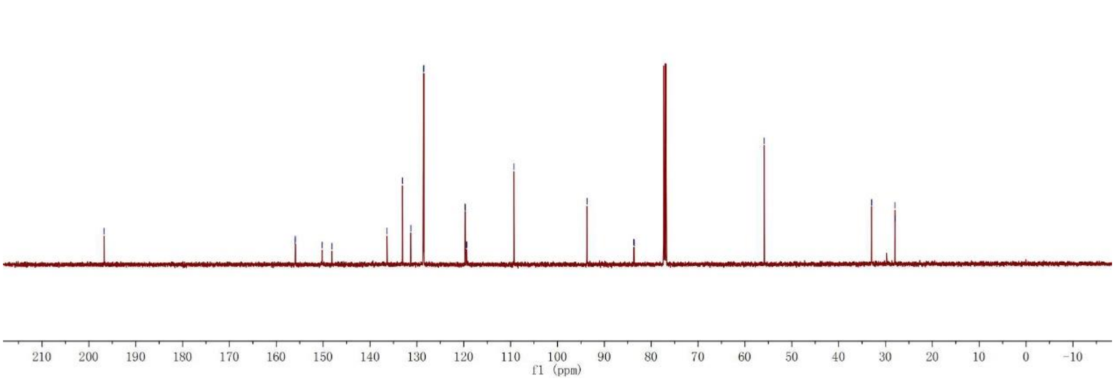

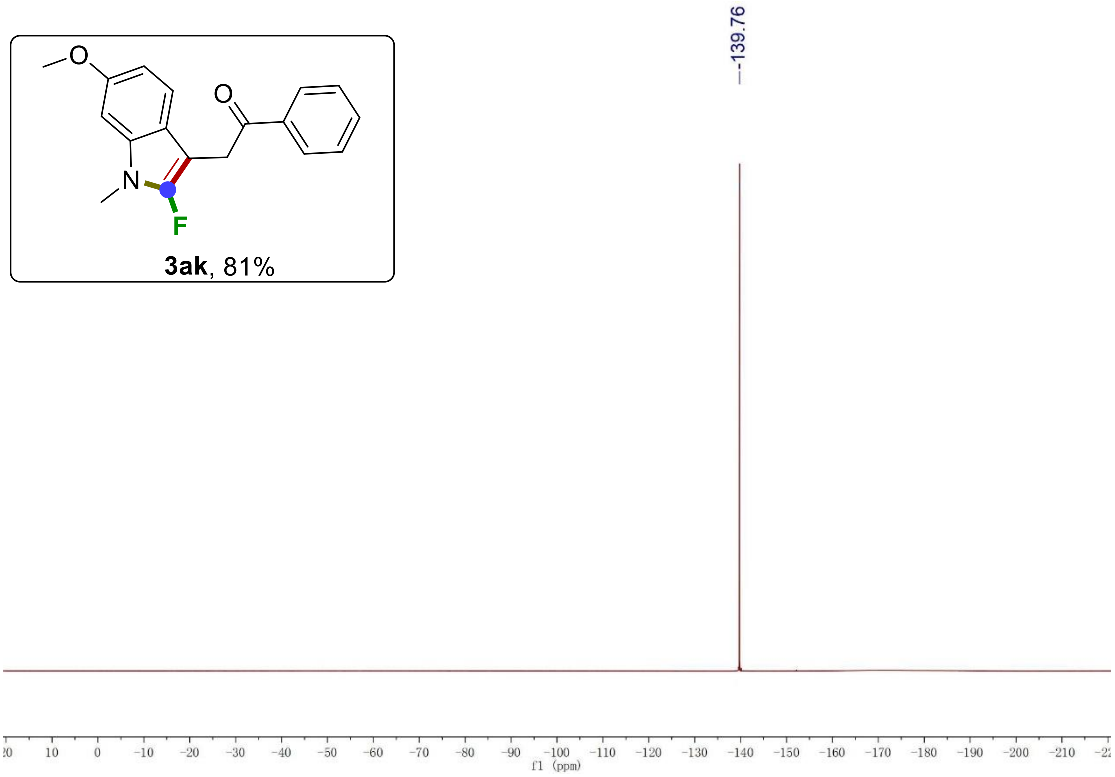


Supplementary Figure 129. $^{1}H$ NMR, $^{13}C$ NMR and $^{19}F$ NMR spectrum of **3ak**.

## 2-(6-bromo-2-fluoro-1-methyl-1H-indol-3-yl)-1-phenylethan-1-one (3al)

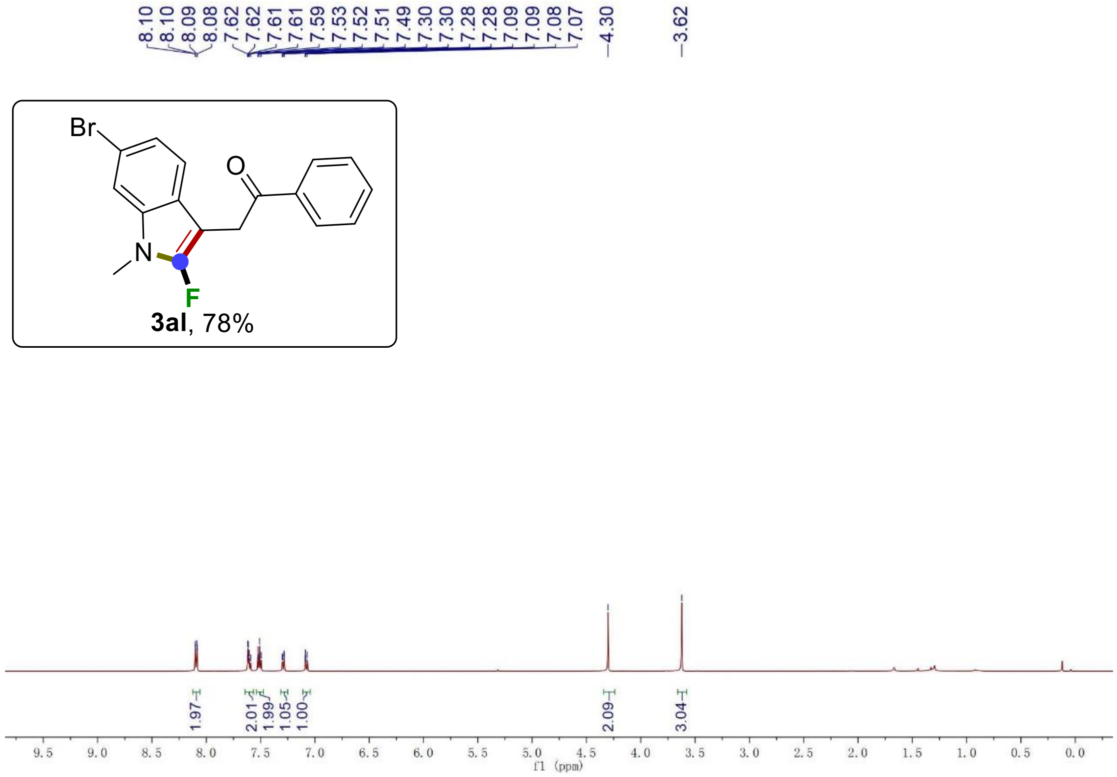


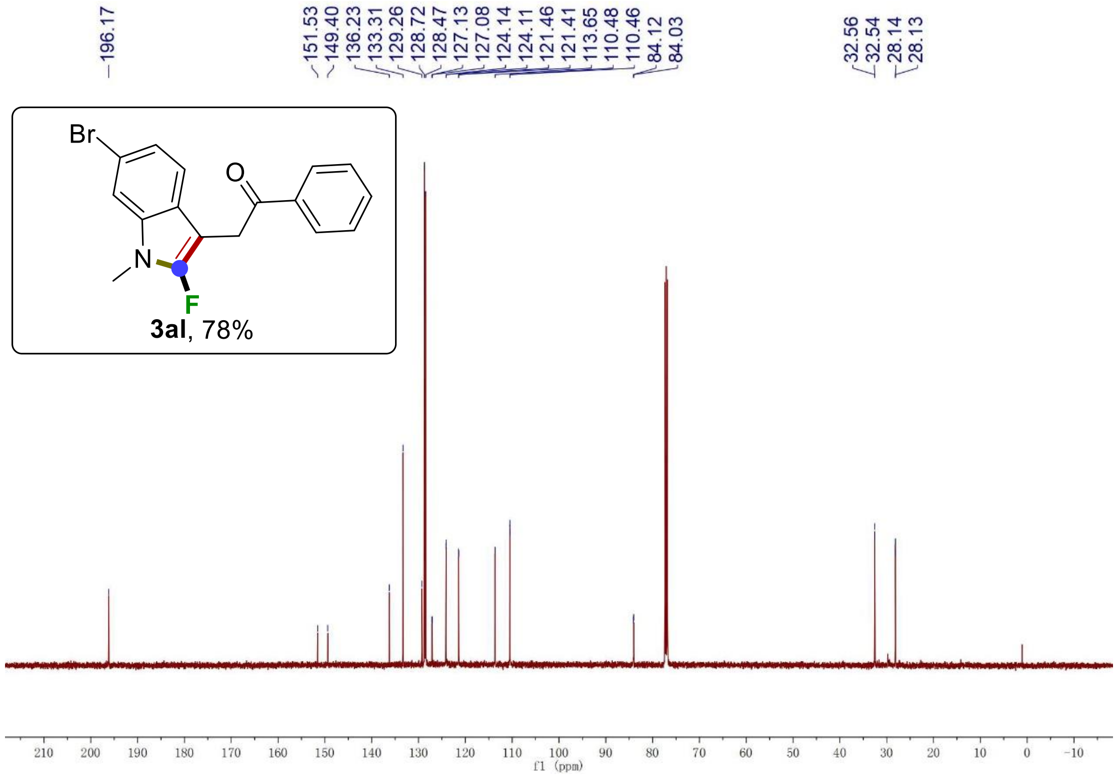

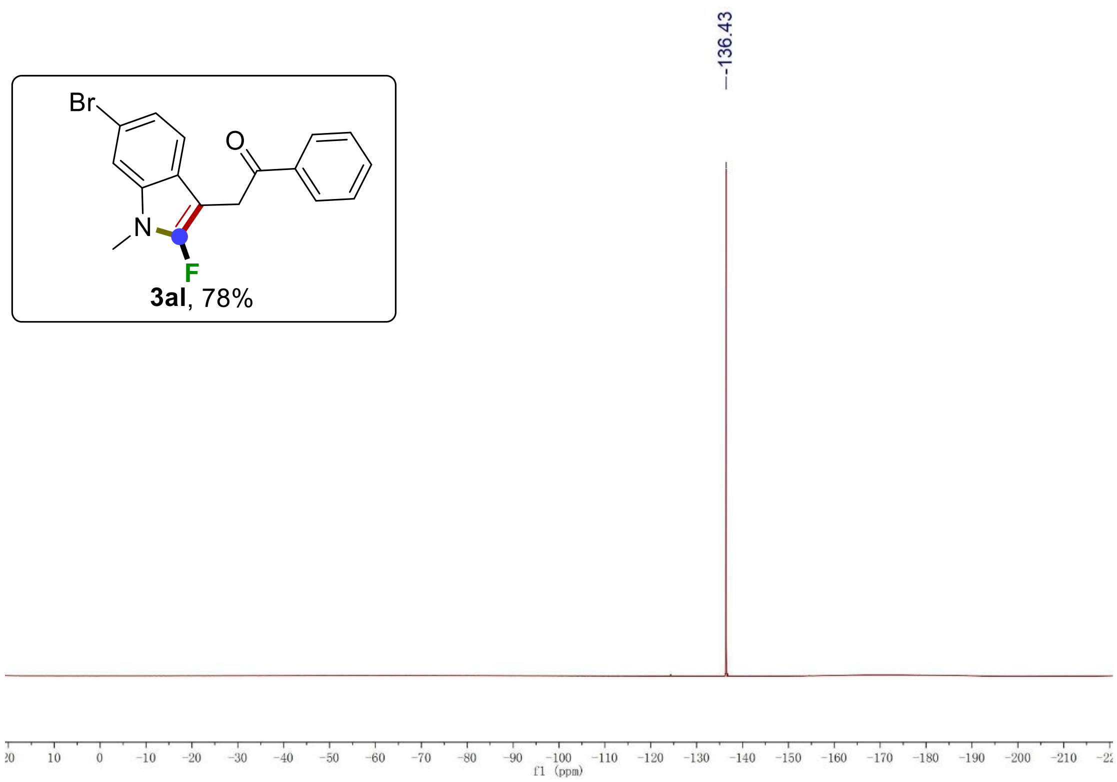


Supplementary Figure 130. $^{1}H$ NMR, $^{13}C$ NMR and $^{19}F$ NMR spectrum of **3al**.

## 2-(2,7-difluoro-1-methyl-1H-indol-3-yl)-1-phenylethan-1-one (3am)

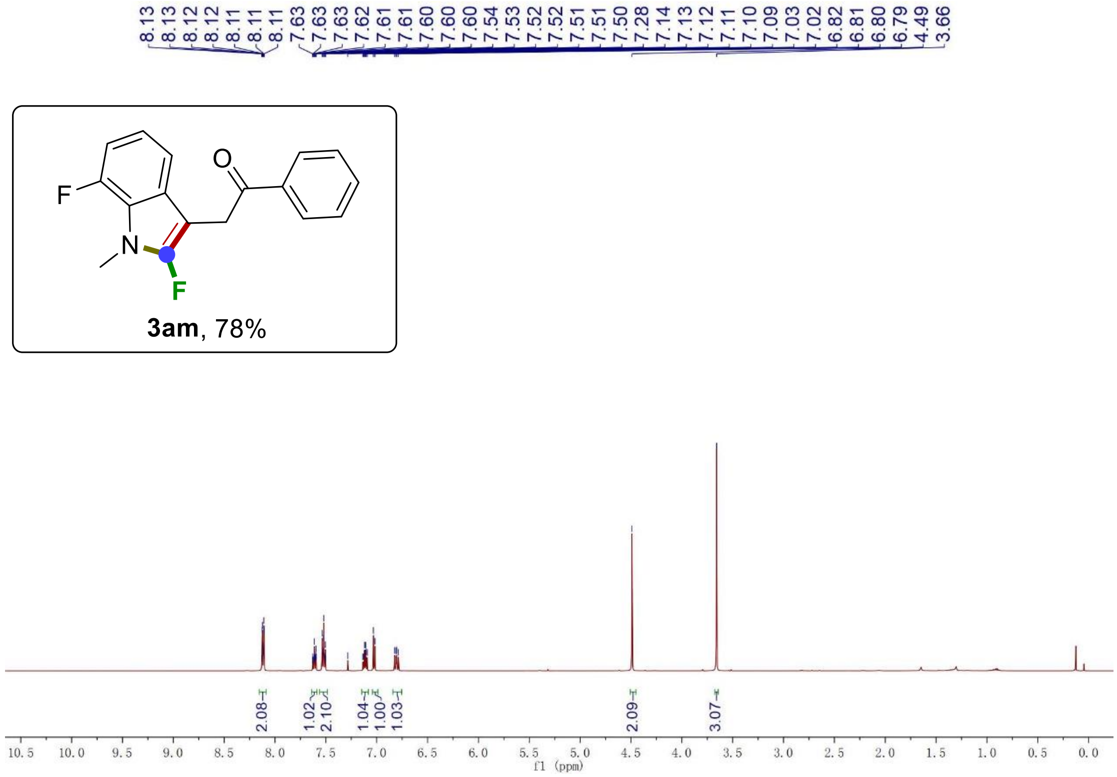


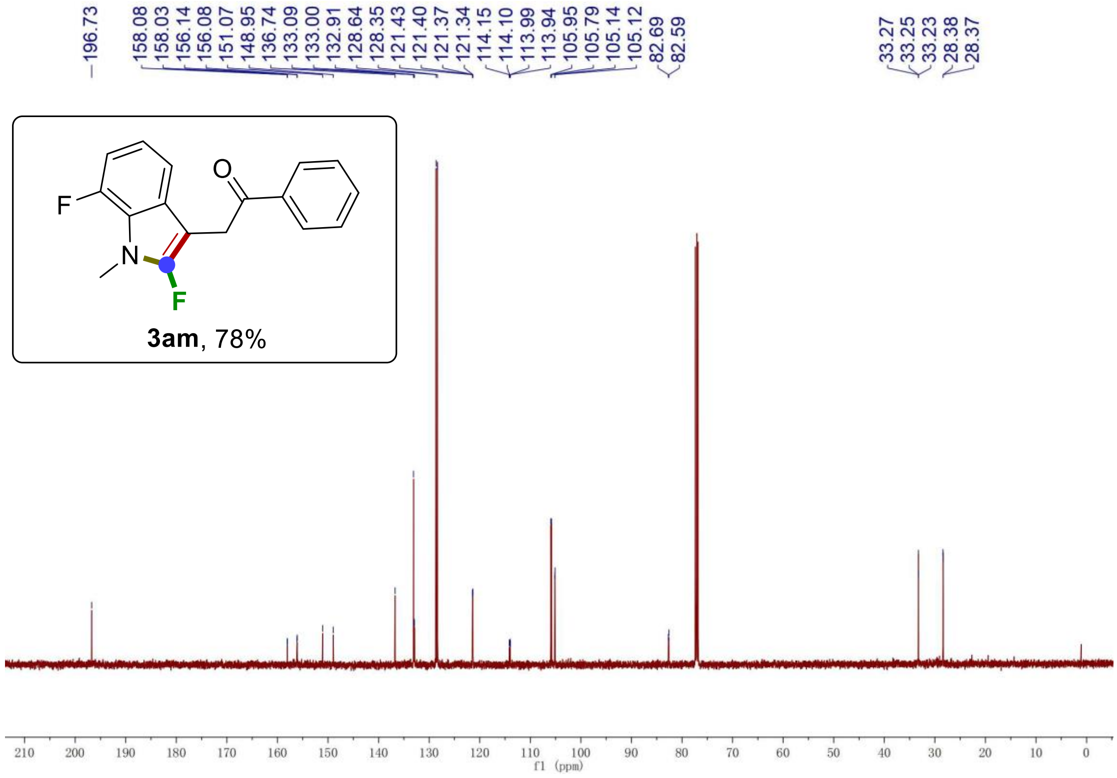

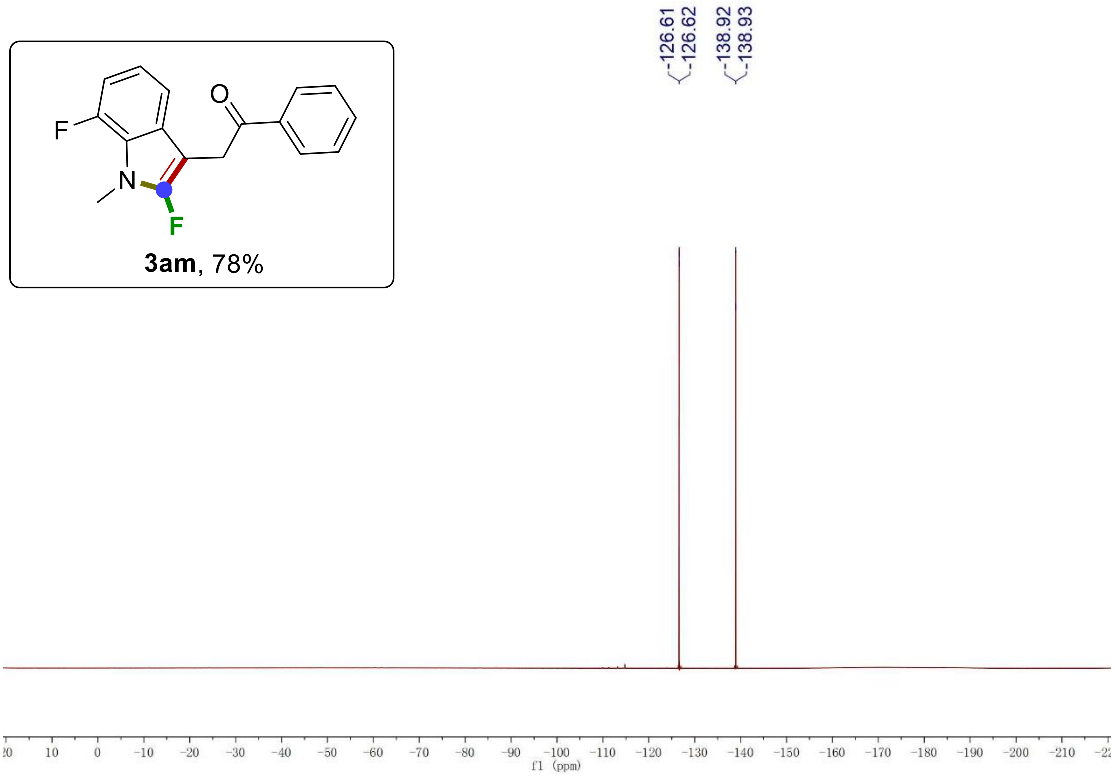


Supplementary Figure 131. $^{1}H$ NMR, $^{13}C$ NMR and $^{19}F$ NMR spectrum of **3am**.

**2-(7-chloro-2-fluoro-1-methyl-1H-indol-3-yl)-1-phenylethan-1-one (3an)**

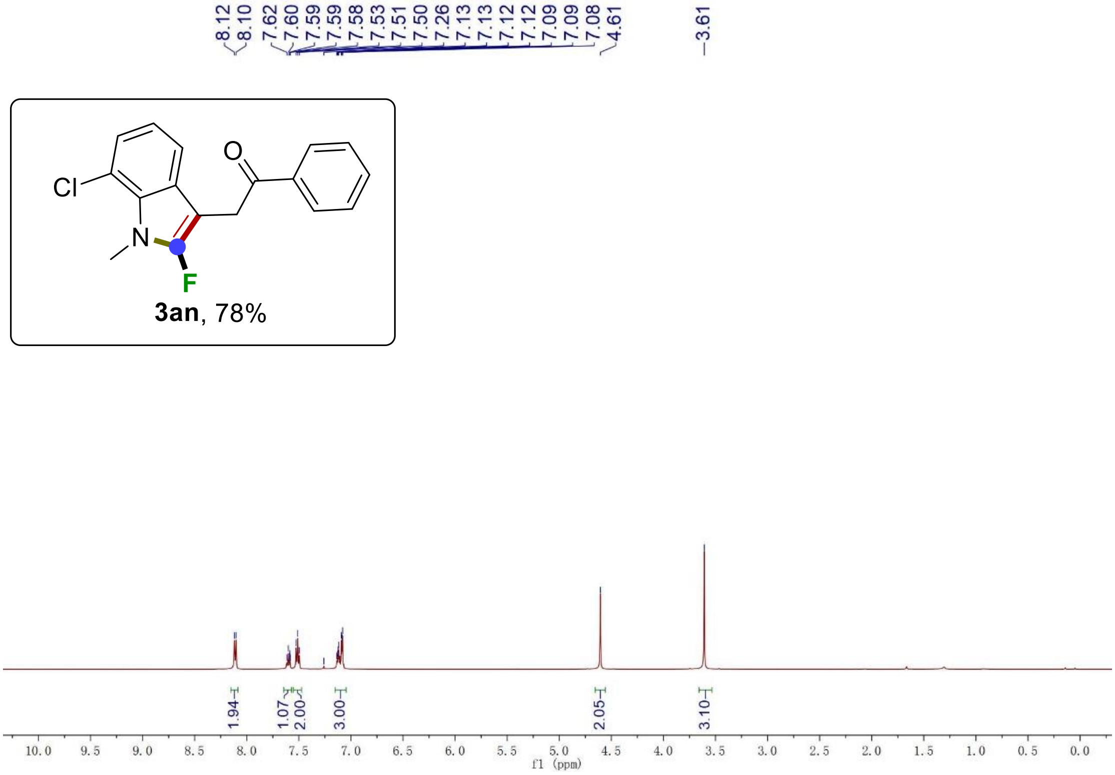


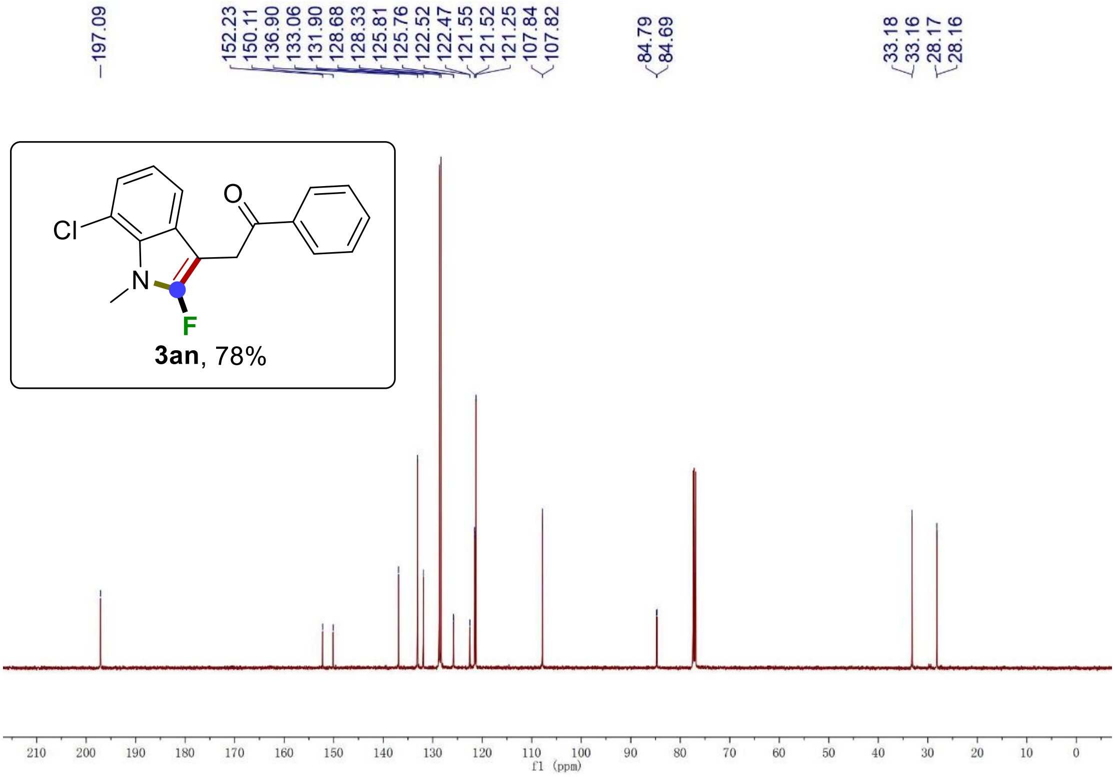

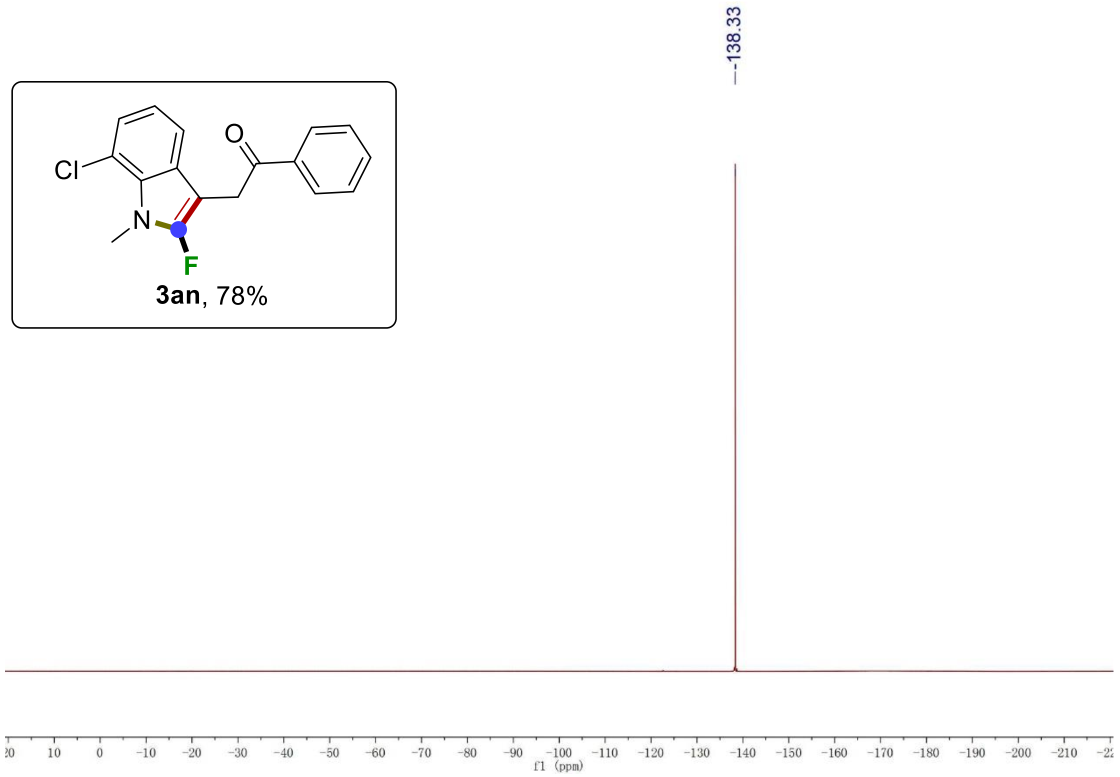


Supplementary Figure 132. $^{1}H$ NMR, $^{13}C$ NMR and $^{19}F$ NMR spectrum of **3an**.

## 2-(1-ethyl-2-fluoro-1H-indol-3-yl)-1-phenylethan-1-one (3ao)

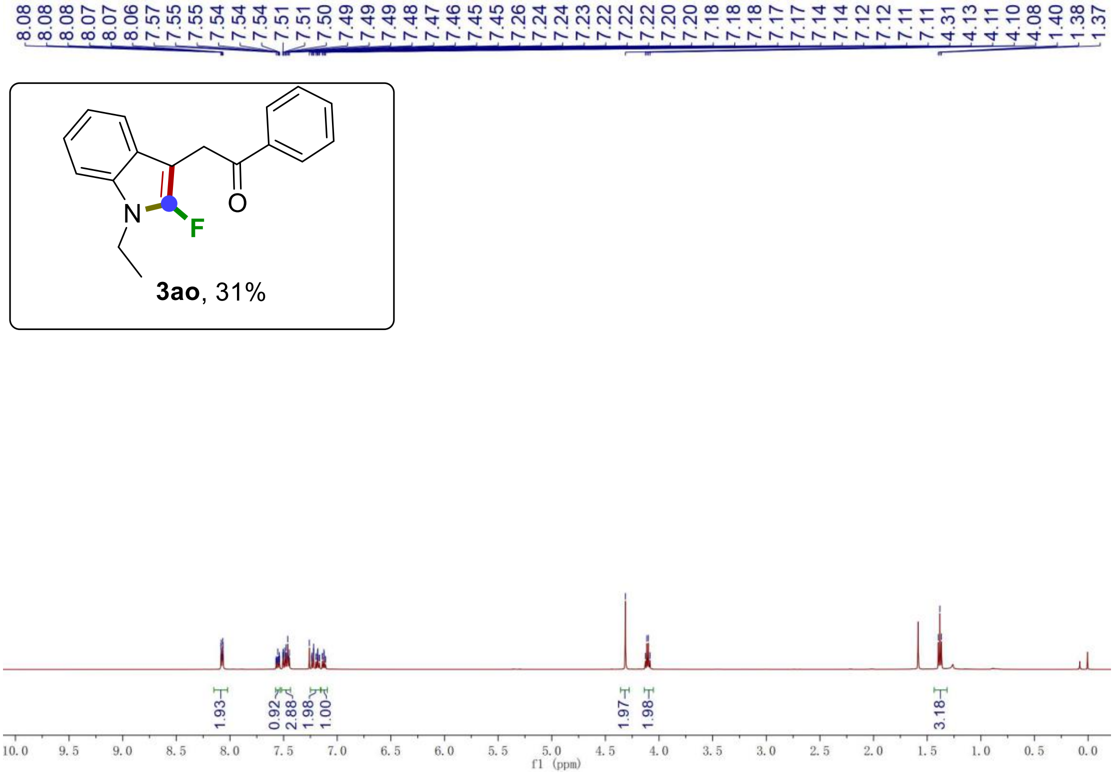




**3ao**, 31%

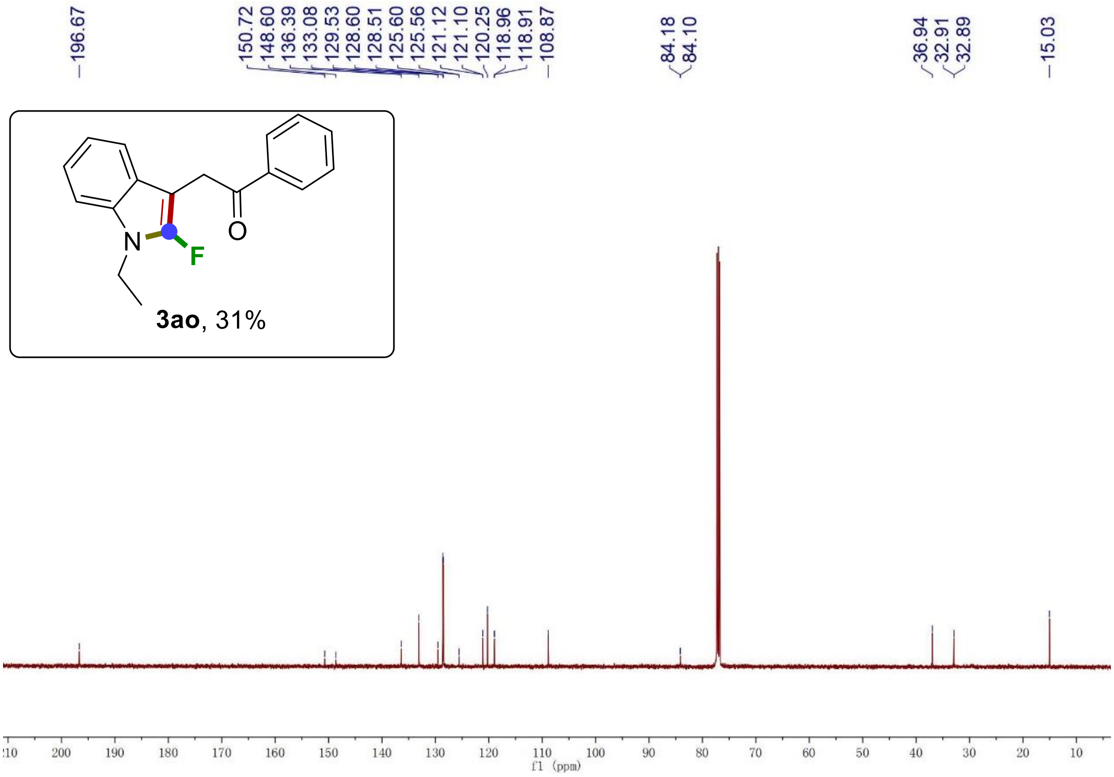




**3ao**, 31%

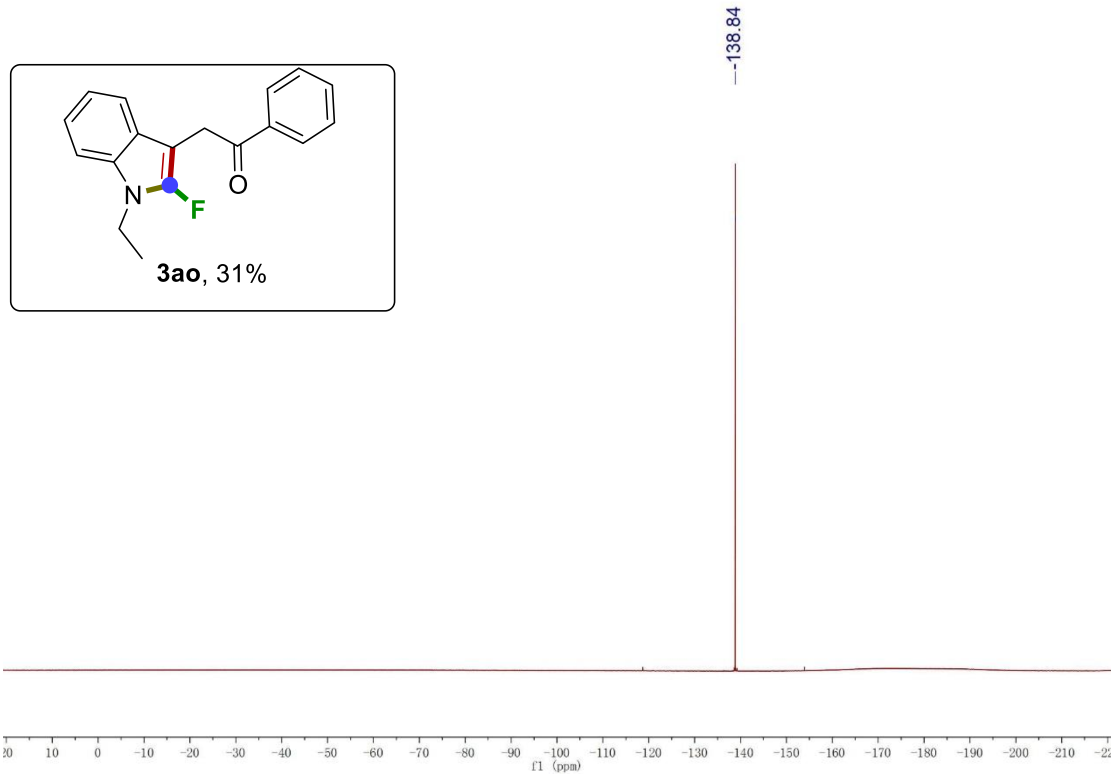


Supplementary Figure 133. $^{1}$H NMR, $^{13}$C NMR and $^{19}$F NMR spectrum of **3ao**.

**methyl 2-(2-fluoro-1-methyl-1H-indol-3-yl)acetate (5a)**

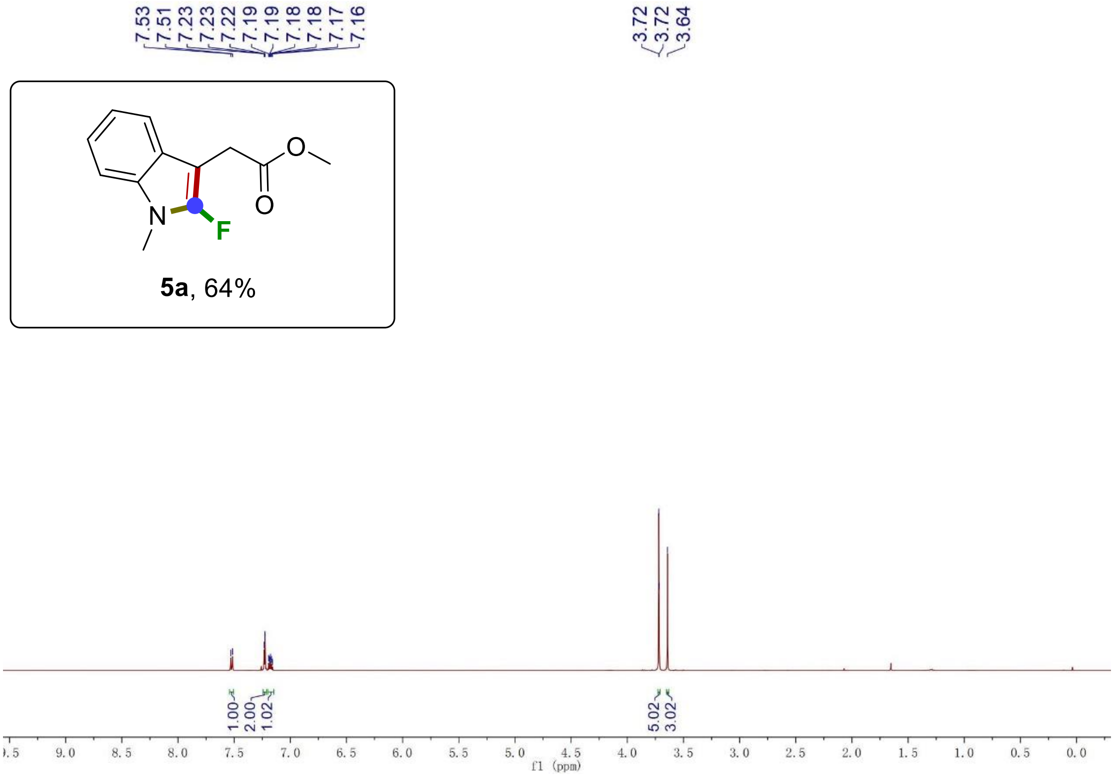


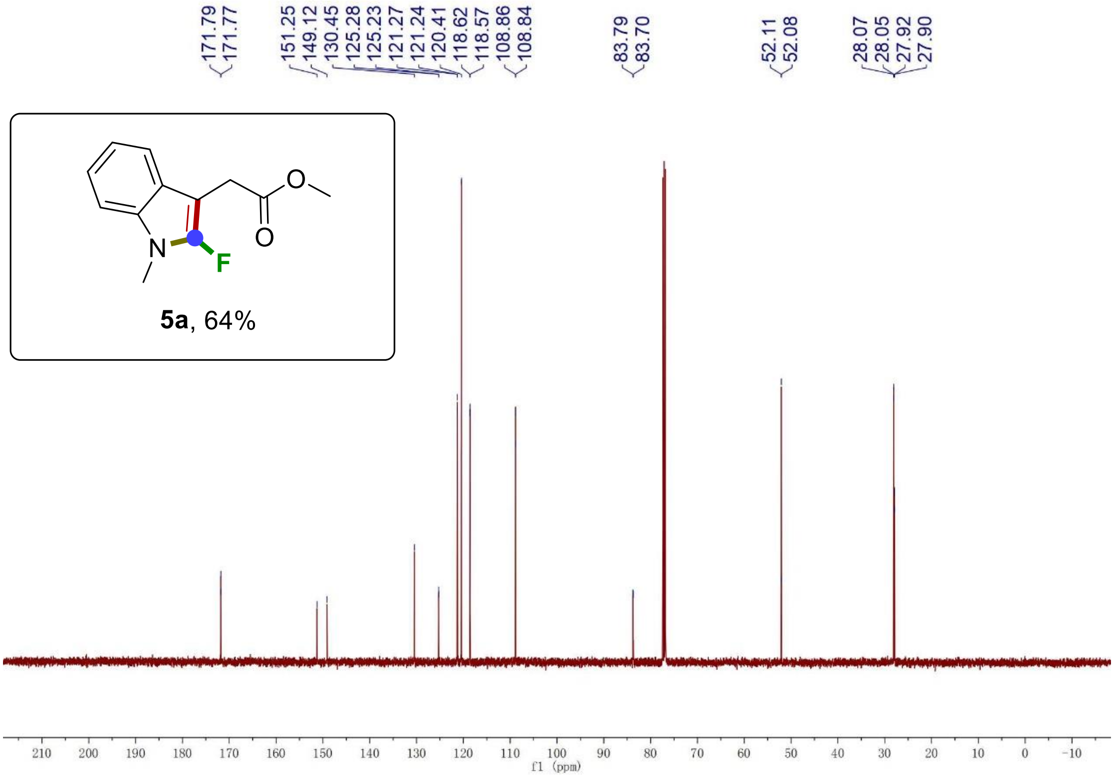

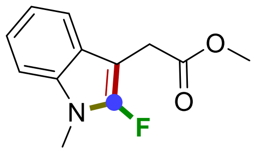


**5a**, 64%

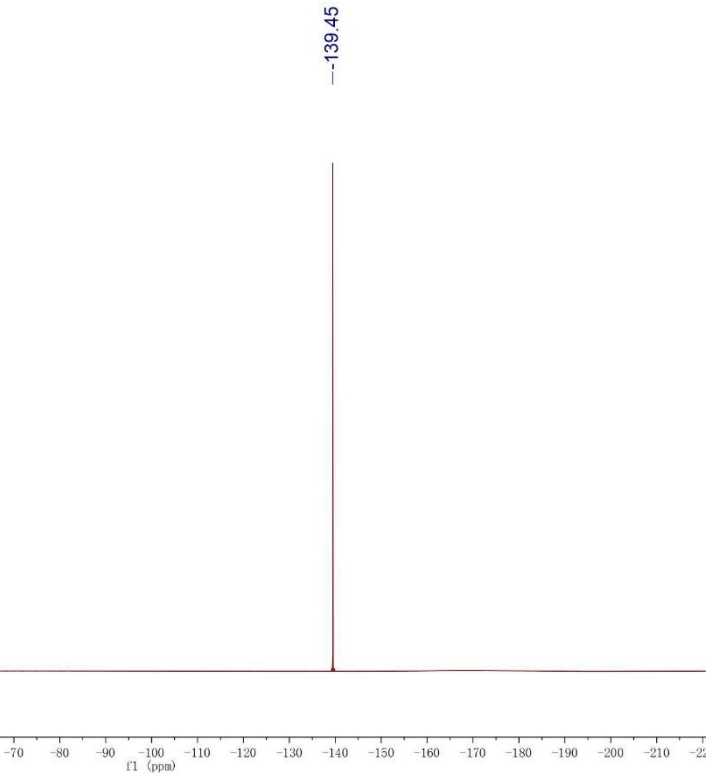


Supplementary Figure 134. $^{1}H$ NMR, $^{13}C$ NMR and $^{19}F$ NMR spectrum of **5a**.

**ethyl 2-(2-fluoro-1-methyl-1H-indol-3-yl)acetate (5b)**

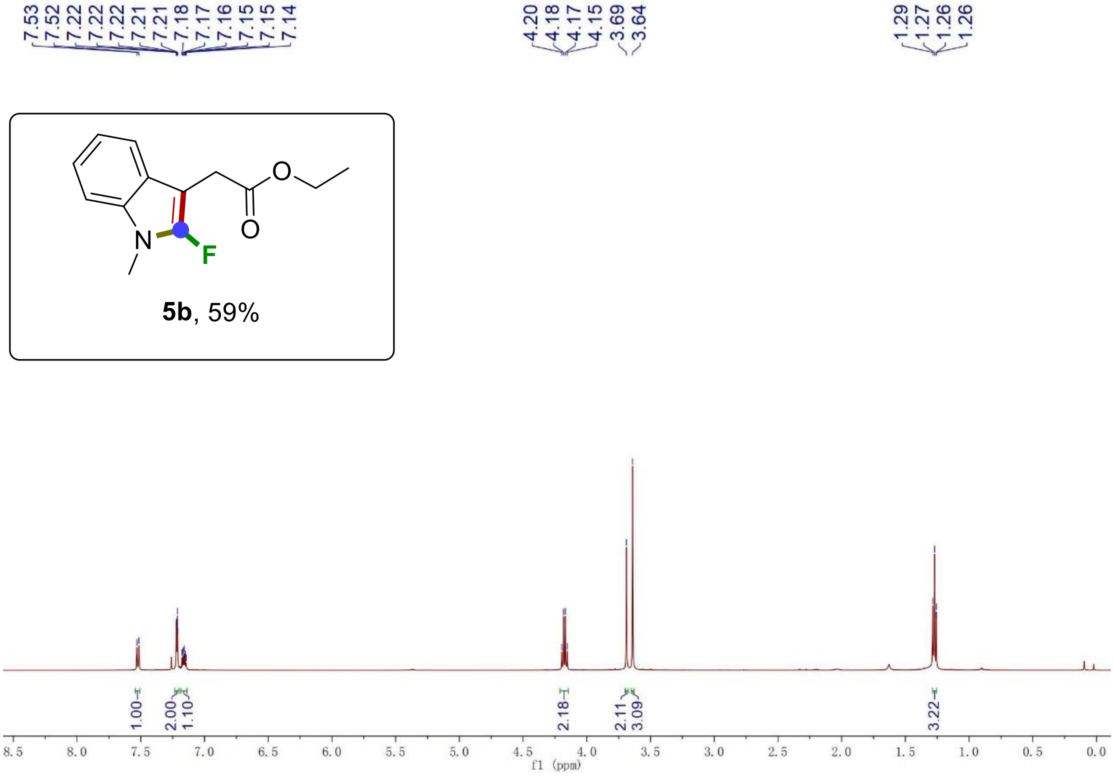


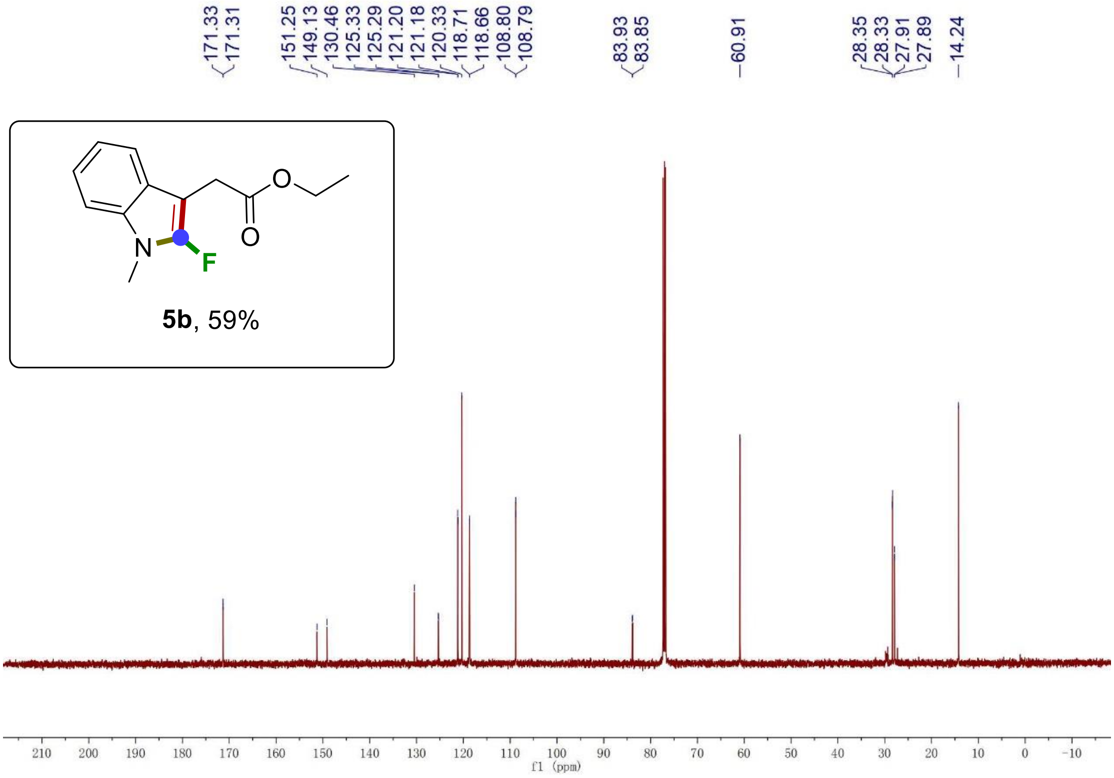

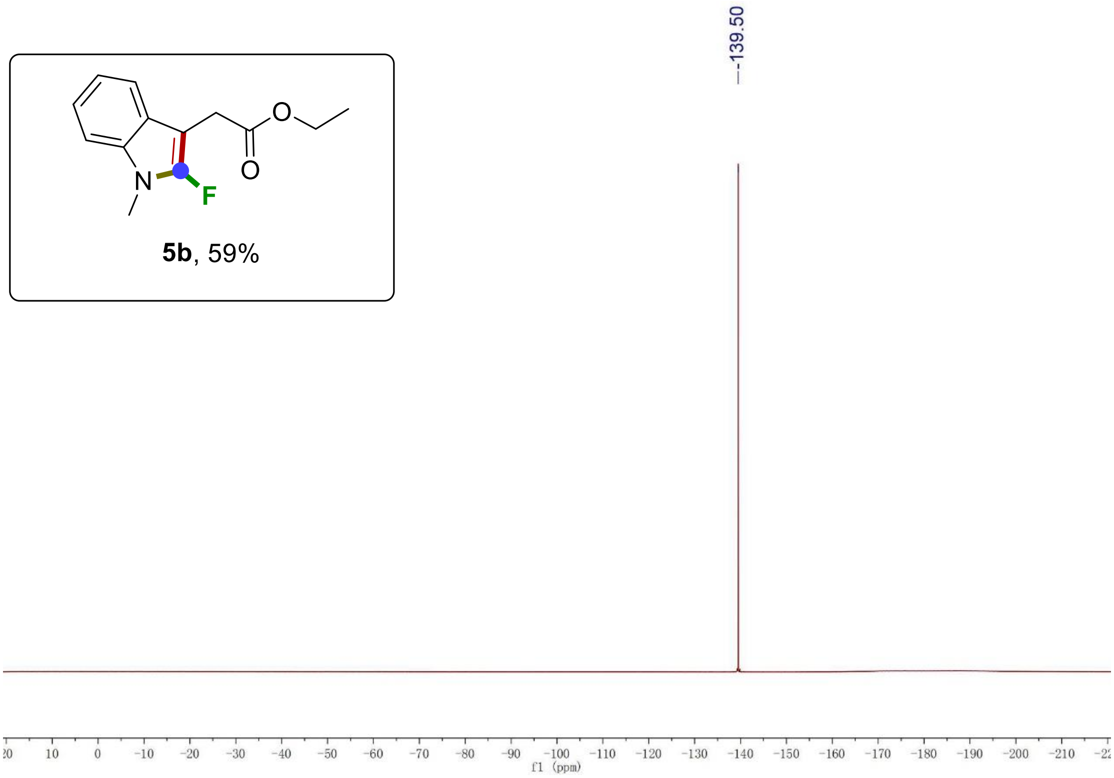


Supplementary Figure 135. $^{1}H$ NMR, $^{13}C$ NMR and $^{19}F$ NMR spectrum of **5b**.

**tert-butyl 2-(2-fluoro-1-methyl-1H-indol-3-yl)acetate (5c)**

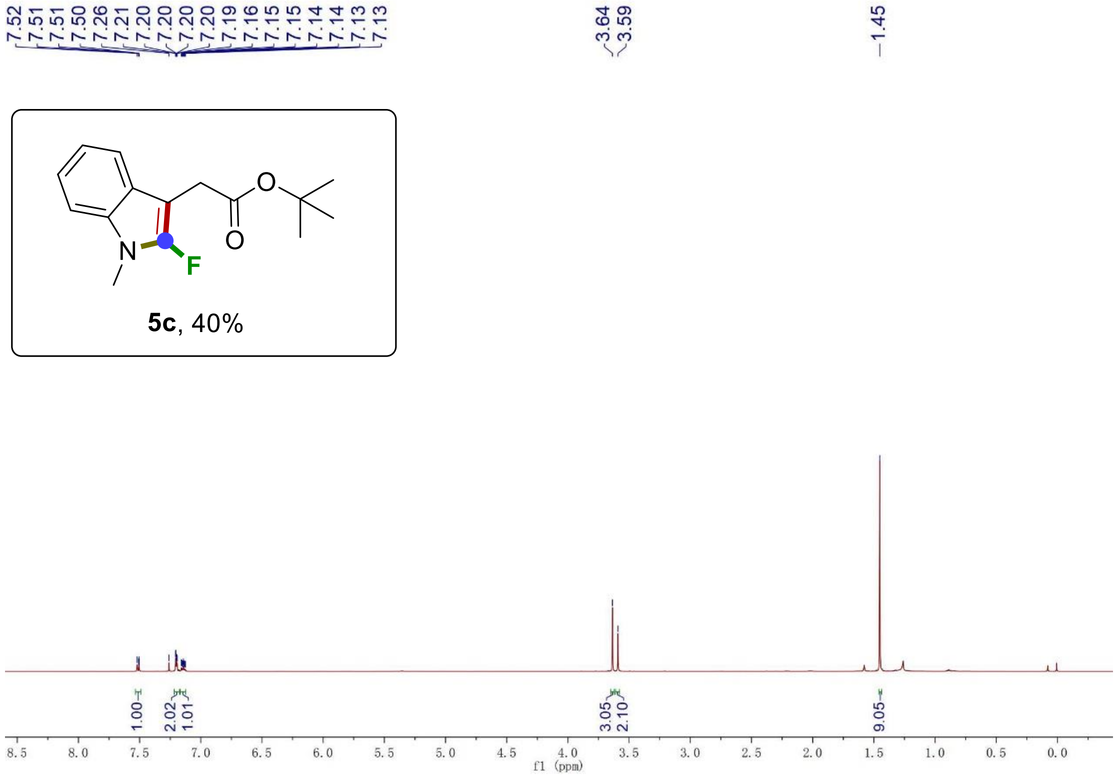


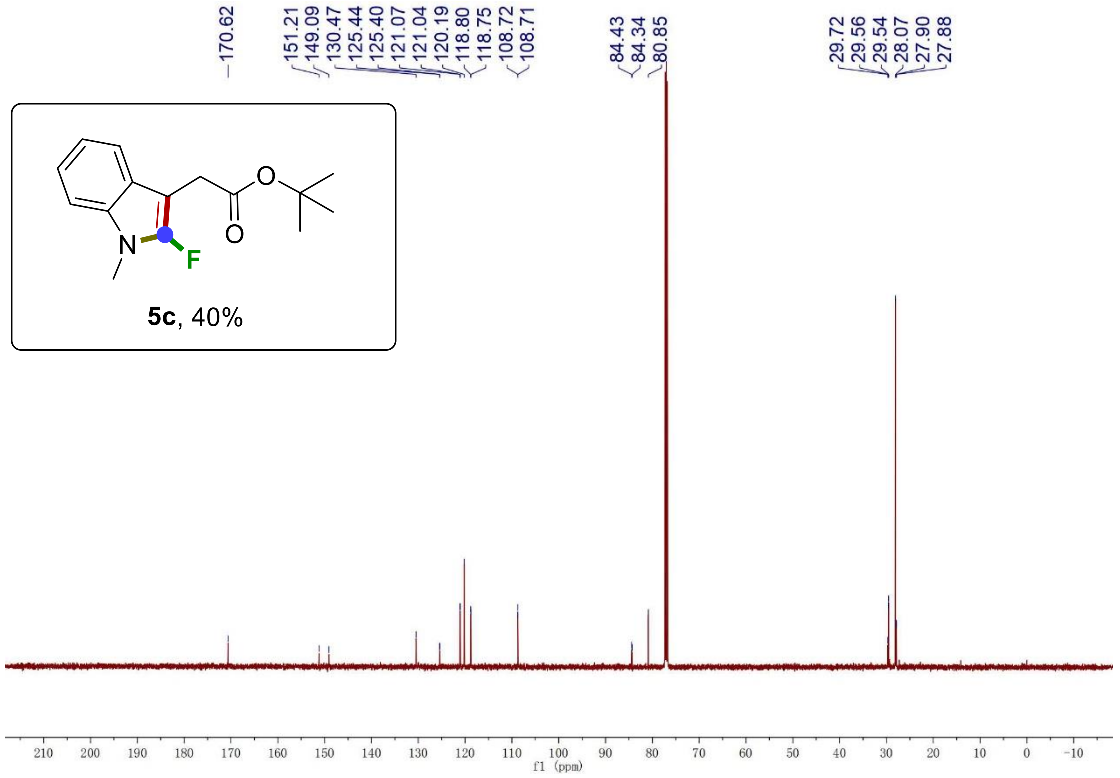

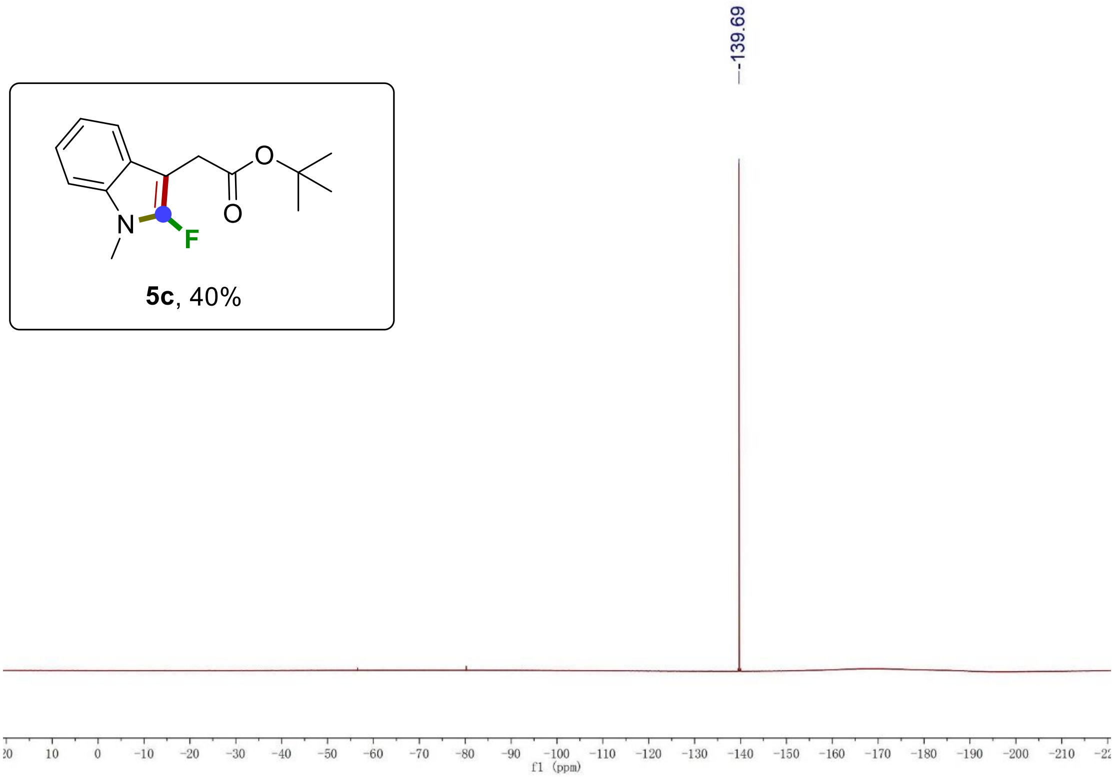


Supplementary Figure 136. $^{1}H$ NMR, $^{13}C$ NMR and $^{19}F$ NMR spectrum of **5c**.

**benzyl 2-(2-fluoro-1-methyl-1H-indol-3-yl)acetate (5d)**

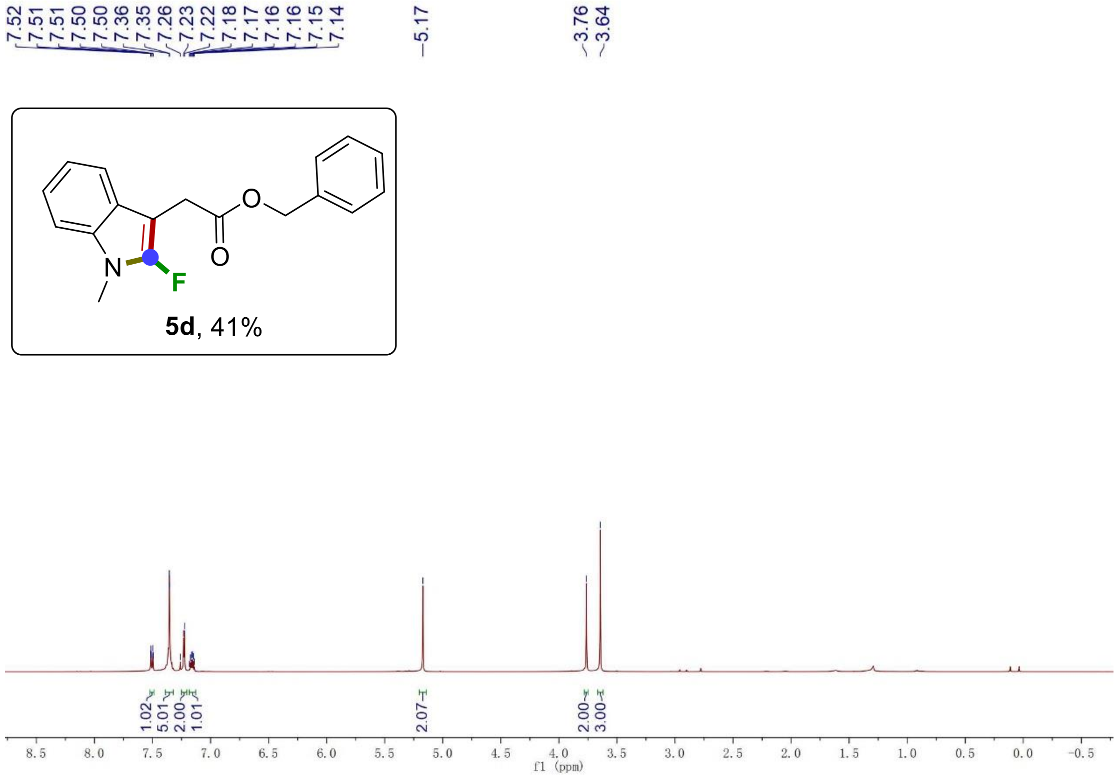


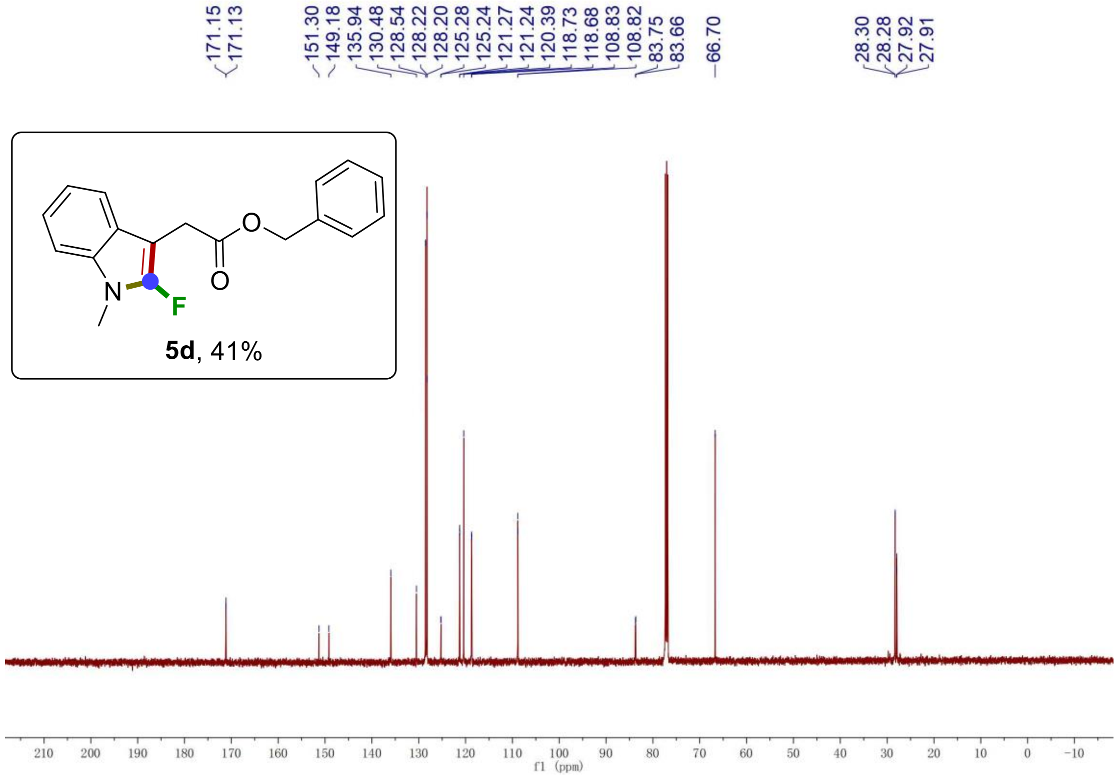

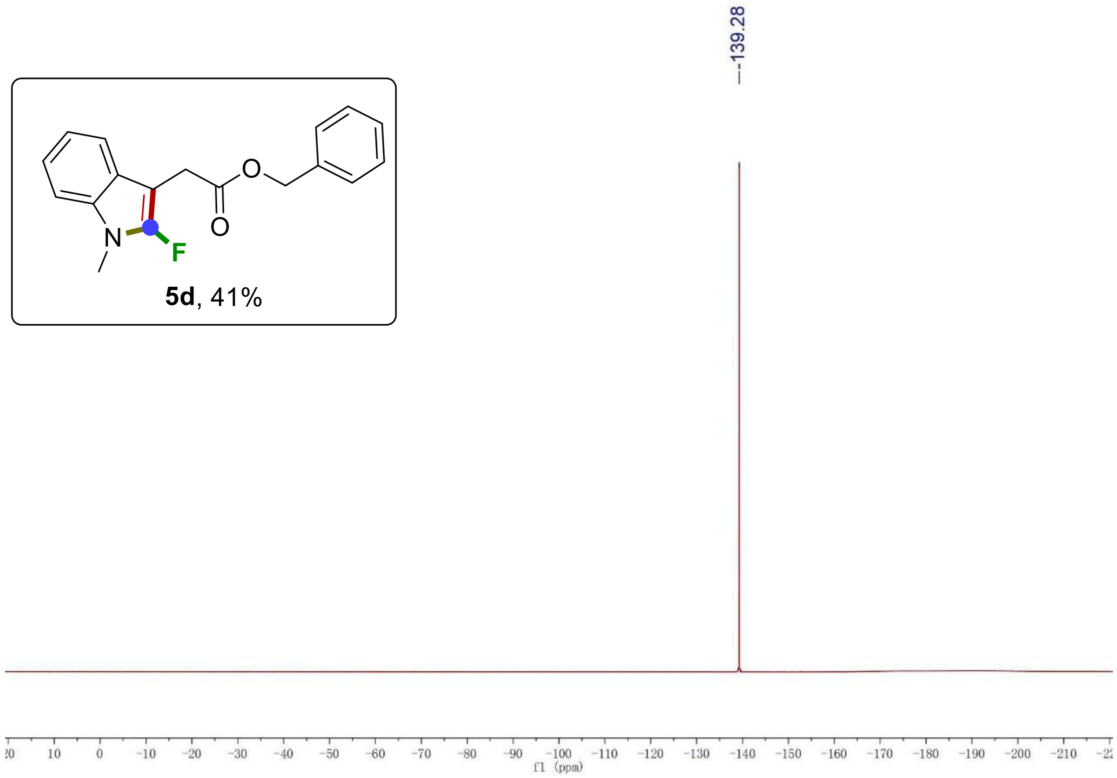


Supplementary Figure 137. $^{1}H$ NMR, $^{13}C$ NMR and $^{19}F$ NMR spectrum of **5d**.

**2-(2-fluoro-1-methyl-1H-indol-3-yl)acetonitrile (5e)**

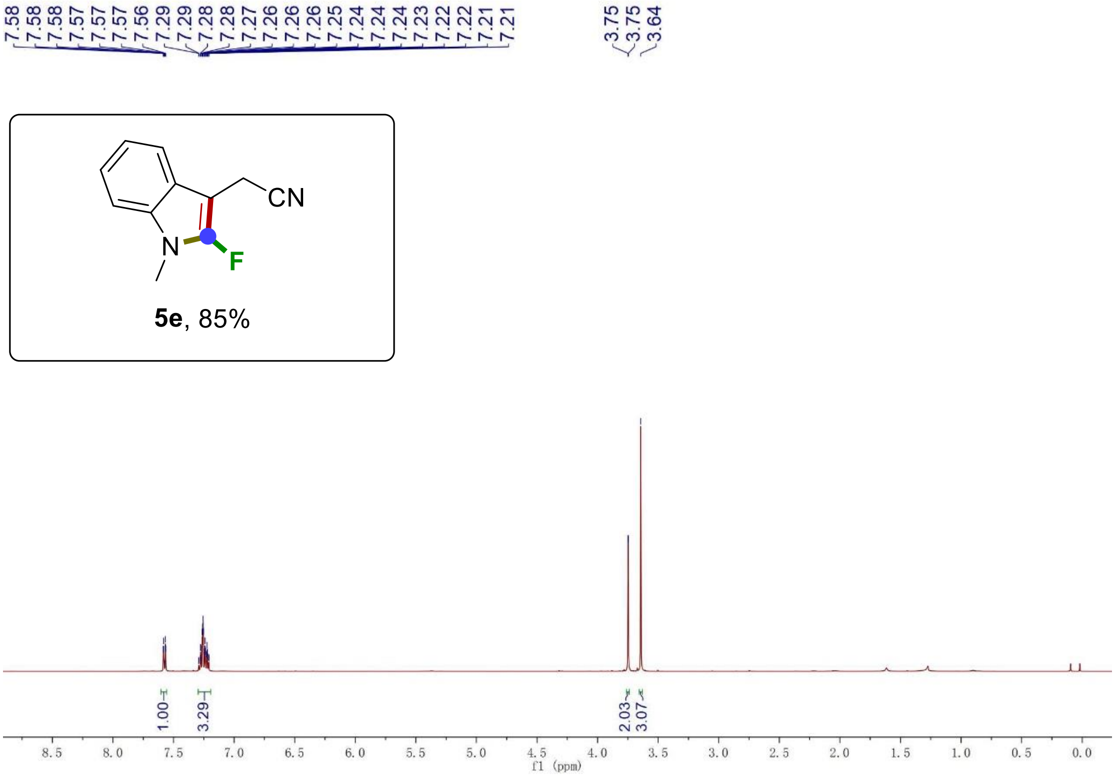

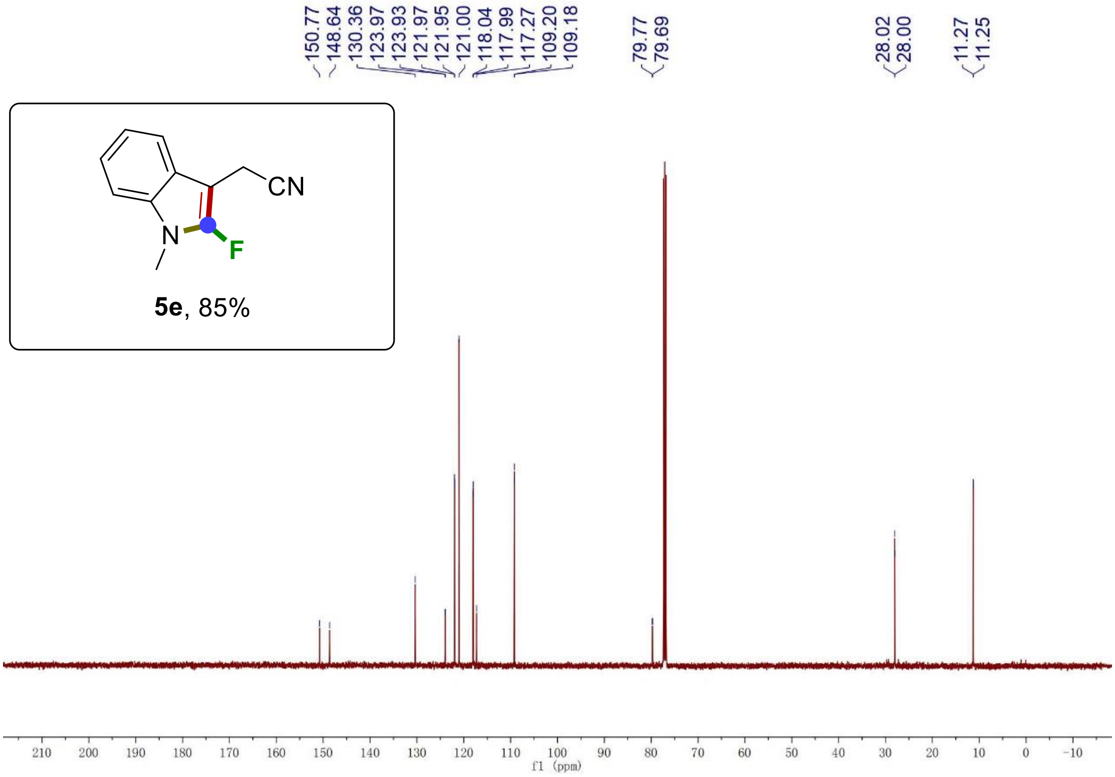

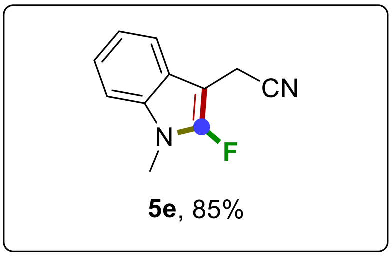


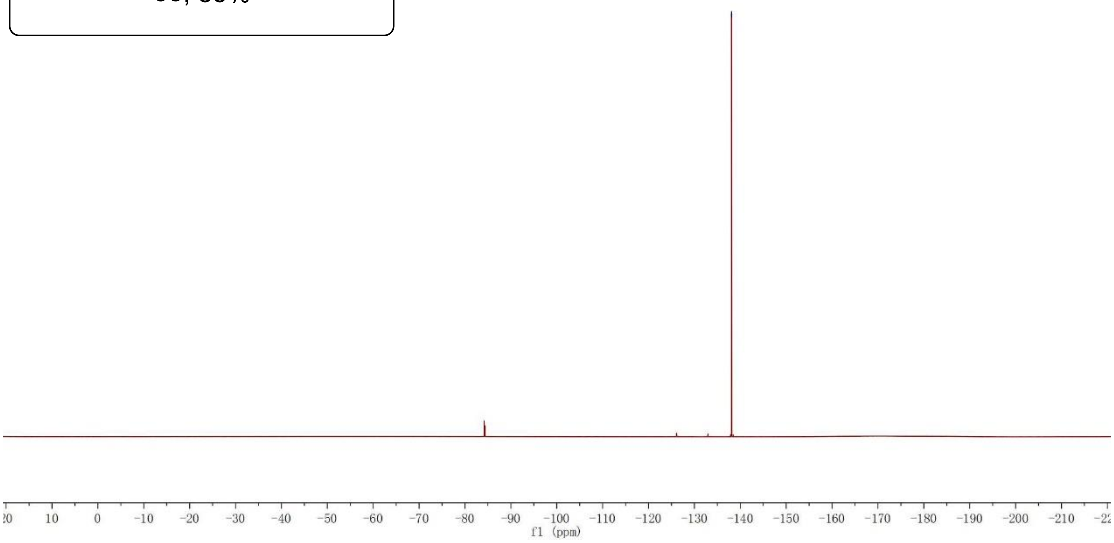


Supplementary Figure 138. $^{1}H$ NMR, $^{13}C$ NMR and $^{19}F$ NMR spectrum of **5e**.

**2,6-di-tert-butyl-4-(2-fluoro-1-methyl-1H-indol-3-yl)phenol (7a)**

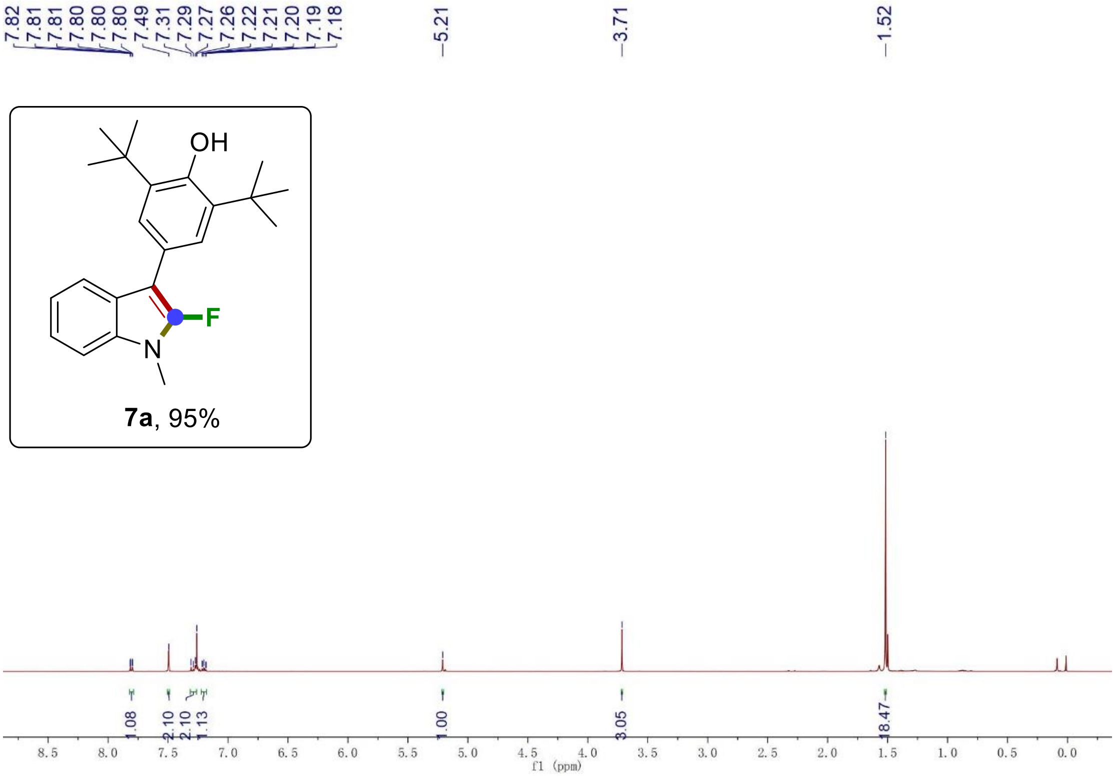


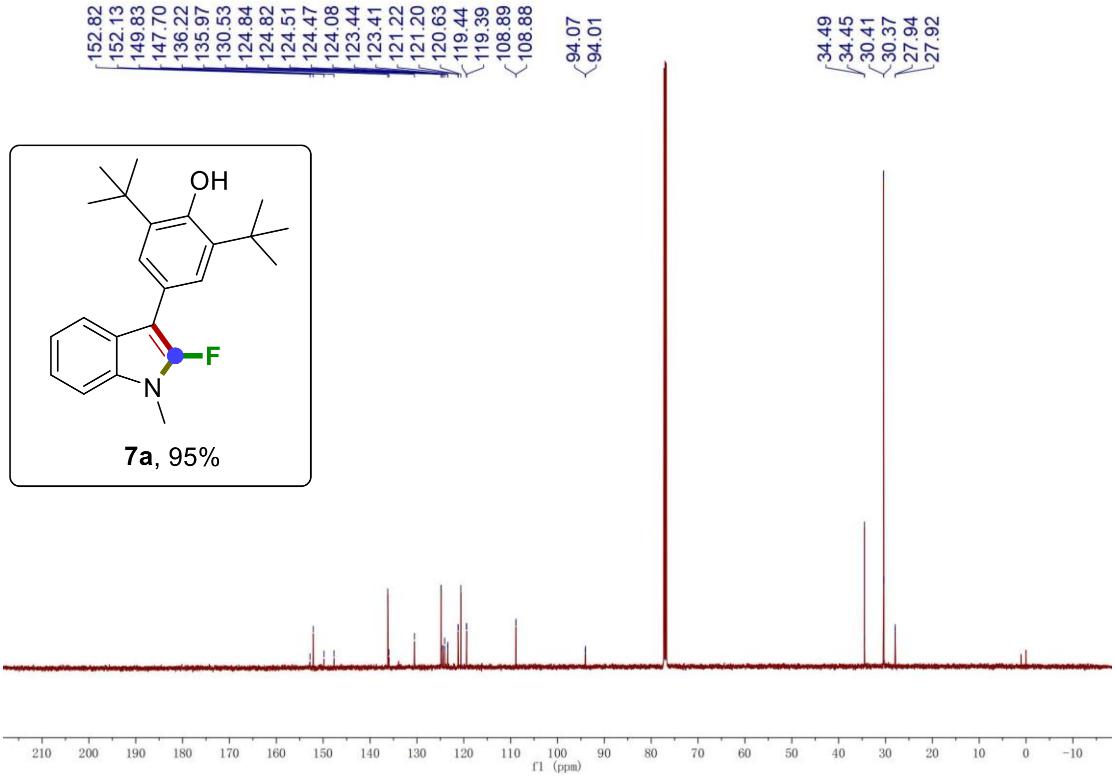

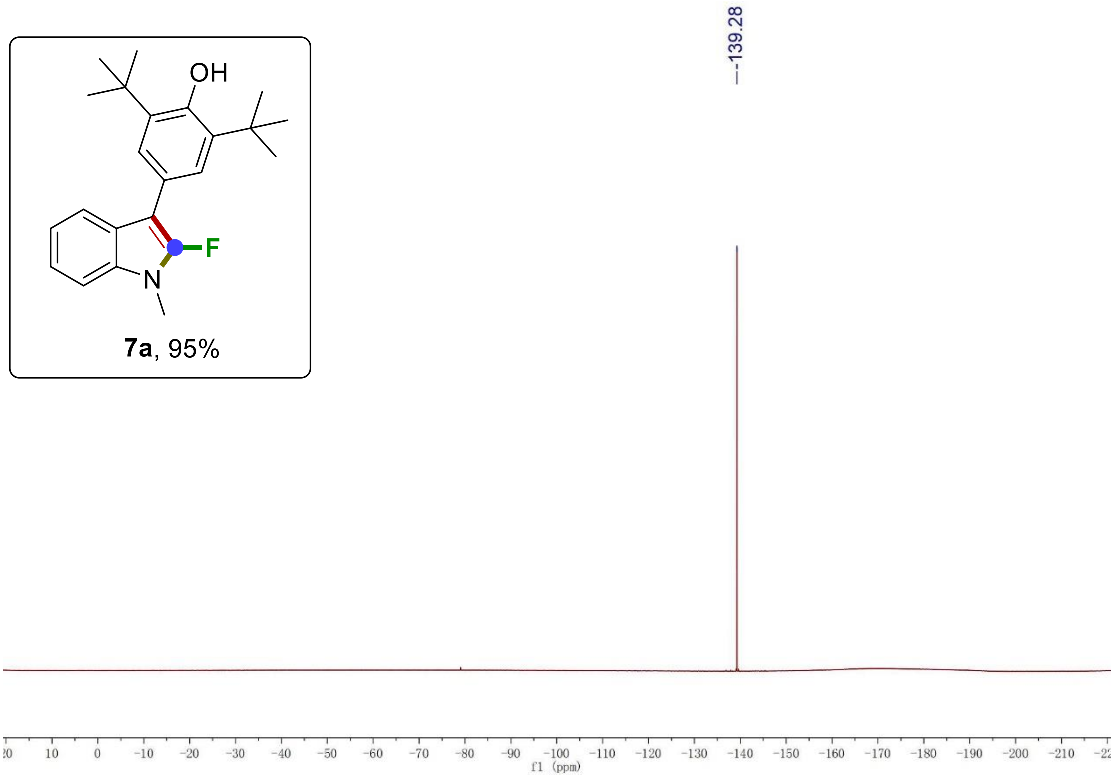


Supplementary Figure 139. $^{1}$H NMR, $^{13}$C NMR and $^{19}$F NMR spectrum of **7a**.

# 2,6-di-tert-butyl-4-(2-fluoro-1,5-dimethyl-1H-indol-3-yl)phenol (7b)

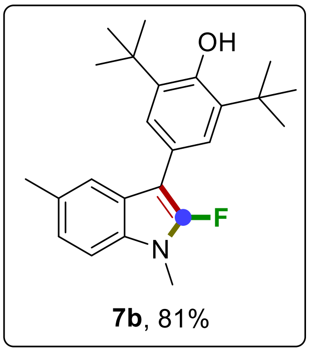


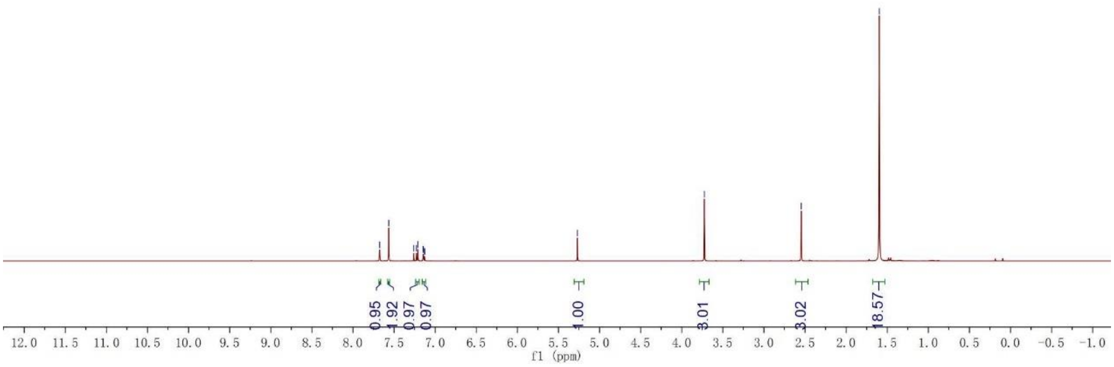


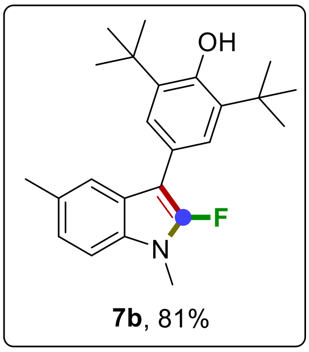


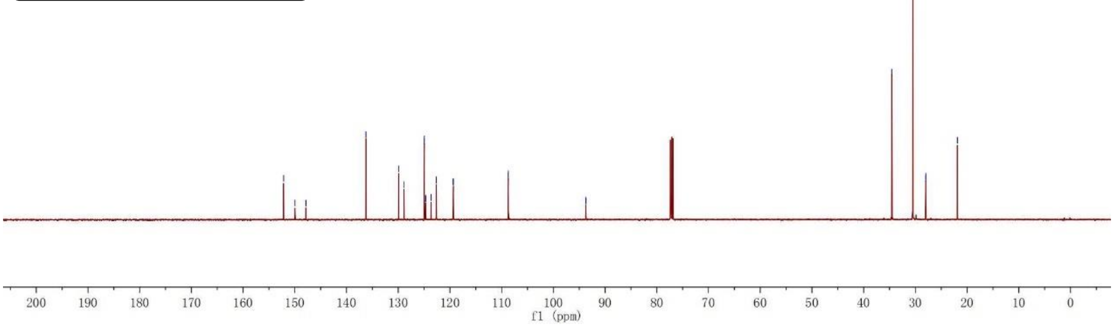

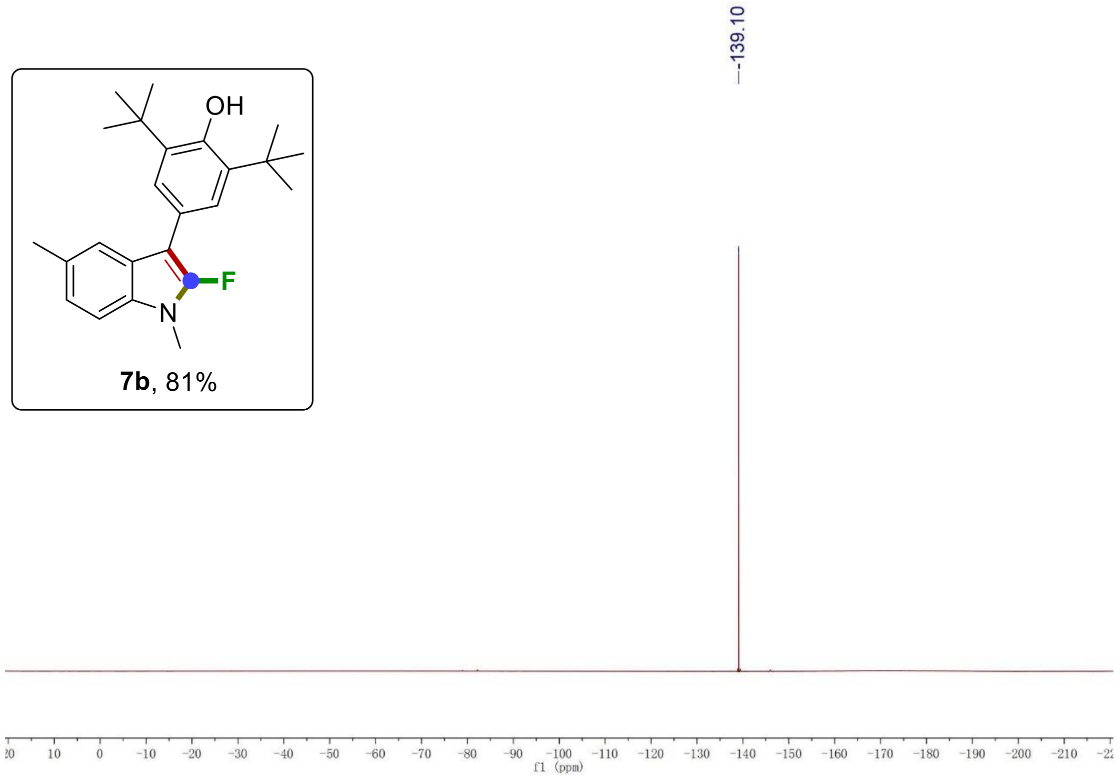


Supplementary Figure 140. $^{1}$H NMR, $^{13}$C NMR and $^{19}$F NMR spectrum of **7b**.

**4-(5-bromo-2-fluoro-1-methyl-1H-indol-3-yl)-2,6-di-tert-butylphenol (7c)**

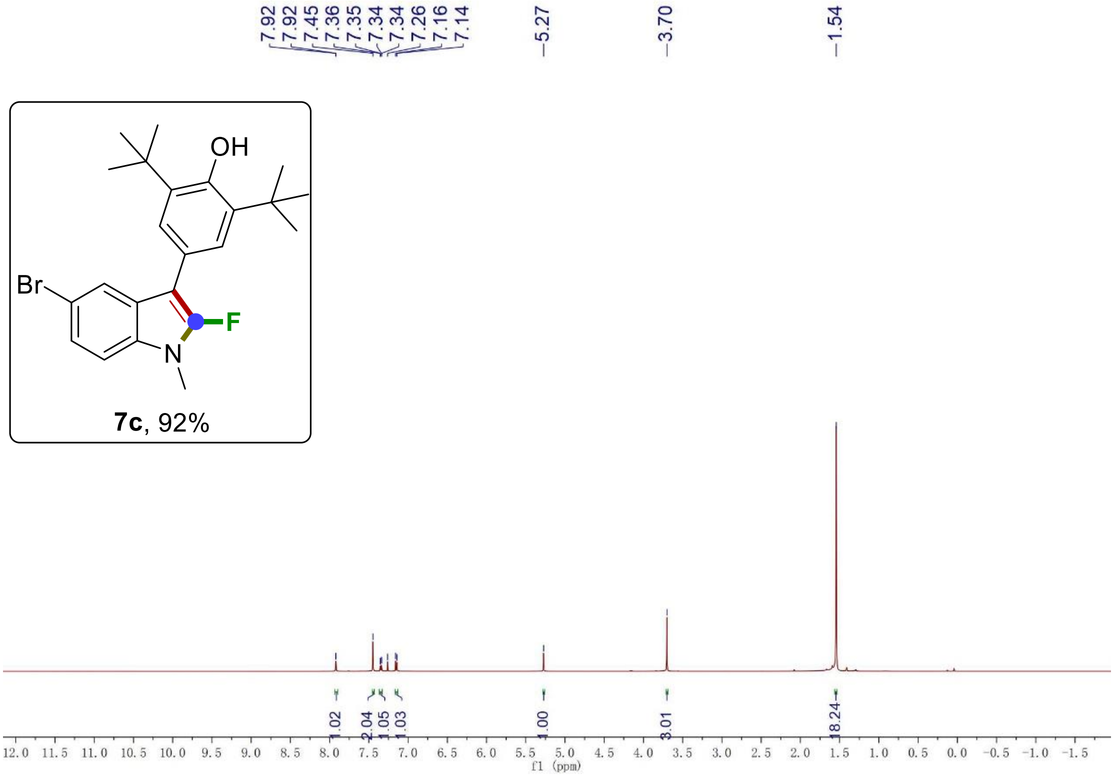


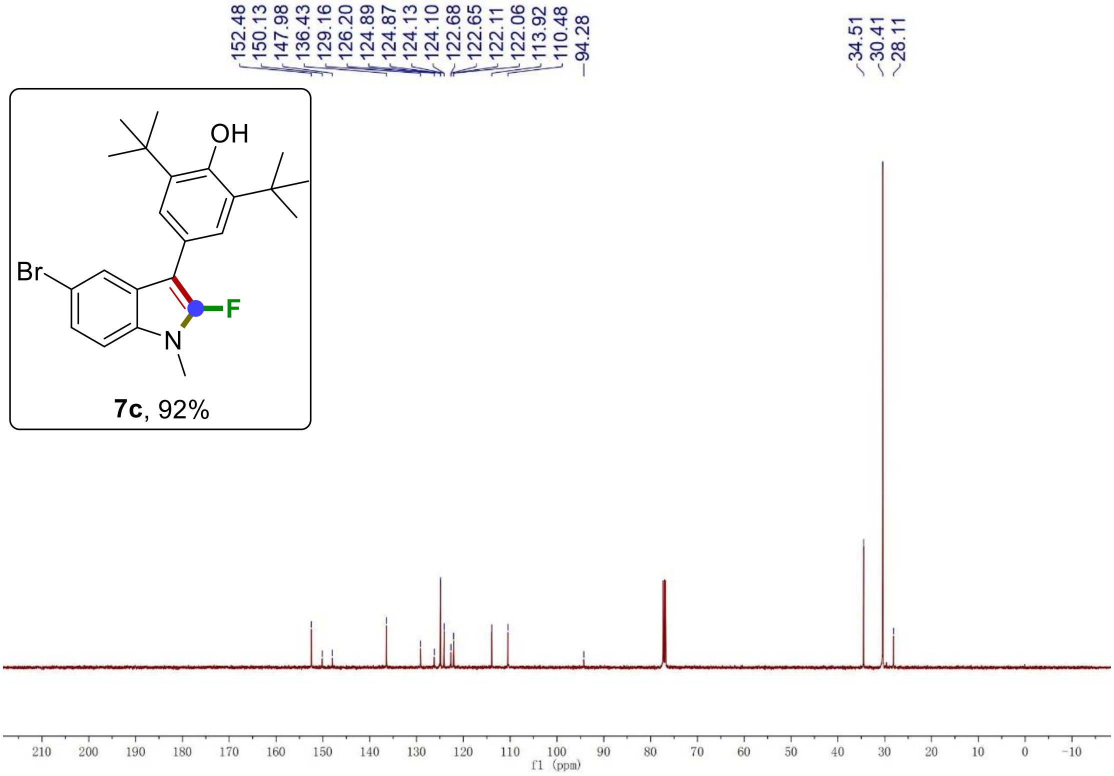

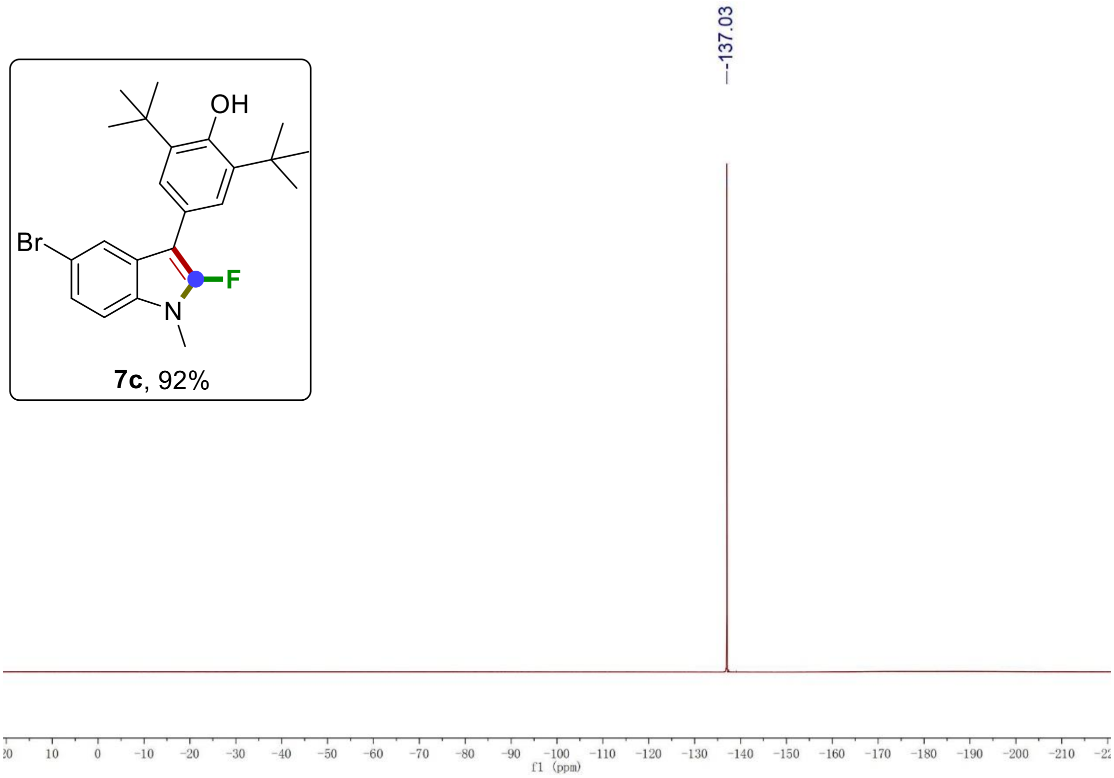


Supplementary Figure 141. $^{1}H$ NMR, $^{13}C$ NMR and $^{19}F$ NMR spectrum of **7c**.

## 2,6-di-tert-butyl-4-(4-chloro-2-fluoro-1-methyl-1H-indol-3-yl)phenol (7d)

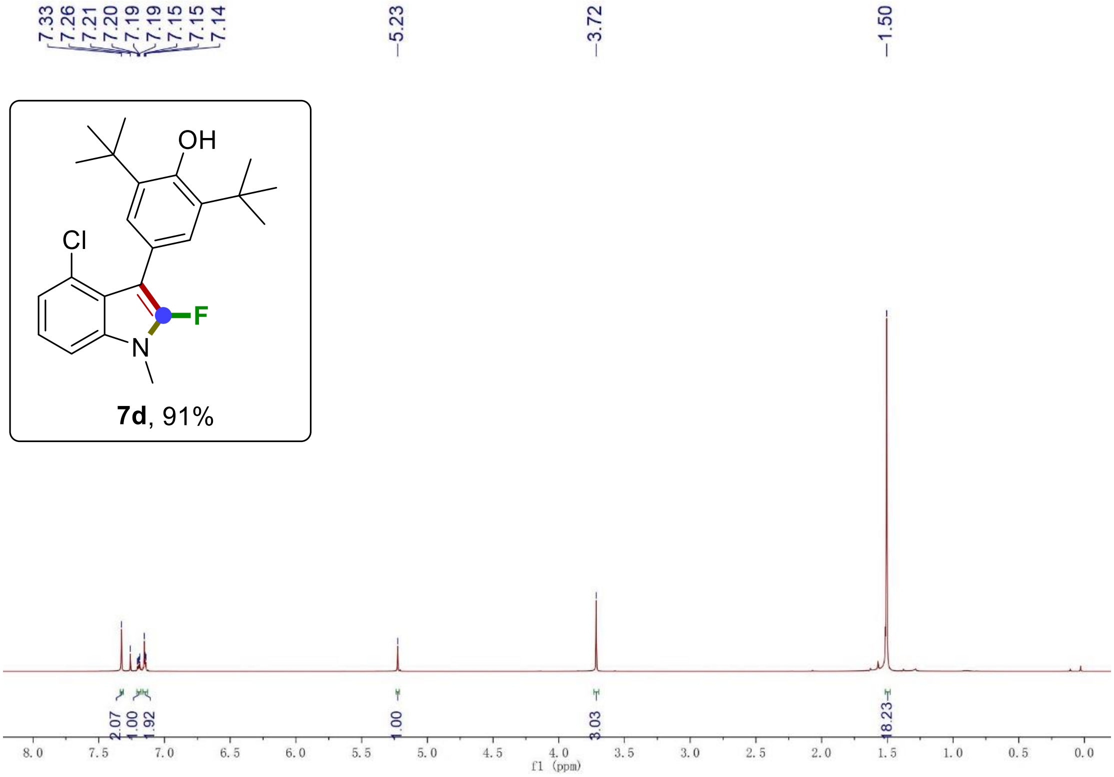


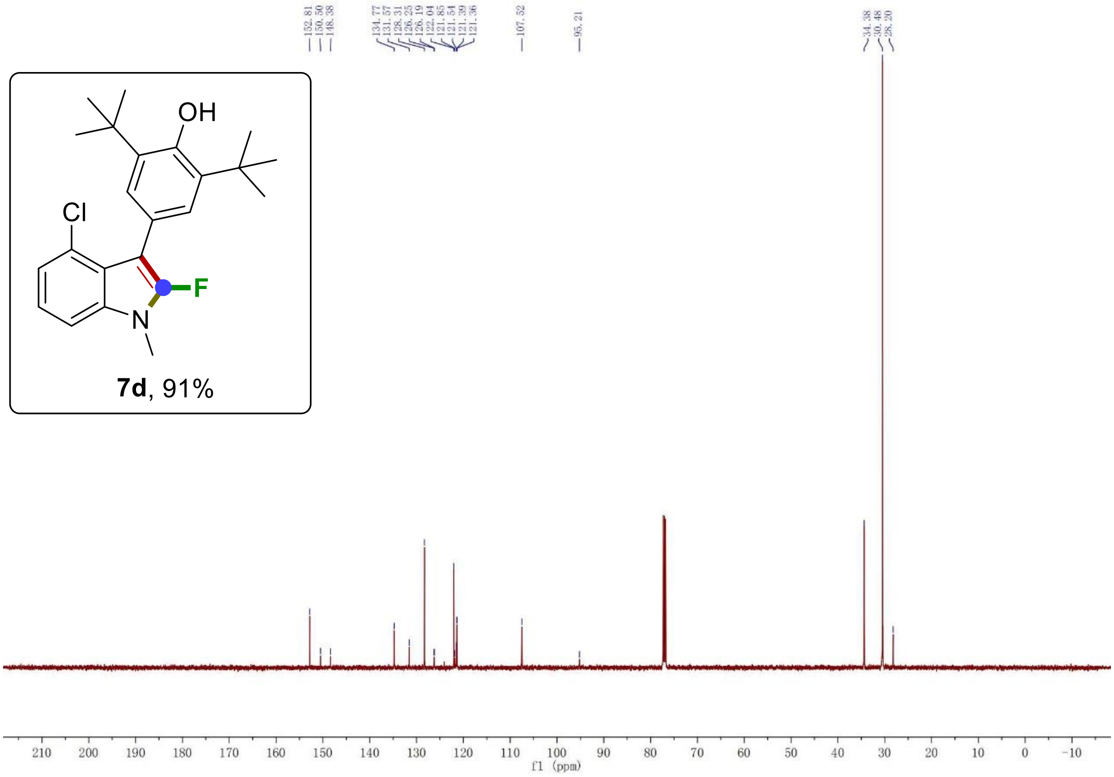

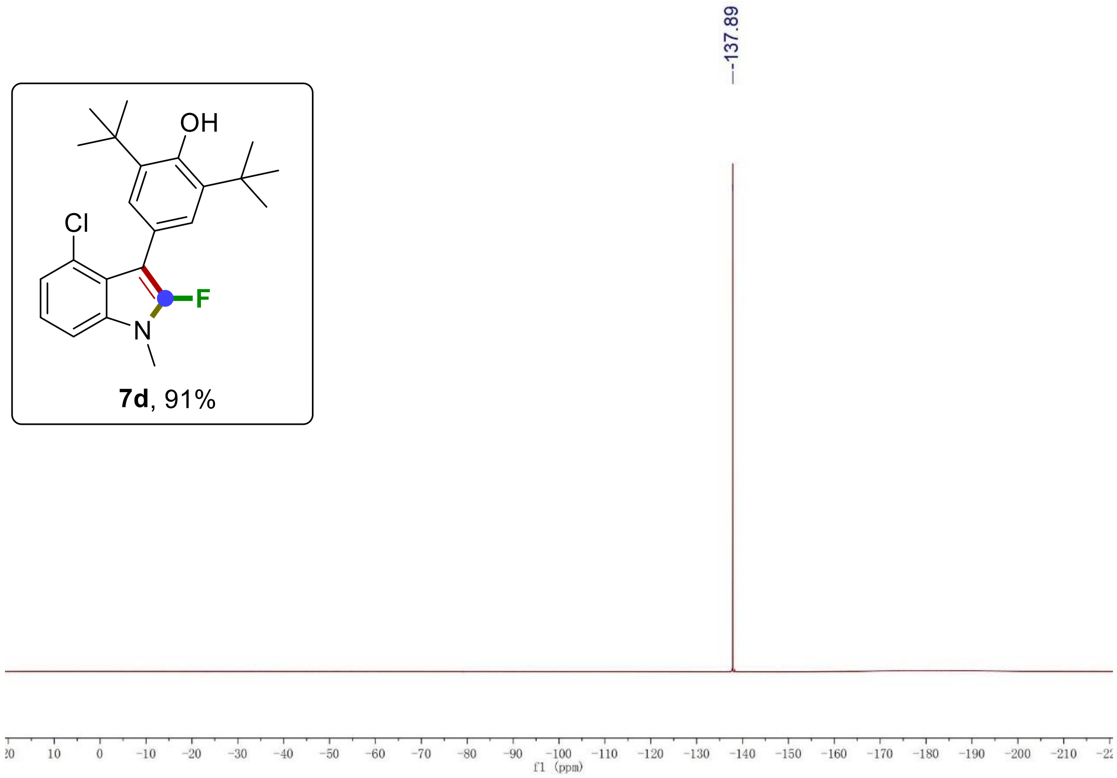


Supplementary Figure 141. $^{1}H$ NMR, $^{13}C$ NMR and $^{19}F$ NMR spectrum of **7d**.

## 2,6-di-tert-butyl-4-(2,7-difluoro-1-methyl-1H-indol-3-yl)phenol (7e)

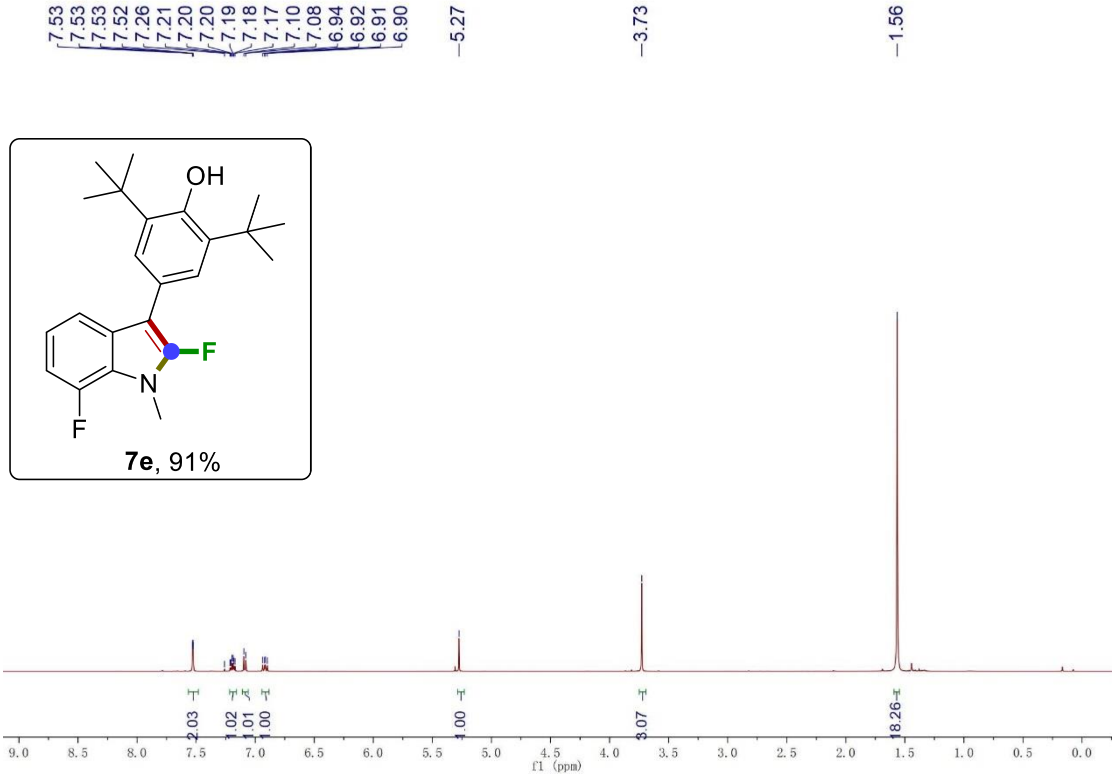


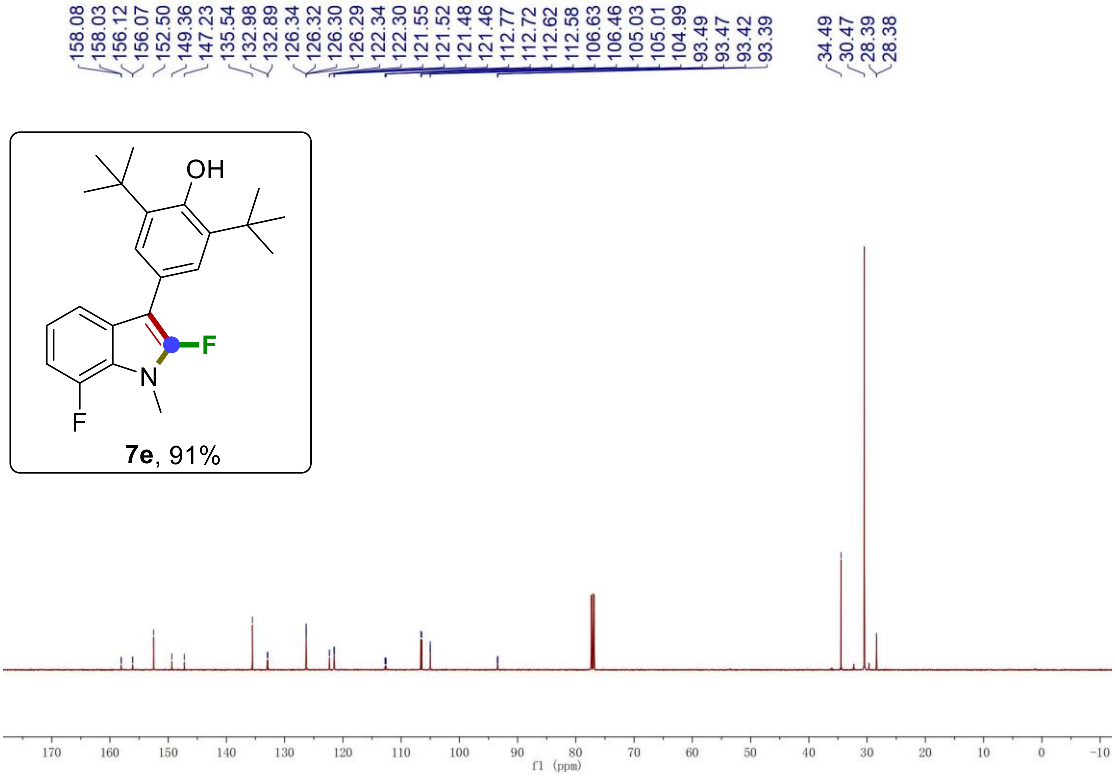

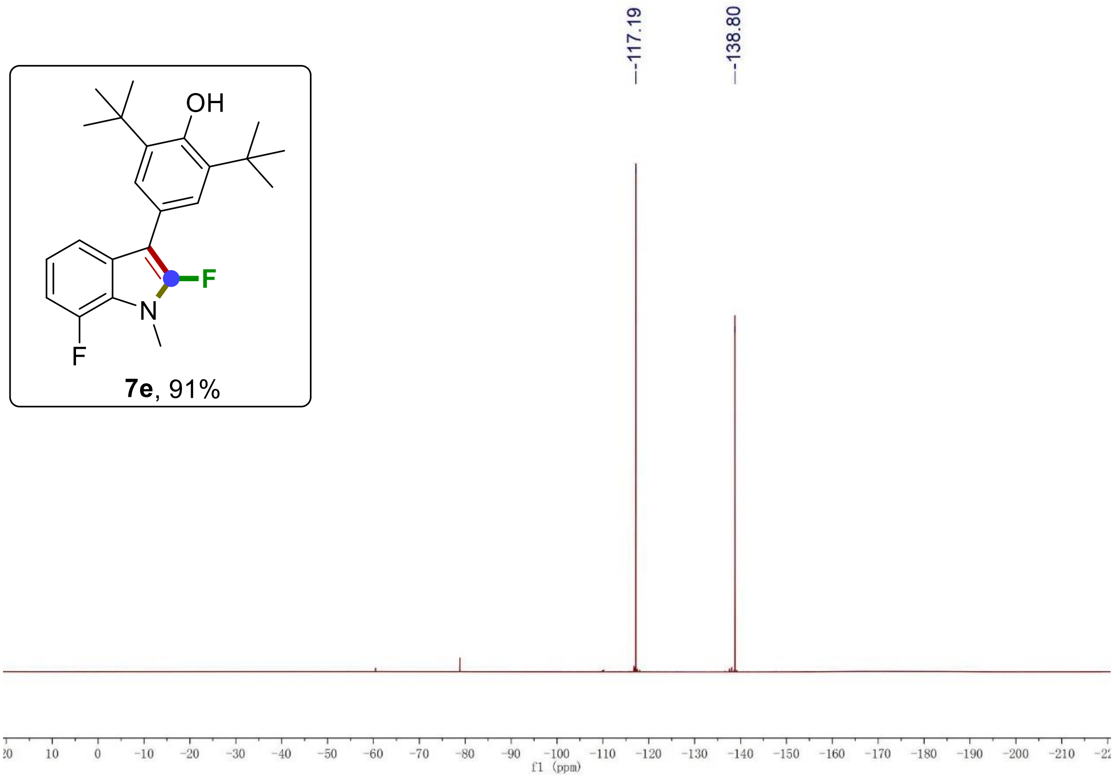


Supplementary Figure 142. $^{1}$H NMR, $^{13}$C NMR and $^{19}$F NMR spectrum of **7e**.

# 2-(1-(3-bromopropyl)-2-fluoro-1H-indol-3-yl)-1-phenylethan-1-one (9a)

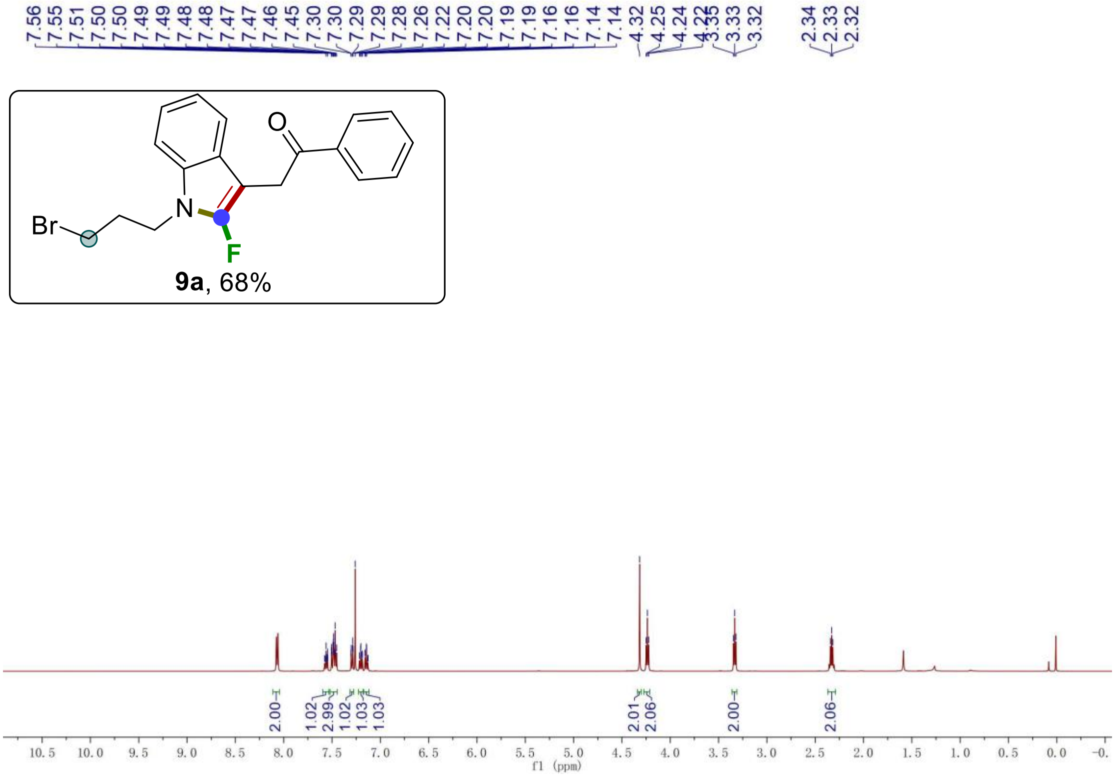


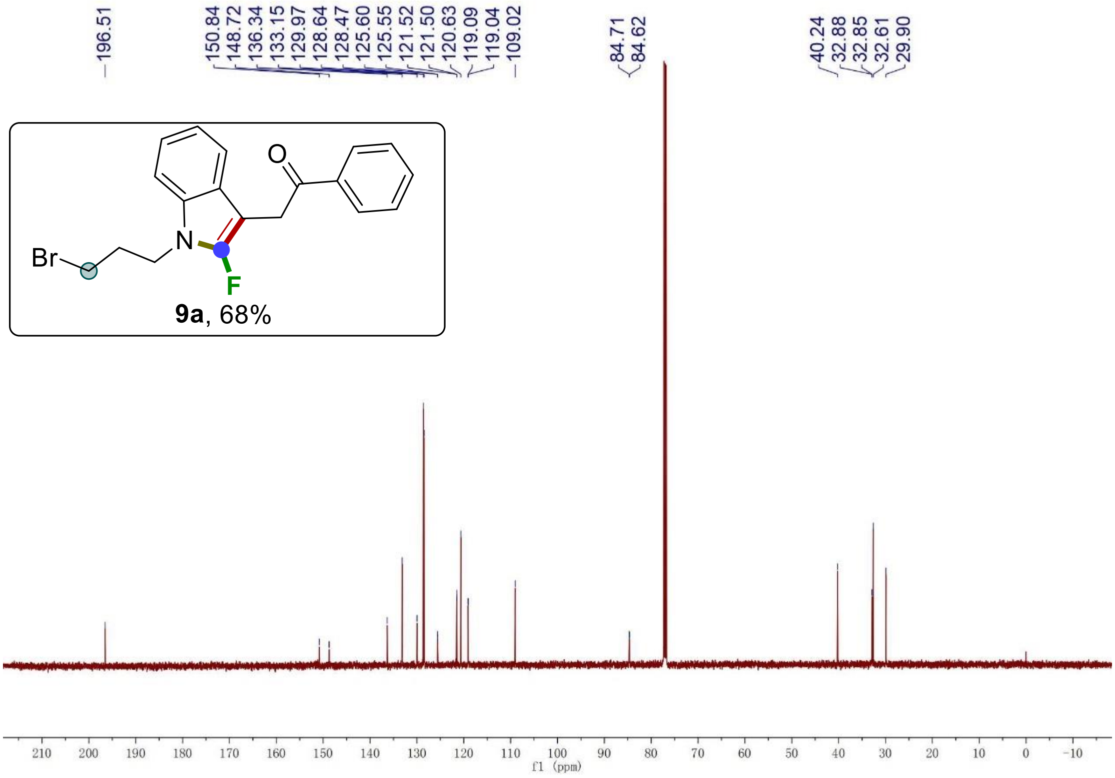

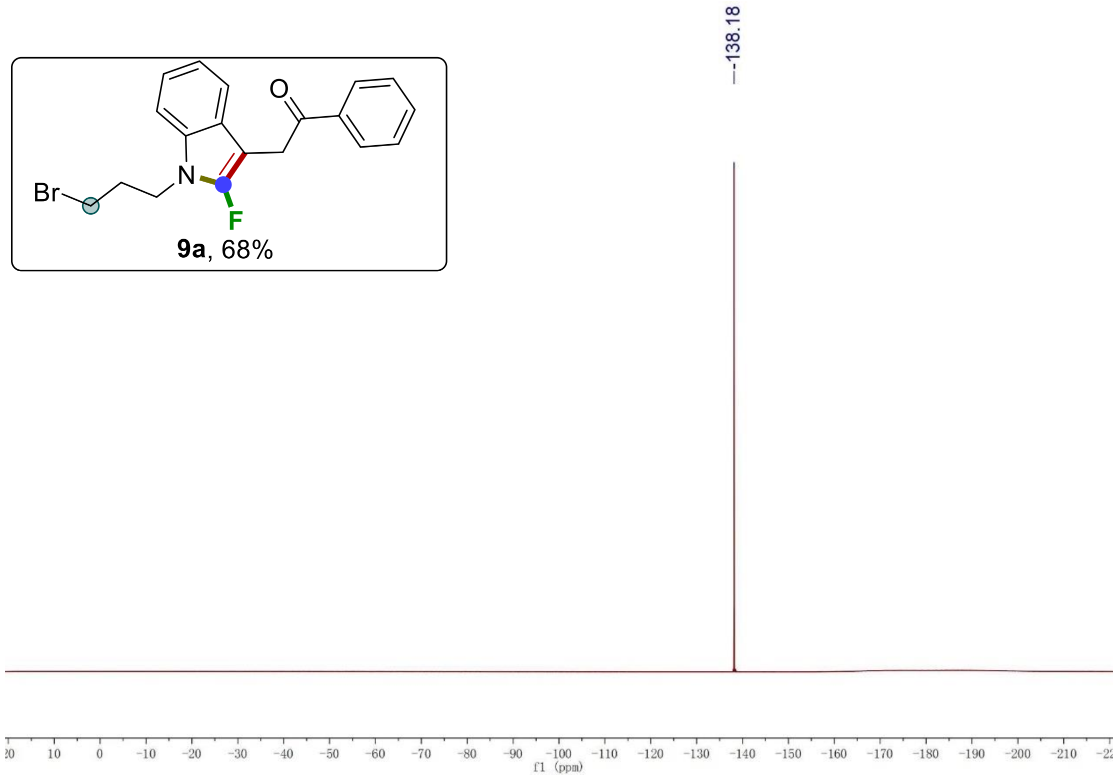


Supplementary Figure 143. $^{1}H$ NMR, $^{13}C$ NMR and $^{19}F$ NMR spectrum of **9a**.

# 2-(1-(4-bromobutyl)-2-fluoro-1H-indol-3-yl)-1-phenylethan-1-one (9b)

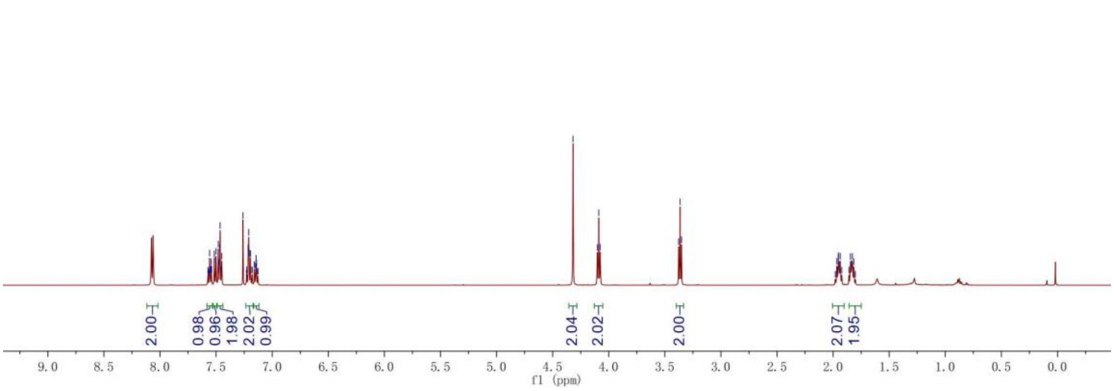


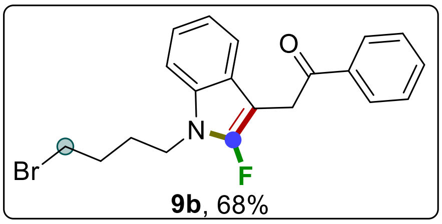


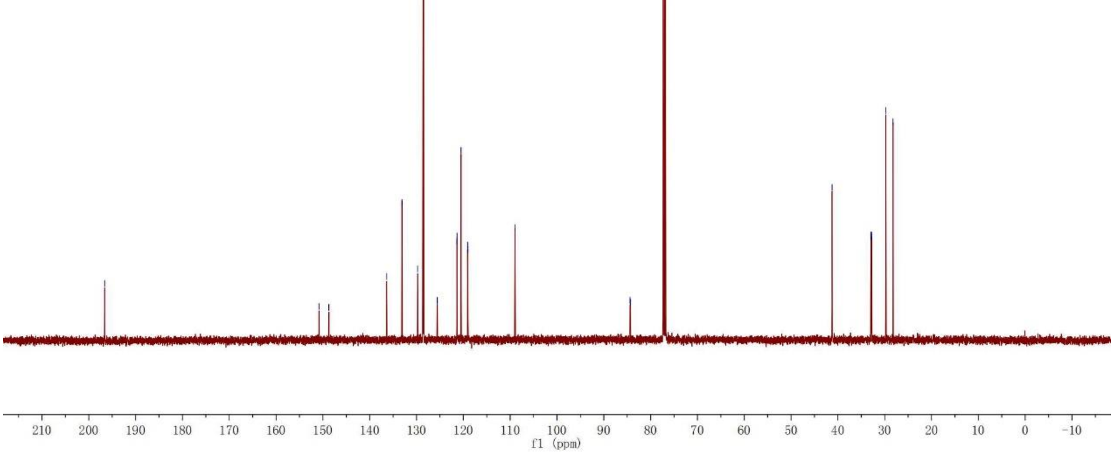


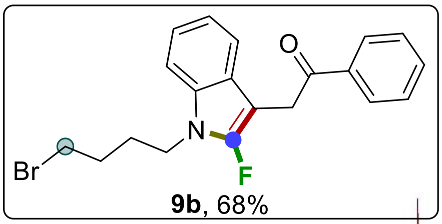

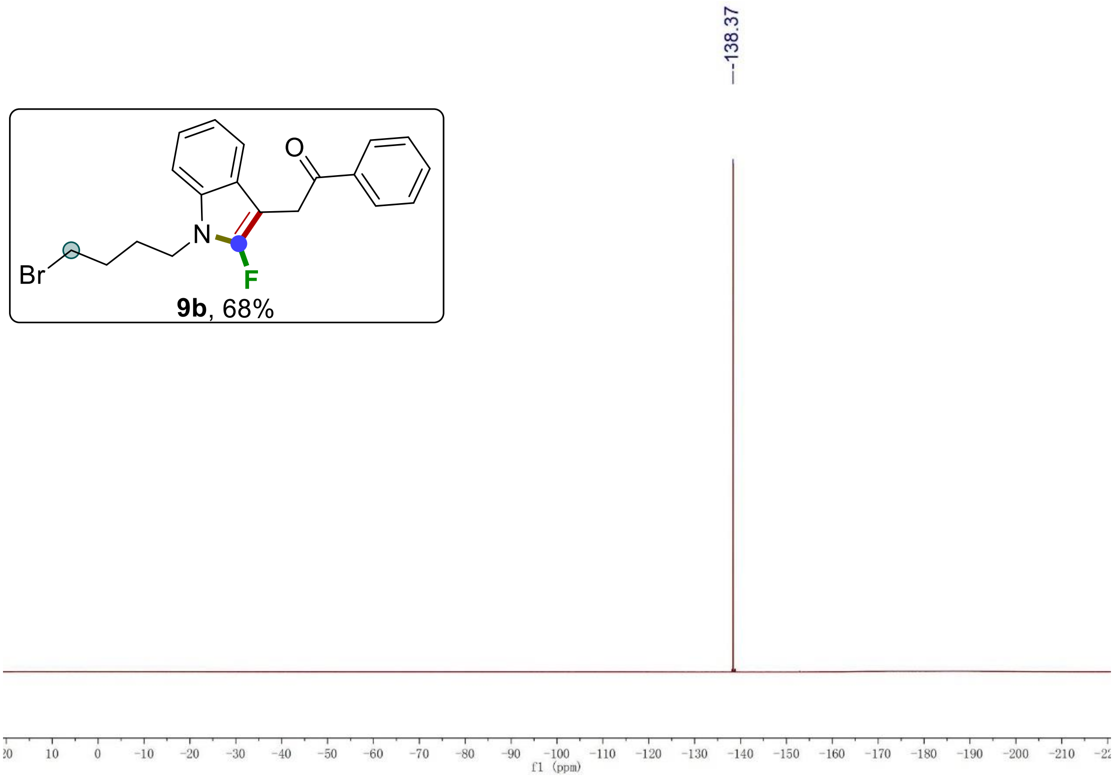


Supplementary Figure 144. $^{1}H$ NMR, $^{13}C$ NMR and $^{19}F$ NMR spectrum of **9b**.

**2-(2-fluoro-1-(4-iodobutyl)-1H-indol-3-yl)-1-phenylethan-1-one (9c)**

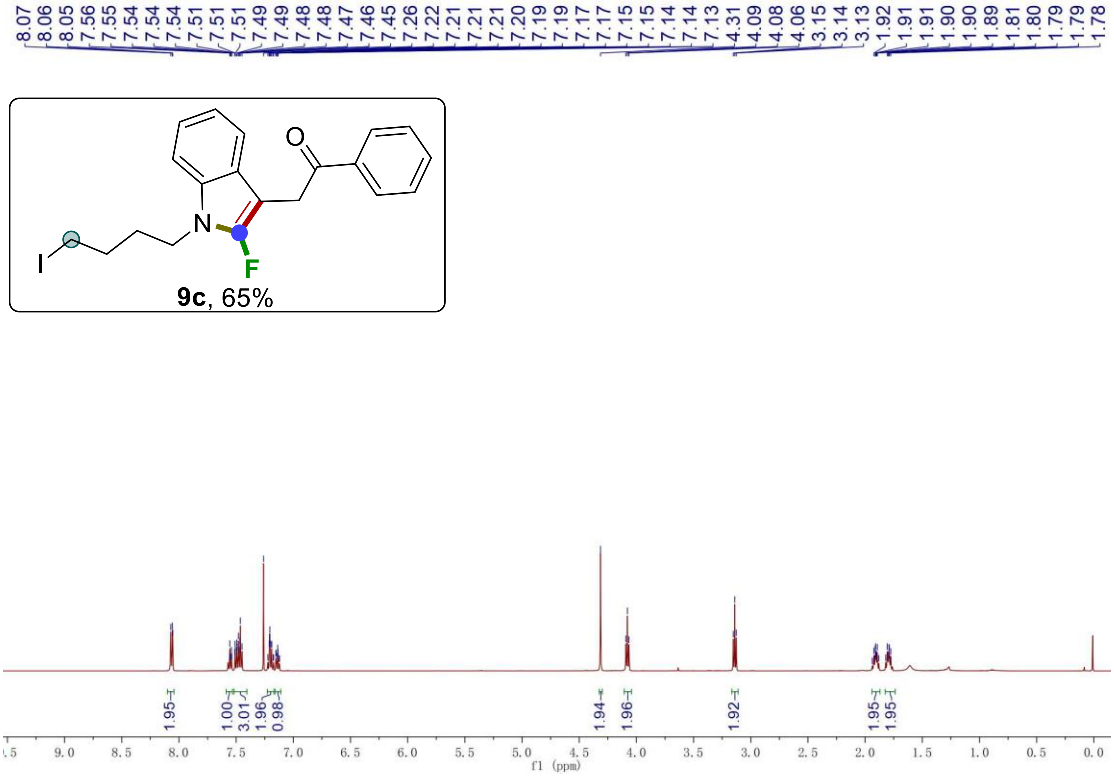

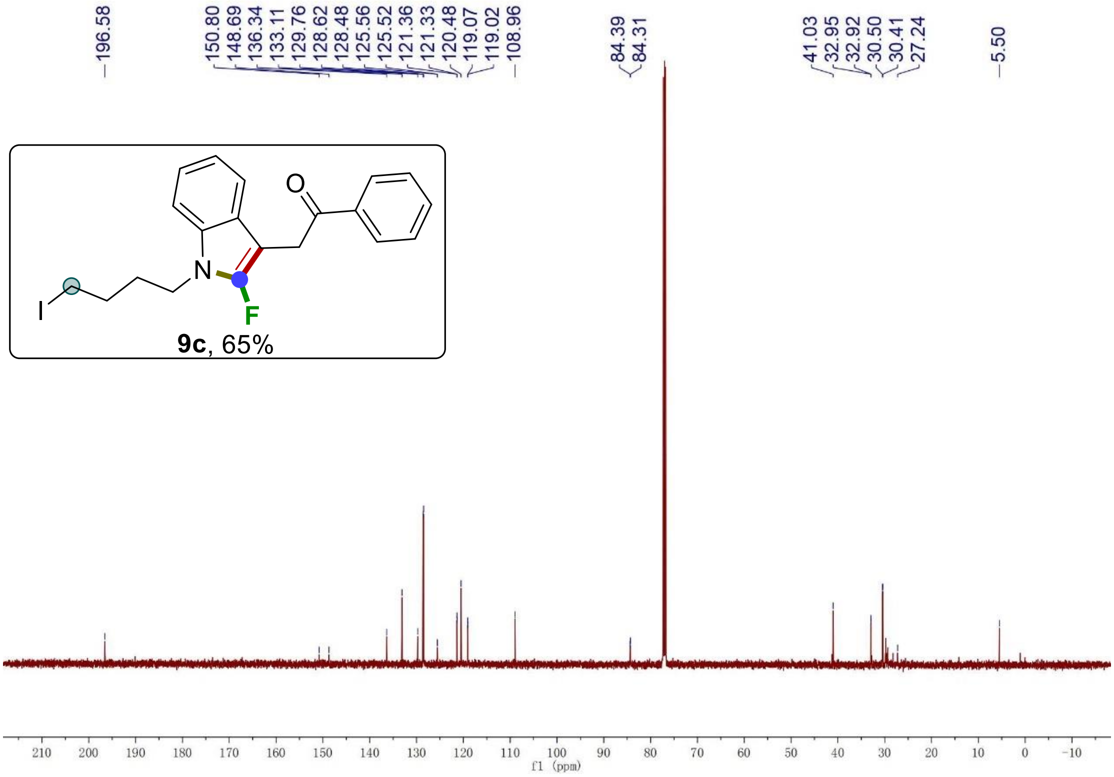

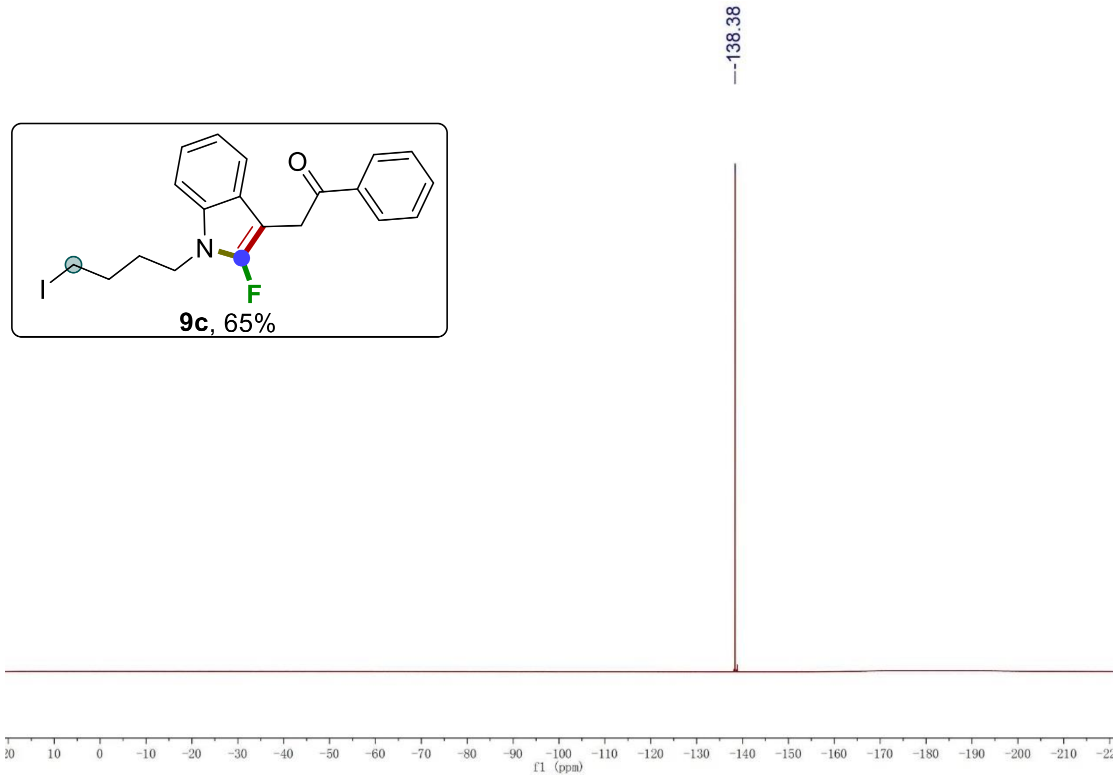


Supplementary Figure 145. $^{1}H$ NMR, $^{13}C$ NMR and $^{19}F$ NMR spectrum of **9c**.

**1-([1,1'-biphenyl]-4-yl)-2-(1-(4-bromobutyl)-2-fluoro-1H-indol-3-yl)ethan-1-one (9d)**

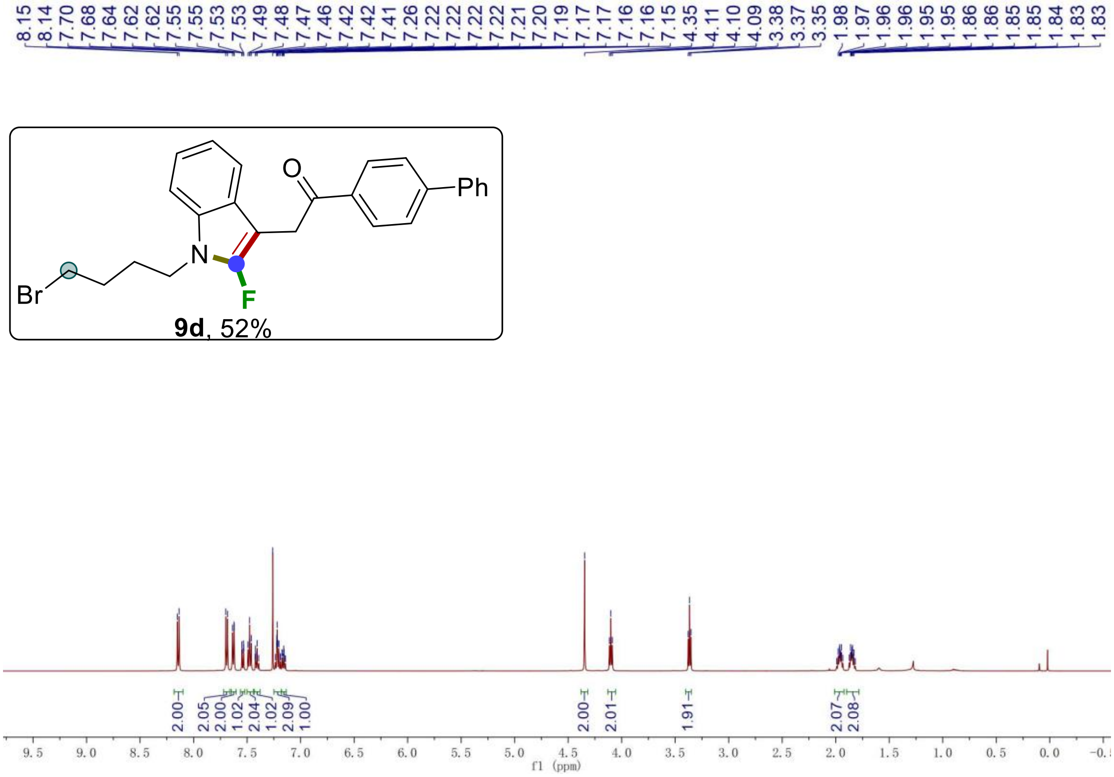


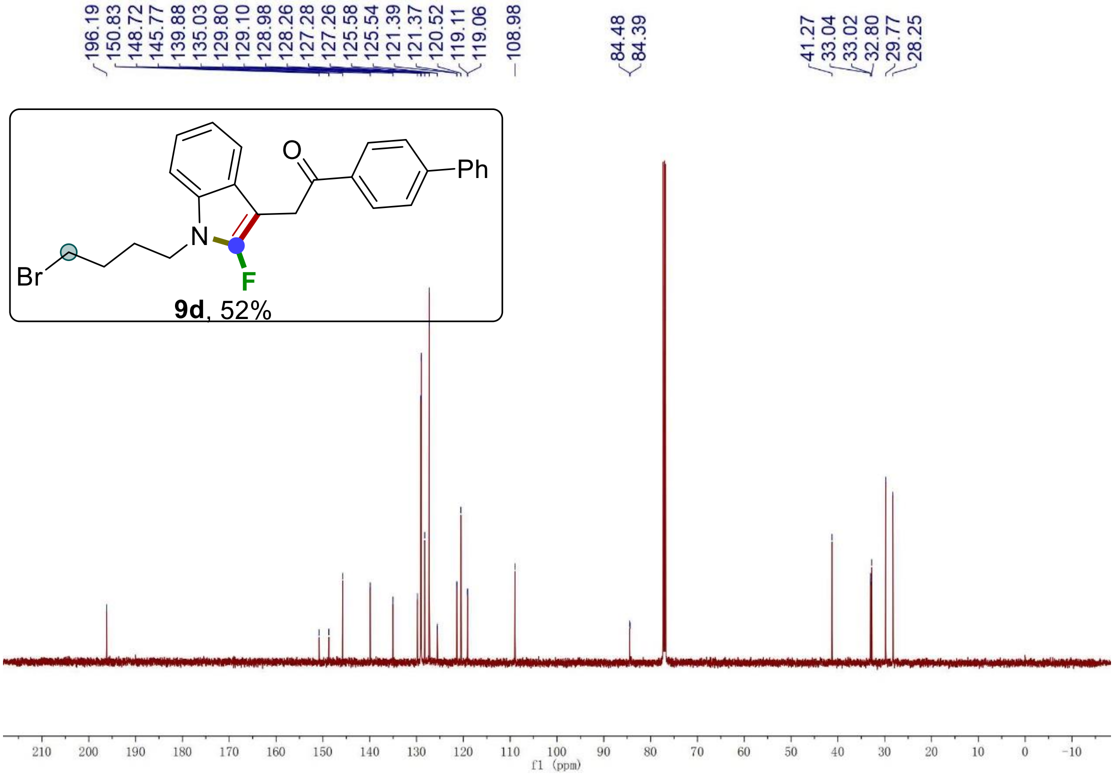

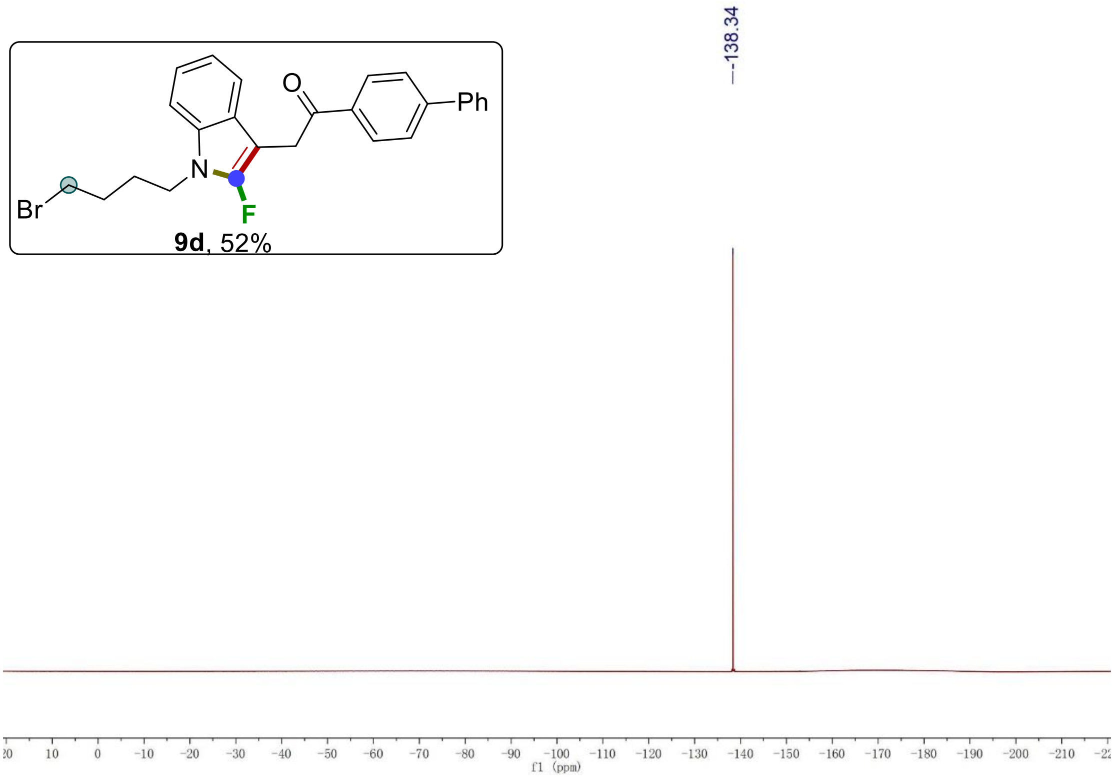


Supplementary Figure 146. $^{1}H$ NMR, $^{13}C$ NMR and $^{19}F$ NMR spectrum of **9d**.

**2-(1-(4-bromobutyl)-2-fluoro-1H-indol-3-yl)-1-(4-isobutylphenyl)ethan-1-one (9e)**

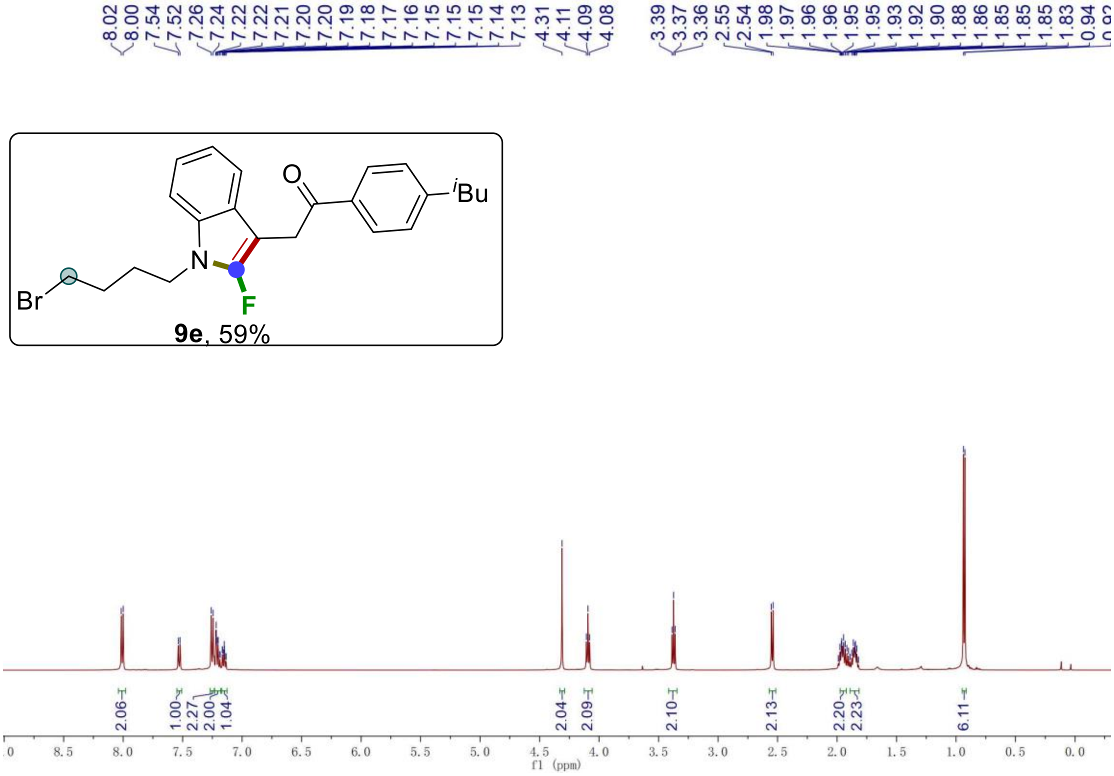


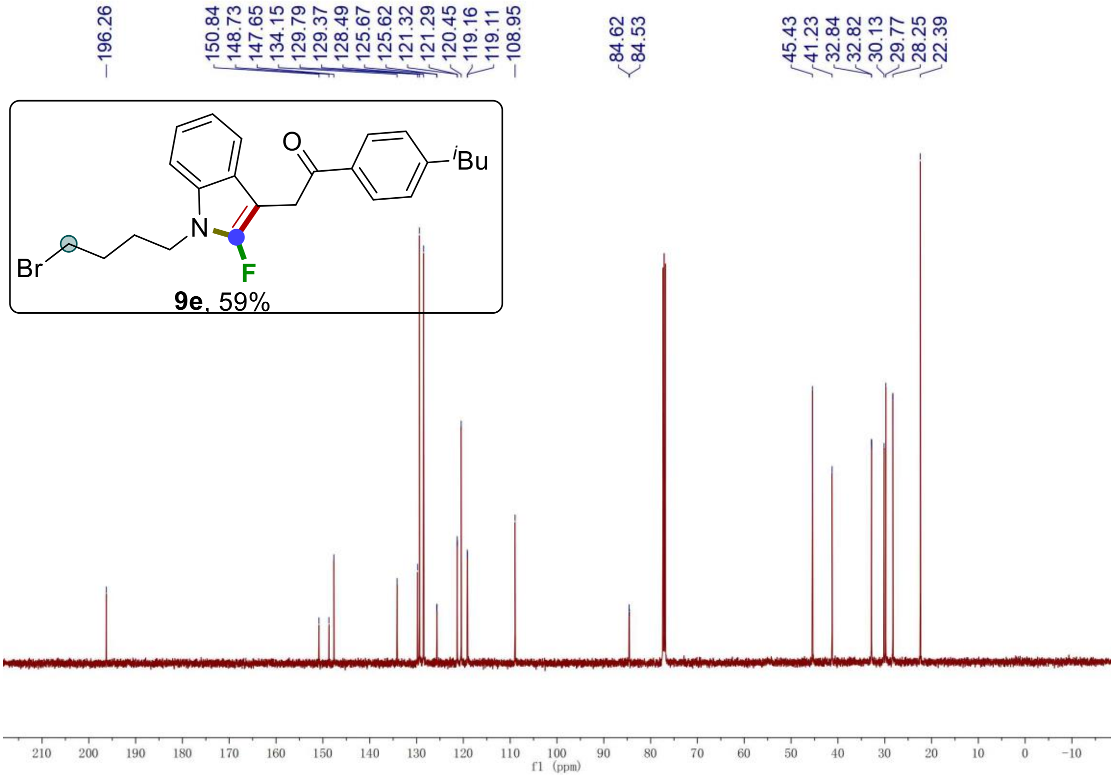

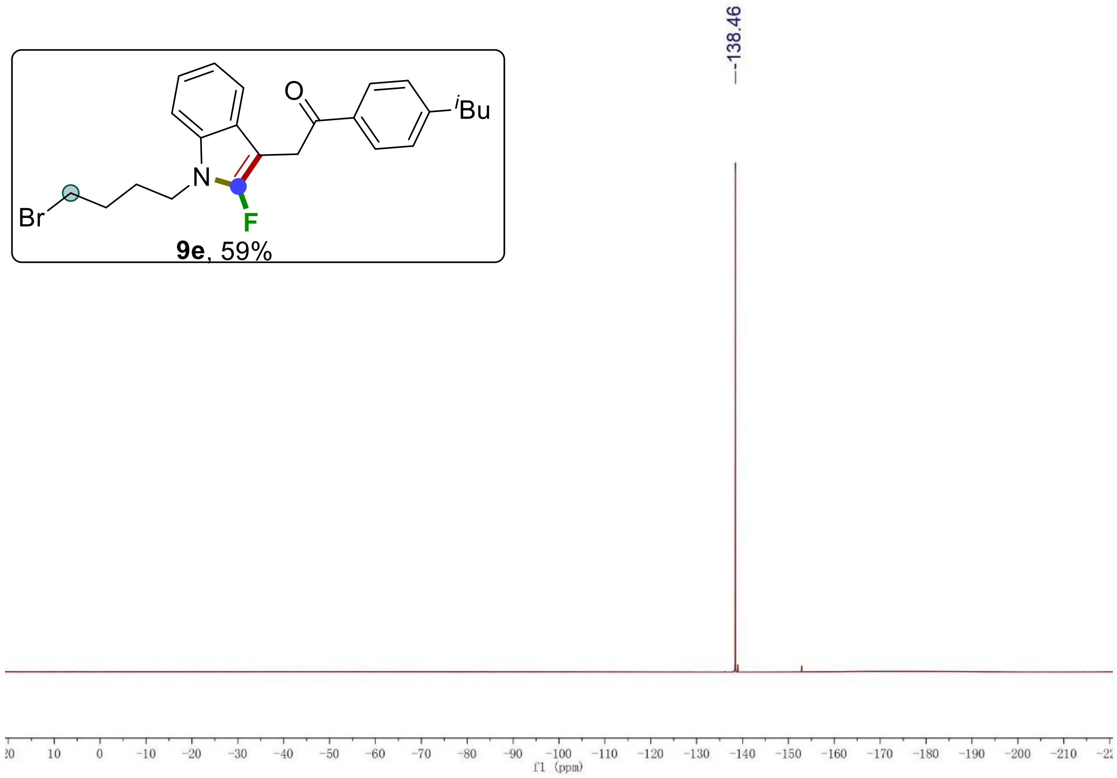


Supplementary Figure 147. $^{1}H$ NMR, $^{13}C$ NMR and $^{19}F$ NMR spectrum of **9e**.

**2-(1-(4-bromobutyl)-2-fluoro-1H-indol-3-yl)-1-(4-methoxyphenyl)ethan-1-one (9f)**

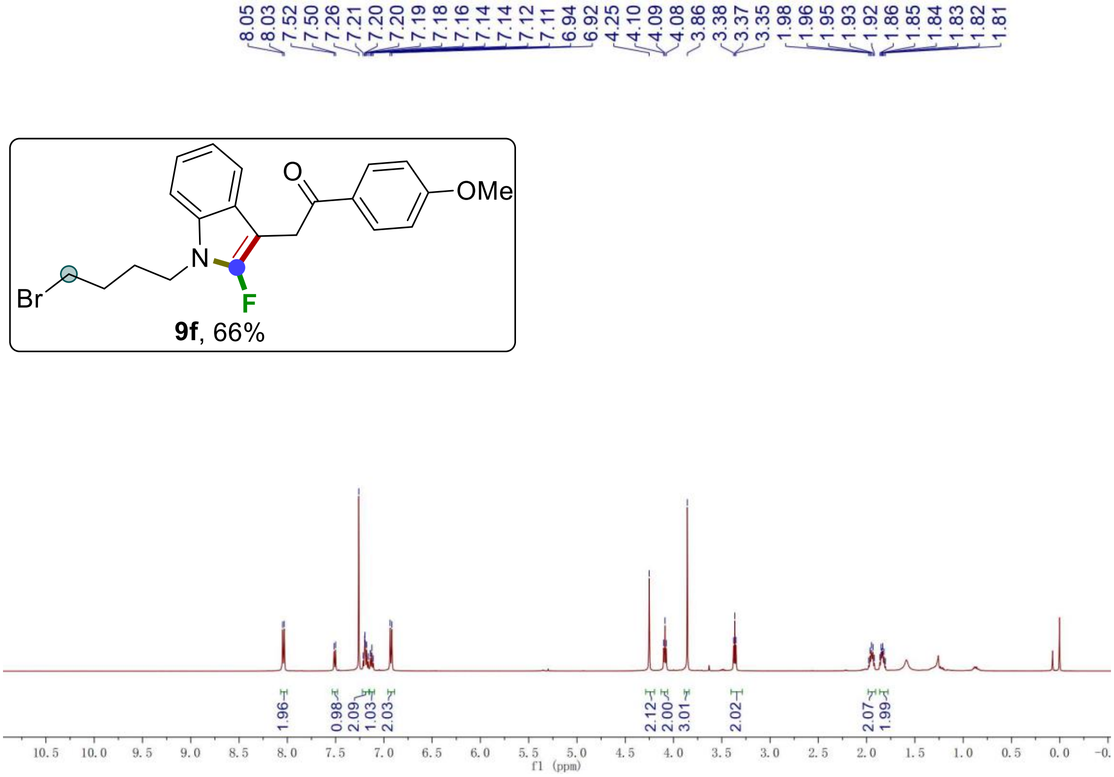


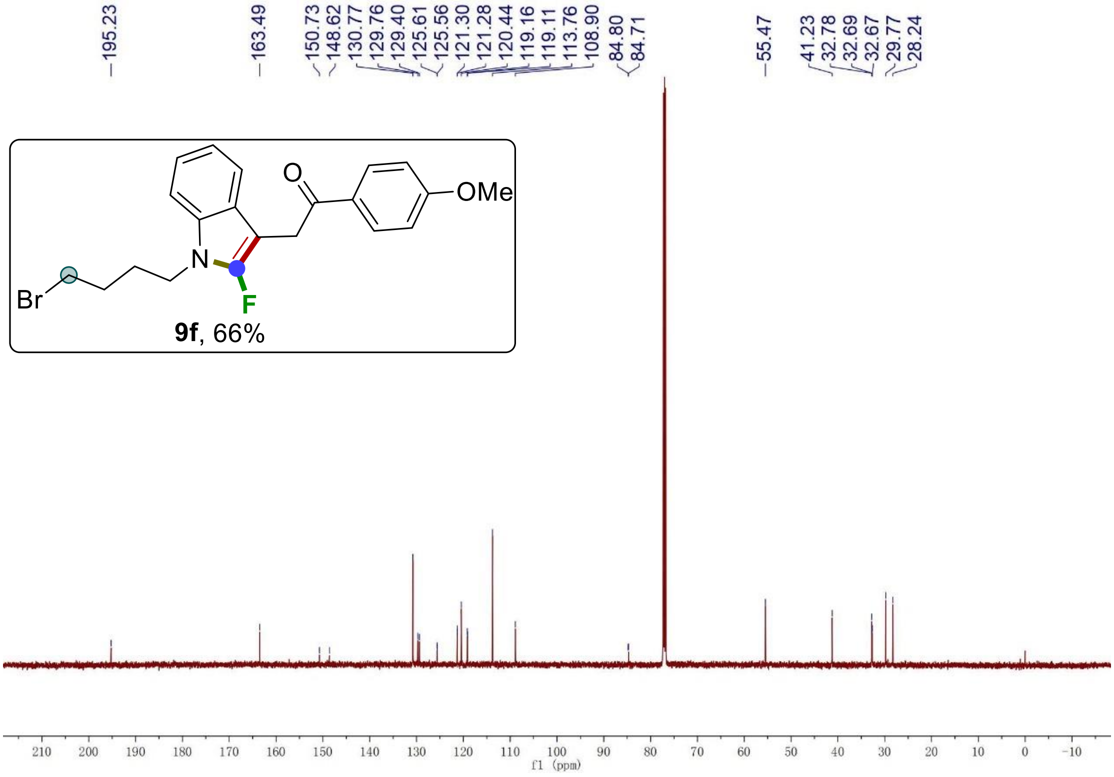

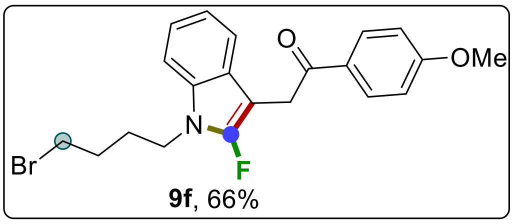


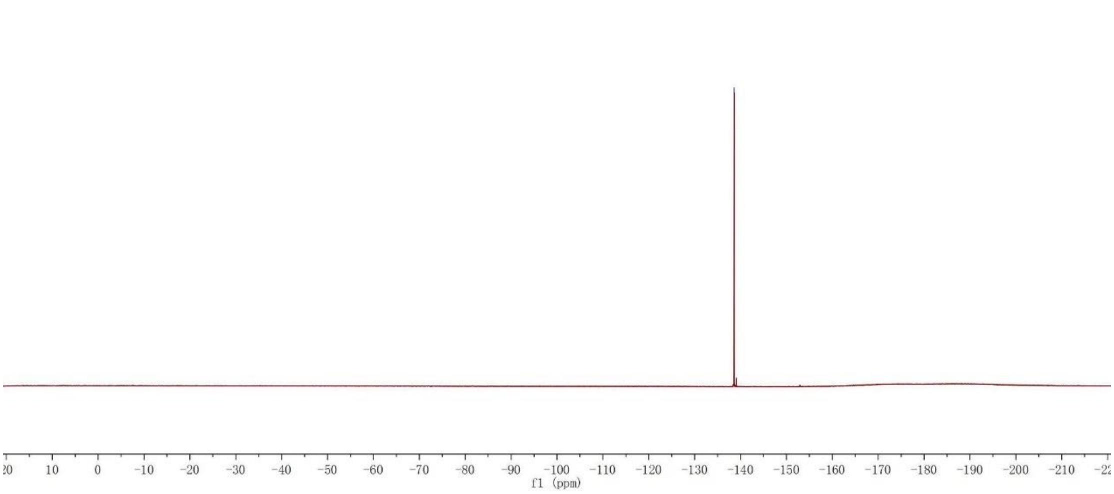


Supplementary Figure 148. $^{1}H$ NMR, $^{13}C$ NMR and $^{19}F$ NMR spectrum of **9f**.

## 2-(1-(4-bromobutyl)-2-fluoro-1H-indol-3-yl)-1-(4-bromophenyl)ethan-1-one (9g)

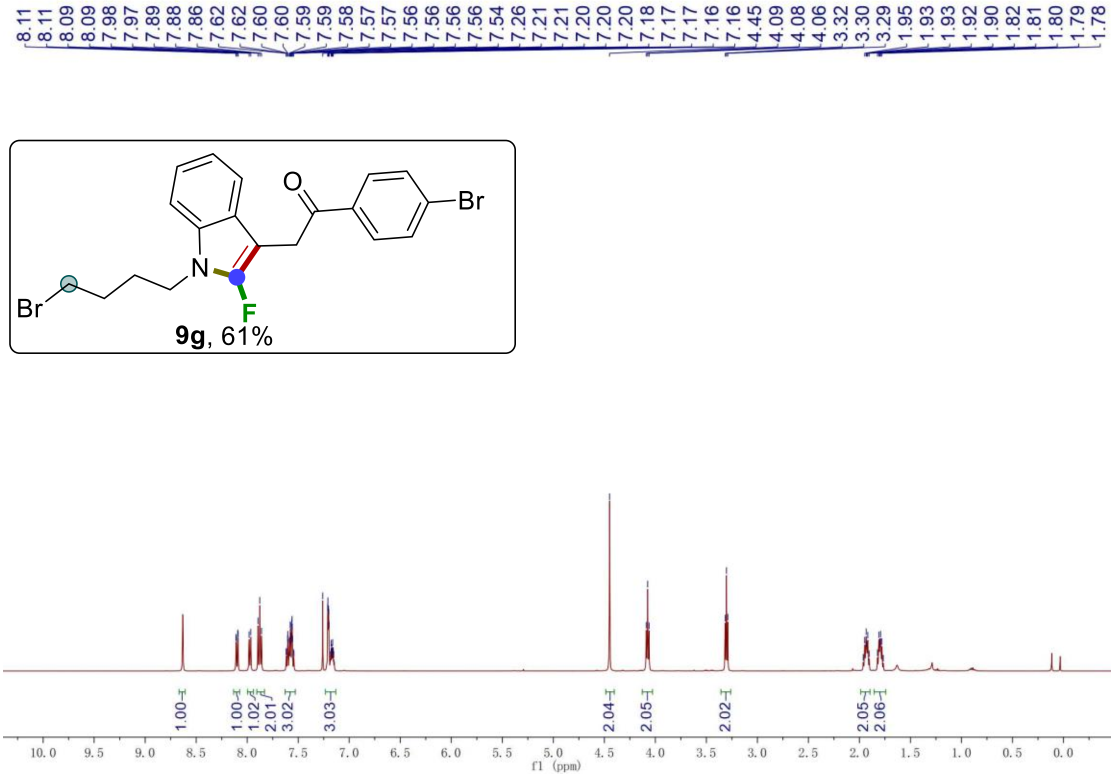


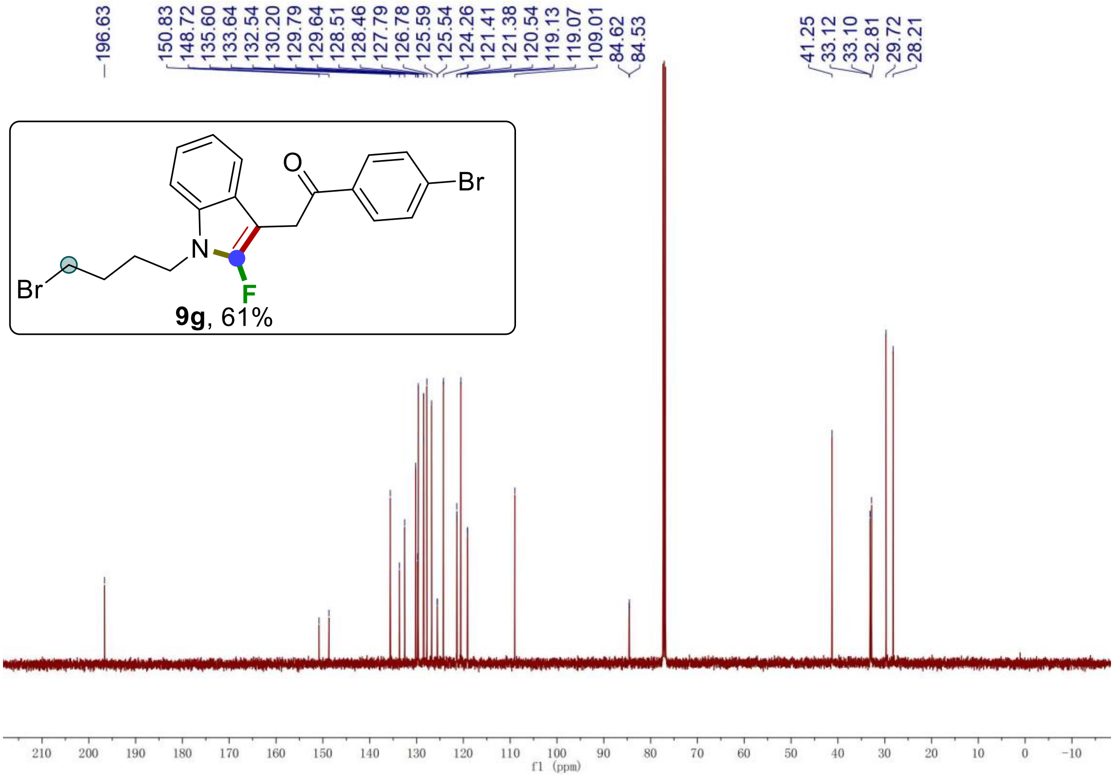

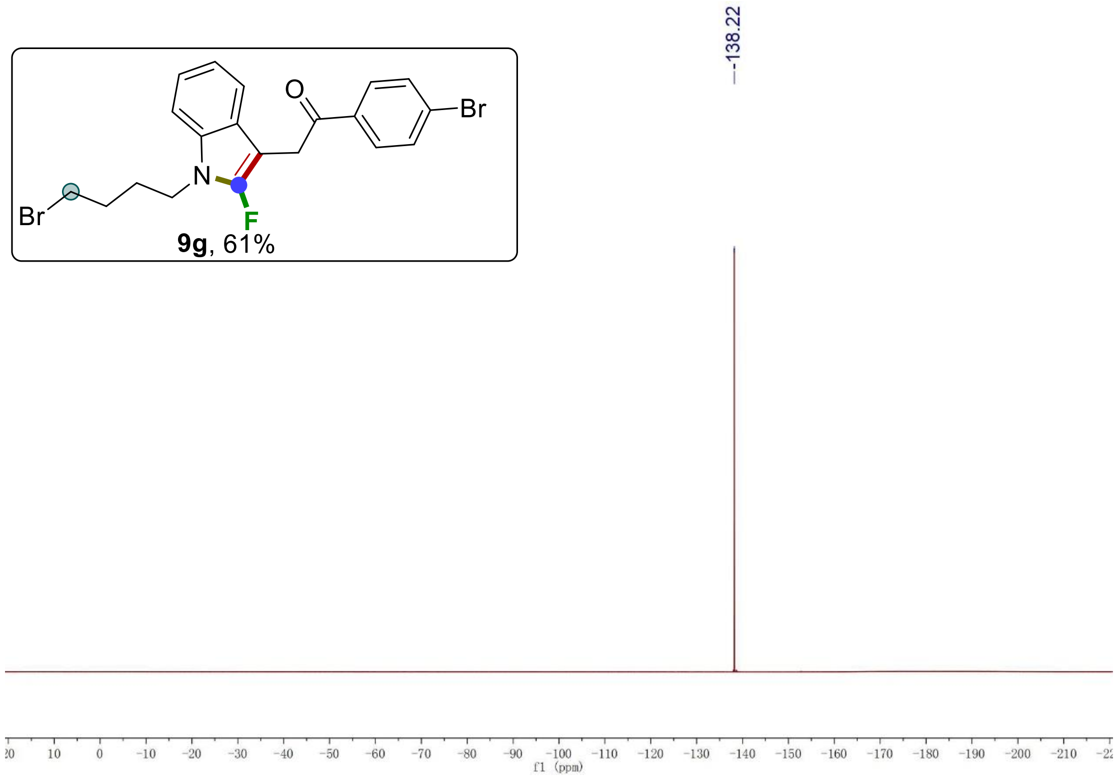


Supplementary Figure 149. $^{1}H$ NMR, $^{13}C$ NMR and $^{19}F$ NMR spectrum of **9g**.

## 2-(1-(4-bromobutyl)-2-fluoro-1H-indol-3-yl)-1-(4-iodophenyl)ethan-1-one (9h)

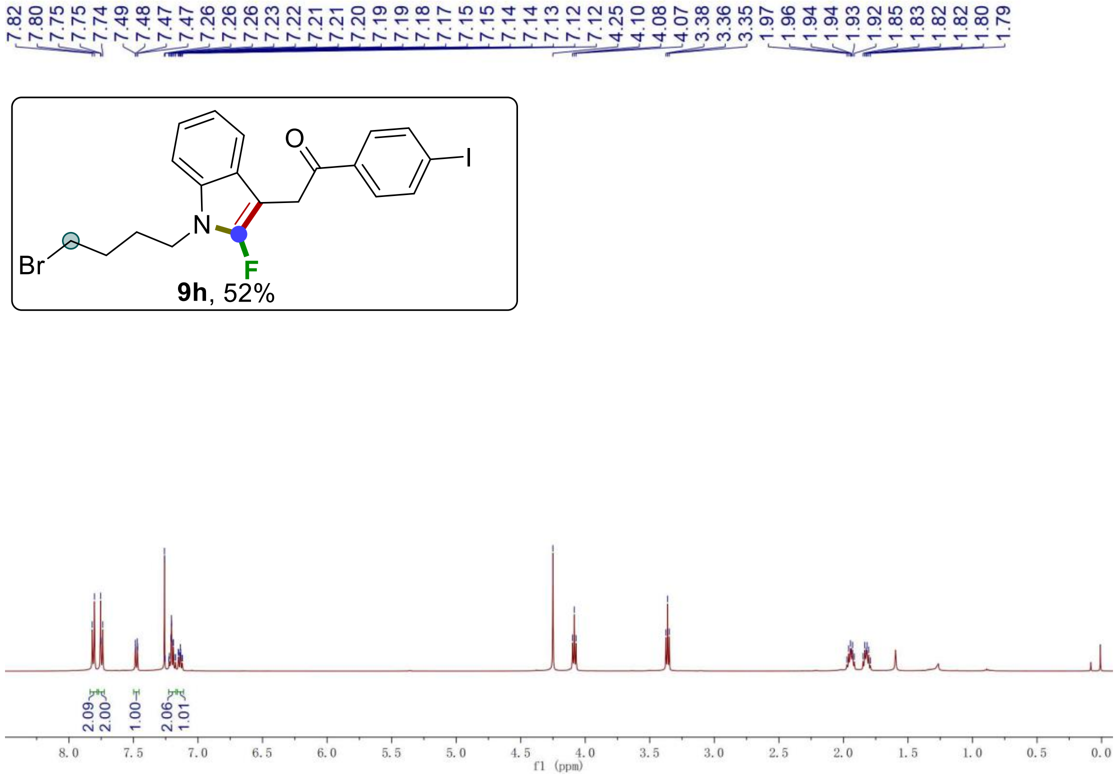


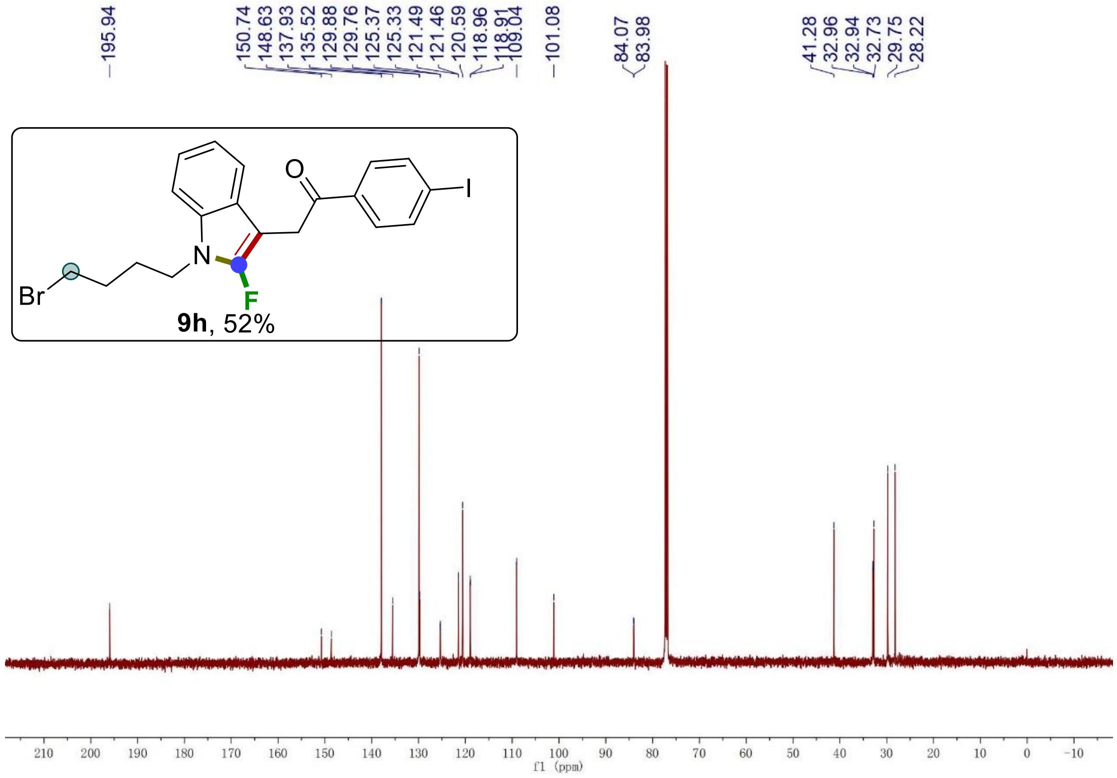

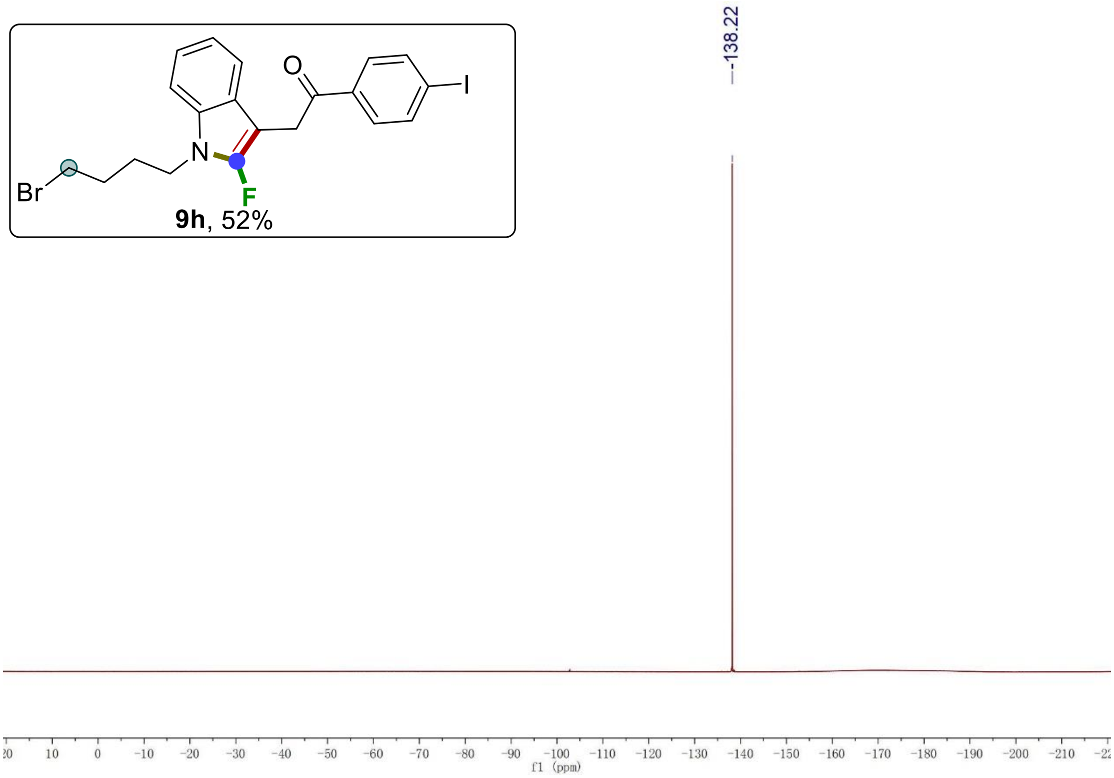


Supplementary Figure 150. $^{1}H$ NMR, $^{13}C$ NMR and $^{19}F$ NMR spectrum of **9h**.

## 2-(1-(4-bromobutyl)-2-fluoro-1H-indol-3-yl)-1-(o-tolyl)ethan-1-one (9i)

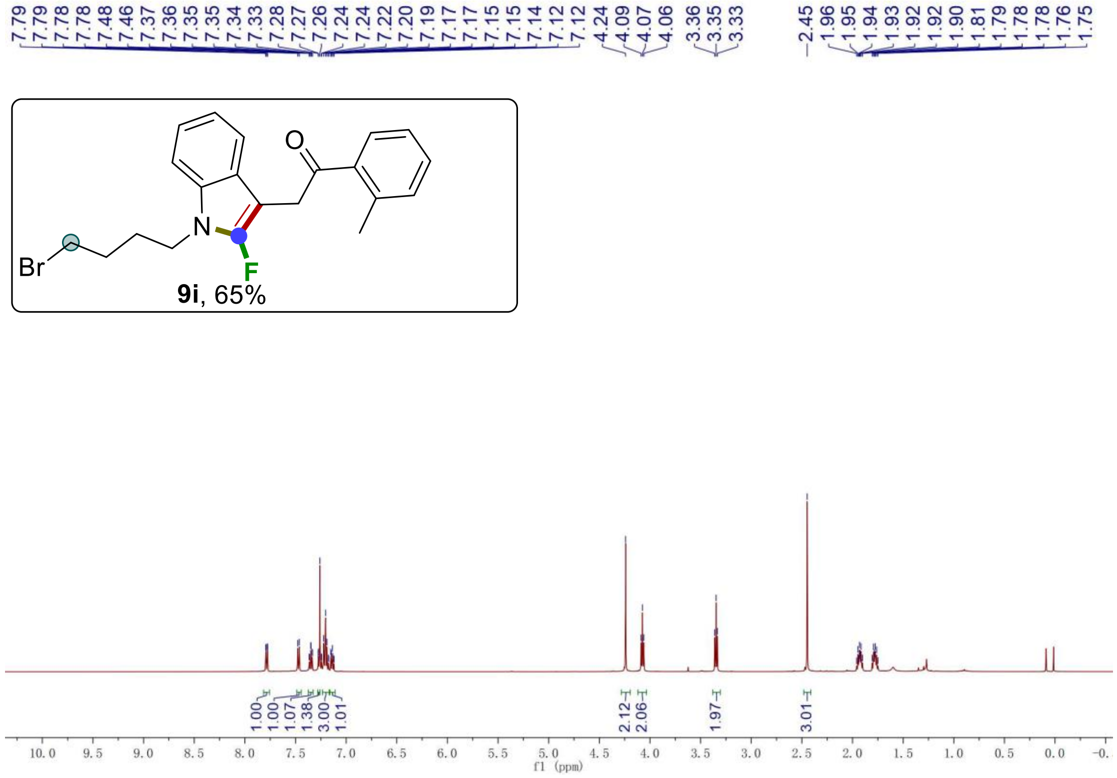


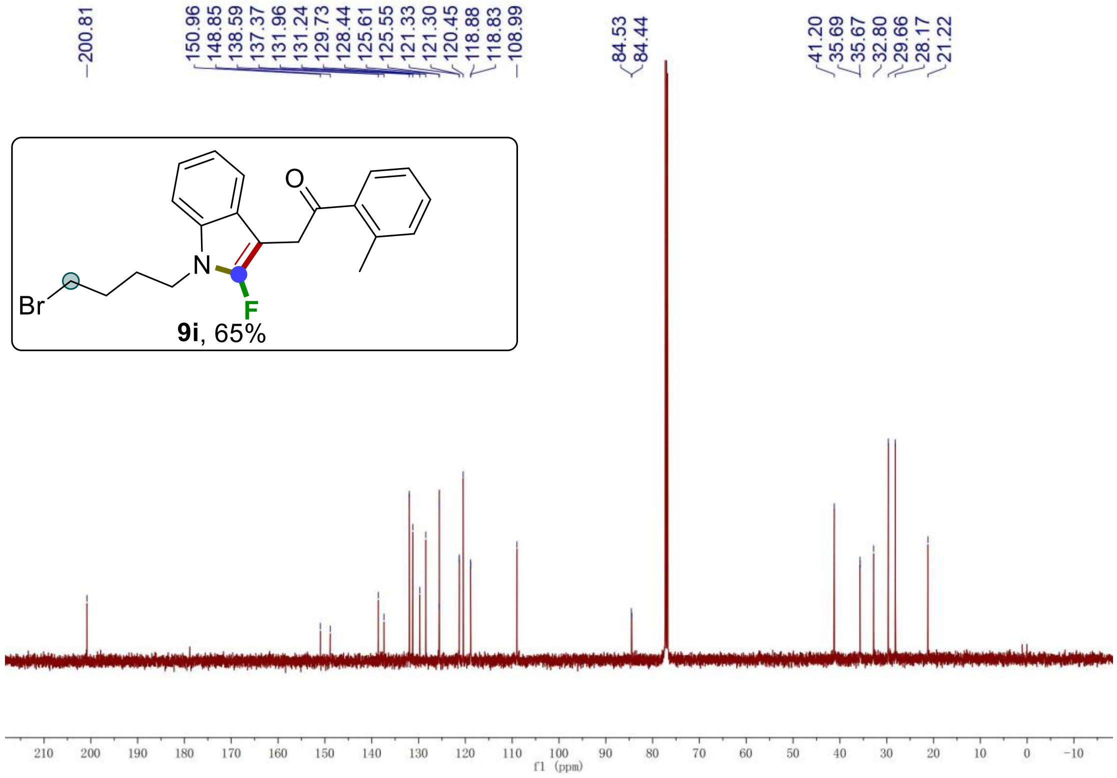

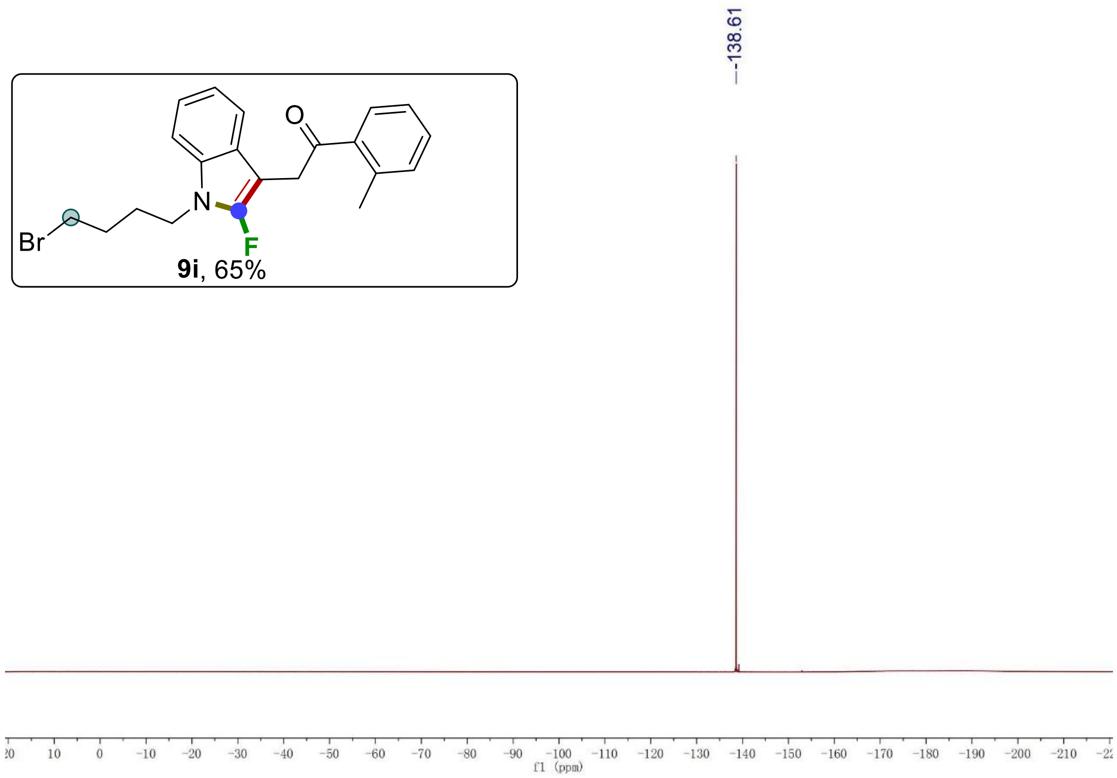


Supplementary Figure 151. $^{1}H$ NMR, $^{13}C$ NMR and $^{19}F$ NMR spectrum of **9i**.

**2-(1-(4-bromobutyl)-2-fluoro-1H-indol-3-yl)-1-(2-(trifluoromethyl)phenyl)ethan-1-one (9j)**

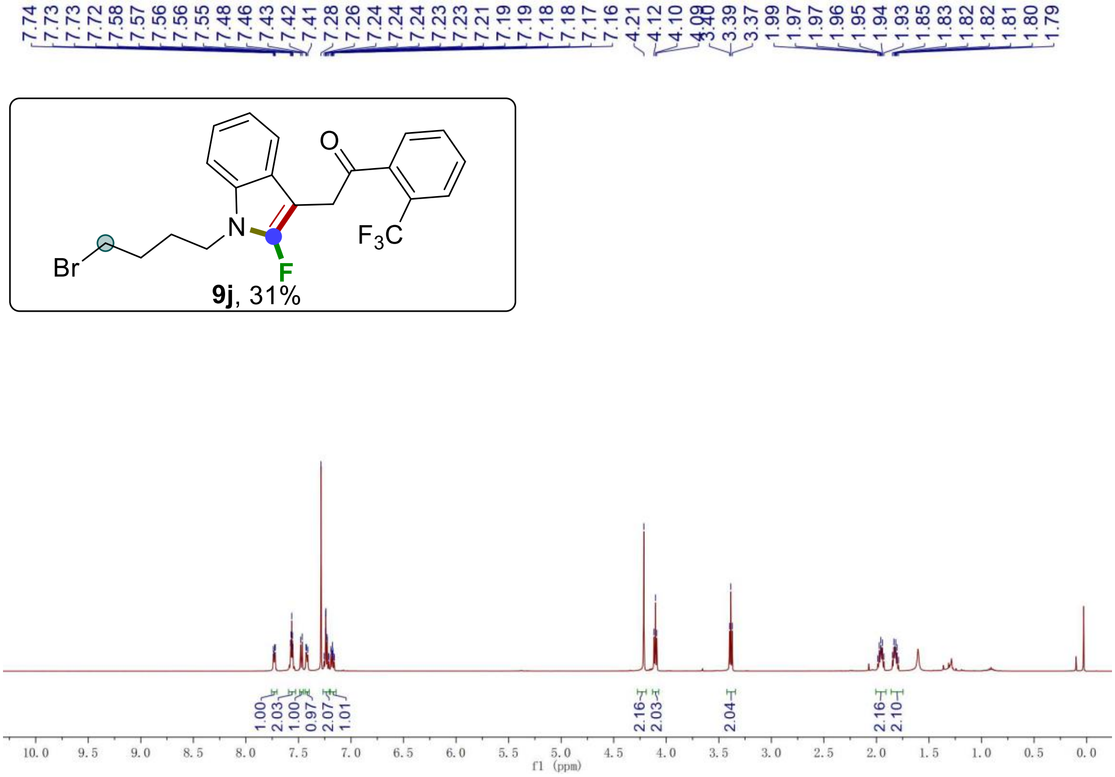


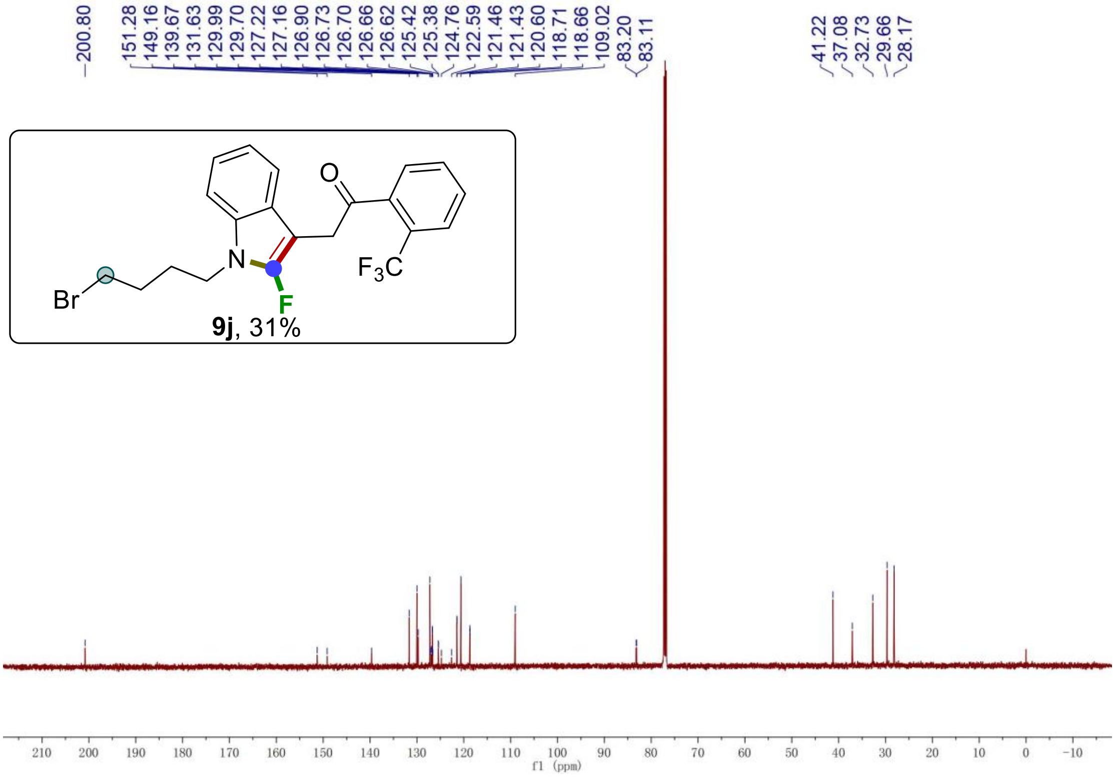

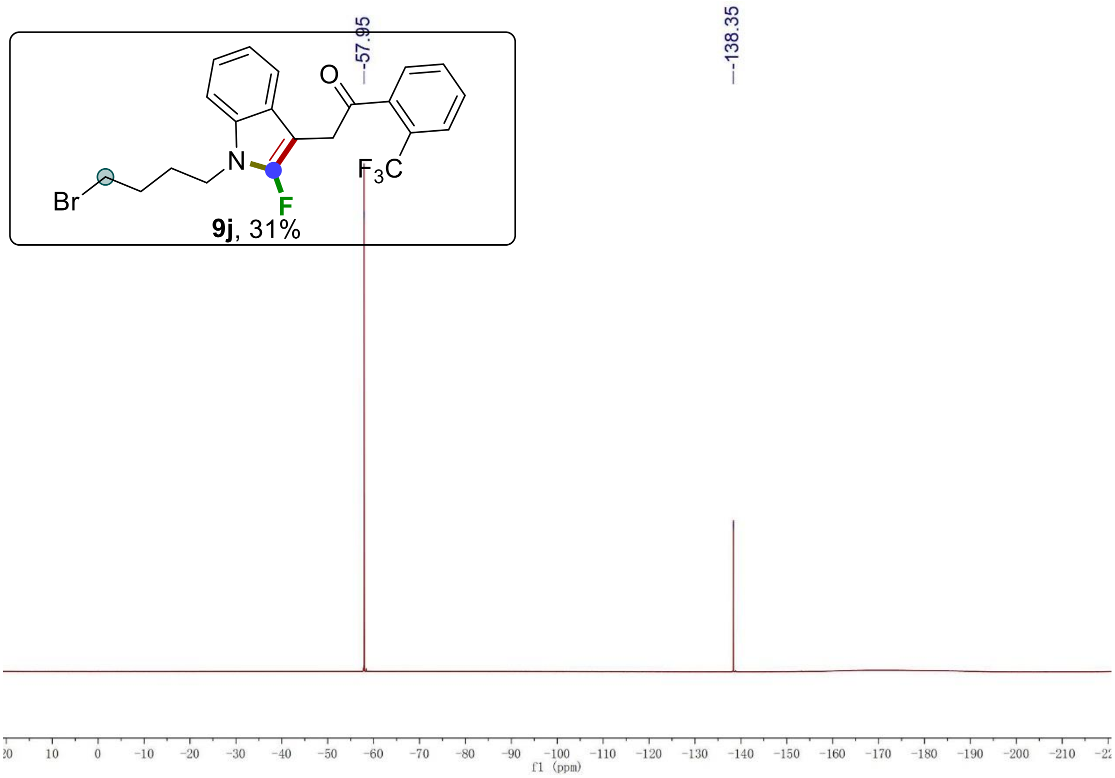


Supplementary Figure 152. $^{1}$H NMR, $^{13}$C NMR and $^{19}$F NMR spectrum of **9j**.

## 2-(1-(4-bromobutyl)-2-fluoro-1H-indol-3-yl)-1-(3-chlorophenyl)ethan-1-one (9k)

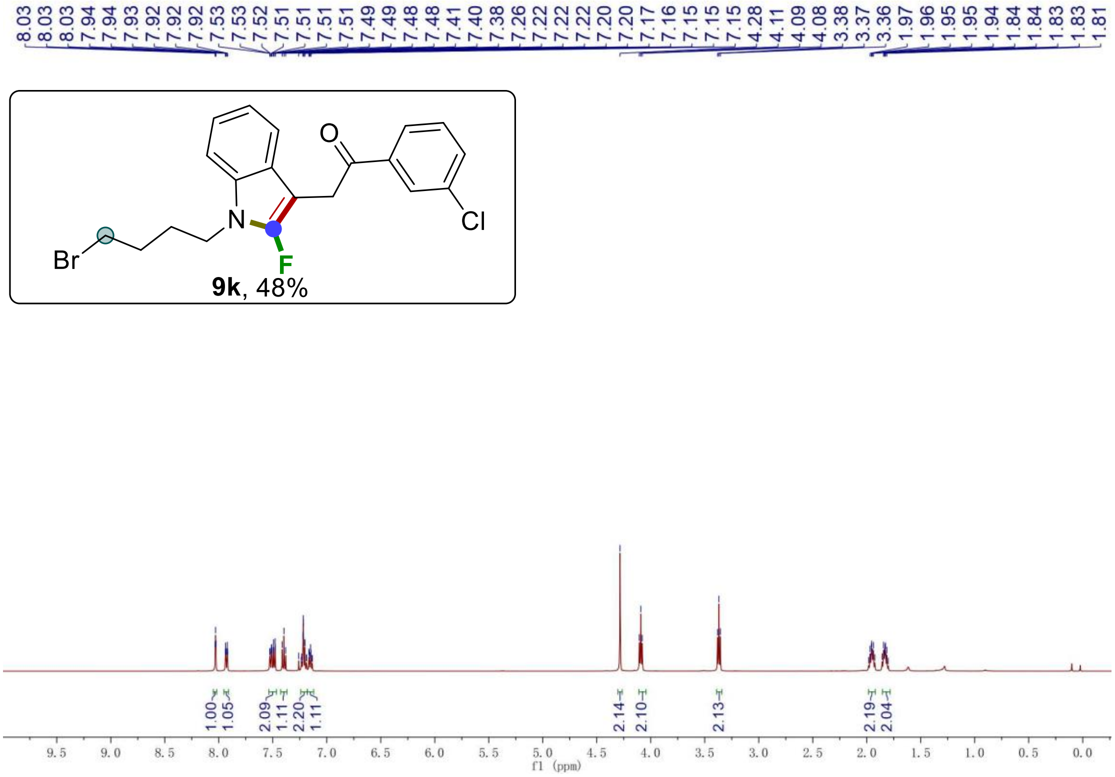


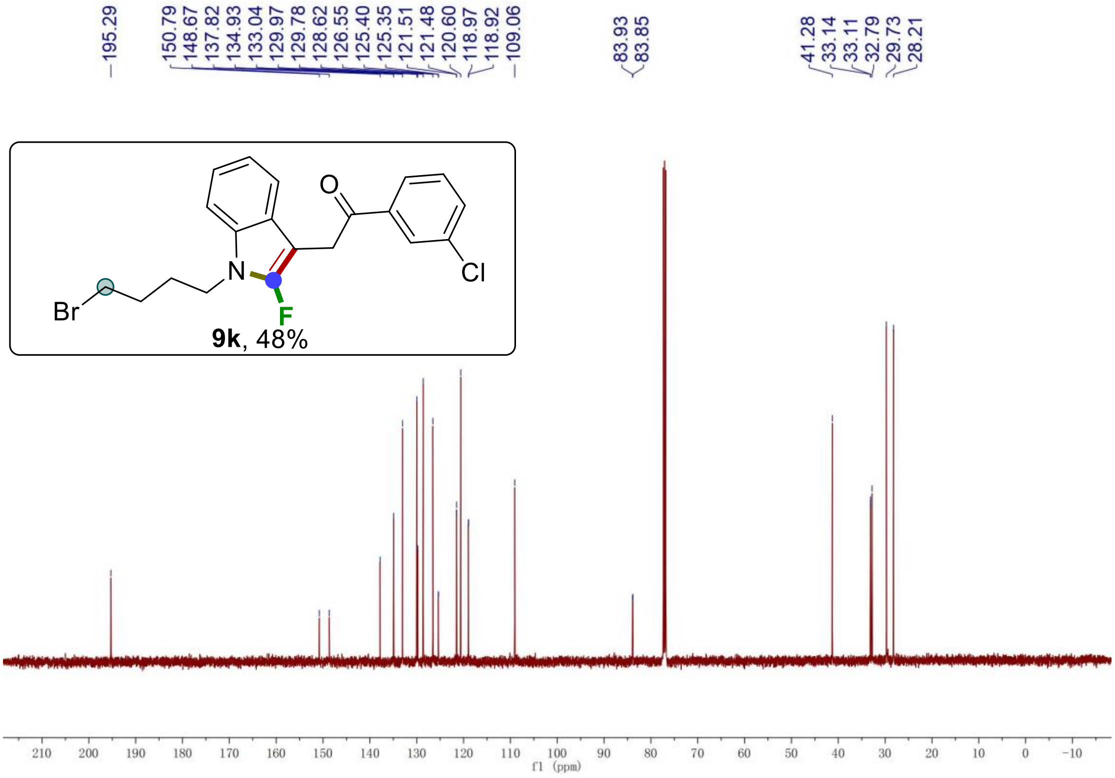

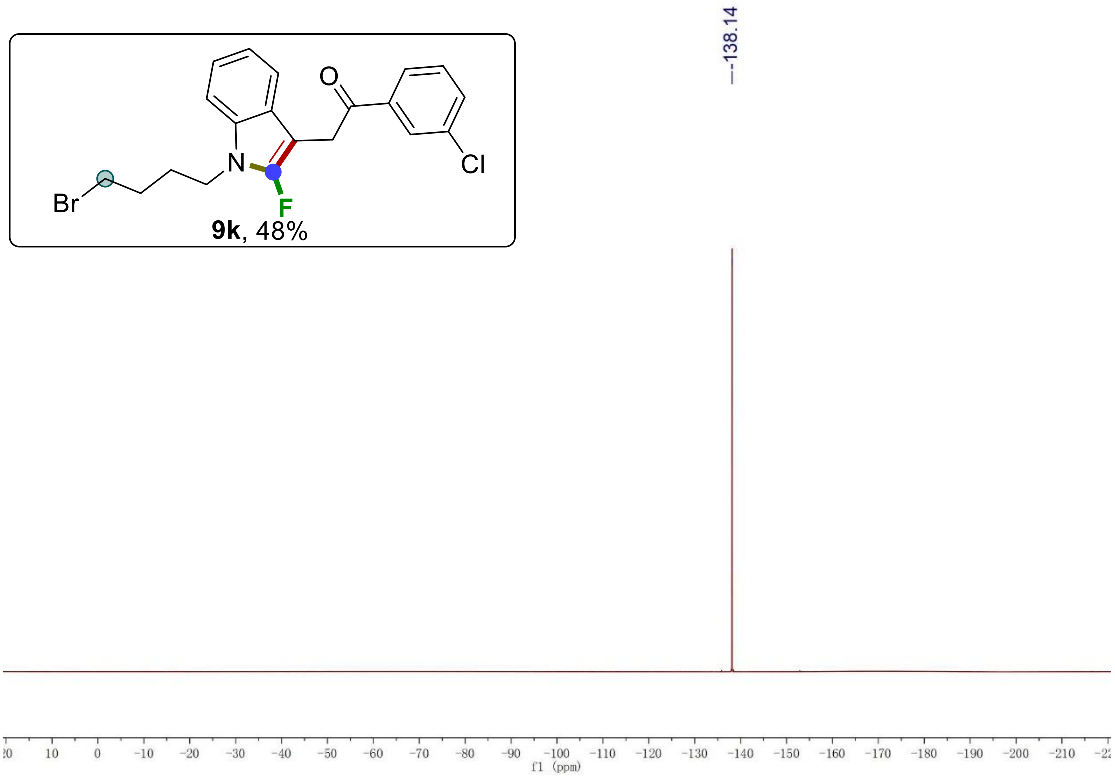


Supplementary Figure 151. $^{1}H$ NMR, $^{13}C$ NMR and $^{19}F$ NMR spectrum of **9k**.

# 1-(3-chlorophenyl)-2-(2-fluoro-1-(4-iodobutyl)-1H-indol-3-yl)ethan-1-one (9l)

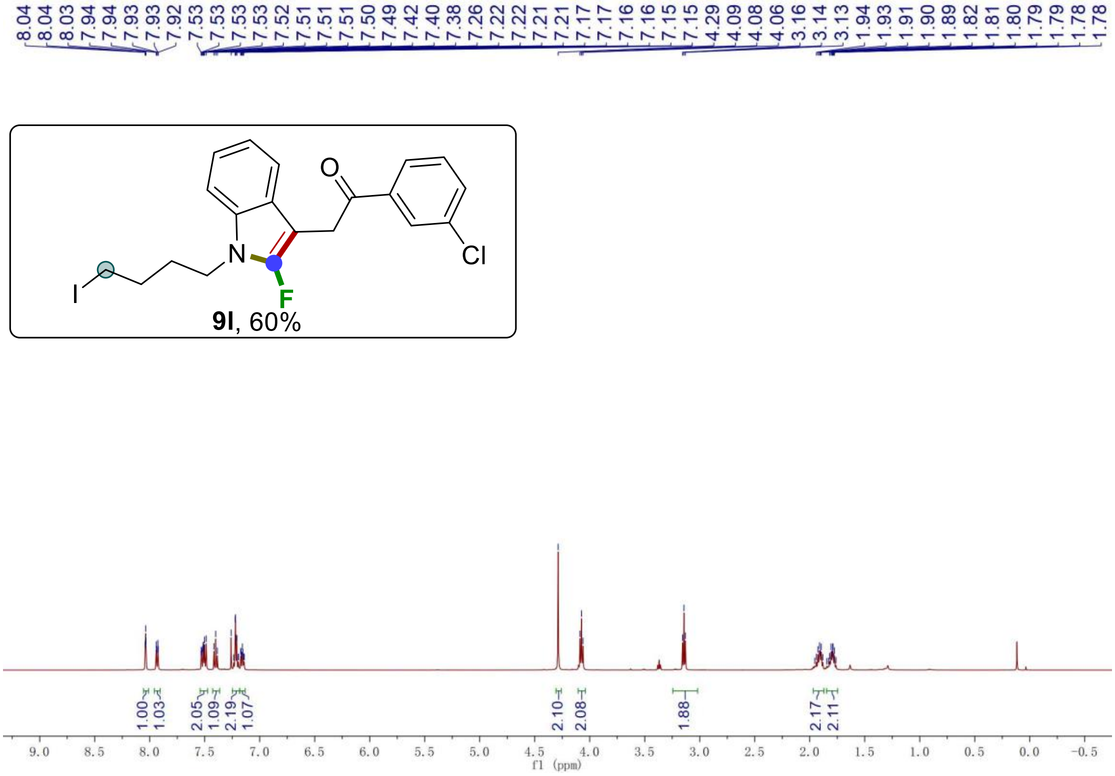


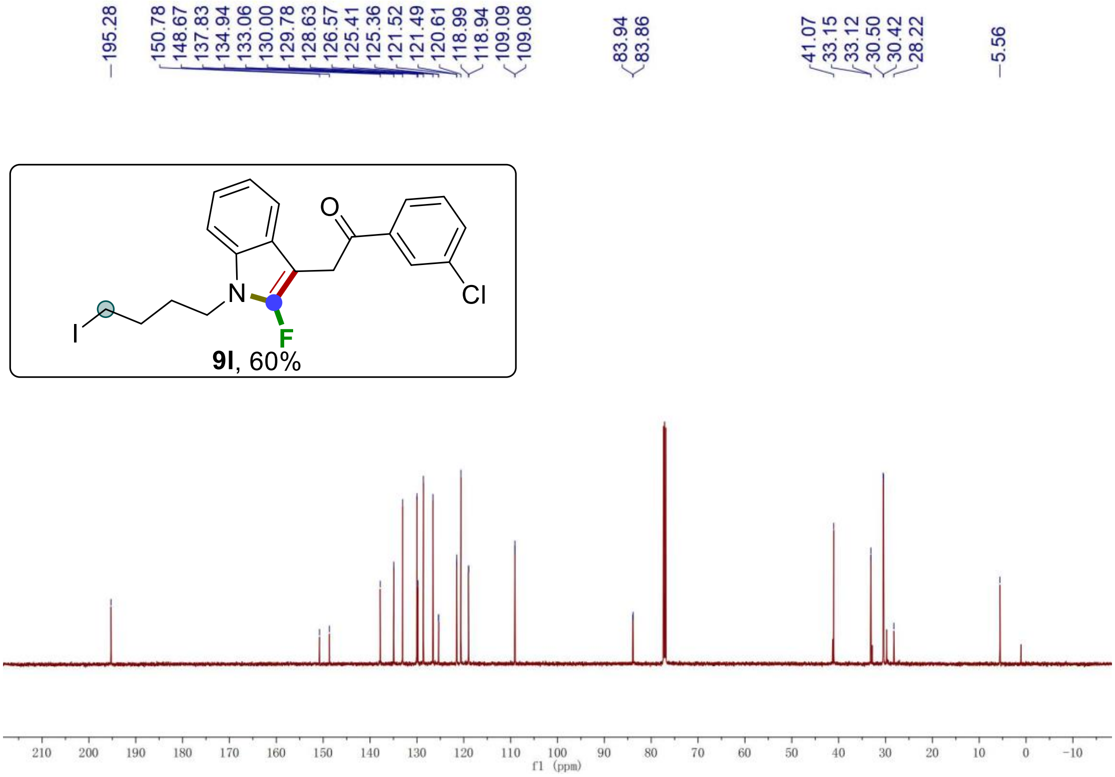

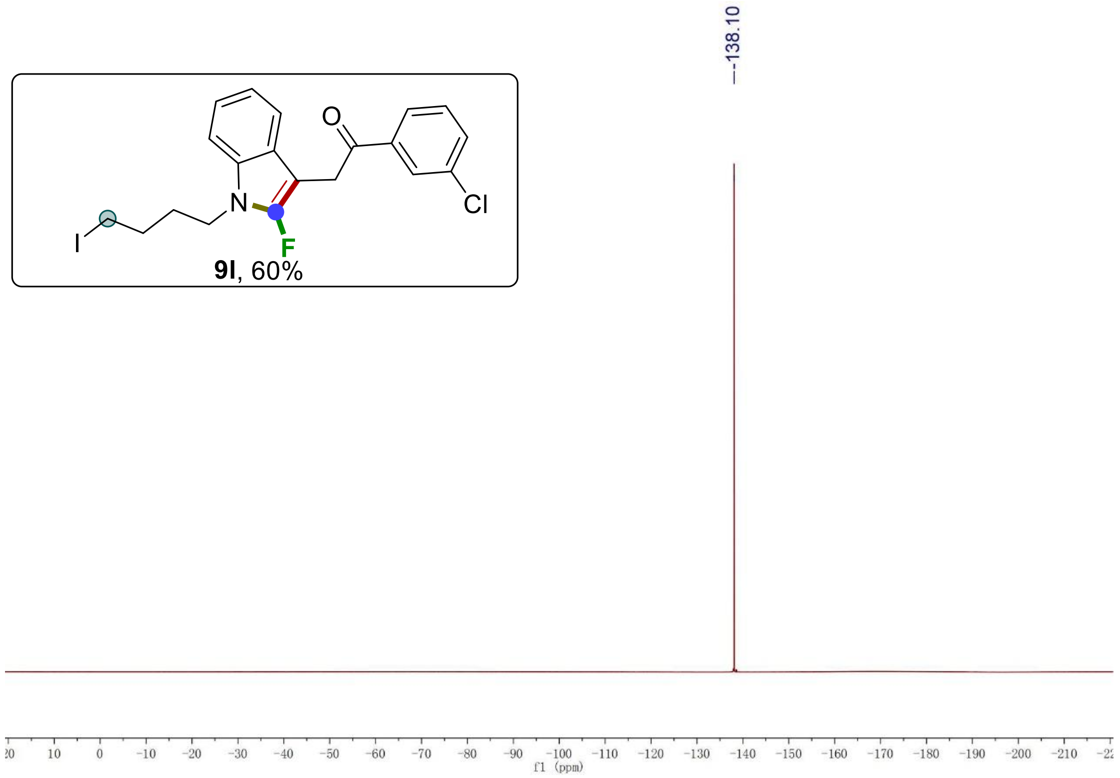


Supplementary Figure 152. $^{1}$H NMR, $^{13}$C NMR and $^{19}$F NMR spectrum of **9l**.

**2-(1-(4-bromobutyl)-2-fluoro-1H-indol-3-yl)-1-(3,4,5-trimethoxyphenyl)ethan-1-one (9m)**

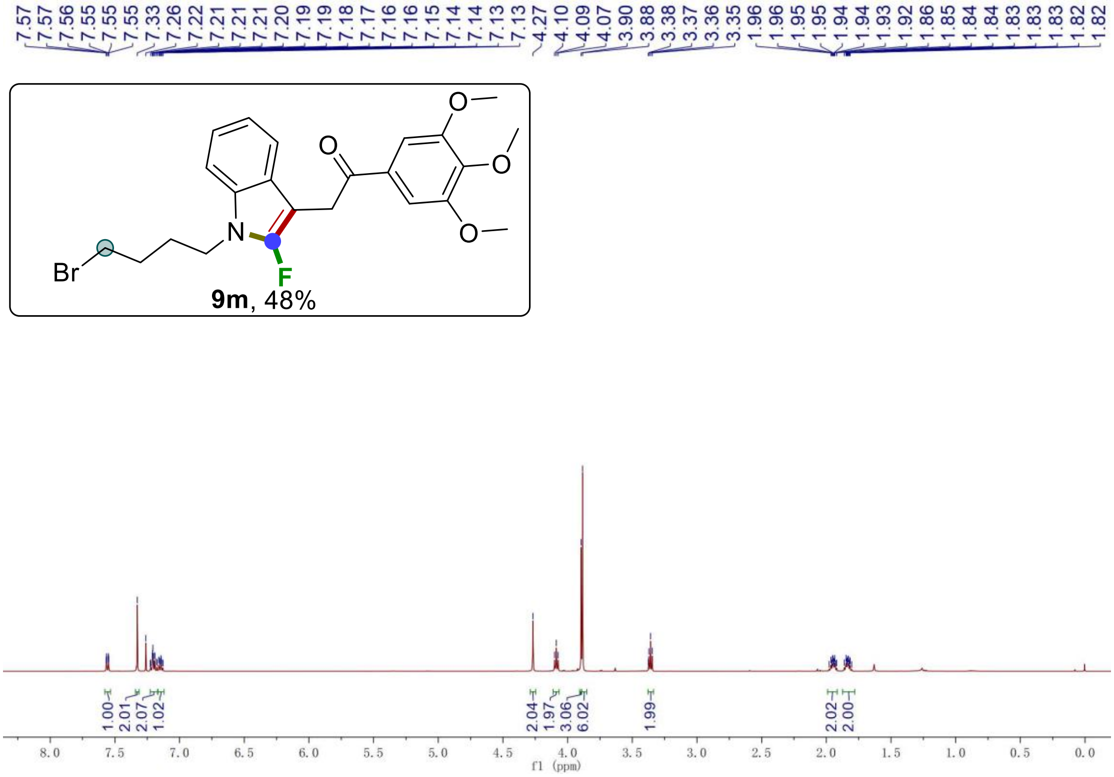


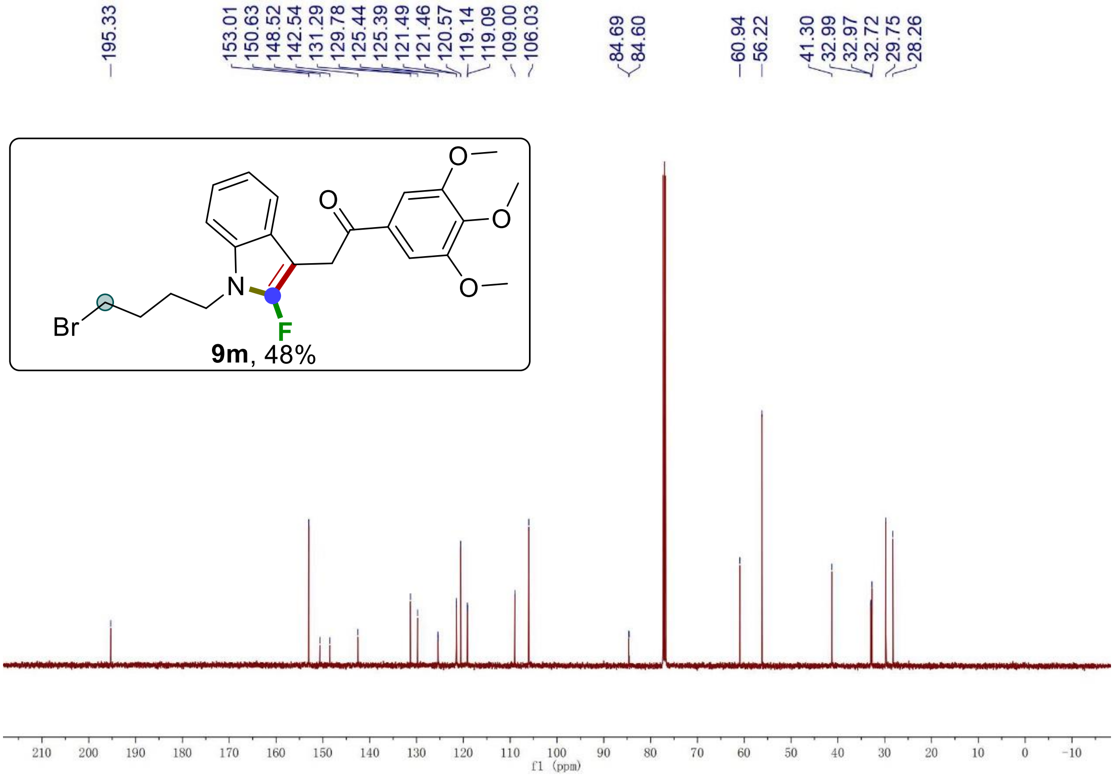

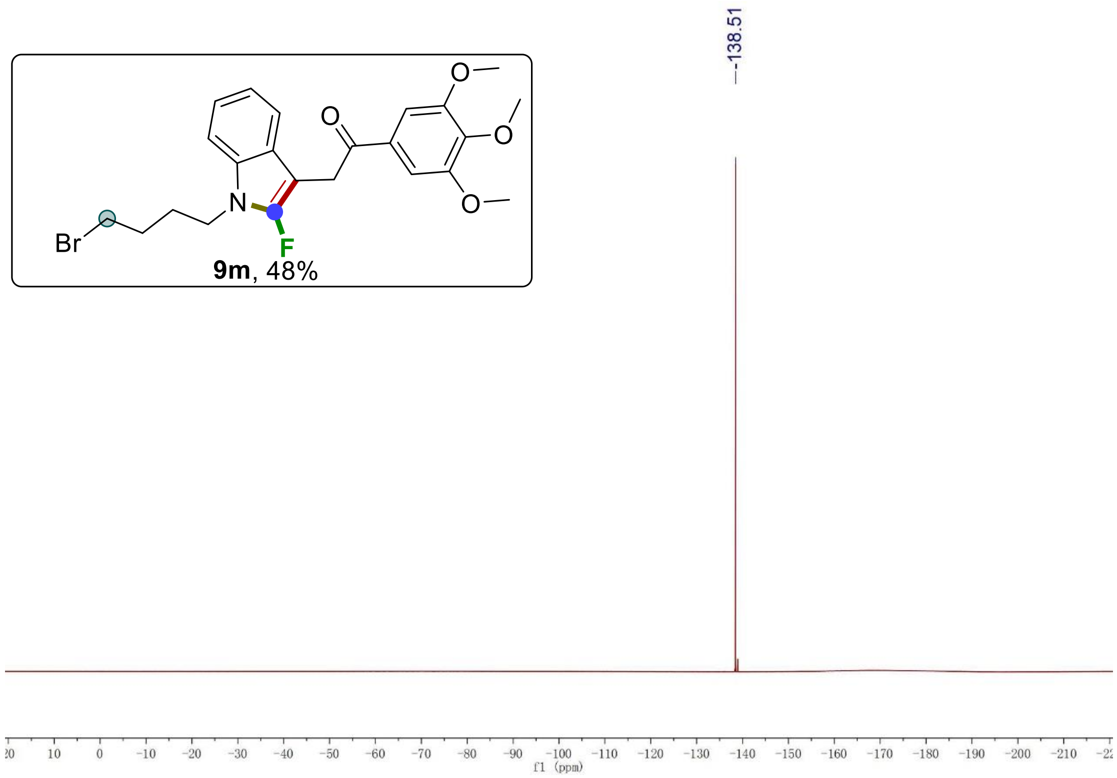


Supplementary Figure 153. $^{1}H$ NMR, $^{13}C$ NMR and $^{19}F$ NMR spectrum of **9m**.

## 2-(1-(4-bromobutyl)-2-fluoro-1H-indol-3-yl)-1-(naphthalen-2-yl)ethan-1-one (9n)

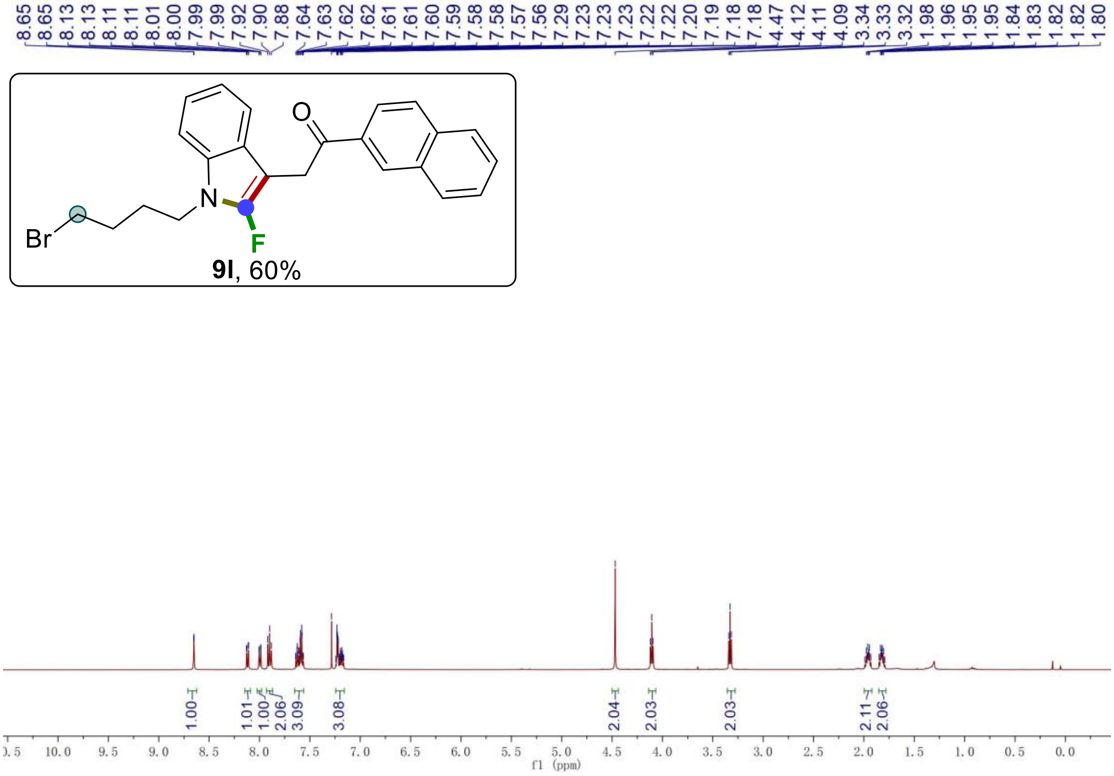


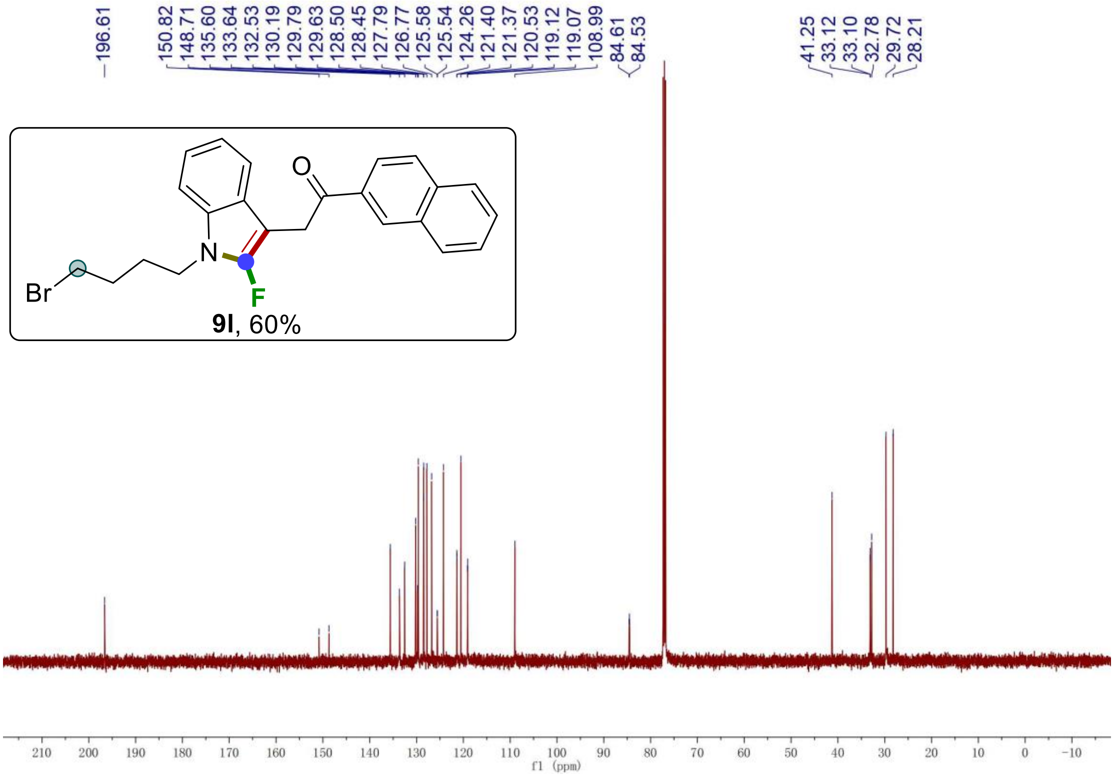

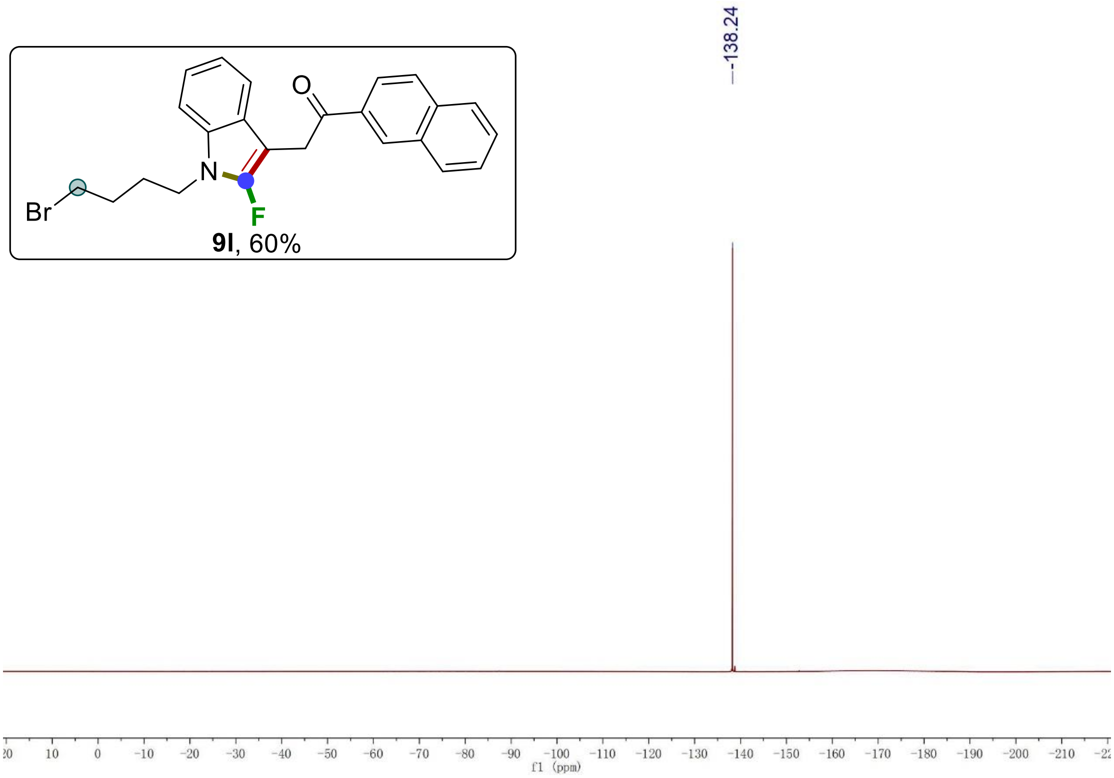


**9l**, 60%

Supplementary Figure 154. $^{1}$H NMR, $^{13}$C NMR and $^{19}$F NMR spectrum of **9n**.

**2-(1-(4-bromobutyl)-2-fluoro-1H-indol-3-yl)-1-(furan-2-yl)ethan-1-one (9o)**

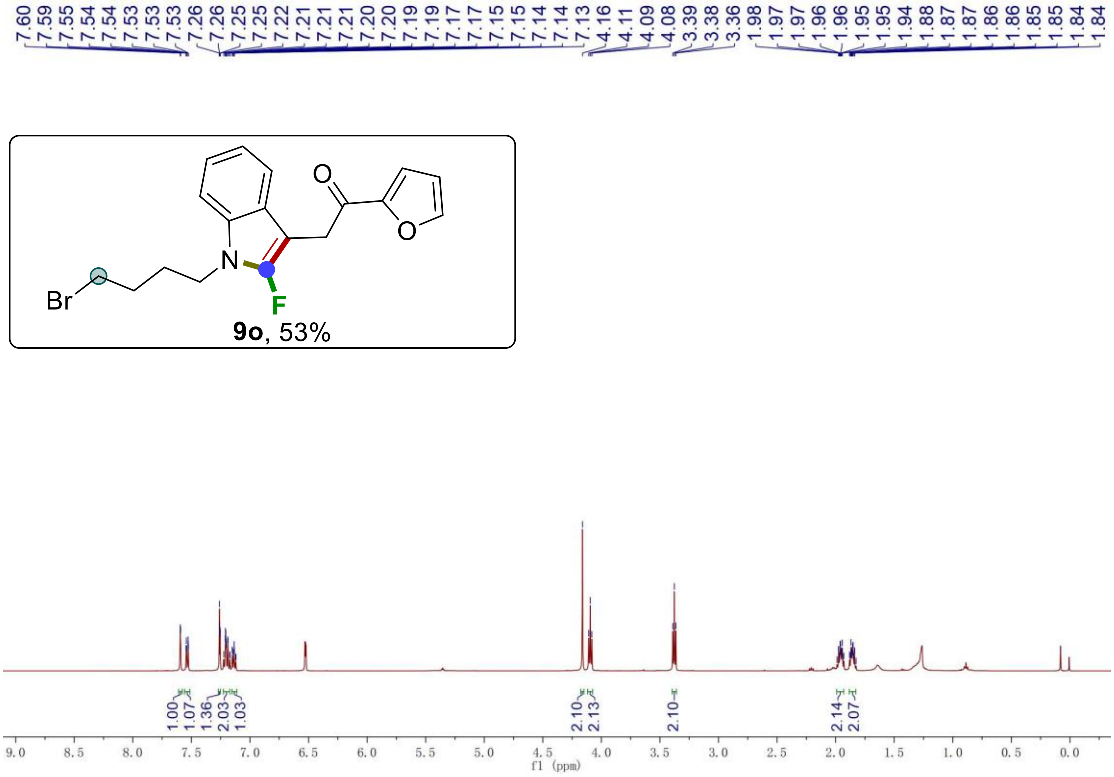


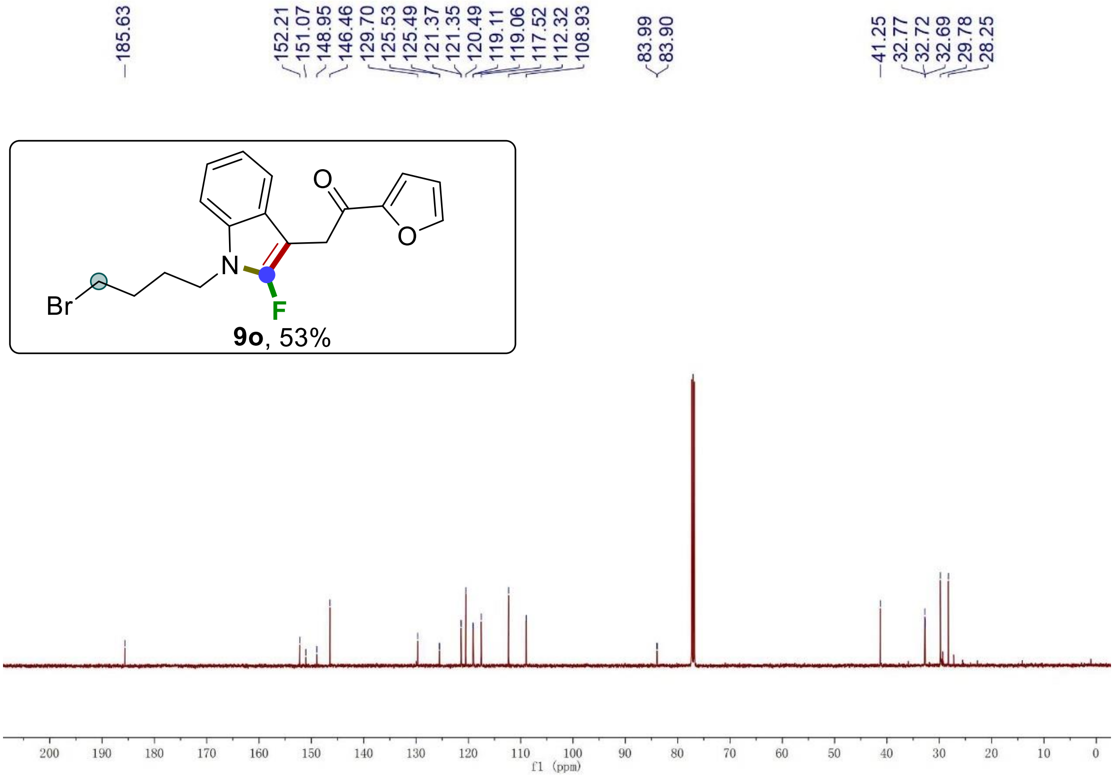

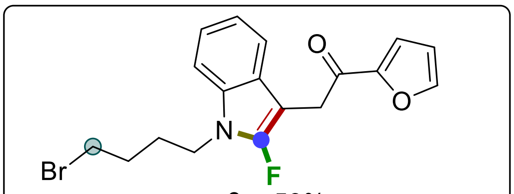


**9o**, 53%

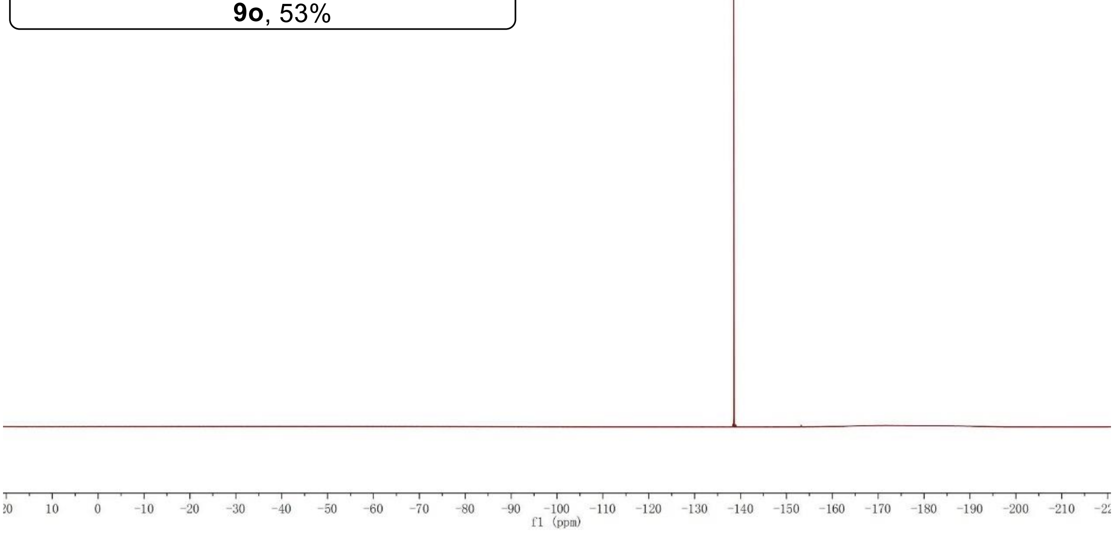


Supplementary Figure 155. $^{1}H$ NMR, $^{13}C$ NMR and $^{19}F$ NMR spectrum of **9o**.

## 2-(2-fluoro-1-(4-iodobutyl)-1H-indol-3-yl)-1-(furan-2-yl)ethan-1-one (9p)

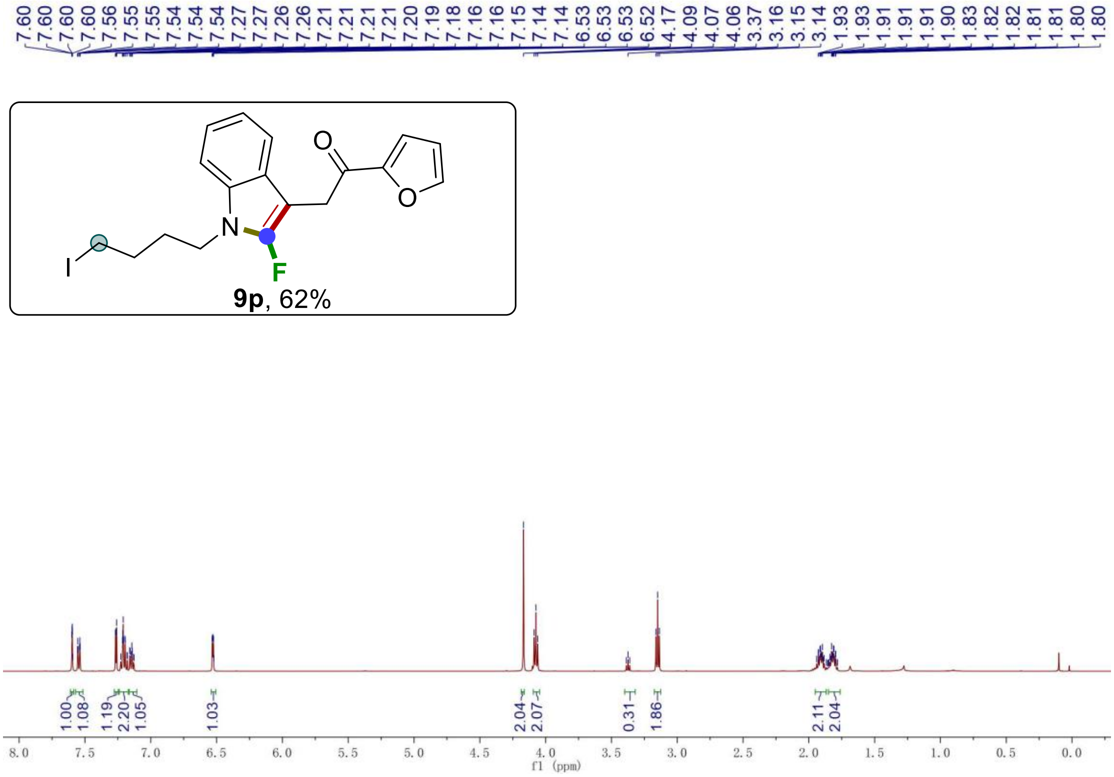

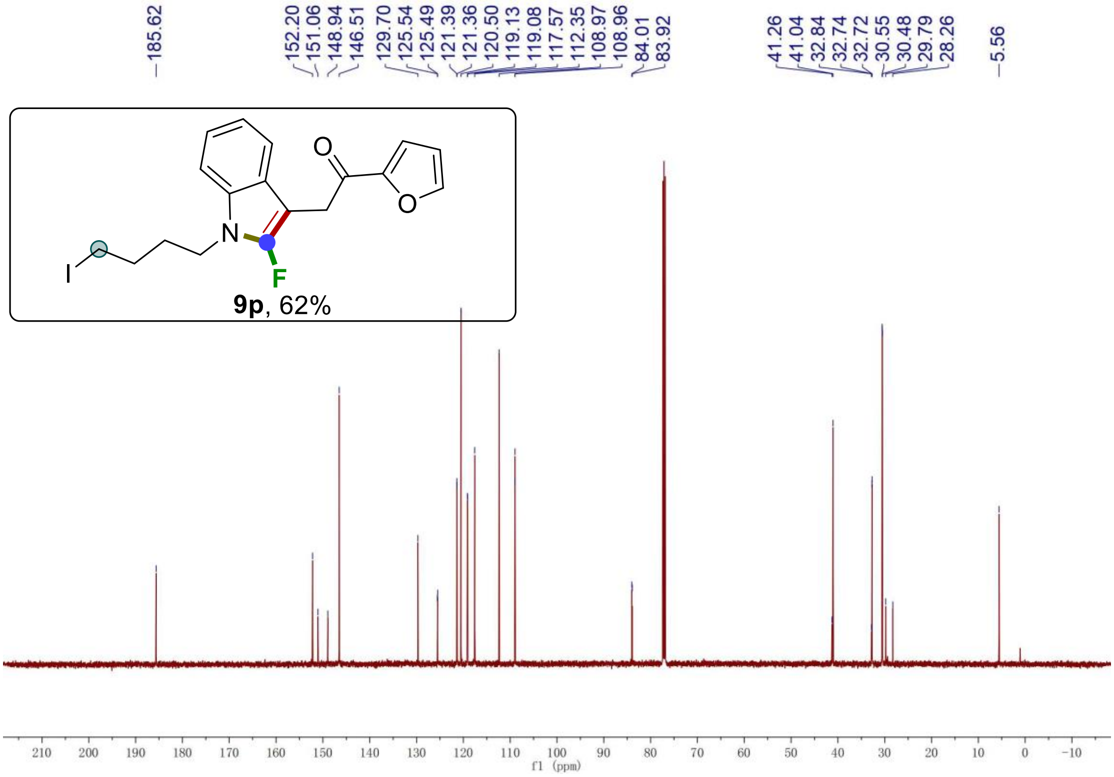

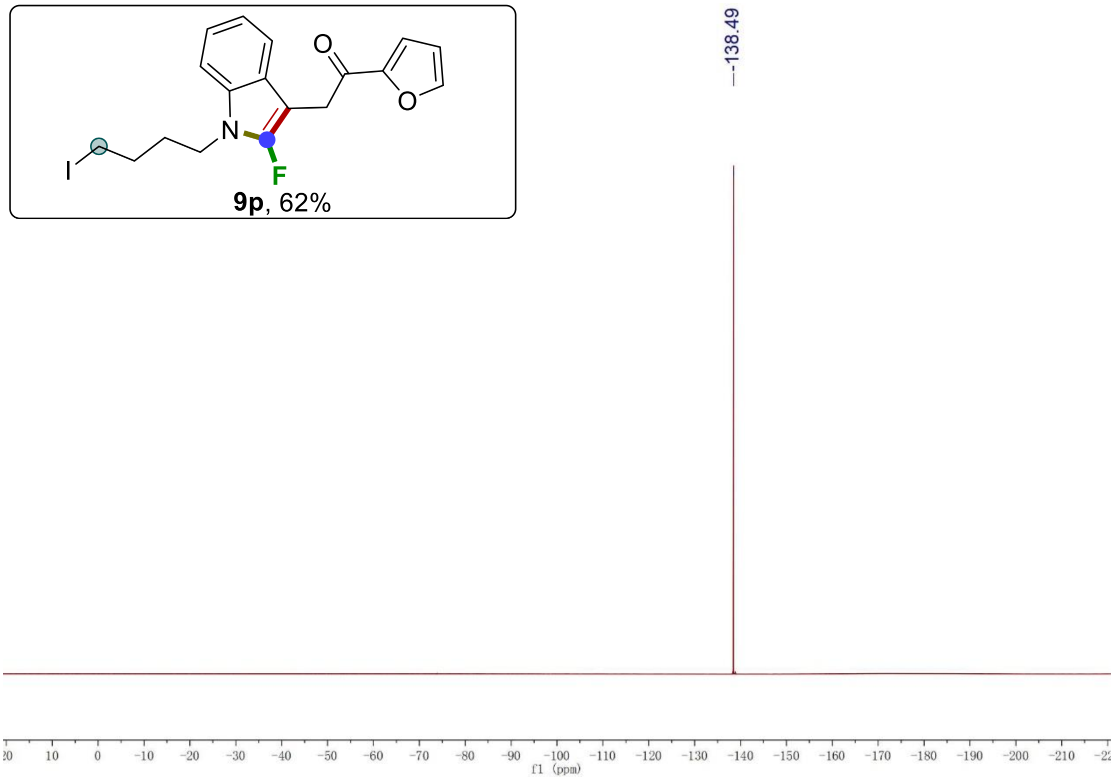


Supplementary Figure 156. $^{1}H$ NMR, $^{13}C$ NMR and $^{19}F$ NMR spectrum of **9p**.

**2-(1-(4-bromobutyl)-2-fluoro-1H-indol-3-yl)-1-(thiophen-2-yl)ethan-1-one (9q)**

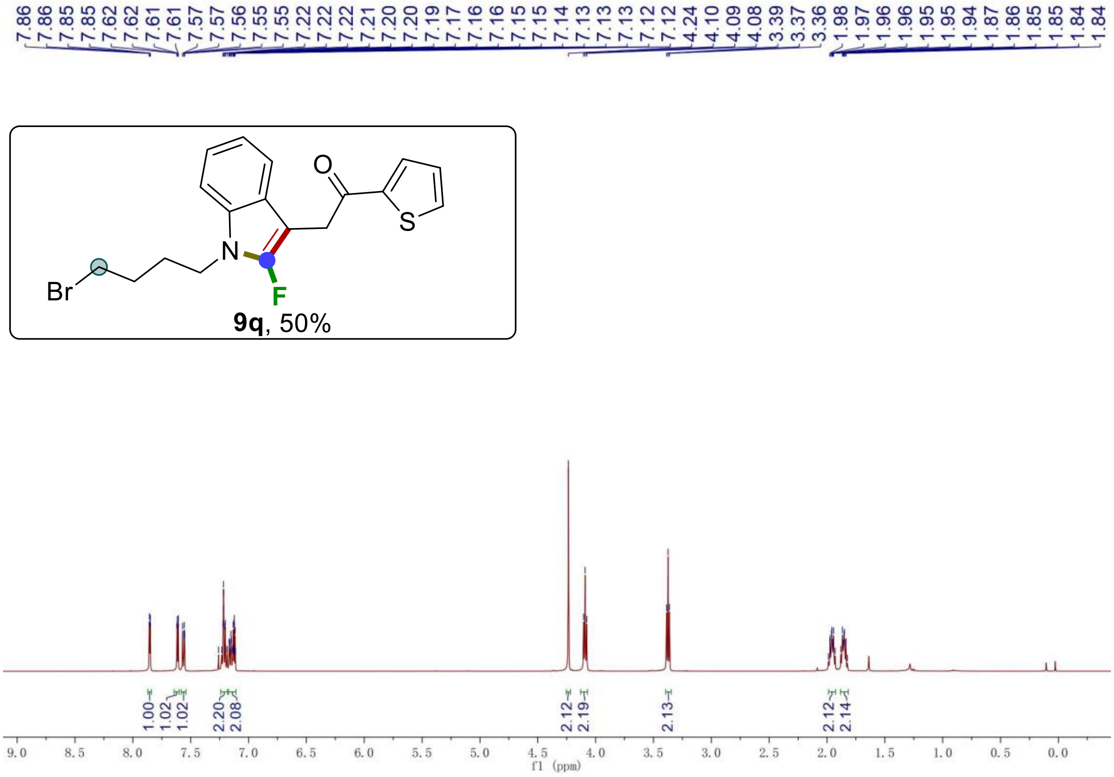


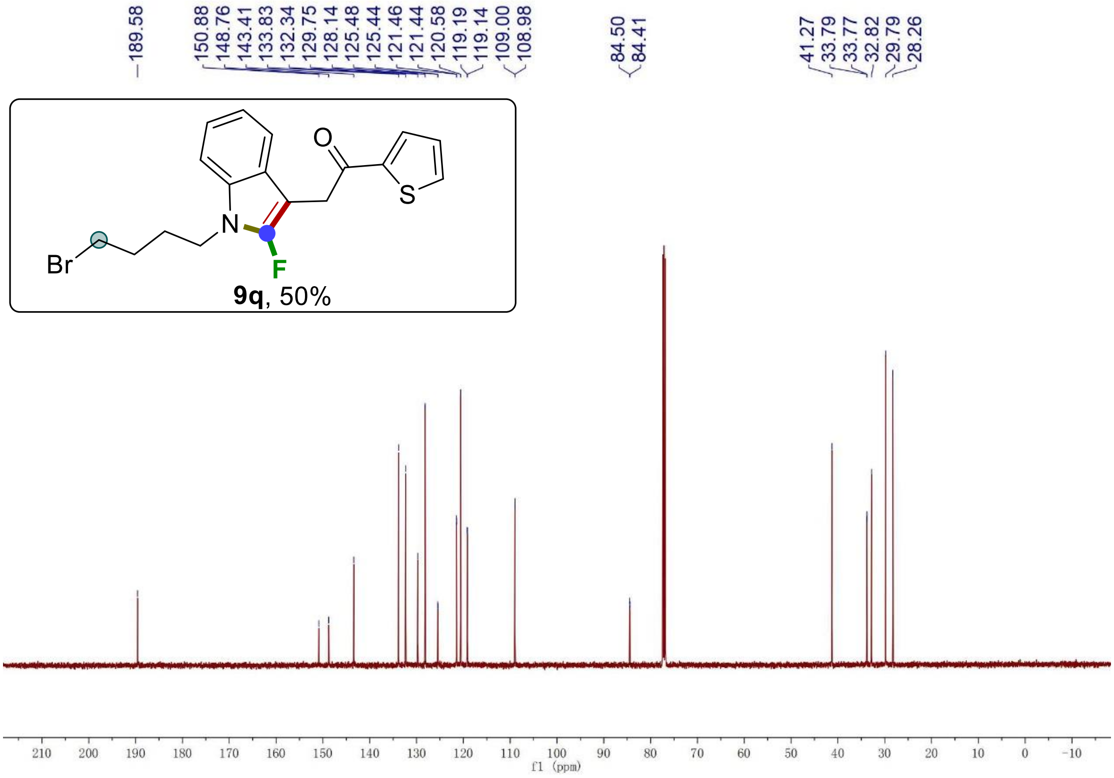

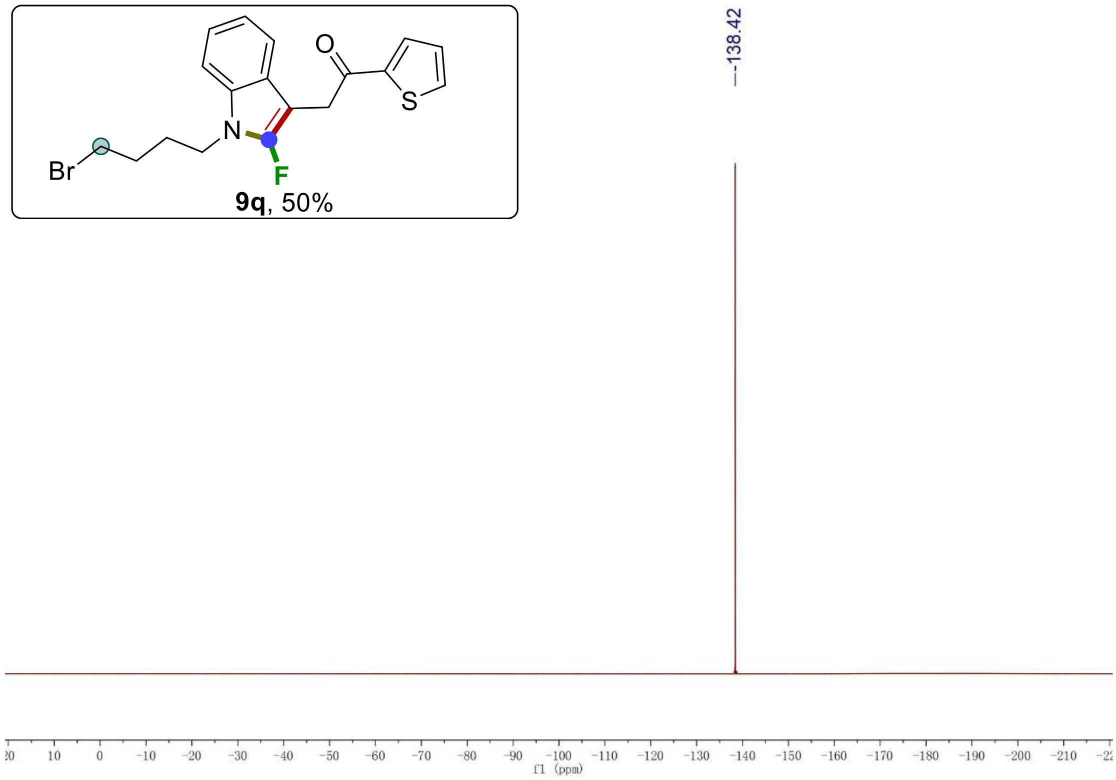


Supplementary Figure 157. $^{1}H$ NMR, $^{13}C$ NMR and $^{19}F$ NMR spectrum of **9q**.

2-(1-(4-bromobutyl)-2-fluoro-5-methyl-1H-indol-3-yl)-1-phenylethan-1-one **(9r)**

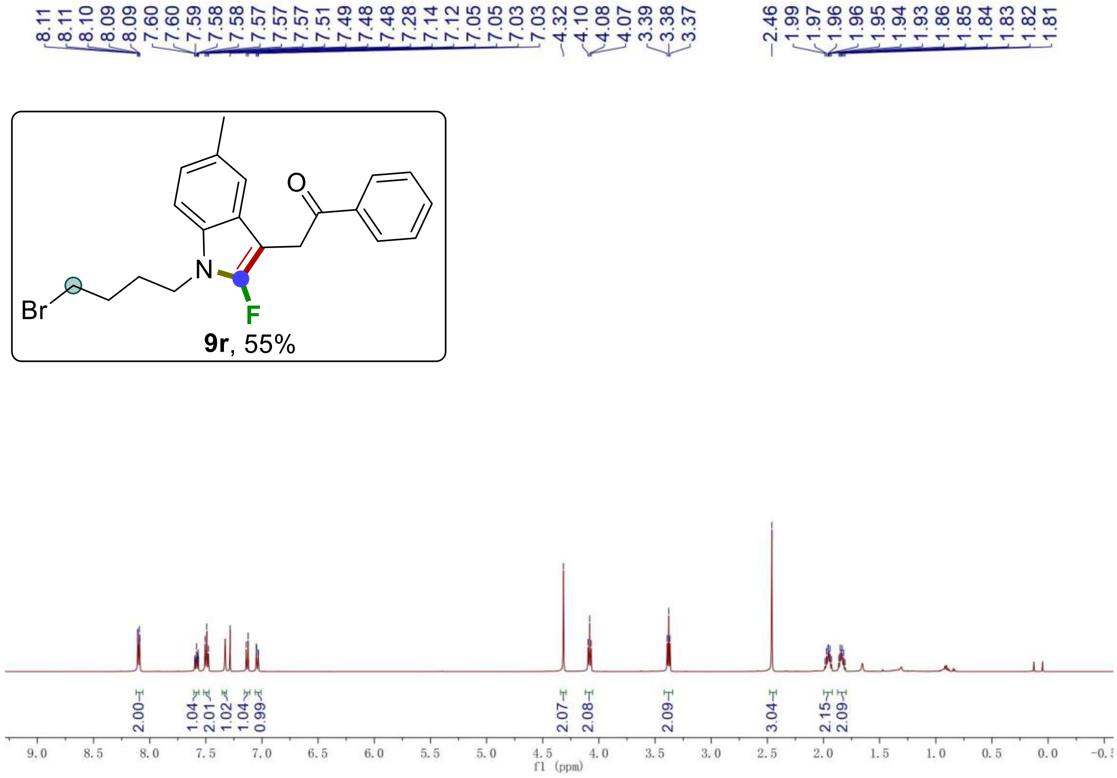

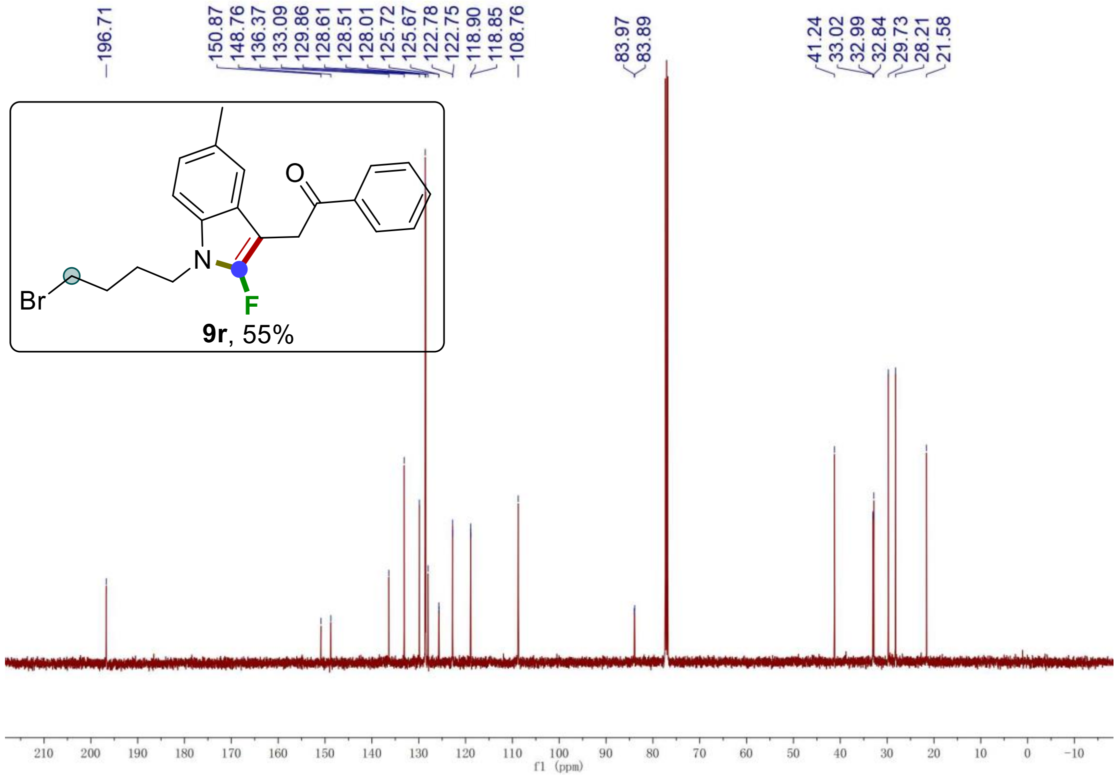

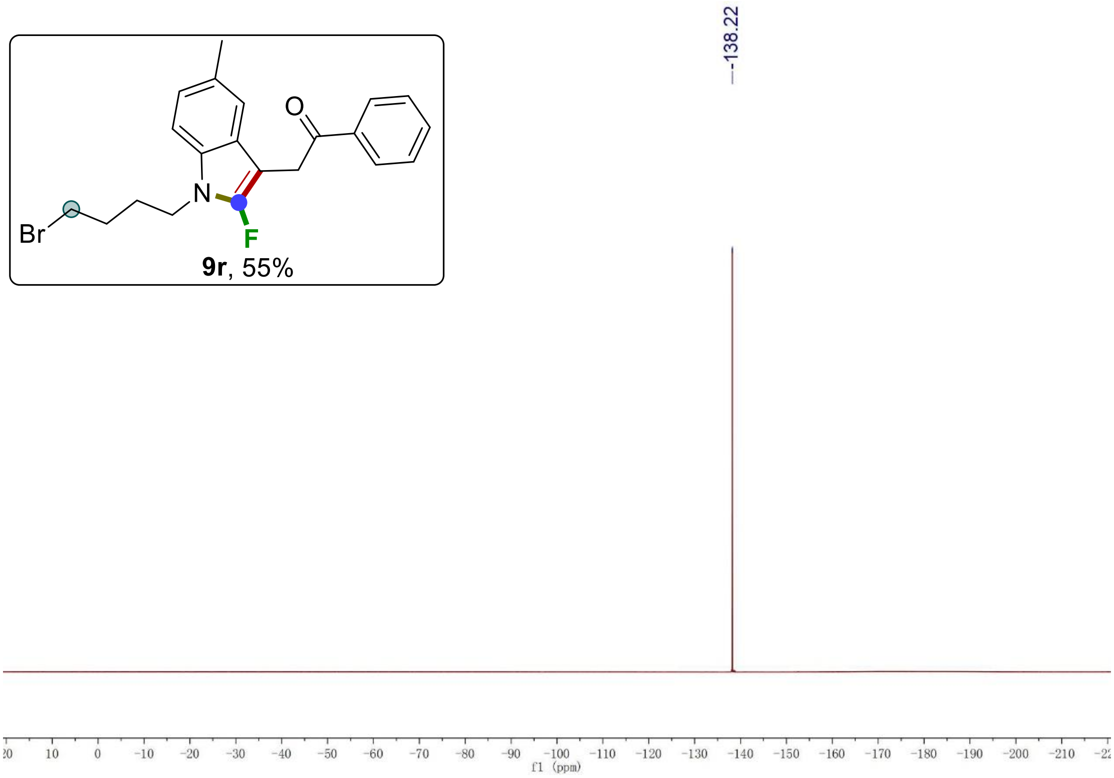


Supplementary Figure 158. $^{1}H$ NMR, $^{13}C$ NMR and $^{19}F$ NMR spectrum of **9r**.

# 2-(1-(4-bromobutyl)-2-fluoro-6-methyl-1H-indol-3-yl)-1-phenylethan-1-one (9s)

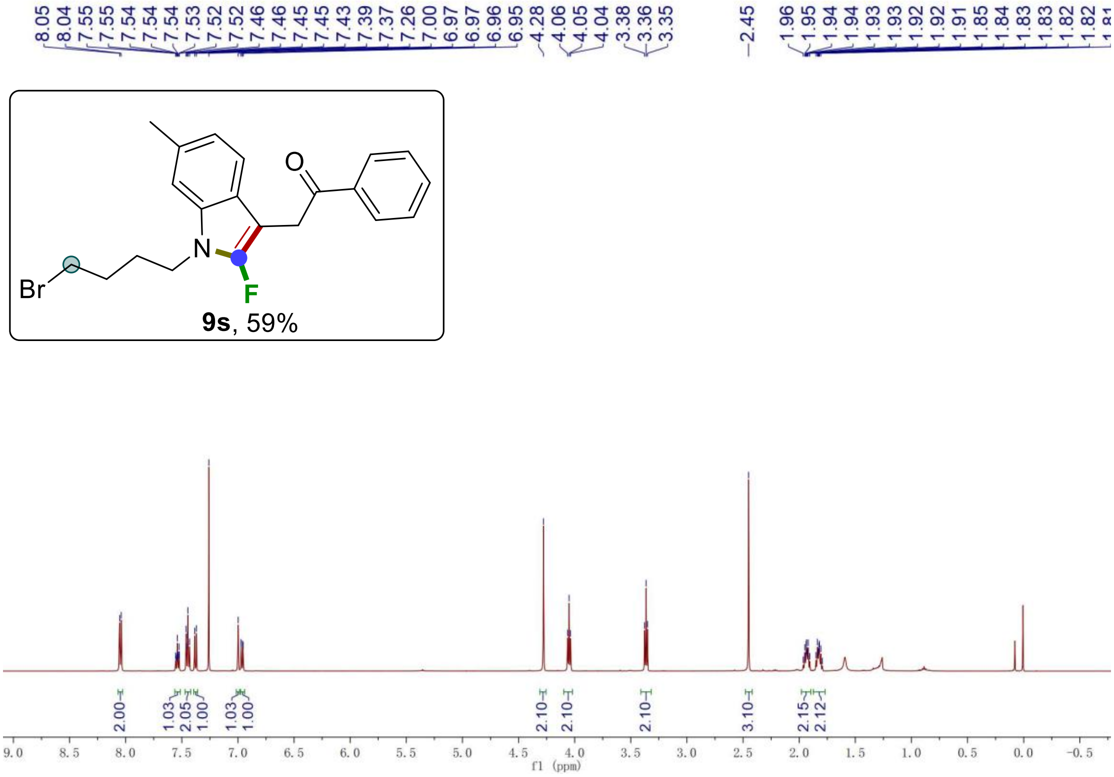


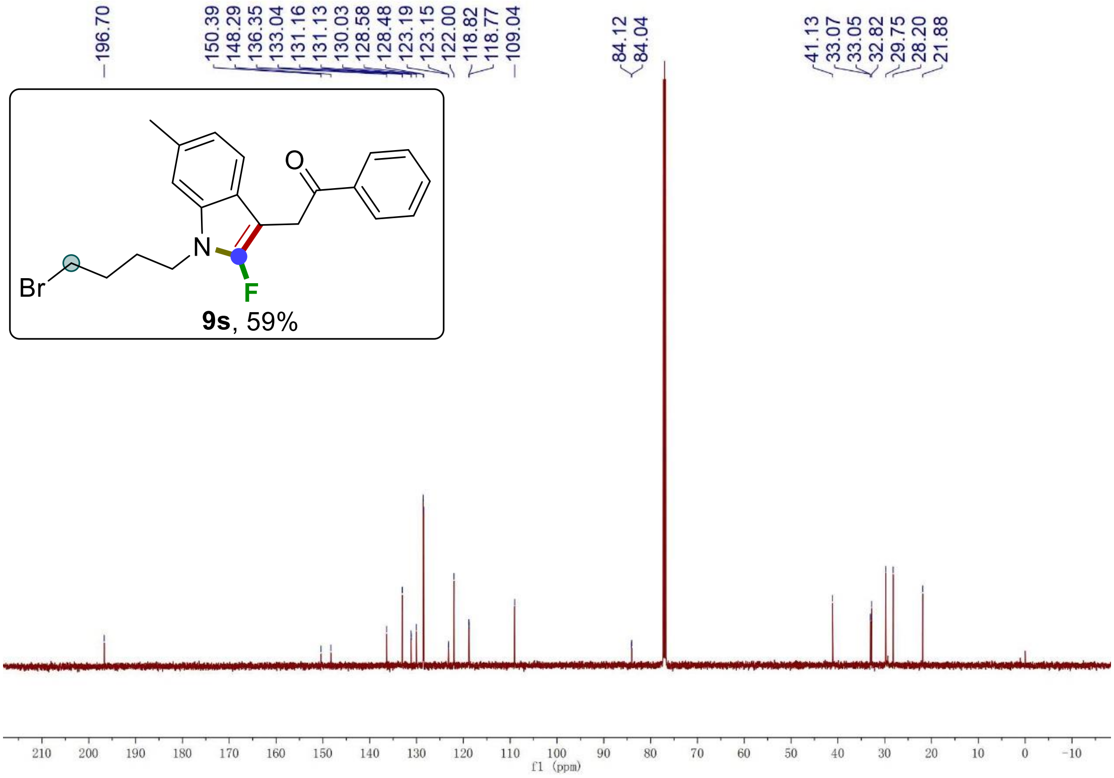

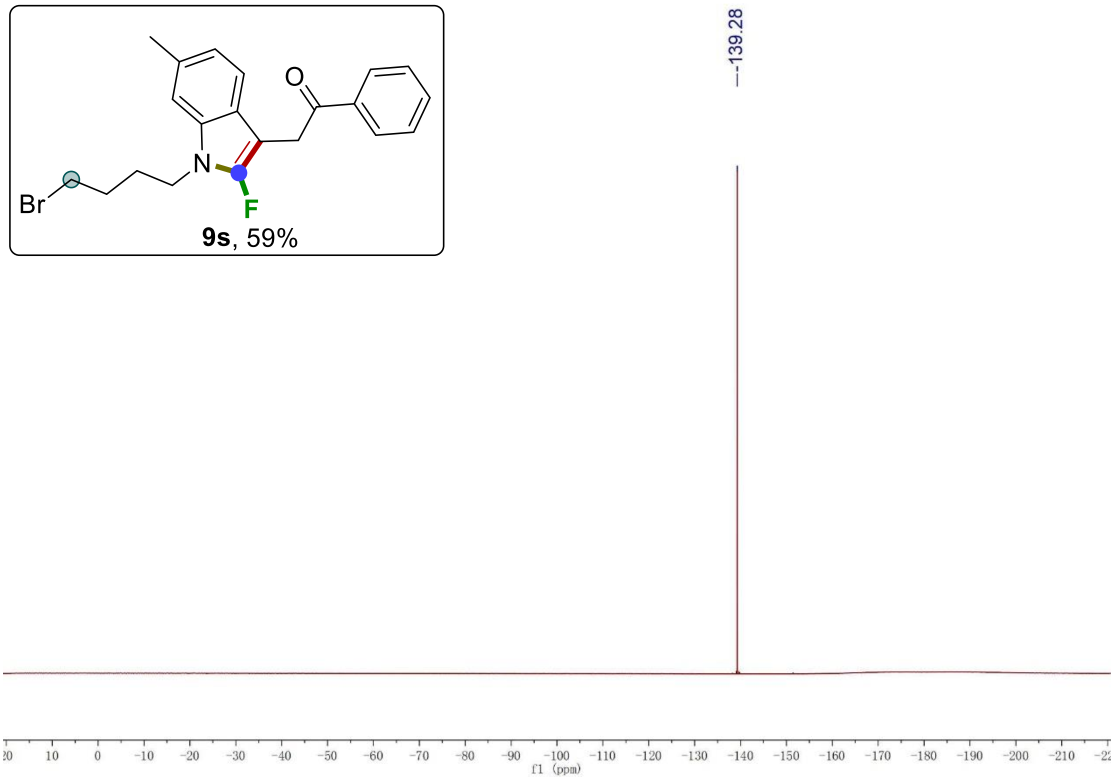


Supplementary Figure 159. $^{1}H$ NMR, $^{13}C$ NMR and $^{19}F$ NMR spectrum of **9s**.

## 1-(1-(4-bromobutyl)-2-fluoro-6-methyl-1H-indol-3-yl)-4-phenylbutan-2-one (9t)

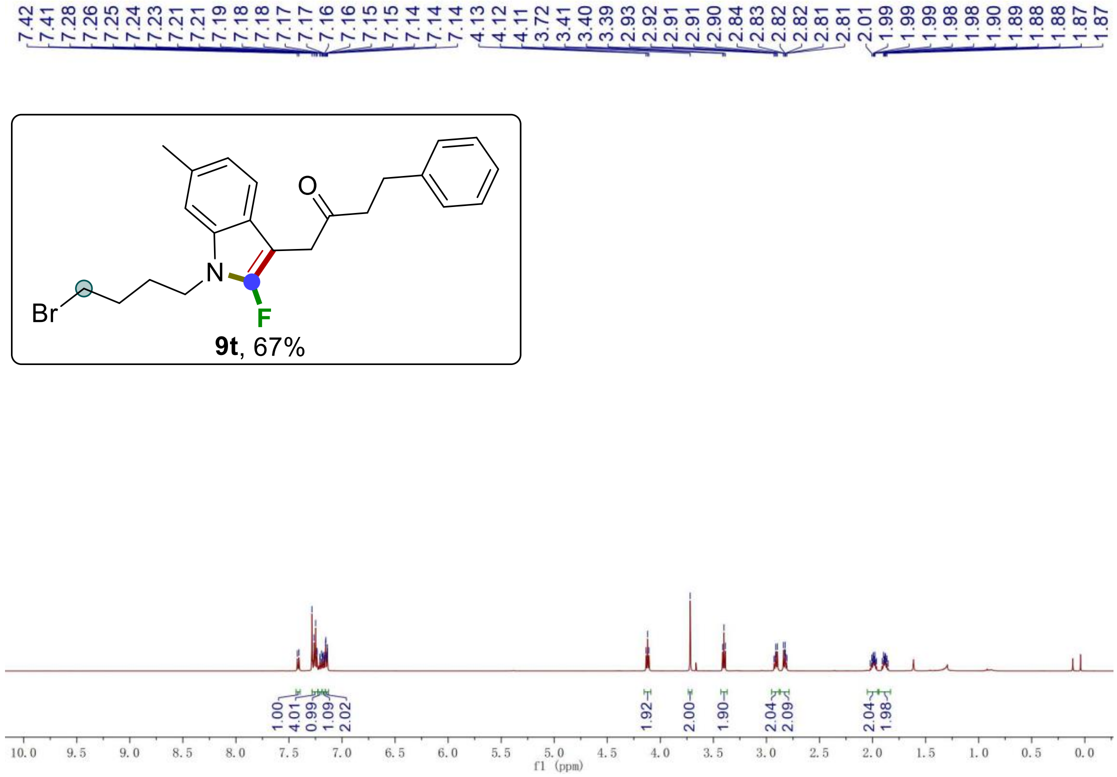


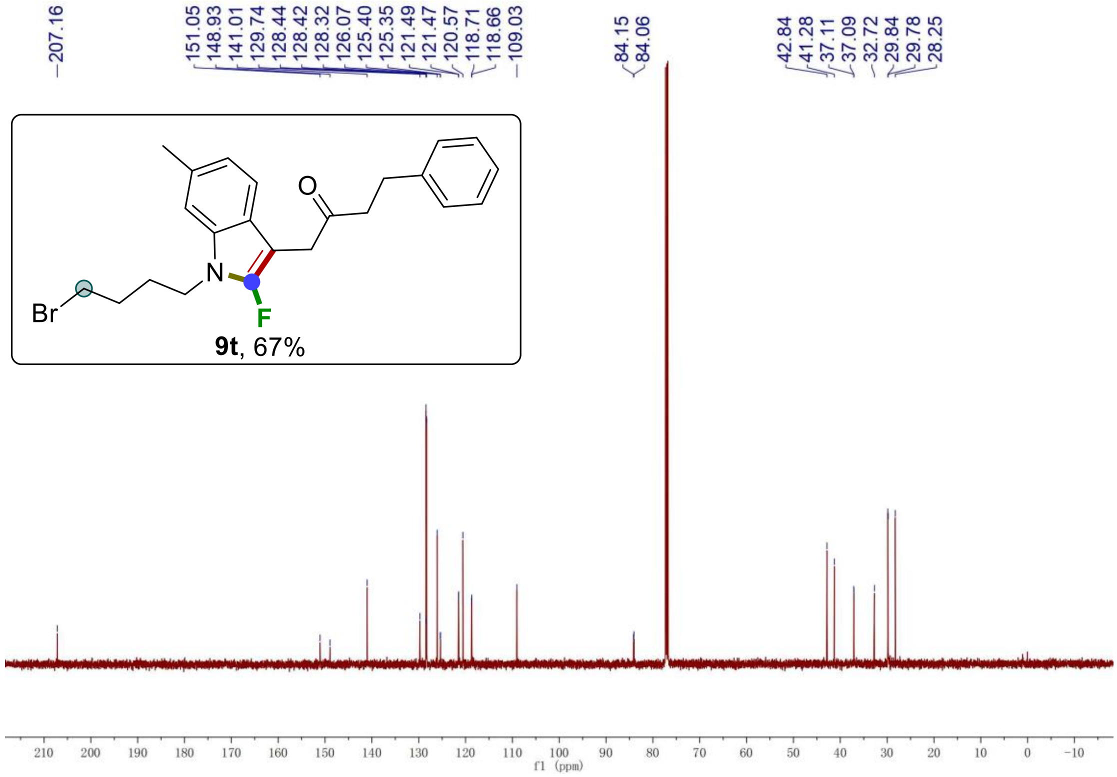

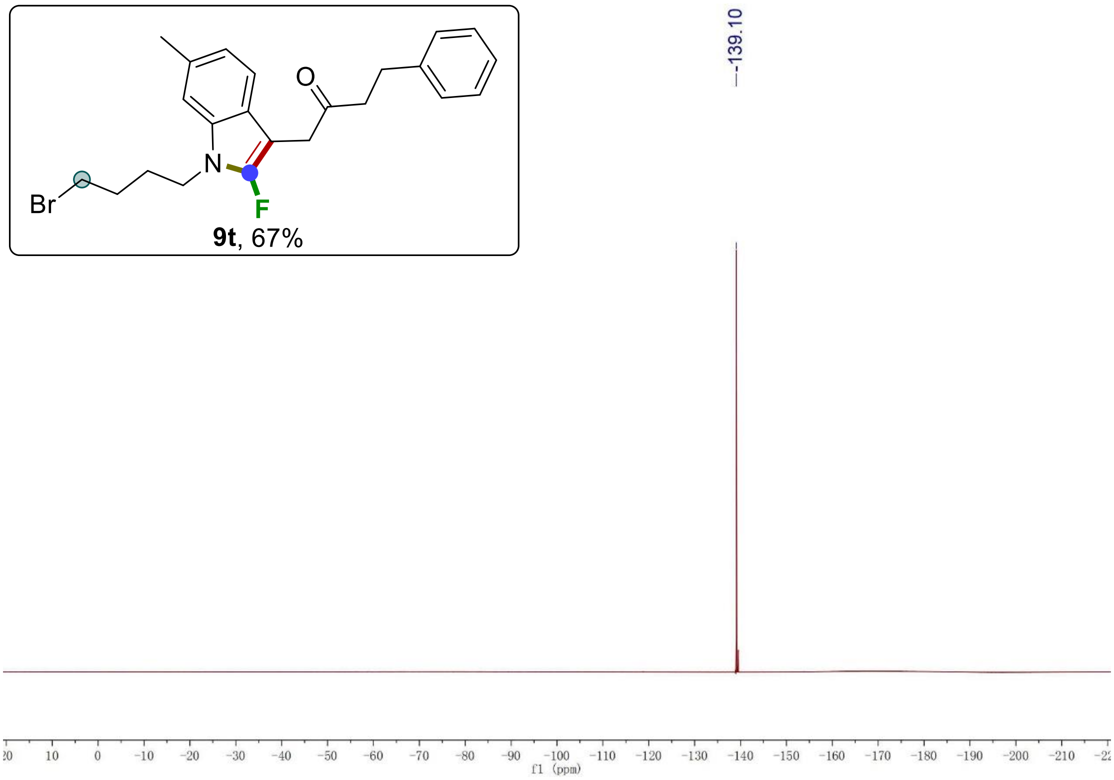


Supplementary Figure 160. $^{1}$H NMR, $^{13}$C NMR and $^{19}$F NMR spectrum of **9t**.

## 1-(1-(4-bromobutyl)-2-fluoro-1H-indol-3-yl)nonan-2-one (9u)

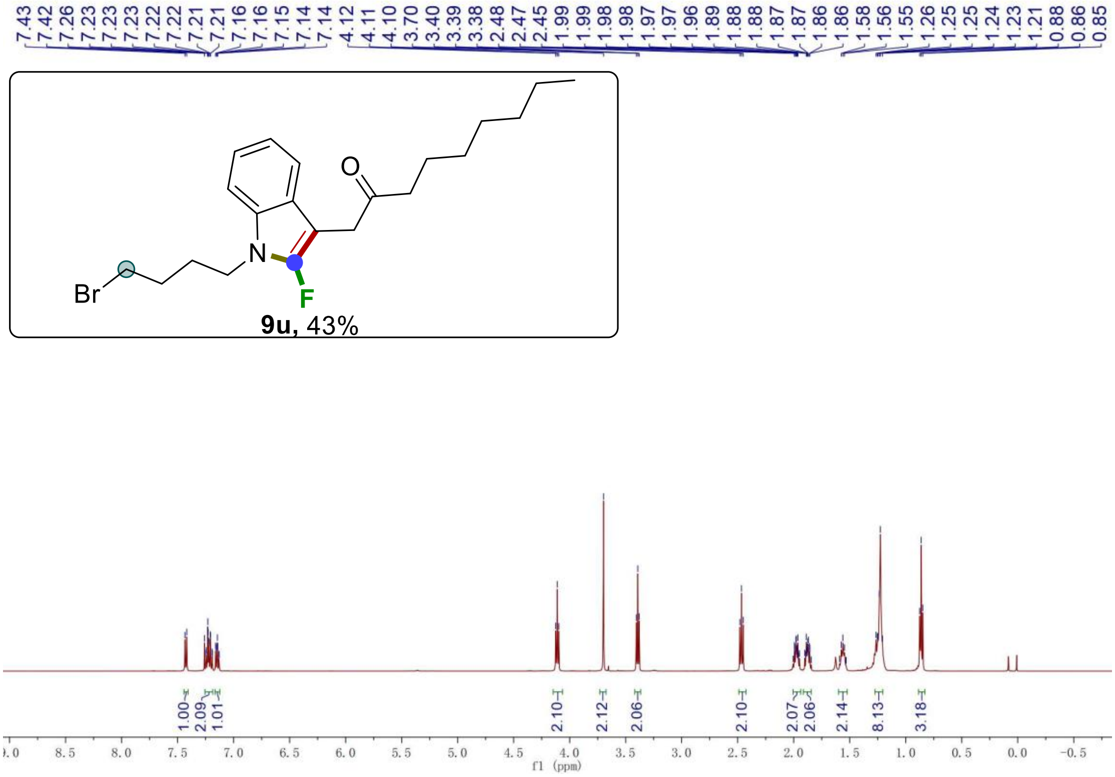


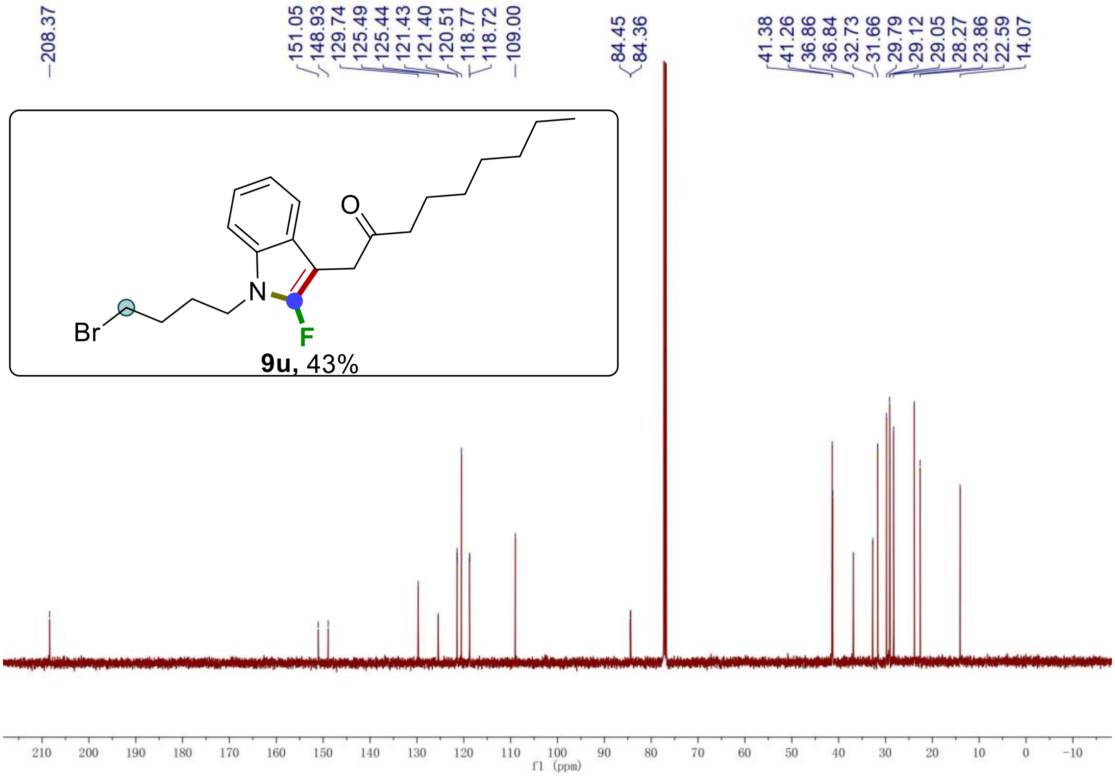

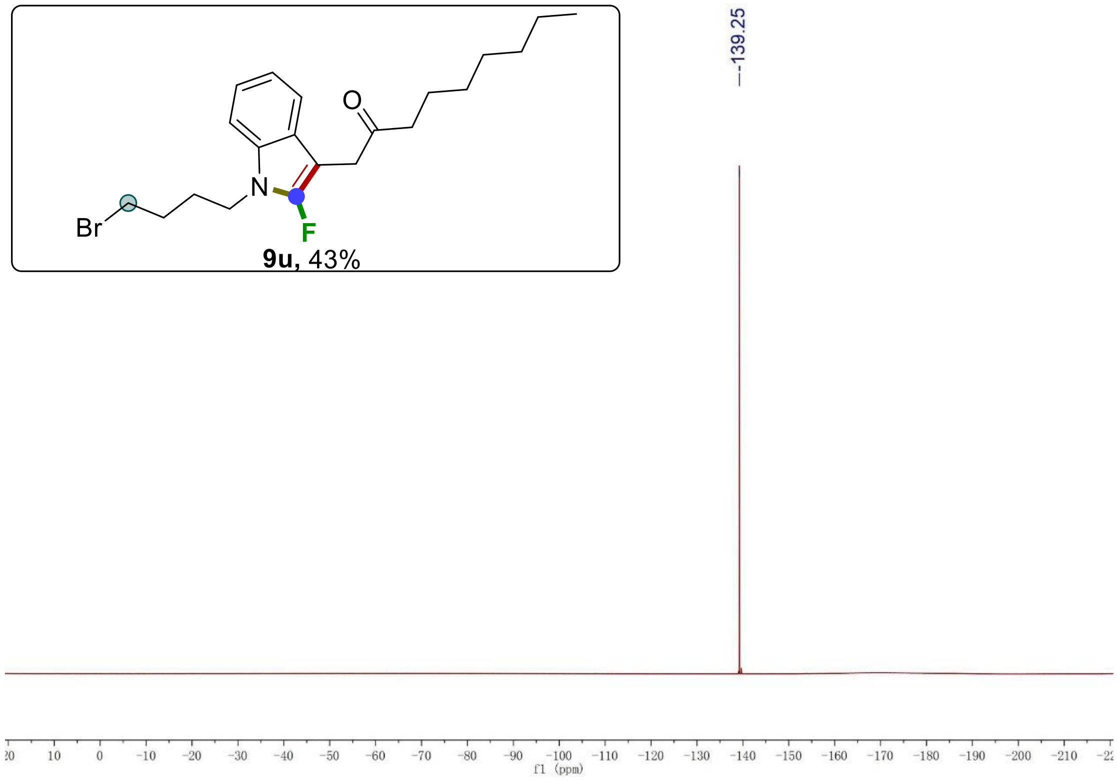


Supplementary Figure 161. $^{1}$H NMR, $^{13}$C NMR and $^{19}$F NMR spectrum of **9u**.

## 2-(-2-fluoro-1-methyl-1H-indol-3-yl)-1- ferroceneyl -1-one (11a)

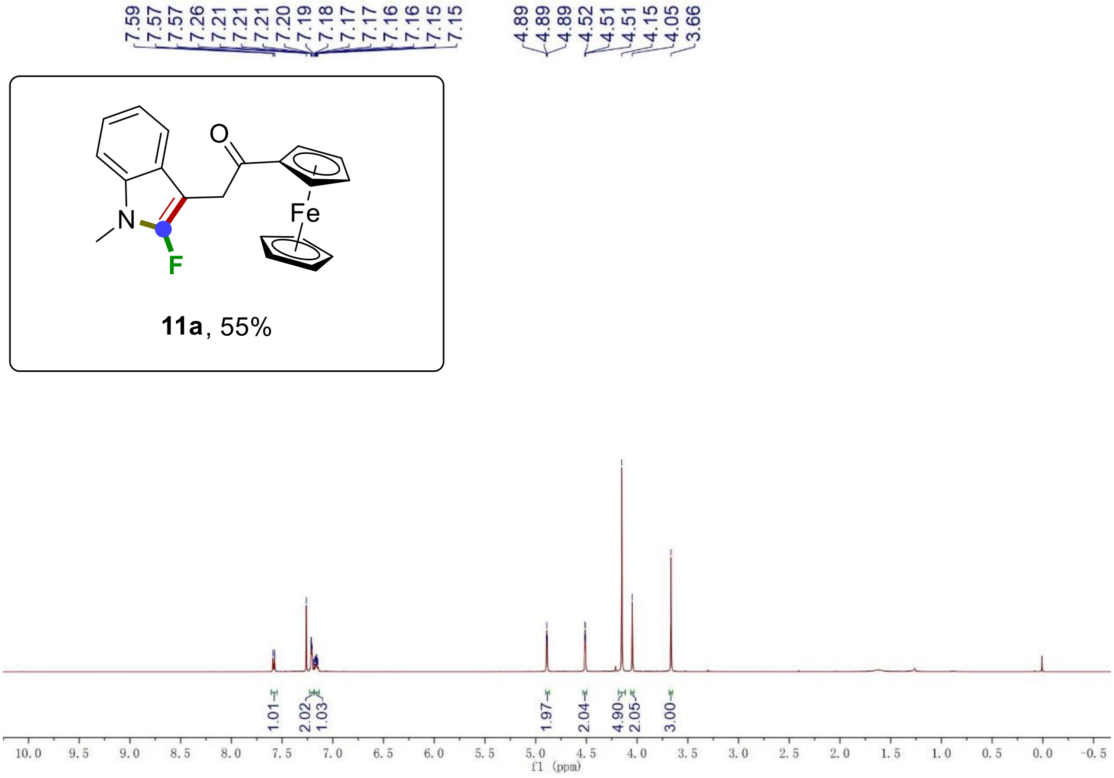


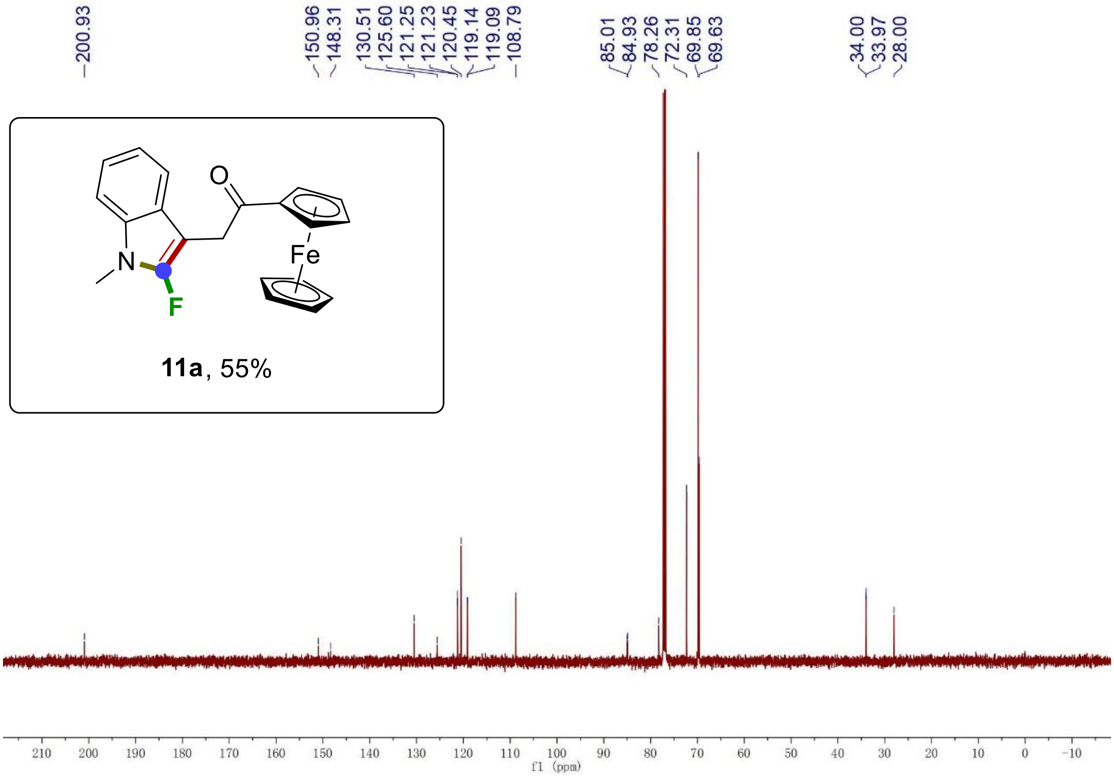

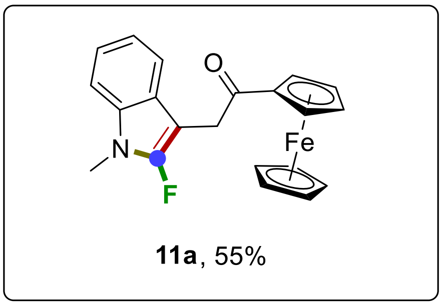


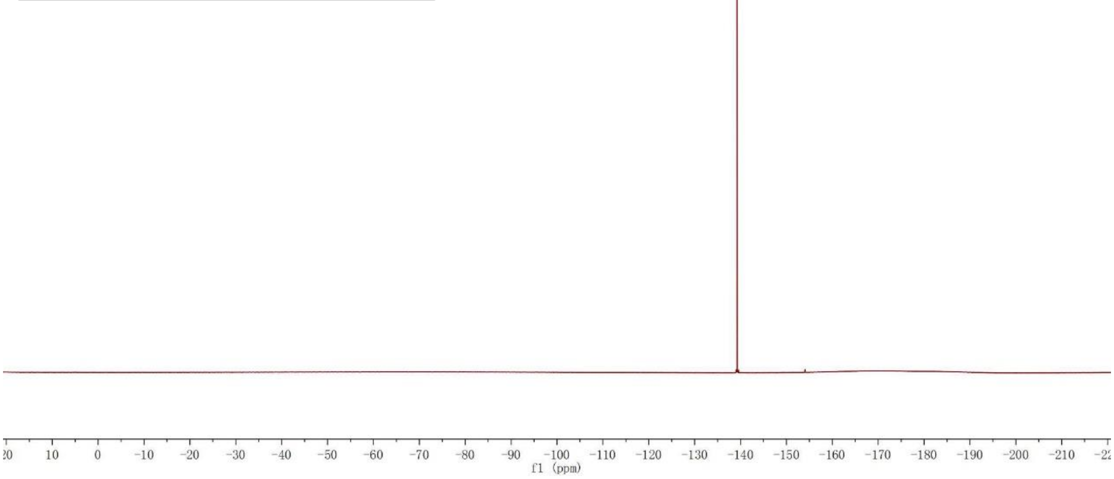


Supplementary Figure 162. $^{1}$H NMR, $^{13}$C NMR and $^{19}$F NMR spectrum of **11a**.

**4-(2-(2-fluoro-1-methyl-1H-indol-3-yl)acetyl)phenyl (tert-butoxycarbonyl)-D-valinate (11b)**

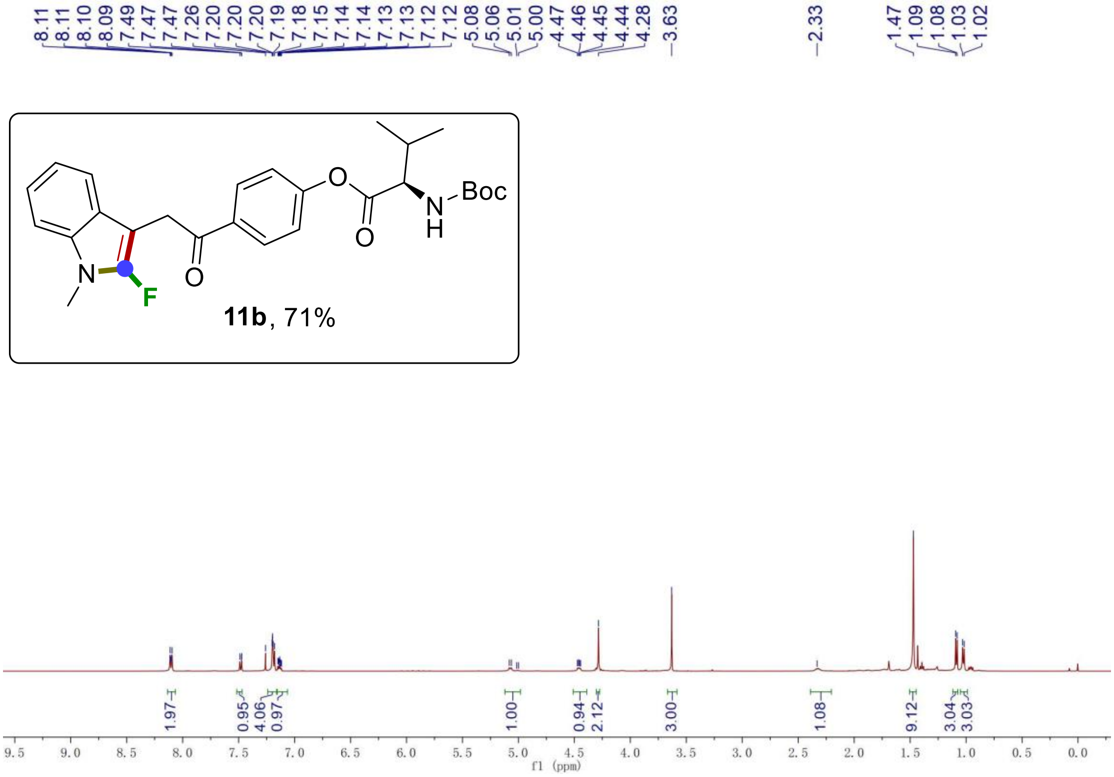


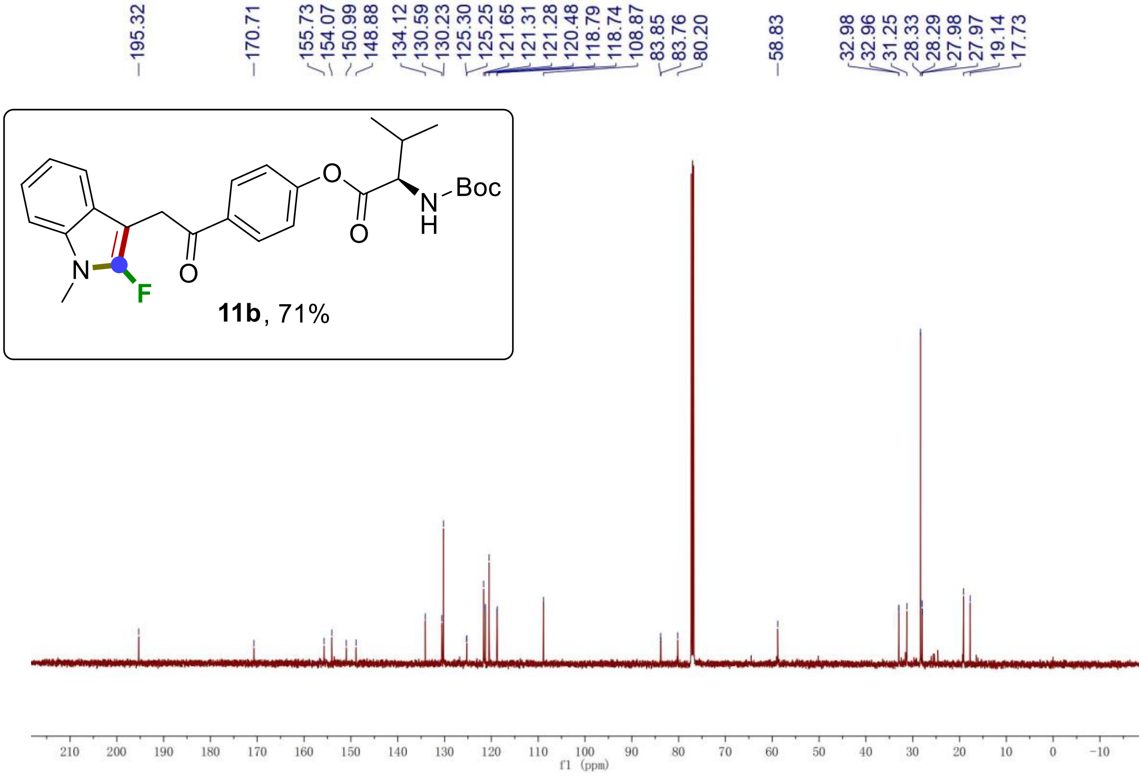

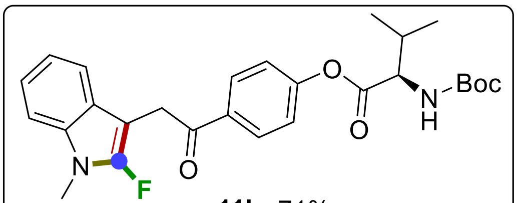
**11b**, 71%

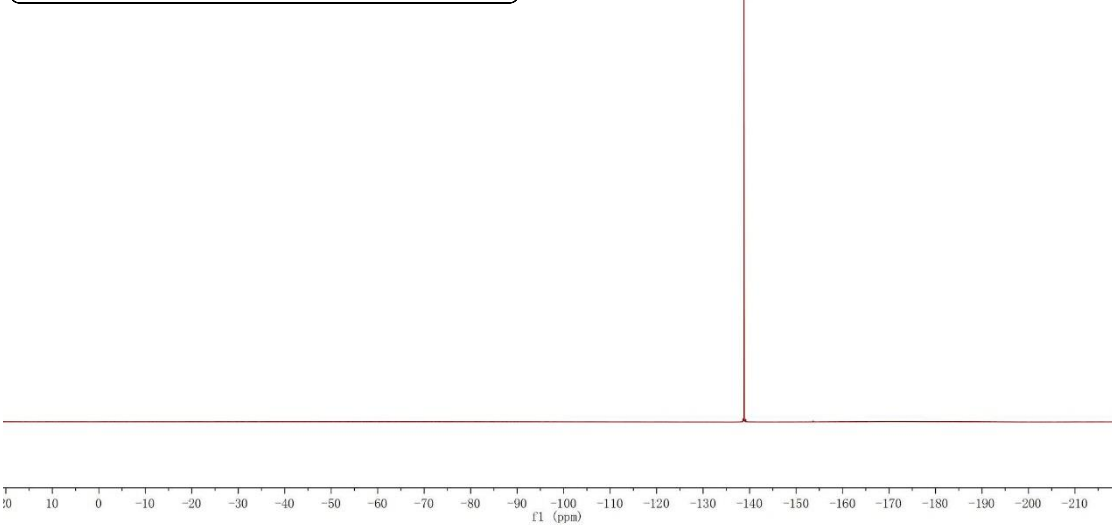


Supplementary Figure 163. $^{1}H$ NMR, $^{13}C$ NMR and $^{19}F$ NMR spectrum of **11b**.

**1-(tert-butyl) 2-(4-(2-(2-fluoro-1-methyl-1H-indol-3-yl)acetyl)phenyl) (S)-pyrrolidine-1,2-dicarboxylate (11c)**

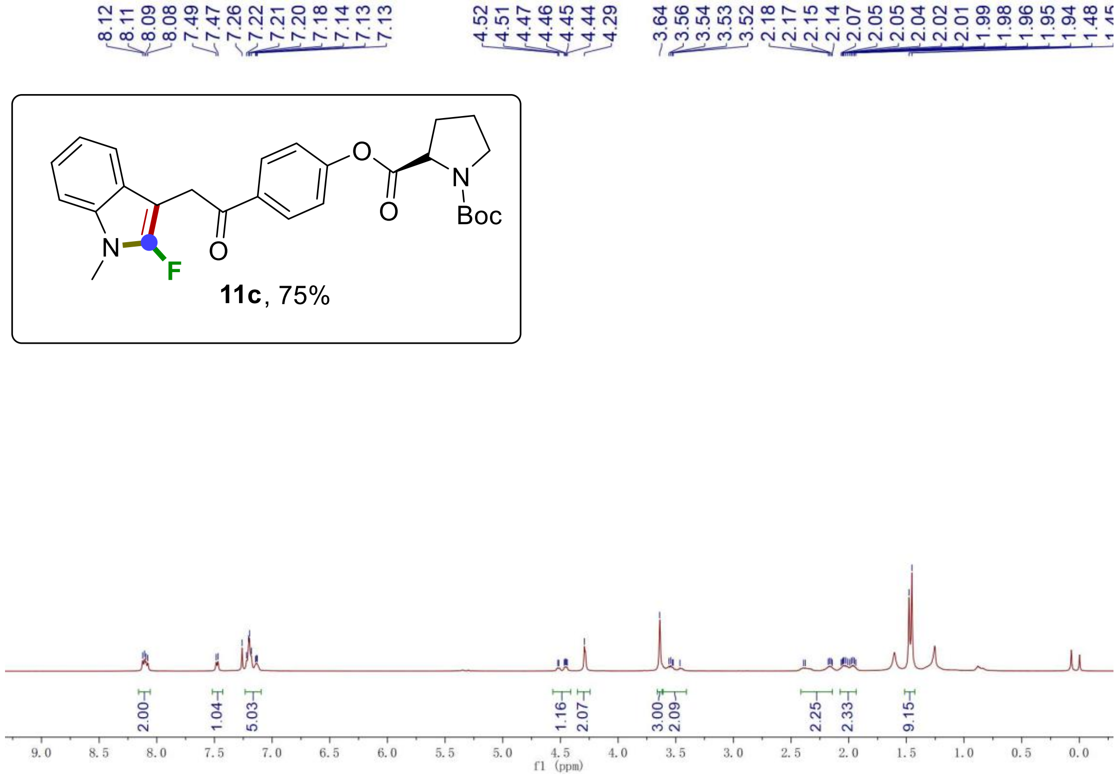


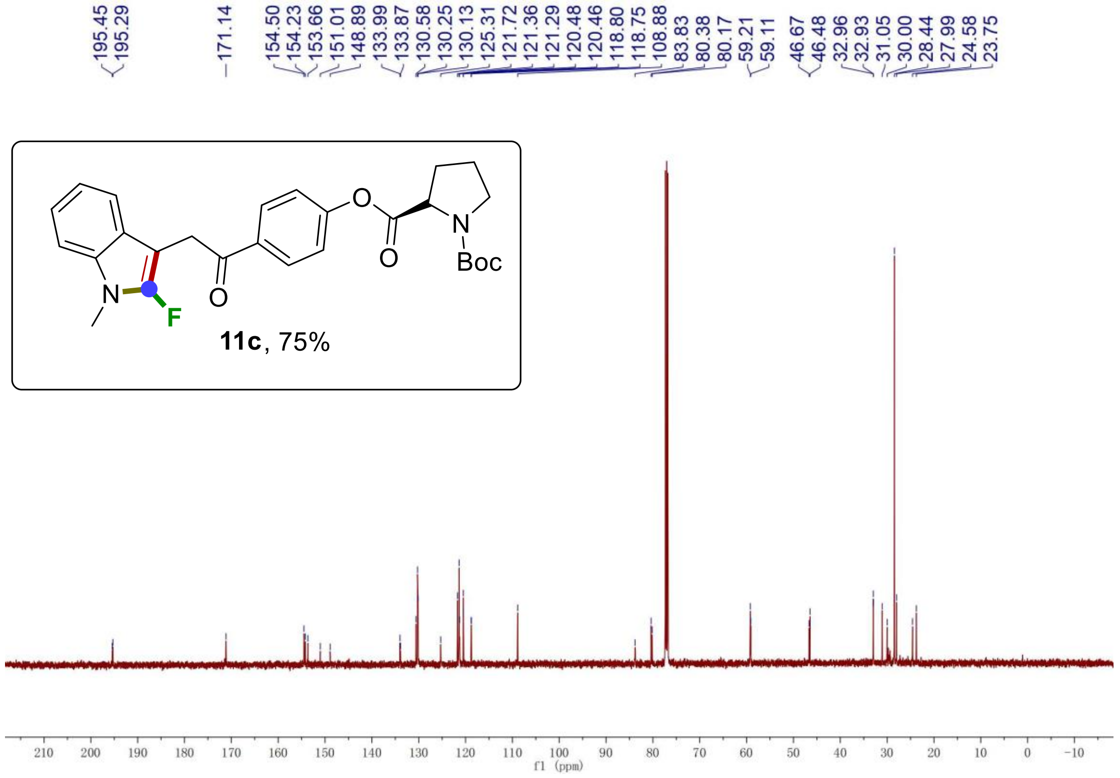

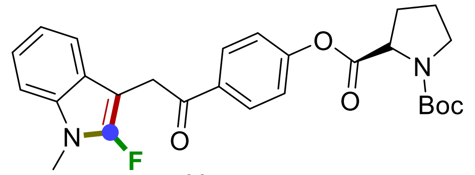

**11c**, 75%

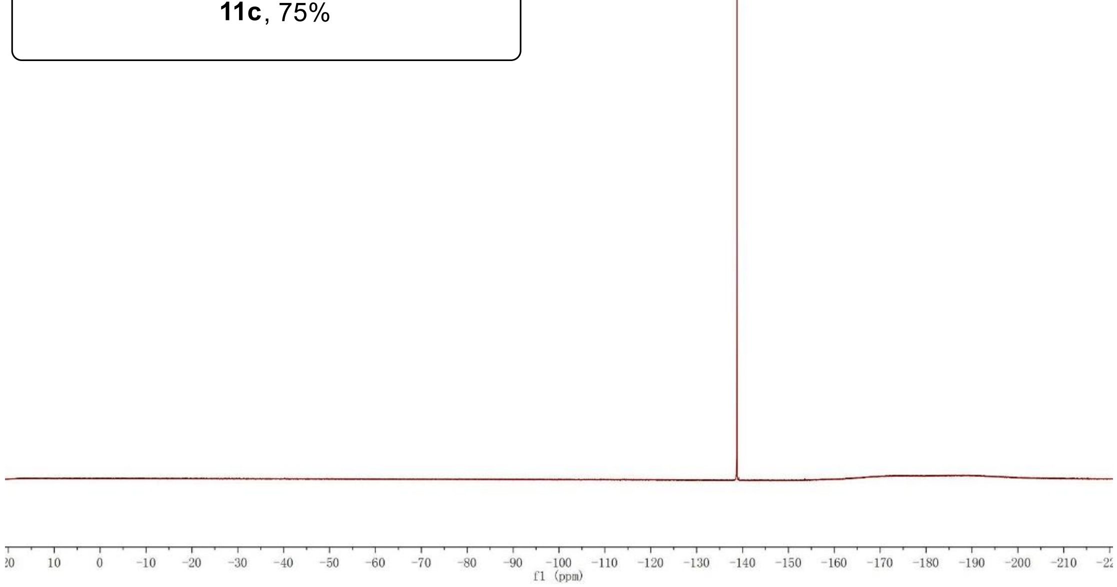


Supplementary Figure 164. $^{1}H$ NMR, $^{13}C$ NMR and $^{19}F$ NMR spectrum of **11c**.

**2-(2-fluoro-1-methyl-1H-indol-3-yl)-1-((3S,8S,9S,10R,14S,17R)-3-hydroxy-10-methyl-2,3,4,7,8,9,10,11,12,13,14,15,16,17-tetradecahydro-1H-cyclopenta[a]phenanthren-17-yl)ethan-1-one (11d)**

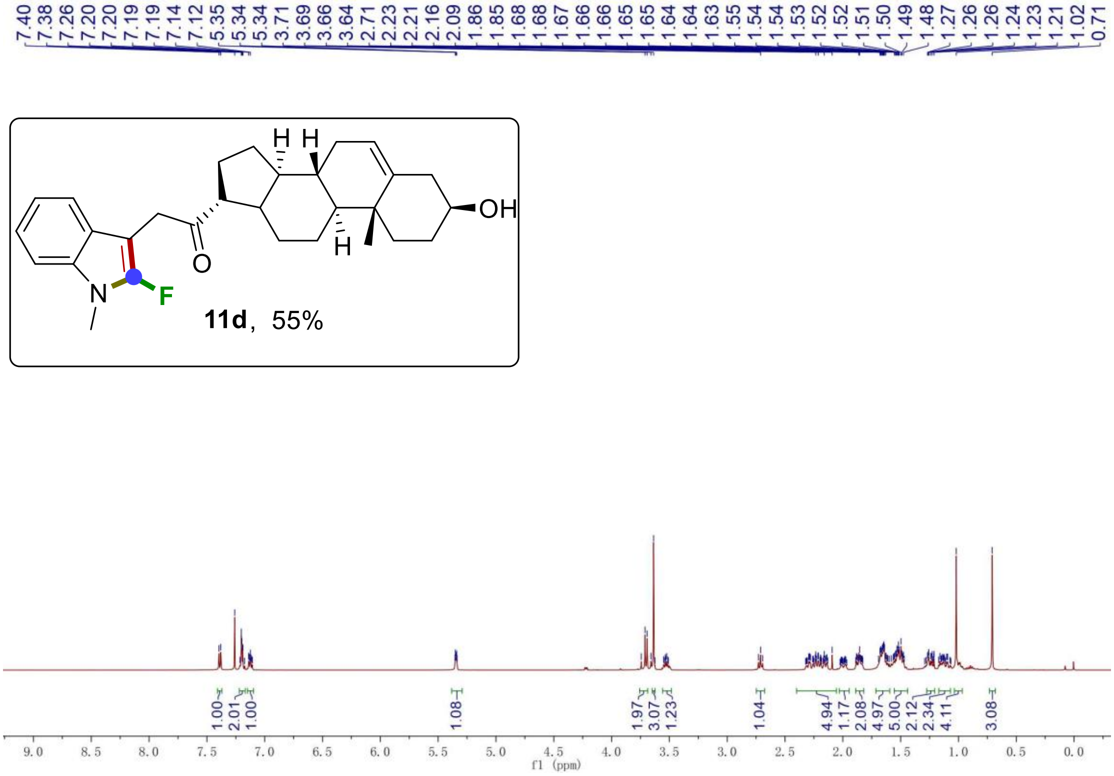


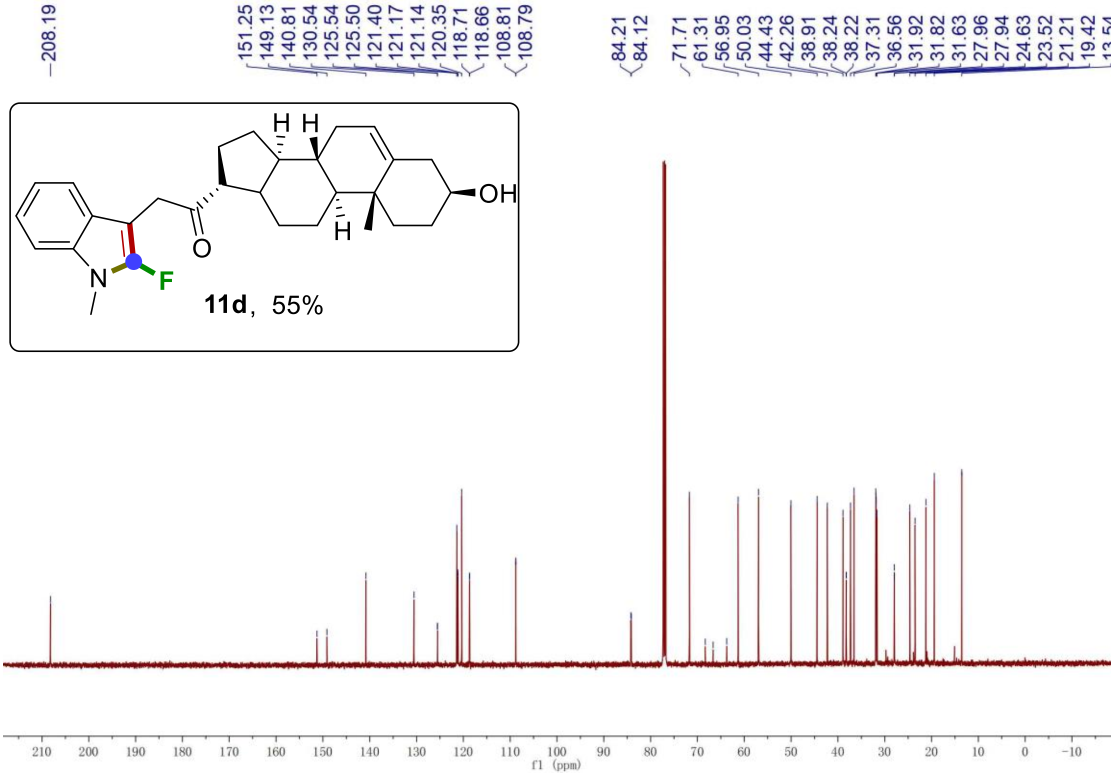

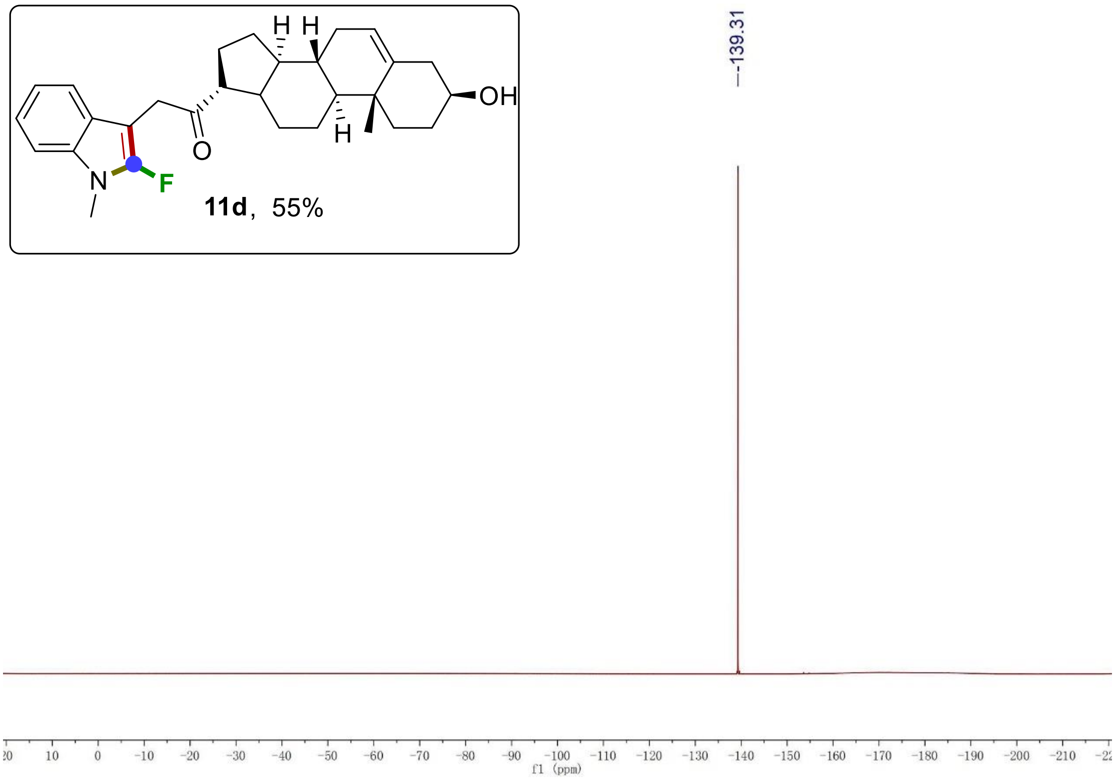


Supplementary Figure 165. $^{1}$H NMR, $^{13}$C NMR and $^{19}$F NMR spectrum of **11d**.

**1-((1r,3R,5S,7s)-adamantan-1-yl)-2-(2-fluoro-1-methyl-1H-indol-3-yl)ethan-1-one (11e)**

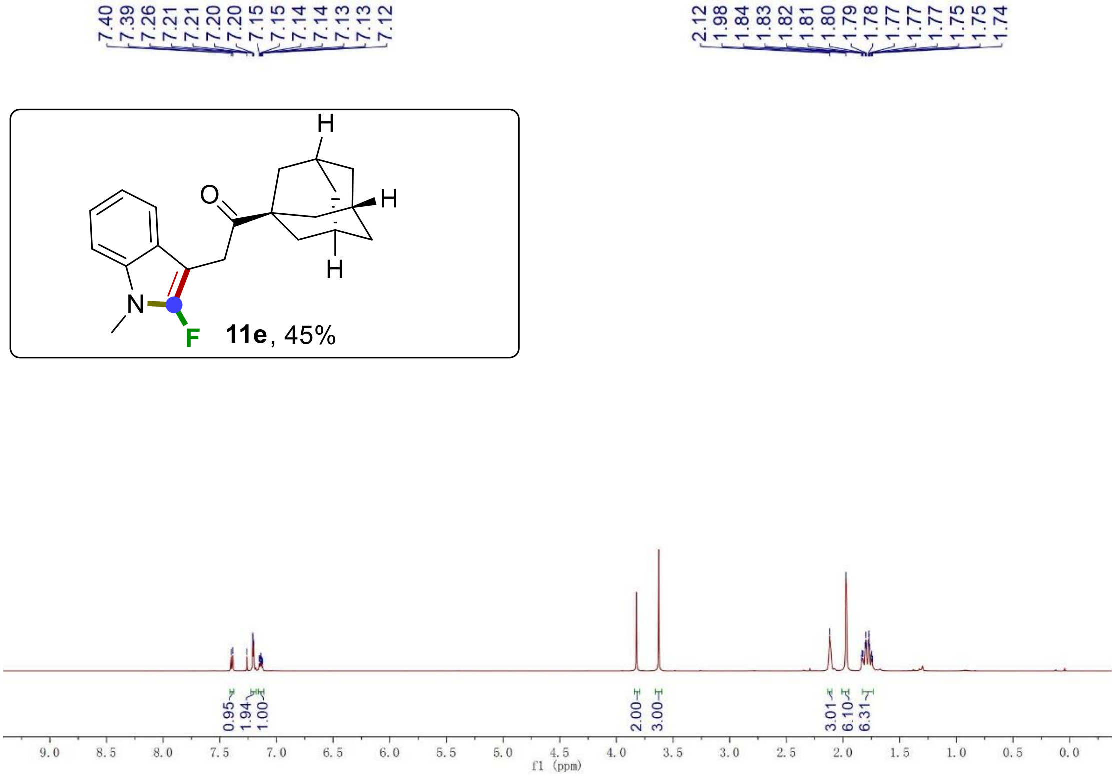


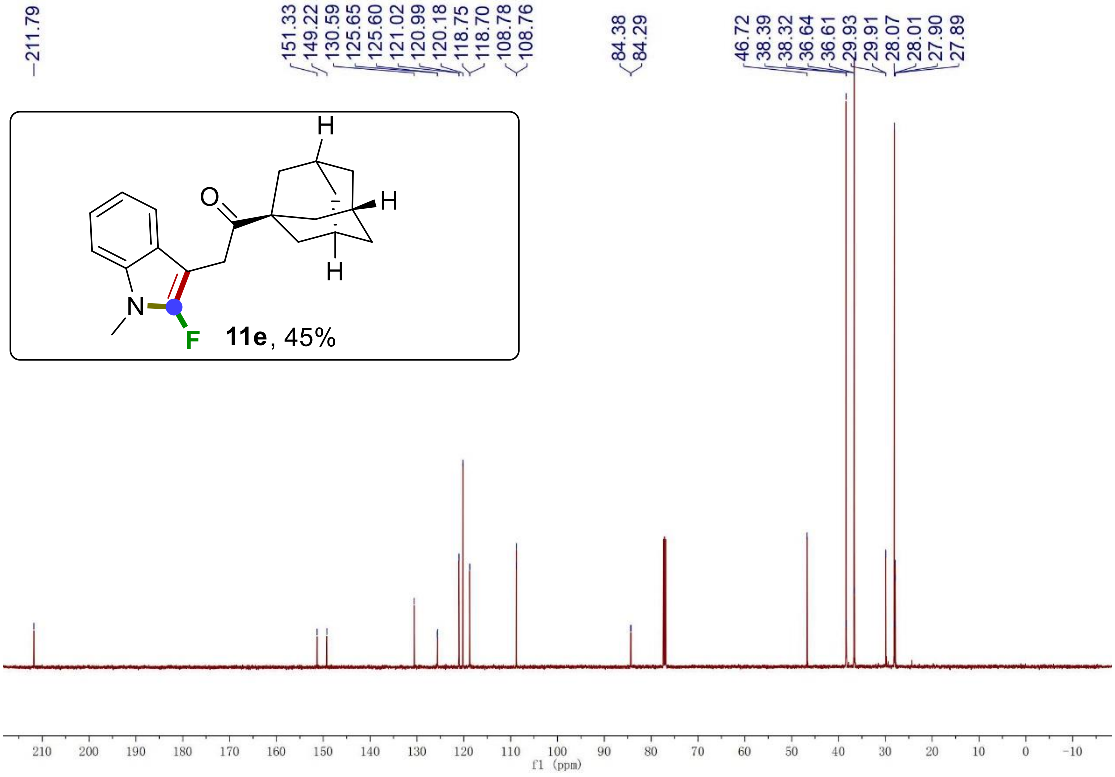

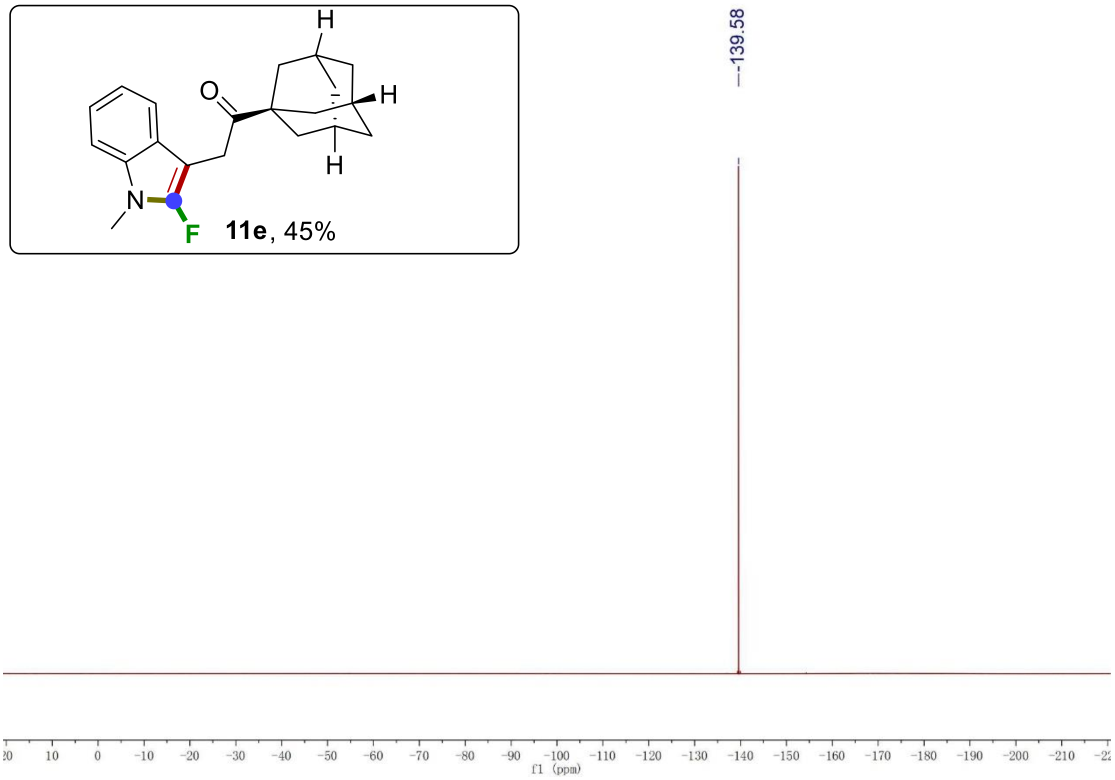


Supplementary Figure 166. $^{1}H$ NMR, $^{13}C$ NMR and $^{19}F$ NMR spectrum of **11e**.

**(8S,9S,10R,14S,17R)-17-(2-(2-fluoro-1-methyl-1H-indol-3-yl)acetyl)-10-methyl-1,2,6,7,8,9,10,11,12,13,14,15,16,17-tetradecahydro-3H-cyclopenta[a]phenanthren-3-one (11f)**

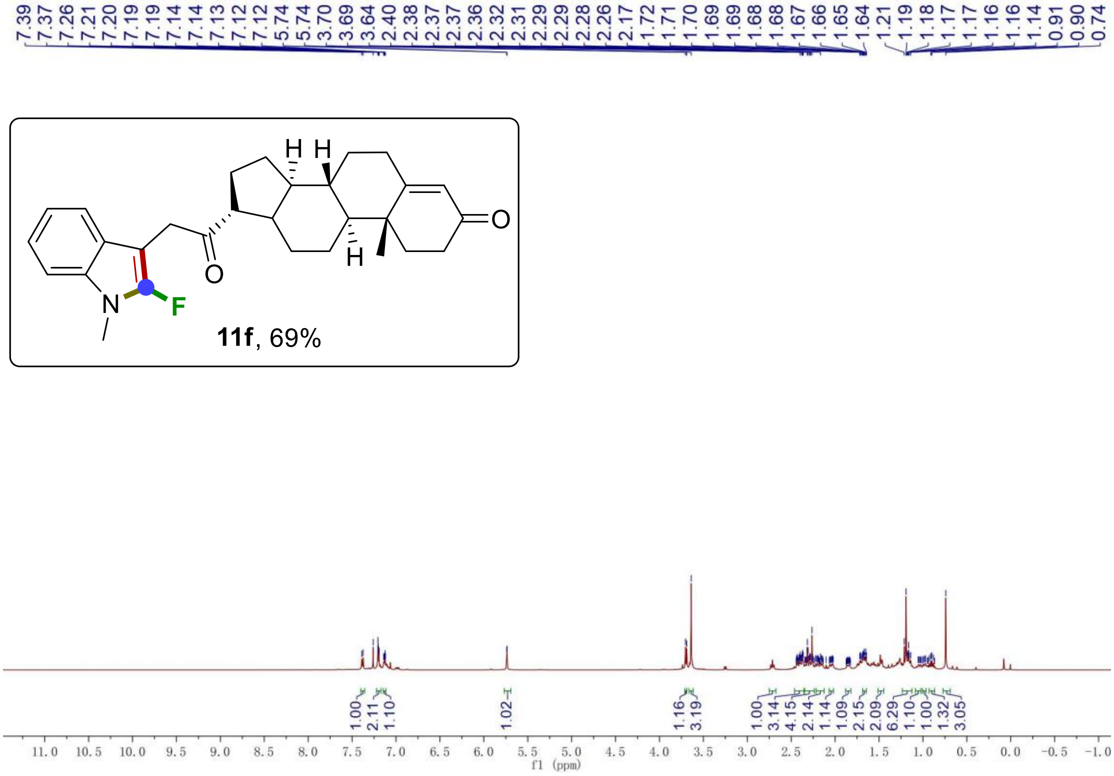


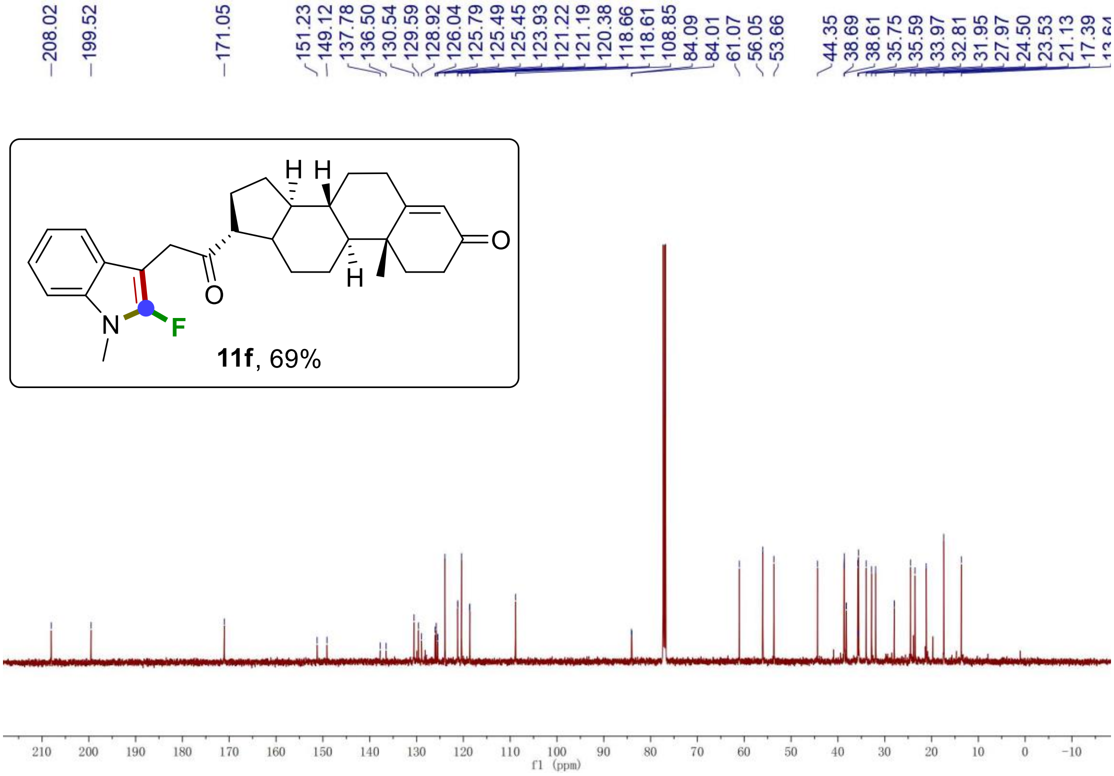

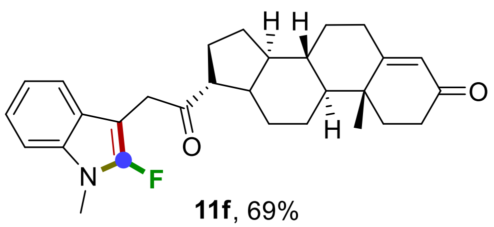

**11f**, 69%

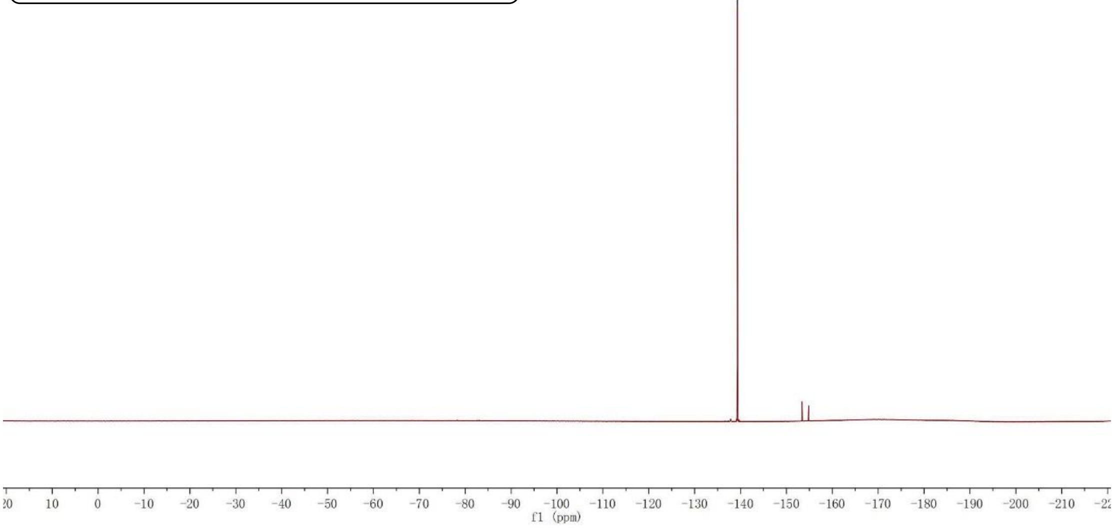


Supplementary Figure 167. $^{1}H$ NMR, $^{13}C$ NMR and $^{19}F$ NMR spectrum of **11f**.

**(E)-3,7-dimethylocta-2,6-dien-1-yl 2-(2-fluoro-1-methyl-1H-indol-3-yl)acetate (11g)**

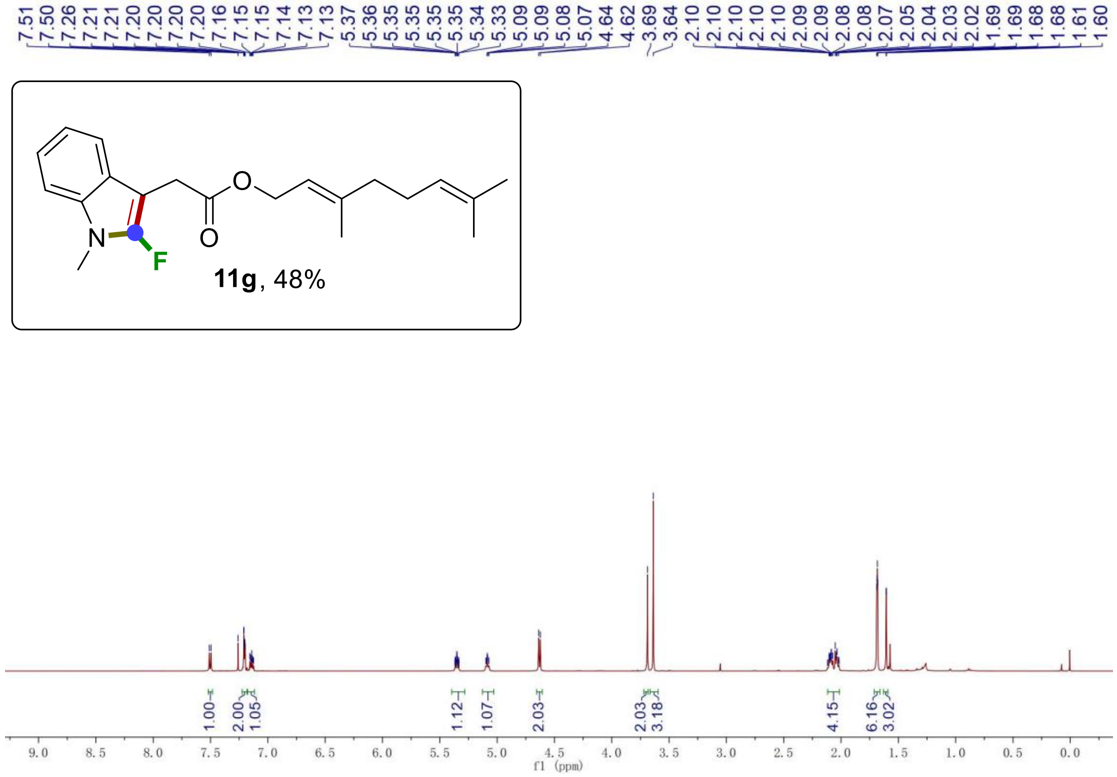


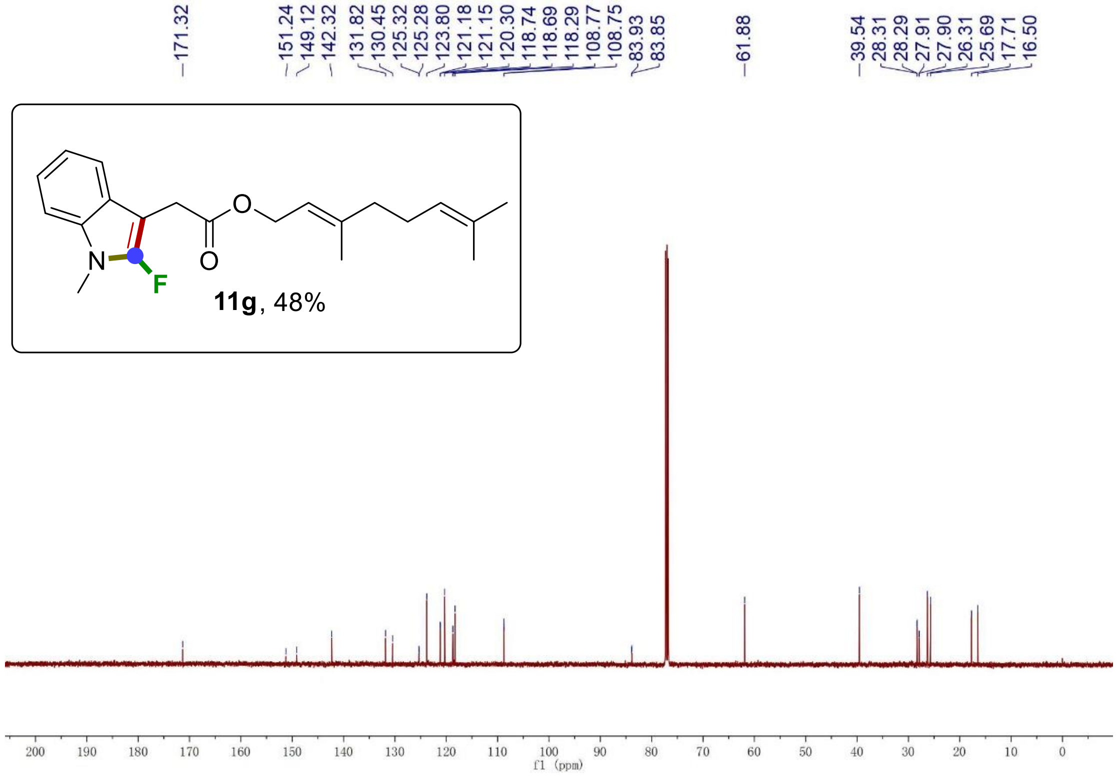

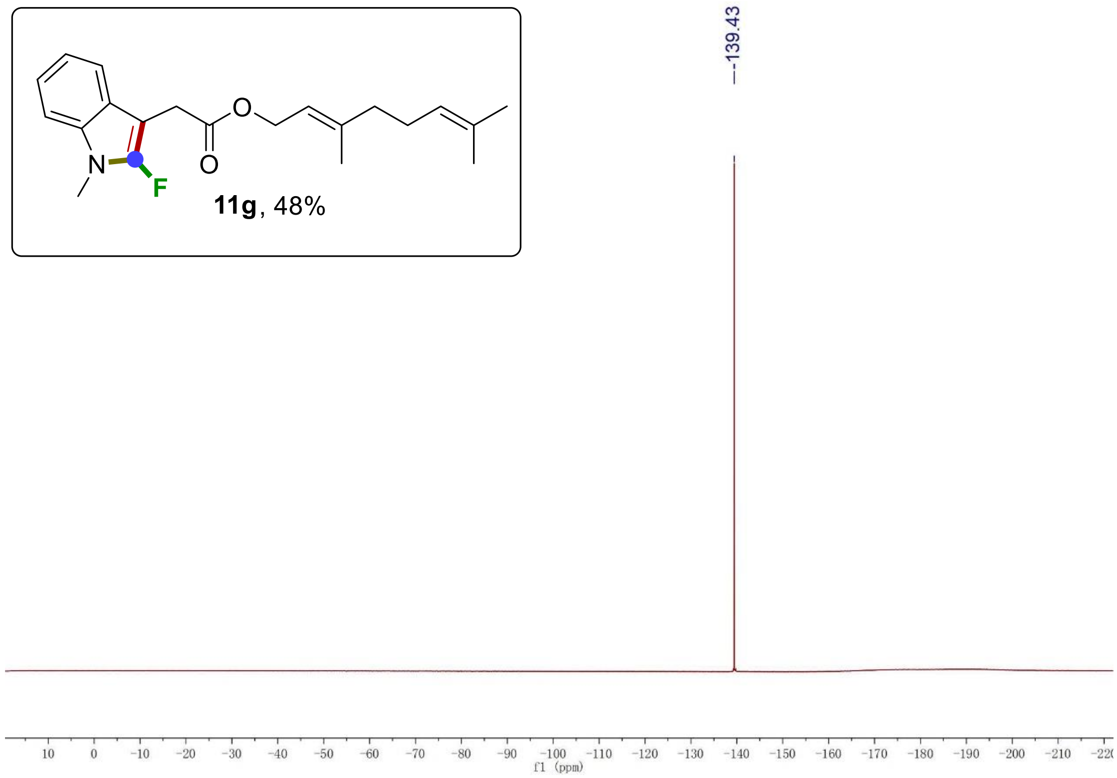

Supplementary Figure 168. $^{1}$H NMR, $^{13}$C NMR and $^{19}$F NMR spectrum of **11g**.

**2-((1R,5S)-6,6-dimethylbicyclo[3.1.1]hept-2-en-3-yl)ethyl 2-(2-fluoro-1-methyl-1H-indol-3-yl)acetate (11h)**

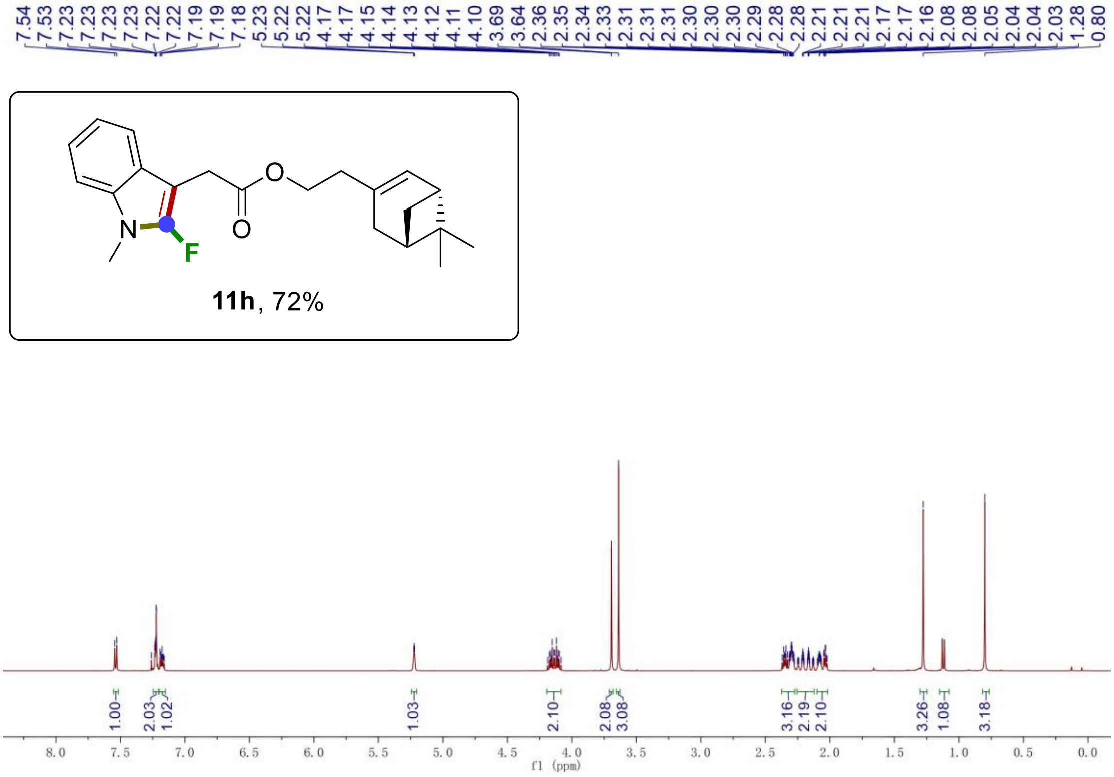


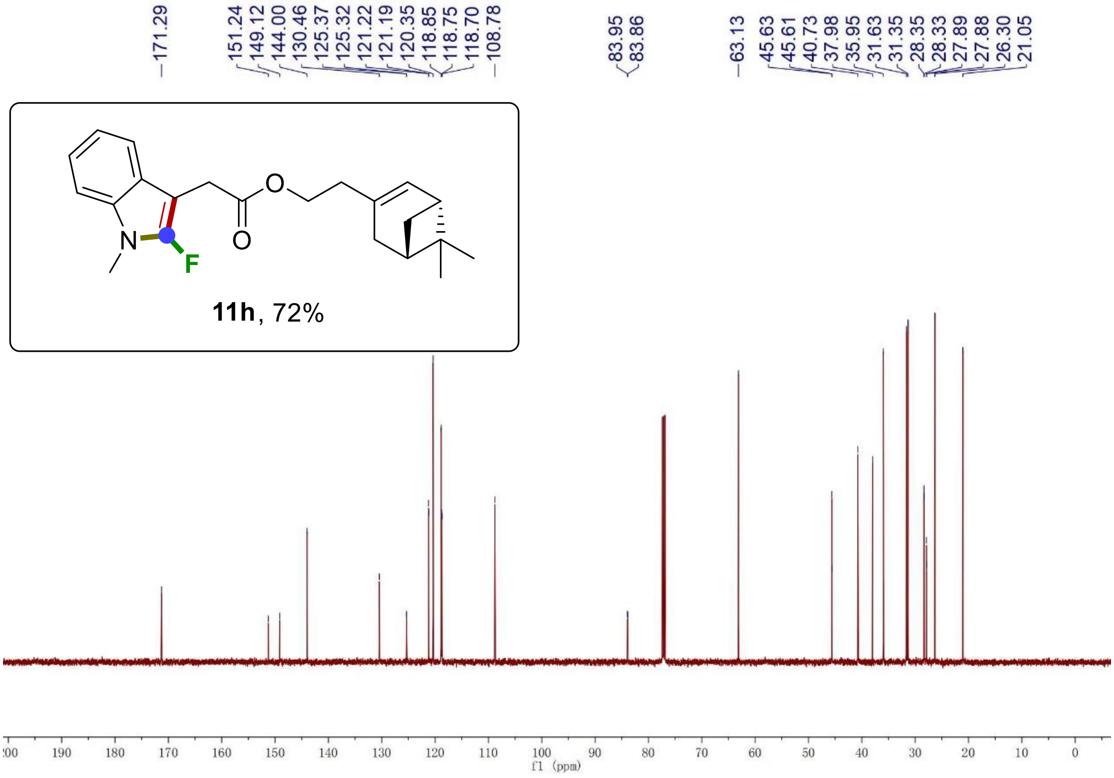

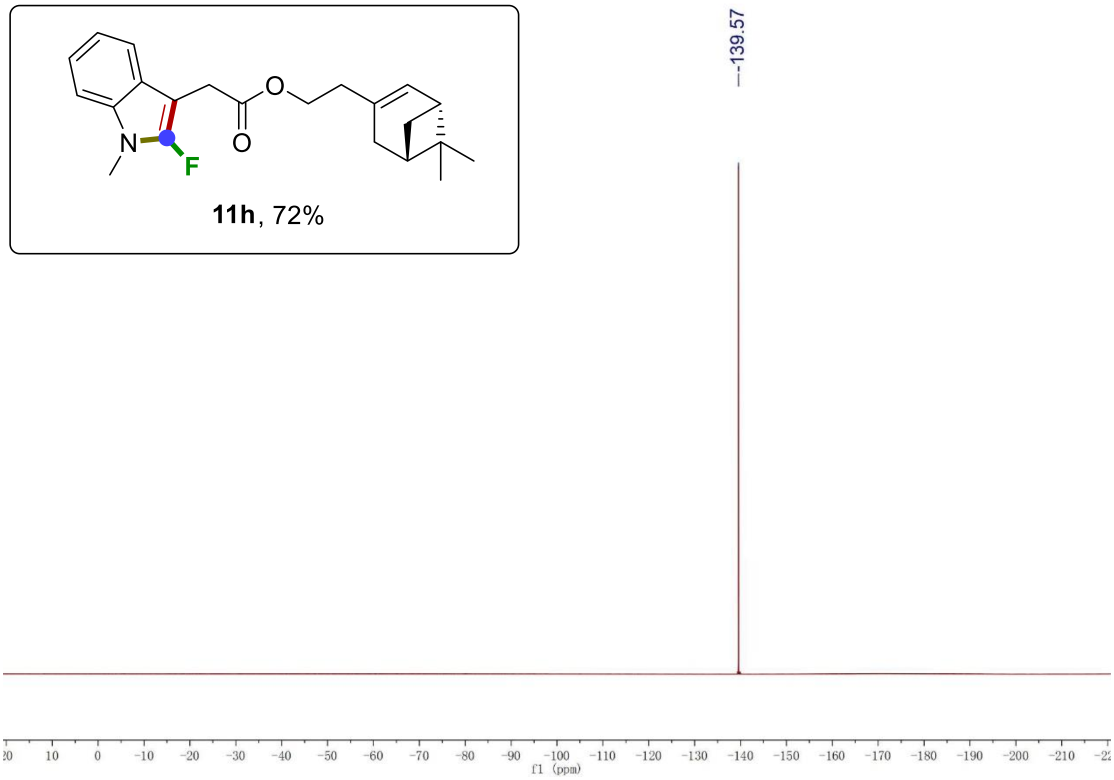


Supplementary Figure 169. $^{1}H$ NMR, $^{13}C$ NMR and $^{19}F$ NMR spectrum of **11h**.

**(1R,2S,5R)-2-isopropyl-5-methylcyclohexyl 2-(2-fluoro-1-methyl-1H-indol-3-yl)acetate (11i)**

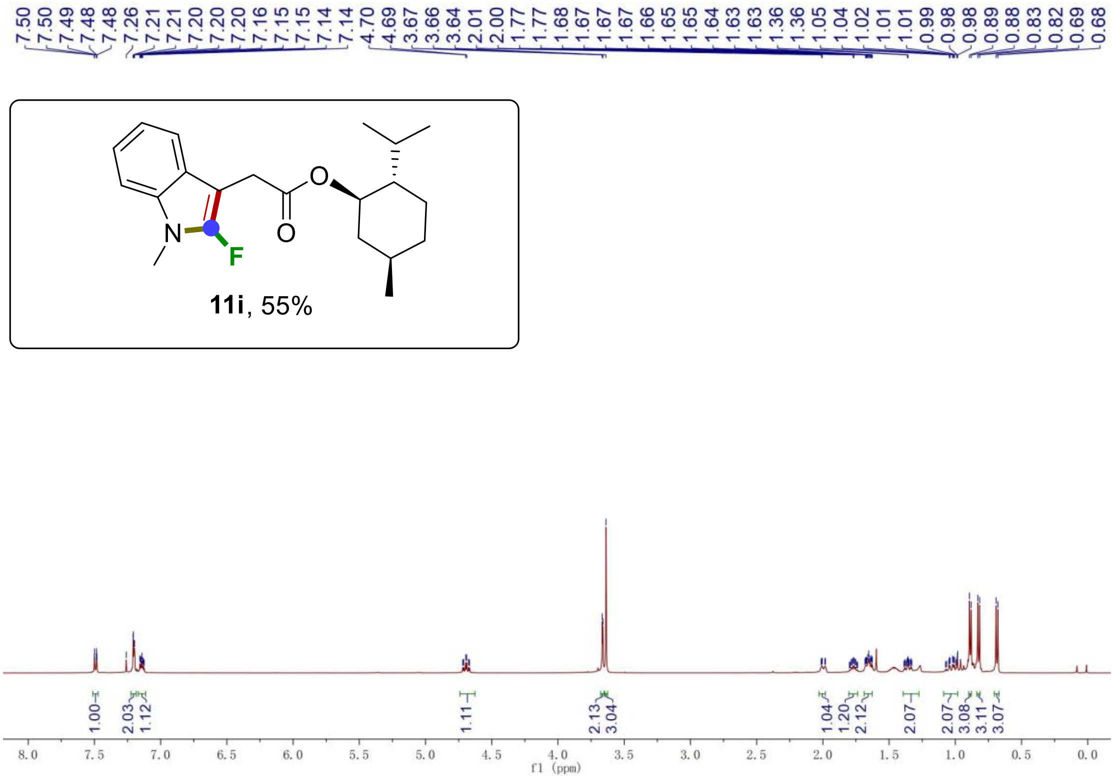


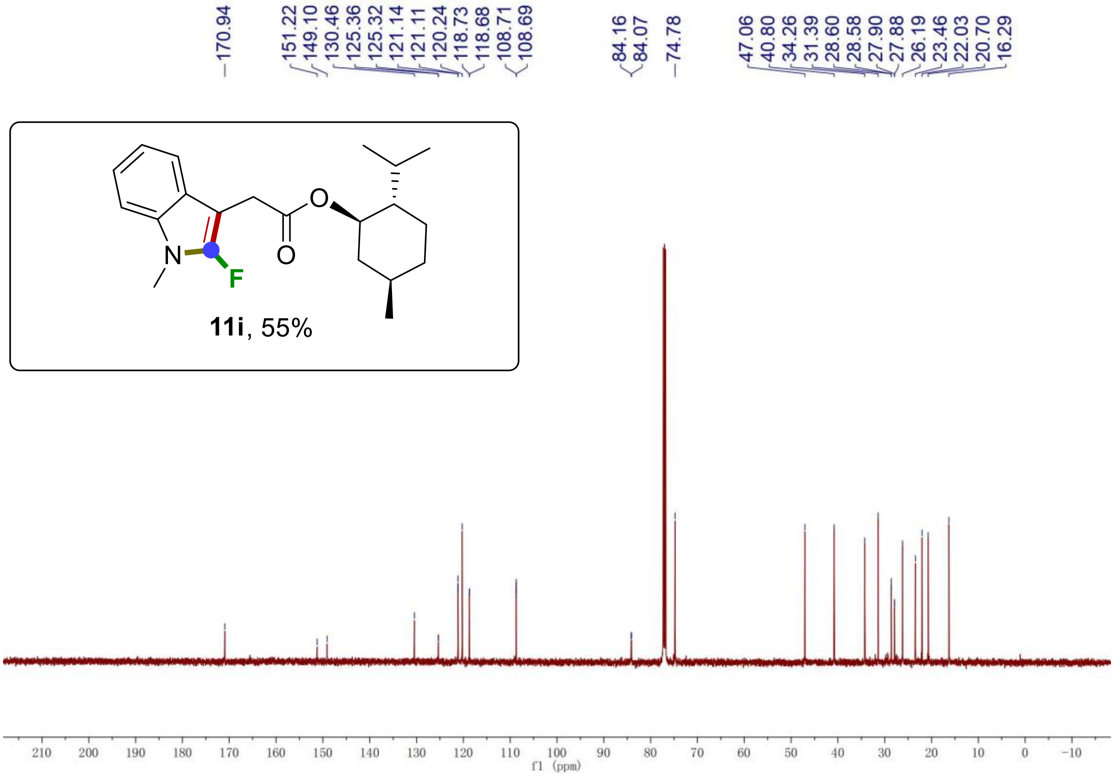

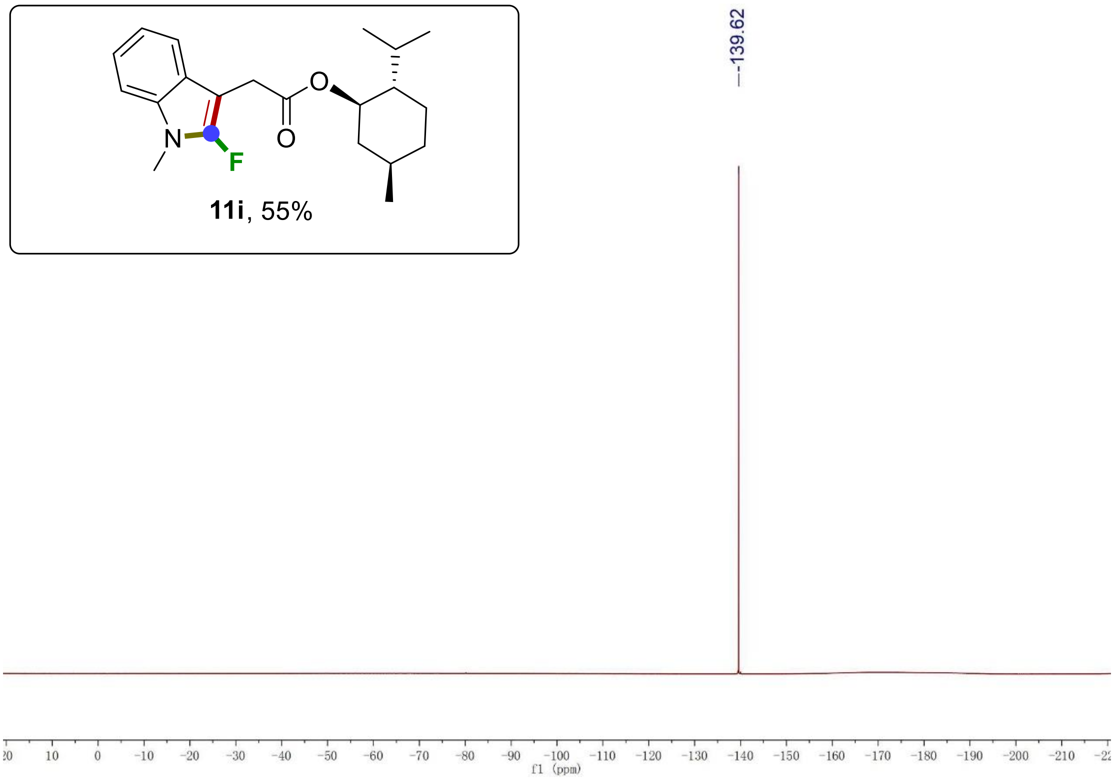


Supplementary Figure 170. $^{1}H$ NMR, $^{13}C$ NMR and $^{19}F$ NMR spectrum of **11i**.

**(R)-3,7-dimethyloct-6-en-1-yl 2-(2-fluoro-1-methyl-1H-indol-3-yl)acetate (11j)**

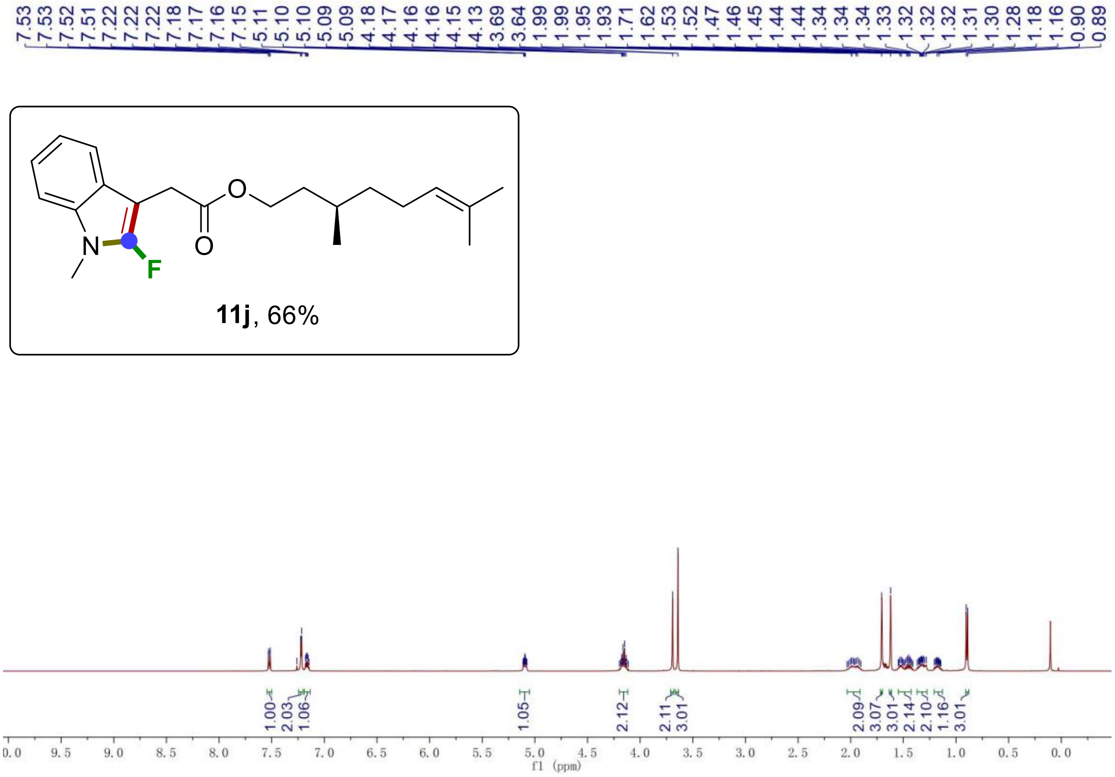


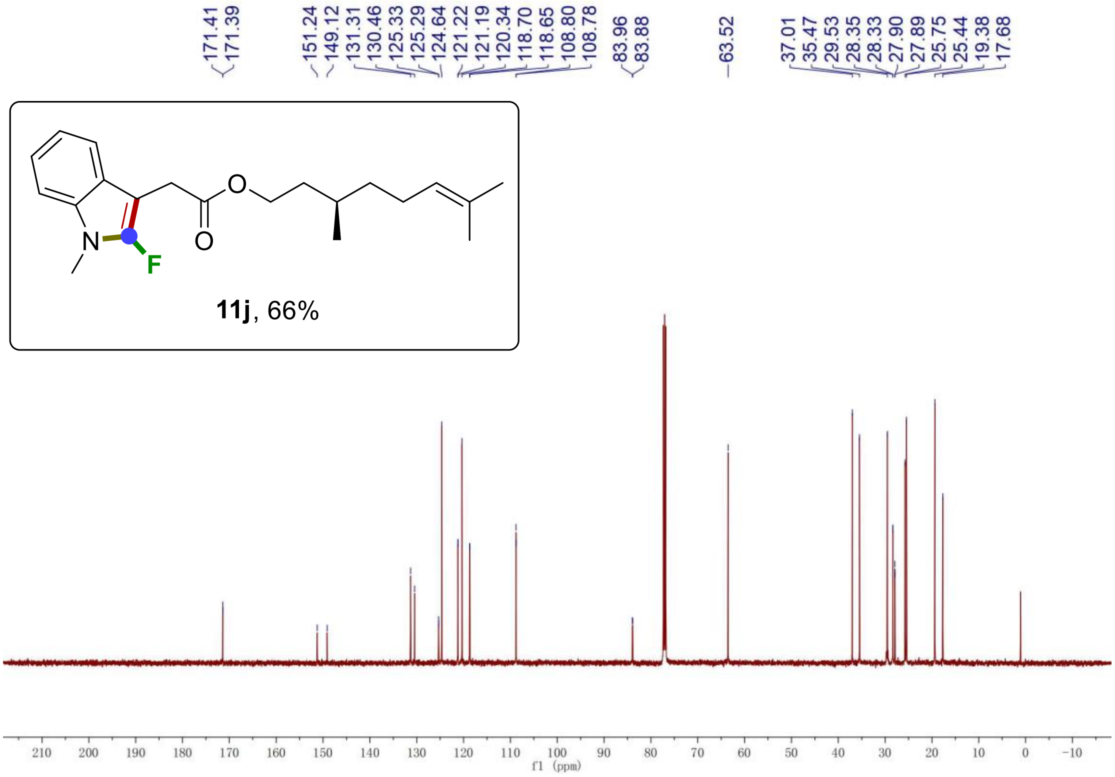

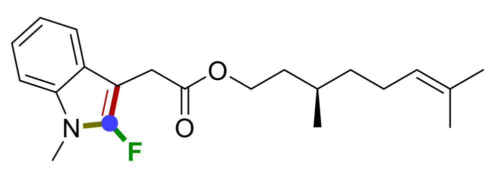

**11j**, 66%

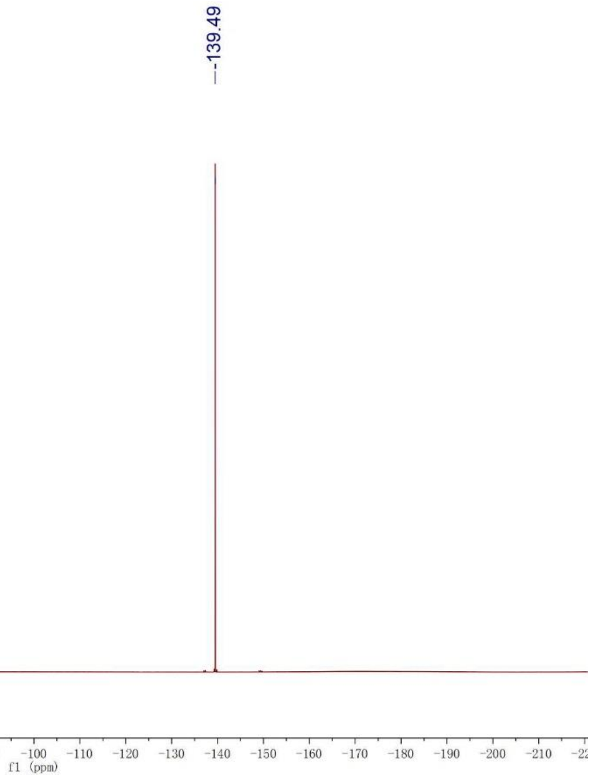


Supplementary Figure 171. $^{1}$H NMR, $^{13}$C NMR and $^{19}$F NMR spectrum of **11j**.

**(3aS,5R,6R,6aS)-5-((S)-2,2-dimethyl-1,3-dioxolan-4-yl)-2,2-dimethyltetrahydrofuro[2,3-d][1,3]dioxol-6-yl 2-(2-fluoro-1-methyl-1H-indol-3-yl)acetate (11k)**

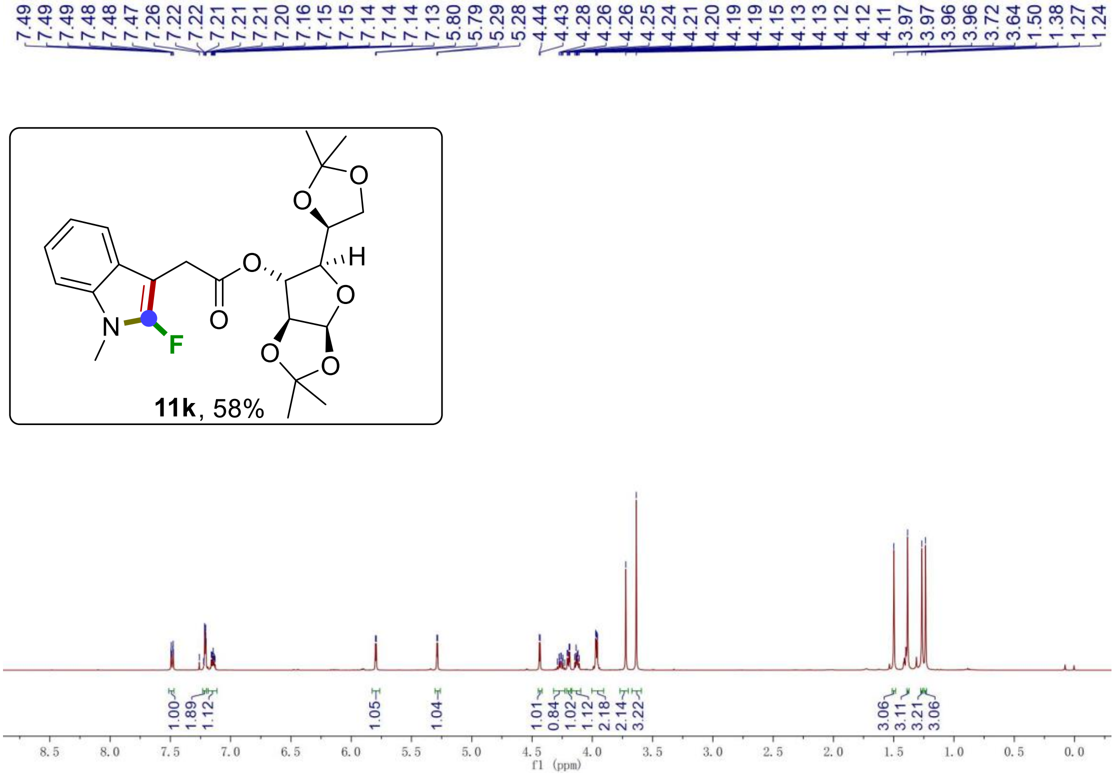


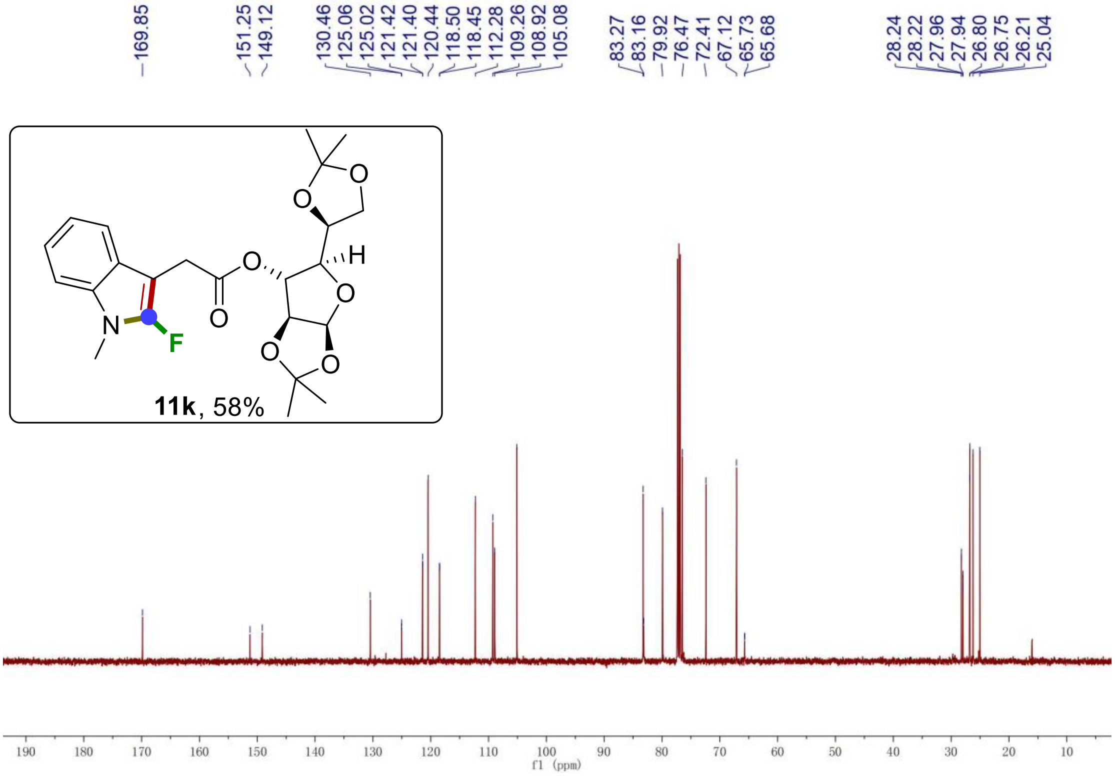

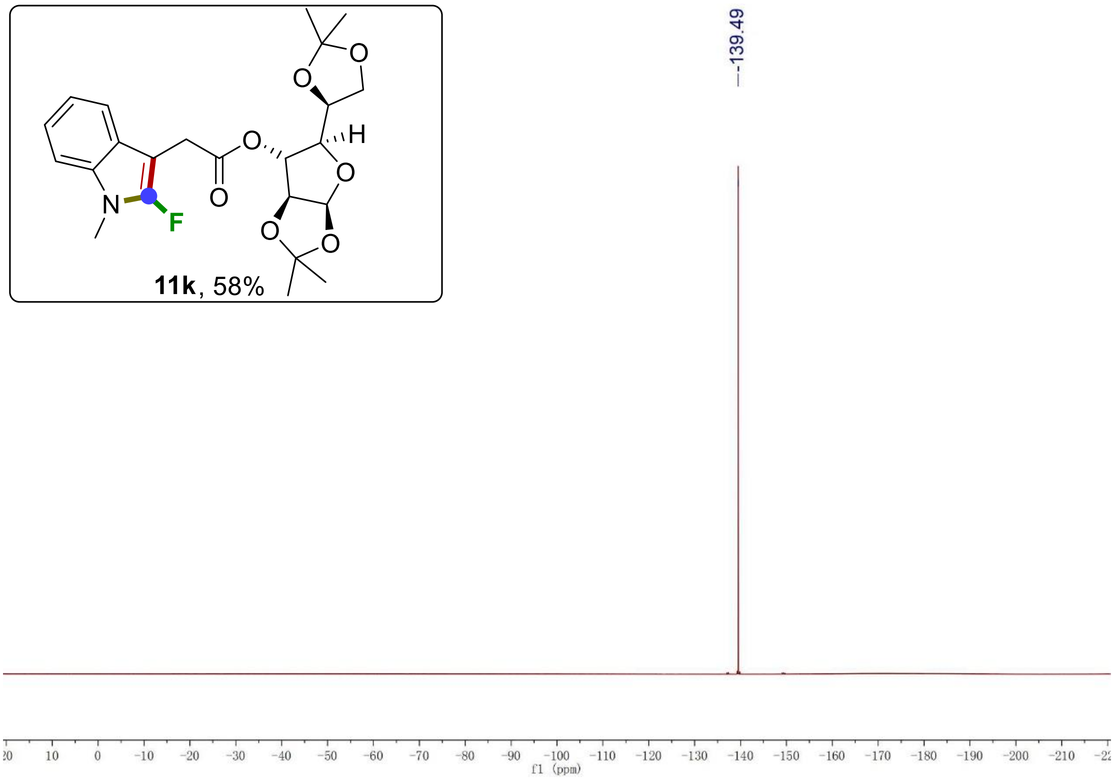


Supplementary Figure 172. $^{1}H$ NMR, $^{13}C$ NMR and $^{19}F$ NMR spectrum of **11k**.

**(3S,8S,9S,10R,13R,14S,17R)-10,13-dimethyl-17-((R)-6-methylheptan-2-yl)-2,3,4,7,8,9,10,11,12,13,14,15,16,17-tetradecahydro-1H-cyclopenta[a]phenanthren-3-yl 2-(2-fluoro-1-methyl-1H-indol-3-yl)acetate (11l)**

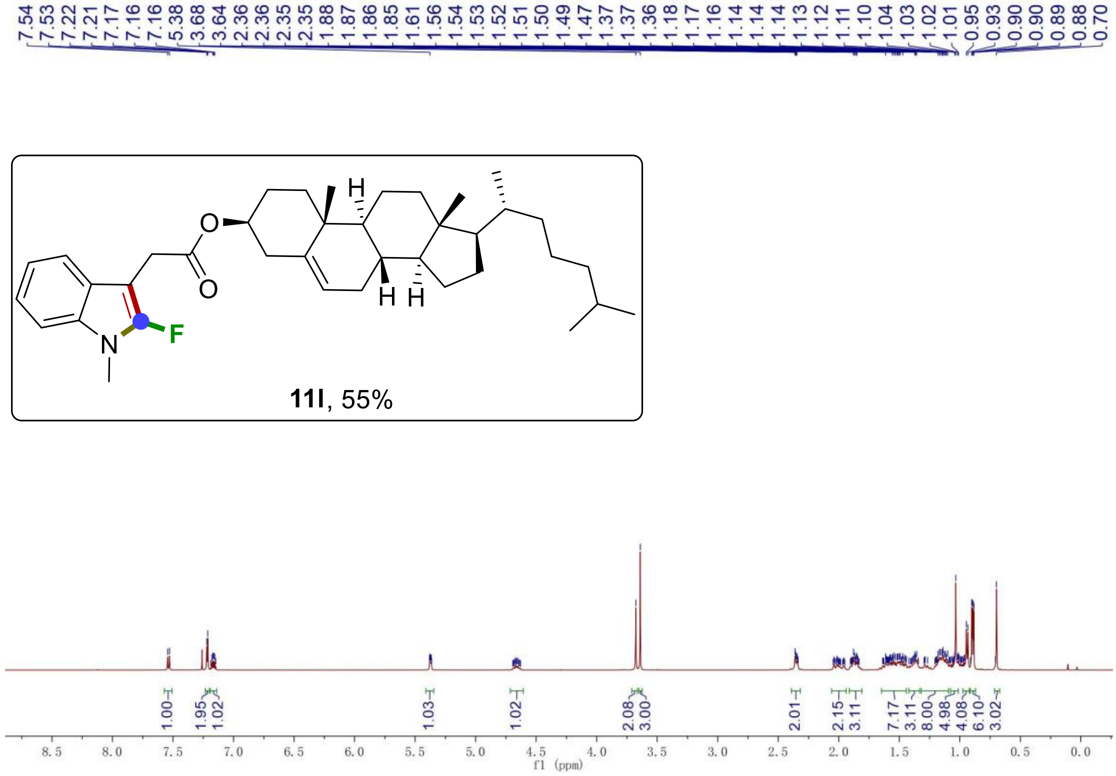


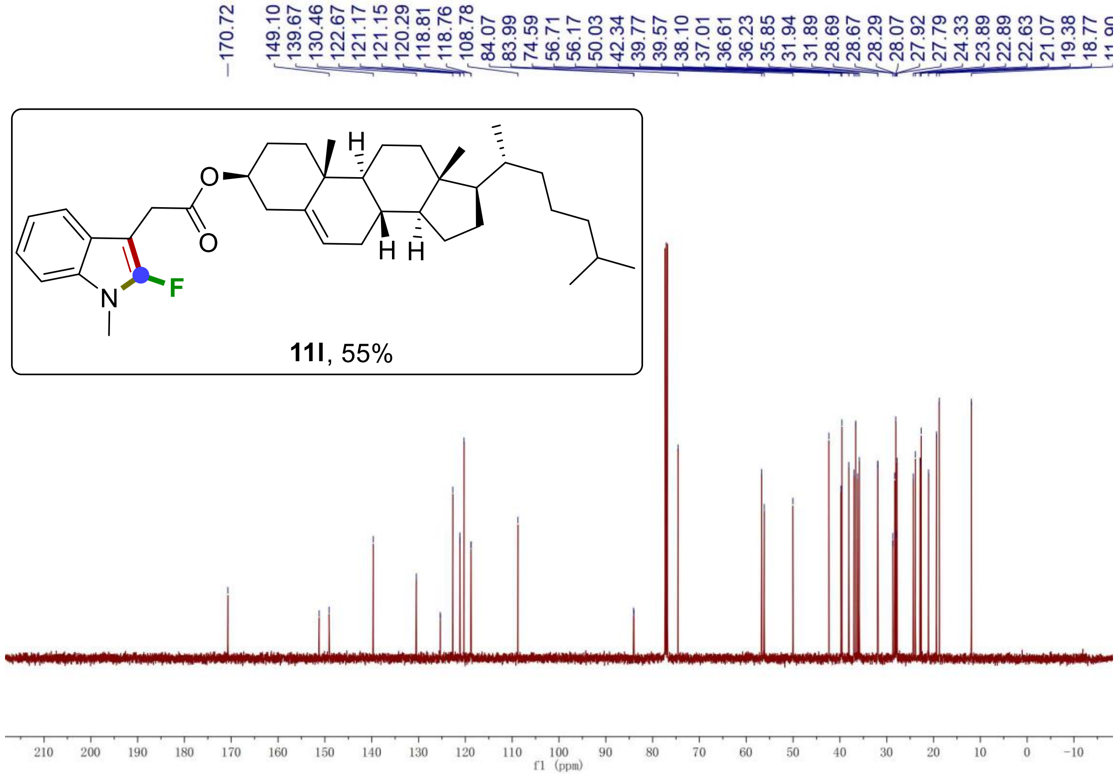

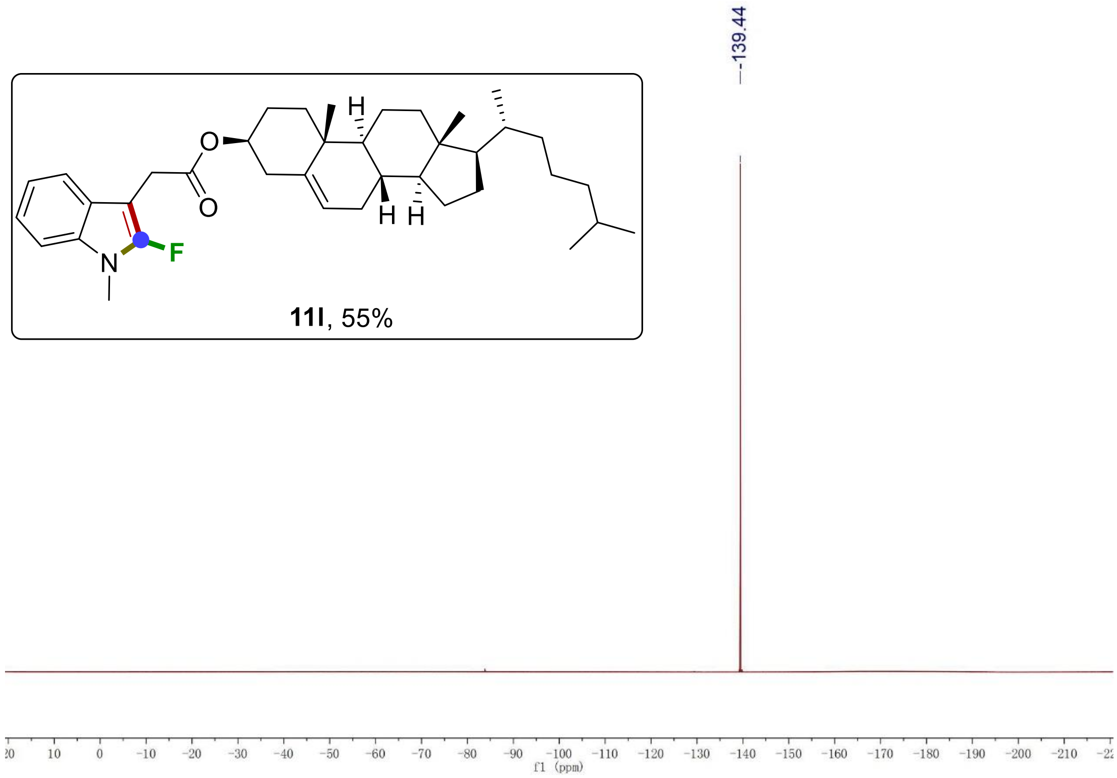


Supplementary Figure 173. $^{1}H$ NMR, $^{13}C$ NMR and $^{19}F$ NMR spectrum of **11l**.

**4-(2-(2-fluoro-1-methyl-1H-indol-3-yl)acetyl)phenyl (R)-2-(4-isobutylphenyl)propanoate (11m)**

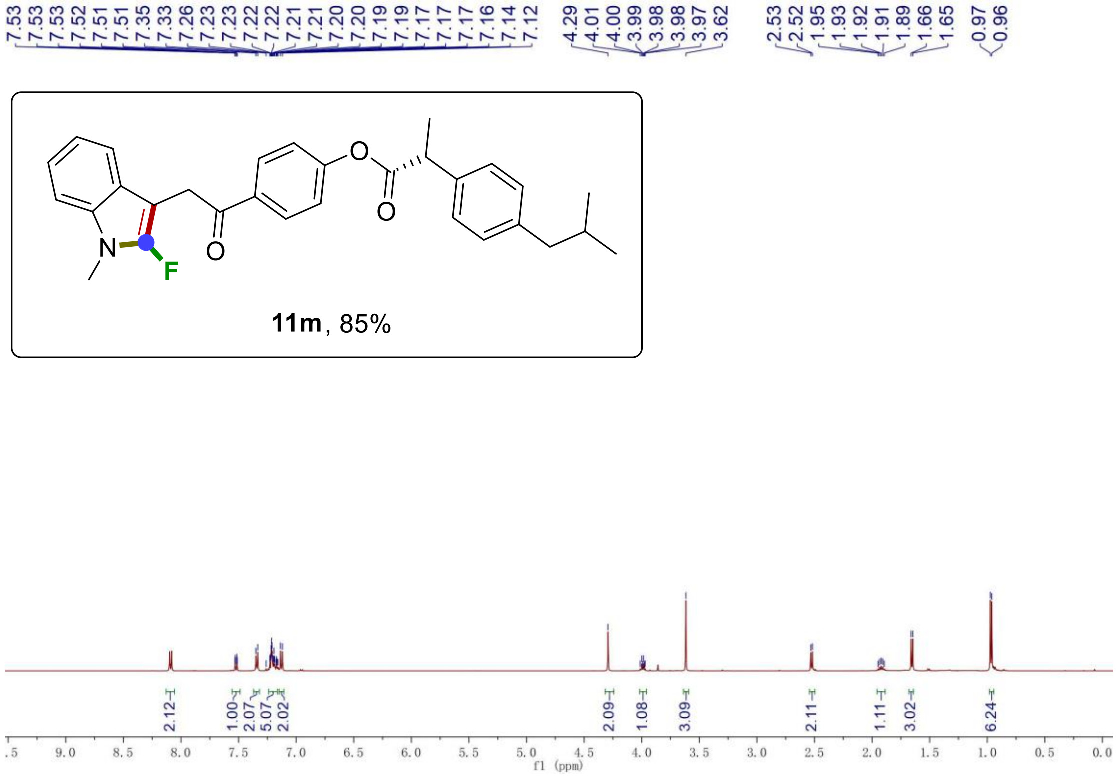


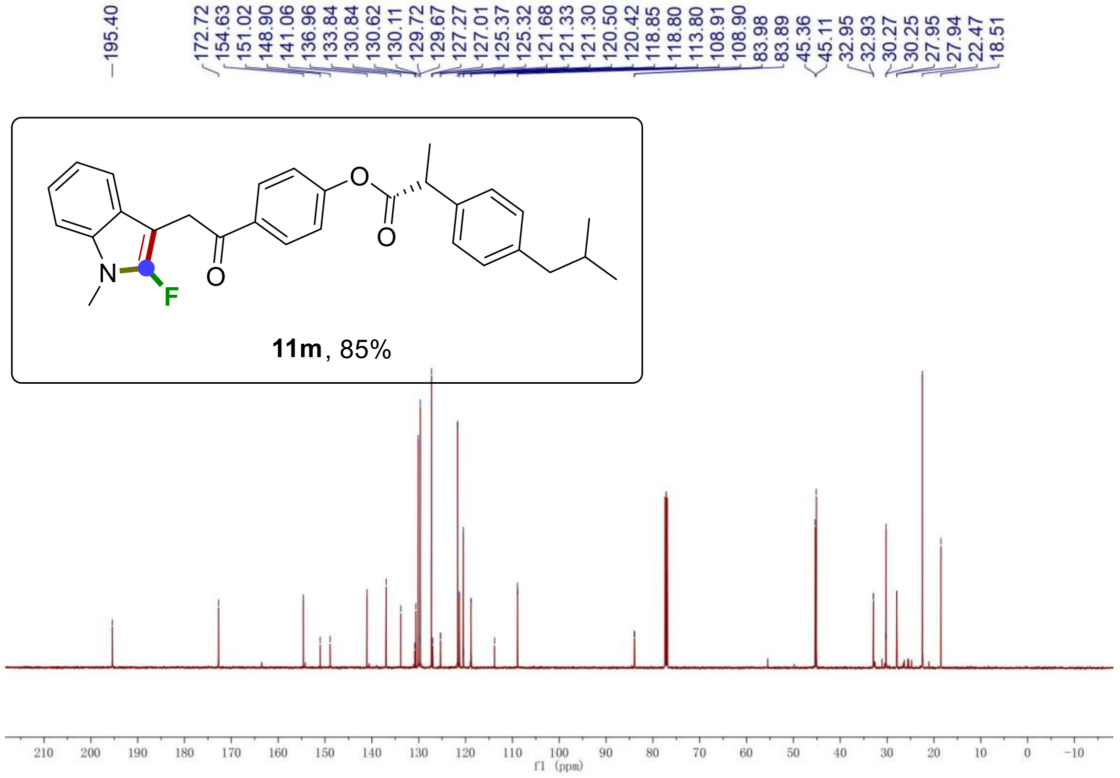

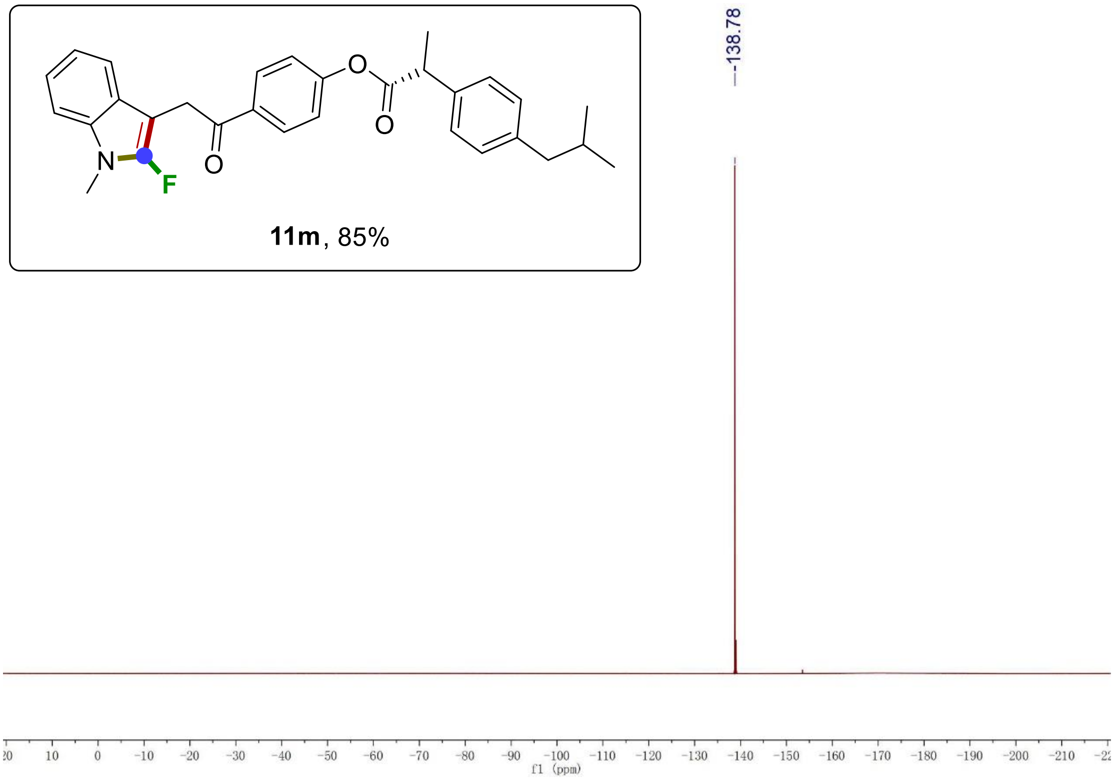


Supplementary Figure 174. $^{1}H$ NMR, $^{13}C$ NMR and $^{19}F$ NMR spectrum of **11m**.

**4-(2-(2-fluoro-1-methyl-1H-indol-3-yl)acetyl)phenyl (R)-2-(6-methoxynaphthalen-2-yl)propanoate (11n)**

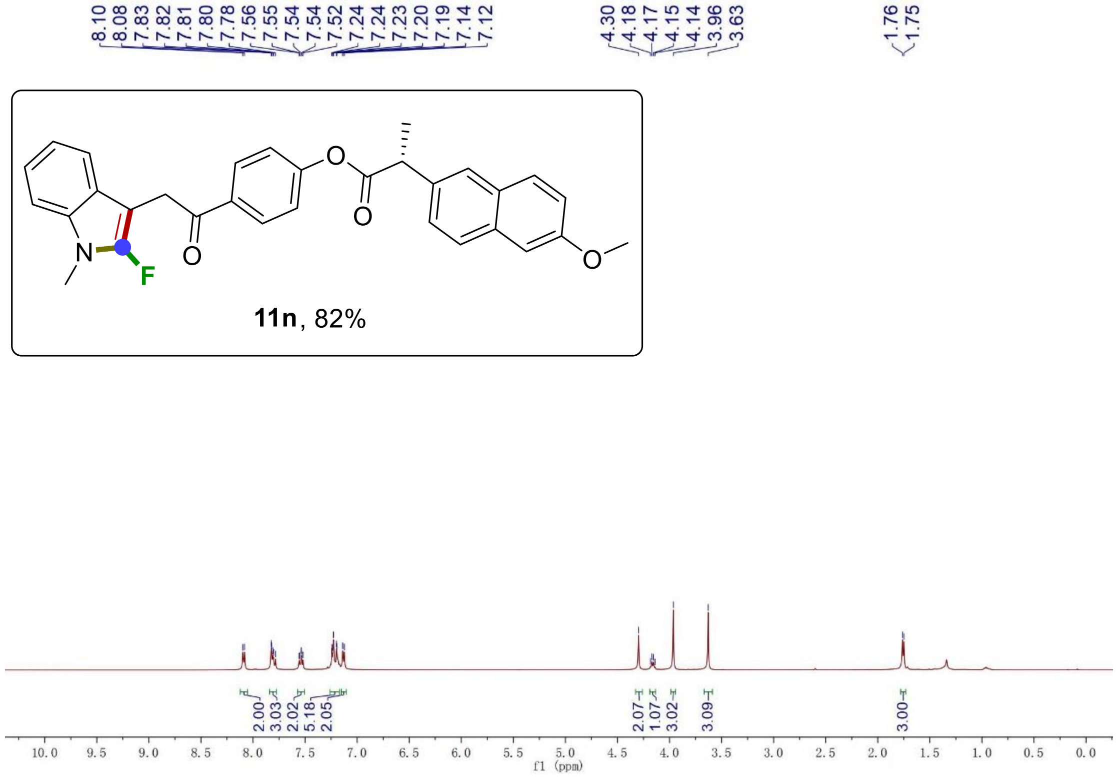


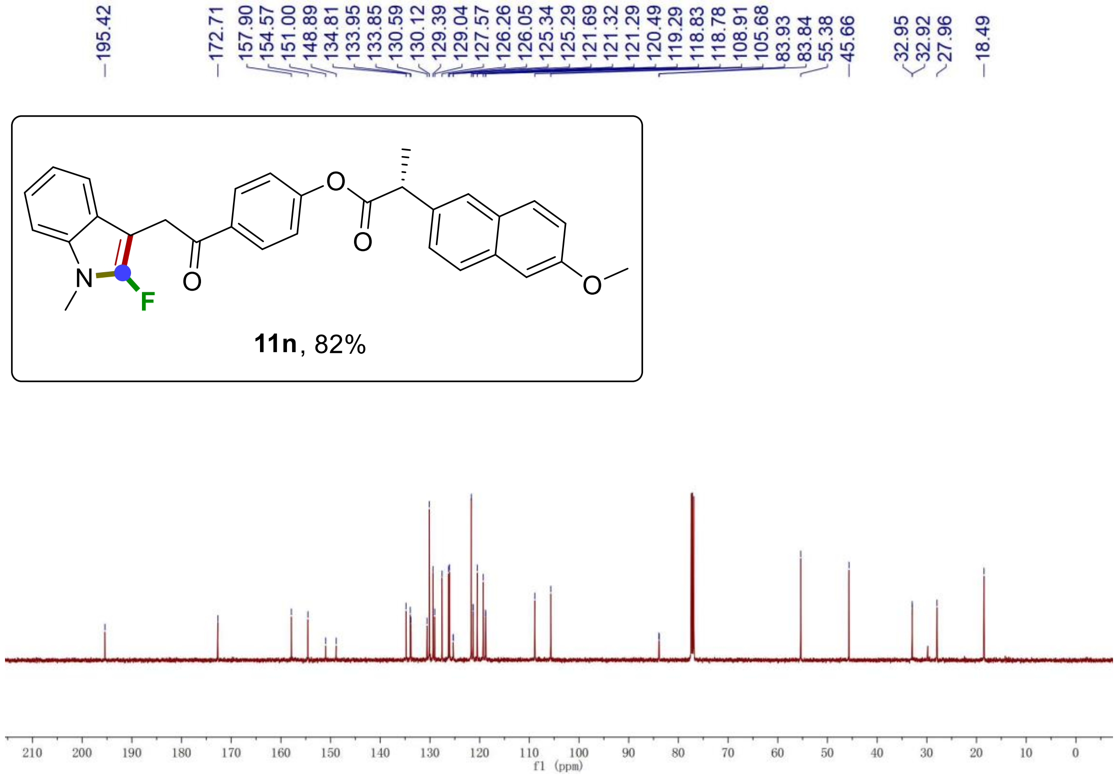

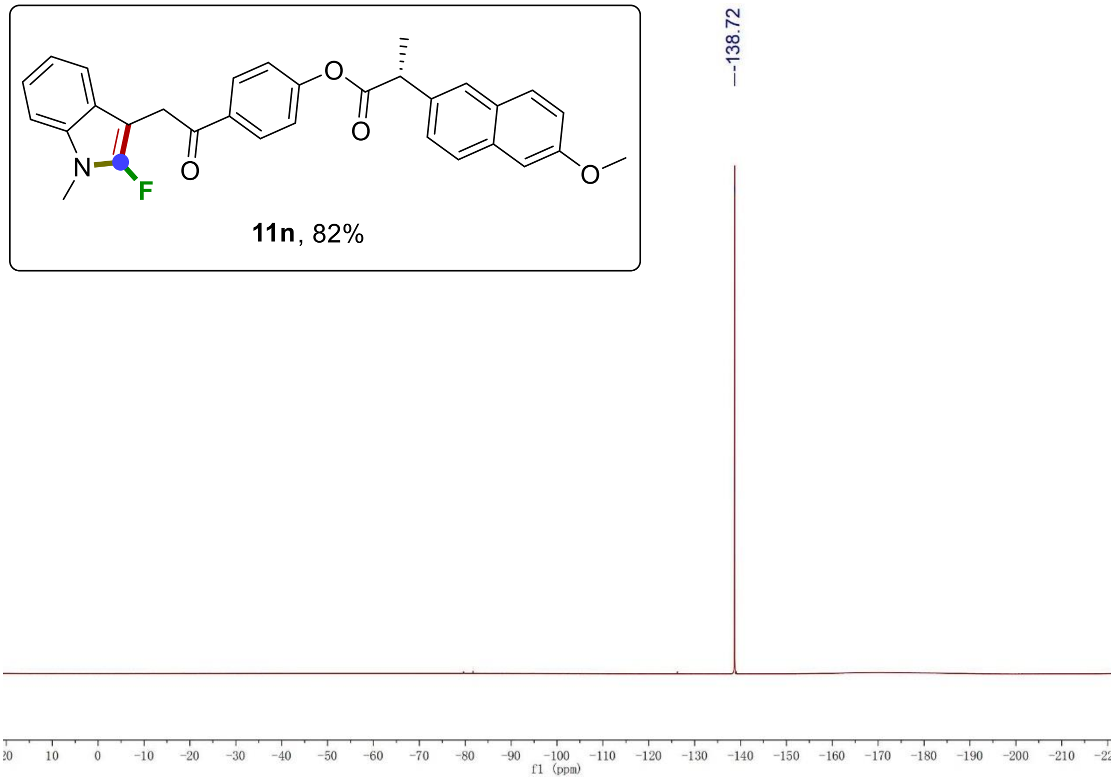


Supplementary Figure 175. $^{1}H$ NMR, $^{13}C$ NMR and $^{19}F$ NMR spectrum of **11n**.

**-(2-(2-fluoro-1-methyl-1H-indol-3-yl)acetyl)phenyl 4-(N,N-dipropylsulfamoyl)benzoate (11o)**

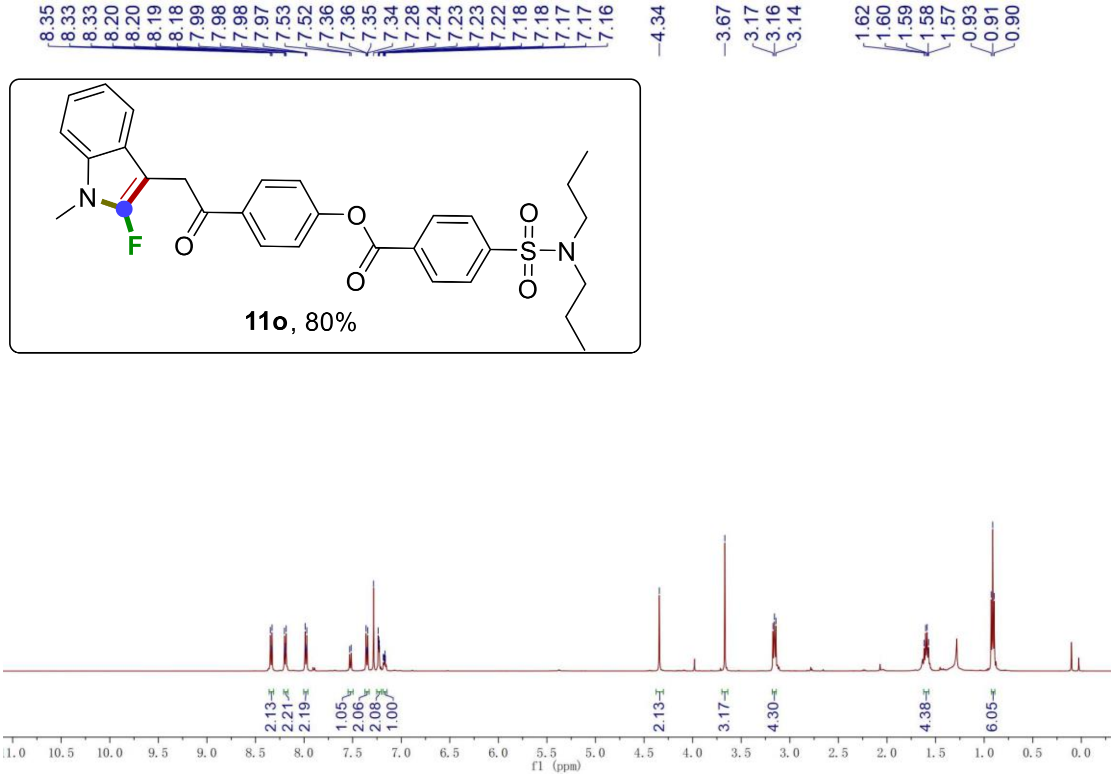


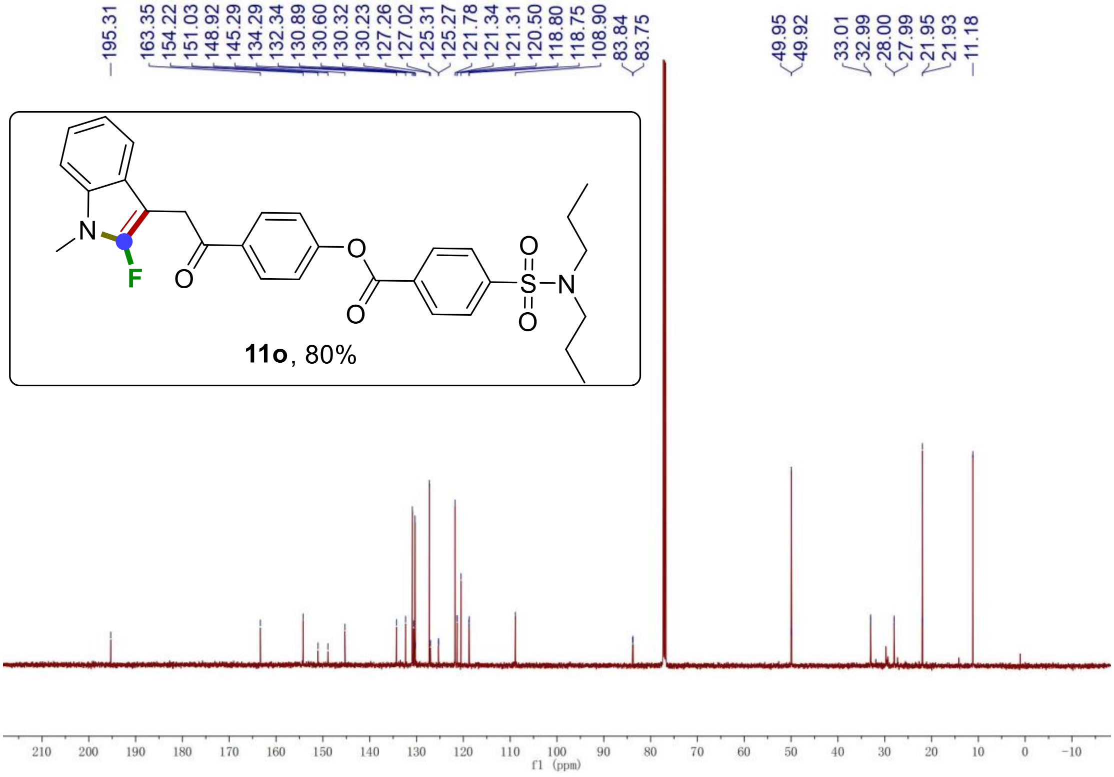

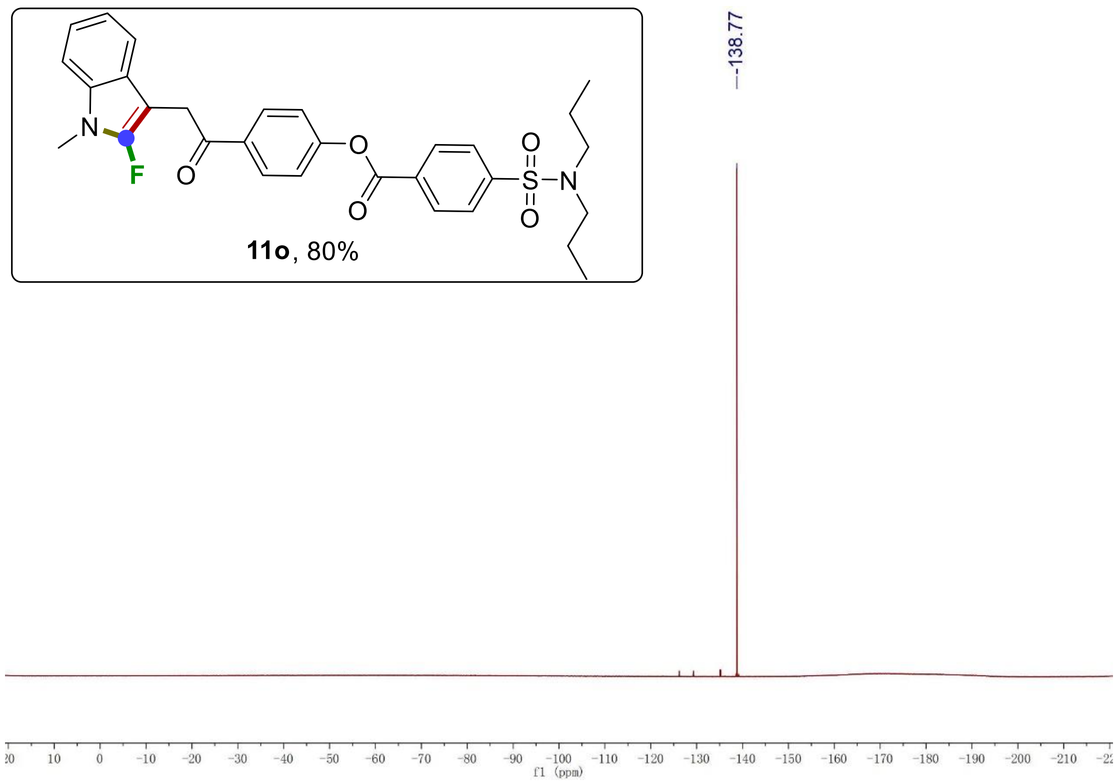


Supplementary Figure 176. $^{1}H$ NMR, $^{13}C$ NMR and $^{19}F$ NMR spectrum of **11o**.

**2-(5-chloro-2-fluoro-1-methyl-1H-indol-3-yl)-1-phenylethan-1-one (13a)**

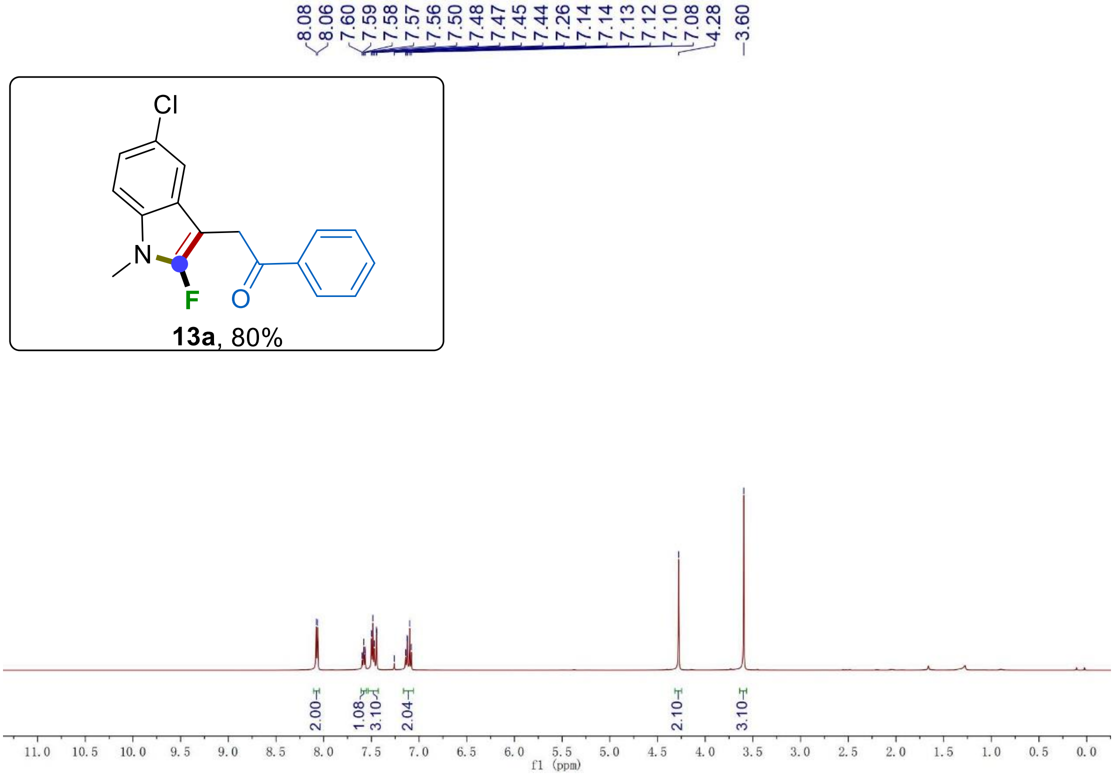


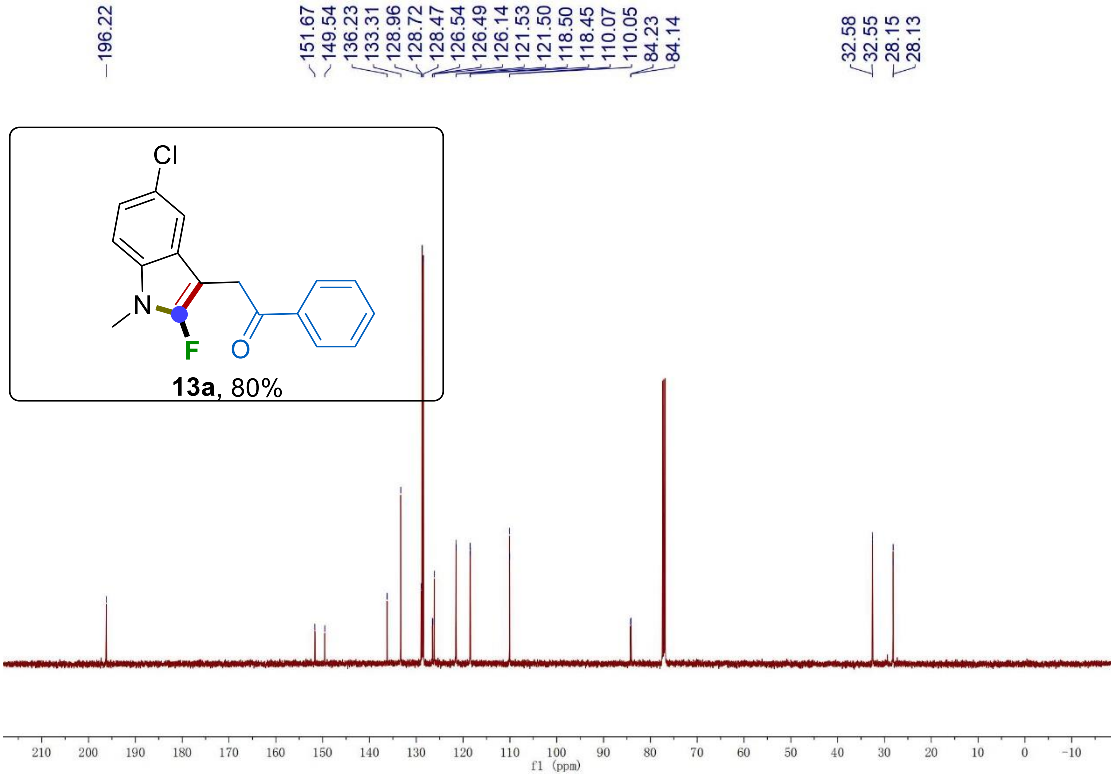

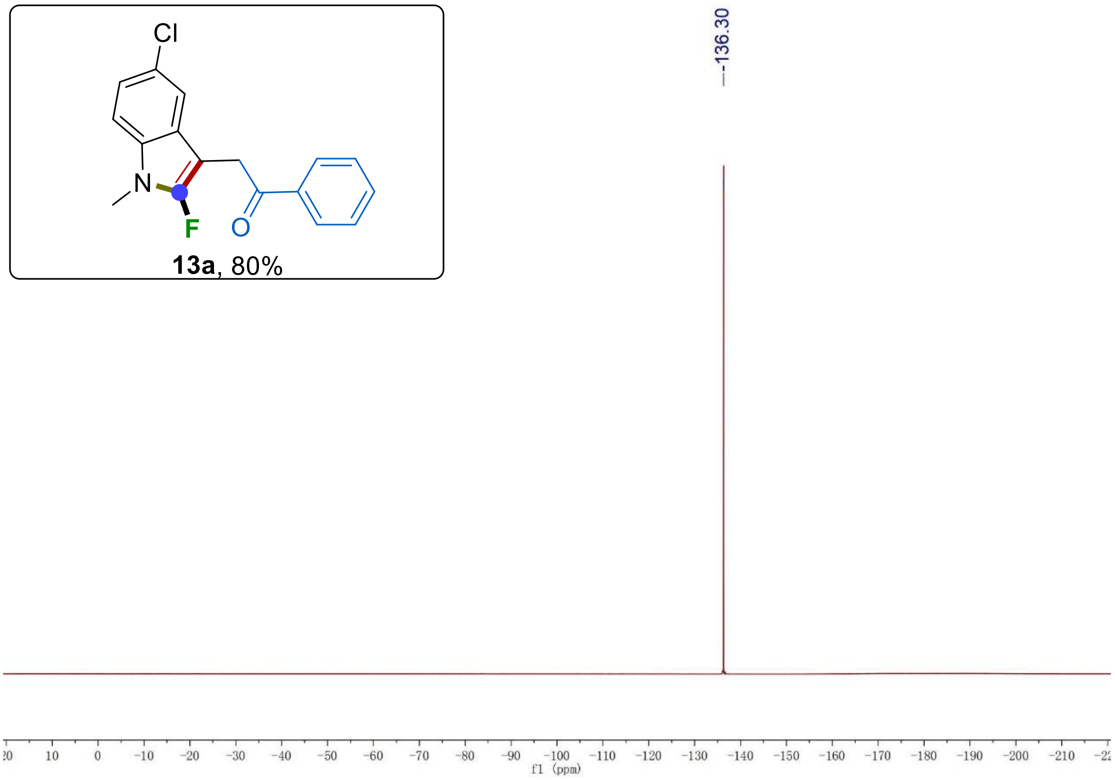


Supplementary Figure 177. $^{1}H$ NMR, $^{13}C$ NMR and $^{19}F$ NMR spectrum of **13a**.

# 2-(5-chloro-2-fluoro-1-methyl-1H-indol-3-yl)-1-(thiophen-2-yl)ethan-1-one (13b)

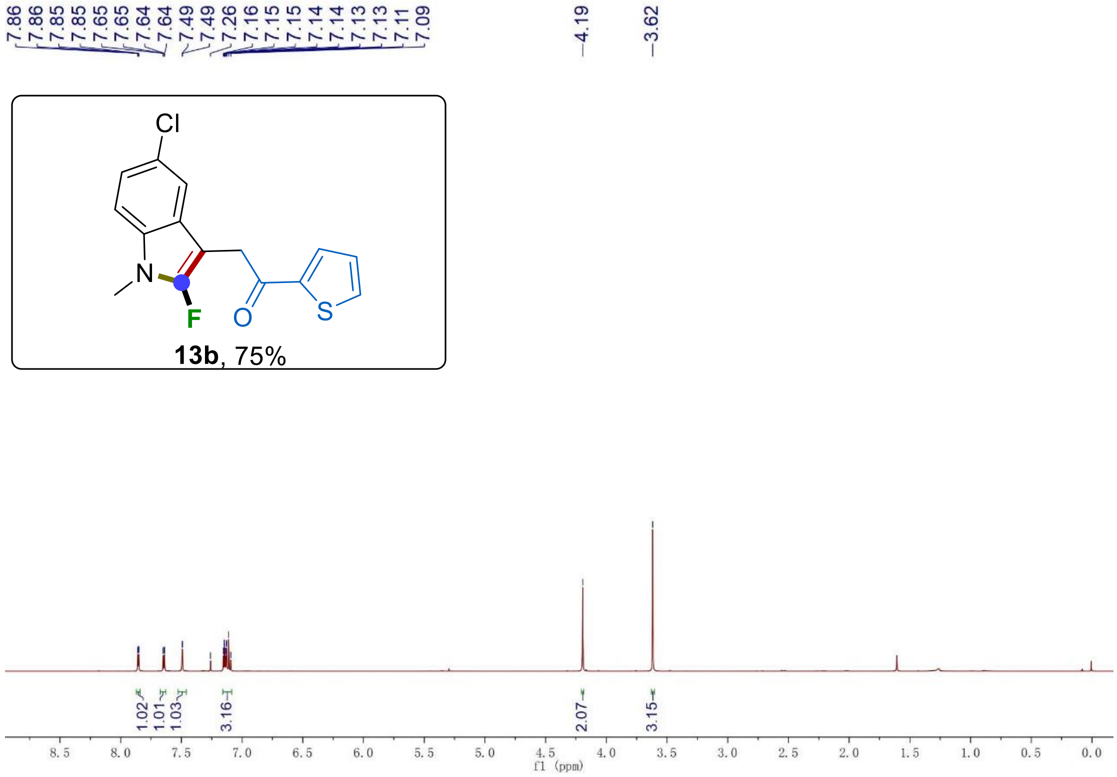


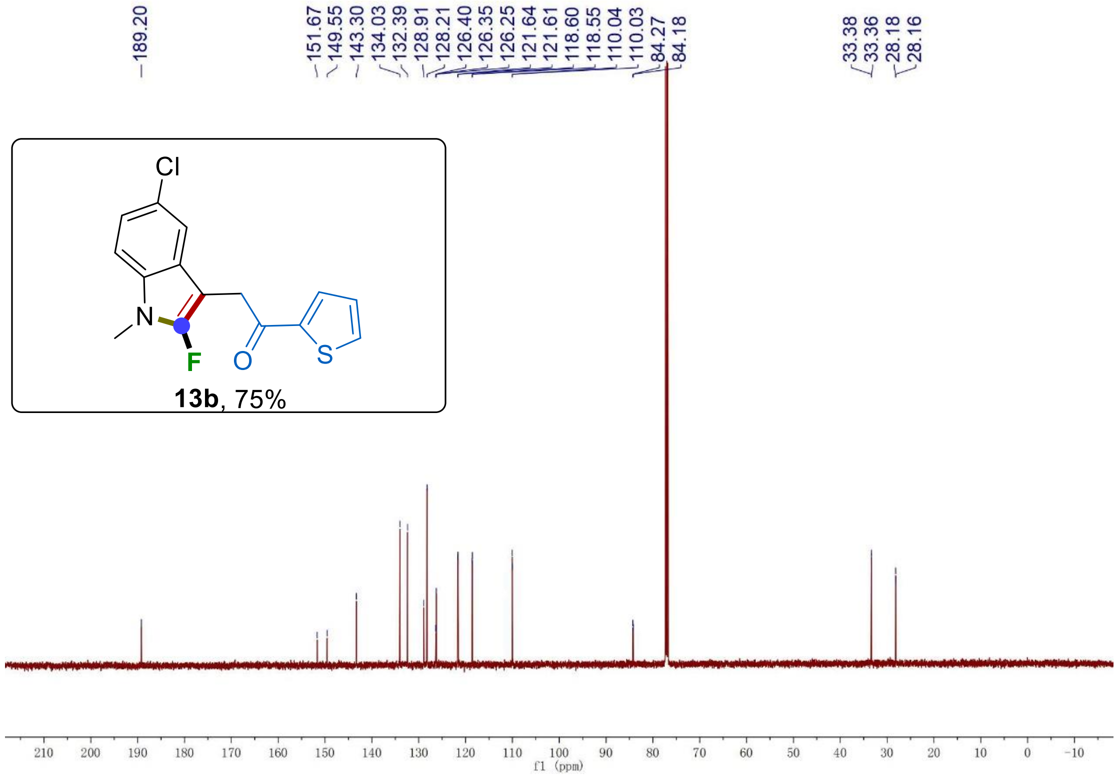

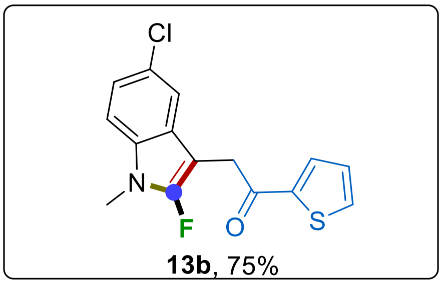


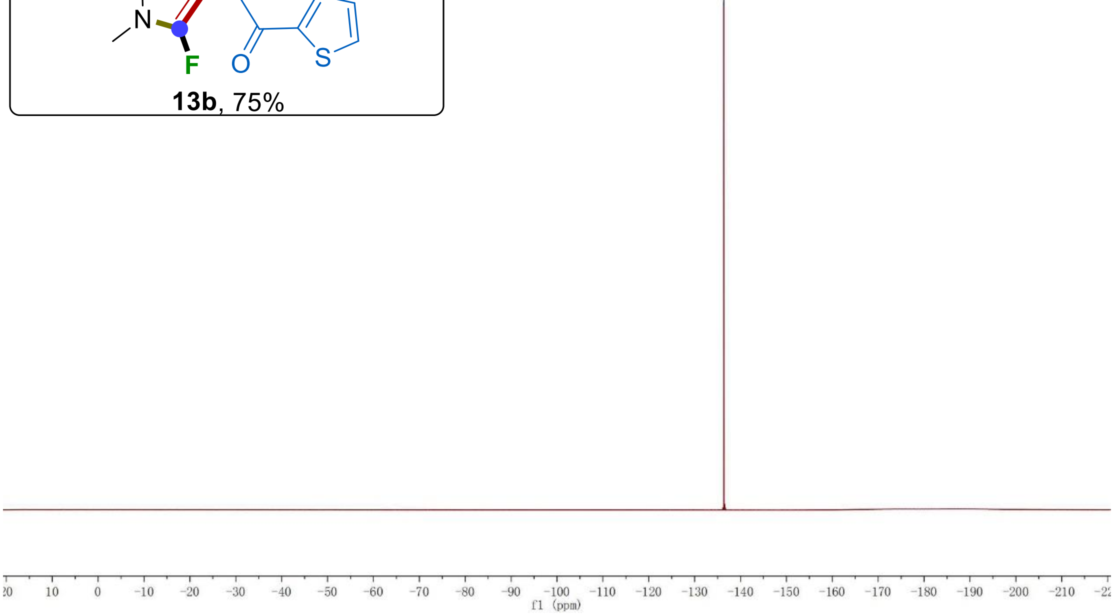


Supplementary Figure 178. $^{1}H$ NMR, $^{13}C$ NMR and $^{19}F$ NMR spectrum of **13b**.

# 1-(5-chloro-2-fluoro-1-methyl-1H-indol-3-yl)-3-methylpentan-2-one (13c)

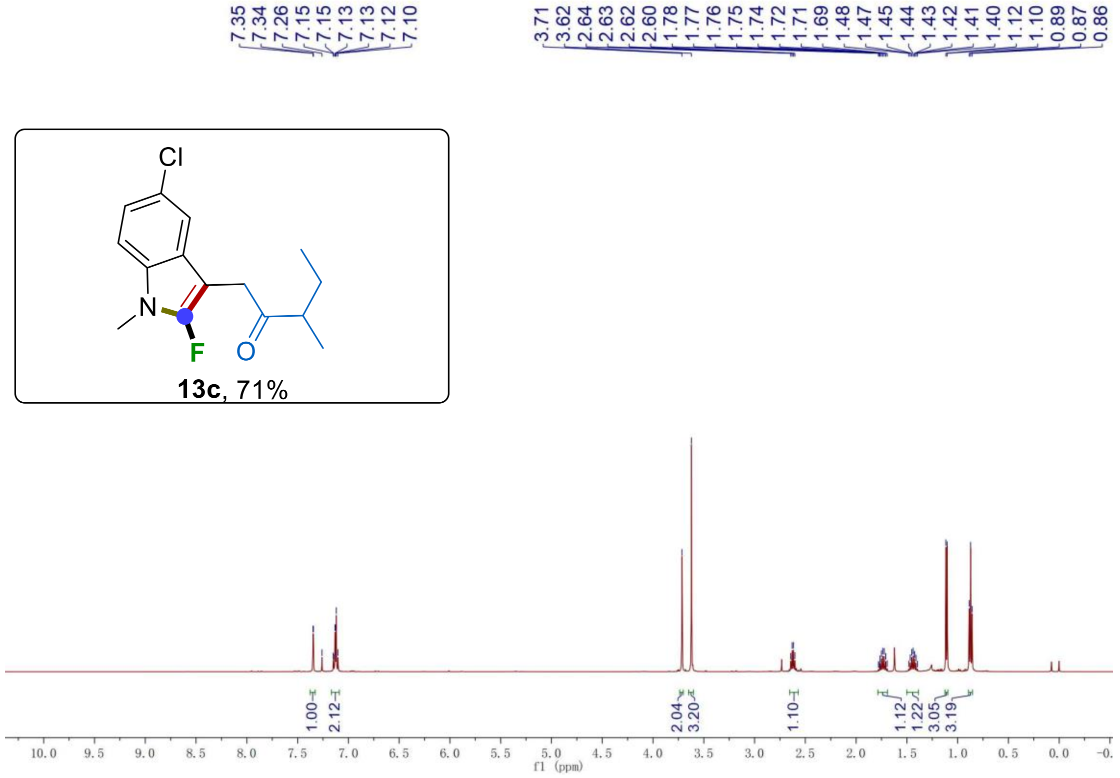


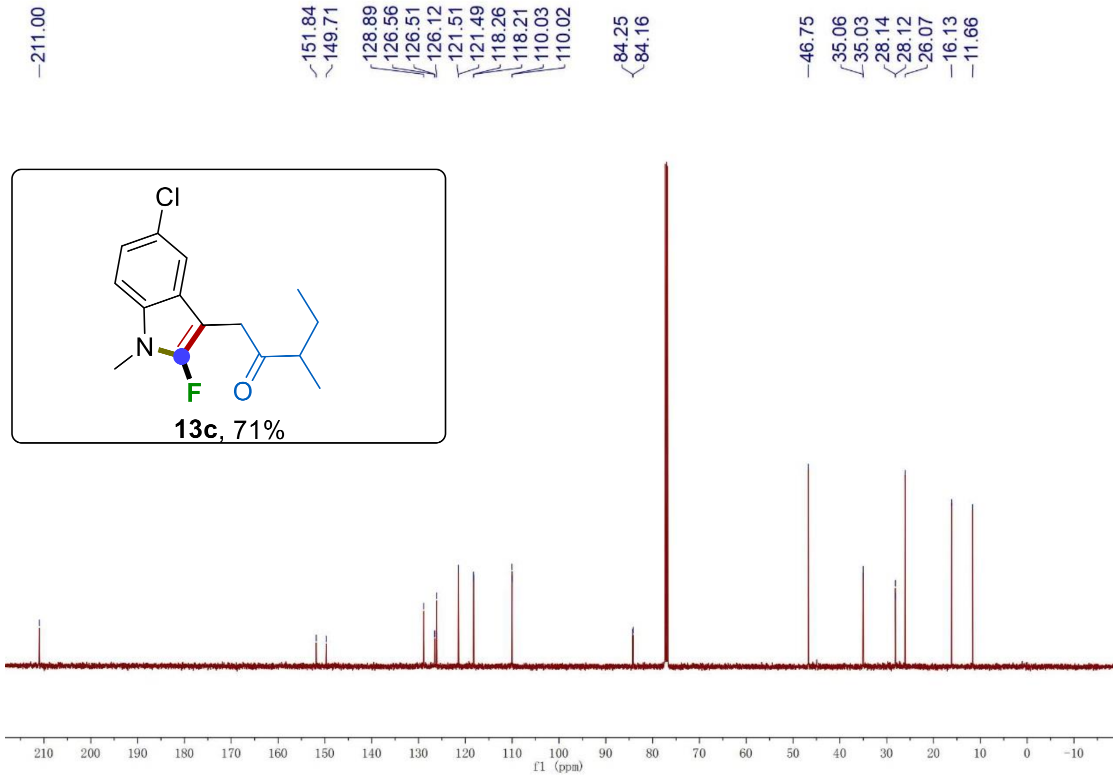

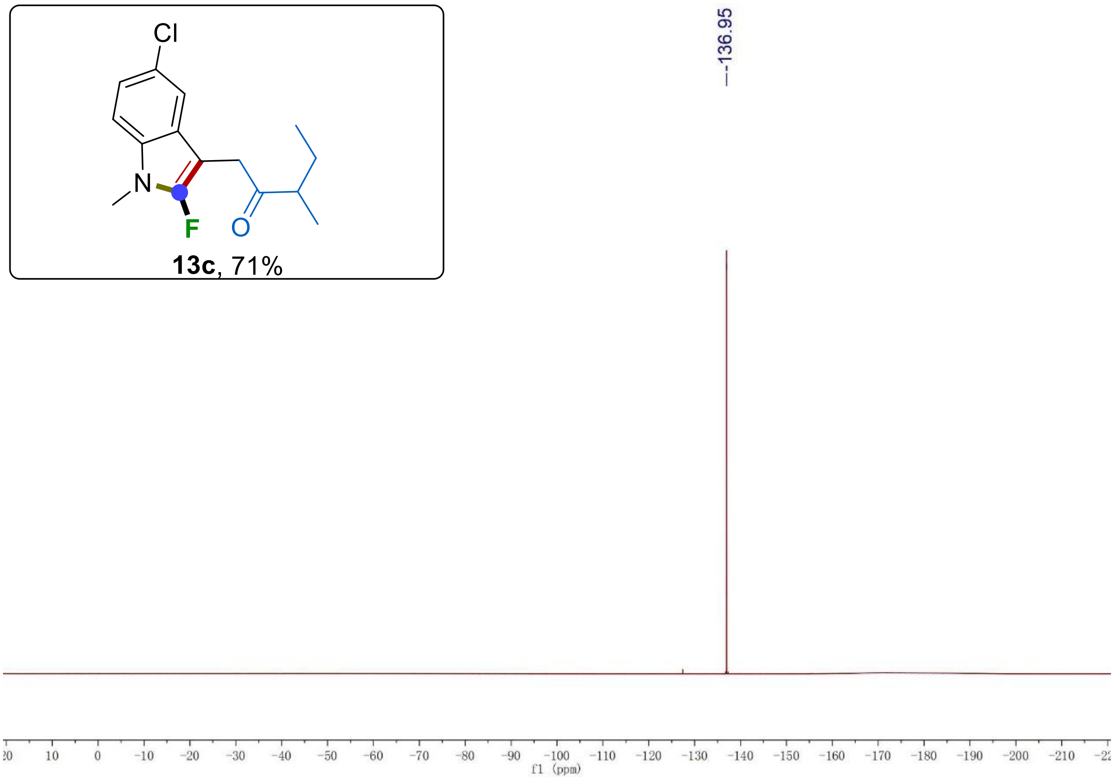


Supplementary Figure 179. $^{1}H$ NMR, $^{13}C$ NMR and $^{19}F$ NMR spectrum of **13c**.

**2-(5-chloro-2-fluoro-1-methyl-1H-indol-3-yl)-1-cyclopropylethan-1-one (13d)**

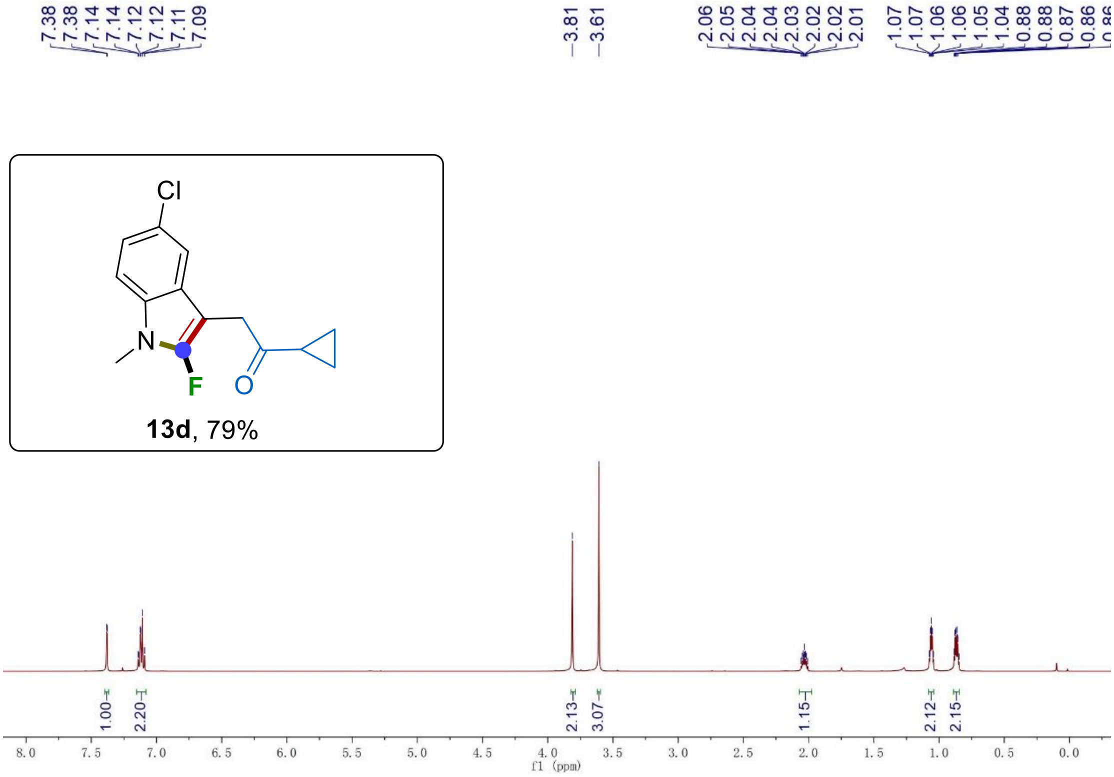

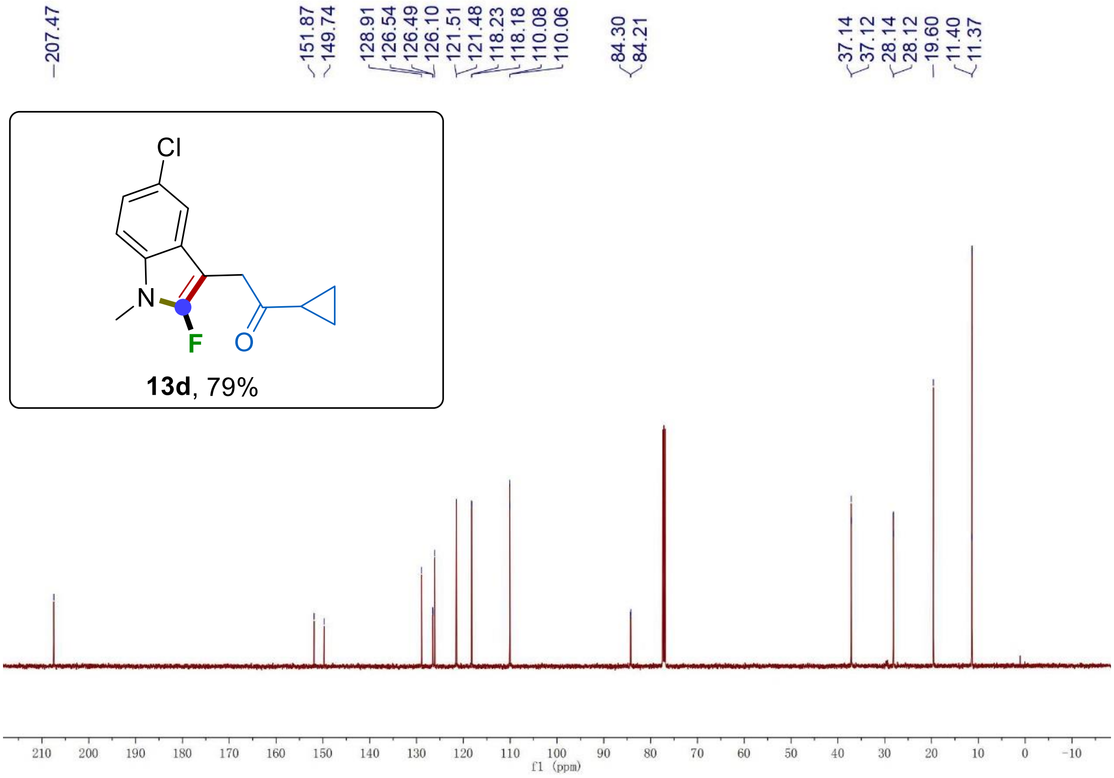

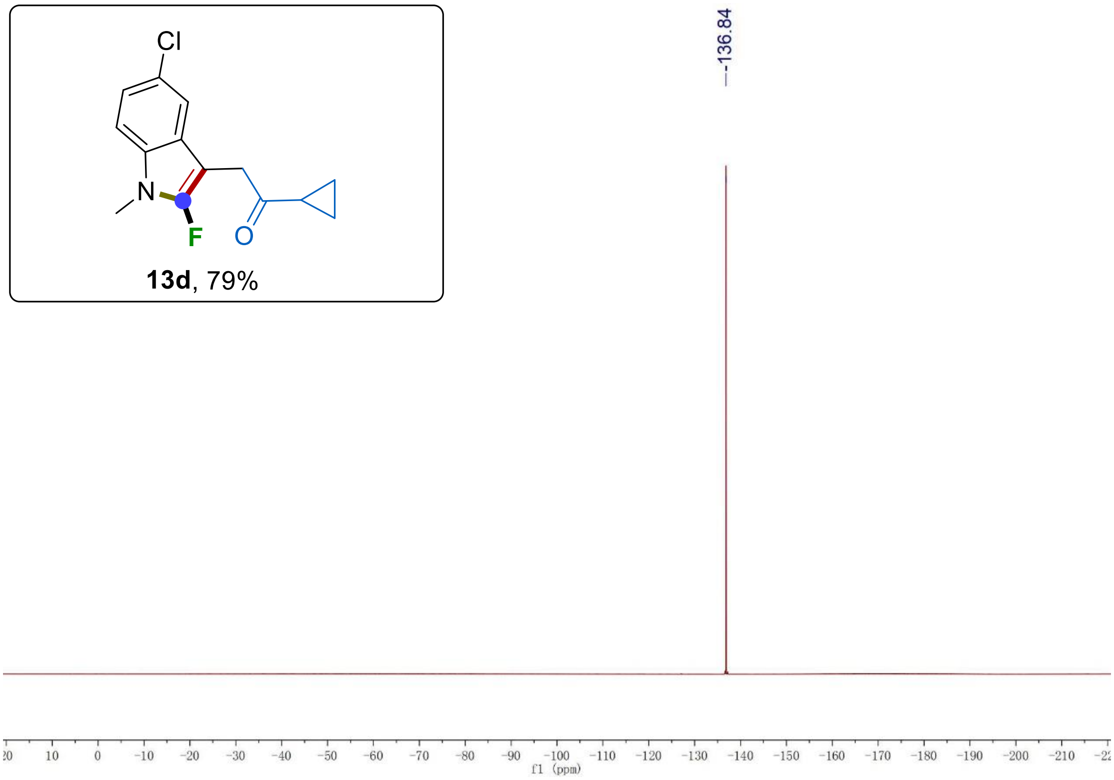

Supplementary Figure 180. $^{1}H$ NMR, $^{13}C$ NMR and $^{19}F$ NMR spectrum of **13d**.

## 2-(5-chloro-2-fluoro-1-methyl-1H-indol-3-yl)-1- ferroceneyl -1-one (13e)

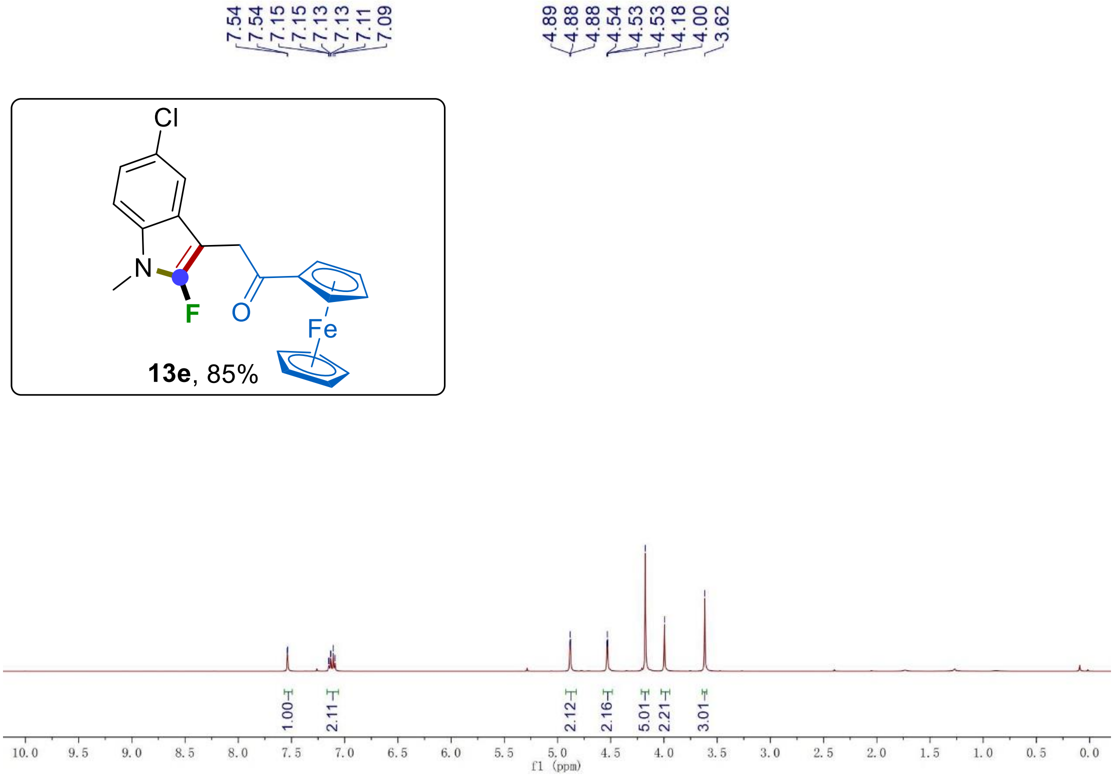


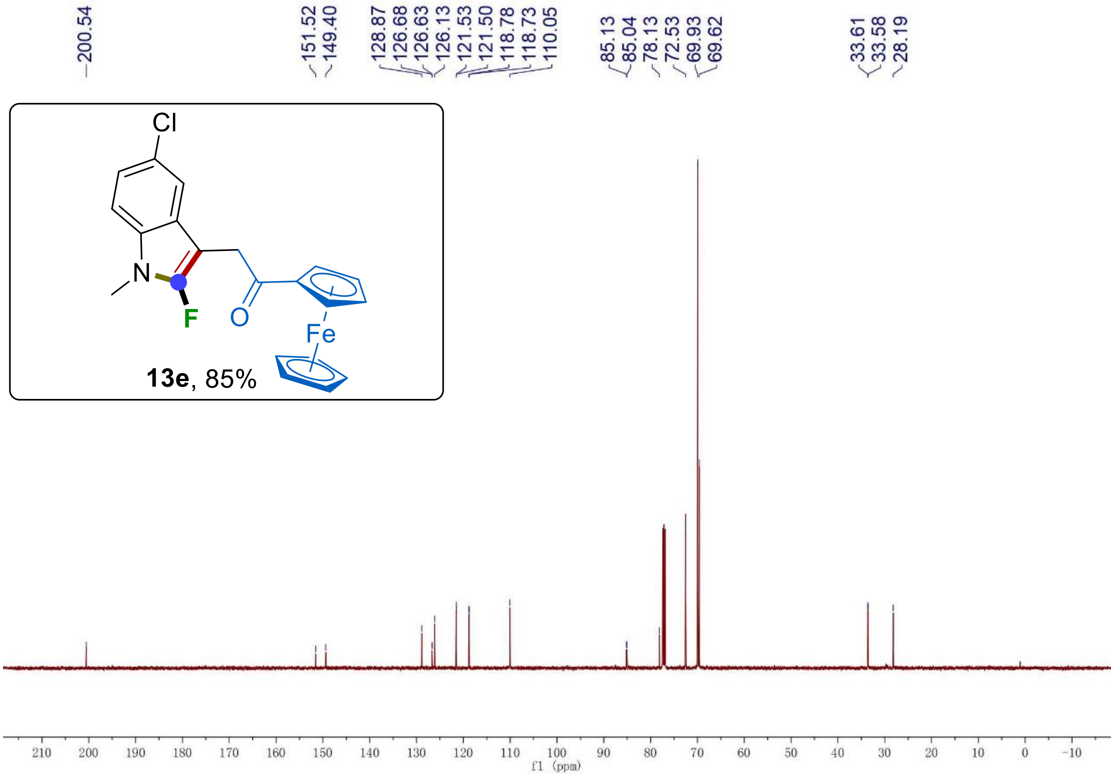

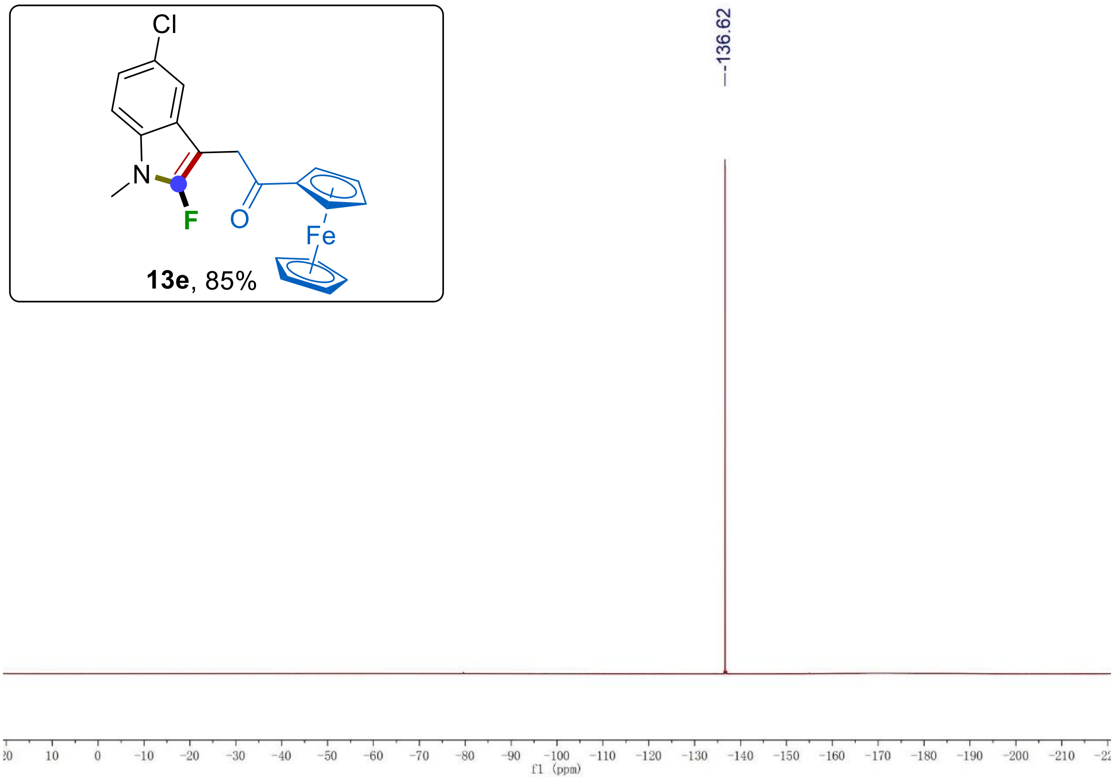


Supplementary Figure 181. $^{1}H$ NMR, $^{13}C$ NMR and $^{19}F$ NMR spectrum of **13e**.

**2-(5-bromo-1-ethyl-2-fluoro-1H-indol-3-yl)-1-phenylethan-1-one (15)**

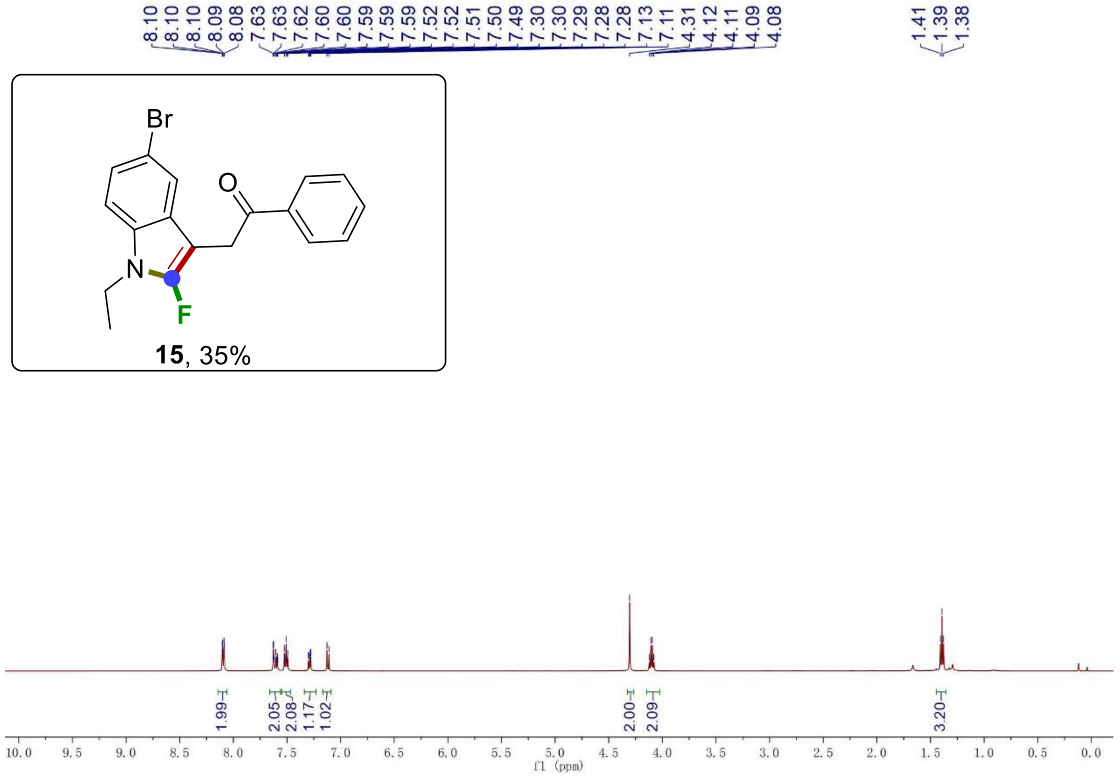


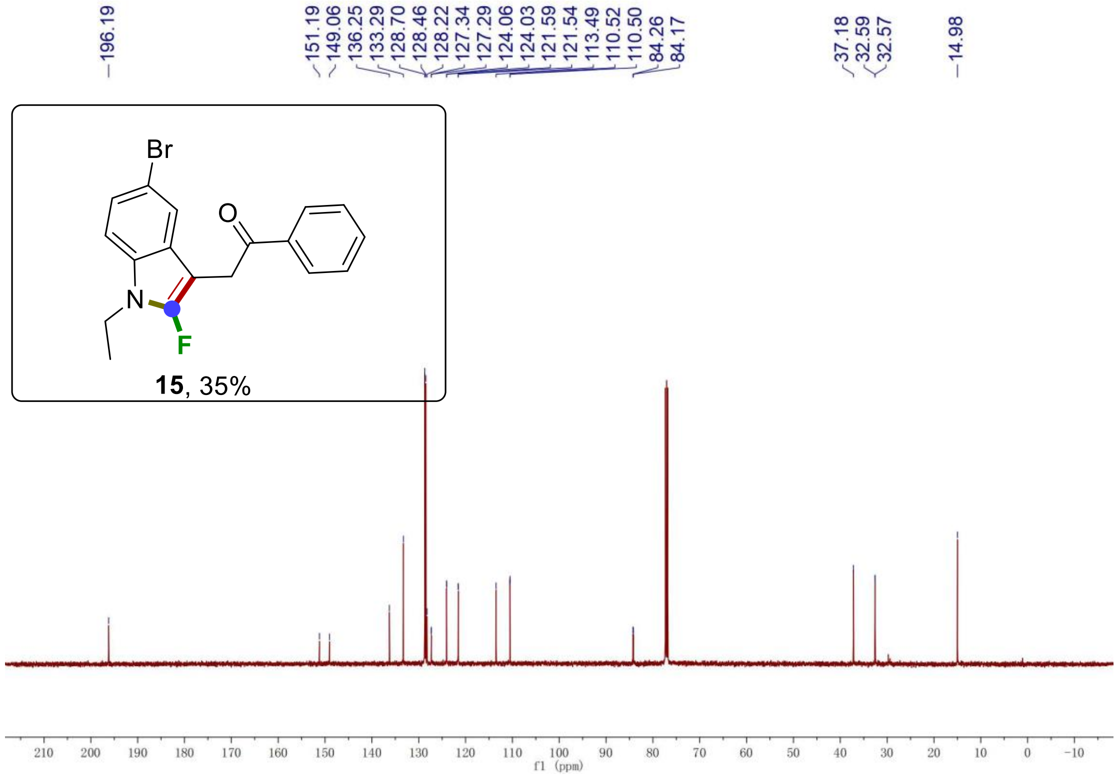

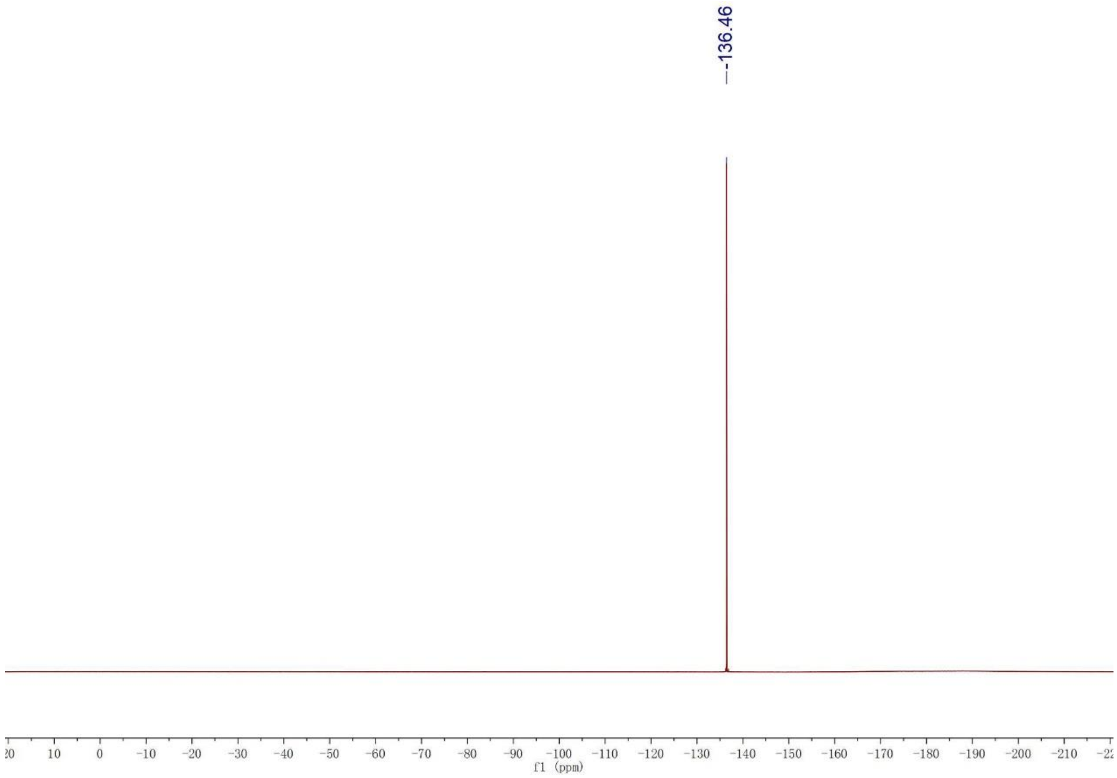


Supplementary Figure 182. $^{1}H$ NMR, $^{13}C$ NMR and $^{19}F$ NMR spectrum of **15**.

**2-(2-fluoro-1-methyl-1H-indol-3-yl)-1-phenylethan-1-one-2-d (3a-D)**

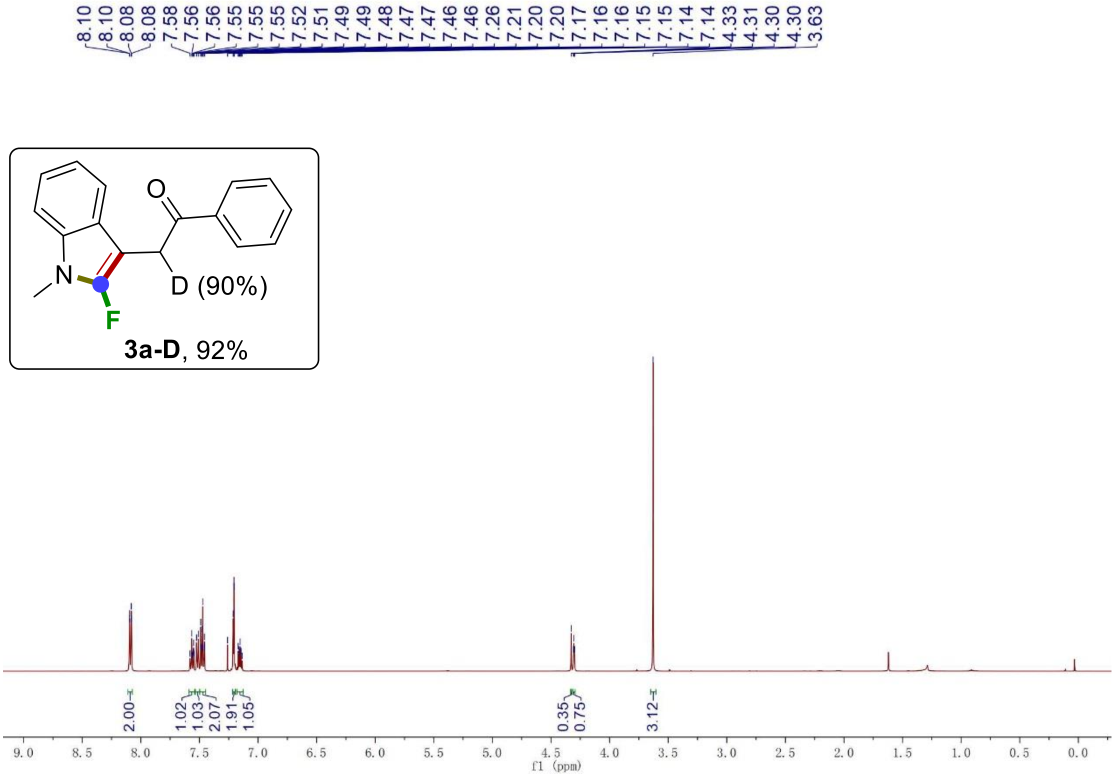


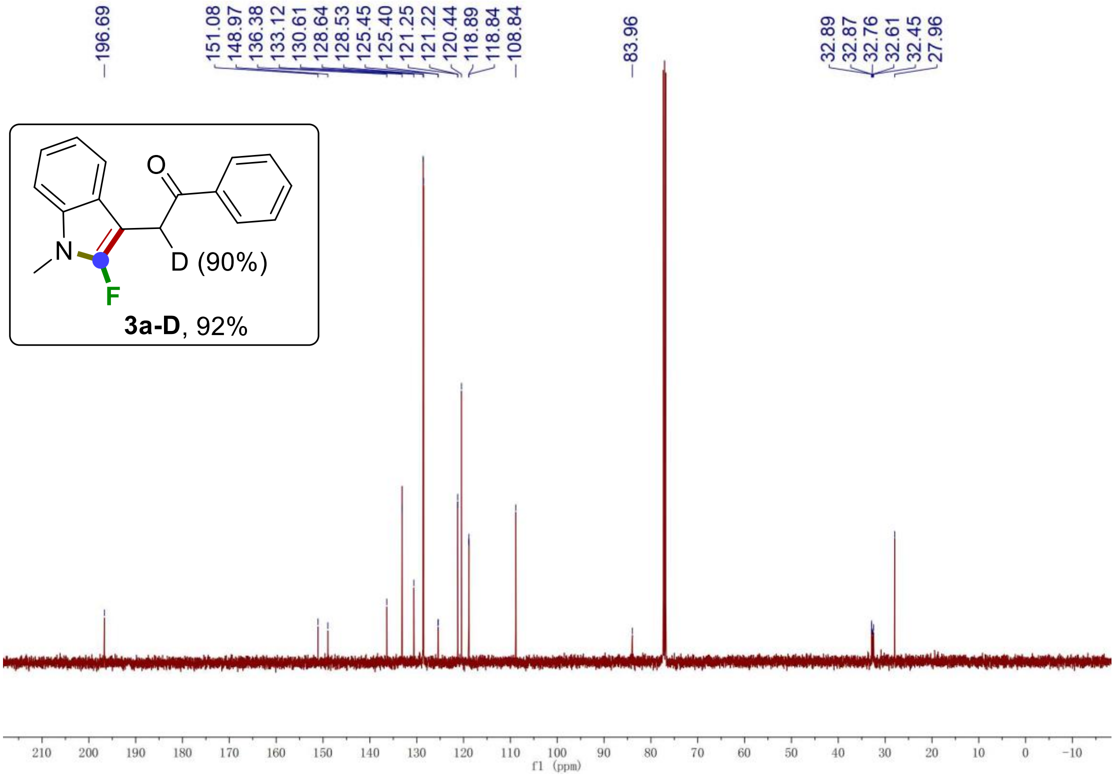

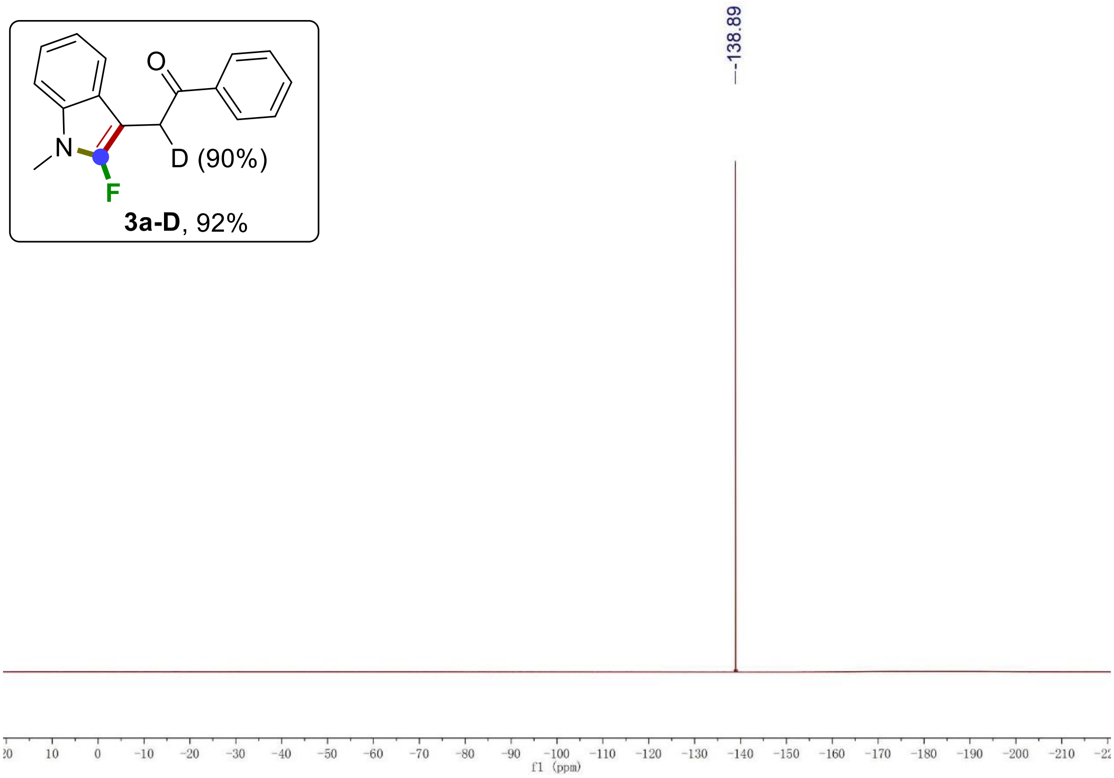


Supplementary Figure 183. $^{1}H$ NMR, $^{13}C$ NMR and $^{19}F$ NMR spectrum of **3a-D**.

**1-(difluoromethyl)-1H-benzo[d]imidazole (19)**

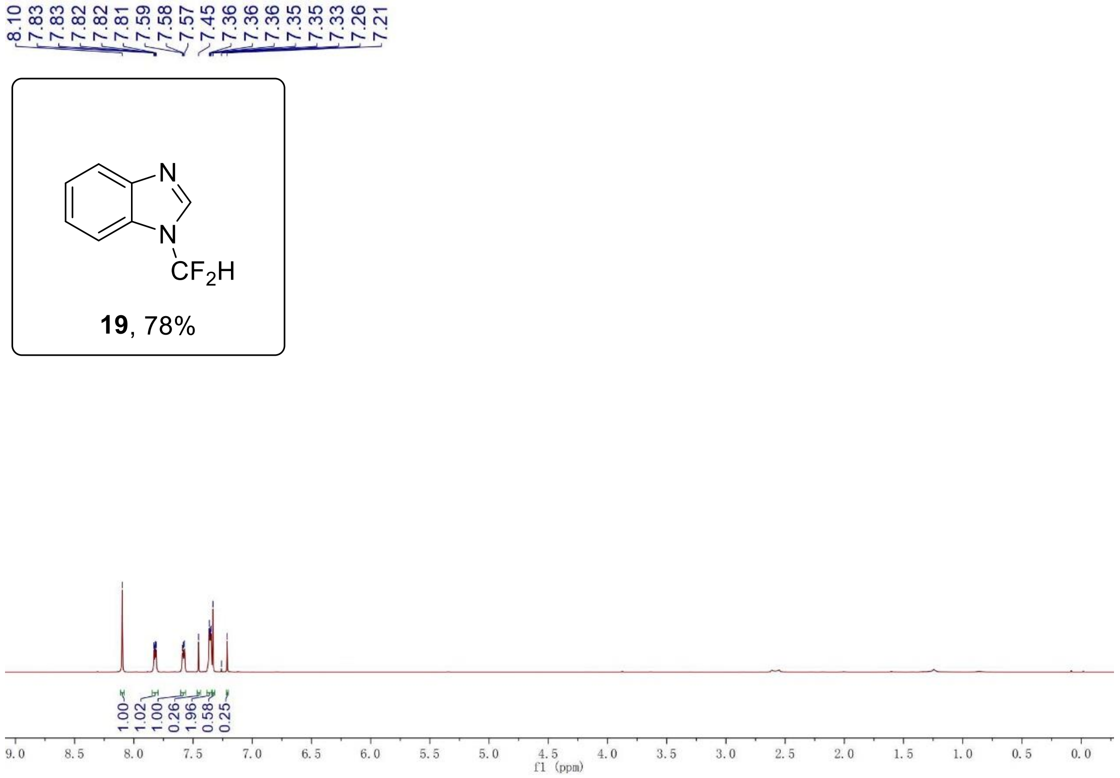


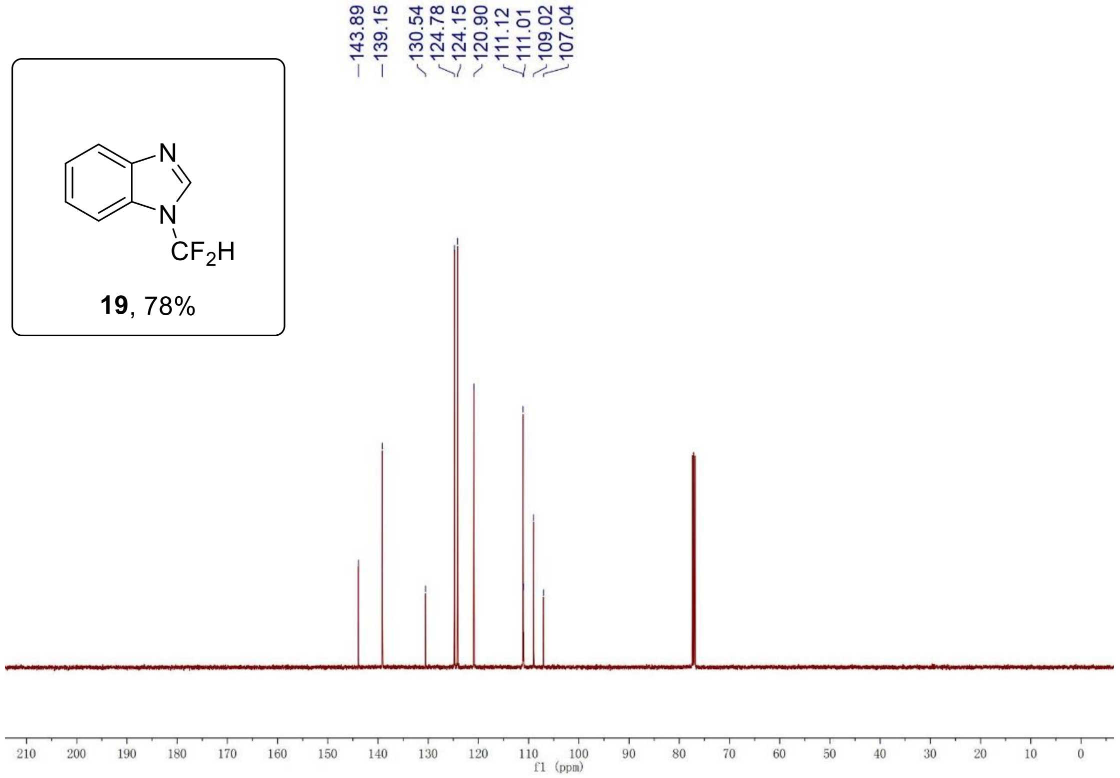

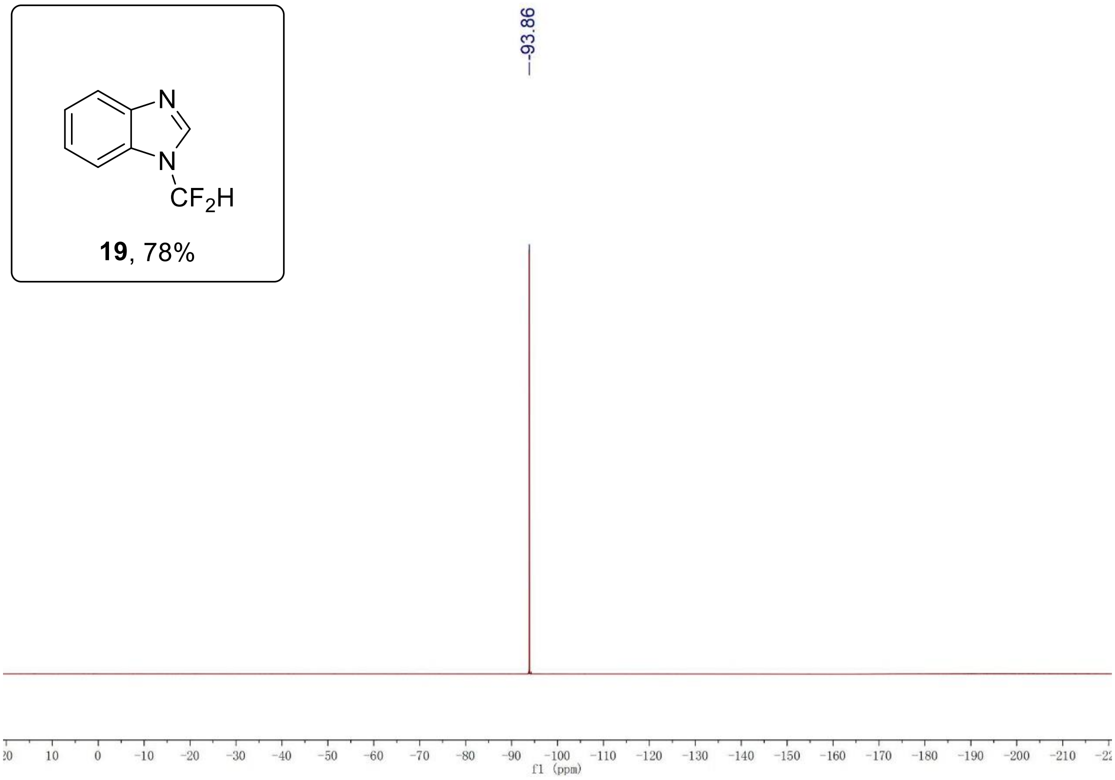


Supplementary Figure 184. $^{1}H$ NMR, $^{13}C$ NMR and $^{19}F$ NMR spectrum of **19**.

## 2-phenylquinoline (22) (CAS: 612-96-4)

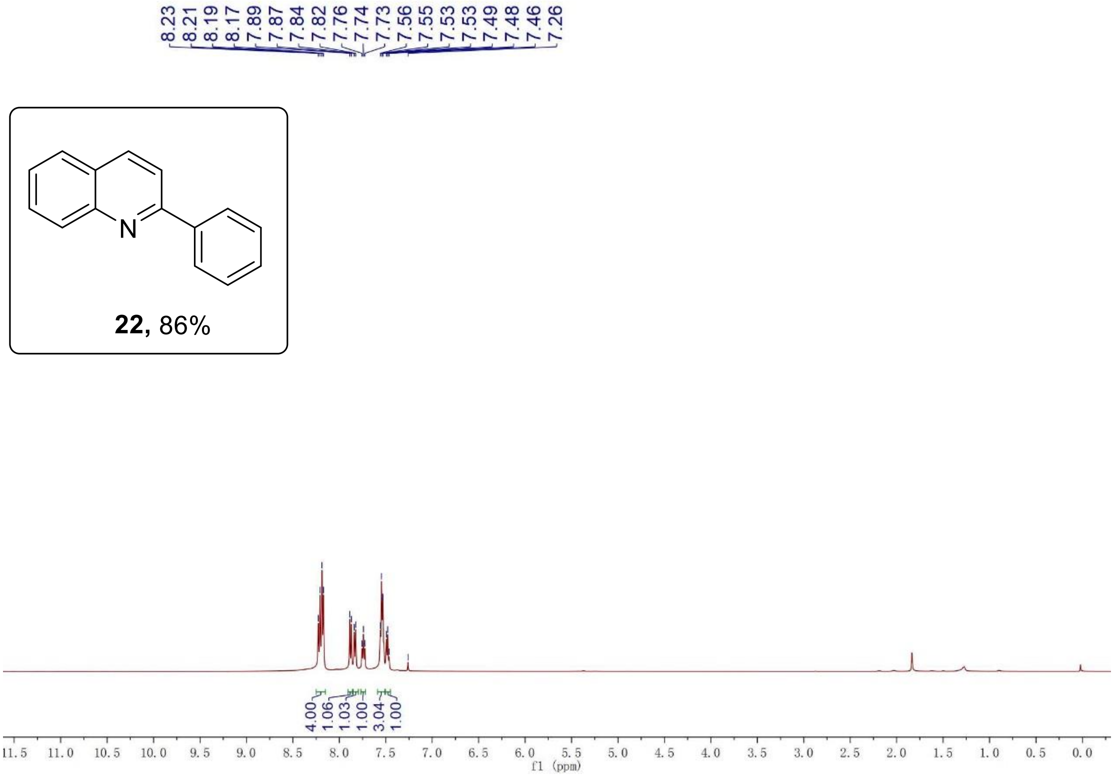


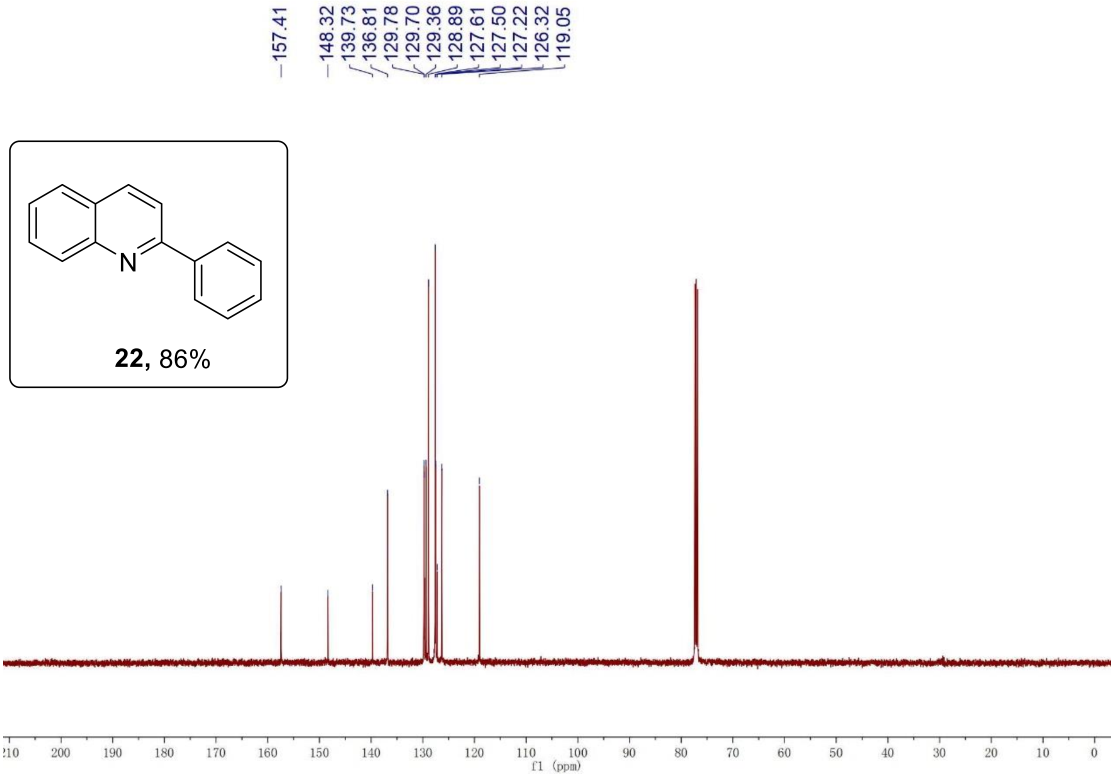


Supplementary Figure 185. $^{1}H$ NMR and $^{13}C$ NMR spectrum of **22**.

## 2.6 References


1. Fereyduni, E.; Sanders, J. N.; Gonzalez, G.; Houk, K. N.; Grenning, A. J. *Chem. Sci.*, **2018**, 9, 8760-8764.
2. Santos, M. S.; Betim, H. L. I.; Kisukuri, C. M.; Delgado, J. A. C.; Corrêa, A. G.; Paixão, M. W. *Org. Lett.* **2020**, *22*, 4266-4271
3. Lou, Y.; Cao, P.; Jia T.; Zhang Y.; Wang, M.; Liao, J. *Angew. Chem. Int. Ed*. **2015**, *54*, 12134-12138.
4. Jin, S.; Kuang, Z.; Song, Q. *Org. Lett.* **2020**, *22,* 615-619.
5. Kim, K. D., Lee, J. H. *Org. Lett.* **2018**, *20*, 7712-7716.
